# Pharmacological treatment for bipolar mania: a systematic review and network meta-analysis of double-blind randomized controlled trials

**DOI:** 10.1038/s41380-021-01334-4

**Published:** 2021-10-12

**Authors:** Taro Kishi, Toshikazu Ikuta, Yuki Matsuda, Kenji Sakuma, Makoto Okuya, Ikuo Nomura, Masakazu Hatano, Nakao Iwata

**Affiliations:** 1 Department of Psychiatry, Fujita Health University School of Medicine, Toyoake, Aichi 470–1192 Japan; 2 Department of Communication Sciences and Disorders, School of Applied Sciences, University of Mississippi, Oxford, MS 38677 USA; 3 Department of Psychiatry, The Jikei University School of Medicine, Minato-ku, Tokyo 105–8461 Japan; 4 Department of Psychiatry, The Moriyama General Mental Hospital, Nagoya, Aichi 463–8570 Japan; 5 Department of Clinical Pharmacy, Fujita Health University School of Medicine, Toyoake, Aichi 470–1192 Japan

**Keywords:** Bipolar disorder, Drug discovery

## Abstract

A systematic review and random-effects model network meta-analysis was conducted to compare the efficacy, acceptability, tolerability, and safety of pharmacological interventions for adults with acute bipolar mania. We searched PubMed, the Cochrane Library, and Embase databases for eligible studies published before March 14, 2021. Randomized controlled trials (RCTs) of oral medication monotherapy lasting ≥10 days in adults with mania were included, and studies that allowed the use of antipsychotics as a rescue medication during a trial were excluded. The primary outcomes were response to treatment (efficacy) and all-cause discontinuation (acceptability). The secondary outcomes were the improvement of mania symptoms and discontinuation due to inefficacy. Of the 79 eligible RCTs, 72 double-blind RCTs of 23 drugs and a placebo were included in the meta-analysis (mean study duration = 3.96 ± 2.39 weeks, *n* = 16442, mean age = 39.55 years, with 50.93% males). Compared with the placebo, aripiprazole, asenapine, carbamazepine, cariprazine, haloperidol, lithium, olanzapine, paliperidone, quetiapine, risperidone, tamoxifen, valproate, and ziprasidone outperformed response to treatment (*N* = 56, *n* = 14503); aripiprazole, olanzapine, quetiapine, and risperidone had lower all-cause discontinuation; however, topiramate had higher all-cause discontinuation (*N* = 70, *n* = 16324). Compared with the placebo, aripiprazole, asenapine, carbamazepine, cariprazine, haloperidol, lithium, olanzapine, paliperidone, quetiapine, risperidone, tamoxifen, valproate, and ziprasidone outperformed the improvement of mania symptoms (*N* = 61, *n* = 15466), and aripiprazole, asenapine, carbamazepine, cariprazine, haloperidol, lithium, olanzapine, paliperidone, quetiapine, risperidone, valproate, and ziprasidone had lower discontinuation due to inefficacy (*N* = 50, *n* = 14284). In conclusions, these antipsychotics, carbamazepine, lithium, tamoxifen, and valproate were effective for acute mania. However, only aripiprazole, olanzapine, quetiapine, and risperidone had better acceptability than the placebo.

## Introduction

Bipolar disorder (BD) is a severe chronic mood disorder characterized by episodes of mania, hypomania, and alternating or intertwining episodes of depression, with a worldwide prevalence of ~1% [1–3]. Acute bipolar mania can be a medical emergency, often leading to psychiatric hospitalization to protect individuals from hyperactive and impulsive activity, and sometimes involving the intervention of law enforcement agencies responding to dangerous behavior [1–3].

Pharmacotherapy is one of the main treatments for acute bipolar mania [1–3]. Recent guidelines recommend various second generation antipsychotics (SGAs), lithium, and valproate as first-line monotherapy for adults with acute mania [4–6]. The acute mania section in these guidelines was developed evidence-based recommendations citing two important network meta-analyses [7, 8]. However, clinical trials of some newer drugs have been conducted for individuals with acute mania after publication of these meta-analyses. Moreover, these network meta-analyses did not evaluate the following important outcomes: clinical remission, efficacy for psychotic symptoms, and the risk of individual adverse events. Therefore, we conducted a systematic review and network meta-analysis for 21 outcomes related to the efficacy, acceptability, tolerability, and safety of 23 drugs in the treatment of adults with acute bipolar mania.

## Materials and methods

This study was conducted according to the Preferred Reporting Items for Systematic Reviews and Meta-Analyses guidelines [9] (Supplementary Table S1) and was registered on the Open Science Framework (https://osf.io/tcd9a/). At least two authors double-checked the literature search, data transfer accuracy, and calculations.

### Search strategy and inclusion criteria

Detailed information about the search strategy is shown in Supplementary Fig. S1. The inclusion criteria for studies were as follows: (1) published and unpublished randomized controlled trials (RCTs) of oral monotherapy lasting for ≥10 days, (2) studies of adults with acute bipolar mania, and (3) double- and single-blind studies. The exclusion criteria were as follows: (1) open-label studies, (2) studies in which selection bias was evaluated as high risk according to the Cochrane risk of bias (ROB) criteria [10], (3) studies including children/adolescents with mania, (4) studies that included individuals with a dual diagnosis of BD and other disorders, (5) studies that allowed antipsychotics as a rescue medication during a trial, and (6) studies that terminated early without efficacy analysis. We searched PubMed, the Cochrane Library, and Embase databases for studies published before March 14, 2021.

### Data synthesis, outcome measures, and data extraction

The primary outcomes for efficacy and acceptability were response to treatment and all-cause discontinuation, respectively. The secondary outcomes were improvement of mania symptoms and discontinuation due to inefficacy. Other outcomes included clinical remission, improvement of psychotic symptoms, discontinuation due to adverse events, discontinuation due to withdrawal consent, depression, and individual adverse events. We targeted outcome assessments at 3 or 4 weeks. For studies without 3- or 4-week data, we used data at the points closest to 3 weeks over 10 days to 12 weeks. All flexible dose studies were included because they allow investigators to titrate to the optimum dose for each individual. Fixed dose studies that used the dose recommended for mania treatment according to recent treatment guidelines were also included [5]. For drugs in which the recommended dose was not stated, we included fixed dose studies that employed clinically used doses [11]. As the therapeutic dose for nonpsychotropic drugs for mania (e.g., tamoxifen) was unknown, all treatment arms of these drugs were included. For studies involving two or more treatment arms of the same drug with different doses, data from the treatment arms were pooled for analysis, provided that they were administered within a therapeutic dose range [5, 11].

The extracted data were analyzed on the basis of intention-to-treat or modified intention-to-treat principles. If required data were missing in the studies, we searched for the data in published systematic review articles. We also attempted to contact the original investigators to obtain unpublished data. While double-blind studies were included to avoid performance and detection bias for subjective outcomes, single-blind studies were included for objective outcomes [12].

### Meta-analysis methods

Both pairwise and frequentist network meta-analyses were performed using the random-effects model [13, 14]. The risk ratio (RR) for dichotomous variables or the standardized mean difference (SMD) for continuous variables was calculated, with 95% confidence intervals (95% CI). Network heterogeneity was assessed using *τ*² statistics. For pairwise meta-analyses, heterogeneity was assessed using *I*^2^ statistics. Statistical evaluation of incoherence was performed using the design-by-treatment test (globally) [15] and the Separate Direct from Indirect Evidence (SIDE) test (locally) [16]. To rank the treatments for each outcome, we used *P*-scores (Supplementary Table S1) [17]. The assumption of transitivity was evaluated by extracting potential effect modifiers (e.g., sample size, duration of study, and mean age; Supplementary Table S2) and comparing their distribution across comparisons in the network. We classified an overall ROB for every RCT based on the individual ROB items (Supplementary Fig. S2) [18]. A meta-regression analysis was performed to determine whether potentially confounding factors (e.g., publication year, mean age, number of total individuals, male individuals [%], and individuals with psychotic features [%]) were associated with the extent of the effect on primary outcomes for efficacy and acceptability. A sensitivity analysis was performed for primary outcomes, in which only half the weight was given to studies (1) with a placebo arm, (2) supported by industry sponsors, (3) without a high-quality design, (4) without 3–4-week data, (5) including individuals with rapid-cycling, (6) including individuals with mixed state/episode, (7) with a low-dose arm, and (8) that did not use common definition of response to treatment (The common definition is ≥50% improvement in the mania rating scale score; this analysis was performed for the primary efficacy outcome only. Supplementary  Appendix S1) [19]. Moreover, we performed additional network meta-analyses for the response to treatment along the time course lasting for 7–10 days, 3 weeks, 4–6 weeks, and 8–12 weeks to examine when the antimanic effects of these drugs appeared. Funnel plots were created to explore potential publication bias. Finally, the results were incorporated into the Confidence in Network Meta-Analysis (CINeMA) application, an adaptation of the Grading of Recommendations Assessment, Development, and Evaluation approach, to assess the credibility of the findings of each of the network meta-analyses [20–22].

## Results

### Study characteristics

A flowchart of the literature search and a detailed explanation of the process are shown in Supplementary Fig. S1. Of the 13489 articles initially identified, 3572 were duplicates, 9835 were excluded after reviewing the titles and abstracts, and 10 were excluded after reviewing the full texts. In total, 72 articles on eligible studies were selected, and 2 articles were detected from previous review articles. Two articles each included data from two RCTs [23, 24], and one article included data from four RCTs [25]. Of 79 eligible RCTs, 5 single-blind RCTs did not report available data for performing a meta-analysis regarding objective outcomes [26–30]. Two double-blind RCTs did not report any available data for performing a meta-analysis [31, 32]. Finally, 72 double-blinded RCTs (*n* = 16442, males = 50.93%, mean age = 39.55 years, mean study duration = 3.96 ± 2.39 weeks) were included with the following treatment arms (number of studies (*N*)/individuals (*n*)): aripiprazole (9/1205), asenapine (3/620), brexpiprazole (2/321), carbamazepine (6/305), cariprazine (3/612), chlorpromazine (1/10), endoxifen (2/55), eslicarbazepine (2/148), haloperidol (10/1023), lamotrigine (3/173), licarbazepine (1/324), lithium (20/965), olanzapine (14/1565), oxcarbazepine (1/30), paliperidone (2/542), quetiapine (5/630), risperidone (7/676), tamoxifen (2/43), topiramate (4/659), valnoctamide (1/71), valproate (14/981), verapamil (1/17), ziprasidone (3/458), and a placebo (48/5009). The study characteristics are summarized in Supplementary Table S2. In addition, 56 studies were industry sponsored; 14 included individuals with rapid-cycling, and 26 excluded these individuals; 38 included individuals with mixed state/episode, and 11 excluded these individuals; and 35 included individuals with psychosis, and 4 excluded these individuals. While 21 studies were evaluated as low overall ROB, other studies were evaluated as moderate overall ROB (Supplementary Fig. S2).

### Network meta-analysis results

The network meta-analysis results are shown in Supplementary  Appendixes S1–S21.

### Response to treatment

**Fig. 1:**
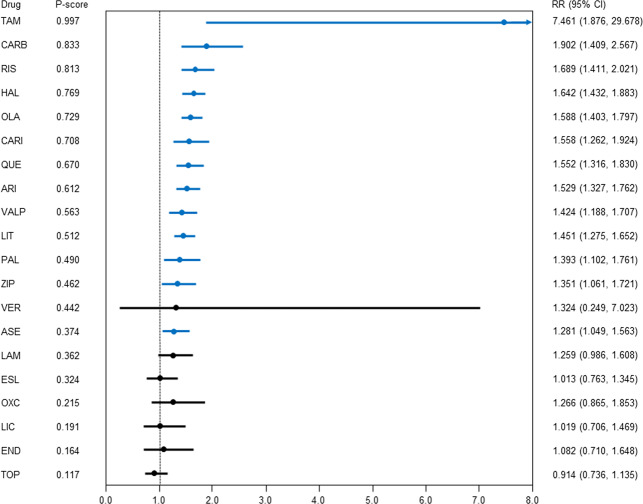
Response to treatment. Drugs were compared with the placebo. Colors indicate the presence or absence of a significant difference: blue, the drug was superior to the placebo; black, the drug was similar to the placebo. 95% CI 95% confidence interval, ARI aripiprazole, ASE asenapine, CARB carbamazepine, CARI cariprazine, END endoxifen, ESL eslicarbazepine, HAL haloperidol, LAM lamotrigine, LIC licarbazepine, LIT lithium, OLA olanzapine, OXC oxcarbazepine, PAL paliperidone, QUE quetiapine, RIS risperidone, RR risk ratio, TAM tamoxifen, TOP topiramate, VALP valproate, VER verapamil, ZIP ziprasidone.

Aripiprazole, asenapine, carbamazepine, cariprazine, haloperidol, lithium, olanzapine, paliperidone, quetiapine, risperidone, tamoxifen, valproate, and ziprasidone showed a better response to treatment than the placebo (*N* = 56, *n* = 14503; Fig. 1, Table 1); the RR (95% CI) ranged from 7.461 (1.876, 29.678) for tamoxifen to 1.281 (1.049, 1.563) for asenapine. Aripiprazole, cariprazine, and quetiapine outperformed eslicarbazepine, licarbazepine, and topiramate; asenapine, lamotrigine, paliperidone, and ziprasidone outperformed topiramate; carbamazepine outperformed asenapine, endoxifen, eslicarbazepine, lamotrigine, licarbazepine, and topiramate; haloperidol, olanzapine, and risperidone outperformed asenapine, eslicarbazepine, licarbazepine, and topiramate; lithium and valproate outperformed eslicarbazepine and topiramate; and tamoxifen outperformed all active-drugs other than carbamazepine and verapamil. Global heterogeneity was low, and the network did not show significant global inconsistency. There was statistical agreement between direct and indirect estimates, except for three comparisons: aripiprazole vs. placebo (aripiprazole outperformed the placebo in both direct and indirect comparisons), paliperidone vs. quetiapine (quetiapine outperformed paliperidone in the indirect comparison but not in the direct comparison), and ziprasidone vs. placebo (ziprasidone outperformed the placebo in the direct comparison but not the indirect comparison). No comparisons included at least 10 studies.

**Table 1:** Head-to-head comparisons for response to treatment (left lower half) and all-cause discontinuation (right upper half).

ARI	0.841 (0.628, 1.127)	0.749 (0.494, 1.136)	0.993 (0.729, 1.352)	0.834 (0.609, 1.142)	0.316 (0.014, 7.087)	0.340 (0.034, 3.387)	1.035 (0.508, 2.107)	0.948 (0.767, 1.172)	0.754 (0.533, 1.067)	0.715 (0.450, 1.136)	0.816 (0.659, 1.009)	**1.297 (1.046, 1.609)**		1.103 (0.779, 1.562)	1.106 (0.829, 1.477)	1.074 (0.809, 1.425)	1.100 (0.515, 2.347)	**0.629 (0.467, 0.847)**	**0.479 (0.262, 0.876)**	0.989 (0.791, 1.237)	0.544 (0.286, 1.035)	0.970 (0.735, 1.282)	**0.840 (0.719, 0.980)**
1.194 (0.938, 1.519)	ASE	0.890 (0.561, 1.411)	1.180 (0.818, 1.704)	0.991 (0.684, 1.436)	0.376 (0.017, 8.486)	0.405 (0.040, 4.056)	1.230 (0.588, 2.572)	1.127 (0.834, 1.522)	0.897 (0.600, 1.339)	0.850 (0.514, 1.406)	0.969 (0.720, 1.306)	**1.542 (1.183, 2.010)**		1.311 (0.879, 1.956)	1.315 (0.926, 1.868)	1.276 (0.905, 1.800)	1.307 (0.597, 2.861)	0.747 (0.522, 1.070)	0.570 (0.302, 1.074)	1.176 (0.878, 1.575)	0.646 (0.330, 1.267)	1.154 (0.819, 1.626)	0.998 (0.777, 1.282)
		BRE	1.326 (0.828, 2.125)	1.114 (0.693, 1.789)	0.422 (0.018, 9.675)	0.455 (0.044, 4.649)	1.382 (0.624, 3.059)	1.266 (0.827, 1.938)	1.007 (0.612, 1.659)	0.955 (0.533, 1.711)	1.089 (0.715, 1.660)	**1.733 (1.141, 2.632)**		1.473 (0.896, 2.422)	1.478 (0.934, 2.339)	1.434 (0.908, 2.267)	1.469 (0.636, 3.392)	0.840 (0.528, 1.337)	0.640 (0.318, 1.290)	1.321 (0.867, 2.012)	0.726 (0.348, 1.514)	1.296 (0.824, 2.040)	1.121 (0.762, 1.651)
0.804 (0.577, 1.119)	**0.673 (0.470, 0.964)**		CARB	0.840 (0.572, 1.233)	0.318 (0.014, 7.205)	0.343 (0.034, 3.445)	1.042 (0.495, 2.194)	0.955 (0.692, 1.317)	0.760 (0.505, 1.143)	0.720 (0.431, 1.203)	0.821 (0.610, 1.106)	1.307 (0.958, 1.782)		1.111 (0.736, 1.677)	1.114 (0.774, 1.604)	1.081 (0.752, 1.555)	1.108 (0.503, 2.440)	**0.633 (0.438, 0.916)**	**0.483 (0.254, 0.918)**	0.996 (0.730, 1.360)	0.548 (0.277, 1.081)	0.977 (0.683, 1.399)	0.846 (0.646, 1.107)
0.981 (0.761, 1.265)	0.822 (0.615, 1.099)		1.221 (0.846, 1.762)	CARI	0.379 (0.017, 8.585)	0.408 (0.041, 4.108)	1.241 (0.589, 2.617)	1.137 (0.820, 1.576)	0.905 (0.596, 1.373)	0.858 (0.513, 1.435)	0.978 (0.710, 1.348)	**1.556 (1.134, 2.135)**		1.323 (0.873, 2.004)	1.327 (0.918, 1.919)	1.288 (0.892, 1.859)	1.319 (0.598, 2.909)	0.754 (0.518, 1.099)	0.575 (0.301, 1.096)	1.186 (0.861, 1.634)	0.652 (0.330, 1.290)	1.164 (0.811, 1.672)	1.007 (0.766, 1.324)
					CHL	1.077 (0.023, 51.229)	3.275 (0.136, 79.146)	3.000 (0.135, 66.806)	2.387 (0.105, 54.277)	2.264 (0.098, 52.243)	2.581 (0.115, 58.009)	4.106 (0.183, 92.121)		3.491 (0.154, 79.353)	3.502 (0.155, 79.075)	3.399 (0.151, 76.715)	3.481 (0.142, 85.030)	1.990 (0.088, 45.024)	1.517 (0.064, 35.834)	3.131 (0.139, 70.344)	1.721 (0.072, 40.997)	3.072 (0.136, 69.214)	2.658 (0.119, 59.490)
1.413 (0.909, 2.197)	1.184 (0.749, 1.871)		**1.758 (1.050, 2.944)**	1.440 (0.899, 2.306)		END	3.041 (0.277, 33.368)	2.785 (0.280, 27.752)	2.216 (0.219, 22.405)	2.102 (0.204, 21.685)	2.396 (0.241, 23.834)	3.812 (0.384, 37.845)		3.241 (0.320, 32.779)	3.251 (0.324, 32.619)	3.156 (0.315, 31.633)	3.232 (0.290, 35.978)	1.848 (0.184, 18.556)	1.408 (0.132, 15.001)	2.906 (0.295, 28.608)	1.598 (0.148, 17.202)	2.852 (0.285, 28.583)	2.467 (0.249, 24.432)
**1.510 (1.099, 2.073)**	1.264 (0.894, 1.789)		**1.878 (1.243, 2.838)**	**1.538 (1.080, 2.191)**		1.068 (0.643, 1.775)	ESL	0.916 (0.447, 1.876)	0.729 (0.340, 1.562)	0.691 (0.305, 1.569)	0.788 (0.386, 1.609)	1.254 (0.615, 2.555)		1.066 (0.498, 2.281)	1.069 (0.512, 2.234)	1.038 (0.497, 2.166)	1.063 (0.385, 2.936)	0.608 (0.290, 1.274)	0.463 (0.187, 1.147)	0.956 (0.468, 1.951)	0.526 (0.207, 1.337)	0.938 (0.451, 1.953)	0.811 (0.405, 1.624)
0.931 (0.787, 1.102)	**0.780 (0.619, 0.983)**		1.158 (0.834, 1.609)	0.949 (0.738, 1.220)		0.659 (0.426, 1.020)	**0.617 (0.450, 0.845)**	HAL	0.796 (0.555, 1.140)	0.755 (0.471, 1.209)	0.860 (0.679, 1.090)	**1.369 (1.106, 1.694)**		1.164 (0.814, 1.664)	1.167 (0.871, 1.564)	1.133 (0.849, 1.513)	1.160 (0.541, 2.489)	**0.663 (0.485, 0.907)**	**0.506 (0.275, 0.929)**	1.044 (0.824, 1.322)	0.574 (0.300, 1.099)	1.024 (0.782, 1.341)	0.886 (0.741, 1.059)
1.214 (0.919, 1.603)	1.017 (0.745, 1.387)		**1.510 (1.028, 2.218)**	1.237 (0.896, 1.709)		0.859 (0.531, 1.389)	0.804 (0.553, 1.170)	1.304 (0.990, 1.717)	LAM	0.948 (0.554, 1.624)	1.081 (0.785, 1.489)	**1.720 (1.215, 2.436)**		1.462 (0.939, 2.278)	1.467 (0.986, 2.183)	1.424 (0.957, 2.119)	1.458 (0.651, 3.265)	0.834 (0.559, 1.243)	0.635 (0.327, 1.234)	1.311 (0.923, 1.862)	0.721 (0.358, 1.452)	1.287 (0.868, 1.908)	1.113 (0.812, 1.526)
**1.501 (1.014, 2.222)**	1.257 (0.829, 1.907)		**1.867 (1.163, 2.997)**	**1.529 (1.002, 2.334)**		1.062 (0.608, 1.855)	0.994 (0.626, 1.580)	**1.612 (1.090, 2.383)**	1.236 (0.796, 1.920)	LIC	1.140 (0.715, 1.819)	**1.814 (1.141, 2.884)**		1.542 (0.902, 2.636)	1.547 (0.937, 2.554)	1.501 (0.911, 2.475)	1.538 (0.650, 3.636)	0.879 (0.530, 1.459)	0.670 (0.323, 1.389)	1.383 (0.867, 2.204)	0.760 (0.355, 1.629)	1.357 (0.826, 2.228)	1.174 (0.759, 1.815)
1.053 (0.881, 1.260)	0.882 (0.702, 1.109)		1.311 (0.951, 1.806)	1.074 (0.838, 1.375)		0.745 (0.485, 1.145)	**0.698 (0.511, 0.953)**	1.131 (0.948, 1.350)	0.868 (0.680, 1.107)	0.702 (0.476, 1.035)	LIT	**1.591 (1.278, 1.981)**		1.352 (0.951, 1.924)	**1.357 (1.016, 1.812)**	1.317 (0.983, 1.765)	1.349 (0.630, 2.886)	0.771 (0.581, 1.023)	0.588 (0.320, 1.078)	1.213 (0.975, 1.508)	0.667 (0.349, 1.273)	1.190 (0.892, 1.588)	1.030 (0.871, 1.217)
0.963 (0.805, 1.151)	**0.806 (0.662, 0.983)**		1.198 (0.867, 1.655)	0.981 (0.768, 1.253)		0.681 (0.447, 1.040)	**0.638 (0.468, 0.869)**	1.034 (0.885, 1.209)	0.793 (0.609, 1.032)	**0.642 (0.436, 0.944)**	0.914 (0.782, 1.068)	OLA		0.850 (0.599, 1.206)	0.853 (0.638, 1.141)	0.828 (0.633, 1.083)	0.848 (0.397, 1.810)	**0.485 (0.359, 0.655)**	**0.369 (0.202, 0.674)**	**0.762 (0.626, 0.928)**	**0.419 (0.220, 0.798)**	**0.748 (0.565, 0.990)**	**0.647 (0.552, 0.758)**
1.208 (0.807, 1.809)	1.012 (0.664, 1.543)		1.503 (0.926, 2.438)	1.231 (0.796, 1.904)		0.855 (0.515, 1.419)	0.800 (0.498, 1.287)	1.298 (0.871, 1.934)	0.995 (0.637, 1.555)	0.805 (0.474, 1.366)	1.147 (0.776, 1.694)	1.255 (0.855, 1.840)	OXC										
1.097 (0.837, 1.440)	0.919 (0.677, 1.248)		1.365 (0.934, 1.996)	1.118 (0.816, 1.533)		0.777 (0.481, 1.255)	0.727 (0.503, 1.050)	1.179 (0.903, 1.539)	0.904 (0.647, 1.263)	0.731 (0.473, 1.129)	1.042 (0.805, 1.349)	1.140 (0.877, 1.481)	0.908 (0.582, 1.418)	PAL	1.003 (0.698, 1.441)	0.974 (0.655, 1.447)	0.997 (0.446, 2.230)	**0.570 (0.380, 0.855)**	**0.434 (0.224, 0.843)**	0.897 (0.630, 1.277)	**0.493 (0.245, 0.991)**	0.880 (0.595, 1.301)	0.761 (0.557, 1.040)
0.985 (0.798, 1.216)	0.825 (0.640, 1.064)		1.226 (0.872, 1.722)	1.004 (0.768, 1.312)		0.697 (0.446, 1.090)	**0.653 (0.470, 0.906)**	1.058 (0.867, 1.292)	0.812 (0.611, 1.078)	**0.656 (0.439, 0.981)**	0.935 (0.784, 1.116)	1.023 (0.841, 1.245)	0.816 (0.541, 1.229)	0.898 (0.702, 1.148)	QUE	0.971 (0.686, 1.372)	0.994 (0.455, 2.173)	**0.568 (0.398, 0.811)**	**0.433 (0.230, 0.817)**	0.894 (0.665, 1.202)	**0.491 (0.251, 0.962)**	0.877 (0.624, 1.232)	**0.759 (0.592, 0.972)**
0.906 (0.725, 1.131)	**0.758 (0.586, 0.981)**		1.127 (0.795, 1.597)	0.923 (0.699, 1.218)		0.641 (0.408, 1.007)	**0.600 (0.429, 0.839)**	0.973 (0.794, 1.192)	0.746 (0.553, 1.007)	**0.603 (0.401, 0.907)**	0.860 (0.694, 1.065)	0.940 (0.777, 1.138)	0.750 (0.495, 1.135)	0.825 (0.615, 1.107)	0.919 (0.724, 1.167)	RIS	1.024 (0.469, 2.237)	**0.586 (0.411, 0.835)**	**0.446 (0.246, 0.810)**	0.921 (0.689, 1.230)	**0.506 (0.259, 0.990)**	0.904 (0.645, 1.267)	**0.782 (0.612, 0.999)**
**0.205 (0.051, 0.821)**	**0.172 (0.043, 0.693)**		0.255 (0.062, 1.047)	**0.209 (0.052, 0.844)**		**0.145 (0.034, 0.614)**	**0.136 (0.033, 0.556)**	**0.220 (0.055, 0.881)**	**0.169 (0.042, 0.686)**	**0.137 (0.033, 0.570)**	**0.195 (0.049, 0.778)**	**0.213 (0.053, 0.851)**	**0.170 (0.040, 0.710)**	**0.187 (0.046, 0.758)**	**0.208 (0.052, 0.835)**	**0.226 (0.056, 0.911)**	TAM	0.572 (0.261, 1.254)	0.436 (0.169, 1.121)	0.899 (0.420, 1.924)	0.494 (0.187, 1.304)	0.882 (0.405, 1.923)	0.763 (0.363, 1.604)
**1.673 (1.296, 2.161)**	**1.401 (1.046, 1.877)**		**2.082 (1.440, 3.008)**	**1.705 (1.260, 2.308)**		1.184 (0.740, 1.893)	1.108 (0.775, 1.585)	**1.797 (1.395, 2.315)**	**1.378 (1.004, 1.892)**	1.115 (0.728, 1.706)	**1.588 (1.267, 1.991)**	**1.738 (1.362, 2.217)**	1.385 (0.897, 2.138)	**1.525 (1.111, 2.092)**	**1.698 (1.305, 2.210)**	**1.848 (1.397, 2.444)**	**8.165 (2.018, 33.033)**	TOP	0.762 (0.402, 1.443)	**1.573 (1.161, 2.131)**	0.865 (0.440, 1.700)	**1.543 (1.088, 2.190)**	**1.335 (1.032, 1.728)**
																			VALN	**2.064 (1.126, 3.784)**	1.135 (0.482, 2.669)	**2.025 (1.079, 3.802)**	1.752 (0.977, 3.142)
1.074 (0.858, 1.345)	0.899 (0.696, 1.162)		1.336 (0.943, 1.894)	1.094 (0.829, 1.445)		0.760 (0.520, 1.111)	**0.711 (0.508, 0.996)**	1.153 (0.929, 1.432)	0.885 (0.659, 1.188)	0.716 (0.476, 1.077)	1.019 (0.835, 1.244)	1.115 (0.927, 1.342)	0.889 (0.636, 1.243)	0.979 (0.730, 1.312)	1.090 (0.861, 1.380)	1.186 (0.928, 1.515)	**5.240 (1.302, 21.092)**	**0.642 (0.487, 0.846)**		VALP	0.550 (0.288, 1.049)	0.981 (0.736, 1.308)	0.849 (0.719, 1.002)
1.155 (0.216, 6.168)	0.968 (0.180, 5.196)		1.437 (0.264, 7.833)	1.177 (0.219, 6.331)		0.818 (0.146, 4.571)	0.765 (0.141, 4.160)	1.241 (0.233, 6.621)	0.952 (0.176, 5.141)	0.770 (0.139, 4.250)	1.097 (0.206, 5.849)	1.200 (0.225, 6.396)	0.956 (0.173, 5.297)	1.053 (0.195, 5.678)	1.173 (0.219, 6.273)	1.276 (0.238, 6.836)	5.637 (0.646, 49.176)	0.690 (0.128, 3.716)		1.076 (0.201, 5.765)	VER	1.785 (0.915, 3.482)	1.544 (0.827, 2.884)
1.132 (0.861, 1.487)	0.948 (0.695, 1.293)		1.408 (0.958, 2.069)	1.153 (0.836, 1.590)		0.801 (0.494, 1.299)	0.750 (0.516, 1.089)	1.215 (0.947, 1.560)	0.932 (0.662, 1.313)	0.754 (0.486, 1.169)	1.074 (0.819, 1.409)	1.175 (0.902, 1.531)	0.937 (0.598, 1.468)	1.031 (0.737, 1.442)	1.149 (0.861, 1.533)	1.250 (0.930, 1.679)	**5.522 (1.359, 22.430)**	**0.676 (0.489, 0.935)**		1.054 (0.781, 1.421)	0.980 (0.181, 5.289)	ZIP	0.865 (0.683, 1.096)
**1.529 (1.327, 1.762)**	**1.281 (1.049, 1.563)**		**1.902 (1.409, 2.567)**	**1.558 (1.262, 1.924)**		1.082 (0.710, 1.648)	1.013 (0.763, 1.345)	**1.642 (1.432, 1.883)**	1.259 (0.986, 1.608)	1.019 (0.706, 1.469)	**1.451 (1.275, 1.652)**	**1.588 (1.403, 1.797)**	1.266 (0.865, 1.853)	**1.393 (1.102, 1.761)**	**1.552 (1.316, 1.830)**	**1.689 (1.411, 2.021)**	**7.461 (1.876, 29.678)**	0.914 (0.736, 1.135)		**1.424 (1.188, 1.707)**	1.324 (0.249, 7.023)	**1.351 (1.061, 1.721)**	PLA

Drugs are reported alphabetically. Data are presented as risk ratios (95% confidence interval) in the column-defining treatment compared with the row-defining treatment. For efficacy, risk ratios >1 favor the column-defining treatment. For acceptability, risk ratios <1 favor the row-defining treatment.

Significant results are in bold.

*ARI* aripiprazole, *ASE* asenapine, *BRE* brexpiprazole, *CARB* carbamazepine, *CARI* cariprazine, *CHL* chlorpromazine, *END* endoxifen, *ESL* eslicarbazepine, *HAL* haloperidol, *LAM* lamotrigine, *LIC* licarbazepine, *LIT* lithium, *OLA* olanzapine, *OXC* oxcarbazepine, *PAL* paliperidone, *PLA* placebo, *QUE* quetiapine, *RIS* risperidone, *TAM* tamoxifen, *TOP* topiramate, *VALN* valnoctamide, *VALP* valproate, *VER* verapamil, *ZIP* ziprasidone.

Meta-regression analyses showed that older studies had a higher RR for the response to treatment (Supplementary Appendix S1). Studies including more male individuals had a higher RR for the outcome (Supplementary Appendix S1). The between-study variance of these meta-regression analyses were decreased compared with the primary analysis (Supplementary Appendix S1). Five sensitivity analyses (focusing on studies without a placebo arm, nonindustry-sponsored studies, studies with high-quality design, studies not including individuals with rapid-cycling, and studies not including individuals with mixed state/episode) reduced the between-study variance compared with the primary analysis (Supplementary Appendix S1). However, when compared with the placebo, the effect size for each drug on the outcome of the primary analysis was similar to that of the adjusted analyses (Supplementary Appendix S1).

The data of response to treatment at the time of two or more observational points were available for seven drugs (Supplementary Appendix S1). Compared with the placebo, the effect size of most drugs other than lamotrigine seemed to increase over time. However, the number of studies included in the meta-analysis at all observational points other than at 3 weeks was small.

### All-cause discontinuation

**Fig. 2:**
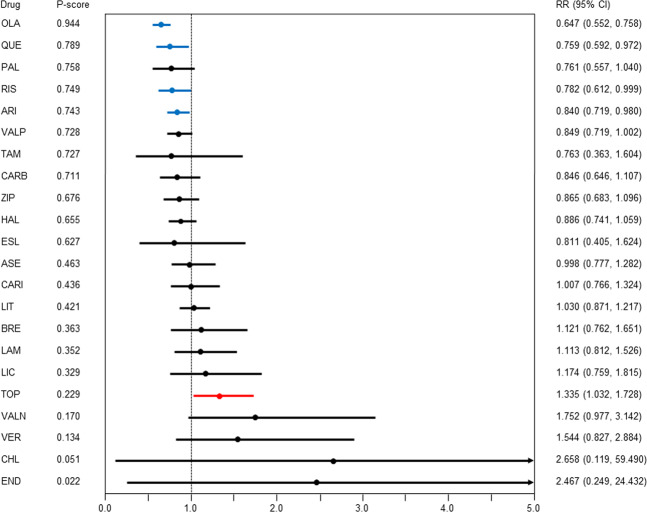
All-cause discontinuation. Drugs were compared with the placebo. Colors indicate the presence or absence of a significant difference: blue, the drug was superior to the placebo; black, the drug was similar to the placebo; red, the drug was inferior to the placebo. 95% CI 95% confidence interval, ARI aripiprazole, ASE asenapine, BRE brexpiprazole, CARB carbamazepine, CARI cariprazine, CHL chlorpromazine, END endoxifen, ESL eslicarbazepine, HAL haloperidol, LAM lamotrigine, LIC licarbazepine, LIT lithium, OLA olanzapine, PAL paliperidone, QUE quetiapine, RIS risperidone, RR risk ratio, TAM tamoxifen, TOP topiramate, VALN valnoctamide, VALP valproate, VER verapamil, ZIP ziprasidone.

Compared with the placebo, aripiprazole, olanzapine, quetiapine, and risperidone had lower all-cause discontinuation (RR [95% CI] ranged from 0.647 [0.552–0.758] for olanzapine to 0.840 [0.719–0.980] for aripiprazole; *N* = 70, *n* = 16324; Fig. 2, Table 1), whereas topiramate had higher all-cause discontinuation (1.335 [1.032–1.728]). Aripiprazole, carbamazepine, haloperidol, valproate, and ziprasidone outperformed topiramate and valnoctamide; olanzapine outperformed aripiprazole, asenapine, brexpiprazole, cariprazine, haloperidol, lamotrigine, licarbazepine, lithium, topiramate, valnoctamide, valproate, verapamil, and ziprasidone; paliperidone and risperidone outperformed topiramate, valnoctamide, and verapamil; and quetiapine outperformed lithium, topiramate, valnoctamide, and verapamil. Although global heterogeneity was low, we detected significant global inconsistency. There was statistical agreement between direct and indirect estimates, with the exception of the following three comparisons: aripiprazole vs. haloperidol (aripiprazole outperformed haloperidol in the direct comparison but not in the indirect comparison), aripiprazole vs. placebo (aripiprazole outperformed the placebo in the indirect comparison but not in the direct comparison), and haloperidol vs. quetiapine (quetiapine outperformed haloperidol in the indirect comparison but in the direct comparison).

Meta-regression analyses showed that studies involving more individuals with psychotic features had a lower RR for all-cause discontinuation (Supplementary Appendix S2). The between-study variance of the meta-regression analysis decreased compared with the unadjusted analysis (Supplementary Appendix S2). Compared with the placebo, cariprazine, haloperidol, lithium, paliperidone, tamoxifen, valproate, and ziprasidone had lower all-cause discontinuation (Supplementary Appendix S2). For other drugs, the adjusted analyses had similar results with the unadjusted analysis (Supplementary Appendix S2).

Three sensitivity analyses (focusing on studies without a placebo arm, nonindustry-sponsored studies, and studies with high-quality design) reduced the between-study variance compared with the primary analysis (Supplementary Appendix S2). These sensitivity analyses showed that chlorpromazine and endoxifen had higher, and valproate had lower all-cause discontinuation compared with the placebo (Supplementary Appendix S2). For the other drugs, the adjusted analyses had similar results with the unadjusted analysis (Supplementary Appendix S2).

### Mania rating scale scores

**Fig. 3:**
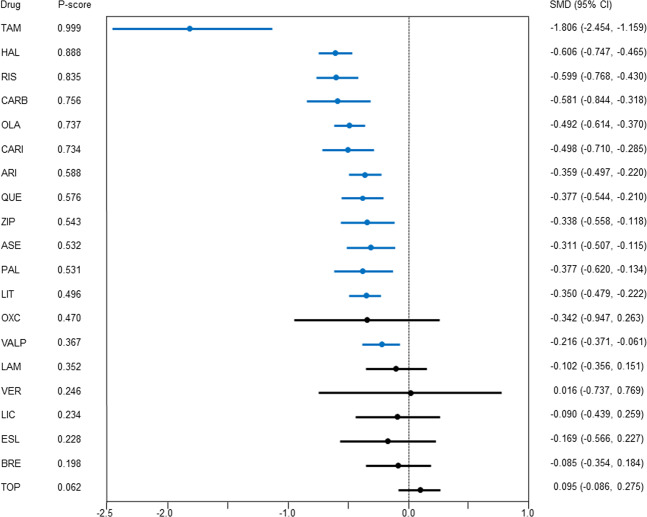
Mania rating scale scores. Drugs were compared with the placebo. Colors indicate the presence or absence of a significant difference: blue, the drug was superior to the placebo; black, the drug was similar to the placebo. 95% CI 95% confidence interval, ARI aripiprazole, ASE asenapine, BRE brexpiprazole, CARB carbamazepine, CARI cariprazine, ESL eslicarbazepine, HAL haloperidol, LAM lamotrigine, LIC licarbazepine, LIT lithium, OLA olanzapine, OXC oxcarbazepine, PAL paliperidone, QUE quetiapine, RIS risperidone, SMD standardized mean difference, TAM tamoxifen, TOP topiramate, VALP valproate, VER verapamil, ZIP ziprasidone.

Compared with the placebo, aripiprazole, asenapine, carbamazepine, cariprazine, haloperidol, lithium, olanzapine, paliperidone, quetiapine, risperidone, tamoxifen, valproate, and ziprasidone showed better improvement of the mania rating scale scores (*N* = 61, *n* = 15466; Fig. 3); the SMD (95% CI) ranged from −1.806 (−2.454, −1.159) for tamoxifen to −0.216 (−0.371, −0.061) for valproate.

### Discontinuation due to inefficacy

**Fig. 4:**
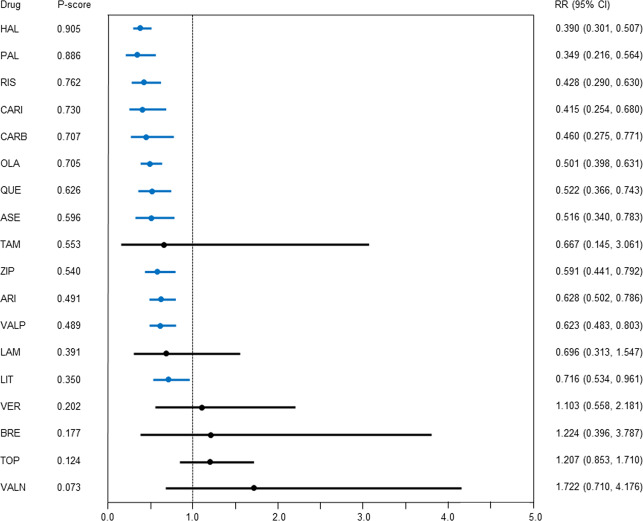
Discontinuation due to inefficacy. Drugs were compared with the placebo. Colors indicate the presence or absence of a significant difference: blue, the drug was superior to the placebo; black, the drug was similar to the placebo. 95% CI 95% confidence interval, ARI aripiprazole, ASE asenapine, BRE brexpiprazole, CARB carbamazepine, CARI cariprazine, HAL haloperidol, LAM lamotrigine, LIT lithium, OLA olanzapine, PAL paliperidone, QUE quetiapine, RIS risperidone, RR risk ratio, TAM tamoxifen, TOP topiramate, VALN valnoctamide, VALP valproate, VER verapamil, ZIP ziprasidone.

Compared with the placebo, aripiprazole, asenapine, carbamazepine, cariprazine, haloperidol, lithium, olanzapine, paliperidone, quetiapine, risperidone, valproate, and ziprasidone had lower discontinuation due to inefficacy, with the RR (95% CI) ranging from 0.349 (0.216–0.564) for paliperidone to 0.716 (0.534–0.961) for lithium (*N* = 50, *n* = 14284; Fig. 4).

### Clinical remission and psychotic symptoms

Aripiprazole, asenapine, cariprazine, haloperidol, lithium, olanzapine, paliperidone, quetiapine, risperidone, and tamoxifen outperformed the placebo for clinical remission (*N* = 31, *n* = 9320); the RR (95% CI) ranged from 8.441 (1.116, 63.841) for tamoxifen to 1.259 (1.007, 1.576) for lithium.

Compared with the placebo, aripiprazole, cariprazine, haloperidol, olanzapine, quetiapine, risperidone, tamoxifen, and ziprasidone showed better improvement of psychotic symptoms (*N* = 30, *n* = 7029); the SMD (95% CI) ranged from −1.640 (−2.335, −0.945) for tamoxifen to −0.266 (−0.490, −0.041) for aripiprazole.

### Tolerability and safety outcomes

Compared with the placebo, asenapine (RR [95% CI] = 1.896 [1.117–3.218]), haloperidol (1.867 [1.255–2.776]), and lithium (1.791 [1.093–2.936]) had higher discontinuation due to adverse events (*N* = 52, *n* = 14629), while olanzapine had lower discontinuation due to withdrawal consent (0.643 [0.466–0.889], *N* = 42, *n* = 11968).

No drug was associated with the incidence of depression compared with the placebo (*N* = 19, *n* = 5740).

In addition, compared with the placebo, olanzapine (RR [95% CI] = 0.881 [0.800–0.971]) and quetiapine (0.767 [0.665–0.885]) were associated with a lower frequency of anxiolytic use (*N* = 28, *n* = 8082); aripiprazole, cariprazine, haloperidol, paliperidone, risperidone, and ziprasidone were associated with a higher frequency of anticholinergic use (RR [95% CI] ranged from 2.374 [1.384–4.072] for paliperidone to 6.299 [4.159–9.541] for haloperidol, *N* = 20, *n* = 6256); aripiprazole, brexpiprazole, cariprazine, haloperidol, paliperidone, risperidone, and ziprasidone were associated with a higher incidence of akathisia (RR [95% CI] ranged from 2.586 [1.188–5.631] for paliperidone to 5.579 [3.959–7.862] for haloperidol, *N* = 25, *n* = 8711); aripiprazole, asenapine, cariprazine, haloperidol, lithium, olanzapine, risperidone, and ziprasidone were associated with a higher incidence of extrapyramidal symptoms (RR [95% CI] ranged from 1.817 [1.012, 3.261] for olanzapine to 5.337 [3.997–7.126] for haloperidol, *N* = 31, *n* = 9265); aripiprazole, asenapine, carbamazepine, cariprazine, haloperidol, lithium, olanzapine, paliperidone, quetiapine, risperidone, valproate, and ziprasidone were associated with a higher incidence of somnolence (RR [95% CI] ranged from 1.609 [1.055, 2.453] for lithium to 5.158 [1.515, 17.561] for cariprazine, *N* = 37, *n* = 10395); asenapine, carbamazepine, haloperidol, olanzapine, quetiapine, valproate, and ziprasidone were associated with a higher incidence of dizziness (RR [95% CI] ranged from 2.037 [1.334, 3.110] for valproate to 3.552 [2.369, 5.323] for carbamazepine, *N* = 33, *n* = 8775); carbamazepine (RR [95% CI] = 4.079 [1.109, 15.010]), olanzapine (3.758 [2.147–6.577]), and quetiapine (3.630 [2.243–5.876]) were associated with a higher incidence of dry mouth (*N* = 16, *n* = 3967); aripiprazole, cariprazine, olanzapine, and quetiapine were associated with a higher incidence of constipation (RR [95% CI] ranged from 1.735 [1.152–2.613] for aripiprazole to 2.866 [1.537–5.345] for quetiapine, *N* = 27, *n* = 6670); and asenapine, olanzapine, paliperidone, quetiapine, valproate, and ziprasidone were associated with a higher incidence of weight gain (RR [95% CI] ranged from 2.928 [1.259–6.807] for ziprasidone to 8.180 [4.419–15.142] for olanzapine, *N* = 31, *n* = 8704).

Compared with the placebo, quetiapine was associated with a lower incidence of nausea (RR [95% CI] = 0.313 [0.130–0.758]), whereas aripiprazole, carbamazepine, cariprazine, lithium, risperidone, and valproate were associated with a higher incidence of nausea (*N* = 29, *n* = 7915); the RR (95% CI) ranged from 1.558 (1.164–2.085) for aripiprazole to 4.664 (1.320–16.479) for risperidone.

There were no significant differences in the incidence of headache (*N* = 37, *n* = 10330) and diarrhea (*N* = 20, *n* = 4981) between each drug and the placebo.

### Heterogeneity, inconsistency, and network meta-analysis results graded using the CINeMA application

Global heterogeneity was low or low–moderate for most outcomes, moderate–high for discontinuation due to adverse events and diarrhea, and high for depression (Supplementary Appendixes S1–S21). There was considerable local heterogeneity for most of the outcomes in specific comparisons. We detected significant global inconsistency for all-cause discontinuation (as mentioned before), psychotic symptoms, discontinuation due to adverse events, and depression. The SIDE test for local inconsistency showed some hotspots: haloperidol vs. lithium, haloperidol vs. placebo, lithium vs. risperidone, risperidone vs. valnoctamide, and valnoctamide vs. placebo for psychotic symptoms; valproate vs. placebo for discontinuation due to adverse events; and olanzapine vs. placebo for depression. The proportion of comparisons with evidence of inconsistency was few for all outcomes (0.00%–20.83%). However, the within-study bias of most of the comparisons was evaluated “Some concerns.” Moreover, because funnel plots with less than 10 studies were not meaningful [10], all comparisons for publication bias were evaluated as “Suspected.” Furthermore, if the comparison had only indirect evidence, the comparison was downgraded one level. Consequently, the confidence in the evidence was generally evaluated as low or very low.

## Discussion

This systematic review and network meta-analysis was conducted to compare the efficacy, acceptability, tolerability, and safety of pharmacological interventions for adults with acute bipolar mania. We included only double-blind RCTs and extended a recent study by including brexpiprazole, endoxifen, and eslicarbazepine, and by investigating many more adverse events [7]. Supplementary Fig. S3 shows two-dimensional graphs of the primary efficacy and acceptability outcomes. The agents that outperformed the placebo in the primary and secondary outcomes were aripiprazole, olanzapine, quetiapine, and risperidone. These SGAs also outperformed the placebo in terms of clinical remission and improvement of psychotic symptoms. Therefore, they appear to have a better balance of efficacy and acceptability in the treatment of acute mania than that of other drugs. As treatments prescribed for an acute mood episode are usually continued into maintenance treatment, clinicians and individuals with mania should consider the efficacy and safety in the maintenance phase when selecting a treatment for the acute phase [4]. A recent network meta-analysis of BD in the maintenance phase showed that these SGAs prevent the recurrence/relapse of any mood episode [33]. However, because these agents have several adverse events, clinicians must monitor individuals with BD for health conditions.

Tamoxifen is the best treatment for all efficacy outcomes except for discontinuation due to inefficacy. However, our results were based on two small studies (<40 individuals in each treatment arm) [34, 35]. Tamoxifen, which is approved to treat breast cancer, has serious and specific side effects, including uterine malignancy, thromboembolic events, and embryo-fetal toxicity [11]. Therefore, large-scale trials are needed to examine the efficacy and safety of tamoxifen in cases with ineffective existing treatments.

Haloperidol and carbamazepine ranked high in most of the efficacy outcomes. However, we found that haloperidol was not well-tolerated and had a high risk of akathisia and extrapyramidal symptoms. Carbamazepine carries a risk of cutaneous adverse reactions, such as Stevens–Johnson syndrome and toxic epidermal necrolysis [11], for which the presence of a specific allele are strong risk factor in some races/ethnicities [36].

Although our network meta-analysis confirmed that lithium and valproate were effective for mania symptoms, they ranked lower in efficacy outcomes compared with most antipsychotics. While these drugs were gradually increased according to the individual’s condition, antipsychotics were used at relatively high doses from the start of treatment (Supplementary Table S2). Moreover, we found that lithium was not well-tolerated. These factors might affect the efficacy outcomes of lithium and valproate. Meanwhile, a recent network meta-analysis demonstrated that lithium and valproate prevented the recurrence/relapse of any mood episode [33]. A Finnish nationwide cohort study also showed that these drugs prevented hospitalization [37]. Moreover, a meta-review reported that lithium had anti-suicidal effects for individuals with mood disorders [38]. Taken together, these findings suggest that lithium and valproate are still key drugs for BD treatment, although clinicians must pay close attention to the side effects of these drugs [33, 39, 40].

Although most antipsychotics improved psychotic symptoms, carbamazepine, lithium, and valproate did not. Thus, these antipsychotics should be reserved for individuals with psychotic features. Although the network showed significant global inconsistency, when performing a sensitivity analysis using only the Positive and Negative Syndrome Scale [41] data, significant global inconsistency disappeared (Supplementary Appendix S6). Furthermore, compared with the placebo, the effect size for each drug on the outcome of the primary analysis was similar to that of the adjusted analysis.

Our study had some limitations. First, 70.8% of the studies included in our meta-analysis were evaluated as moderate overall ROB. Although global heterogeneity was low for the primary efficacy and acceptability outcomes, the sensitivity analyses focusing on studies with high-quality design reduced the between-study variance for both outcomes compared with the primary analysis. Second, mixed episodes are not clustered with hypomania and mania in the Diagnostic and Statistical Manual of Mental Disorders, Fifth Edition (DSM-5) [42]. In the DSM-5, the term “mixed episode” has been changed to “mixed features.” Third, the range of the study duration included in our meta-analysis was 1–12 weeks. Thus, the long-term efficacy and safety of drugs still need to be verified. Fourth, we did not examine whether the magnitude of the placebo-response influenced our results. A recent meta-regression analysis including RCTs of antipsychotics and mood stabilizers compared with placebo demonstrated that the effect size for efficacy was influenced by the magnitude of both the drug- and placebo-response [43]. Further studies will need to explore whether there is an interaction between the drug-response and the placebo-response regarding the effect size. It will also be important to identify modifiers of the drug-response and explore how they interplay with modifiers of the placebo-response in the formation of effect sizes. Finally, we did not cover important clinical issues that might inform treatment decision-making in routine clinical practice (e.g., combination with nonpharmacological treatments and cost-effectiveness).

In conclusions, the aforementioned antipsychotics, carbamazepine, lithium, tamoxifen, and valproate were found to have efficacy for acute bipolar mania. However, only aripiprazole, olanzapine, quetiapine, and risperidone had better acceptability than the placebo. Because these agents carry the risk of several adverse events, clinicians must monitor individuals with BD for health conditions.

## Supplementary information

Supplementary materials
