# Supplementary material for: Pharmacological treatment for bipolar mania: a systematic review and network meta-analysis of double-blind randomized controlled trials
Source: Mol Psychiatry. 2021 Oct 12;27(2):1136–44. doi: 10.1038/s41380-021-01334-4 (PMC9054678; doi:10.1038/s41380-021-01334-4)
Supplement: Supplementary file 1 — Supplementary materials [file 41380_2021_1334_MOESM1_ESM.pdf]

Supplementary Figure 1. Flow diagram of literature search.

The inclusion criteria for studies were as follows: (1) published and unpublished randomized controlled trials (RCTs) of oral monotherapy lasting for ≥10 days, (2) studies of adults with acute bipolar mania, and (3) double- and single-blind studies. The exclusion criteria were as follows: (1) open-label studies, (2) studies in which selection bias was evaluated as high risk according to the Cochrane risk of bias criteria (Higgins 2019), (3) studies including children/adolescents with mania, (4) studies included individuals with a dual diagnosis of bipolar disorder and other disorders, (5) studies that allowed antipsychotics as a rescue medication during a trial, and (6) studies that terminated early without efficacy analysis. We searched PubMed, Cochrane Library, and Embase databases for studies published before March 14, 2021. The search terms for PubMed and Cochrane Library included (bipolar disorder OR mania OR manic) AND (random*). No language restriction was applied to the literature search. The search terms for Embase included ('randomized controlled trial'/exp OR 'randomized controlled trial') AND ('bipolar mania'/exp OR 'bipolar mania'). In addition, reference lists of the included articles were manually searched for additional relevant published and unpublished research, including conference abstracts. We also searched clinical trial registries (ClinicalTrials.gov [http://clinicaltrials.gov/] and the World Health Organization International Clinical Trials Registry Platform [http://www.who.int/ictrp/search/en/]) to ensure the RCTs were comprehensive and to minimize the effect of publication bias. Any discrepancies in the selected articles were resolved by consensus in a meeting among the authors. If multiple papers or academic conference abstracts were reported despite the same research, the literature was screened by confirming the clinical trial registration number and/or reference to past review articles.

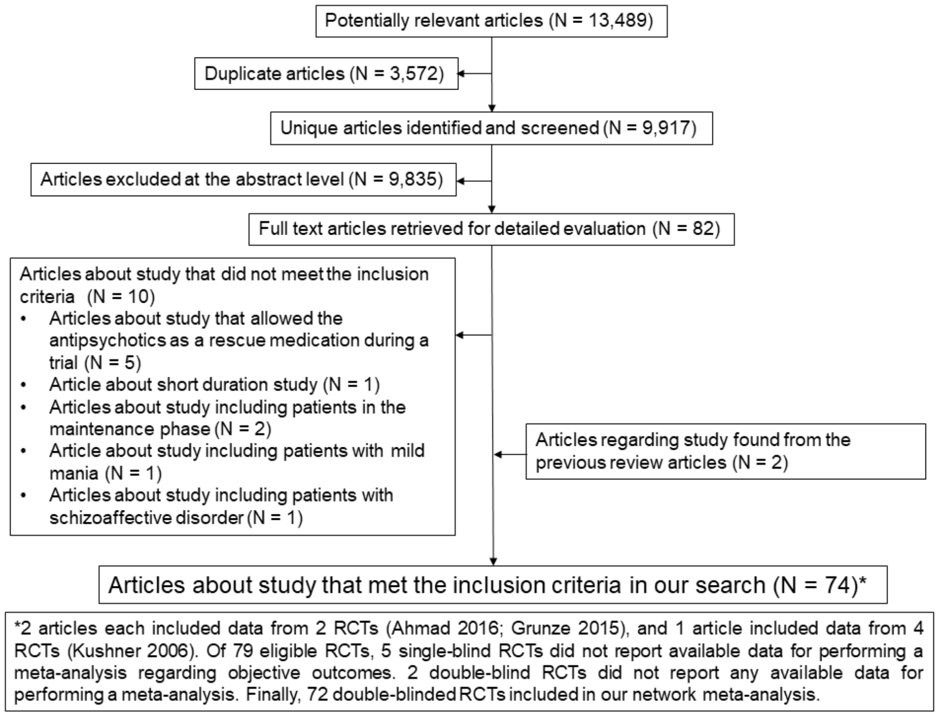

Of the 13489 articles initially identified, 3572 were duplicates, 9835 were excluded after reviewing the titles and abstracts, and 10 were excluded after reviewing the full texts. In total, 72 articles on eligible studies were selected, and 2 articles were detected from previous review articles. Two articles each included data from two RCTs (Ahmad 2016; Grunze 2015), and one article included data from four RCTs (Kushner 2006). Of 79 eligible RCTs, 5 single-blind RCTs did not report available data for performing a meta-analysis regarding objective outcomes. Two double-blind RCTs did not report any available data for performing a meta-analysis. Finally, 72 double-blinded RCTs included in our network meta-analysis.

Higgins J, et al. Cochrane Handbook for Systematic Reviews of Interventions version 6.0. wwwtrainingcochraneorg/handbook 2019.

Articles about study that did not meet inclusion criteria (10 articles)

1. Ahmad A, Sheikh S, Khan MA, Chaturvedi A, Patel P, Patel R et al. Endoxifen: A new, protein kinase C inhibitor to treat acute and mixed mania associated with bipolar I disorder. Bipolar Disord 2020.

Reason: study that allowed the antipsychotics as a rescue medication during a trial

2. Berk M, Daglas R, Dandash O, Yucel M, Henry L, Hallam K et al. Quetiapine v. lithium in the maintenance phase following a first episode of mania: randomized controlled trial. Br J Psychiatry 2017; 210(6): 413-421.

Reason: study included patients in the maintenance phase

3. Brown D, Silverstone T, Cookson J. Carbamazepine compared to haloperidol in acute mania. Int Clin Psychopharmacol 1989; 4(3): 229-238.

Reason: study that allowed the antipsychotics as a rescue medication during a trial

4. Chouinard G, Young SN, Annable L. Antimanic effect of clonazepam. Biol Psychiatry 1983; 18(4): 451-466.

Reason: study that allowed the antipsychotics as a rescue medication during a trial

5. Daglas R, Cotton SM, Allott K, Yucel M, Macneil CA, Hasty MK et al. A single-blind, randomised controlled trial on the effects of lithium and quetiapine monotherapy on the trajectory of cognitive functioning in first episode mania: A 12-month follow-up study. Eur Psychiatry 2016; 31: 20-28.

Reason: study included patients in the maintenance phase

6. Garza-Trevino ES, Overall JE, Hollister LE. Verapamil versus lithium in acute mania. Am J Psychiatry 1992; 149(1): 121-122.

Reason: study that allowed the antipsychotics as a rescue medication during a trial

7. Hegerl U, Mergl R, Sander C, Dietzel J, Bitter I, Demyttenaere K et al. A multi-centre, randomised, double-blind, placebo-controlled clinical trial of methylphenidate in the initial treatment of acute mania (MEMAP study). Eur Neuropsychopharmacol 2018; 28(1): 185-194.

Reason: short duration study

8. McElroy SL, Martens BE, Creech RS, Welge JA, Jefferson L, Guerdjikova AI et al. Randomized, double-blind, placebo-controlled study of divalproex extended release loading monotherapy in ambulatory bipolar spectrum disorder patients with moderate-to-severe hypomania or mild mania. J Clin Psychiatry 2010; 71(5): 557-565.

Reason: patients with mild mania

9. Prien RF, Caffey EM, Jr., Klett CJ. Comparison of lithium carbonate and chlorpromazine in the treatment of mania. Report of the Veterans Administration and National Institute of Mental Health Collaborative Study Group. Arch Gen Psychiatry 1972; 26(2): 146-153.

Reason: study included patients with mania and patients with schizoaffective disorder

10. Shafti SS, Shahveisi B. Comparison between lithium and valproate in the treatment of acute mania. J Clin Psychopharmacol 2008; 28(6): 718-720.

Reason: study that allowed the antipsychotics as a rescue medication during a trial

Articles about study that met inclusion criteria in our search (72 articles)

1. Ahmad A, Sheikh S, Shah T, Reddy MS, Prasad B, Verma KK et al. Endoxifen, a New Treatment Option for Mania: A Double-Blind, Active-Controlled Trial Demonstrates the Antimanic Efficacy of Endoxifen. Clin Transl Sci 2016; 9(5): 252-259.

2. Berk M, Ichim L, Brook S. Olanzapine compared to lithium in mania: a double-blind randomized controlled trial. Int Clin Psychopharmacol 1999; 14(6): 339-343.

3. Berwaerts J, Xu H, Nuamah I, Lim P, Hough D. Evaluation of the efficacy and safety of paliperidone extended-release in the treatment of acute mania: a randomized, double-blind, dose-response study. J Affect Disord 2012; 136(1-2): e51-e60.

4. Bowden CL, Brugger AM, Swann AC, Calabrese JR, Janicak PG, Petty F et al. Efficacy of divalproex vs lithium and placebo in the treatment of mania. The Depakote Mania Study Group. JAMA 1994; 271(12): 918-924.

5. Bowden CL, Grunze H, Mullen J, Brecher M, Paulsson B, Jones M et al. A randomized, double-blind, placebo-controlled efficacy and safety study of quetiapine or lithium as monotherapy for mania in bipolar disorder. J Clin Psychiatry 2005; 66(1): 111-121.

6. Bowden CL, Swann AC, Calabrese JR, Rubenfaer LM, Wozniak PJ, Collins MA et al. A randomized, placebo-controlled, multicenter study of divalproex sodium extended release in the treatment of acute mania. J Clin Psychiatry 2006; 67(10): 1501-1510.

7. Calabrese JR, Keck PE, Jr., Starace A, Lu K, Ruth A, Laszlovszky I et al. Efficacy and safety of low- and high-dose cariprazine in acute and mixed mania associated with bipolar I disorder: a double-blind, placebo-controlled study. J Clin Psychiatry 2015; 76(3): 284-292.

8. Clark M, Berk M, Brook S. A randomized controlled single blind study of the efficacy of clonazepam and lithium in the treatment of acute mania. Human Psychopharmacology: Clinical and Experimental 1997; (July/August): 325-328.

9. Cutler AJ, Datto C, Nordenhem A, Minkwitz M, Acevedo L, Darko D. Extended-release quetiapine as monotherapy for the treatment of adults with acute mania: a randomized, double-blind, 3-week trial. Clin Ther 2011; 33(11): 1643-1658.

10. Durgam S, Starace A, Li D, Migliore R, Ruth A, Nemeth G et al. The efficacy and tolerability of cariprazine in acute mania associated with bipolar I disorder: a phase II trial. Bipolar Disord 2015; 17(1): 63-75.

11. El Mallakh RS, Vieta E, Rollin L, Marcus R, Carson WH, McQuade R. A comparison of two fixed doses of aripiprazole with placebo in acutely relapsed, hospitalized patients with bipolar disorder I (manic or mixed) in subpopulations (CN138-007). Eur Neuropsychopharmacol 2010; 20(11): 776-783.

12. Feifel D, Galangue B, Macdonald K, Cobb P, Dinca A, Becker O et al. A Naturalistic, Single-blind Comparison of Rapid Dose Administration of Divalproex ER Versus

Quetiapine in Patients with Acute Bipolar Mania. Innov Clin Neurosci 2011; 8(1): 29-35.

13. Freeman TW, Clothier JL, Pazzaglia P, Lesem MD, Swann AC. A double-blind comparison of valproate and lithium in the treatment of acute mania. Am J Psychiatry 1992; 149(1): 108-111.

14. Garfinkel PE, Stancer HC, Persad E. A comparison of haloperidol, lithium carbonate and their combination in the treatment of mania. J Affect Disord 1980; 2(4): 279-288.

15. Goldsmith DR, Wagstaff AJ, Ibbotson T, Perry CM. Lamotrigine: a review of its use in bipolar disorder. Drugs 2003; 63(19): 2029-2050.

16. Gouliaev G, Licht RW, Vestergaard P, Merinder L, Lund H, Bjerre L. Treatment of manic episodes: zuclopenthixol and clonazepam versus lithium and clonazepam. Acta Psychiatr Scand 1996; 93(2): 119-124.

17. Grunze H, Kotlik E, Costa R, Nunes T, Falcao A, Almeida L et al. Assessment of the efficacy and safety of eslicarbazepine acetate in acute mania and prevention of recurrence: experience from multicentre, double-blind, randomised phase II clinical studies in patients with bipolar disorder I. J Affect Disord 2015; 174: 70-82.

18. Hirschfeld RM, Allen MH, McEvoy JP, Keck PE, Jr., Russell JM. Safety and tolerability of oral loading divalproex sodium in acutely manic bipolar patients. J Clin Psychiatry 1999; 60(12): 815-818.

19. Hirschfeld RM, Bowden CL, Vigna NV, Wozniak P, Collins M. A randomized, placebo-controlled, multicenter study of divalproex sodium extended-release in the acute treatment of mania. J Clin Psychiatry 2010; 71(4): 426-432.

20. Hirschfeld RM, Keck PE, Jr., Kramer M, Karcher K, Canuso C, Eerdekens M et al. Rapid antimanic effect of risperidone monotherapy: a 3-week multicenter, double-blind, placebo-controlled trial. Am J Psychiatry 2004; 161(6): 1057-1065.

21. Janicak PG, Sharma RP, Pandey G, Davis JM. Verapamil for the treatment of acute mania: a double-blind, placebo-controlled trial. Am J Psychiatry 1998; 155(7): 972-973.

22. Kakkar AK, Rehan HS, Unni KE, Gupta NK, Chopra D, Kataria D. Comparative efficacy and safety of oxcarbazepine versus divalproex sodium in the treatment of acute mania: a pilot study. Eur Psychiatry 2009; 24(3): 178-182.

23. Kanba S, Kawasaki H, Ishigooka J, Sakamoto K, Kinoshita T, Kuroki T. A placebo-controlled, double-blind study of the efficacy and safety of aripiprazole for the treatment of acute manic or mixed episodes in Asian patients with bipolar I disorder (the AMAZE study). World J Biol Psychiatry 2014; 15(2): 113-121.

24. Katagiri H, Takita Y, Tohen M, Higuchi T, Kanba S, Takahashi M. Efficacy and safety of olanzapine in the treatment of Japanese patients with bipolar I disorder in a current manic or mixed episode: a randomized, double-blind, placebo- and haloperidol-controlled study. J Affect Disord 2012; 136(3): 476-484.

25. Keck PE, Jr., Marcus R, Tourkodimitris S, Ali M, Liebeskind A, Saha A et al. A placebo-controlled, double-blind study of the efficacy and safety of aripiprazole in patients with acute bipolar mania. Am J Psychiatry 2003; 160(9): 1651-1658.

26. Keck PE, Orsulak PJ, Cutler AJ, Sanchez R, Torbeyns A, Marcus RN et al. Aripiprazole monotherapy in the treatment of acute bipolar I mania: a randomized, double-blind, placebo- and lithium-controlled study. J Affect Disord 2009; 112(1-3): 36-49.

27. Keck PE, Jr., Versiani M, Potkin S, West SA, Giller E, Ice K et al. Ziprasidone in the treatment of acute bipolar mania: a three-week, placebo-controlled, double-blind, randomized trial. Am J Psychiatry 2003; 160(4): 741-748.

28. Khan N, Nasar A, Bajwa S, Jawad Butt A, Azher A, Mushtaq T et al. TULIP study: Trail of Lurasidone in bipolar disorder in Pakistan. Saudi J Biol Sci 2021; 28(1): 1128-1132.

29. Khanna S, Vieta E, Lyons B, Grossman F, Eerdekens M, Kramer M. Risperidone in the treatment of acute mania: double-blind, placebo-controlled study. Br J Psychiatry 2005; 187: 229-234.

30. Kushner SF, Khan A, Lane R, Olson WH. Topiramate monotherapy in the management of acute mania: results of four double-blind placebo-controlled trials. Bipolar Disord 2006; 8(1): 15-27.

31. Landbloom RL, Mackle M, Wu X, Kelly L, Snow-Adami L, McIntyre RS et al. Asenapine: Efficacy and safety of 5 and 10mg bid in a 3-week, randomized, double-blind, placebo-controlled trial in adults with a manic or mixed episode associated with bipolar I disorder. J Affect Disord 2016; 190: 103-110.

32. Lerer B, Moore N, Meyendorff E, Cho SR, Gershon S. Carbamazepine versus lithium in mania: a double-blind study. J Clin Psychiatry 1987; 48(3): 89-93.

33. Li H, Ma C, Wang G, Zhu X, Peng M, Gu N. Response and remission rates in Chinese patients with bipolar mania treated for 4 weeks with either quetiapine or lithium: a randomized and double-blind study. Curr Med Res Opin 2008; 24(1): 1-10.

34. McElroy SL, Keck PE, Stanton SP, Tugrul KC, Bennett JA, Strakowski SM. A randomized comparison of divalproex oral loading versus haloperidol in the initial treatment of acute psychotic mania. J Clin Psychiatry 1996; 57(4): 142-146.

35. McIntyre RS, Brecher M, Paulsson B, Huizar K, Mullen J. Quetiapine or haloperidol as monotherapy for bipolar mania--a 12-week, double-blind, randomised, parallel-group, placebo-controlled trial. Eur Neuropsychopharmacol 2005; 15(5): 573-585.

36. McIntyre RS, Cohen M, Zhao J, Alphs L, Macek TA, Panagides J. A 3-week, randomized, placebo-controlled trial of asenapine in the treatment of acute mania in bipolar mania and mixed states. Bipolar Disord 2009; 11(7): 673-686.

37. McIntyre RS, Cohen M, Zhao J, Alphs L, Macek TA, Panagides J. Asenapine in the treatment of acute mania in bipolar I disorder: a randomized, double-blind, placebo-controlled trial. J Affect Disord 2010; 122(1-2): 27-38.

38. NCT03257865. A Trial to Assess Brexpiprazole Versus Placebo for the Treatment of Acute Manic Episodes Associated With Bipolar I Disorde. ClinicalTrialsgov 1997.

39. NCT03259555. A Trial to Assess Brexpiprazole Versus Placebo for the Treatment of Acute Manic Episodes Associated With Bipolar I Disorde. ClinicalTrialsgov 1997.

40. Niufan G, Tohen M, Qiuqing A, Fude Y, Pope E, McElroy H et al. Olanzapine versus lithium in the acute treatment of bipolar mania: a double-blind, randomized,

controlled trial. J Affect Disord 2008; 105(1-3): 101-108.

41. Perlis RH, Baker RW, Zarate CA, Jr., Brown EB, Schuh LM, Jamal HH et al. Olanzapine versus risperidone in the treatment of manic or mixed States in bipolar I disorder: a randomized, double-blind trial. J Clin Psychiatry 2006; 67(11): 1747-1753.

42. Pope HG, Jr., McElroy SL, Keck PE, Jr., Hudson JI. Valproate in the treatment of acute mania. A placebo-controlled study. Arch Gen Psychiatry 1991; 48(1): 62-68.

43. Potkin SG, Keck PE, Jr., Segal S, Ice K, English P. Ziprasidone in acute bipolar mania: a 21-day randomized, double-blind, placebo-controlled replication trial. J Clin Psychopharmacol 2005; 25(4): 301-310.

44. Rezayat AA, Hebrani P, Behdani F, Salaran M, Marvast MN. Comparison the effectiveness of aripiprazole and risperidone for the treatment of acute bipolar mania. J Res Med Sci 2014; 19(8): 733-738.

45. Sachs G, Sanchez R, Marcus R, Stock E, McQuade R, Carson W et al. Aripiprazole in the treatment of acute manic or mixed episodes in patients with bipolar I disorder: a 3-week placebo-controlled study. J Psychopharmacol 2006; 20(4): 536-546.

46. Sachs GS, Greenberg WM, Starace A, Lu K, Ruth A, Laszlovszky I et al. Cariprazine in the treatment of acute mania in bipolar I disorder: a double-blind, placebo-controlled, phase III trial. J Affect Disord 2015; 174: 296-302.

47. Segal J, Berk M, Brook S. Risperidone compared with both lithium and haloperidol in mania: a double-blind randomized controlled trial. Clin Neuropharmacol 1998; 21(3): 176-180.

48. Shafti SS. Olanzapine vs. lithium in management of acute mania. J Affect Disord 2010; 122(3): 273-276.

49. Shafti SS. Aripiprazole Versus Lithium in Management of Acute Mania: a Randomized Clinical Trial. East Asian Arch Psychiatry 2018; 28(3): 80-84.

50. Shafti SS, Kaviani H. Extended-release carbamazepine versus lithium in management of acute mania in male inpatients with bipolar I disorder. Psychiatry and Clinical Psychopharmacology 2018; 28(4): 371-377.

51. Shopsin B, Gershon S, Thompson H, Collins P. Psychoactive drugs in mania. A controlled comparison of lithium carbonate, chlorpromazine, and haloperidol. Arch Gen Psychiatry 1975; 32(1): 34-42.

52. Singh M, Kaur S, Sehgal VK. Comparative study of electrocardiographic changes in patients of acute mania receiving verapamil or lithium carbonate. J Clin Diagn Res 2014; 8(12): HC08-11.

53. Small JG, Klapper MH, Milstein V, Kellams JJ, Miller MJ, Marhenke JD et al. Carbamazepine compared with lithium in the treatment of mania. Arch Gen Psychiatry 1991; 48(10): 915-921.

54. Smulevich AB, Khanna S, Eerdekens M, Karcher K, Kramer M, Grossman F. Acute and continuation risperidone monotherapy in bipolar mania: a 3-week placebo-controlled trial followed by a 9-week double-blind trial of risperidone and haloperidol. Eur Neuropsychopharmacol 2005; 15(1): 75-84.

55. Suppes T, Kelly DI, Hynan LS, Snow DE, Sureddi S, Foster B et al. Comparison of two anticonvulsants in a randomized, single-blind treatment of hypomanic symptoms in patients with bipolar disorder. Aust N Z J Psychiatry 2007; 41(5): 397-402.

56. Tohen M, Baker RW, Altshuler LL, Zarate CA, Suppes T, Ketter TA et al. Olanzapine versus divalproex in the treatment of acute mania. Am J Psychiatry 2002; 159(6): 1011-1017.

57. Tohen M, Goldberg JF, Gonzalez-Pinto Arrillaga AM, Azorin JM, Vieta E, Hardy-Bayle MC et al. A 12-week, double-blind comparison of olanzapine vs haloperidol in the treatment of acute mania. Arch Gen Psychiatry 2003; 60(12): 1218-1226.

58. Tohen M, Jacobs TG, Grundy SL, McElroy SL, Banov MC, Janicak PG et al. Efficacy of olanzapine in acute bipolar mania: a double-blind, placebo-controlled study. The Olanzipine HGGW Study Group. Arch Gen Psychiatry 2000; 57(9): 841-849.

59. Tohen M, Sanger TM, McElroy SL, Tollefson GD, Chengappa KN, Daniel DG et al. Olanzapine versus placebo in the treatment of acute mania. Olanzapine HGEH Study Group. Am J Psychiatry 1999; 156(5): 702-709.

60. Tohen M, Vieta E, Goodwin GM, Sun B, Amsterdam JD, Banov M et al. Olanzapine versus divalproex versus placebo in the treatment of mild to moderate mania: a randomized, 12-week, double-blind study. J Clin Psychiatry 2008; 69(11): 1776-1789.

61. Vasudev K, Goswami U, Kohli K. Carbamazepine and valproate monotherapy: feasibility, relative safety and efficacy, and therapeutic drug monitoring in manic disorder. Psychopharmacology (Berl) 2000; 150(1): 15-23.

62. Vieta E, Bourin M, Sanchez R, Marcus R, Stock E, McQuade R et al. Effectiveness of aripiprazole v. haloperidol in acute bipolar mania: double-blind, randomised, comparative 12-week trial. Br J Psychiatry 2005; 187: 235-242.

63. Vieta E, Nuamah IF, Lim P, Yuen EC, Palumbo JM, Hough DW et al. A randomized, placebo- and active-controlled study of paliperidone extended release for the treatment of acute manic and mixed episodes of bipolar I disorder. Bipolar Disord 2010; 12(3): 230-243.

64. Vieta E, Ramey T, Keller D, English PA, Loebel AD, Miceli J. Ziprasidone in the treatment of acute mania: a 12-week, placebo-controlled, haloperidol-referenced study. J Psychopharmacol 2010; 24(4): 547-558.

65. Weiser M, Levi L, Levine SZ, Bialer M, Shekh-Ahmad T, Matei V et al. A randomized, double-blind, placebo- and risperidone-controlled study on valnoctamide for acute mania. Bipolar Disord 2017; 19(4): 285-294.

66. Weisler RH, Kalali AH, Ketter TA, Group SPDS. A multicenter, randomized, double-blind, placebo-controlled trial of extended-release carbamazepine capsules as monotherapy for bipolar disorder patients with manic or mixed episodes. J Clin Psychiatry 2004; 65(4): 478-484.

67. Weisler RH, Keck PE, Jr., Swann AC, Cutler AJ, Ketter TA, Kalali AH et al. Extended-release carbamazepine capsules as monotherapy for acute mania in bipolar disorder: a multicenter, randomized, double-blind, placebo-controlled trial. J Clin Psychiatry 2005; 66(3): 323-330.

68. Xu L, Lu Y, Yang Y, Zheng Y, Chen F, Lin Z. Olanzapine-valproate combination versus olanzapine or valproate monotherapy in the treatment of bipolar I mania: a randomized controlled study in a Chinese population group. Neuropsychiatr Dis Treat 2015; 11: 1265-1271.

69. Yildiz A, Guleryuz S, Ankerst DP, Ongur D, Renshaw PF. Protein kinase C inhibition in the treatment of mania: a double-blind, placebo-controlled trial of tamoxifen. Arch Gen Psychiatry 2008; 65(3): 255-263.

70. Young AH, Oren DA, Lowy A, McQuade RD, Marcus RN, Carson WH et al. Aripiprazole monotherapy in acute mania: 12-week randomised placebo- and haloperidol-controlled study. Br J Psychiatry 2009; 194(1): 40-48.

71. Zajecka JM, Weisler R, Sachs G, Swann AC, Wozniak P, Sommerville KW. A comparison of the efficacy, safety, and tolerability of divalproex sodium and olanzapine in the treatment of bipolar disorder. J Clin Psychiatry 2002; 63(12): 1148-1155.

72. Zarate CA, Jr., Singh JB, Carlson PJ, Quiroz J, Jolkovsky L, Luckenbaugh DA et al. Efficacy of a protein kinase C inhibitor (tamoxifen) in the treatment of acute mania: a pilot study. Bipolar Disord 2007; 9(6): 561-570.

Review articles used in the hand search (24 articles)

1. Bai Y, Yang H, Chen G, Gao K. Acceptability of Acute and Maintenance Pharmacotherapy of Bipolar Disorder: A Systematic Review of Randomized, Double-Blind, Placebo-Controlled Clinical Trials. J Clin Psychopharmacol 2020; 40(2): 167-179.

2. Baldessarini RJ, Tondo L, Vazquez GH. Pharmacological treatment of adult bipolar disorder. Mol Psychiatry 2019; 24(2): 198-217.

3. Bartoli F, Clerici M, Di Brita C, Riboldi I, Crocamo C, Carra G. Effect of clinical response to active drugs and placebo on antipsychotics and mood stabilizers relative efficacy for bipolar depression and mania: A meta-regression analysis. J Psychopharmacol 2018; 32(4): 416-422.

4. Brown R, Taylor MJ, Geddes J. Aripiprazole alone or in combination for acute mania. Cochrane Database Syst Rev 2013; (12): CD005000.

5. Butler M, Urosevic S, Desai P, Sponheim SR, Popp J, Nelson VA et al. Treatment for Bipolar Disorder in Adults: A Systematic Review: Rockville (MD), 2018.

6. Chang HY, Tseng PT, Stubbs B, Chu CS, Li DJ, Fornaro M et al. The efficacy and tolerability of paliperidone in mania of bipolar disorder: A preliminary meta-analysis. Exp Clin Psychopharmacol 2017; 25(5): 422-433.

7. Cipriani A, Barbui C, Salanti G, Rendell J, Brown R, Stockton S et al. Comparative efficacy and acceptability of antimanic drugs in acute mania: a multiple-treatments meta-analysis. Lancet 2011; 378(9799): 1306-1315.

8. Cipriani A, Rendell JM, Geddes JR. Haloperidol alone or in combination for acute mania. Cochrane Database Syst Rev 2006; (3): CD004362.

9. Citrome L, Landbloom R, Chang CT, Earley W. Effects of asenapine on agitation and hostility in adults with acute manic or mixed episodes associated with bipolar I disorder. Neuropsychiatr Dis Treat 2017; 13: 2955-2963.

10. Glue P, Herbison P. Comparative efficacy and acceptability of combined antipsychotics and mood stabilizers versus individual drug classes for acute mania: Network meta-analysis. Aust N Z J Psychiatry 2015; 49(12): 1215-1220.
11. Jochim J, Rifkin-Zybutz RP, Geddes J, Cipriani A. Valproate for acute mania. Cochrane Database Syst Rev 2019; 10: CD004052.
12. Lao KS, He Y, Wong IC, Besag FM, Chan EW. Tolerability and Safety Profile of Cariprazine in Treating Psychotic Disorders, Bipolar Disorder and Major Depressive Disorder: A Systematic Review with Meta-Analysis of Randomized Controlled Trials. CNS Drugs 2016; 30(11): 1043-1054.
13. Li DJ, Tseng PT, Stubbs B, Chu CS, Chang HY, Vieta E et al. Efficacy, safety and tolerability of aripiprazole in bipolar disorder: An updated systematic review and meta-analysis of randomized controlled trials. Prog Neuropsychopharmacol Biol Psychiatry 2017; 79(Pt B): 289-301.
14. McKnight RF, de La Motte de Broons de Vauvert S, Chesney E, Amit BH, Geddes J, Cipriani A. Lithium for acute mania. Cochrane Database Syst Rev 2019; 6: CD004048.
15. Pigott K, Galizia I, Vasudev K, Watson S, Geddes J, Young AH. Topiramate for acute affective episodes in bipolar disorder in adults. Cochrane Database Syst Rev 2016; 9: CD003384.
16. Rendell JM, Gijsman HJ, Bauer MS, Goodwin GM, Geddes GR. Risperidone alone or in combination for acute mania. Cochrane Database Syst Rev 2006; (1): CD004043.
17. Rendell JM, Gijsman HJ, Keck P, Goodwin GM, Geddes JR. Olanzapine alone or in combination for acute mania. Cochrane Database Syst Rev 2003; (3): CD004040.
18. Scheidemantel T, Korobkova I, Rej S, Sajatovic M. Asenapine for bipolar disorder. Neuropsychiatr Dis Treat 2015; 11: 3007-3017.
19. Talaei A, Pourgholami M, Khatibi-Moghadam H, Faridhosseini F, Farhoudi F, Askari-Noghani A et al. Tamoxifen: A Protein Kinase C Inhibitor to Treat Mania: A Systematic Review and Meta-Analysis of Randomized, Placebo-Controlled Trials. J Clin Psychopharmacol 2016; 36(3): 272-275.
20. Vasudev A, Macritchie K, Vasudev K, Watson S, Geddes J, Young AH. Oxcarbazepine for acute affective episodes in bipolar disorder. Cochrane Database Syst Rev 2011; (12): CD004857.
21. Vieta E, Montes JM. A Review of Asenapine in the Treatment of Bipolar Disorder. Clin Drug Investig 2018; 38(2): 87-99.
22. Yildiz A, Nikodem M, Vieta E, Correll CU, Baldessarini RJ. A network meta-analysis on comparative efficacy and all-cause discontinuation of antimanic treatments in acute bipolar mania. Psychol Med 2015; 45(2): 299-317.
23. Yildiz A, Vieta E, Leucht S, Baldessarini RJ. Efficacy of antimanic treatments: meta-analysis of randomized, controlled trials. Neuropsychopharmacology 2011; 36(2): 375-389.
24. Yildiz A, Vieta E, Tohen M, Baldessarini RJ. Factors modifying drug and placebo responses in randomized trials for bipolar mania. Int J Neuropsychopharmacol 2011; 14(7): 863-875.

Articles regarding study found from the previous review articles (2 articles)

1. Bowden C, Calabrese J, Ascher J, DeVeaugh-Geiss J, Earl N, Evoniuk G et al. Spectrum of efficacy of Lamotrigine in bipolar disorder: Overview of double-blind, placebo-controlled studies. American College of Neuropsychopharmacology 39th Annual Meeting, San Juan, Puerto Rico 2000.

2. NCT00099229. Study of Licarbazepine in the Treatment of Manic Episodes of Bipolar Disorder.

Studies not included in the network meta-analysis (7 studies)

1. Clark M, Berk M, Brook S. A randomized controlled single blind study of the efficacy of clonazepam and lithium in the treatment of acute mania. Human Psychopharmacology: Clinical and Experimental 1997; (July/August): 325-328. (single-blind study; no available data for objective outcomes reported)

2. Feifel D, Galangue B, Macdonald K, Cobb P, Dinca A, Becker O et al. A Naturalistic, Single-blind Comparison of Rapid Dose Administration of Divalproex ER Versus Quetiapine in Patients with Acute Bipolar Mania. Innov Clin Neurosci 2011; 8(1): 29-35. (single-blind study; no available data for objective outcomes reported)

3. Gouliaev G, Licht RW, Vestergaard P, Merinder L, Lund H, Bjerre L. Treatment of manic episodes: zuclopenthixol and clonazepam versus lithium and clonazepam. Acta Psychiatr Scand 1996; 93(2): 119-124. (single-blind study; no available data for objective outcomes reported)

4. Khan N, Nasar A, Bajwa S, Jawad Butt A, Azher A, Mushtaq T et al. TULIP study: Trail of Lurasidone in bipolar disorder in Pakistan. Saudi J Biol Sci 2021; 28(1): 1128-1132. (no available data reported)

5. McElroy SL, Keck PE, Stanton SP, Tugrul KC, Bennett JA, Strakowski SM. A randomized comparison of divalproex oral loading versus haloperidol in the initial treatment of acute psychotic mania. J Clin Psychiatry 1996; 57(4): 142-146. (single-blind study; no available data for objective outcomes reported)

6. Singh M, Kaur S, Sehgal VK. Comparative study of electrocardiographic changes in patients of acute mania receiving verapamil or lithium carbonate. J Clin Diagn Res 2014; 8(12): HC08-11. (no available data reported)

7. Suppes T, Kelly DI, Hynan LS, Snow DE, Sureddi S, Foster B et al. Comparison of two anticonvulsants in a randomized, single-blind treatment of hypomanic symptoms in patients with bipolar disorder. Aust N Z J Psychiatry 2007; 41(5): 397-402. (single-blind study; no available data for objective outcomes reported)

Supplementary Figure 2. Risk of bias summary.

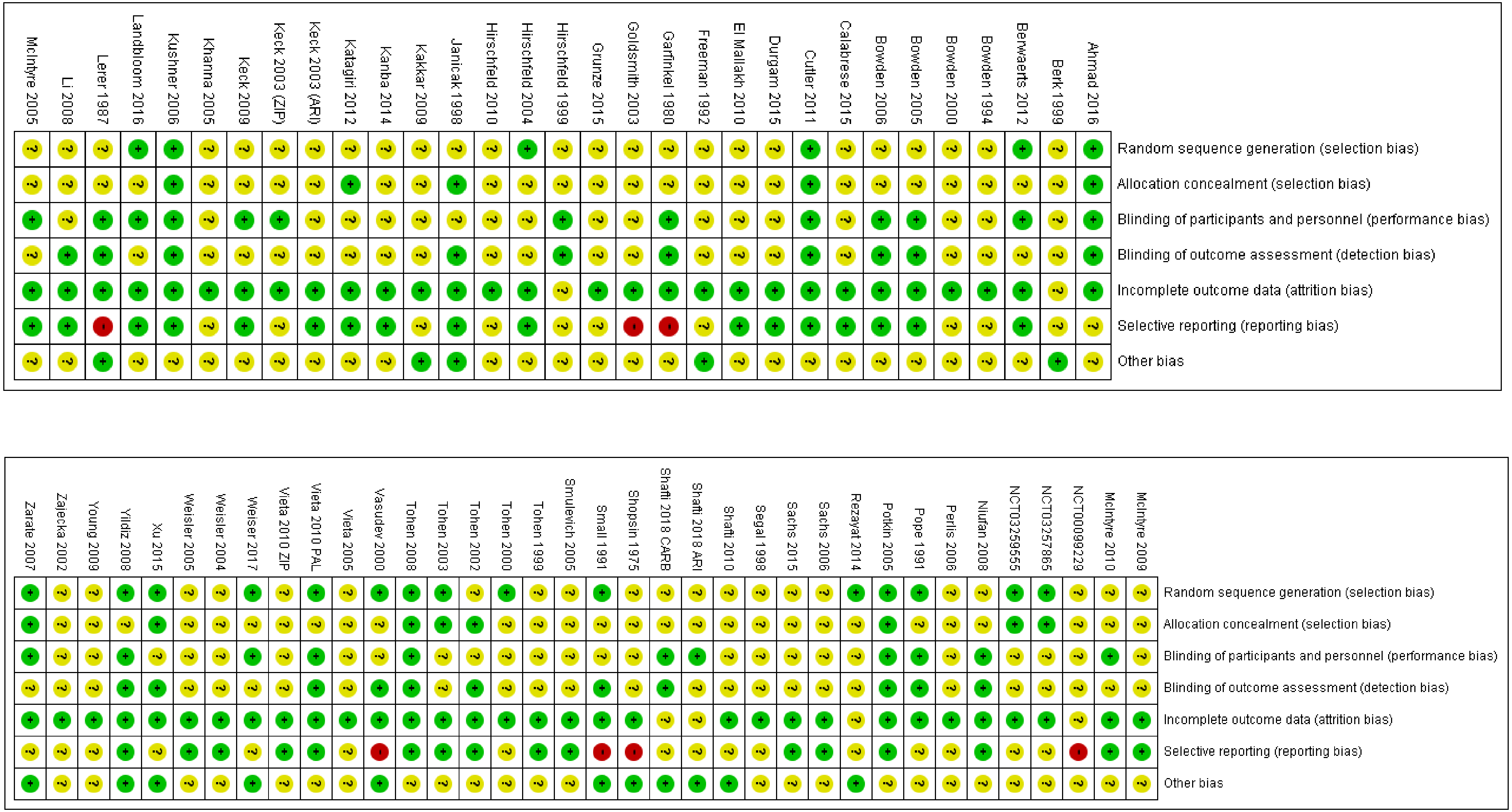

Risk of bias in RCTs for the main outcomes was assessed independently using the Cochrane Collaboration's risk of bias tool.

Higgins J, et al. Cochrane Handbook for Systematic Reviews of Interventions version 6.0. wwwtrainingcochraneorg/handbook 2019.

Selective reporting: We focused on two primary outcomes (response to treatment and all-cause discontinuation). We also focused on mania symptoms because most of the definitions for the treatment response used the mania rating scale score. The domain was as rated high risk if a study reported data on only one outcome and unclear risk if a study reported data on two outcomes.

Other bias: The domain was rated as low risk is the study was non-industry-sponsored and unclear if the person related to industry was involved in the study.

We classified an overall risk of bias for every study based on the individual risk of bias items. The overall risk of bias was classified into high, moderate, or low.

The classification is based on the following article.

Furukawa TA, et al., Comparative efficacy and acceptability of first-generation and second-generation antidepressants in the acute treatment of major depression: protocol for a network meta-analysis. BMJ Open. 2016 Jul 8;6(7):e010919.

Supplementary Figure 3. Two dimensional plot.

Supplementary Figure 3-1. Two-dimensional plot including all drugs.

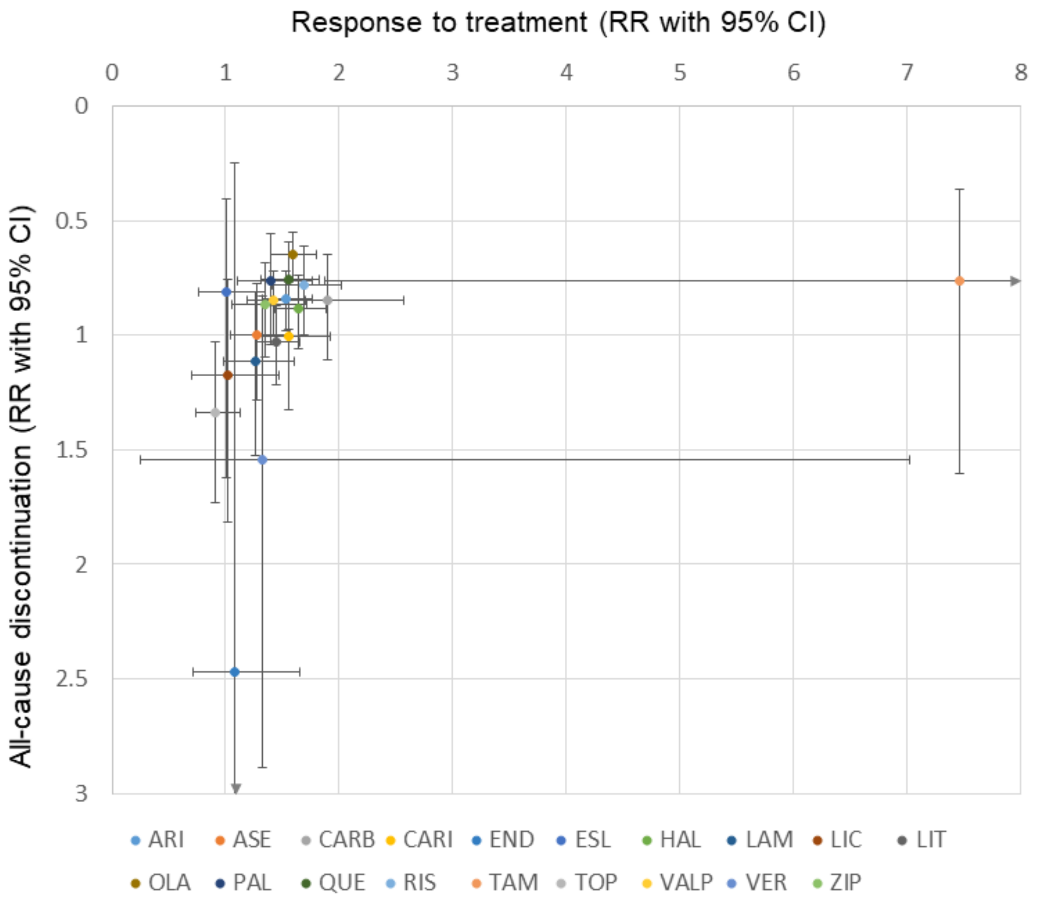

95% CI: 95% confidence interval, ARI: aripiprazole; ASE: asenapine; CARB: carbamazepine; CARI: cariprazine; END: endoxifen, ESL: eslicarbazepine; HAL: haloperidol; LAM: lamotrigine; LIC: licarbazepine; LIT: lithium; OLA: olanzapine; PAL: paliperidone; QUE: quetiapine; RIS: risperidone; RR: risk ratio; TAM: tamoxifen; TOP: topiramate; VALP: valproate; VER: verapamil; ZIP: ziprasidone

Supplementary Figure 3-2. Two-dimensional plot excluding endoxifen and tamoxifen

The effect size of tamoxifen for the response to treatment was an outlier, as was the effect size of endoxifen for all-cause discontinuation.

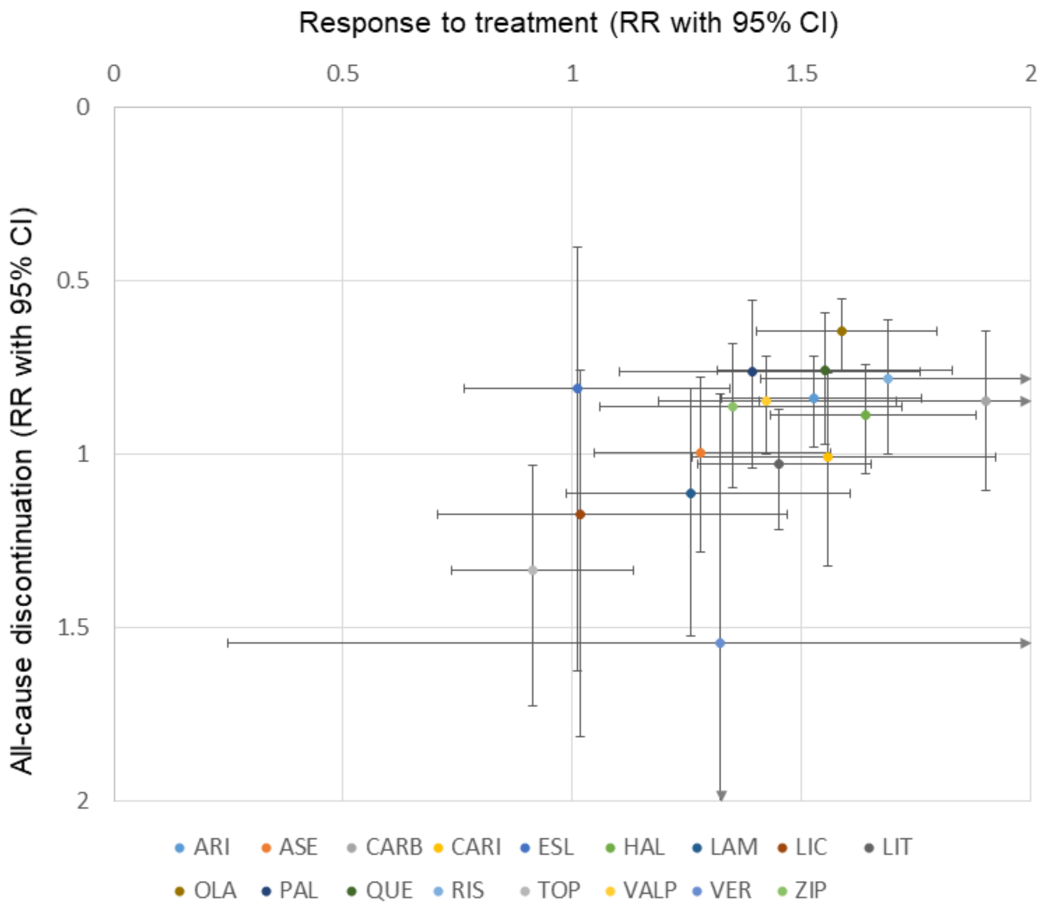

95% CI: 95% confidence interval, ARI: aripiprazole; ASE: asenapine; CARB: carbamazepine; CARI: cariprazine; ESL: eslicarbazepine; HAL: haloperidol; LAM: lamotrigine; LIC: licarbazepine; LIT: lithium; OLA: olanzapine; PAL: paliperidone; QUE: quetiapine; RIS: risperidone; RR: risk ratio; TOP: topiramate; VALP: valproate; VER: verapamil; ZIP: ziprasidone

Supplementary Table 1. PRISMA for Network Meta-Analyses Checklist.

Section/Topic	Item #	Checklist Item	Reported on Page #
**TITLE**			
Title	1	Identify the report as a systematic review *incorporating a network meta-analysis (or related form of meta-analysis).*	1
**ABSTRACT**			
Structured summary	2	Provide a structured summary including, as applicable:<br>**Background:** main objectives<br>**Methods:** data sources; study eligibility criteria, participants, and interventions; study appraisal; and *synthesis methods, such as network meta-analysis.*<br>**Results:** number of studies and participants identified; summary estimates with corresponding confidence/credible intervals; *treatment rankings may also be discussed. Authors may choose to summarize pairwise comparisons against a chosen treatment included in their analyses for brevity.*<br>**Discussion/Conclusions:** limitations; conclusions and implications of findings.<br>**Other:** primary source of funding; systematic review registration number with registry name.	3
**INTRODUCTION**			
Rationale	3	Describe the rationale for the review in the context of what is already known*, including mention of why a network meta-analysis has been conducted.*_	5
Objectives	4	Provide an explicit statement of questions being addressed, with reference to participants, interventions, comparisons, outcomes, and study design (PICOS).	5
**METHODS**			

Protocol and registration	5	Indicate whether a review protocol exists and if and where it can be accessed (e.g., Web address); and, if available, provide registration information, including registration number.	5
Eligibility criteria	6	Specify study characteristics (e.g., PICOS, length of follow-up) and report characteristics (e.g., years considered, language, publication status) used as criteria for eligibility, giving rationale. *Clearly describe eligible treatments included in the treatment network, and note whether any have been clustered or merged into the same node (with justification).*	6
Information sources	7	Describe all information sources (e.g., databases with dates of coverage, contact with study authors to identify additional studies) in the search and date last searched.	6
Search	8	Present full electronic search strategy for at least one database, including any limits used, such that it could be repeated.	6
Study selection	9	State the process for selecting studies (i.e., screening, eligibility, included in systematic review, and, if applicable, included in the meta-analysis).	6
Data collection process	10	Describe method of data extraction from reports (e.g., piloted forms, independently, in duplicate) and any processes for obtaining and confirming data from investigators.	6
Data items	11	List and define all variables for which data were sought (e.g., PICOS, funding sources) and any assumptions and simplifications made.	6
**Geometry of the network**	**S1**	Describe methods used to explore the geometry of the treatment network under study and potential biases related to it. This should include how the evidence base has been graphically summarized for presentation, and what characteristics were compiled and used to describe the evidence base to readers.	7
Risk of bias within individual studies	12	Describe methods used for assessing risk of bias of individual studies (including specification of whether this was done at the study or outcome level), and how this information is to be used in any data synthesis.	7
Summary measures	13	State the principal summary measures (e.g., risk ratio, difference in means). *Also describe the use of additional summary measures assessed, such as treatment rankings and surface under the cumulative ranking curve (SUCRA)* values, as well as modified approaches used to present summary findings from meta-analyses.*	7
Planned methods of analysis	14	Describe the methods of handling data and combining results of studies for each network meta-analysis. This should include, but not be limited to:<br>• *Handling of multi-arm trials;*<br>• *Selection of variance structure;*	7

		• *Selection of prior distributions in Bayesian analyses; and*<br>• *Assessment of model fit.*	
**Assessment of Inconsistency**	**S2**	Describe the statistical methods used to evaluate the agreement of direct and indirect evidence in the treatment network(s) studied. Describe efforts taken to address its presence when found.	7
Risk of bias across studies	15	Specify any assessment of risk of bias that may affect the cumulative evidence (e.g., publication bias, selective reporting within studies).	7
Additional analyses	16	Describe methods of additional analyses if done, indicating which were pre-specified. This may include, but not be limited to, the following:<br>• Sensitivity or subgroup analyses;<br>• Meta-regression analyses;<br>• *Alternative formulations of the treatment network; and*<br>• *Use of alternative prior distributions for Bayesian analyses (if applicable).*_	7
**RESULTS†**			
Study selection	17	Give numbers of studies screened, assessed for eligibility, and included in the review, with reasons for exclusions at each stage, ideally with a flow diagram.	9
**Presentation of network structure**	**S3**	Provide a network graph of the included studies to enable visualization of the geometry of the treatment network.	9
**Summary of network geometry**	**S4**	Provide a brief overview of characteristics of the treatment network. This may include commentary on the abundance of trials and randomized patients for the different interventions and pairwise comparisons in the network, gaps of evidence in the treatment network, and potential biases reflected by the network structure.	9

Study characteristics	18	For each study, present characteristics for which data were extracted (e.g., study size, PICOS, follow-up period) and provide the citations.	9
Risk of bias within studies	19	Present data on risk of bias of each study and, if available, any outcome level assessment.	9
Results of individual studies	20	For all outcomes considered (benefits or harms), present, for each study: 1) simple summary data for each intervention group, and 2) effect estimates and confidence intervals. *Modified approaches may be needed to deal with information from larger networks.*	9
Synthesis of results	21	Present results of each meta-analysis done, including confidence/credible intervals. *In larger networks, authors may focus on comparisons versus a particular comparator (e.g. placebo or standard care), with full findings presented in an appendix. League tables and forest plots may be considered to summarize pairwise comparisons.* If additional summary measures were explored (such as treatment rankings), these should also be presented.	9
**Exploration for inconsistency**	**S5**	Describe results from investigations of inconsistency. This may include such information as measures of model fit to compare consistency and inconsistency models, *P* values from statistical tests, or summary of inconsistency estimates from different parts of the treatment network.	9
Risk of bias across studies	22	Present results of any assessment of risk of bias across studies for the evidence base being studied.	9
Results of additional analyses	23	Give results of additional analyses, if done (e.g., sensitivity or subgroup analyses, meta-regression analyses, *alternative network geometries studied, alternative choice of prior distributions for Bayesian analyses,* and so forth).	9
**DISCUSSION**			
Summary of evidence	24	Summarize the main findings, including the strength of evidence for each main outcome; consider their relevance to key groups (e.g., healthcare providers, users, and policy-makers).	17
Limitations	25	Discuss limitations at study and outcome level (e.g., risk of bias), and at review level (e.g., incomplete retrieval of identified research, reporting bias). *Comment on the validity of the assumptions, such as transitivity and consistency. Comment on any concerns regarding network geometry (e.g., avoidance of certain comparisons).*	19

Conclusions	26	Provide a general interpretation of the results in the context of other evidence, and implications for future research.	20
**FUNDING**			20
Funding	27	Describe sources of funding for the systematic review and other support (e.g., supply of data); role of funders for the systematic review. This should also include information regarding whether funding has been received from manufacturers of treatments in the network and/or whether some of the authors are content experts with professional conflicts of interest that could affect use of treatments in the network.	

*We performed frequentist network meta-analysis. Rücker and Schwarzer suggest a frequentist analogue to SUCRA which they call P-score that works without resampling (Rücker and Schwarzer, 2015). Therefore, we calculated P-scores to explore the potential order of treatment hierarchy. The P-score has been shown to be equivalent to the SUCRA score (Rücker and Schwarzer, 2015). The P-scores would be 1 when a treatment is certain to be the best and 0 when a treatment is certain to be the worst, with values ranging from 1 (the best intervention) to 0 (the worst intervention).

Gerta Rücker, Guido Schwarzer. Ranking treatments in frequentist network meta-analysis works without resampling methods. BMC Med Res Methodol. 2015 Jul 31;15:58. doi: 10.1186/s12874-015-0060-8.

Supplementary Table 2. Study characteristics.

Study*	Treatment (dosing schedule, plasma concentration)**	n	Diagnosis	Structured Interview	Severity threshold for mania	Male (%)	Mean age (years)	Patients with rapid cycling (%)	Patients with mixed episode (%)	Patients with psychotic features (%)	Observational point of main outcomes (week)***	Duration (week)	Country	The definition of response to treatment
Ahmad 2016 (END4)	END 4 mg/d (fixed)	27	DSM-IV-TR	ni	YMRS ≥ 20 + CGI-S ≥ 4	ni	36.8	ni	ni	ni	3	3	India	≥ 50% improvement for YMRS
	VALP 1000 mg/d (fixed, ni)	15												
Ahmad 2016 (END8)	END 8 mg/d (fixed)	28	DSM-IV-TR	ni	YMRS ≥ 20 + CGI-S ≥ 4	ni	36.8	ni	ni	ni	3	3	India	≥ 50% improvement for YMRS
	VALP 1000 mg/d (fixed, ni)	14												
Berk 1999	LAM 100 mg/d (fixed)	15	DSM-IV	MINI	ni	55.56	31.63	ni	ni	ni	4	4	South Africa	≥ 50% improvement for MRS or BPRS or CGI-S = 1 or 2
	LIT 800 mg/d (fixed, mean 0.74 mmol/L)	15												
	OLA 10 mg/d (fixed)	15												
Berwaerts 2012	PAL 3, 6, 12 mg/d (fixed)	347	DSM-IV	MINI	YMRS ≥ 20	53.50	39.52	0.00	36.79	22.80	3	3	International	≥ 50% improvement for YMRS

	PLA	122												
Bowden 1994	LIT ≥ 1200 mg/d (flexible, ≤ 1.5 mmol/L)	36	RDC	RDC	MRS ≥ 14	58.09	39.56	ni	ni	ni	3	3	USA	≥ 50% improvement for MRS
	VALP ≥ 1000 mg/d (flexible, ≤ 150 µg/mL)	69												
	PLA	74												
Bowden 2000	LAM 25-200 mg/d (flexible)	74	DSM-IV	ni	MRS ≥ 18	52.84	37.54	ni	ni	ni	6	6	International	≥ 50% improvement for MRS
	LIT (flexible, 0.7-1.3 mEq/L)	77												
	PLA	77												
Bowden 2005	LIT initial 900 mg/d (flexible, 0.6-1.4 mEq/L)	98	DSM-IV	ni	YMRS ≥ 20 + CGI-BP ≥ 4	57.67	39.31	0.00	0.00	27.67	3	12	International	≥ 50% improvement for YMRS
	QUE 600-800 mg/d (flexible)	107												
	PLA	97												
Bowden 2006	VALP mean 3057 mg/d (flexible, 85-125 µg/mL)	187	DSM-IV-TR	SCID	MRS ≥ 18	57.42	37.53	5.97	43.68	20.60	3	3	USA	≥ 50% improvement for MRS
	PLA	177												

Calabrese 2015	CARI 3-6, 6-12 mg/d (flexible)	336	DSM-IV-TR	ni	YMRS ≥ 20 + MADRS < 18	53.12	41.94	0.00	ni	ni	3	3	International	≥ 50% improvement for YMRS
	PLA	161												
Cutler 2011	QUE 400-800 mg/d (flexible)	151	DSM-IV-TR	SCID	YMRS ≥ 20 + CGI-S ≥ 4	60.06	41.04	31.49	43.51	ni	3	3	USA	≥ 50% improvement for YMRS
	PLA	160												
Durgam 2015	CARI 3-12 mg/d (flexible)	118	DSM-IV-TR	SCID	YMRS ≥ 20	66.53	38.35	0.00	19.07	ni	3	3	International	≥ 50% improvement for YMRS
	PLA	120												
El Mallakh 2010	ARI 15, 30 mg/d (fixed)	267	DSM-IV	ni	YMRS ≥ 20	48.31	40.45	20.20	39.40	ni	3	3	International	≥ 50% improvement for YMRS
	PLA	134												
Freeman 1992	LIT (flexible, 0.8-1.4 rnmol/L)	13	DSM-III-R	Semi	ni	22.22	ni	ni	ni	ni	3	3	USA	≥ 50% improvement for MRS
	VALP (flexible, 65-126 µg/mL)	14												
Garfinkel 1980	HAL initial 30 mg/d (flexible)	7	Criteria of Feighner for mania	ni	ni	42.86	39.25	ni	ni	ni	3	3	Canada	

	LIT initial dose 900 mg/d (flexible, 1.2 mEq/L)	7												
Goldsmith 2003****	LAM 50 mg/d (fixed)	84	DSM-IV	ni	MRS ≥ 18	52.78	37.91	ni	ni	ni	3	3	International	≥ 50% improvement for MRS
	LIT (flexible, 0.8-1.3 mEq/L)	36												
	PLA	95												
Grunze 2015 (203)	ESL 600-1800, 800-2400 mg/d (flexible)	121	DSM-IV	ni	YMRS ≥ 20	49.69	42.47	0.00	ni	0.00	3	3	Europe	≥ 50% improvement for YMRS or YMRS < 12
	PLA	40												
Grunze 2015 (204)	ESL 600, 1200, 1800 mg/d (fixed)	27	DSM-IV	ni	YMRS ≥ 20	42.11	41.93	0.00	ni	0.00	3	3	International	≥ 50% improvement for YMRS or YMRS < 12
	PLA	11												
Hirschfeld 1999	LIT initial dose 900 mg/d (flexible, ni)	19	DSM-IV	ni	YMRS ≥ 14	57.63	34.91	ni	ni	ni	10 d	10 d	ni	
	VALP loading group (fixed, mean 83.8 µg/mL), non-loading group (flexible, mean 41.8 µg/mL)	40												

Hirschfeld 2004	RIS 1-6 mg/d (flexible)	134	DSM-IV	SCID	YMRS ≥ 20 + MADRS ≤ 20	56.76	38.78	ni	0.00	42.47	3	3	USA	≥ 50% improvement for YMRS
	PLA	125												
Hirschfeld 2010	VALP mean 2210.5 mg/d (flexible, mean 83.24 µg/mL)	147	DSM-IV	SCID	MRS ≥ 25	51.35	39.17	ni	31.50	40.10	3	3	USA	
	PLA	78												
Janicak 1998	VER mean 480 mg/d (flexible)	17	DSM-III-R	ni	ni	59.38	36.2	ni	6.30	71.88	3	3	USA	≥ 40% improvement for YMRS
	PLA	15												
Kakkar 2009	OXC 1000-2400 mg/d (flexible, ni)	30	DSM-IV	ni	YMRS ≥ 20	55	29.39	0.00	0.00	ni	12	12	India	≥ 50% improvement for YMRS
	VALP 750-2000 mg/d (flexible, ni)	30												
Kanba 2014	ARI 12-24 mg/d (flexible)	128	DSM-IV-TR	MINI	YMRS ≥ 20	41.30	37.65	9.31	11.34	36.03	3	3	Asia	≥ 50% improvement for YMRS
	PLA	130												
Katagiri 2012	HAL 2.5-10 mg/d (flexible)	20	DSM-IV-TR	MINI	YMRS ≥ 20	45.25	43.40	7.24	8.14	18.10	3	3	Japan	≥ 50% improvement for YMRS
	OLA 5-20 mg/d (flexible)	105												

	PLA	99												
Keck 2003 ARI	ARI 15-30 mg/d (flexible)	130	DSM-IV	ni	YMRS ≥ 20	43.51	40.50	23.28	32.82	25.7	3	3	USA	≥ 50% improvement for YMRS
	PLA	132												
Keck 2003 ZIP	ZIP 80-160 mg/d (flexible)	140	DSM-IV	SCID	MRS ≥ 14	54.31	38.33	ni	35.53	45.7	3	3	USA and Brazil	≥ 50% improvement for MRS
	PLA	70												
Keck 2009	ARI 15-30 mg/d (flexible)	155	DSM-IV-TR	MINI	YMRS ≥ 20 + MADRS ≤ 17	51.88	39.67	0.00	39.17	23.13	3	3	USA	≥ 50% improvement for YMRS
	LIT 900-1500 mg/d (flexible, mean 0.76 mEq/L)	160												
	PLA	165												
Khanna 2005	RIS 1-6 mg/d (flexible)	146	DSM-IV	ni	YMRS ≥ 20	62.04	35.10	0.00	4.49	59.01	3	3	India	≥ 50% improvement for YMRS
	PLA	145												
Kushner 2006 (004)	LIT 900-1800 mg/d (flexible, 0.8–1.2 mEq/L)	113	DSM-IV	SCID	YMRS ≥ 20	47.39	42.50	0.00	15.30	29.39	3	3	International	≥ 50% improvement for YMRS
	TOP 200, 400 mg/d (fixed)	220												

	PLA	111												
Kushner 2006 (005)	TOP 400, 600 mg/d (fixed)	214	DSM-IV	SCID	YMRS $\geq$ 20	46.60	38.71	0.00	53.62	27.32	3	3	USA	$\geq$ 50% improvement for YMRS
	PLA	100												
Kushner 2006 (006)	TOP 400 mg/d (fixed)	109	DSM-IV	SCID	YMRS $\geq$ 20	63.50	40.50	0.00	34.01	33.50	3	3	USA	$\geq$ 50% improvement for YMRS
	PLA	106												
Kushner 2006 (008)	LIT 900-1800 mg/d (flexible, 0.8–1.2 mEq/L)	114	DSM-IV	SCID	YMRS $\geq$ 20	37.31	41.00	0.00	11.00	22.01	3	3	International	$\geq$ 50% improvement for YMRS
	TOP 400 mg/d	116												
	PLA	112												
Landbloom 2016	ASE 10, 20 mg/d (fixed)	241	DSM-IV-TR	MINI	YMRS $\geq$ 20	45.23	43.82	0.00	29.43	ni	3	3	International	$\geq$ 50% improvement for YMRS
	PLA	126												
Lerer 1987	CARB mean 1400 mg/d (flexible, 8-12 μg/L)	15	DSM-III	ni	ni	46.43	40.50	ni	ni	ni	4	4	USA	
	LIT (flexible, mean 0.87 mmol/L)	19												

Li 2008	LIT 250-2000 mg/d (flexible, mean 0.85 mEq/L)	77	CCMD-3	ni	YMRS $\geq$ 20	47.40	33.15	0.00	0.00	27.92	4	4	China	$\geq$ 50% improvement for YMRS
	QUE 200-800 mg/d (flexible)	77												
McIntyre 2005	HAL 2-8 mg/d (flexible)	99	DSM-IV	ni	YMRS $\geq$ 20 + CGI-BP $\geq$ 4	36.79	42.82	0.00	0.00	41.81	3	12	International	$\geq$ 50% improvement for YMRS
	QUE 400-800 mg/d (flexible)	102												
	PLA	101												
McIntyre 2009	ASE 10-20 mg/d (flexible)	194	DSM-IV	MINI	YMRS $\geq$ 20	57.38	39.39	0.00	30.74	ni	3	3	International	$\geq$ 50% improvement for YMRS
	OLA 5-20 mg/d (flexible)	190												
	PLA	104												
McIntyre 2010	ASE 10-20 mg/d (flexible)	185	DSM-IV	MINI	YMRS $\geq$ 20	52.66	38.61	0.00	31.15	ni	3	3	International	$\geq$ 50% improvement for YMRS
	OLA 5-20 mg/d (flexible)	205												
	PLA	98												
Niufan 2008	LIT 600-1800 mg/d (flexible, 0.81-1.20 mEq/L)	71	DSM-IV	ni	YMRS $\geq$ 20	47.14	32.62	0.71	0.00	13.57	4	4	China	$\geq$ 50% improvement for YMRS

	OLA 5-20 mg/d (flexible)	69												
Perlis 2006	OLA 5-20 mg/d (flexible)	165	DSM-IV	ni	YMRS ≥ 20	45.29	37.90	45.31	58.66	0.00	3	3	USA	≥ 50% improvement for YMRS
	RIS 1-6 mg/d (flexible)	164												
Pope 1991	VALP ≥ 750 mg/d (flexible, 50-100 mg/L)	20	DSM-III-R	SCID	ni	72.22	37.00	ni	ni	ni	3	3	USA	≥ 50% improvement for YMRS
	PLA	23												
Potkin 2005	ZIP 80-160 mg/d (flexible)	140	DSM-IV	SCID	MRS ≥ 14	50.73	38.93	ni	40.49	31.20	3	3	International	≥ 50% improvement for MRS
	PLA	66												
Rezayat 2014	ARI 5-30 mg/d (flexible)	31	DSM-IV-TR	ni	YMRS ≥ 20	50.00	34.00	ni	ni	ni	6	6	Iran	
	RIS 2-8 mg/d (flexible)	31												
Sachs 2006	ARI 15-30 mg/d (flexible)	137	DSM-IV	ni	YMRS ≥ 20	48.53	38.84	17.65	41.54	13.1	3	3	USA	≥ 50% improvement for YMRS
	PLA	135												
Sachs 2015	CARI 3-12 mg/d (flexible)	158	DSM-IV-TR	SCID	YMRS ≥ 20 + MADRS < 18	64.10	36.24	0.00	9.94	33.01	3	3	USA and India	≥ 50% improvement for YMRS

	PLA	154												
Segal 1998	HAL 10 mg/d (fixed)	15	DSM-IV	Yes	ni	22.22	33.63	ni	ni	ni	4	4	South Africa	
	LIT 800-1200 mg/d (flexible, 0.6-1.2 mmol/L)	15												
	RIS 6 mg/d (fixed)	15												
Shafti 2010	LIT mean 1156 mg/d (flexible, mean 0.781 mEq/L)	20	DSM-IV-TR	ni	ni	0.00	ni	ni	0.00	ni	3	3	Iran	
	OLA mean 20.52 mg/d (flexible)	20												
Shafti 2018 ARI	ARI mean 25.83 mg/d (flexible)	15	DSM	No	ni	100.00	30.25	ni	0.00	50.00	4	4	Iran	
	LIT mean 981.81 mg/d (flexible, mean 0.80 mEq/L)	15												
Shafti 2018 CARB	CARB mean 912.5 mg/d (flexible, ni)	25	DSM-5	ni	ni	100.00	30.96	ni	0.00	ni	3	3	Iran	
	LIT mean 965.78 mg/d (flexible, 0.74 mEq/L)	25												
Shopsin 1975	CHL 300-1800 mg/d (flexible)	10	DSM-2	ni	ni	ni	ni	ni	ni	ni	3	3	USA	
	HAL 6-36 mg/d (flexible)	10												

	LIT 750-4500 mg/d (flexible, 1.0-2.0 mEq/L)	10												
Small 1991	CARB 1052 mg/d (flexible, 25-50 µmol/L)	27	RDC and DSM-III-R	ni	SDMD-D&M ≥ 7	43.75	38.45	ni	ni	ni	8	8	USA	≥ 50% improvement for YMRS
	LIT (flexible, 0.6-1.5 mmol/L)	25												
Smulevich 2005	HAL 2-12 mg/d (flexible)	144	DSM-IV	ni	YMRS ≥ 20 + MADRS ≤ 20	52.97	39.77	0.00	0.00	33.11	3	3	International	≥ 50% improvement for YMRS
	RIS 1-6 mg/d (flexible)	154												
	PLA	140												
Tohen 1999	OLA 5-20 mg/d (flexible)	70	DSM-IV	SCID	YMRS ≥ 20	51.8	39.5	32.4	17.3	53.2	3	3	USA	≥ 50% improvement for YMRS
	PLA	69												
Tohen 2000	OLA 5-20 mg/d (flexible)	55	DSM-IV	SCID	YMRS ≥ 20	49.57	38.67	39.13	42.61	55.65	4	4	International	≥ 50% improvement for YMRS
	PLA	60												
Tohen 2002	OLA 5-20 mg/d (flexible)	125	DSM-IV	SCID	YMRS ≥ 20	42.63	40.55	57.37	43.03	45.42	3	3	USA	≥ 50% improvement for YMRS

	VALP 500-2500 mg/d (flexible, 50-125 µg/mL)	126												
Tohen 2003	HAL 3-15 mg/d (flexible)	219	DSM-IV	SCID	YMRS ≥ 20	39.74	40.52	ni	ni	57.40	6	6	International	≥ 50% improvement for YMRS
	OLA 5-20 mg/d (flexible)	234												
Tohen 2008	OLA 5-20 mg/d (flexible)	215	DSM-IV-TR	SCID	YMRS ≥ 20 + CGI-BP-mania 3 or 4	44.53	39.61	0.00	27.06	0.00	3	3	International	≥ 50% improvement for YMRS
	VALP 500-2500 mg/d (flexible, 50-125 µg/mL)	201												
	PLA	105												
Vasudev 2000*****	CAR 800-1200 mg/d (flexible, < 14 µg/ml)	15	DSM-III-R	SCID	YMRS ≥ 20	20.00	ni	ni	ni	66.67	4	4	India	
	VALP 800-1400 mg/d (flexible, < 125 µg/ml)	15												
Vieta 2005	ARI 15-30 mg/d (flexible)	175	DSM-IV	ni	YMRS ≥ 20	38.33	41.81	0.00	10.95	ni	3	12	International	≥ 50% improvement for YMRS
	HAL 10-15 mg/d (flexible)	172												

Vieta 2010 ZIP	HAL 8-30 mg/d (flexible)	172	DSM-IV	ni	MRS ≥ 14	58.81	38.33	ni	ni	ni	3	3	International	≥ 50% improvement for MRS
	ZIP 80-160 mg/d (flexible)	178												
	PLA	88												
Vieta 2010 PAL	PAL 3-12 mg/d (flexible)	195	DSM-IV	ni	YMRS ≥ 20	57.61	39.18	0.00	35.19	20.99	3	3	International	≥ 50% improvement for YMRS
	QUE 400-800 mg/d (flexible)	193												
	PLA	105												
Weiser 2017	RIS 6 mg/d (fixed)	32	DSM-IV-TR	SCID	YMRS ≥ 20	33.53	45.95	ni	0.00	ni	3	3	Romania	
	VALN 1500 mg/d (fixed)	71												
	PLA	70												
Weisler 2004	CARB 200-1600 mg/d (flexible, mean 8.9 µg/mL)	101	DSM-IV	ni	YMRS ≥ 20	52.45	38.05	ni	52.94	ni	3	3	ni	≥ 50% improvement for YMRS
	PLA	103												
Weisler 2005	CARB 200-1600 mg/d (flexible, ni)	122	DSM-IV	ni	YMRS ≥ 20	70.29	36.96	ni	20.92	ni	3	3	USA and India	≥ 50% improvement for YMRS
	PLA	117												

Xu 2015	OLA 5-20 mg/d (flexible)	40	DSM-IV	ni	YMRS ≥ 17	48.06	30.55	ni	24.68	ni	4	4	China	
	VALP 1200-1800 mg/d (flexible, ni)	40												
Yildiz 2008	TAM 80 mg/d (flexible)	35	DSM-IV	SCID	YMRS > 20	48.48	34.8	ni	3.03	66.67	3	3	Turkey	≥ 50% improvement for YMRS
	PLA	31												
Young 2009	ARI 15-30 mg/d (flexible)	167	DSM-IV-TR	MINI	YMRS ≥ 20 + MADRS ≤ 17	44.33	40.78	0.00	18.76	9.69	3	3	International	≥ 50% improvement for YMRS
	HAL 5-15 mg/d (flexible)	165												
	PLA	153												
Zajecka 2002	OLA 5-20 mg/d (flexible)	57	DSM-IV	SCID	MRS ≥ 25	54.17	38.52	29.17	47.50	33.33	3	12	International	
	VALP 750-3250 mg/d (flexible, 101.2 µg/mL)	63												
Zarate 2007	TAM 20-140 mg/d (flexible)	8	DSM-IV-TR	SCID	YMRS ≥ 14	87.50	35.35	37.50	31.25	50.00	3	3	USA	≥ 50% improvement for YMRS
	PLA	8												
NCT03259555	BRE 2-4 mg/d (flexible)	158	DSM-5	MINI	YMRS ≥ 24	49.38	43.96	0.00	ni	ni	3	3	International	

	PLA	164											
NCT03257865	BRE 2-4 mg/d (flexible)	163	DSM-5	MINI	YMRS $\geq$ 24	49.85	44.45	0.00	ni	ni	3	3	International	
	PLA	170											
NCT00099229	LIC 1000-2000 mg/d (flexible, ni)	324	ni	ni	ni	53.4	40.0	ni	36.7	36.7	3	3	International	$\geq$ 50% improvement for YMRS
	PLA	163											

ARI: aripiprazole, ASE: asenapine, BRE: brexpiprazole, CARB: carbamazepine, CARI: cariprazine, CGI-(BP)-S: Clinical Global Impressions(-Bipolar. Version) - severity of illness, CHL: chlorpromazine, DSM(-TR); Diagnostic and Statistical Manual of Mental Disorders(-Text Revision), END: endoxifen, ESL: eslicarbazepine, HAL: haloperidol, LAM: lamotrigine, LIC: licarbazepine, LIT: lithium, MADRS: Montgomery Åsberg Depression Rating Scale, MINI: Mini International Neuropsychiatric Interview, MRS: Mania Rating Scale (Schedule for Affective Disorders and Schizophrenia), ni. no information, OLA: olanzapine, OXC: oxcarbazepine, PAL: paliperidone, PLA: placebo, QUE: quetiapine, RIS: risperidone, SCID: Structured Clinical Interview for DSM, SDMD-D&M: Manic subsection of the Depression & Mania Scale, TAM: tamoxifen, TOP: topiramate, VALN: valnoctamide, VALP: valproate, VER: verapamil, YMRS: Young Mania Rating Scale, ZIP: ziprasidone

When the original article did not report the information, we extracted the information from previous review articles.

*The underlined studies were included Yildiz's previous network meta-analysis study (2015)

We did not include the following two studies that included Yildiz's (2015) previous network meta-analysis

McElroy SL, Keck PE, Stanton SP, Tugrul KC, Bennett JA, Strakowski SM. A randomized comparison of divalproex oral loading versus haloperidol in the initial treatment of acute psychotic mania. J Clin Psychiatry 1996; 57(4): 142-146.

→The study was a single-blind study. No available data for objective outcomes were reported.

Jochim J, Rifkin-Zybutz RP, Geddes J, Cipriani A. Valproate for acute mania. Cochrane Database Syst Rev. 2019 Oct 7;10(10):CD004052

**We aimed to examine antimanic drugs' efficacy for acute treatment, so we targeted outcome assessments at 3 or 4 weeks. If 3-or 4-weeks data were not available, we showed data at the points closest to 3 weeks.

***Main outcomes: response to treatment, all-cause discontinuation, and improvement of mania rating scales scores.

****We defined this study as a very-low-dose study, followed by Yildiz's (2015) previous network meta-analysis study.

*****The study did not allow the antipsychotics as a rescue medication during a trial. However, because there were patients who received antipsychotics during the study, we did not include the data of this study in the meta-analysis for efficacy outcomes. However, because those patients discontinued the trial, we included the data of this study in the meta-analysis for all-cause discontinuation.

Supplementary Appendix 1. Response to treatment (N = 56, n = 14503)

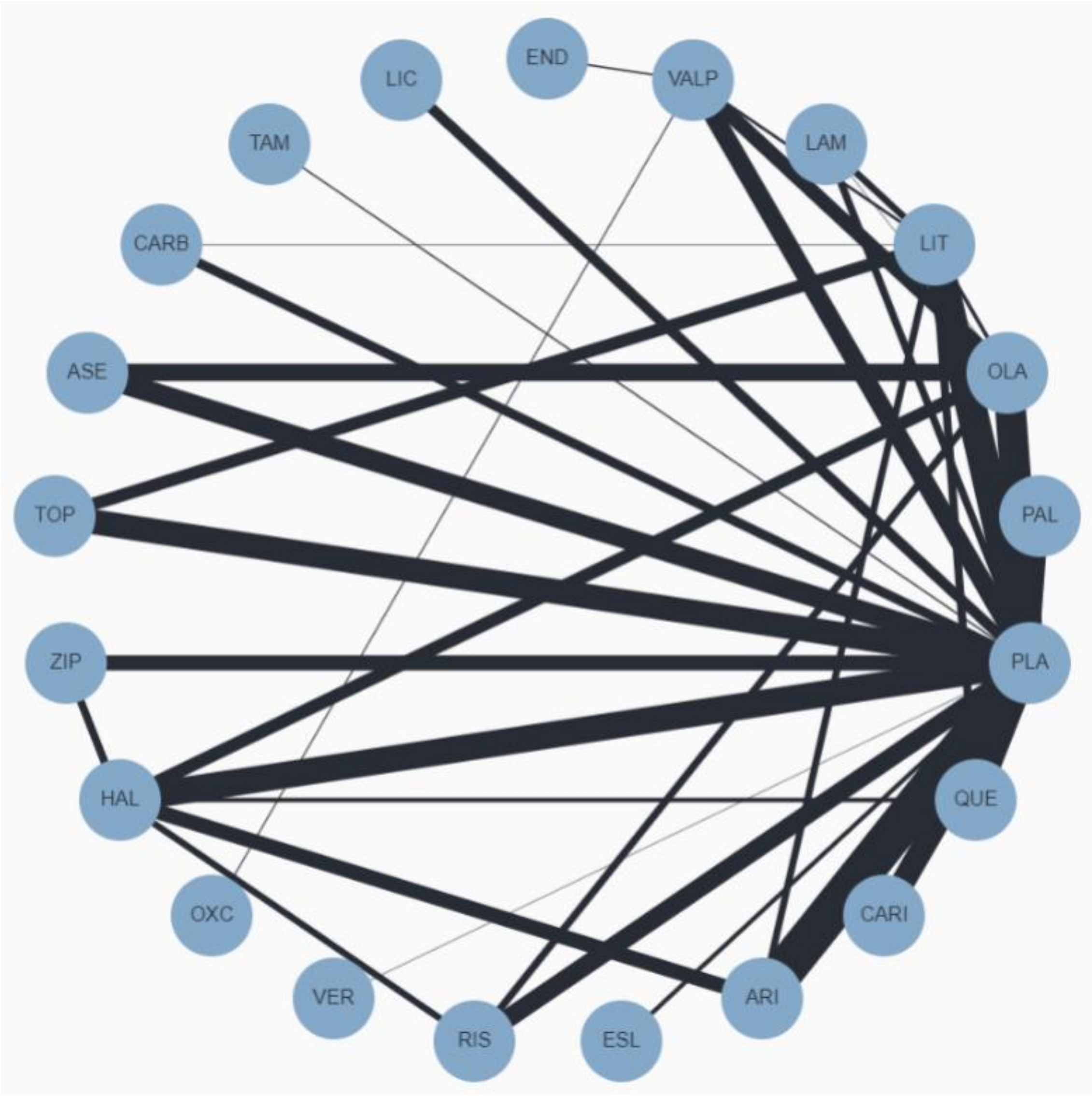

We described the reason why we selected "response to treatment" for the primary outcome for efficacy. It is sometimes difficult for clinicians to interpret the results of efficacy measured using rating scales. The clinical meaning of the results is especially unclear when different measures are used in different studies, and a standardized mean difference is employed as the effect size. We considered that a pragmatic outcome, such as the response to treatment, would make the results easier to interpret.

League table (risk ratio with 95% confidence interval)

ARI	1.194 (0.938, 1.519)	0.804 (0.577, 1.119)	0.981 (0.761, 1.265)	1.413 (0.909, 2.197)	**1.510 (1.099, 2.073)**	0.931 (0.787, 1.102)	1.214 (0.919, 1.603)	**1.501 (1.014, 2.222)**	1.053 (0.881, 1.260)	0.963 (0.805, 1.151)	1.208 (0.807, 1.809)	1.097 (0.837, 1.440)	0.985 (0.798, 1.216)	0.906 (0.725, 1.131)	**0.205 (0.051, 0.821)**	**1.673 (1.296, 2.161)**	1.074 (0.858, 1.345)	1.155 (0.216, 6.168)	1.132 (0.861, 1.487)	**1.529 (1.327, 1.762)**
	ASE	**0.673 (0.470, 0.964)**	0.822 (0.615, 1.099)	1.184 (0.749, 1.871)	1.264 (0.894, 1.789)	**0.780 (0.619, 0.983)**	1.017 (0.745, 1.387)	1.257 (0.829, 1.907)	0.882 (0.702, 1.109)	**0.806 (0.662, 0.983)**	1.012 (0.664, 1.543)	0.919 (0.677, 1.248)	0.825 (0.640, 1.064)	**0.758 (0.586, 0.981)**	**0.172 (0.043, 0.693)**	**1.401 (1.046, 1.877)**	0.899 (0.696, 1.162)	0.968 (0.180, 5.196)	0.948 (0.695, 1.293)	**1.281 (1.049, 1.563)**
		CARB	1.221 (0.846, 1.762)	**1.758 (1.050, 2.944)**	**1.878 (1.243, 2.838)**	1.158 (0.834, 1.609)	**1.510 (1.028, 2.218)**	**1.867 (1.163, 2.997)**	1.311 (0.951, 1.806)	1.198 (0.867, 1.655)	1.503 (0.926, 2.438)	1.365 (0.934, 1.996)	1.226 (0.872, 1.722)	1.127 (0.795, 1.597)	0.255 (0.062, 1.047)	**2.082 (1.440, 3.008)**	1.336 (0.943, 1.894)	1.437 (0.264, 7.833)	1.408 (0.958, 2.069)	**1.902 (1.409, 2.567)**
			CARI	1.440 (0.899, 2.306)	**1.538 (1.080, 2.191)**	0.949 (0.738, 1.220)	1.237 (0.896, 1.709)	**1.529 (1.002, 2.334)**	1.074 (0.838, 1.375)	0.981 (0.768, 1.253)	1.231 (0.796, 1.904)	1.118 (0.816, 1.533)	1.004 (0.768, 1.312)	0.923 (0.699, 1.218)	**0.209 (0.052, 0.844)**	**1.705 (1.260, 2.308)**	1.094 (0.829, 1.445)	1.177 (0.219, 6.331)	1.153 (0.836, 1.590)	**1.558 (1.262, 1.924)**
				END	1.068 (0.643, 1.775)	0.659 (0.426, 1.020)	0.859 (0.531, 1.389)	1.062 (0.608, 1.855)	0.745 (0.485, 1.145)	0.681 (0.447, 1.040)	0.855 (0.515, 1.419)	0.777 (0.481, 1.255)	0.697 (0.446, 1.090)	0.641 (0.408, 1.007)	**0.145 (0.034, 0.614)**	1.184 (0.740, 1.893)	0.760 (0.520, 1.111)	0.818 (0.146, 4.571)	0.801 (0.494, 1.299)	1.082 (0.710, 1.648)
					ESL	**0.617 (0.450, 0.845)**	0.804 (0.553, 1.170)	0.994 (0.626, 1.580)	**0.698 (0.511, 0.953)**	**0.638 (0.468, 0.869)**	0.800 (0.498, 1.287)	0.727 (0.503, 1.050)	**0.653 (0.470, 0.906)**	**0.600 (0.429, 0.839)**	**0.136 (0.033, 0.556)**	1.108 (0.775, 1.585)	**0.711 (0.508, 0.996)**	0.765 (0.141, 4.160)	0.750 (0.516, 1.089)	1.013 (0.763, 1.345)
						HAL	1.304 (0.990, 1.717)	**1.612 (1.090, 2.383)**	1.131 (0.948, 1.350)	1.034 (0.885, 1.209)	1.298 (0.871, 1.934)	1.179 (0.903, 1.539)	1.058 (0.867, 1.292)	0.973 (0.794, 1.192)	**0.220 (0.055, 0.881)**	**1.797 (1.395, 2.315)**	1.153 (0.929, 1.432)	1.241 (0.233, 6.621)	1.215 (0.947, 1.560)	**1.642 (1.432, 1.883)**
							LAM	1.236 (0.796, 1.920)	0.868 (0.680, 1.107)	0.793 (0.609, 1.032)	0.995 (0.637, 1.555)	0.904 (0.647, 1.263)	0.812 (0.611, 1.078)	0.746 (0.553, 1.007)	**0.169 (0.042, 0.686)**	**1.378 (1.004, 1.892)**	0.885 (0.659, 1.188)	0.952 (0.176, 5.141)	0.932 (0.662, 1.313)	1.259 (0.986, 1.608)

								LIC	0.702 (0.476, 1.035)	**0.642 (0.436, 0.944)**	0.805 (0.474, 1.366)	0.731 (0.473, 1.129)	**0.656 (0.439, 0.981)**	**0.603 (0.401, 0.907)**	**0.137 (0.033, 0.570)**	1.115 (0.728, 1.706)	0.716 (0.476, 1.077)	0.770 (0.139, 4.250)	0.754 (0.486, 1.169)	1.019 (0.706, 1.469)
									LIT	0.914 (0.782, 1.068)	1.147 (0.776, 1.694)	1.042 (0.805, 1.349)	0.935 (0.784, 1.116)	0.860 (0.694, 1.065)	**0.195 (0.049, 0.778)**	**1.588 (1.267, 1.991)**	1.019 (0.835, 1.244)	1.097 (0.206, 5.849)	1.074 (0.819, 1.409)	**1.451 (1.275, 1.652)**
										OLA	1.255 (0.855, 1.840)	1.140 (0.877, 1.481)	1.023 (0.841, 1.245)	0.940 (0.777, 1.138)	**0.213 (0.053, 0.851)**	**1.738 (1.362, 2.217)**	1.115 (0.927, 1.342)	1.200 (0.225, 6.396)	1.175 (0.902, 1.531)	**1.588 (1.403, 1.797)**
											OXC	0.908 (0.582, 1.418)	0.816 (0.541, 1.229)	0.750 (0.495, 1.135)	**0.170 (0.040, 0.710)**	1.385 (0.897, 2.138)	0.889 (0.636, 1.243)	0.956 (0.173, 5.297)	0.937 (0.598, 1.468)	1.266 (0.865, 1.853)
												PAL	0.898 (0.702, 1.148)	0.825 (0.615, 1.107)	**0.187 (0.046, 0.758)**	**1.525 (1.111, 2.092)**	0.979 (0.730, 1.312)	1.053 (0.195, 5.678)	1.031 (0.737, 1.442)	**1.393 (1.102, 1.761)**
													QUE	0.919 (0.724, 1.167)	**0.208 (0.052, 0.835)**	**1.698 (1.305, 2.210)**	1.090 (0.861, 1.380)	1.173 (0.219, 6.273)	1.149 (0.861, 1.533)	**1.552 (1.316, 1.830)**
														RIS	**0.226 (0.056, 0.911)**	**1.848 (1.397, 2.444)**	1.186 (0.928, 1.515)	1.276 (0.238, 6.836)	1.250 (0.930, 1.679)	**1.689 (1.411, 2.021)**
															TAM	**8.165 (2.018, 33.033)**	**5.240 (1.302, 21.092)**	5.637 (0.646, 49.176)	**5.522 (1.359, 22.430)**	**7.461 (1.876, 29.678)**

																TOP	**0.642 (0.487, 0.846)**	0.690 (0.128, 3.716)	**0.676 (0.489, 0.935)**	0.914 (0.736, 1.135)
																	VALP	1.076 (0.201, 5.765)	1.054 (0.781, 1.421)	**1.424 (1.188, 1.707)**
																		VER	0.980 (0.181, 5.289)	1.324 (0.249, 7.023)
																			ZIP	**1.351 (1.061, 1.721)**
																				PLA

The boldface result indicates statistical significance.

Study	The definition of response to treatment
Ahmad 2016 (END4)	≥ 50% improvement for YMRS
Ahmad 2016 (END8)	≥ 50% improvement for YMRS
Berk 1999	≥ 50% improvement for MRS or BPRS or CGI-S = 1 or 2
Berwaerts 2012	≥ 50% improvement for YMRS
Bowden 1994	≥ 50% improvement for MRS
Bowden 2000	≥ 50% improvement for MRS
Bowden 2005	≥ 50% improvement for YMRS
Bowden 2006	≥ 50% improvement for MRS

Calabrese 2015	≥ 50% improvement for YMRS
Cutler 2011	≥ 50% improvement for YMRS
Durgam 2015	≥ 50% improvement for YMRS
El Mallakh 2010	≥ 50% improvement for YMRS
Freeman 1992	≥ 50% improvement for MRS
Garfinkel 1980	
Goldsmith 2003	≥ 50% improvement for MRS
Grunze 2015 (203)	≥ 50% improvement for YMRS or YMRS < 12
Grunze 2015 (204)	≥ 50% improvement for YMRS or YMRS < 12
Hirschfeld 1999	
Hirschfeld 2004	≥ 50% improvement for YMRS
Hirschfeld 2010	
Janicak 1998	≥ 40% improvement for YMRS
Kakkar 2009	≥ 50% improvement for YMRS
Kanba 2014	≥ 50% improvement for YMRS
Katagiri 2012	≥ 50% improvement for YMRS
Keck 2003 ARI	≥ 50% improvement for YMRS
Keck 2003 ZIP	≥ 50% improvement for MRS
Keck 2009	≥ 50% improvement for YMRS
Khanna 2005	≥ 50% improvement for YMRS
Kushner 2006 (004)	≥ 50% improvement for YMRS
Kushner 2006 (005)	≥ 50% improvement for YMRS
Kushner 2006 (006)	≥ 50% improvement for YMRS
Kushner 2006 (008)	≥ 50% improvement for YMRS
Landbloom 2016	≥ 50% improvement for YMRS

Lerer 1987	
Li 2008	≥ 50% improvement for YMRS
McIntyre 2005	≥ 50% improvement for YMRS
McIntyre 2009	≥ 50% improvement for YMRS
McIntyre 2010	≥ 50% improvement for YMRS
Niufan 2008	≥ 50% improvement for YMRS
Perlis 2006	≥ 50% improvement for YMRS
Pope 1991	≥ 50% improvement for YMRS
Potkin 2005	≥ 50% improvement for MRS
Rezayat 2014	
Sachs 2006	≥ 50% improvement for YMRS
Sachs 2015	≥ 50% improvement for YMRS
Segal 1998	
Shafti 2010	
Shafti 2018 ARI	
Shafti 2018 CARB	
Shopsin 1975	
Small 1991	≥ 50% improvement for YMRS
Smulevich 2005	≥ 50% improvement for YMRS
Tohen 1999	≥ 50% improvement for YMRS
Tohen 2000	≥ 50% improvement for YMRS
Tohen 2002	≥ 50% improvement for YMRS
Tohen 2003	≥ 50% improvement for YMRS
Tohen 2008	≥ 50% improvement for YMRS
Vasudev 2000	

Vieta 2005	≥ 50% improvement for YMRS
Vieta 2010 ZIP	≥ 50% improvement for MRS
Vieta 2010 PAL	≥ 50% improvement for YMRS
Weiser 2017	
Weisler 2004	≥ 50% improvement for YMRS
Weisler 2005	≥ 50% improvement for YMRS
Xu 2015	
Yildiz 2008	≥ 50% improvement for YMRS
Young 2009	≥ 50% improvement for YMRS
Zajecka 2002	
Zarate 2007	≥ 50% improvement for YMRS
NCT03259555	
NCT03257865	
NCT00099229	≥ 50% improvement for YMRS

BPRS: Brief Psychiatric Rating Scale, CGI-S: Clinical Global Impressions - severity of illness, MRS: Mania Rating Scale (Schedule for Affective Disorders and Schizophrenia), YMRS: Young Mania Rating Scale

Evaluation of heterogeneity and inconsistency

We inferred the magnitude of heterogeneity by comparing the estimated τ^2 to empirical distributions of heterogeneity typically found in meta-analyses. The predictive τ^2 distribution for mental health outcomes according to Rhodes et al. has a median of 0.049 and an IQR of 0.01– 0.242. Low heterogeneity could be considered when the estimated τ^2 is less than the 25% quantile of the empirical distribution, moderate heterogeneity for τ^2 between 25% and 50% quantile and high heterogeneity for τ^2 larger than the 50% quantile. We evaluated global consistency under the assumption of a full design-by-treatment interaction model in netmeta.

Rhodes KM, Turner RM, Higgins JPT. Empirical evidence about inconsistency among studies in a pairwise meta-analysis. Research synthesis methods 2016; 7: 346–70.

Between study variance (τ^2)	Heterogeneity assessment	Random-effects design-by-treatment interaction model		
		Q	df	p
0.017	Low	46.528	33	0.059

Incoherence

	NMA, RR (95% CI)	Direct, RR (95% CI)	I^2	Indirect, RR (95% CI)	Inconsistency measures	
					Difference of RR	P value
ARI vs HAL	0.931 (0.787, 1.102)	1.063 (0.834, 1.355)	52.4%	0.823 (0.652, 1.040)	1.291 (0.922, 1.808)	0.137
ARI vs LIT	1.053 (0.881, 1.260)	1.022 (0.719, 1.451)	na	1.065 (0.865, 1.312)	0.959 (0.638, 1.443)	0.842
ARI vs PLA	1.529 (1.327, 1.762)	1.420 (1.210, 1.665)	40.4%	2.012 (1.480, 2.736)	0.705 (0.499, 0.997)	0.048
ASE vs OLA	0.806 (0.662, 0.983)	0.812 (0.641, 1.029)	0.0%	0.794 (0.554, 1.138)	1.022 (0.664, 1.572)	0.922
ASE vs PLA	1.281 (1.049, 1.563)	1.311 (1.037, 1.656)	24.1%	1.205 (0.824, 1.762)	1.087 (0.696, 1.699)	0.713
CARB vs LIT	1.311 (0.951, 1.806)	1.000 (0.414, 2.418)	na	1.366 (0.968, 1.927)	0.732 (0.284, 1.889)	0.519
CARB vs PLA	1.902 (1.409, 2.567)	1.970 (1.433, 2.709)	0.0%	1.443 (0.591, 3.523)	1.366 (0.529, 3.523)	0.519
CARI vs PLA		1.558 (1.262, 1.924)	39.0%			
END vs VALP		0.760 (0.520, 1.111)	23.9%			
ESL vs PLA		1.013 (0.763, 1.345)	69.0%			

HAL vs OLA	1.034 (0.885, 1.209)	1.088 (0.858, 1.382)	18.1%	0.995 (0.810, 1.223)	1.094 (0.798, 1.499)	0.578
HAL vs QUE	1.058 (0.867, 1.292)	1.318 (0.897, 1.937)	na	0.976 (0.773, 1.233)	1.350 (0.861, 2.117)	0.191
HAL vs RIS	0.973 (0.794, 1.192)	0.990 (0.696, 1.407)	na	0.964 (0.751, 1.237)	1.027 (0.667, 1.580)	0.905
HAL vs ZIP	1.215 (0.947, 1.560)	1.481 (1.044, 2.101)	na	0.988 (0.691, 1.413)	1.499 (0.909, 2.472)	0.113
HAL vs PLA	1.642 (1.432, 1.883)	1.559 (1.293, 1.879)	51.7%	1.744 (1.426, 2.133)	0.894 (0.679, 1.176)	0.423
LAM vs LIT	0.868 (0.680, 1.107)	0.933 (0.708, 1.230)	0.0%	0.679 (0.408, 1.130)	1.374 (0.770, 2.453)	0.282
LAM vs OLA	0.793 (0.609, 1.032)	1.143 (0.533, 2.452)	na	0.755 (0.570, 1.000)	1.514 (0.671, 3.415)	0.318
LAM vs PLA	1.259 (0.986, 1.608)	1.153 (0.855, 1.554)	63.8%	1.508 (0.984, 2.310)	0.765 (0.454, 1.287)	0.313
LIC vs PLA		1.019 (0.706, 1.469)	na			
LIT vs OLA	0.914 (0.782, 1.068	0.898 (0.677, 1.191	28.5%	0.921 (0.764, 1.110)	0.975 (0.695, 1.368)	0.883
LIT vs QUE	0.935 (0.784, 1.116)	0.807 (0.627, 1.039)	0.0%	1.078 (0.841, 1.382)	0.749 (0.526, 1.067)	0.109
LIT vs TOP	1.588 (1.267, 1.991)	1.712 (1.276, 2.297)	0.0%	1.426 (1.002, 2.029)	1.201 (0.759, 1.900)	0.435
LIT vs VALP	1.019 (0.835, 1.244)	1.136 (0.793, 1.628)	59.6%	0.972 (0.765, 1.234)	1.170 (0.759, 1.802)	0.477
LIT vs PLA	1.451 (1.275, 1.652)	1.547 (1.299, 1.843)	47.7%	1.342 (1.107, 1.628)	1.153 (0.889, 1.495)	0.284
OLA vs RIS	0.940 (0.777, 1.138)	1.034 (0.758, 1.411)	na	0.888 (0.697, 1.131)	1.166 (0.786, 1.728)	0.445
OLA vs VALP	1.115 (0.927, 1.342)	1.136 (0.881, 1.464)	43.0%	1.093 (0.834, 1.432)	1.039 (0.717, 1.506)	0.839
OLA vs PLA	1.588 (1.403, 1.797)	1.525 (1.277, 1.822)	35.6%	1.649 (1.389, 1.959)	0.925 (0.722, 1.184)	0.536
OXC vs VALP		0.889 (0.636, 1.243)	na			
PAL vs QUE	0.898 (0.702, 1.148)	1.140 (0.827, 1.571)	na	0.639 (0.435, 0.937)	1.784 (1.082, 2.943)	0.023
PAL vs PLA	1.393 (1.102, 1.761)	1.258 (0.967, 1.635)	81.6%	2.072 (1.236, 3.474)	0.607 (0.340, 1.084)	0.091
QUE vs PLA	1.552 (1.316, 1.830)	1.605 (1.302, 1.980)	66.7%	1.469 (1.126, 1.917)	1.093 (0.778, 1.533)	0.609
RIS vs PLA	1.689 (1.411, 2.021)	1.749 (1.396, 2.191)	42.5%	1.588 (1.179, 2.139)	1.101 (0.758, 1.601)	0.613
TAM vs PLA		7.461 (1.876, 29.678)	0.0%			
TOP vs PLA	0.914 (0.736, 1.135)	0.959 (0.755, 1.217)	0.0%	0.724 (0.428, 1.223)	1.325 (0.745, 2.358)	0.338
VALP vs PLA	1.424 (1.188, 1.707)	1.566 (1.226, 2.001)	56.2%	1.269 (0.969, 1.661)	1.234 (0.858, 1.776)	0.257

VER vs PLA		1.324 (0.249, 7.023)	na			
ZIP vs PLA	1.351 (1.061, 1.721)	1.575 (1.189, 2.086)	0.0%	0.868 (0.538, 1.398)	1.815 (1.043, 3.159)	0.035

95% CI: 95% confidence intervals, na: not applicable, NMA: network meta-analysis, RR: risk ratio

Funnel plot (only double-blind, placebo-controlled trials)

No comparisons included at least 10 studies. A funnel plot including all placebo-controlled trials was shown.

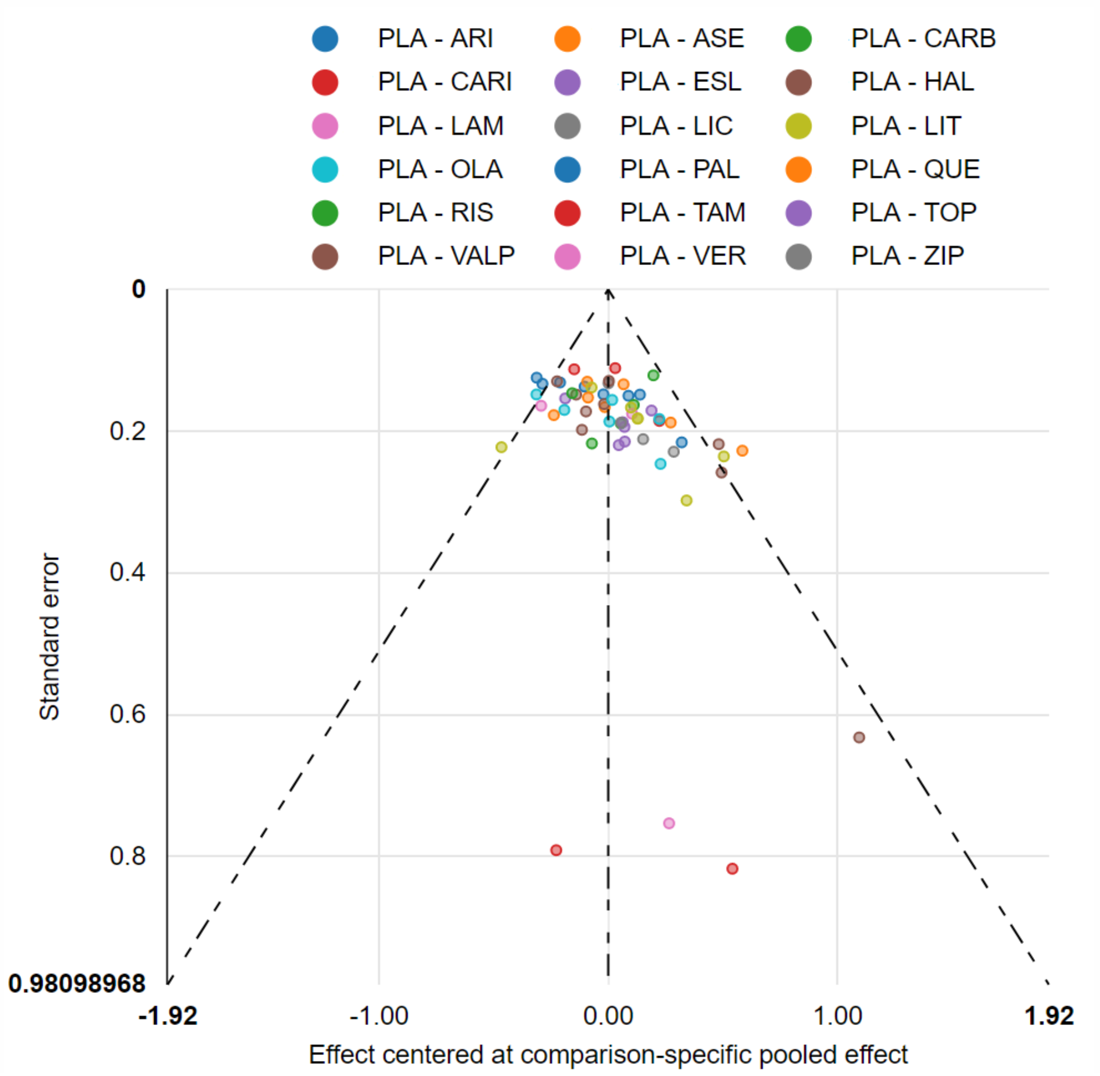

Meta-regression analysis (the placebo was the control)

	Primary analysis	Publication year (N = 56)	Mean age (N = 55)	Number of total individuals (N = 56)	%Male individuals (N = 54)	%Individuals with psychotic features (N = 35)
τ^2	0.017	0.011	0.017	0.016	0.013	0.011
β, median (95% CrI)		**−0.302 (−0.518, −0.093)**	−0.150 (−0.353, 0.051)	-0.133 (-0.316, 0.045)	**0.252 (0.063, 0.436)**	0.209 (-0.032, 0.447)
Interpretation of the results		Older studies had a higher RR.			Studies involving more male individuals had a higher RR.	

The results of network meta-analysis (drug vs. placebo) adjusting for the above moderators demonstrated an association with the effect size.

vs PLA	Primary analysis	Publication year	%Male individuals
**ARI**	**1.529 (1.327, 1.762)**	**1.584 (1.381, 1.807)**	**1.587 (1.385, 1.830)**
**ASE**	**1.281 (1.049, 1.563)**	**1.378 (1.131, 1.671)**	**1.286 (1.058, 1.562)**
**CARB**	**1.902 (1.409, 2.567)**	**1.819 (1.373, 2.447)**	**1.666 (1.243, 2.267)**
**CARI**	**1.558 (1.262, 1.924)**	**1.912 (1.488, 2.456)**	**1.393 (1.125, 1.727)**
END	1.082 (0.710, 1.648)	1.101 (0.736, 1.648)	
ESL	1.013 (0.763, 1.345)	1.301 (0.965, 1.816)	1.121 (0.854, 1.522)
**HAL**	**1.642 (1.432, 1.883)**	**1.660 (1.461, 1.896)**	**1.656 (1.451, 1.895)**
LAM	1.259 (0.986, 1.608)	1.202 (0.949, 1.520)	1.268 (0.994, 1.599)
LIC	1.019 (0.706, 1.469)	0.957 (0.676, 1.366)	0.991 (0.703, 1.405)
**LIT**	**1.451 (1.275, 1.652)**	**1.412 (1.254, 1.598)**	**1.442 (1.279, 1.637)**
**OLA**	**1.588 (1.403, 1.797)**	**1.589 (1.413, 1.777)**	**1.580 (1.404, 1.770)**
OXC	1.266 (0.865, 1.853)	1.234 (0.849, 1.762)	1.238 (0.842, 1.810)
**PAL**	**1.393 (1.102, 1.761)**	**1.524 (1.207, 1.911)**	**1.363 (1.077, 1.709)**
**QUE**	**1.552 (1.316, 1.830)**	**1.590 (1.366, 1.855)**	**1.512 (1.289, 1.773)**
**RIS**	**1.689 (1.411, 2.021)**	**1.645 (1.389, 1.952)**	**1.610 (1.364, 1.912)**

**TAM**	**7.461 (1.876, 29.678)**	**4.583 (1.911, 16.492)**	**4.043 (1.592, 14.428)**
TOP	0.914 (0.736, 1.135)	0.888 (0.724, 1.093)	0.915 (0.742, 1.133)
**VALP**	**1.424 (1.188, 1.707)**	**1.371 (1.158, 1.616)**	**1.388 (1.168, 1.651)**
VER	1.324 (0.249, 7.023)	0.796 (0.157, 4.434)	0.888 (0.184, 4.571)
**ZIP**	**1.351 (1.061, 1.721)**	**1.311 (1.060, 1.660)**	**1.320 (1.059, 1.667)**

Sensitivity analysis for response to treatment (design-adjusted model)

vs. PLA	Primary analysis (N = 56, τ^2 = 0.017)	Focusing on studies without a placebo arm (N = 56, τ^2 = 0.004)	Focusing on nonindustry-sponsored studies (N = 56, τ^2 = 0.003)	Focusing on studies with high-quality design (N = 56, τ^2 = 0.007)	Focusing on studies with 3-4 weeks data (N = 56, τ^2 = 0.017)	Focusing on studies not including individuals with rapid-cycling (N = 37, τ^2 = 0.015)	Focusing on studies not including individuals with mixed state/episode (N = 42, τ^2 = 0.006)	Focusing on studies including a common drug dose arm (N = 56, τ^2 = 0.017)	Focusing on studies that used common definition of response to treatment. (N = 56, τ^2 = 0.017)
ARI	**1.529 (1.327, 1.762)**	**1.572 (1.346, 1.836)**	**1.536 (1.324, 1.783)**	**1.545 (1.319, 1.824)**	**1.542 (1.333, 1.790)**	**1.499 (1.274, 1.767)**	**1.506 (1.291, 1.782)**	**1.548 (1.339, 1.790)**	**1.547 (1.341, 1.790)**
ASE	**1.281 (1.049, 1.563)**	**1.279 (1.023, 1.589)**	**1.278 (1.024, 1.580)**	**1.272 (1.039, 1.562)**	**1.294 (1.048, 1.599)**	**1.284 (1.039, 1.579)**	**1.279 (1.019, 1.602)**	**1.292 (1.058, 1.594)**	**1.296 (1.060, 1.589)**
CARB	**1.902 (1.409, 2.567)**	**1.893 (1.359, 2.667)**	**1.922 (1.335, 2.668)**	**1.949 (1.325, 2.845)**	**1.949 (1.422, 2.669)**		**1.991 (1.352, 3.012)**	**1.919 (1.424, 2.594)**	**1.909 (1.417, 2.598)**
CARI	**1.558 (1.262, 1.924)**	**1.539 (1.239, 1.950)**	**1.529 (1.240, 1.914)**	**1.539 (1.238, 1.951)**	**1.566 (1.257, 1.950)**	**1.561 (1.271, 1.945)**	**1.513 (1.119, 2.085)**	**1.561 (1.272, 1.955)**	**1.562 (1.265, 1.956)**
END	1.082 (0.710, 1.648)	1.063 (0.707, 1.621)	1.129 (0.631, 2.027)	1.169 (0.701, 2.129)	1.142 (0.743, 1.791)			1.136 (0.746, 1.749)	1.133 (0.741, 1.762)
ESL	1.013 (0.763, 1.345)	1.103 (0.779, 1.781)	1.106 (0.797, 1.776)	1.100 (0.786, 1.743)	1.077 (0.798, 1.506)	1.074 (0.801, 1.496)		1.070 (0.801, 1.520)	1.108 (0.762, 1.728)
HAL	**1.642 (1.432, 1.883)**	**1.629 (1.401, 1.874)**	**1.641 (1.427, 1.882)**	**1.659 (1.436, 1.915)**	**1.658 (1.439, 1.928)**	**1.525 (1.284, 1.807)**	**1.573 (1.323, 1.861)**	**1.662 (1.442, 1.918)**	**1.665 (1.449, 1.919)**
LAM	1.259 (0.986, 1.608)	1.270 (0.967, 1.667)	1.272 (0.992, 1.662)	1.275 (0.929, 1.693)	1.254 (0.932, 1.675)			1.327 (0.999, 1.719)	1.270 (0.981, 1.648)
LIC	1.019 (0.706, 1.469)	1.033 (0.693, 1.577)	1.048 (0.706, 1.554)	1.036 (0.689, 1.597)	1.026 (0.700, 1.505)		1.027 (0.681, 1.573)	1.024 (0.698, 1.483)	1.024 (0.705, 1.508)
LIT	**1.451 (1.275, 1.652)**	**1.435 (1.252, 1.640)**	**1.452 (1.274, 1.658)**	**1.458 (1.277, 1.665)**	**1.467 (1.278, 1.694)**	**1.426 (1.226, 1.690)**	**1.390 (1.192, 1.655)**	**1.489 (1.307, 1.705)**	**1.460 (1.287, 1.673)**
OLA	**1.588 (1.403, 1.797)**	**1.602 (1.406, 1.811)**	**1.587 (1.400, 1.793)**	**1.615 (1.428, 1.824)**	**1.609 (1.411, 1.842)**	**1.592 (1.363, 1.860)**	**1.595 (1.375, 1.845)**	**1.613 (1.421, 1.827)**	**1.615 (1.426, 1.837)**
OXC	1.266 (0.865, 1.853)	1.220 (0.858, 1.720)	1.254 (0.878, 1.726)	1.281 (0.796, 1.976)	1.305 (0.798, 2.108)	1.238 (0.801, 1.895)	1.245 (0.830, 1.862)	1.299 (0.866, 1.936)	1.294 (0.855, 1.944)
PAL	**1.393 (1.102, 1.761)**	**1.479 (1.125, 1.923)**	**1.437 (1.125, 1.913)**	**1.436 (1.152, 1.794)**	**1.404 (1.096, 1.804)**	**1.406 (1.103, 1.785)**	**1.440 (1.110, 1.883)**	**1.424 (1.114, 1.811)**	**1.416 (1.106, 1.809)**
QUE	**1.552 (1.316, 1.830)**	**1.592 (1.342, 1.884)**	**1.537 (1.291, 1.853)**	**1.558 (1.342, 1.835)**	**1.546 (1.304, 1.846)**	**1.528 (1.289, 1.826)**	**1.551 (1.321, 1.832)**	**1.582 (1.336, 1.878)**	**1.568 (1.328, 1.860)**
RIS	**1.689 (1.411, 2.021)**	**1.660 (1.384, 1.975)**	**1.660 (1.247, 2.028)**	**1.693 (1.404, 2.064)**	**1.705 (1.414, 2.061)**	**1.667 (1.367, 2.053)**	**1.654 (1.390, 1.982)**	**1.705 (1.419, 2.042)**	**1.707 (1.422, 2.053)**
TAM	**7.461 (1.876, 29.678)**	**13.741 (1.930, >100)**	**8.207 (2.970, 62.215)**	**8.006 (2.508, 45.833)**	**8.542 (2.698, 42.068)**	**8.742 (1.735, >100)**	**9.377 (2.042, >100)**	**9.930 (2.710, 76.269)**	**9.072 (2.737, 58.555)**
TOP	0.914 (0.736, 1.135)	0.909 (0.709, 1.179)	0.906 (0.709, 1.168)	0.916 (0.750, 1.125)	0.918 (0.733, 1.149)	0.913 (0.735, 1.138)	0.891 (0.686, 1.167)	0.926 (0.740, 1.147)	0.920 (0.734, 1.143)
VALP	**1.424 (1.188, 1.707)**	**1.368 (1.140, 1.636)**	**1.394 (1.150, 1.684)**	**1.447 (1.215, 1.715)**	**1.458 (1.215, 1.760)**	**1.397 (1.097, 1.785)**	**1.392 (1.090, 1.793)**	**1.457 (1.221, 1.756)**	**1.458 (1.212, 1.753)**
VER	1.324 (0.249, 7.023)	1.307 (0.108, 50.828)	1.500 (0.279, 7.506)	1.276 (0.242, 8.033)	1.374 (0.232, 10.682)		1.526 (0.087, 22.304)	1.340 (0.253, 10.584)	1.511 (0.121, 68.608)

ZIP	**1.351 (1.061, 1.721)**	**1.328 (1.020, 1.721)**	**1.322 (1.037, 1.758)**	**1.368 (1.069, 1.781)**	**1.371 (1.078, 1.767)**		**1.523 (1.020, 2.303)**	**1.369 (1.079, 1.761)**	**1.369 (1.080, 1.747)**

Studies with a placebo arm: when focusing on studies without a placebo arm

Studies supported by industry sponsors: when focusing on nonindustry-sponsored studies

Studies without high-quality design (i.e. studies with moderate overall risk of bias): when focusing on studies with high-quality design (i.e. studies with low overall risk of bias)

Studies without 3–4-week data: when focusing on studies with 3–4-week data

Studies that included individuals with rapid-cycling: when focusing on studies not including rapid-cycling individuals

Studies that included individuals with mixed state/episode: when focusing on studies not including individuals with mixed state/episode

Studies that included a very-low-dose arm: when focusing on studies including a common drug dose arm (we defined 50 mg/d as a very-low-dose arm [Goldsmith 2003], followed by the previous network meta-analysis [Yildiz 2015]).

Studies that did not use common definition of response to treatment (i.e., common definition was >50% improvement for the mania rating scale score): when focusing on studies that used common definition of response to treatment.

Risk ratio with 95% confidence intervals regarding the response to treatment at each observational point

	7–10 days* (N = 5)	3 weeks (N = 48)	4–6 weeks (N = 6)	8–12 weeks** (N = 6)
ARI vs PLA	**1.450 (1.163, 1.807)**	**1.539 (1.321, 1.794)**	Not estimated	**3.245 (2.249, 4.682)**
CARB vs PLA	Not estimated	**1.768 (1.049, 2.980)**	Not estimated	**1.767 (1.023, 3.232)**
HAL vs PLA	Not estimated	**1.640 (1.397, 1.924)**	**1.749 (1.320, 2.317)**	**1.882 (1.493, 2.371)**
LAM vs PLA	Not estimated	1.130 (0.752, 1.698)	1.223 (0.996, 1.868)	Not estimated
LIT vs PLA	**2.205 (1.583, 3.071)**	**1.543 (1.311, 1.815)**	**1.498 (1.162, 1.932)**	**1.767 (1.416, 2.206)**
OLA vs PLA	Not estimated	**1.589 (1.352, 1.868)**	**1.707 (1.319, 2.210)**	**1.924 (1.521, 2.433)**
QUE vs PLA	**1.672 (1.210, 2.310)**	**1.423 (1.186, 1.706)**	**1.954 (1.398, 2.732)**	**1.668 (1.370, 2.031)**

In this table, the results of only drugs for which a network meta-analysis for this outcome was performed at two or more observational points are shown.

Not estimated: Because there were no available data for performing a network meta-analysis, we did not estimate the risk ratio.

*Some studies reported the response to treatment at 1 week.

**Kakkar (2009) reported the response to treatment at 12 weeks. However, because the treatment arms in the study did not connect other treatment arms in this network, we excluded this study in this network meta-analysis.

CINeMA confidence rating

CINeMA is a web application that simplifies the evaluation of confidence in the findings from a network meta-analysis. CINeMA is based on a methodological framework described in the following articles, which consider the following six domains: within-study bias, reporting bias, indirectness, imprecision, heterogeneity, and incoherence. CINeMA grades the confidence in the results of each treatment comparison as high, moderate, low, or very low.

Nikolakopoulou A, et al., CINeMA: An approach for assessing confidence in the results of a network meta-analysis PLOS Medicine 2020 17 1-19

Papakonstantinou T, et al., CINeMA: Software for semiautomated assessment of the confidence in the results of network meta-analysis Campbell Systematic Reviews 2020 16 e1080

(1) Within-study bias: We referred to the following article:

Furukawa TA, et al., Comparative efficacy and acceptability of first-generation and second-generation antidepressants in the acute treatment of major depression: protocol for a network meta-analysis. BMJ Open. 2016 Jul 8;6(7):e010919. doi: 10.1136/bmjopen-2015-010919.

Selected rule: Average RoB

(2) Reporting bias: Comparison-adjusted funnel plots with less than 10 studies are not meaningful. Therefore, all comparisons were “Suspected.”

(3) Indirectness: No indirectness was assumed.

Selected rule: Average

(4) Imprecision: A clinically meaningful threshold was set at a risk ratio of higher or lower than 1. A clinically meaningful threshold was set at a standardized mean difference of higher or lower than 0.

(5) Heterogeneity: We used recommendations automatically provided by CINeMA.

(6) Incoherence: We used recommendations automatically provided by CINeMA.

*If the comparison had only indirect evidence, the comparison was downgraded one level.

Comparison	Number of studies	Within-study bias	Reporting bias	Indirectness	Imprecision	Heterogeneity	Incoherence	Confidence rating
ARI vs HAL	2	Some concerns	Suspected	No concerns	Major concerns	No concerns	No concerns	Very low
ARI vs LIT	1	Some concerns	Suspected	No concerns	Major concerns	No concerns	No concerns	Very low
ARI vs PLA	6	Some concerns	Suspected	No concerns	No concerns	No concerns	No concerns	Low
ASE vs OLA	2	Some concerns	Suspected	No concerns	No concerns	Major concerns	No concerns	Very low
ASE vs PLA	3	Some concerns	Suspected	No concerns	No concerns	Major concerns	No concerns	Very low
CARB vs LIT	1	Some concerns	Suspected	No concerns	Major concerns	No concerns	No concerns	Very low
CARB vs PLA	2	Some concerns	Suspected	No concerns	No concerns	No concerns	No concerns	Low
CARI vs PLA	3	Some concerns	Suspected	No concerns	No concerns	No concerns	Some concerns	Very low
END vs VALP	2	No concerns	Suspected	No concerns	Major concerns	No concerns	Some concerns	Very low
ESL vs PLA	2	Some concerns	Suspected	No concerns	Major concerns	No concerns	Some concerns	Very low
HAL vs OLA	2	Some concerns	Suspected	No concerns	Major concerns	No concerns	No concerns	Very low
HAL vs QUE	1	Some concerns	Suspected	No concerns	Major concerns	No concerns	No concerns	Very low
HAL vs RIS	1	Some concerns	Suspected	No concerns	Major concerns	No concerns	No concerns	Very low
HAL vs ZIP	1	Some concerns	Suspected	No concerns	Major concerns	No concerns	No concerns	Very low
HAL vs PLA	5	Some concerns	Suspected	No concerns	No concerns	No concerns	No concerns	Low
LAM vs LIT	3	Some concerns	Suspected	No concerns	Major concerns	No concerns	No concerns	Very low
LAM vs OLA	1	Some concerns	Suspected	No concerns	Major concerns	No concerns	No concerns	Very low
LAM vs PLA	2	Some concerns	Suspected	No concerns	Major concerns	No concerns	No concerns	Very low
LIC vs PLA	1	Some concerns	Suspected	No concerns	Major concerns	No concerns	Some concerns	Very low
LIT vs OLA	2	Some concerns	Suspected	No concerns	Major concerns	No concerns	No concerns	Very low
LIT vs QUE	2	No concerns	Suspected	No concerns	Major concerns	No concerns	No concerns	Low
LIT vs TOP	2	No concerns	Suspected	No concerns	No concerns	No concerns	No concerns	Moderate
LIT vs VALP	2	Some concerns	Suspected	No concerns	Major concerns	No concerns	No concerns	Very low
LIT vs PLA	7	Some concerns	Suspected	No concerns	No concerns	No concerns	No concerns	Low

OLA vs RIS	1	Some concerns	Suspected	No concerns	Major concerns	No concerns	No concerns	Very low
OLA vs VALP	2	No concerns	Suspected	No concerns	Major concerns	No concerns	No concerns	Low
OLA vs PLA	6	Some concerns	Suspected	No concerns	No concerns	No concerns	No concerns	Low
OXC vs VALP	1	Some concerns	Suspected	No concerns	Major concerns	No concerns	Some concerns	Very low
PAL vs QUE	1	No concerns	Suspected	No concerns	Major concerns	No concerns	Major concerns	Very low
PAL vs PLA	2	No concerns	Suspected	No concerns	No concerns	Major concerns	Major concerns	Very low
QUE vs PLA	4	No concerns	Suspected	No concerns	No concerns	No concerns	No concerns	Moderate
RIS vs PLA	3	Some concerns	Suspected	No concerns	No concerns	No concerns	No concerns	Low
TAM vs PLA	2	No concerns	Suspected	No concerns	No concerns	No concerns	Some concerns	Low
TOP vs PLA	4	No concerns	Suspected	No concerns	Major concerns	No concerns	No concerns	Low
VALP vs PLA	4	No concerns	Suspected	No concerns	No concerns	No concerns	No concerns	Moderate
VER vs PLA	1	No concerns	Suspected	No concerns	Major concerns	No concerns	Some concerns	Very low
ZIP vs PLA	3	Some concerns	Suspected	No concerns	No concerns	Major concerns	Major concerns	Very low
ARI vs ASE	0	Some concerns	Suspected	No concerns	Major concerns	No concerns	Some concerns	Very low
ARI vs CARB	0	Some concerns	Suspected	No concerns	Major concerns	No concerns	Some concerns	Very low
ARI vs CARI	0	Some concerns	Suspected	No concerns	Major concerns	No concerns	Some concerns	Very low
ARI vs END	0	No concerns	Suspected	No concerns	Major concerns	No concerns	Some concerns	Very low
ARI vs ESL	0	Some concerns	Suspected	No concerns	No concerns	Major concerns	Some concerns	Very low
ARI vs LAM	0	Some concerns	Suspected	No concerns	Major concerns	No concerns	Some concerns	Very low
ARI vs LIC	0	Some concerns	Suspected	No concerns	No concerns	Major concerns	Some concerns	Very low
ARI vs OLA	0	Some concerns	Suspected	No concerns	Major concerns	No concerns	Some concerns	Very low
ARI vs OXC	0	Some concerns	Suspected	No concerns	Major concerns	No concerns	Some concerns	Very low
ARI vs PAL	0	Some concerns	Suspected	No concerns	Major concerns	No concerns	Some concerns	Very low
ARI vs QUE	0	Some concerns	Suspected	No concerns	Major concerns	No concerns	Some concerns	Very low
ARI vs RIS	0	Some concerns	Suspected	No concerns	Major concerns	No concerns	Some concerns	Very low

ARI vs TAM	0	Some concerns	Suspected	No concerns	No concerns	No concerns	Some concerns	Very low
ARI vs TOP	0	Some concerns	Suspected	No concerns	No concerns	No concerns	Some concerns	Very low
ARI vs VALP	0	Some concerns	Suspected	No concerns	Major concerns	No concerns	Some concerns	Very low
ARI vs VER	0	Some concerns	Suspected	No concerns	Major concerns	No concerns	Some concerns	Very low
ARI vs ZIP	0	Some concerns	Suspected	No concerns	Major concerns	No concerns	Some concerns	Very low
ASE vs CARB	0	Some concerns	Suspected	No concerns	No concerns	Major concerns	Some concerns	Very low
ASE vs CARI	0	Some concerns	Suspected	No concerns	Major concerns	No concerns	Some concerns	Very low
ASE vs END	0	No concerns	Suspected	No concerns	Major concerns	No concerns	Some concerns	Very low
ASE vs ESL	0	Some concerns	Suspected	No concerns	Major concerns	No concerns	Some concerns	Very low
ASE vs HAL	0	Some concerns	Suspected	No concerns	No concerns	Major concerns	Some concerns	Very low
ASE vs LAM	0	Some concerns	Suspected	No concerns	Major concerns	No concerns	Some concerns	Very low
ASE vs LIC	0	Some concerns	Suspected	No concerns	Major concerns	No concerns	Some concerns	Very low
ASE vs LIT	0	Some concerns	Suspected	No concerns	Major concerns	No concerns	Some concerns	Very low
ASE vs OXC	0	Some concerns	Suspected	No concerns	Major concerns	No concerns	Some concerns	Very low
ASE vs PAL	0	No concerns	Suspected	No concerns	Major concerns	No concerns	Some concerns	Very low
ASE vs QUE	0	No concerns	Suspected	No concerns	Major concerns	No concerns	Some concerns	Very low
ASE vs RIS	0	Some concerns	Suspected	No concerns	No concerns	Major concerns	Some concerns	Very low
ASE vs TAM	0	No concerns	Suspected	No concerns	No concerns	No concerns	Some concerns	Very low
ASE vs TOP	0	No concerns	Suspected	No concerns	No concerns	Major concerns	Some concerns	Very low
ASE vs VALP	0	No concerns	Suspected	No concerns	Major concerns	No concerns	Some concerns	Very low
ASE vs VER	0	No concerns	Suspected	No concerns	Major concerns	No concerns	Some concerns	Very low
ASE vs ZIP	0	Some concerns	Suspected	No concerns	Major concerns	No concerns	Some concerns	Very low
CARB vs CARI	0	Some concerns	Suspected	No concerns	Major concerns	No concerns	Some concerns	Very low
CARB vs END	0	No concerns	Suspected	No concerns	No concerns	Major concerns	Some concerns	Very low
CARB vs ESL	0	Some concerns	Suspected	No concerns	No concerns	No concerns	Some concerns	Very low

CARB vs HAL	0	Some concerns	Suspected	No concerns	Major concerns	No concerns	Some concerns	Very low
CARB vs LAM	0	Some concerns	Suspected	No concerns	No concerns	Major concerns	Some concerns	Very low
CARB vs LIC	0	Some concerns	Suspected	No concerns	No concerns	No concerns	Some concerns	Very low
CARB vs OLA	0	Some concerns	Suspected	No concerns	Major concerns	No concerns	Some concerns	Very low
CARB vs OXC	0	Some concerns	Suspected	No concerns	Major concerns	No concerns	Some concerns	Very low
CARB vs PAL	0	No concerns	Suspected	No concerns	Major concerns	No concerns	Some concerns	Very low
CARB vs QUE	0	Some concerns	Suspected	No concerns	Major concerns	No concerns	Some concerns	Very low
CARB vs RIS	0	Some concerns	Suspected	No concerns	Major concerns	No concerns	Some concerns	Very low
CARB vs TAM	0	Some concerns	Suspected	No concerns	Major concerns	No concerns	Some concerns	Very low
CARB vs TOP	0	Some concerns	Suspected	No concerns	No concerns	No concerns	Some concerns	Very low
CARB vs VALP	0	Some concerns	Suspected	No concerns	Major concerns	No concerns	Some concerns	Very low
CARB vs VER	0	Some concerns	Suspected	No concerns	Major concerns	No concerns	Some concerns	Very low
CARB vs ZIP	0	Some concerns	Suspected	No concerns	Major concerns	No concerns	Some concerns	Very low
CARI vs END	0	No concerns	Suspected	No concerns	Major concerns	No concerns	Some concerns	Very low
CARI vs ESL	0	Some concerns	Suspected	No concerns	No concerns	Major concerns	Some concerns	Very low
CARI vs HAL	0	Some concerns	Suspected	No concerns	Major concerns	No concerns	Some concerns	Very low
CARI vs LAM	0	Some concerns	Suspected	No concerns	Major concerns	No concerns	Some concerns	Very low
CARI vs LIC	0	Some concerns	Suspected	No concerns	No concerns	Major concerns	Some concerns	Very low
CARI vs LIT	0	Some concerns	Suspected	No concerns	Major concerns	No concerns	Some concerns	Very low
CARI vs OLA	0	Some concerns	Suspected	No concerns	Major concerns	No concerns	Some concerns	Very low
CARI vs OXC	0	Some concerns	Suspected	No concerns	Major concerns	No concerns	Some concerns	Very low
CARI vs PAL	0	No concerns	Suspected	No concerns	Major concerns	No concerns	Some concerns	Very low
CARI vs QUE	0	Some concerns	Suspected	No concerns	Major concerns	No concerns	Some concerns	Very low
CARI vs RIS	0	Some concerns	Suspected	No concerns	Major concerns	No concerns	Some concerns	Very low
CARI vs TAM	0	Some concerns	Suspected	No concerns	No concerns	No concerns	Some concerns	Very low

CARI vs TOP	0	Some concerns	Suspected	No concerns	No concerns	No concerns	Some concerns	Very low
CARI vs VALP	0	Some concerns	Suspected	No concerns	Major concerns	No concerns	Some concerns	Very low
CARI vs VER	0	Some concerns	Suspected	No concerns	Major concerns	No concerns	Some concerns	Very low
CARI vs ZIP	0	Some concerns	Suspected	No concerns	Major concerns	No concerns	Some concerns	Very low
END vs ESL	0	No concerns	Suspected	No concerns	Major concerns	No concerns	Some concerns	Very low
END vs HAL	0	No concerns	Suspected	No concerns	Major concerns	No concerns	Some concerns	Very low
END vs LAM	0	No concerns	Suspected	No concerns	Major concerns	No concerns	Some concerns	Very low
END vs LIC	0	No concerns	Suspected	No concerns	Major concerns	No concerns	Some concerns	Very low
END vs LIT	0	No concerns	Suspected	No concerns	Major concerns	No concerns	Some concerns	Very low
END vs OLA	0	No concerns	Suspected	No concerns	Major concerns	No concerns	Some concerns	Very low
END vs OXC	0	Some concerns	Suspected	No concerns	Major concerns	No concerns	Some concerns	Very low
END vs PAL	0	No concerns	Suspected	No concerns	Major concerns	No concerns	Some concerns	Very low
END vs QUE	0	No concerns	Suspected	No concerns	Major concerns	No concerns	Some concerns	Very low
END vs RIS	0	No concerns	Suspected	No concerns	Major concerns	No concerns	Some concerns	Very low
END vs TAM	0	No concerns	Suspected	No concerns	No concerns	No concerns	Some concerns	Very low
END vs TOP	0	No concerns	Suspected	No concerns	Major concerns	No concerns	Some concerns	Very low
END vs VER	0	No concerns	Suspected	No concerns	Major concerns	No concerns	Some concerns	Very low
END vs ZIP	0	No concerns	Suspected	No concerns	Major concerns	No concerns	Some concerns	Very low
END vs PLA	0	No concerns	Suspected	No concerns	Major concerns	No concerns	Some concerns	Very low
ESL vs HAL	0	Some concerns	Suspected	No concerns	No concerns	No concerns	Some concerns	Very low
ESL vs LAM	0	Some concerns	Suspected	No concerns	Major concerns	No concerns	Some concerns	Very low
ESL vs LIC	0	Some concerns	Suspected	No concerns	Major concerns	No concerns	Some concerns	Very low
ESL vs LIT	0	Some concerns	Suspected	No concerns	No concerns	Major concerns	Some concerns	Very low
ESL vs OLA	0	Some concerns	Suspected	No concerns	No concerns	No concerns	Some concerns	Very low
ESL vs OXC	0	Some concerns	Suspected	No concerns	Major concerns	No concerns	Some concerns	Very low

ESL vs PAL	0	No concerns	Suspected	No concerns	Major concerns	No concerns	Some concerns	Very low
ESL vs QUE	0	Some concerns	Suspected	No concerns	No concerns	Major concerns	Some concerns	Very low
ESL vs RIS	0	Some concerns	Suspected	No concerns	No concerns	No concerns	Some concerns	Very low
ESL vs TAM	0	Some concerns	Suspected	No concerns	No concerns	No concerns	Some concerns	Very low
ESL vs TOP	0	Some concerns	Suspected	No concerns	Major concerns	No concerns	Some concerns	Very low
ESL vs VALP	0	Some concerns	Suspected	No concerns	No concerns	Major concerns	Some concerns	Very low
ESL vs VER	0	Some concerns	Suspected	No concerns	Major concerns	No concerns	Some concerns	Very low
ESL vs ZIP	0	Some concerns	Suspected	No concerns	Major concerns	No concerns	Some concerns	Very low
HAL vs LAM	0	Some concerns	Suspected	No concerns	Major concerns	No concerns	Some concerns	Very low
HAL vs LIC	0	Some concerns	Suspected	No concerns	No concerns	Major concerns	Some concerns	Very low
HAL vs LIT	0	Some concerns	Suspected	No concerns	Major concerns	No concerns	Some concerns	Very low
HAL vs OXC	0	Some concerns	Suspected	No concerns	Major concerns	No concerns	Some concerns	Very low
HAL vs PAL	0	No concerns	Suspected	No concerns	Major concerns	No concerns	Some concerns	Very low
HAL vs TAM	0	Some concerns	Suspected	No concerns	No concerns	No concerns	Some concerns	Very low
HAL vs TOP	0	Some concerns	Suspected	No concerns	No concerns	No concerns	Some concerns	Very low
HAL vs VALP	0	Some concerns	Suspected	No concerns	Major concerns	No concerns	Some concerns	Very low
HAL vs VER	0	Some concerns	Suspected	No concerns	Major concerns	No concerns	Some concerns	Very low
LAM vs LIC	0	Some concerns	Suspected	No concerns	Major concerns	No concerns	Some concerns	Very low
LAM vs OXC	0	Some concerns	Suspected	No concerns	Major concerns	No concerns	Some concerns	Very low
LAM vs PAL	0	Some concerns	Suspected	No concerns	Major concerns	No concerns	Some concerns	Very low
LAM vs QUE	0	Some concerns	Suspected	No concerns	Major concerns	No concerns	Some concerns	Very low
LAM vs RIS	0	Some concerns	Suspected	No concerns	Major concerns	No concerns	Some concerns	Very low
LAM vs TAM	0	Some concerns	Suspected	No concerns	No concerns	No concerns	Some concerns	Very low
LAM vs TOP	0	Some concerns	Suspected	No concerns	No concerns	Major concerns	Some concerns	Very low
LAM vs VALP	0	Some concerns	Suspected	No concerns	Major concerns	No concerns	Some concerns	Very low

LAM vs VER	0	Some concerns	Suspected	No concerns	Major concerns	No concerns	Some concerns	Very low
LAM vs ZIP	0	Some concerns	Suspected	No concerns	Major concerns	No concerns	Some concerns	Very low
LIC vs LIT	0	Some concerns	Suspected	No concerns	Major concerns	No concerns	Some concerns	Very low
LIC vs OLA	0	Some concerns	Suspected	No concerns	No concerns	Major concerns	Some concerns	Very low
LIC vs OXC	0	Some concerns	Suspected	No concerns	Major concerns	No concerns	Some concerns	Very low
LIC vs PAL	0	No concerns	Suspected	No concerns	Major concerns	No concerns	Some concerns	Very low
LIC vs QUE	0	Some concerns	Suspected	No concerns	No concerns	Major concerns	Some concerns	Very low
LIC vs RIS	0	Some concerns	Suspected	No concerns	No concerns	No concerns	Some concerns	Very low
LIC vs TAM	0	Some concerns	Suspected	No concerns	No concerns	No concerns	Some concerns	Very low
LIC vs TOP	0	Some concerns	Suspected	No concerns	Major concerns	No concerns	Some concerns	Very low
LIC vs VALP	0	Some concerns	Suspected	No concerns	Major concerns	No concerns	Some concerns	Very low
LIC vs VER	0	Some concerns	Suspected	No concerns	Major concerns	No concerns	Some concerns	Very low
LIC vs ZIP	0	Some concerns	Suspected	No concerns	Major concerns	No concerns	Some concerns	Very low
LIT vs OXC	0	Some concerns	Suspected	No concerns	Major concerns	No concerns	Some concerns	Very low
LIT vs PAL	0	No concerns	Suspected	No concerns	Major concerns	No concerns	Some concerns	Very low
LIT vs RIS	0	Some concerns	Suspected	No concerns	Major concerns	No concerns	Some concerns	Very low
LIT vs TAM	0	No concerns	Suspected	No concerns	No concerns	No concerns	Some concerns	Very low
LIT vs VER	0	No concerns	Suspected	No concerns	Major concerns	No concerns	Some concerns	Very low
LIT vs ZIP	0	Some concerns	Suspected	No concerns	Major concerns	No concerns	Some concerns	Very low
OLA vs OXC	0	Some concerns	Suspected	No concerns	Major concerns	No concerns	Some concerns	Very low
OLA vs PAL	0	No concerns	Suspected	No concerns	Major concerns	No concerns	Some concerns	Very low
OLA vs QUE	0	No concerns	Suspected	No concerns	Major concerns	No concerns	Some concerns	Very low
OLA vs TAM	0	No concerns	Suspected	No concerns	No concerns	No concerns	Some concerns	Very low
OLA vs TOP	0	No concerns	Suspected	No concerns	No concerns	No concerns	Some concerns	Very low
OLA vs VER	0	No concerns	Suspected	No concerns	Major concerns	No concerns	Some concerns	Very low

OLA vs ZIP	0	Some concerns	Suspected	No concerns	Major concerns	No concerns	Some concerns	Very low
OXC vs PAL	0	No concerns	Suspected	No concerns	Major concerns	No concerns	Some concerns	Very low
OXC vs QUE	0	Some concerns	Suspected	No concerns	Major concerns	No concerns	Some concerns	Very low
OXC vs RIS	0	Some concerns	Suspected	No concerns	Major concerns	No concerns	Some concerns	Very low
OXC vs TAM	0	No concerns	Suspected	No concerns	No concerns	No concerns	Some concerns	Very low
OXC vs TOP	0	No concerns	Suspected	No concerns	Major concerns	No concerns	Some concerns	Very low
OXC vs VER	0	No concerns	Suspected	No concerns	Major concerns	No concerns	Some concerns	Very low
OXC vs ZIP	0	Some concerns	Suspected	No concerns	Major concerns	No concerns	Some concerns	Very low
OXC vs PLA	0	Some concerns	Suspected	No concerns	Major concerns	No concerns	Some concerns	Very low
PAL vs RIS	0	Some concerns	Suspected	No concerns	Major concerns	No concerns	Some concerns	Very low
PAL vs TAM	0	No concerns	Suspected	No concerns	No concerns	No concerns	Some concerns	Very low
PAL vs TOP	0	No concerns	Suspected	No concerns	No concerns	No concerns	Some concerns	Very low
PAL vs VALP	0	No concerns	Suspected	No concerns	Major concerns	No concerns	Some concerns	Very low
PAL vs VER	0	No concerns	Suspected	No concerns	Major concerns	No concerns	Some concerns	Very low
PAL vs ZIP	0	No concerns	Suspected	No concerns	Major concerns	No concerns	Some concerns	Very low
QUE vs RIS	0	Some concerns	Suspected	No concerns	Major concerns	No concerns	Some concerns	Very low
QUE vs TAM	0	No concerns	Suspected	No concerns	No concerns	No concerns	Some concerns	Very low
QUE vs TOP	0	No concerns	Suspected	No concerns	No concerns	No concerns	Some concerns	Very low
QUE vs VALP	0	No concerns	Suspected	No concerns	Major concerns	No concerns	Some concerns	Very low
QUE vs VER	0	No concerns	Suspected	No concerns	Major concerns	No concerns	Some concerns	Very low
QUE vs ZIP	0	Some concerns	Suspected	No concerns	Major concerns	No concerns	Some concerns	Very low
RIS vs TAM	0	Some concerns	Suspected	No concerns	No concerns	No concerns	Some concerns	Very low
RIS vs TOP	0	Some concerns	Suspected	No concerns	No concerns	No concerns	Some concerns	Very low
RIS vs VALP	0	Some concerns	Suspected	No concerns	Major concerns	No concerns	Some concerns	Very low
RIS vs VER	0	Some concerns	Suspected	No concerns	Major concerns	No concerns	Some concerns	Very low

RIS vs ZIP	0	Some concerns	Suspected	No concerns	Major concerns	No concerns	Some concerns	Very low
TAM vs TOP	0	No concerns	Suspected	No concerns	No concerns	No concerns	Some concerns	Very low
TAM vs VALP	0	No concerns	Suspected	No concerns	No concerns	No concerns	Some concerns	Very low
TAM vs VER	0	No concerns	Suspected	No concerns	Major concerns	No concerns	Some concerns	Very low
TAM vs ZIP	0	No concerns	Suspected	No concerns	No concerns	No concerns	Some concerns	Very low
TOP vs VALP	0	No concerns	Suspected	No concerns	No concerns	No concerns	Some concerns	Very low
TOP vs VER	0	No concerns	Suspected	No concerns	Major concerns	No concerns	Some concerns	Very low
TOP vs ZIP	0	No concerns	Suspected	No concerns	No concerns	Major concerns	Some concerns	Very low
VALP vs VER	0	No concerns	Suspected	No concerns	Major concerns	No concerns	Some concerns	Very low
VALP vs ZIP	0	Some concerns	Suspected	No concerns	Major concerns	No concerns	Some concerns	Very low
VER vs ZIP	0	No concerns	Suspected	No concerns	Major concerns	No concerns	Some concerns	Very low

Supplementary Appendix 2. All-cause discontinuation (N = 70, n = 16324)

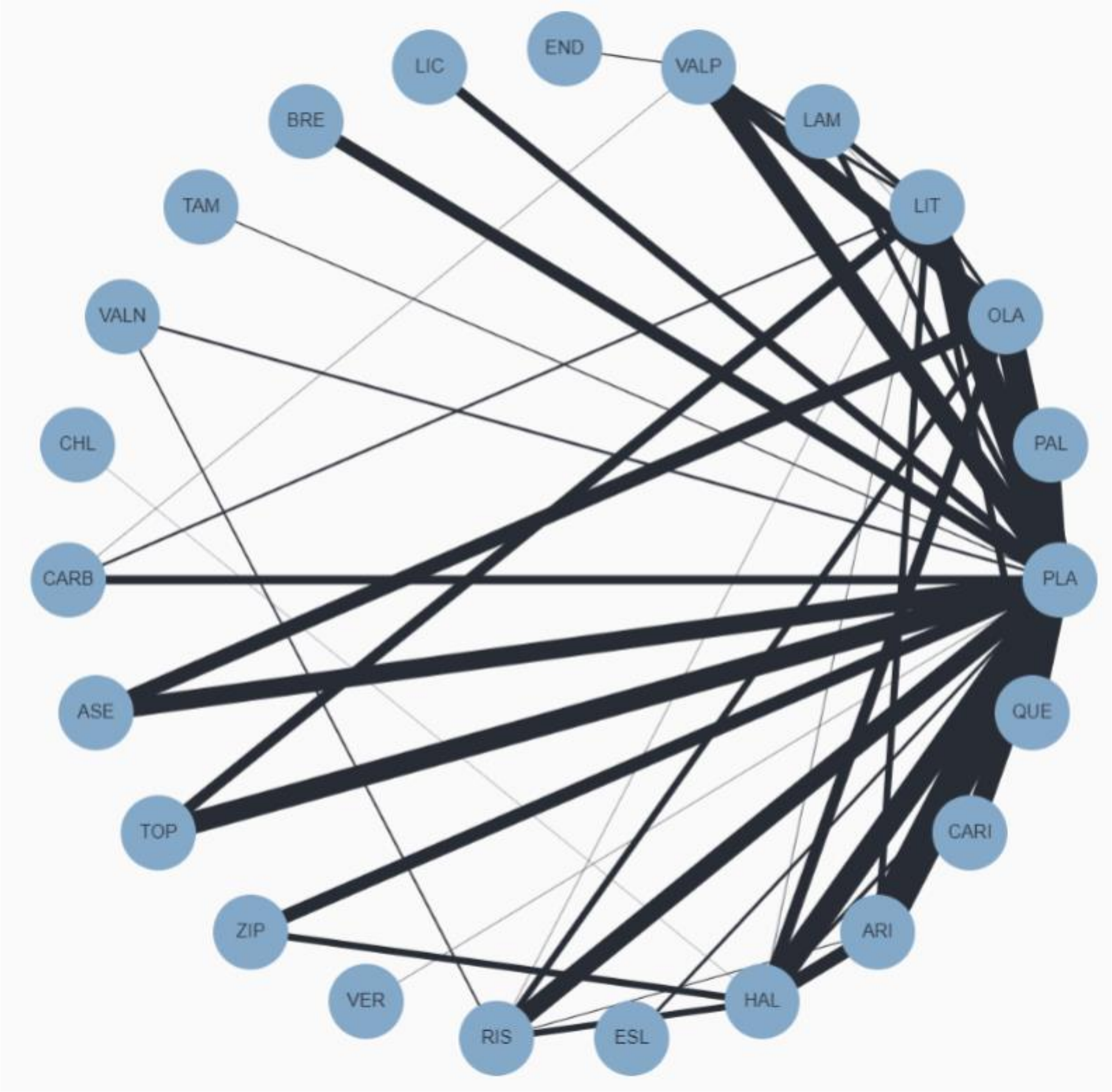

League table (risk ratio with 95% confidence interval)

ARI	0.841 (0.628, 1.127)	0.749 (0.494, 1.136)	0.993 (0.729, 1.352)	0.834 (0.609, 1.142)	0.316 (0.014, 7.087)	0.340 (0.034, 3.387)	1.035 (0.508, 2.107)	0.948 (0.767, 1.172)	0.754 (0.533, 1.067)	0.715 (0.450, 1.136)	0.816 (0.659, 1.009)	**1.297 (1.046, 1.609)**	1.103 (0.779, 1.562)	1.106 (0.829, 1.477)	1.074 (0.809, 1.425)	1.100 (0.515, 2.347)	**0.629 (0.467, 0.847)**	**0.479 (0.262, 0.876)**	0.989 (0.791, 1.237)	0.544 (0.286, 1.035)	0.970 (0.735, 1.282)	**0.840 (0.719, 0.980)**
	ASE	0.890 (0.561, 1.411)	1.180 (0.818, 1.704)	0.991 (0.684, 1.436)	0.376 (0.017, 8.486)	0.405 (0.040, 4.056)	1.230 (0.588, 2.572)	1.127 (0.834, 1.522)	0.897 (0.600, 1.339)	0.850 (0.514, 1.406)	0.969 (0.720, 1.306)	**1.542 (1.183, 2.010)**	1.311 (0.879, 1.956)	1.315 (0.926, 1.868)	1.276 (0.905, 1.800)	1.307 (0.597, 2.861)	0.747 (0.522, 1.070)	0.570 (0.302, 1.074)	1.176 (0.878, 1.575)	0.646 (0.330, 1.267)	1.154 (0.819, 1.626)	0.998 (0.777, 1.282)
		BRE	1.326 (0.828, 2.125)	1.114 (0.693, 1.789)	0.422 (0.018, 9.675)	0.455 (0.044, 4.649)	1.382 (0.624, 3.059)	1.266 (0.827, 1.938)	1.007 (0.612, 1.659)	0.955 (0.533, 1.711)	1.089 (0.715, 1.660)	**1.733 (1.141, 2.632)**	1.473 (0.896, 2.422)	1.478 (0.934, 2.339)	1.434 (0.908, 2.267)	1.469 (0.636, 3.392)	0.840 (0.528, 1.337)	0.640 (0.318, 1.290)	1.321 (0.867, 2.012)	0.726 (0.348, 1.514)	1.296 (0.824, 2.040)	1.121 (0.762, 1.651)
			CARB	0.840 (0.572, 1.233)	0.318 (0.014, 7.205)	0.343 (0.034, 3.445)	1.042 (0.495, 2.194)	0.955 (0.692, 1.317)	0.760 (0.505, 1.143)	0.720 (0.431, 1.203)	0.821 (0.610, 1.106)	1.307 (0.958, 1.782)	1.111 (0.736, 1.677)	1.114 (0.774, 1.604)	1.081 (0.752, 1.555)	1.108 (0.503, 2.440)	**0.633 (0.438, 0.916)**	**0.483 (0.254, 0.918)**	0.996 (0.730, 1.360)	0.548 (0.277, 1.081)	0.977 (0.683, 1.399)	0.846 (0.646, 1.107)
				CARI	0.379 (0.017, 8.585)	0.408 (0.041, 4.108)	1.241 (0.589, 2.617)	1.137 (0.820, 1.576)	0.905 (0.596, 1.373)	0.858 (0.513, 1.435)	0.978 (0.710, 1.348)	**1.556 (1.134, 2.135)**	1.323 (0.873, 2.004)	1.327 (0.918, 1.919)	1.288 (0.892, 1.859)	1.319 (0.598, 2.909)	0.754 (0.518, 1.099)	0.575 (0.301, 1.096)	1.186 (0.861, 1.634)	0.652 (0.330, 1.290)	1.164 (0.811, 1.672)	1.007 (0.766, 1.324)
					CHL	1.077 (0.023, 51.229)	3.275 (0.136, 79.146)	3.000 (0.135, 66.806)	2.387 (0.105, 54.277)	2.264 (0.098, 52.243)	2.581 (0.115, 58.009)	4.106 (0.183, 92.121)	3.491 (0.154, 79.353)	3.502 (0.155, 79.075)	3.399 (0.151, 76.715)	3.481 (0.142, 85.030)	1.990 (0.088, 45.024)	1.517 (0.064, 35.834)	3.131 (0.139, 70.344)	1.721 (0.072, 40.997)	3.072 (0.136, 69.214)	2.658 (0.119, 59.490)
						END	3.041 (0.277, 33.368)	2.785 (0.280, 27.752)	2.216 (0.219, 22.405)	2.102 (0.204, 21.685)	2.396 (0.241, 23.834)	3.812 (0.384, 37.845)	3.241 (0.320, 32.779)	3.251 (0.324, 32.619)	3.156 (0.315, 31.633)	3.232 (0.290, 35.978)	1.848 (0.184, 18.556)	1.408 (0.132, 15.001)	2.906 (0.295, 28.608)	1.598 (0.148, 17.202)	2.852 (0.285, 28.583)	2.467 (0.249, 24.432)
							ESL	0.916 (0.447, 1.876)	0.729 (0.340, 1.562)	0.691 (0.305, 1.569)	0.788 (0.386, 1.609)	1.254 (0.615, 2.555)	1.066 (0.498, 2.281)	1.069 (0.512, 2.234)	1.038 (0.497, 2.166)	1.063 (0.385, 2.936)	0.608 (0.290, 1.274)	0.463 (0.187, 1.147)	0.956 (0.468, 1.951)	0.526 (0.207, 1.337)	0.938 (0.451, 1.953)	0.811 (0.405, 1.624)

								HAL	0.796 (0.555, 1.140)	0.755 (0.471, 1.209)	0.860 (0.679, 1.090)	**1.369 (1.106, 1.694)**	1.164 (0.814, 1.664)	1.167 (0.871, 1.564)	1.133 (0.849, 1.513)	1.160 (0.541, 2.489)	**0.663 (0.485, 0.907)**	**0.506 (0.275, 0.929)**	1.044 (0.824, 1.322)	0.574 (0.300, 1.099)	1.024 (0.782, 1.341)	0.886 (0.741, 1.059)
									LAM	0.948 (0.554, 1.624)	1.081 (0.785, 1.489)	**1.720 (1.215, 2.436)**	1.462 (0.939, 2.278)	1.467 (0.986, 2.183)	1.424 (0.957, 2.119)	1.458 (0.651, 3.265)	0.834 (0.559, 1.243)	0.635 (0.327, 1.234)	1.311 (0.923, 1.862)	0.721 (0.358, 1.452)	1.287 (0.868, 1.908)	1.113 (0.812, 1.526)
										LIC	1.140 (0.715, 1.819)	**1.814 (1.141, 2.884)**	1.542 (0.902, 2.636)	1.547 (0.937, 2.554)	1.501 (0.911, 2.475)	1.538 (0.650, 3.636)	0.879 (0.530, 1.459)	0.670 (0.323, 1.389)	1.383 (0.867, 2.204)	0.760 (0.355, 1.629)	1.357 (0.826, 2.228)	1.174 (0.759, 1.815)
											LIT	**1.591 (1.278, 1.981)**	1.352 (0.951, 1.924)	**1.357 (1.016, 1.812)**	1.317 (0.983, 1.765)	1.349 (0.630, 2.886)	0.771 (0.581, 1.023)	0.588 (0.320, 1.078)	1.213 (0.975, 1.508)	0.667 (0.349, 1.273)	1.190 (0.892, 1.588)	1.030 (0.871, 1.217)
												OLA	0.850 (0.599, 1.206)	0.853 (0.638, 1.141)	0.828 (0.633, 1.083)	0.848 (0.397, 1.810)	**0.485 (0.359, 0.655)**	**0.369 (0.202, 0.674)**	**0.762 (0.626, 0.928)**	**0.419 (0.220, 0.798)**	**0.748 (0.565, 0.990)**	**0.647 (0.552, 0.758)**
													PAL	1.003 (0.698, 1.441)	0.974 (0.655, 1.447)	0.997 (0.446, 2.230)	**0.570 (0.380, 0.855)**	**0.434 (0.224, 0.843)**	0.897 (0.630, 1.277)	**0.493 (0.245, 0.991)**	0.880 (0.595, 1.301)	0.761 (0.557, 1.040)
														QUE	0.971 (0.686, 1.372)	0.994 (0.455, 2.173)	**0.568 (0.398, 0.811)**	**0.433 (0.230, 0.817)**	0.894 (0.665, 1.202)	**0.491 (0.251, 0.962)**	0.877 (0.624, 1.232)	**0.759 (0.592, 0.972)**
															RIS	1.024 (0.469, 2.237)	**0.586 (0.411, 0.835)**	**0.446 (0.246, 0.810)**	0.921 (0.689, 1.230)	**0.506 (0.259, 0.990)**	0.904 (0.645, 1.267)	**0.782 (0.612, 0.999)**

																TAM	0.572 (0.261, 1.254)	0.436 (0.169, 1.121)	0.899 (0.420, 1.924)	0.494 (0.187, 1.304)	0.882 (0.405, 1.923)	0.763 (0.363, 1.604)
																	TOP	0.762 (0.402, 1.443)	**1.573 (1.161, 2.131)**	0.865 (0.440, 1.700)	**1.543 (1.088, 2.190)**	**1.335 (1.032, 1.728)**
																		VALN	**2.064 (1.126, 3.784)**	1.135 (0.482, 2.669)	**2.025 (1.079, 3.802)**	1.752 (0.977, 3.142)
																			VALP	0.550 (0.288, 1.049)	0.981 (0.736, 1.308)	0.849 (0.719, 1.002)
																				VER	1.785 (0.915, 3.482)	1.544 (0.827, 2.884)
																					ZIP	0.865 (0.683, 1.096)
																						PLA

Evaluation of heterogeneity and inconsistency

Between study variance (τ^2)	Heterogeneity assessment	Random-effects design-by-treatment interaction model		
		Q	df	p
0.031	Low	61.469	40	0.016

Incoherence

	NMA, RR (95% CI)	Direct, RR (95% CI)	I^2	Indirect, RR (95% CI)	Inconsistency measures	
					Difference of RR	P value
ARI vs HAL	0.948 (0.767, 1.172)	0.679 (0.483, 0.956)	80.9%	1.167 (0.891, 1.529)	0.582 (0.377, 0.900)	0.015
ARI vs LIT	0.816 (0.659, 1.009)	1.006 (0.683, 1.481)	0.0%	0.744 (0.577, 0.961)	1.351 (0.850, 2.148)	0.203
ARI vs RIS	1.074 (0.809, 1.425)	0.714 (0.240, 2.124)	na	1.106 (0.825, 1.482)	0.646 (0.209, 1.996)	0.448
ARI vs PLA	0.840 (0.719, 0.980)	0.905 (0.764, 1.073)	39.1%	0.574 (0.393, 0.840)	1.577 (1.040, 2.391)	0.032
ASE vs OLA	1.542 (1.183, 2.010)	1.680 (1.198, 2.356)	0.0%	1.346 (0.879, 2.061)	1.248 (0.725, 2.150)	0.424
ASE vs PLA	0.998 (0.777, 1.282)	0.934 (0.712, 1.225)	24.1%	1.460 (0.763, 2.793)	0.640 (0.317, 1.293)	0.213
BRE vs PLA		1.121 (0.762, 1.651)	0.0%			
CARB vs LIT	0.821 (0.610, 1.106)	0.755 (0.421, 1.354)	53.7%	0.846 (0.598, 1.196)	0.892 (0.453, 1.760)	0.742
CARB vs VALP	0.996 (0.730, 1.360)	1.000 (0.229, 4.358)	na	0.996 (0.724, 1.369)	1.004 (0.223, 4.527)	0.996
CARB vs PLA	0.846 (0.646, 1.107)	0.865 (0.637, 1.176)	20.1%	0.782 (0.445, 1.375)	1.106 (0.582, 2.103)	0.758
CARI vs PLA		1.007 (0.766, 1.324)	0.0%			
CHL vs HAL		3.000 (0.135, 66.806)	na			
END vs VALP		2.906 (0.295, 28.608)	16.4%			
ESL vs PLA		0.811 (0.405, 1.624)	na			
HAL vs LIT	0.860 (0.679, 1.090)	0.836 (0.261, 2.679)	48.2%	0.862 (0.676, 1.097)	0.970 (0.295, 3.187)	0.960
HAL vs OLA	1.369 (1.106, 1.694)	1.443 (1.021, 2.039)	62.9%	1.325 (1.011, 1.738)	1.088 (0.701, 1.689)	0.705
HAL vs QUE	1.167 (0.871, 1.564)	0.630 (0.357, 1.111)	na	1.459 (1.037, 2.053)	0.431 (0.222, 0.837)	0.013

HAL vs RIS	1.133 (0.849, 1.513)	0.963 (0.485, 1.914)	0.0%	1.173 (0.853, 1.613)	0.821 (0.385, 1.750)	0.609
HAL vs ZIP	1.024 (0.782, 1.341)	0.932 (0.632, 1.375)	na	1.117 (0.768, 1.625)	0.834 (0.486, 1.431)	0.510
HAL vs PLA	0.886 (0.741, 1.059)	0.819 (0.652, 1.029)	54.8%	1.004 (0.753, 1.338)	0.816 (0.565, 1.178)	0.277
LAM vs LIT	1.081 (0.785, 1.489)	1.077 (0.743, 1.561)	82.4%	1.095 (0.583, 2.056)	0.983 (0.473, 2.044)	0.964
LAM vs OLA	1.720 (1.215, 2.436)	0.667 (0.125, 3.560)	na	1.795 (1.258, 2.563)	0.371 (0.067, 2.059)	0.257
LAM vs PLA	1.113 (0.812, 1.526)	1.153 (0.808, 1.647)	0.0%	0.980 (0.499, 1.926)	1.177 (0.548, 2.526)	0.676
LIC vs PLA		1.174 (0.759, 1.815)	na			
LIT vs OLA	1.591 (1.278, 1.981)	1.556 (0.754, 3.209)	13.5%	1.594 (1.267, 2.007)	0.976 (0.457, 2.086)	0.950
LIT vs QUE	1.357 (1.016, 1.812)	2.116 (1.084, 4.130)	45.1%	1.225 (0.888, 1.688)	1.728 (0.823, 3.628)	0.148
LIT vs RIS	1.317 (0.983, 1.765)	0.500 (0.049, 5.072)	na	1.337 (0.996, 1.797)	0.374 (0.036, 3.864)	0.409
LIT vs TOP	0.771 (0.581, 1.023)	0.927 (0.606, 1.419)	44.2%	0.666 (0.456, 0.973)	1.392 (0.787, 2.461)	0.255
LIT vs VALP	1.213 (0.975, 1.508)	1.318 (0.886, 1.960)	0.0%	1.170 (0.902, 1.518)	1.126 (0.700, 1.811)	0.624
LIT vs PLA	1.030 (0.871, 1.217)	0.950 (0.776, 1.163)	61.2%	1.224 (0.909, 1.647)	0.777 (0.542, 1.112)	0.168
OLA vs RIS	0.828 (0.633, 1.083)	0.644 (0.390, 1.064)	na	0.915 (0.666, 1.259)	0.704 (0.388, 1.275)	0.247
OLA vs VALP	0.762 (0.626, 0.928)	0.909 (0.677, 1.220)	0.0%	0.661 (0.507, 0.862)	1.376 (0.926, 2.045)	0.115
OLA vs PLA	0.647 (0.552, 0.758)	0.634 (0.517, 0.778)	35.5%	0.667 (0.520, 0.856)	0.950 (0.688, 1.312)	0.757
PAL vs QUE	1.003 (0.698, 1.441)	0.966 (0.575, 1.620)	na	1.041 (0.626, 1.729)	0.928 (0.449, 1.916)	0.840
PAL vs PLA	0.761 (0.557, 1.040)	0.731 (0.529, 1.012)	84.2%	1.258 (0.400, 3.961)	0.581 (0.176, 1.914)	0.372
QUE vs PLA	0.759 (0.592, 0.972)	0.704 (0.539, 0.920)	79.2%	1.205 (0.620, 2.339)	0.584 (0.286, 1.195)	0.141
RIS vs VALN	0.446 (0.246, 0.810)	0.575 (0.259, 1.276)	na	0.323 (0.131, 0.794)	1.781 (0.536, 5.922)	0.346
RIS vs PLA	0.782 (0.612, 0.999)	0.678 (0.506, 0.910)	58.1%	1.076 (0.693, 1.673)	0.630 (0.371, 1.071)	0.088
TAM vs PLA		0.763 (0.363, 1.604)	36.3%			
TOP vs PLA	1.335 (1.032, 1.728)	1.388 (1.053, 1.830)	0.0%	1.028 (0.501, 2.111)	1.350 (0.625, 2.918)	0.445
VALN vs PLA	1.752 (0.977, 3.142)	2.048 (1.050, 3.995)	na	1.057 (0.317, 3.520)	1.937 (0.489, 7.672)	0.346
VALP vs PLA	0.849 (0.719, 1.002)	0.917 (0.756, 1.112)	0.0%	0.682 (0.493, 0.944)	1.344 (0.921, 1.962)	0.125

VER vs PLA		1.544 (0.827, 2.884)	na			
ZIP vs PLA	0.865 (0.683, 1.096)	0.838 (0.653, 1.074)	0.0%	1.190 (0.545, 2.598)	0.704 (0.310, 1.598)	0.401

Funnel plot (only double-blind, placebo-controlled trials)

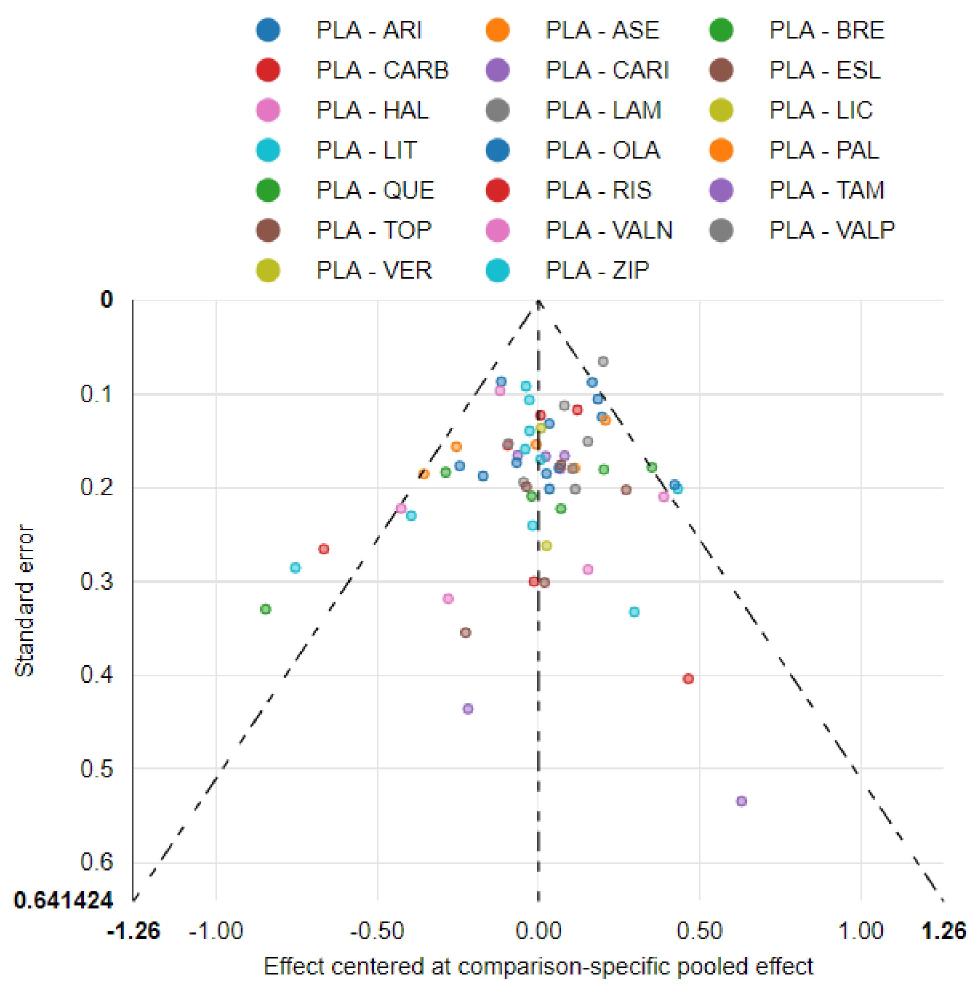

Meta-regression analysis (the placebo was the control)

	Primary analysis (N = 70)	Publication year (N = 70)	Mean age (N = 66)	Number of total individuals (N = 70)	%Male individuals (N = 67)	%Individuals with psychotic features (N = 38)
τ^2	0.031	0.033	0.035	0.035	0.035	0.007
β, median (95% CrI)		0.151 (−0.147, 0.458)	0.231 (−0.060, 0.536)	−0.059 (−0.267, 0.151)	−0.117 (−0.445, 0.191)	**−0.193 (−0.374, −0.026)**
Interpretation of the results						Studies involving more patients with psychotic features had a lower RR.

The results of network meta-analysis (drug vs. placebo) adjusting for the above moderators demonstrated an association with the effect size.

vs PLA	Primary analysis	%Individuals with psychotic features
ARI	**0.840 (0.719, 0.980)**	**0.724 (0.639, 0.821)**
ASE	0.998 (0.777, 1.282)	
BRE	1.121 (0.762, 1.651)	
CARB	0.846 (0.646, 1.107)	
CARI	1.007 (0.766, 1.324)	**0.738 (0.544, 0.991)**
CHL	2.658 (0.119, 59.490)	
END	2.467 (0.249, 24.432)	
ESL	0.811 (0.405, 1.624)	0.663 (0.435, 1.030)
HAL	0.886 (0.741, 1.059)	**0.694 (0.600, 0.794)**
LAM	1.113 (0.812, 1.526)	
LIC	1.174 (0.759, 1.815)	1.018 (0.801, 1.292)
LIT	1.030 (0.871, 1.217)	**0.777 (0.691, 0.891)**
OLA	**0.647 (0.552, 0.758)**	**0.709 (0.618, 0.802)**

PAL	0.761 (0.557, 1.040)	**0.769 (0.631, 0.924)**
QUE	**0.759 (0.592, 0.972)**	**0.732 (0.625, 0.852)**
RIS	**0.782 (0.612, 0.999)**	**0.701 (0.597, 0.812)**
TAM	0.763 (0.363, 1.604)	**0.660 (0.441, 0.929)**
TOP	1.335 (1.032, 1.728)	1.010 (0.902, 1.143)
VALN	1.752 (0.977, 3.142)	
VALP	0.849 (0.719, 1.002)	**0.765 (0.644, 0.901)**
VER	1.544 (0.827, 2.884)	1.162 (0.744, 1.810)
ZIP	0.865 (0.683, 1.096)	**0.794 (0.641, 0.987)**

The results of network meta-analysis (drug vs. placebo) adjusting for the above moderators demonstrated an association with the effect size.

vs PLA	Primary analysis (N = 70, τ^2 = 0.031)	Focusing on studies without a placebo arm (N = 70, τ^2 = 0.016)	Focusing on nonindustry-sponsored studies (N = 70, τ^2 = 0.005)	Focusing on studies with high-quality design (N = 70, τ^2 = 0.015)	Focusing on studies with 3-4 weeks' data (N = 70, τ^2 = 0.031)	Focusing on studies not including individuals with rapid-cycling (N = 38, τ^2 = 0.053)	Focusing on studies not including individuals with mixed state/episode (N = 48, τ^2 = 0.031)	Focusing on studies including a common drug dose arm (N = 70, τ^2 = 0.094)
ARI	**0.840 (0.719, 0.980)**	**0.823 (0.709, 0.955)**	**0.846 (0.737, 0.968)**	**0.844 (0.721, 0.985)**	**0.837 (0.709, 0.983)**	**0.828 (0.670, 0.988)**	**0.837 (0.690, 0.999)**	**0.682 (0.514, 0.904)**
ASE	0.998 (0.777, 1.282)	0.980 (0.743, 1.286)	0.987 (0.764, 1.281)	1.007 (0.777, 1.300)	0.985 (0.765, 1.271)	0.971 (0.716, 1.332)	0.971 (0.709, 1.312)	0.964 (0.630, 1.464)
BRE	1.121 (0.762, 1.651)	1.064 (0.669, 1.696)	1.081 (0.690, 1.639)	1.062 (0.667, 1.680)	1.097 (0.739, 1.624)	1.098 (0.696, 1.729)		1.151 (0.641, 2.058)
CARB	0.846 (0.646, 1.107)	0.857 (0.652, 1.118)	0.854 (0.657, 1.101)	0.857 (0.651, 1.132)	0.888 (0.672, 1.176)		0.924 (0.662, 1.301)	0.703 (0.434, 1.130)
CARI	1.007 (0.766, 1.324)	0.984 (0.719, 1.321)	0.980 (0.731, 1.313)	0.976 (0.724, 1.331)	0.993 (0.754, 1.315)	0.995 (0.713, 1.402)	0.945 (0.625, 1.409)	1.015 (0.641, 1.596)
CHL	2.658 (0.119, 59.490)	>10 (3.149, >100)	>10 (1.598, >100)	>10 (1.308, >100)	>10 (1.950, >100)			>10 (3.754, >100)
END	2.467 (0.249, 24.432)	>10 (2.858, >100)	>10 (2.526, >100)	>10 (8.375, >100)	>10 (6.829, >100)			>10 (9.091, >100)
ESL	0.811 (0.405, 1.624)	0.766 (0.337, 1.891)	0.738 (0.314, 1.826)	0.763 (0.331, 2.100)	0.783 (0.400, 1.585)	0.788 (0.377, 1.718)		0.771 (0.280, 2.220)
HAL	0.886 (0.741, 1.059)	0.894 (0.739, 1.073)	0.873 (0.731, 1.050)	0.868 (0.715, 1.054)	0.871 (0.721, 1.049)	0.871 (0.643, 1.150)	0.845 (0.646, 1.095)	0.757 (0.558, 1.021)
LAM	1.113 (0.812, 1.526)	1.106 (0.766, 1.600)	1.098 (0.768, 1.526)	1.117 (0.774, 1.617)	1.063 (0.736, 1.542)			1.259 (0.694, 2.212)
LIC	1.174 (0.759, 1.815)	1.156 (0.734, 1.848)	1.139 (0.780, 1.797)	1.165 (0.747, 1.866)	1.167 (0.741, 1.855)		1.155 (0.690, 1.947)	1.275 (0.612, 2.656)
LIT	1.030 (0.871, 1.217)	1.001 (0.841, 1.194)	0.981 (0.828, 1.156)	0.985 (0.832, 1.173)	0.989 (0.830, 1.178)	1.013 (0.771, 1.357)	0.940 (0.730, 1.204)	0.991 (0.748, 1.296)
OLA	**0.647 (0.552, 0.758)**	**0.648 (0.546, 0.761)**	**0.653 (0.554, 0.762)**	**0.666 (0.560, 0.786)**	**0.635 (0.536, 0.747)**	**0.604 (0.478, 0.754)**	**0.627 (0.507, 0.770)**	**0.491 (0.374, 0.640)**
PAL	0.761 (0.557, 1.040)	0.767 (0.550, 1.077)	0.783 (0.586, 1.061)	**0.766 (0.576, 0.992)**	0.760 (0.551, 1.035)	0.737 (0.503, 1.074)	0.756 (0.524, 1.095)	0.653 (0.388, 1.094)
QUE	**0.759 (0.592, 0.972)**	**0.717 (0.534, 0.951)**	**0.736 (0.572, 0.998)**	**0.730 (0.571, 0.920)**	**0.753 (0.579, 0.957)**	**0.698 (0.509, 0.929)**	**0.709 (0.532, 0.932)**	**0.640 (0.426, 0.923)**
RIS	**0.782 (0.612, 0.999)**	**0.780 (0.602, 0.995)**	**0.767 (0.602, 0.975)**	**0.760 (0.581, 0.985)**	**0.742 (0.577, 0.950)**	**0.646 (0.428, 0.964)**	**0.749 (0.567, 0.989)**	**0.669 (0.463, 0.983)**
TAM	0.763 (0.363, 1.604)	0.646 (0.223, 1.572)	0.711 (0.346, 1.327)	0.692 (0.329, 1.338)	0.690 (0.321, 1.415)	0.957 (0.139, 4.711)	0.628 (0.220, 1.562)	0.625 (0.204, 1.911)
TOP	1.335 (1.032, 1.728)	1.292 (0.970, 1.735)	1.283 (0.993, 1.682)	1.304 (1.037, 1.641)	1.297 (0.995, 1.663)	1.294 (0.960, 1.767)	1.268 (0.928, 1.764)	1.464 (0.971, 2.193)
VALN	1.752 (0.977, 3.142)	1.666 (0.814, 3.545)	1.709 (1.051, 2.879)	1.724 (1.010, 2.974)	1.699 (0.966, 3.114)		1.705 (0.948, 3.116)	2.208 (0.919, 5.416)

VALP	0.849 (0.719, 1.002)	**0.843 (0.701, 0.987)**	**0.871 (0.740, 0.997)**	**0.859 (0.722, 0.993)**	**0.829 (0.692, 0.979)**	0.747 (0.535, 1.035)	0.854 (0.648, 1.068)	**0.691 (0.500, 0.963)**
VER	1.544 (0.827, 2.884)	1.394 (0.670, 3.429)	1.496 (0.895, 2.685)	1.487 (0.853, 2.908)	1.473 (0.823, 2.953)		1.399 (0.636, 3.586)	4.591 (0.830, 30.392)
ZIP	0.865 (0.683, 1.096)	0.861 (0.676, 1.101)	0.860 (0.696, 1.066)	0.860 (0.684, 1.076)	0.858 (0.672, 1.102)		0.834 (0.564, 1.243)	0.738 (0.465, 1.172)

CINeMA confidence rating

Comparison	Number of studies	Within-study bias	Reporting bias	Indirectness	Imprecision	Heterogeneity	Incoherence	Confidence rating
ARI vs HAL	2	Some concerns	Suspected	No concerns	Major concerns	No concerns	Major concerns	Very low
ARI vs LIT	2	Some concerns	Suspected	No concerns	Major concerns	No concerns	No concerns	Very low
ARI vs RIS	1	Some concerns	Suspected	No concerns	Major concerns	No concerns	No concerns	Very low
ARI vs PLA	6	Some concerns	Suspected	No concerns	No concerns	Major concerns	Major concerns	Very low
ASE vs OLA	2	Some concerns	Suspected	No concerns	No concerns	Major concerns	No concerns	Very low
ASE vs PLA	3	Some concerns	Suspected	No concerns	Major concerns	No concerns	No concerns	Very low
BRE vs PLA	2	Some concerns	Suspected	No concerns	Major concerns	No concerns	Major concerns	Very low
CARB vs LIT	3	Some concerns	Suspected	No concerns	Major concerns	No concerns	No concerns	Very low
CARB vs VALP	1	Some concerns	Suspected	No concerns	Major concerns	No concerns	No concerns	Very low
CARB vs PLA	2	Some concerns	Suspected	No concerns	Major concerns	No concerns	No concerns	Very low
CARI vs PLA	3	Some concerns	Suspected	No concerns	Major concerns	No concerns	Major concerns	Very low
CHL vs HAL	1	Some concerns	Suspected	No concerns	Major concerns	No concerns	Major concerns	Very low
END vs VALP	2	No concerns	Suspected	No concerns	Major concerns	No concerns	Major concerns	Very low
ESL vs PLA	1	Some concerns	Suspected	No concerns	Major concerns	No concerns	Major concerns	Very low
HAL vs LIT	2	Some concerns	Suspected	No concerns	Major concerns	No concerns	No concerns	Very low
HAL vs OLA	2	Some concerns	Suspected	No concerns	No concerns	Major concerns	No concerns	Very low
HAL vs QUE	1	Some concerns	Suspected	No concerns	Major concerns	No concerns	Major concerns	Very low
HAL vs RIS	2	Some concerns	Suspected	No concerns	Major concerns	No concerns	No concerns	Very low
HAL vs ZIP	1	Some concerns	Suspected	No concerns	Major concerns	No concerns	No concerns	Very low
HAL vs PLA	5	Some concerns	Suspected	No concerns	Major concerns	No concerns	No concerns	Very low
LAM vs LIT	3	Some concerns	Suspected	No concerns	Major concerns	No concerns	No concerns	Very low
LAM vs OLA	1	Some concerns	Suspected	No concerns	No concerns	No concerns	No concerns	Low
LAM vs PLA	2	Some concerns	Suspected	No concerns	Major concerns	No concerns	No concerns	Very low

LIC vs PLA	1	Some concerns	Suspected	No concerns	Major concerns	No concerns	Major concerns	Very low
LIT vs OLA	3	Some concerns	Suspected	No concerns	No concerns	No concerns	No concerns	Low
LIT vs QUE	2	Some concerns	Suspected	No concerns	No concerns	Major concerns	No concerns	Very low
LIT vs RIS	1	Some concerns	Suspected	No concerns	Major concerns	No concerns	No concerns	Very low
LIT vs TOP	2	No concerns	Suspected	No concerns	Major concerns	No concerns	No concerns	Low
LIT vs VALP	3	Some concerns	Suspected	No concerns	Major concerns	No concerns	No concerns	Very low
LIT vs PLA	7	Some concerns	Suspected	No concerns	Major concerns	No concerns	No concerns	Very low
OLA vs RIS	1	Some concerns	Suspected	No concerns	Major concerns	No concerns	No concerns	Very low
OLA vs VALP	4	Some concerns	Suspected	No concerns	No concerns	Major concerns	No concerns	Very low
OLA vs PLA	6	Some concerns	Suspected	No concerns	No concerns	No concerns	No concerns	Low
PAL vs QUE	1	No concerns	Suspected	No concerns	Major concerns	No concerns	No concerns	Low
PAL vs PLA	2	No concerns	Suspected	No concerns	Major concerns	No concerns	No concerns	Low
QUE vs PLA	4	No concerns	Suspected	No concerns	No concerns	Major concerns	No concerns	Low
RIS vs VALN	1	No concerns	Suspected	No concerns	No concerns	No concerns	No concerns	Moderate
RIS vs PLA	4	Some concerns	Suspected	No concerns	No concerns	Major concerns	Major concerns	Very low
TAM vs PLA	2	No concerns	Suspected	No concerns	Major concerns	No concerns	Major concerns	Very low
TOP vs PLA	4	No concerns	Suspected	No concerns	No concerns	Major concerns	No concerns	Low
VALN vs PLA	1	No concerns	Suspected	No concerns	Major concerns	No concerns	No concerns	Low
VALP vs PLA	5	Some concerns	Suspected	No concerns	Major concerns	No concerns	No concerns	Very low
VER vs PLA	1	No concerns	Suspected	No concerns	Major concerns	No concerns	Major concerns	Very low
ZIP vs PLA	3	Some concerns	Suspected	No concerns	Major concerns	No concerns	No concerns	Very low
ARI vs ASE	0	Some concerns	Suspected	No concerns	Major concerns	No concerns	Major concerns	Very low
ARI vs BRE	0	Some concerns	Suspected	No concerns	Major concerns	No concerns	Major concerns	Very low
ARI vs CARB	0	Some concerns	Suspected	No concerns	Major concerns	No concerns	Major concerns	Very low
ARI vs CARI	0	Some concerns	Suspected	No concerns	Major concerns	No concerns	Major concerns	Very low

ARI vs CHL	0	Some concerns	Suspected	No concerns	Major concerns	No concerns	Major concerns	Very low
ARI vs END	0	Some concerns	Suspected	No concerns	Major concerns	No concerns	Major concerns	Very low
ARI vs ESL	0	Some concerns	Suspected	No concerns	Major concerns	No concerns	Major concerns	Very low
ARI vs LAM	0	Some concerns	Suspected	No concerns	Major concerns	No concerns	Major concerns	Very low
ARI vs LIC	0	Some concerns	Suspected	No concerns	Major concerns	No concerns	Major concerns	Very low
ARI vs OLA	0	Some concerns	Suspected	No concerns	No concerns	Major concerns	Major concerns	Very low
ARI vs PAL	0	Some concerns	Suspected	No concerns	Major concerns	No concerns	Major concerns	Very low
ARI vs QUE	0	Some concerns	Suspected	No concerns	Major concerns	No concerns	Major concerns	Very low
ARI vs TAM	0	Some concerns	Suspected	No concerns	Major concerns	No concerns	Major concerns	Very low
ARI vs TOP	0	Some concerns	Suspected	No concerns	No concerns	No concerns	Major concerns	Very low
ARI vs VALN	0	Some concerns	Suspected	No concerns	No concerns	No concerns	Major concerns	Very low
ARI vs VALP	0	Some concerns	Suspected	No concerns	Major concerns	No concerns	Major concerns	Very low
ARI vs VER	0	Some concerns	Suspected	No concerns	Major concerns	No concerns	Major concerns	Very low
ARI vs ZIP	0	Some concerns	Suspected	No concerns	Major concerns	No concerns	Major concerns	Very low
ASE vs BRE	0	Some concerns	Suspected	No concerns	Major concerns	No concerns	Major concerns	Very low
ASE vs CARB	0	Some concerns	Suspected	No concerns	Major concerns	No concerns	Major concerns	Very low
ASE vs CARI	0	Some concerns	Suspected	No concerns	Major concerns	No concerns	Major concerns	Very low
ASE vs CHL	0	Some concerns	Suspected	No concerns	Major concerns	No concerns	Major concerns	Very low
ASE vs END	0	No concerns	Suspected	No concerns	Major concerns	No concerns	Major concerns	Very low
ASE vs ESL	0	Some concerns	Suspected	No concerns	Major concerns	No concerns	Major concerns	Very low
ASE vs HAL	0	Some concerns	Suspected	No concerns	Major concerns	No concerns	Major concerns	Very low
ASE vs LAM	0	Some concerns	Suspected	No concerns	Major concerns	No concerns	Major concerns	Very low
ASE vs LIC	0	Some concerns	Suspected	No concerns	Major concerns	No concerns	Major concerns	Very low
ASE vs LIT	0	Some concerns	Suspected	No concerns	Major concerns	No concerns	Major concerns	Very low
ASE vs PAL	0	No concerns	Suspected	No concerns	Major concerns	No concerns	Major concerns	Very low

ASE vs QUE	0	Some concerns	Suspected	No concerns	Major concerns	No concerns	Major concerns	Very low
ASE vs RIS	0	Some concerns	Suspected	No concerns	Major concerns	No concerns	Major concerns	Very low
ASE vs TAM	0	No concerns	Suspected	No concerns	Major concerns	No concerns	Major concerns	Very low
ASE vs TOP	0	No concerns	Suspected	No concerns	Major concerns	No concerns	Major concerns	Very low
ASE vs VALN	0	No concerns	Suspected	No concerns	Major concerns	No concerns	Major concerns	Very low
ASE vs VALP	0	Some concerns	Suspected	No concerns	Major concerns	No concerns	Major concerns	Very low
ASE vs VER	0	No concerns	Suspected	No concerns	Major concerns	No concerns	Major concerns	Very low
ASE vs ZIP	0	Some concerns	Suspected	No concerns	Major concerns	No concerns	Major concerns	Very low
BRE vs CARB	0	Some concerns	Suspected	No concerns	Major concerns	No concerns	Major concerns	Very low
BRE vs CARI	0	Some concerns	Suspected	No concerns	Major concerns	No concerns	Major concerns	Very low
BRE vs CHL	0	Some concerns	Suspected	No concerns	Major concerns	No concerns	Major concerns	Very low
BRE vs END	0	Some concerns	Suspected	No concerns	Major concerns	No concerns	Major concerns	Very low
BRE vs ESL	0	Some concerns	Suspected	No concerns	Major concerns	No concerns	Major concerns	Very low
BRE vs HAL	0	Some concerns	Suspected	No concerns	Major concerns	No concerns	Major concerns	Very low
BRE vs LAM	0	Some concerns	Suspected	No concerns	Major concerns	No concerns	Major concerns	Very low
BRE vs LIC	0	Some concerns	Suspected	No concerns	Major concerns	No concerns	Major concerns	Very low
BRE vs LIT	0	Some concerns	Suspected	No concerns	Major concerns	No concerns	Major concerns	Very low
BRE vs OLA	0	Some concerns	Suspected	No concerns	No concerns	Major concerns	Major concerns	Very low
BRE vs PAL	0	No concerns	Suspected	No concerns	Major concerns	No concerns	Major concerns	Very low
BRE vs QUE	0	Some concerns	Suspected	No concerns	Major concerns	No concerns	Major concerns	Very low
BRE vs RIS	0	Some concerns	Suspected	No concerns	Major concerns	No concerns	Major concerns	Very low
BRE vs TAM	0	Some concerns	Suspected	No concerns	Major concerns	No concerns	Major concerns	Very low
BRE vs TOP	0	Some concerns	Suspected	No concerns	Major concerns	No concerns	Major concerns	Very low
BRE vs VALN	0	Some concerns	Suspected	No concerns	Major concerns	No concerns	Major concerns	Very low
BRE vs VALP	0	Some concerns	Suspected	No concerns	Major concerns	No concerns	Major concerns	Very low

BRE vs VER	0	Some concerns	Suspected	No concerns	Major concerns	No concerns	Major concerns	Very low
BRE vs ZIP	0	Some concerns	Suspected	No concerns	Major concerns	No concerns	Major concerns	Very low
CARB vs CARI	0	Some concerns	Suspected	No concerns	Major concerns	No concerns	Major concerns	Very low
CARB vs CHL	0	Some concerns	Suspected	No concerns	Major concerns	No concerns	Major concerns	Very low
CARB vs END	0	Some concerns	Suspected	No concerns	Major concerns	No concerns	Major concerns	Very low
CARB vs ESL	0	Some concerns	Suspected	No concerns	Major concerns	No concerns	Major concerns	Very low
CARB vs HAL	0	Some concerns	Suspected	No concerns	Major concerns	No concerns	Major concerns	Very low
CARB vs LAM	0	Some concerns	Suspected	No concerns	Major concerns	No concerns	Major concerns	Very low
CARB vs LIC	0	Some concerns	Suspected	No concerns	Major concerns	No concerns	Major concerns	Very low
CARB vs OLA	0	Some concerns	Suspected	No concerns	Major concerns	No concerns	Major concerns	Very low
CARB vs PAL	0	No concerns	Suspected	No concerns	Major concerns	No concerns	Major concerns	Very low
CARB vs QUE	0	Some concerns	Suspected	No concerns	Major concerns	No concerns	Major concerns	Very low
CARB vs RIS	0	Some concerns	Suspected	No concerns	Major concerns	No concerns	Major concerns	Very low
CARB vs TAM	0	Some concerns	Suspected	No concerns	Major concerns	No concerns	Major concerns	Very low
CARB vs TOP	0	Some concerns	Suspected	No concerns	No concerns	Major concerns	Major concerns	Very low
CARB vs VALN	0	Some concerns	Suspected	No concerns	No concerns	Major concerns	Major concerns	Very low
CARB vs VER	0	Some concerns	Suspected	No concerns	Major concerns	No concerns	Major concerns	Very low
CARB vs ZIP	0	Some concerns	Suspected	No concerns	Major concerns	No concerns	Major concerns	Very low
CARI vs CHL	0	Some concerns	Suspected	No concerns	Major concerns	No concerns	Major concerns	Very low
CARI vs END	0	Some concerns	Suspected	No concerns	Major concerns	No concerns	Major concerns	Very low
CARI vs ESL	0	Some concerns	Suspected	No concerns	Major concerns	No concerns	Major concerns	Very low
CARI vs HAL	0	Some concerns	Suspected	No concerns	Major concerns	No concerns	Major concerns	Very low
CARI vs LAM	0	Some concerns	Suspected	No concerns	Major concerns	No concerns	Major concerns	Very low
CARI vs LIC	0	Some concerns	Suspected	No concerns	Major concerns	No concerns	Major concerns	Very low
CARI vs LIT	0	Some concerns	Suspected	No concerns	Major concerns	No concerns	Major concerns	Very low

CARI vs OLA	0	Some concerns	Suspected	No concerns	No concerns	Major concerns	Major concerns	Very low
CARI vs PAL	0	No concerns	Suspected	No concerns	Major concerns	No concerns	Major concerns	Very low
CARI vs QUE	0	Some concerns	Suspected	No concerns	Major concerns	No concerns	Major concerns	Very low
CARI vs RIS	0	Some concerns	Suspected	No concerns	Major concerns	No concerns	Major concerns	Very low
CARI vs TAM	0	Some concerns	Suspected	No concerns	Major concerns	No concerns	Major concerns	Very low
CARI vs TOP	0	Some concerns	Suspected	No concerns	Major concerns	No concerns	Major concerns	Very low
CARI vs VALN	0	Some concerns	Suspected	No concerns	Major concerns	No concerns	Major concerns	Very low
CARI vs VALP	0	Some concerns	Suspected	No concerns	Major concerns	No concerns	Major concerns	Very low
CARI vs VER	0	Some concerns	Suspected	No concerns	Major concerns	No concerns	Major concerns	Very low
CARI vs ZIP	0	Some concerns	Suspected	No concerns	Major concerns	No concerns	Major concerns	Very low
CHL vs END	0	Some concerns	Suspected	No concerns	Major concerns	No concerns	Major concerns	Very low
CHL vs ESL	0	Some concerns	Suspected	No concerns	Major concerns	No concerns	Major concerns	Very low
CHL vs LAM	0	Some concerns	Suspected	No concerns	Major concerns	No concerns	Major concerns	Very low
CHL vs LIC	0	Some concerns	Suspected	No concerns	Major concerns	No concerns	Major concerns	Very low
CHL vs LIT	0	Some concerns	Suspected	No concerns	Major concerns	No concerns	Major concerns	Very low
CHL vs OLA	0	Some concerns	Suspected	No concerns	Major concerns	No concerns	Major concerns	Very low
CHL vs PAL	0	Some concerns	Suspected	No concerns	Major concerns	No concerns	Major concerns	Very low
CHL vs QUE	0	Some concerns	Suspected	No concerns	Major concerns	No concerns	Major concerns	Very low
CHL vs RIS	0	Some concerns	Suspected	No concerns	Major concerns	No concerns	Major concerns	Very low
CHL vs TAM	0	Some concerns	Suspected	No concerns	Major concerns	No concerns	Major concerns	Very low
CHL vs TOP	0	Some concerns	Suspected	No concerns	Major concerns	No concerns	Major concerns	Very low
CHL vs VALN	0	Some concerns	Suspected	No concerns	Major concerns	No concerns	Major concerns	Very low
CHL vs VALP	0	Some concerns	Suspected	No concerns	Major concerns	No concerns	Major concerns	Very low
CHL vs VER	0	Some concerns	Suspected	No concerns	Major concerns	No concerns	Major concerns	Very low
CHL vs ZIP	0	Some concerns	Suspected	No concerns	Major concerns	No concerns	Major concerns	Very low

CHL vs PLA	0	Some concerns	Suspected	No concerns	Major concerns	No concerns	Major concerns	Very low
END vs ESL	0	Some concerns	Suspected	No concerns	Major concerns	No concerns	Major concerns	Very low
END vs HAL	0	No concerns	Suspected	No concerns	Major concerns	No concerns	Major concerns	Very low
END vs LAM	0	Some concerns	Suspected	No concerns	Major concerns	No concerns	Major concerns	Very low
END vs LIC	0	Some concerns	Suspected	No concerns	Major concerns	No concerns	Major concerns	Very low
END vs LIT	0	No concerns	Suspected	No concerns	Major concerns	No concerns	Major concerns	Very low
END vs OLA	0	No concerns	Suspected	No concerns	Major concerns	No concerns	Major concerns	Very low
END vs PAL	0	No concerns	Suspected	No concerns	Major concerns	No concerns	Major concerns	Very low
END vs QUE	0	No concerns	Suspected	No concerns	Major concerns	No concerns	Major concerns	Very low
END vs RIS	0	No concerns	Suspected	No concerns	Major concerns	No concerns	Major concerns	Very low
END vs TAM	0	No concerns	Suspected	No concerns	Major concerns	No concerns	Major concerns	Very low
END vs TOP	0	No concerns	Suspected	No concerns	Major concerns	No concerns	Major concerns	Very low
END vs VALN	0	No concerns	Suspected	No concerns	Major concerns	No concerns	Major concerns	Very low
END vs VER	0	No concerns	Suspected	No concerns	Major concerns	No concerns	Major concerns	Very low
END vs ZIP	0	No concerns	Suspected	No concerns	Major concerns	No concerns	Major concerns	Very low
END vs PLA	0	No concerns	Suspected	No concerns	Major concerns	No concerns	Major concerns	Very low
ESL vs HAL	0	Some concerns	Suspected	No concerns	Major concerns	No concerns	Major concerns	Very low
ESL vs LAM	0	Some concerns	Suspected	No concerns	Major concerns	No concerns	Major concerns	Very low
ESL vs LIC	0	Some concerns	Suspected	No concerns	Major concerns	No concerns	Major concerns	Very low
ESL vs LIT	0	Some concerns	Suspected	No concerns	Major concerns	No concerns	Major concerns	Very low
ESL vs OLA	0	Some concerns	Suspected	No concerns	Major concerns	No concerns	Major concerns	Very low
ESL vs PAL	0	No concerns	Suspected	No concerns	Major concerns	No concerns	Major concerns	Very low
ESL vs QUE	0	Some concerns	Suspected	No concerns	Major concerns	No concerns	Major concerns	Very low
ESL vs RIS	0	Some concerns	Suspected	No concerns	Major concerns	No concerns	Major concerns	Very low
ESL vs TAM	0	Some concerns	Suspected	No concerns	Major concerns	No concerns	Major concerns	Very low

ESL vs TOP	0	Some concerns	Suspected	No concerns	Major concerns	No concerns	Major concerns	Very low
ESL vs VALN	0	Some concerns	Suspected	No concerns	Major concerns	No concerns	Major concerns	Very low
ESL vs VALP	0	Some concerns	Suspected	No concerns	Major concerns	No concerns	Major concerns	Very low
ESL vs VER	0	Some concerns	Suspected	No concerns	Major concerns	No concerns	Major concerns	Very low
ESL vs ZIP	0	Some concerns	Suspected	No concerns	Major concerns	No concerns	Major concerns	Very low
HAL vs LAM	0	Some concerns	Suspected	No concerns	Major concerns	No concerns	Major concerns	Very low
HAL vs LIC	0	Some concerns	Suspected	No concerns	Major concerns	No concerns	Major concerns	Very low
HAL vs PAL	0	Some concerns	Suspected	No concerns	Major concerns	No concerns	Major concerns	Very low
HAL vs TAM	0	Some concerns	Suspected	No concerns	Major concerns	No concerns	Major concerns	Very low
HAL vs TOP	0	Some concerns	Suspected	No concerns	No concerns	Major concerns	Major concerns	Very low
HAL vs VALN	0	Some concerns	Suspected	No concerns	No concerns	Major concerns	Major concerns	Very low
HAL vs VALP	0	Some concerns	Suspected	No concerns	Major concerns	No concerns	Major concerns	Very low
HAL vs VER	0	Some concerns	Suspected	No concerns	Major concerns	No concerns	Major concerns	Very low
LAM vs LIC	0	Some concerns	Suspected	No concerns	Major concerns	No concerns	Major concerns	Very low
LAM vs PAL	0	Some concerns	Suspected	No concerns	Major concerns	No concerns	Major concerns	Very low
LAM vs QUE	0	Some concerns	Suspected	No concerns	Major concerns	No concerns	Major concerns	Very low
LAM vs RIS	0	Some concerns	Suspected	No concerns	Major concerns	No concerns	Major concerns	Very low
LAM vs TAM	0	Some concerns	Suspected	No concerns	Major concerns	No concerns	Major concerns	Very low
LAM vs TOP	0	Some concerns	Suspected	No concerns	Major concerns	No concerns	Major concerns	Very low
LAM vs VALN	0	Some concerns	Suspected	No concerns	Major concerns	No concerns	Major concerns	Very low
LAM vs VALP	0	Some concerns	Suspected	No concerns	Major concerns	No concerns	Major concerns	Very low
LAM vs VER	0	Some concerns	Suspected	No concerns	Major concerns	No concerns	Major concerns	Very low
LAM vs ZIP	0	Some concerns	Suspected	No concerns	Major concerns	No concerns	Major concerns	Very low
LIC vs LIT	0	Some concerns	Suspected	No concerns	Major concerns	No concerns	Major concerns	Very low
LIC vs OLA	0	Some concerns	Suspected	No concerns	No concerns	No concerns	Major concerns	Very low

LIC vs PAL	0	No concerns	Suspected	No concerns	Major concerns	No concerns	Major concerns	Very low
LIC vs QUE	0	Some concerns	Suspected	No concerns	Major concerns	No concerns	Major concerns	Very low
LIC vs RIS	0	Some concerns	Suspected	No concerns	Major concerns	No concerns	Major concerns	Very low
LIC vs TAM	0	Some concerns	Suspected	No concerns	Major concerns	No concerns	Major concerns	Very low
LIC vs TOP	0	Some concerns	Suspected	No concerns	Major concerns	No concerns	Major concerns	Very low
LIC vs VALN	0	Some concerns	Suspected	No concerns	Major concerns	No concerns	Major concerns	Very low
LIC vs VALP	0	Some concerns	Suspected	No concerns	Major concerns	No concerns	Major concerns	Very low
LIC vs VER	0	Some concerns	Suspected	No concerns	Major concerns	No concerns	Major concerns	Very low
LIC vs ZIP	0	Some concerns	Suspected	No concerns	Major concerns	No concerns	Major concerns	Very low
LIT vs PAL	0	No concerns	Suspected	No concerns	Major concerns	No concerns	Major concerns	Very low
LIT vs TAM	0	No concerns	Suspected	No concerns	Major concerns	No concerns	Major concerns	Very low
LIT vs VALN	0	No concerns	Suspected	No concerns	Major concerns	No concerns	Major concerns	Very low
LIT vs VER	0	No concerns	Suspected	No concerns	Major concerns	No concerns	Major concerns	Very low
LIT vs ZIP	0	Some concerns	Suspected	No concerns	Major concerns	No concerns	Major concerns	Very low
OLA vs PAL	0	No concerns	Suspected	No concerns	Major concerns	No concerns	Major concerns	Very low
OLA vs QUE	0	Some concerns	Suspected	No concerns	Major concerns	No concerns	Major concerns	Very low
OLA vs TAM	0	No concerns	Suspected	No concerns	Major concerns	No concerns	Major concerns	Very low
OLA vs TOP	0	No concerns	Suspected	No concerns	No concerns	No concerns	Major concerns	Very low
OLA vs VALN	0	No concerns	Suspected	No concerns	No concerns	No concerns	Major concerns	Very low
OLA vs VER	0	No concerns	Suspected	No concerns	No concerns	No concerns	Major concerns	Very low
OLA vs ZIP	0	Some concerns	Suspected	No concerns	No concerns	Major concerns	Major concerns	Very low
PAL vs RIS	0	No concerns	Suspected	No concerns	Major concerns	No concerns	Major concerns	Very low
PAL vs TAM	0	No concerns	Suspected	No concerns	Major concerns	No concerns	Major concerns	Very low
PAL vs TOP	0	No concerns	Suspected	No concerns	No concerns	No concerns	Major concerns	Very low
PAL vs VALN	0	No concerns	Suspected	No concerns	No concerns	No concerns	Major concerns	Very low

PAL vs VALP	0	No concerns	Suspected	No concerns	Major concerns	No concerns	Major concerns	Very low
PAL vs VER	0	No concerns	Suspected	No concerns	No concerns	Major concerns	Major concerns	Very low
PAL vs ZIP	0	No concerns	Suspected	No concerns	Major concerns	No concerns	Major concerns	Very low
QUE vs RIS	0	Some concerns	Suspected	No concerns	Major concerns	No concerns	Major concerns	Very low
QUE vs TAM	0	No concerns	Suspected	No concerns	Major concerns	No concerns	Major concerns	Very low
QUE vs TOP	0	No concerns	Suspected	No concerns	No concerns	No concerns	Major concerns	Very low
QUE vs VALN	0	No concerns	Suspected	No concerns	No concerns	No concerns	Major concerns	Very low
QUE vs VALP	0	No concerns	Suspected	No concerns	Major concerns	No concerns	Major concerns	Very low
QUE vs VER	0	No concerns	Suspected	No concerns	No concerns	Major concerns	Major concerns	Very low
QUE vs ZIP	0	Some concerns	Suspected	No concerns	Major concerns	No concerns	Major concerns	Very low
RIS vs TAM	0	Some concerns	Suspected	No concerns	Major concerns	No concerns	Major concerns	Very low
RIS vs TOP	0	Some concerns	Suspected	No concerns	No concerns	No concerns	Major concerns	Very low
RIS vs VALP	0	Some concerns	Suspected	No concerns	Major concerns	No concerns	Major concerns	Very low
RIS vs VER	0	Some concerns	Suspected	No concerns	No concerns	Major concerns	Major concerns	Very low
RIS vs ZIP	0	Some concerns	Suspected	No concerns	Major concerns	No concerns	Major concerns	Very low
TAM vs TOP	0	No concerns	Suspected	No concerns	Major concerns	No concerns	Major concerns	Very low
TAM vs VALN	0	No concerns	Suspected	No concerns	Major concerns	No concerns	Major concerns	Very low
TAM vs VALP	0	No concerns	Suspected	No concerns	Major concerns	No concerns	Major concerns	Very low
TAM vs VER	0	No concerns	Suspected	No concerns	Major concerns	No concerns	Major concerns	Very low
TAM vs ZIP	0	No concerns	Suspected	No concerns	Major concerns	No concerns	Major concerns	Very low
TOP vs VALN	0	No concerns	Suspected	No concerns	Major concerns	No concerns	Major concerns	Very low
TOP vs VALP	0	No concerns	Suspected	No concerns	No concerns	Major concerns	Major concerns	Very low
TOP vs VER	0	No concerns	Suspected	No concerns	Major concerns	No concerns	Major concerns	Very low
TOP vs ZIP	0	No concerns	Suspected	No concerns	No concerns	Major concerns	Major concerns	Very low
VALN vs VALP	0	No concerns	Suspected	No concerns	No concerns	No concerns	Major concerns	Very low

VALN vs VER	0	No concerns	Suspected	No concerns	Major concerns	No concerns	Major concerns	Very low
VALN vs ZIP	0	No concerns	Suspected	No concerns	No concerns	Major concerns	Major concerns	Very low
VALP vs VER	0	No concerns	Suspected	No concerns	Major concerns	No concerns	Major concerns	Very low
VALP vs ZIP	0	Some concerns	Suspected	No concerns	Major concerns	No concerns	Major concerns	Very low
VER vs ZIP	0	No concerns	Suspected	No concerns	Major concerns	No concerns	Major concerns	Very low

Supplementary Appendix 3. Mania rating scale scores (N = 61, n = 15466)

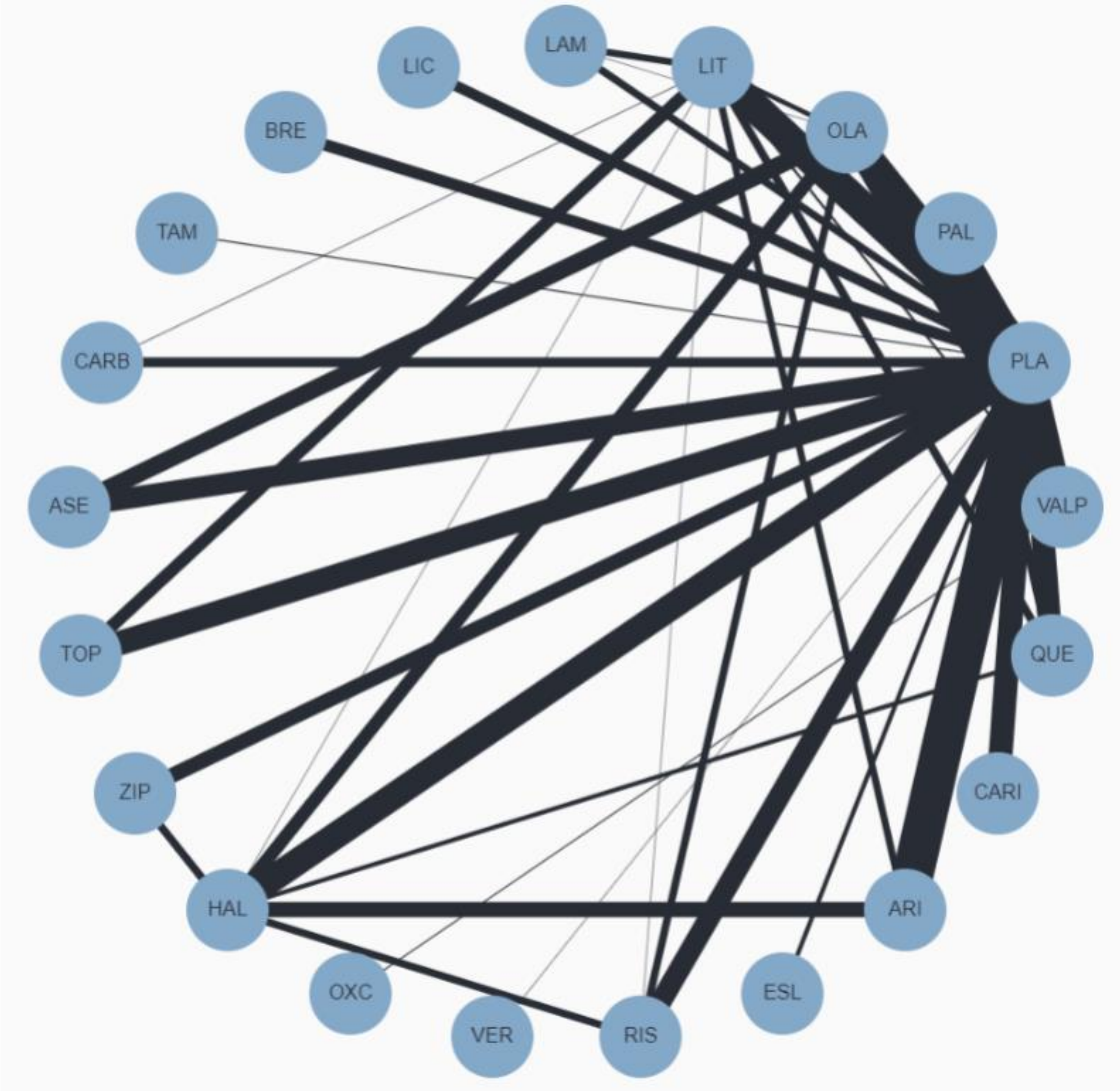

League table (standardized mean difference with 95% confidence interval)

ARI	-0.048 (-0.286, 0.191)	-0.274 (-0.576, 0.028)	0.222 (-0.074, 0.518)	0.139 (-0.115, 0.392)	-0.190 (-0.610, 0.230)	**0.247 (0.075, 0.419)**	-0.257 (-0.543, 0.030)	-0.269 (-0.644, 0.106)	-0.009 (-0.188, 0.170)	0.133 (-0.046, 0.312)	-0.017 (-0.636, 0.603)	0.018 (-0.260, 0.297)	0.018 (-0.194, 0.230)	**0.240 (0.026, 0.453)**	**1.447 (0.785, 2.110)**	**-0.454 (-0.679, -0.229)**	-0.143 (-0.348, 0.062)	-0.375 (-1.141, 0.390)	-0.021 (-0.276, 0.235)	**-0.359 (-0.497, -0.220)**
	ASE	-0.226 (-0.559, 0.106)	0.270 (-0.058, 0.597)	0.186 (-0.103, 0.476)	-0.142 (-0.584, 0.301)	**0.295 (0.059, 0.530)**	-0.209 (-0.527, 0.109)	-0.221 (-0.621, 0.179)	0.039 (-0.190, 0.269)	0.181 (-0.021, 0.384)	0.031 (-0.601, 0.662)	0.066 (-0.246, 0.378)	0.066 (-0.190, 0.322)	**0.287 (0.034, 0.540)**	**1.495 (0.818, 2.172)**	**-0.406 (-0.671, -0.141)**	-0.095 (-0.334, 0.144)	-0.328 (-1.106, 0.451)	0.027 (-0.266, 0.321)	**-0.311 (-0.507, -0.115)**
		BRE	**0.496 (0.120, 0.872)**	**0.413 (0.070, 0.756)**	0.085 (-0.394, 0.564)	**0.521 (0.218, 0.824)**	0.018 (-0.352, 0.387)	0.005 (-0.435, 0.446)	0.266 (-0.032, 0.563)	**0.408 (0.112, 0.703)**	0.257 (-0.405, 0.919)	0.293 (-0.070, 0.655)	0.292 (-0.024, 0.609)	**0.514 (0.196, 0.831)**	**1.722 (1.020, 2.423)**	-0.180 (-0.503, 0.144)	0.131 (-0.179, 0.442)	-0.101 (-0.901, 0.699)	0.254 (-0.094, 0.601)	-0.085 (-0.354, 0.184)
			CARB	-0.083 (-0.421, 0.255)	-0.411 (-0.887, 0.064)	0.025 (-0.272, 0.323)	**-0.478 (-0.841, -0.116)**	**-0.491 (-0.928, -0.054)**	-0.230 (-0.515, 0.054)	-0.088 (-0.377, 0.200)	-0.239 (-0.898, 0.420)	-0.203 (-0.561, 0.154)	-0.204 (-0.513, 0.106)	0.018 (-0.294, 0.330)	**1.226 (0.526, 1.925)**	**-0.676 (-0.992, -0.359)**	**-0.364 (-0.669, -0.060)**	-0.597 (-1.395, 0.201)	-0.242 (-0.585, 0.100)	**-0.581 (-0.844, -0.318)**
				CARI	-0.328 (-0.778, 0.122)	0.108 (-0.146, 0.363)	**-0.395 (-0.726, -0.064)**	-0.408 (-0.816, 0.001)	-0.147 (-0.396, 0.101)	-0.005 (-0.250, 0.240)	-0.156 (-0.797, 0.486)	-0.120 (-0.443, 0.203)	-0.121 (-0.391, 0.150)	0.101 (-0.171, 0.373)	**1.309 (0.627, 1.991)**	**-0.592 (-0.871, -0.314)**	**-0.281 (-0.545, -0.018)**	-0.514 (-1.296, 0.269)	-0.159 (-0.465, 0.147)	**-0.498 (-0.710, -0.285)**
					ESL	**0.437 (0.016, 0.857)**	-0.067 (-0.538, 0.404)	-0.079 (-0.608, 0.449)	0.181 (-0.236, 0.598)	0.323 (-0.092, 0.738)	0.173 (-0.551, 0.896)	0.208 (-0.257, 0.673)	0.208 (-0.223, 0.638)	0.429 (-0.002, 0.860)	**1.637 (0.877, 2.397)**	-0.264 (-0.700, 0.172)	0.047 (-0.379, 0.473)	-0.186 (-1.037, 0.666)	0.169 (-0.285, 0.623)	-0.169 (-0.566, 0.227)
						HAL	**-0.504 (-0.791, -0.217)**	**-0.516 (-0.892, -0.140)**	**-0.256 (-0.438, -0.073)**	-0.114 (-0.281, 0.053)	-0.264 (-0.882, 0.354)	-0.228 (-0.506, 0.049)	**-0.229 (-0.435, -0.023)**	-0.007 (-0.208, 0.193)	**1.200 (0.537, 1.863)**	**-0.701 (-0.928, -0.474)**	**-0.390 (-0.592, -0.188)**	-0.622 (-1.388, 0.144)	**-0.268 (-0.508, -0.027)**	**-0.606 (-0.747, -0.465)**
							LAM	-0.012 (-0.444, 0.419)	0.248 (-0.009, 0.505)	**0.390 (0.116, 0.664)**	0.240 (-0.414, 0.893)	0.275 (-0.074, 0.625)	0.275 (-0.023, 0.572)	**0.496 (0.194, 0.798)**	**1.704 (1.008, 2.400)**	-0.197 (-0.502, 0.107)	0.114 (-0.179, 0.406)	-0.119 (-0.913, 0.676)	0.236 (-0.099, 0.571)	-0.102 (-0.356, 0.151)

								LIC	0.260 (-0.111, 0.632)	**0.402 (0.033, 0.772)**	0.252 (-0.446, 0.950)	0.287 (-0.138, 0.712)	0.287 (-0.100, 0.674)	**0.508 (0.121, 0.896)**	**1.716 (0.980, 2.452)**	-0.185 (-0.578, 0.208)	0.126 (-0.256, 0.508)	-0.106 (-0.936, 0.724)	0.248 (-0.164, 0.661)	-0.090 (-0.439, 0.259)
									LIT	0.142 (-0.020, 0.304)	-0.008 (-0.623, 0.606)	0.027 (-0.242, 0.297)	0.026 (-0.162, 0.215)	**0.248 (0.042, 0.454)**	**1.456 (0.796, 2.116)**	**-0.445 (-0.644, -0.247)**	-0.134 (-0.324, 0.055)	-0.367 (-1.131, 0.397)	-0.012 (-0.265, 0.241)	**-0.350 (-0.479, -0.222)**
										OLA	-0.150 (-0.756, 0.456)	-0.115 (-0.386, 0.156)	-0.115 (-0.317, 0.086)	0.106 (-0.084, 0.296)	**1.314 (0.655, 1.973)**	**-0.587 (-0.802, -0.373)**	**-0.276 (-0.436, -0.116)**	-0.509 (-1.272, 0.254)	-0.154 (-0.402, 0.094)	**-0.492 (-0.614, -0.370)**
											OXC	0.035 (-0.616, 0.687)	0.035 (-0.591, 0.661)	0.257 (-0.369, 0.882)	**1.464 (0.578, 2.351)**	-0.437 (-1.067, 0.193)	-0.126 (-0.710, 0.459)	-0.358 (-1.324, 0.608)	-0.004 (-0.647, 0.639)	-0.342 (-0.947, 0.263)
												PAL	0.000 (-0.263, 0.262)	0.221 (-0.074, 0.516)	**1.429 (0.737, 2.121)**	**-0.472 (-0.774, -0.171)**	-0.161 (-0.449, 0.126)	-0.394 (-1.185, 0.398)	-0.039 (-0.366, 0.288)	**-0.377 (-0.620, -0.134**)
													QUE	0.222 (-0.013, 0.456)	**1.429 (0.760, 2.099)**	**-0.472 (-0.712, -0.231)**	-0.161 (-0.385, 0.064)	-0.393 (-1.165, 0.378)	-0.039 (-0.312, 0.235)	**-0.377 (-0.544, -0.210)**
														RIS	**1.208 (0.538, 1.877)**	**-0.694 (-0.939, -0.448)**	**-0.382 (-0.604, -0.160)**	-0.615 (-1.387, 0.157)	-0.260 (-0.534, 0.013)	**-0.599 (-0.768, -0.430)**
															TAM	**-1.901 (-2.574, -1.229)**	**-1.590 (-2.256, -0.924)**	**-1.823 (-2.816, -0.829)**	**-1.468 (-2.152, -0.784)**	**-1.806 (-2.454, -1.159)**

																TOP	**0.311 (0.076, 0.546)**	0.079 (-0.696, 0.853)	**0.433 (0.149, 0.717)**	0.095 (-0.086, 0.275)
																	VALP	-0.232 (-1.002, 0.536)	0.122 (-0.146, 0.390)	**-0.216 (-0.371, -0.061)**
																		VER	0.355 (-0.430, 1.139)	0.016 (-0.737, 0.769)
																			ZIP	**-0.338 (-0.558, -0.118)**
																				PLA

The outcome was synthesized the following mania rating scale scores.

Young Mania Rating Scale,[1] Mania Rating Scale developed from the Schedule for Affective Disorders and Schizophrenia, Change Version,[2] and Manic-State Rating Scale[3]

1. Young RC, Biggs JT, Ziegler VE, Meyer DA. A rating scale for mania: reliability, validity and sensitivity. Br J Psychiatry 1978; 133: 429-435.
2. Endicott J, Spitzer RL. A diagnostic interview: the schedule for affective disorders and schizophrenia. Arch Gen Psychiatry 1978; 35(7): 837-844.
3. Beigel A, Murphy D, Bunney JW. The Manic-State Rating Scale: Scale Construction, Reliability, and Validity. Arch Gen Psychiatry 1971; 25(3): 256-262.

Study	Mania rating scale
Berk 1999	MRS
Berwaerts 2012	YMRS
Bowden 1994	MRS
Bowden 2000	MRS
Bowden 2005	YMRS

Bowden 2006	MRS
Calabrese 2015	YMRS
Cutler 2011	YMRS
Durgam 2015	YMRS
El Mallakh 2010	YMRS
Freeman 1992	MRS
Grunze 2015 (203)	YMRS
Grunze 2015 (204)	YMRS
Hirschfeld 2004	YMRS
Hirschfeld 2010	MRS
Janicak 1998	YMRS
Kakkar 2009	YMRS
Kanba 2014	YMRS
Katagiri 2012	YMRS
Keck 2003 ARI	YMRS
Keck 2003 ZIP	MRS
Keck 2009	YMRS
Khanna 2005	YMRS
Kushner 2006 (004)	YMRS
Kushner 2006 (005)	YMRS
Kushner 2006 (006)	YMRS
Kushner 2006 (008)	YMRS
Landbloom 2016	YMRS
Li 2008	YMRS
McIntyre 2005	YMRS

McIntyre 2009	YMRS
McIntyre 2010	YMRS
Niufan 2008	YMRS
Perlis 2006	YMRS
Pope 1991	YMRS
Potkin 2005	MRS
Sachs 2006	YMRS
Sachs 2015	YMRS
Segal 1998	YMRS
Shafti 2010	MSRS
Small 1991	YMRS
Smulevich 2005	YMRS
Tohen 1999	YMRS
Tohen 2000	YMRS
Tohen 2002	YMRS
Tohen 2003	YMRS
Tohen 2008	YMRS
Vieta 2005	YMRS
Vieta 2010 ZIP	MRS
Vieta 2010 PAL	YMRS
Weiser 2017	YMRS
Weisler 2004	YMRS
Weisler 2005	YMRS
Xu 2015	YMRS
Yildiz 2008	YMRS

Young 2009	YMRS
Zajecka 2002	MRS
Zarate 2007	YMRS
NCT03259555	YMRS
NCT03257865	YMRS
NCT00099229	YMRS

MRS: Mania Rating Scale (Schedule for Affective Disorders and Schizophrenia), MSRS: Manic State Rating Scale, YMRS: Young Mania Rating Scale

Evaluation of heterogeneity and inconsistency

Between study variance (τ^2)	Heterogeneity assessment	Random-effects design-by-treatment interaction model		
		Q	df	p
0.022	Low	36.73	35	0.389

Incoherence

	NMA, SMD (95% CI)	Direct, SMD (95% CI)	I^2	Indirect, SMD (95% CI)	Inconsistency measures	
					Difference of SMD	P value
ARI vs HAL	0.247 (0.075, 0.419)	0.034 (-0.222, 0.290)	0.0%	0.422 (0.190, 0.654)	-0.388 (-0.734, -0.042)	0.028
ARI vs LIT	-0.009 (-0.188, 0.170)	-0.060 (-0.427, 0.308)	na	0.007 (-0.198, 0.212)	-0.067 (-0.487, 0.354)	0.755
ARI vs PLA	-0.359 (-0.497, -0.220)	-0.280 (-0.432, -0.127)	45.2%	-0.732 (-1.063, -0.402)	0.453 (0.088, 0.817)	0.015
ASE vs OLA	0.181 (-0.021, 0.384)	0.229 (-0.022, 0.480	0.0%	0.091 (-0.252, 0.434	0.138 (-0.287, 0.563)	0.524
ASE vs PLA	-0.311 (-0.507, -0.115)	-0.365 (-0.582, -0.148)	28.3%	-0.072 (-0.530, 0.387)	-0.293 (-0.800, 0.214)	0.258
BRE vs PLA		-0.085 (-0.354, 0.184)	35.3%			
CARB vs LIT	-0.230 (-0.515, 0.054)	-0.105 (-0.790, 0.580)	na	-0.256 (-0.569, 0.056)	0.151 (-0.602, 0.905)	0.694
CARB vs PLA	-0.581 (-0.844, -0.318)	-0.602 (-0.886, -0.318)	84.5%	-0.451 (-1.149, 0.247)	-0.151 (-0.905, 0.602)	0.694
CARI vs PLA		-0.498 (-0.710, -0.285)	0.0%			
ESL vs PLA		-0.169 (-0.566, 0.227)	65.0%			
HAL vs LIT	-0.256 (-0.438, -0.073)	-1.784 (-2.696, -0.872)	na	-0.192 (-0.378, -0.006)	-1.592 (-2.522, -0.661)	0.001
HAL vs OLA	-0.114 (-0.281, 0.053)	-0.185 (-0.479, 0.110)	0.0%	-0.080 (-0.283, 0.123)	-0.105 (-0.462, 0.253)	0.566
HAL vs QUE	-0.229 (-0.435, -0.023)	-0.262 (-0.666, 0.142)	na	-0.217 (-0.458, 0.023)	-0.045 (-0.514, 0.425)	0.852
HAL vs RIS	-0.007 (-0.208, 0.193)	-0.094 (-0.430, 0.241)	72.4%	0.041 (-0.209, 0.291)	-0.135 (-0.554, 0.283)	0.526
HAL vs ZIP	-0.268 (-0.508, -0.027)	-0.516 (-0.878, -0.154)	na	-0.071 (-0.393, 0.251)	-0.445 (-0.929, 0.039)	0.072
HAL vs PLA	-0.606 (-0.747, -0.465)	-0.556 (-0.739, -0.373)	68.0%	-0.679 (-0.899, -0.458)	0.123 (-0.164, 0.409)	0.401
LAM vs LIT	0.248 (-0.009, 0.505)	0.168 (-0.131, 0.467)	0.0%	0.474 (-0.030, 0.979)	-0.306 (-0.892, 0.280)	0.306

LAM vs OLA	0.390 (0.116, 0.664)	0.125 (-0.649, 0.898)	na	0.428 (0.135, 0.721)	-0.303 (-1.130, 0.524)	0.473
LAM vs PLA	-0.102 (-0.356, 0.151)	0.002 (-0.297, 0.301)	0.0%	-0.371 (-0.852, 0.108)	0.373 (-0.192, 0.939)	0.195
LIC vs PLA		-0.090 (-0.439, 0.259)	na			
LIT vs OLA	0.142 (-0.020, 0.304)	0.070 (-0.270, 0.410)	84.6%	0.163 (-0.021, 0.347)	-0.093 (-0.479, 0.293)	0.638
LIT vs QUE	0.026 (-0.162, 0.215)	0.073 (-0.221, 0.366)	21.2%	-0.006 (-0.253, 0.240)	0.079 (-0.304, 0.462)	0.687
LIT vs RIS	0.248 (0.042, 0.454)	1.070 (0.245, 1.896)	na	0.194 (-0.019, 0.406)	0.877 (0.024, 1.729)	0.044
LIT vs TOP	-0.445 (-0.644, -0.247)	-0.529 (-0.799, -0.259)	0.0%	-0.347 (-0.640, -0.054)	-0.182 (-0.580, 0.217)	0.371
LIT vs VALP	-0.134 (-0.324, 0.055)	-0.286 (-0.741, 0.167)	77.8%	-0.102 (-0.310, 0.106)	-0.184 (-0.684, 0.315)	0.469
LIT vs PLA	-0.350 (-0.479, -0.222)	-0.377 (-0.536, -0.217)	71.5%	-0.302 (-0.519, -0.085)	-0.075 (-0.344, 0.194)	0.586
OLA vs RIS	0.106 (-0.084, 0.296)	-0.007 (-0.370, 0.356)	na	0.149 (-0.074, 0.372	-0.156 (-0.582, 0.270	0.472
OLA vs VALP	-0.276 (-0.436, -0.116)	-0.278 (-0.487, -0.068)	6.8%	-0.274 (-0.523, -0.025)	-0.004 (-0.330, 0.322)	0.981
OLA vs PLA	-0.492 (-0.614, -0.370)	-0.498 (-0.664, -0.332)	38.2%	-0.486 (-0.665, -0.306)	-0.012 (-0.257, 0.232)	0.921
OXC vs VALP		-0.126 (-0.710, 0.459)	na			
PAL vs QUE	0.000 (-0.263, 0.262)	-0.160 (-0.514, 0.195)	na	0.193 (-0.198, 0.584)	-0.353 (-0.880, 0.175)	0.190
PAL vs PLA	-0.377 (-0.620, -0.134)	-0.356 (-0.618, -0.094)	88.8%	-0.510 (-1.162, 0.141)	0.154 (-0.548, 0.857	0.667
QUE vs PLA	-0.377 (-0.544, -0.210)	-0.404 (-0.598, -0.210)	0.0%	-0.300 (-0.629, 0.029)	-0.104 (-0.486, 0.278)	0.595
RIS vs PLA	-0.599 (-0.768, -0.430)	-0.613 (-0.815, -0.411)	68.5%	-0.564 (-0.873, -0.255)	-0.049 (-0.418, 0.320)	0.794
TAM vs PLA		-1.806 (-2.454, -1.159)	na			
TOP vs PLA	0.095 (-0.086, 0.275)	0.064 (-0.129, 0.256)	0.0%	0.331 (-0.196, 0.859)	-0.268 (-0.829, 0.294)	0.350
VALP vs PLA	-0.216 (-0.371, -0.061)	-0.258 (-0.451, -0.066)	51.2%	-0.137 (-0.401, 0.126)	-0.121 (-0.447, 0.205)	0.466
VER vs PLA		0.016 (-0.737, 0.769)	na			
ZIP vs PLA	-0.338 (-0.558, -0.118)	-0.424 (-0.660, -0.188)	0.0%	0.234 (-0.376, 0.844)	-0.658 (-1.312, -0.004)	0.049

Funnel plot (only double-blind, placebo-controlled trials)

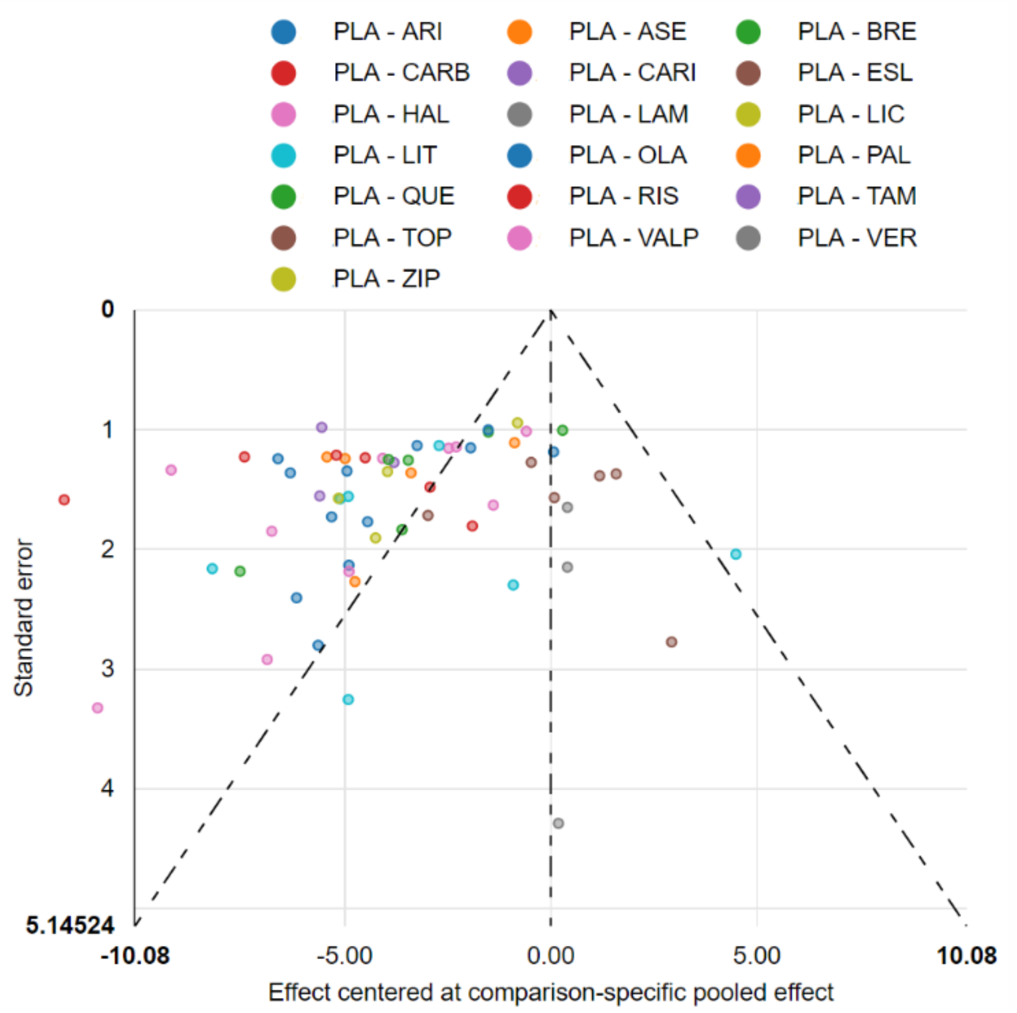

CINeMA confidence rating

Comparison	Number of studies	Within-study bias	Reporting bias	Indirectness	Imprecision	Heterogeneity	Incoherence	Confidence rating
ARI vs HAL	2	Some concerns	Suspected	No concerns	No concerns	Major concerns	Major concerns	Very low
ARI vs LIT	1	Some concerns	Suspected	No concerns	Major concerns	No concerns	No concerns	Very low
ARI vs PLA	6	Some concerns	Suspected	No concerns	No concerns	No concerns	No concerns	Low
ASE vs OLA	2	Some concerns	Suspected	No concerns	Major concerns	No concerns	No concerns	Very low
ASE vs PLA	3	Some concerns	Suspected	No concerns	No concerns	Major concerns	No concerns	Very low
BRE vs PLA	2	Some concerns	Suspected	No concerns	Major concerns	No concerns	No concerns	Very low
CARB vs LIT	1	Some concerns	Suspected	No concerns	Major concerns	No concerns	No concerns	Very low
CARB vs PLA	2	Some concerns	Suspected	No concerns	No concerns	No concerns	No concerns	Low
CARI vs PLA	3	Some concerns	Suspected	No concerns	No concerns	No concerns	No concerns	Low
ESL vs PLA	2	Some concerns	Suspected	No concerns	Major concerns	No concerns	No concerns	Very low
HAL vs LIT	1	Some concerns	Suspected	No concerns	No concerns	Major concerns	No concerns	Very low
HAL vs OLA	2	Some concerns	Suspected	No concerns	Major concerns	No concerns	No concerns	Very low
HAL vs QUE	1	Some concerns	Suspected	No concerns	No concerns	Major concerns	No concerns	Very low
HAL vs RIS	2	Some concerns	Suspected	No concerns	Major concerns	No concerns	No concerns	Very low
HAL vs ZIP	1	Some concerns	Suspected	No concerns	No concerns	Major concerns	Major concerns	Very low
HAL vs PLA	5	Some concerns	Suspected	No concerns	No concerns	No concerns	No concerns	Low
LAM vs LIT	3	Some concerns	Suspected	No concerns	Major concerns	No concerns	No concerns	Very low
LAM vs OLA	1	Some concerns	Suspected	No concerns	No concerns	Major concerns	No concerns	Very low
LAM vs PLA	2	Some concerns	Suspected	No concerns	Major concerns	No concerns	No concerns	Very low
LIC vs PLA	1	Some concerns	Suspected	No concerns	Major concerns	No concerns	No concerns	Very low
LIT vs OLA	3	Some concerns	Suspected	No concerns	Major concerns	No concerns	No concerns	Very low
LIT vs QUE	2	No concerns	Suspected	No concerns	Major concerns	No concerns	No concerns	Low
LIT vs RIS	1	Some concerns	Suspected	No concerns	No concerns	Major concerns	Major concerns	Very low

LIT vs TOP	2	No concerns	Suspected	No concerns	No concerns	No concerns	No concerns	Moderate
LIT vs VALP	2	Some concerns	Suspected	No concerns	Major concerns	No concerns	No concerns	Very low
LIT vs PLA	7	Some concerns	Suspected	No concerns	No concerns	No concerns	No concerns	Low
OLA vs RIS	1	Some concerns	Suspected	No concerns	Major concerns	No concerns	No concerns	Very low
OLA vs VALP	4	No concerns	Suspected	No concerns	No concerns	Major concerns	No concerns	Very low
OLA vs PLA	6	Some concerns	Suspected	No concerns	No concerns	No concerns	No concerns	Low
OXC vs VALP	1	Some concerns	Suspected	No concerns	Major concerns	No concerns	No concerns	Very low
PAL vs QUE	1	No concerns	Suspected	No concerns	Major concerns	No concerns	No concerns	Low
PAL vs PLA	2	No concerns	Suspected	No concerns	No concerns	Major concerns	No concerns	Low
QUE vs PLA	4	No concerns	Suspected	No concerns	No concerns	No concerns	No concerns	Moderate
RIS vs PLA	4	Some concerns	Suspected	No concerns	No concerns	No concerns	No concerns	Low
TAM vs PLA	1	No concerns	Suspected	No concerns	No concerns	No concerns	No concerns	Moderate
TOP vs PLA	4	No concerns	Suspected	No concerns	Major concerns	No concerns	No concerns	Low
VALP vs PLA	5	No concerns	Suspected	No concerns	No concerns	Major concerns	No concerns	Low
VER vs PLA	1	No concerns	Suspected	No concerns	Major concerns	No concerns	No concerns	Low
ZIP vs PLA	3	Some concerns	Suspected	No concerns	No concerns	Major concerns	Major concerns	Very low
ARI vs ASE	0	Some concerns	Suspected	No concerns	Major concerns	No concerns	No concerns	Very low
ARI vs BRE	0	Some concerns	Suspected	No concerns	Major concerns	No concerns	No concerns	Very low
ARI vs CARB	0	Some concerns	Suspected	No concerns	Major concerns	No concerns	No concerns	Very low
ARI vs CARI	0	Some concerns	Suspected	No concerns	Major concerns	No concerns	No concerns	Very low
ARI vs ESL	0	Some concerns	Suspected	No concerns	Major concerns	No concerns	No concerns	Very low
ARI vs LAM	0	Some concerns	Suspected	No concerns	Major concerns	No concerns	No concerns	Very low
ARI vs LIC	0	Some concerns	Suspected	No concerns	Major concerns	No concerns	No concerns	Very low
ARI vs OLA	0	Some concerns	Suspected	No concerns	Major concerns	No concerns	No concerns	Very low
ARI vs OXC	0	Some concerns	Suspected	No concerns	Major concerns	No concerns	No concerns	Very low

ARI vs PAL	0	Some concerns	Suspected	No concerns	Major concerns	No concerns	No concerns	Very low
ARI vs QUE	0	Some concerns	Suspected	No concerns	Major concerns	No concerns	No concerns	Very low
ARI vs RIS	0	Some concerns	Suspected	No concerns	No concerns	Major concerns	No concerns	Very low
ARI vs TAM	0	Some concerns	Suspected	No concerns	No concerns	No concerns	No concerns	Very low
ARI vs TOP	0	Some concerns	Suspected	No concerns	No concerns	No concerns	No concerns	Very low
ARI vs VALP	0	Some concerns	Suspected	No concerns	Major concerns	No concerns	No concerns	Very low
ARI vs VER	0	Some concerns	Suspected	No concerns	Major concerns	No concerns	No concerns	Very low
ARI vs ZIP	0	Some concerns	Suspected	No concerns	Major concerns	No concerns	No concerns	Very low
ASE vs BRE	0	Some concerns	Suspected	No concerns	Major concerns	No concerns	No concerns	Very low
ASE vs CARB	0	Some concerns	Suspected	No concerns	Major concerns	No concerns	No concerns	Very low
ASE vs CARI	0	Some concerns	Suspected	No concerns	Major concerns	No concerns	No concerns	Very low
ASE vs ESL	0	Some concerns	Suspected	No concerns	Major concerns	No concerns	No concerns	Very low
ASE vs HAL	0	Some concerns	Suspected	No concerns	No concerns	Major concerns	No concerns	Very low
ASE vs LAM	0	Some concerns	Suspected	No concerns	Major concerns	No concerns	No concerns	Very low
ASE vs LIC	0	Some concerns	Suspected	No concerns	Major concerns	No concerns	No concerns	Very low
ASE vs LIT	0	Some concerns	Suspected	No concerns	Major concerns	No concerns	No concerns	Very low
ASE vs OXC	0	Some concerns	Suspected	No concerns	Major concerns	No concerns	No concerns	Very low
ASE vs PAL	0	No concerns	Suspected	No concerns	Major concerns	No concerns	No concerns	Very low
ASE vs QUE	0	Some concerns	Suspected	No concerns	Major concerns	No concerns	No concerns	Very low
ASE vs RIS	0	Some concerns	Suspected	No concerns	No concerns	Major concerns	No concerns	Very low
ASE vs TAM	0	No concerns	Suspected	No concerns	No concerns	No concerns	No concerns	Low
ASE vs TOP	0	No concerns	Suspected	No concerns	No concerns	No concerns	No concerns	Low
ASE vs VALP	0	Some concerns	Suspected	No concerns	Major concerns	No concerns	No concerns	Very low
ASE vs VER	0	No concerns	Suspected	No concerns	Major concerns	No concerns	No concerns	Very low
ASE vs ZIP	0	Some concerns	Suspected	No concerns	Major concerns	No concerns	No concerns	Very low

BRE vs CARB	0	Some concerns	Suspected	No concerns	No concerns	No concerns	No concerns	Very low
BRE vs CARI	0	Some concerns	Suspected	No concerns	No concerns	Major concerns	No concerns	Very low
BRE vs ESL	0	Some concerns	Suspected	No concerns	Major concerns	No concerns	No concerns	Very low
BRE vs HAL	0	Some concerns	Suspected	No concerns	No concerns	No concerns	No concerns	Very low
BRE vs LAM	0	Some concerns	Suspected	No concerns	Major concerns	No concerns	No concerns	Very low
BRE vs LIC	0	Some concerns	Suspected	No concerns	Major concerns	No concerns	No concerns	Very low
BRE vs LIT	0	Some concerns	Suspected	No concerns	Major concerns	No concerns	No concerns	Very low
BRE vs OLA	0	Some concerns	Suspected	No concerns	No concerns	Major concerns	No concerns	Very low
BRE vs OXC	0	Some concerns	Suspected	No concerns	Major concerns	No concerns	No concerns	Very low
BRE vs PAL	0	No concerns	Suspected	No concerns	Major concerns	No concerns	No concerns	Very low
BRE vs QUE	0	Some concerns	Suspected	No concerns	Major concerns	No concerns	No concerns	Very low
BRE vs RIS	0	Some concerns	Suspected	No concerns	No concerns	No concerns	No concerns	Very low
BRE vs TAM	0	Some concerns	Suspected	No concerns	No concerns	No concerns	No concerns	Very low
BRE vs TOP	0	Some concerns	Suspected	No concerns	Major concerns	No concerns	No concerns	Very low
BRE vs VALP	0	Some concerns	Suspected	No concerns	Major concerns	No concerns	No concerns	Very low
BRE vs VER	0	Some concerns	Suspected	No concerns	Major concerns	No concerns	No concerns	Very low
BRE vs ZIP	0	Some concerns	Suspected	No concerns	Major concerns	No concerns	No concerns	Very low
CARB vs CARI	0	Some concerns	Suspected	No concerns	Major concerns	No concerns	No concerns	Very low
CARB vs ESL	0	Some concerns	Suspected	No concerns	Major concerns	No concerns	No concerns	Very low
CARB vs HAL	0	Some concerns	Suspected	No concerns	Major concerns	No concerns	No concerns	Very low
CARB vs LAM	0	Some concerns	Suspected	No concerns	No concerns	No concerns	No concerns	Very low
CARB vs LIC	0	Some concerns	Suspected	No concerns	No concerns	Major concerns	No concerns	Very low
CARB vs OLA	0	Some concerns	Suspected	No concerns	Major concerns	No concerns	No concerns	Very low
CARB vs OXC	0	Some concerns	Suspected	No concerns	Major concerns	No concerns	No concerns	Very low
CARB vs PAL	0	No concerns	Suspected	No concerns	Major concerns	No concerns	No concerns	Very low

CARB vs QUE	0	Some concerns	Suspected	No concerns	Major concerns	No concerns	No concerns	Very low
CARB vs RIS	0	Some concerns	Suspected	No concerns	Major concerns	No concerns	No concerns	Very low
CARB vs TAM	0	Some concerns	Suspected	No concerns	No concerns	No concerns	No concerns	Very low
CARB vs TOP	0	Some concerns	Suspected	No concerns	No concerns	No concerns	No concerns	Very low
CARB vs VALP	0	Some concerns	Suspected	No concerns	No concerns	Major concerns	No concerns	Very low
CARB vs VER	0	Some concerns	Suspected	No concerns	Major concerns	No concerns	No concerns	Very low
CARB vs ZIP	0	Some concerns	Suspected	No concerns	Major concerns	No concerns	No concerns	Very low
CARI vs ESL	0	Some concerns	Suspected	No concerns	Major concerns	No concerns	No concerns	Very low
CARI vs HAL	0	Some concerns	Suspected	No concerns	Major concerns	No concerns	No concerns	Very low
CARI vs LAM	0	Some concerns	Suspected	No concerns	No concerns	Major concerns	No concerns	Very low
CARI vs LIC	0	Some concerns	Suspected	No concerns	Major concerns	No concerns	No concerns	Very low
CARI vs LIT	0	Some concerns	Suspected	No concerns	Major concerns	No concerns	No concerns	Very low
CARI vs OLA	0	Some concerns	Suspected	No concerns	Major concerns	No concerns	No concerns	Very low
CARI vs OXC	0	Some concerns	Suspected	No concerns	Major concerns	No concerns	No concerns	Very low
CARI vs PAL	0	No concerns	Suspected	No concerns	Major concerns	No concerns	No concerns	Very low
CARI vs QUE	0	Some concerns	Suspected	No concerns	Major concerns	No concerns	No concerns	Very low
CARI vs RIS	0	Some concerns	Suspected	No concerns	Major concerns	No concerns	No concerns	Very low
CARI vs TAM	0	Some concerns	Suspected	No concerns	No concerns	No concerns	No concerns	Very low
CARI vs TOP	0	Some concerns	Suspected	No concerns	No concerns	No concerns	No concerns	Very low
CARI vs VALP	0	Some concerns	Suspected	No concerns	No concerns	Major concerns	No concerns	Very low
CARI vs VER	0	Some concerns	Suspected	No concerns	Major concerns	No concerns	No concerns	Very low
CARI vs ZIP	0	Some concerns	Suspected	No concerns	Major concerns	No concerns	No concerns	Very low
ESL vs HAL	0	Some concerns	Suspected	No concerns	No concerns	Major concerns	No concerns	Very low
ESL vs LAM	0	Some concerns	Suspected	No concerns	Major concerns	No concerns	No concerns	Very low
ESL vs LIC	0	Some concerns	Suspected	No concerns	Major concerns	No concerns	No concerns	Very low

ESL vs LIT	0	Some concerns	Suspected	No concerns	Major concerns	No concerns	No concerns	Very low
ESL vs OLA	0	Some concerns	Suspected	No concerns	Major concerns	No concerns	No concerns	Very low
ESL vs OXC	0	Some concerns	Suspected	No concerns	Major concerns	No concerns	No concerns	Very low
ESL vs PAL	0	No concerns	Suspected	No concerns	Major concerns	No concerns	No concerns	Very low
ESL vs QUE	0	Some concerns	Suspected	No concerns	Major concerns	No concerns	No concerns	Very low
ESL vs RIS	0	Some concerns	Suspected	No concerns	Major concerns	No concerns	No concerns	Very low
ESL vs TAM	0	Some concerns	Suspected	No concerns	No concerns	No concerns	No concerns	Very low
ESL vs TOP	0	Some concerns	Suspected	No concerns	Major concerns	No concerns	No concerns	Very low
ESL vs VALP	0	Some concerns	Suspected	No concerns	Major concerns	No concerns	No concerns	Very low
ESL vs VER	0	Some concerns	Suspected	No concerns	Major concerns	No concerns	No concerns	Very low
ESL vs ZIP	0	Some concerns	Suspected	No concerns	Major concerns	No concerns	No concerns	Very low
HAL vs LAM	0	Some concerns	Suspected	No concerns	No concerns	No concerns	No concerns	Very low
HAL vs LIC	0	Some concerns	Suspected	No concerns	No concerns	No concerns	No concerns	Very low
HAL vs OXC	0	Some concerns	Suspected	No concerns	Major concerns	No concerns	No concerns	Very low
HAL vs PAL	0	Some concerns	Suspected	No concerns	Major concerns	No concerns	No concerns	Very low
HAL vs TAM	0	Some concerns	Suspected	No concerns	No concerns	No concerns	No concerns	Very low
HAL vs TOP	0	Some concerns	Suspected	No concerns	No concerns	No concerns	No concerns	Very low
HAL vs VALP	0	Some concerns	Suspected	No concerns	No concerns	No concerns	No concerns	Very low
HAL vs VER	0	Some concerns	Suspected	No concerns	Major concerns	No concerns	No concerns	Very low
LAM vs LIC	0	Some concerns	Suspected	No concerns	Major concerns	No concerns	No concerns	Very low
LAM vs OXC	0	Some concerns	Suspected	No concerns	Major concerns	No concerns	No concerns	Very low
LAM vs PAL	0	Some concerns	Suspected	No concerns	Major concerns	No concerns	No concerns	Very low
LAM vs QUE	0	Some concerns	Suspected	No concerns	Major concerns	No concerns	No concerns	Very low
LAM vs RIS	0	Some concerns	Suspected	No concerns	No concerns	No concerns	No concerns	Very low
LAM vs TAM	0	Some concerns	Suspected	No concerns	No concerns	No concerns	No concerns	Very low

LAM vs TOP	0	Some concerns	Suspected	No concerns	Major concerns	No concerns	No concerns	Very low
LAM vs VALP	0	Some concerns	Suspected	No concerns	Major concerns	No concerns	No concerns	Very low
LAM vs VER	0	Some concerns	Suspected	No concerns	Major concerns	No concerns	No concerns	Very low
LAM vs ZIP	0	Some concerns	Suspected	No concerns	Major concerns	No concerns	No concerns	Very low
LIC vs LIT	0	Some concerns	Suspected	No concerns	Major concerns	No concerns	No concerns	Very low
LIC vs OLA	0	Some concerns	Suspected	No concerns	No concerns	Major concerns	No concerns	Very low
LIC vs OXC	0	Some concerns	Suspected	No concerns	Major concerns	No concerns	No concerns	Very low
LIC vs PAL	0	No concerns	Suspected	No concerns	Major concerns	No concerns	No concerns	Very low
LIC vs QUE	0	Some concerns	Suspected	No concerns	Major concerns	No concerns	No concerns	Very low
LIC vs RIS	0	Some concerns	Suspected	No concerns	No concerns	No concerns	No concerns	Very low
LIC vs TAM	0	Some concerns	Suspected	No concerns	No concerns	No concerns	No concerns	Very low
LIC vs TOP	0	Some concerns	Suspected	No concerns	Major concerns	No concerns	No concerns	Very low
LIC vs VALP	0	Some concerns	Suspected	No concerns	Major concerns	No concerns	No concerns	Very low
LIC vs VER	0	Some concerns	Suspected	No concerns	Major concerns	No concerns	No concerns	Very low
LIC vs ZIP	0	Some concerns	Suspected	No concerns	Major concerns	No concerns	No concerns	Very low
LIT vs OXC	0	Some concerns	Suspected	No concerns	Major concerns	No concerns	No concerns	Very low
LIT vs PAL	0	No concerns	Suspected	No concerns	Major concerns	No concerns	No concerns	Very low
LIT vs TAM	0	No concerns	Suspected	No concerns	No concerns	No concerns	No concerns	Low
LIT vs VER	0	No concerns	Suspected	No concerns	Major concerns	No concerns	No concerns	Very low
LIT vs ZIP	0	Some concerns	Suspected	No concerns	Major concerns	No concerns	No concerns	Very low
OLA vs OXC	0	Some concerns	Suspected	No concerns	Major concerns	No concerns	No concerns	Very low
OLA vs PAL	0	No concerns	Suspected	No concerns	Major concerns	No concerns	No concerns	Very low
OLA vs QUE	0	Some concerns	Suspected	No concerns	Major concerns	No concerns	No concerns	Very low
OLA vs TAM	0	No concerns	Suspected	No concerns	No concerns	No concerns	No concerns	Low
OLA vs TOP	0	No concerns	Suspected	No concerns	No concerns	No concerns	No concerns	Low

OLA vs VER	0	No concerns	Suspected	No concerns	Major concerns	No concerns	No concerns	Very low
OLA vs ZIP	0	Some concerns	Suspected	No concerns	Major concerns	No concerns	No concerns	Very low
OXC vs PAL	0	No concerns	Suspected	No concerns	Major concerns	No concerns	No concerns	Very low
OXC vs QUE	0	Some concerns	Suspected	No concerns	Major concerns	No concerns	No concerns	Very low
OXC vs RIS	0	Some concerns	Suspected	No concerns	Major concerns	No concerns	No concerns	Very low
OXC vs TAM	0	No concerns	Suspected	No concerns	No concerns	No concerns	No concerns	Low
OXC vs TOP	0	Some concerns	Suspected	No concerns	Major concerns	No concerns	No concerns	Very low
OXC vs VER	0	No concerns	Suspected	No concerns	Major concerns	No concerns	No concerns	Very low
OXC vs ZIP	0	Some concerns	Suspected	No concerns	Major concerns	No concerns	No concerns	Very low
OXC vs PLA	0	Some concerns	Suspected	No concerns	Major concerns	No concerns	No concerns	Very low
PAL vs RIS	0	No concerns	Suspected	No concerns	Major concerns	No concerns	No concerns	Very low
PAL vs TAM	0	No concerns	Suspected	No concerns	No concerns	No concerns	No concerns	Low
PAL vs TOP	0	No concerns	Suspected	No concerns	No concerns	No concerns	No concerns	Low
PAL vs VALP	0	No concerns	Suspected	No concerns	Major concerns	No concerns	No concerns	Very low
PAL vs VER	0	No concerns	Suspected	No concerns	Major concerns	No concerns	No concerns	Very low
PAL vs ZIP	0	No concerns	Suspected	No concerns	Major concerns	No concerns	No concerns	Very low
QUE vs RIS	0	Some concerns	Suspected	No concerns	Major concerns	No concerns	No concerns	Very low
QUE vs TAM	0	No concerns	Suspected	No concerns	No concerns	No concerns	No concerns	Low
QUE vs TOP	0	No concerns	Suspected	No concerns	No concerns	No concerns	No concerns	Low
QUE vs VALP	0	No concerns	Suspected	No concerns	Major concerns	No concerns	No concerns	Very low
QUE vs VER	0	No concerns	Suspected	No concerns	Major concerns	No concerns	No concerns	Very low
QUE vs ZIP	0	Some concerns	Suspected	No concerns	Major concerns	No concerns	No concerns	Very low
RIS vs TAM	0	No concerns	Suspected	No concerns	No concerns	No concerns	No concerns	Low
RIS vs TOP	0	No concerns	Suspected	No concerns	No concerns	No concerns	No concerns	Low
RIS vs VALP	0	Some concerns	Suspected	No concerns	No concerns	No concerns	No concerns	Very low

RIS vs VER	0	No concerns	Suspected	No concerns	Major concerns	No concerns	No concerns	Very low
RIS vs ZIP	0	Some concerns	Suspected	No concerns	Major concerns	No concerns	No concerns	Very low
TAM vs TOP	0	No concerns	Suspected	No concerns	No concerns	No concerns	No concerns	Low
TAM vs VALP	0	No concerns	Suspected	No concerns	No concerns	No concerns	No concerns	Low
TAM vs VER	0	No concerns	Suspected	No concerns	No concerns	No concerns	No concerns	Low
TAM vs ZIP	0	No concerns	Suspected	No concerns	No concerns	No concerns	No concerns	Low
TOP vs VALP	0	No concerns	Suspected	No concerns	No concerns	Major concerns	No concerns	Very low
TOP vs VER	0	No concerns	Suspected	No concerns	Major concerns	No concerns	No concerns	Very low
TOP vs ZIP	0	No concerns	Suspected	No concerns	No concerns	No concerns	No concerns	Low
VALP vs VER	0	No concerns	Suspected	No concerns	Major concerns	No concerns	No concerns	Very low
VALP vs ZIP	0	Some concerns	Suspected	No concerns	Major concerns	No concerns	No concerns	Very low
VER vs ZIP	0	No concerns	Suspected	No concerns	Major concerns	No concerns	No concerns	Very low

Supplementary Appendix 4. Discontinuation due to inefficacy (N = 50, n = 14284)

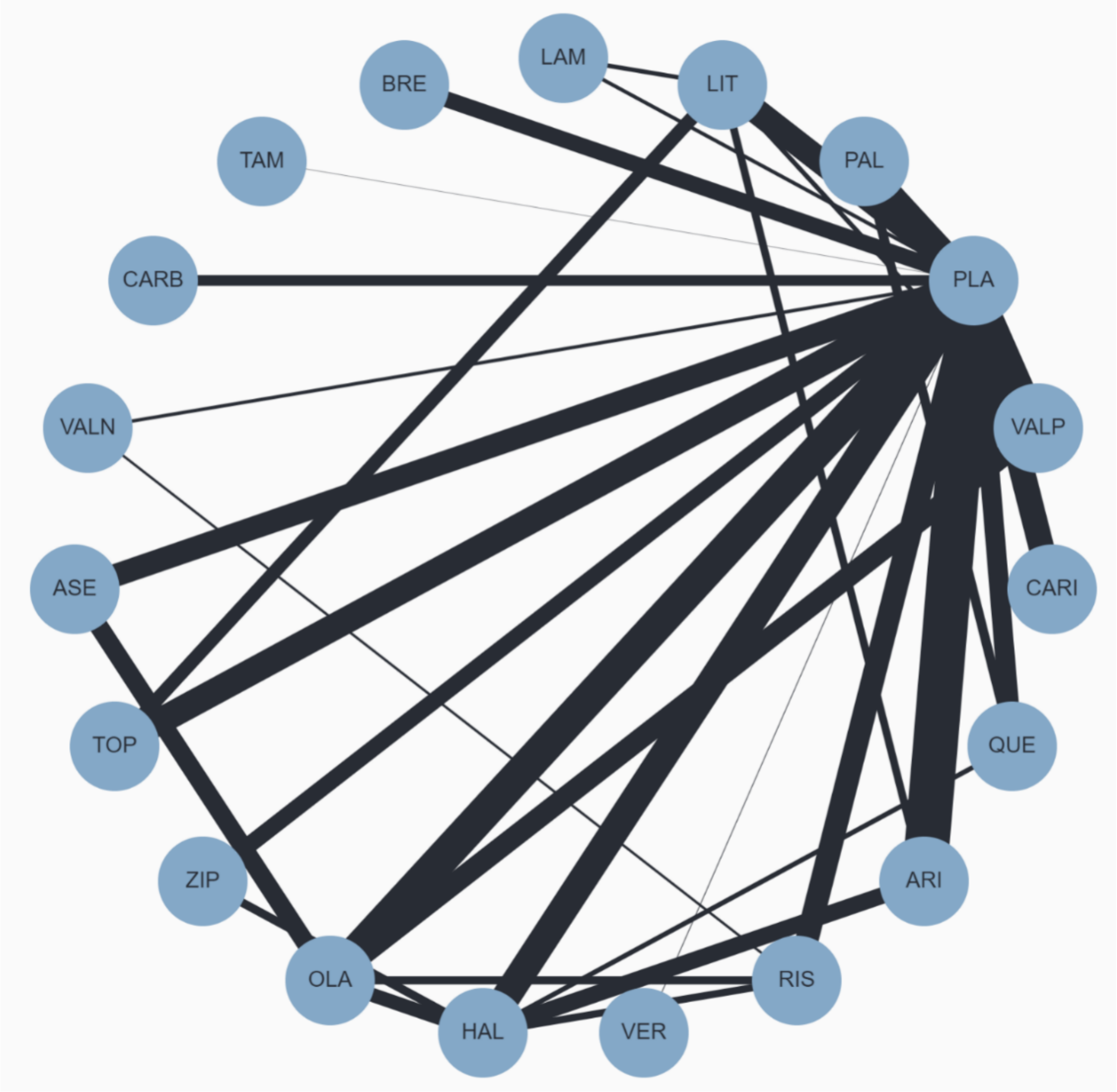

League table (risk ratio with 95% confidence interval)

ARI	1.218 (0.760, 1.954)	0.513 (0.162, 1.623)	1.365 (0.778, 2.396)	1.513 (0.880, 2.600)	**1.609 (1.170, 2.213)**	0.903 (0.395, 2.067)	0.877 (0.613, 1.256)	1.254 (0.914, 1.720)	**1.801 (1.061, 3.058)**	1.204 (0.795, 1.823)	1.469 (0.940, 2.296)	0.942 (0.202, 4.399)	**0.520 (0.345, 0.785)**	**0.365 (0.146, 0.909)**	1.009 (0.721, 1.413)	0.570 (0.278, 1.167)	1.063 (0.738, 1.531)	**0.628 (0.502, 0.786)**
	ASE	0.421 (0.126, 1.404)	1.120 (0.577, 2.176)	1.242 (0.651, 2.369)	1.320 (0.814, 2.142)	0.741 (0.301, 1.825)	0.720 (0.433, 1.197)	1.029 (0.664, 1.594)	1.478 (0.782, 2.793)	0.988 (0.573, 1.706)	1.206 (0.685, 2.122)	0.773 (0.159, 3.757)	**0.427 (0.248, 0.735)**	**0.299 (0.113, 0.797)**	0.828 (0.512, 1.339)	0.468 (0.210, 1.040)	0.872 (0.525, 1.451)	**0.516 (0.340, 0.783)**
		BRE	2.659 (0.768, 9.205)	2.947 (0.859, 10.107)	3.135 (0.983, 9.992)	1.759 (0.441, 7.016)	1.708 (0.532, 5.488)	2.442 (0.771, 7.735)	**3.509 (1.028, 11.972)**	2.346 (0.718, 7.662)	2.861 (0.867, 9.444)	1.836 (0.275, 12.239)	1.014 (0.311, 3.305)	0.711 (0.169, 2.986)	1.966 (0.618, 6.258)	1.110 (0.297, 4.151)	2.071 (0.645, 6.651)	1.224 (0.396, 3.787)
			CARB	1.108 (0.543, 2.263)	1.179 (0.661, 2.103)	0.662 (0.256, 1.712)	0.643 (0.355, 1.163)	0.919 (0.522, 1.617)	1.320 (0.652, 2.671)	0.882 (0.472, 1.650)	1.076 (0.564, 2.052)	0.690 (0.138, 3.452)	**0.381 (0.205, 0.710)**	**0.267 (0.096, 0.745)**	0.739 (0.416, 1.314)	**0.417 (0.177, 0.981)**	0.779 (0.430, 1.410)	**0.460 (0.275, 0.771)**
				CARI	1.064 (0.609, 1.859)	0.597 (0.233, 1.526)	0.580 (0.327, 1.029)	0.829 (0.481, 1.428)	1.190 (0.598, 2.370)	0.796 (0.434, 1.461)	0.971 (0.518, 1.818)	0.623 (0.126, 3.092)	**0.344 (0.188, 0.629)**	**0.241 (0.088, 0.665)**	0.667 (0.383, 1.162)	**0.377 (0.162, 0.873)**	0.703 (0.396, 1.247)	**0.415 (0.254, 0.680)**
					HAL	0.561 (0.242, 1.300)	**0.545 (0.369, 0.805)**	0.779 (0.572, 1.061)	1.119 (0.650, 1.928)	0.748 (0.498, 1.124)	0.913 (0.576, 1.447)	0.586 (0.125, 2.750)	**0.323 (0.209, 0.500)**	**0.227 (0.090, 0.570)**	**0.627 (0.439, 0.896)**	**0.354 (0.171, 0.735)**	**0.661 (0.460, 0.949)**	**0.390 (0.301, 0.507)**
						LAM	0.971 (0.429, 2.199)	1.388 (0.605, 3.186)	1.995 (0.785, 5.066)	1.333 (0.557, 3.195)	1.627 (0.669, 3.952)	1.044 (0.187, 5.833)	0.576 (0.243, 1.368)	0.404 (0.123, 1.331)	1.118 (0.485, 2.574)	0.631 (0.221, 1.803)	1.177 (0.503, 2.756)	0.696 (0.313, 1.547)
							LIT	1.429 (0.990, 2.065)	**2.054 (1.170, 3.605)**	1.373 (0.867, 2.173)	**1.675 (1.031, 2.722)**	1.075 (0.228, 5.074)	**0.593 (0.393, 0.896)**	0.416 (0.164, 1.057)	1.151 (0.802, 1.652)	0.650 (0.309, 1.364)	1.212 (0.801, 1.834)	**0.716 (0.534, 0.961)**

								OLA	1.437 (0.844, 2.446)	0.961 (0.634, 1.456)	1.172 (0.759, 1.809)	0.752 (0.161, 3.512)	**0.415 (0.274, 0.629)**	**0.291 (0.117, 0.725)**	0.805 (0.593, 1.094)	**0.454 (0.221, 0.933)**	0.848 (0.588, 1.224)	**0.501 (0.398, 0.631)**
									PAL	0.669 (0.378, 1.182)	0.816 (0.440, 1.512)	0.523 (0.106, 2.587)	**0.289 (0.160, 0.523)**	**0.203 (0.074, 0.555)**	**0.560 (0.325, 0.965)**	**0.316 (0.137, 0.728)**	0.590 (0.336, 1.036)	**0.349 (0.216, 0.564)**
										QUE	1.220 (0.722, 2.060)	0.783 (0.164, 3.743)	**0.432 (0.263, 0.710)**	**0.303 (0.117, 0.786)**	0.838 (0.543, 1.294)	0.473 (0.219, 1.020)	0.883 (0.561, 1.391)	**0.522 (0.366, 0.743)**
											RIS	0.642 (0.133, 3.093)	**0.354 (0.210, 0.596)**	**0.248 (0.098, 0.627)**	0.687 (0.434, 1.089)	**0.388 (0.177, 0.850)**	0.724 (0.446, 1.175)	**0.428 (0.290, 0.630)**
												TAM	0.552 (0.116, 2.636)	0.387 (0.066, 2.256)	1.071 (0.228, 5.022)	0.604 (0.114, 3.210)	1.128 (0.239, 5.326)	0.667 (0.145, 3.061)
													TOP	0.701 (0.271, 1.816)	**1.940 (1.267, 2.969)**	1.095 (0.509, 2.353)	**2.043 (1.297, 3.219)**	1.207 (0.853, 1.710)
														VALN	**2.767 (1.102, 6.949)**	1.561 (0.511, 4.774)	**2.914 (1.147, 7.405)**	1.722 (0.710, 4.176)
															VALP	0.564 (0.273, 1.168)	1.053 (0.716, 1.550)	**0.623 (0.483, 0.803)**

																VER	1.866 (0.889, 3.919)	1.103 (0.558, 2.181)
																	ZIP	**0.591 (0.441, 0.792)**
																		PLA

Evaluation of heterogeneity and inconsistency

Between study variance (τ^2)	Heterogeneity assessment	Random-effects design-by-treatment interaction model		
		Q	df	p
0.021	Low	35.364	28	0.119

Incoherence

	NMA, RR (95% CI)	Direct, RR (95% CI)	I^2	Indirect, RR (95% CI)	Inconsistency measures	
					Difference of RR	P value
ARI vs HAL	1.609 (1.170, 2.213)	1.839 (1.032, 3.277)	77.7%	1.518 (1.035, 2.224)	1.212 (0.606, 2.422)	0.587
ARI vs LIT	0.877 (0.613, 1.256)	0.357 (0.164, 0.779)	na	1.116 (0.745, 1.672)	0.320 (0.133, 0.771)	0.011
ARI vs PLA	0.628 (0.502, 0.786)	0.610 (0.483, 0.771)	55.7%	0.846 (0.400, 1.786)	0.722 (0.330, 1.579)	0.414
ASE vs OLA	1.029 (0.664, 1.594)	1.313 (0.752, 2.292)	0.0%	0.695 (0.343, 1.410)	1.889 (0.768, 4.650)	0.166
ASE vs PLA	0.516 (0.340, 0.783)	0.468 (0.299, 0.732)	0.0%	0.989 (0.311, 3.146)	0.473 (0.137, 1.637)	0.237
BRE vs PLA		1.224 (0.396, 3.787)	0.0%			
CARB vs PLA		0.460 (0.275, 0.771)	64.4%			
CARI vs PLA		0.415 (0.254, 0.680)	36.9%			
HAL vs OLA	0.779 (0.572, 1.061)	0.941 (0.567, 1.561)	5.4%	0.697 (0.472, 1.029)	1.350 (0.712, 2.558)	0.358
HAL vs QUE	0.748 (0.498, 1.124)	0.878 (0.501, 1.539)	na	0.628 (0.349, 1.132)	1.397 (0.619, 3.153)	0.420
HAL vs RIS	0.913 (0.576, 1.447)	0.428 (0.082, 2.226)	na	0.973 (0.602, 1.572)	0.440 (0.079, 2.448)	0.348
HAL vs ZIP	0.661 (0.460, 0.949)	0.446 (0.258, 0.771)	na	0.898 (0.554, 1.457)	0.497 (0.239, 1.031)	0.061
HAL vs PLA	0.390 (0.301, 0.507)	0.409 (0.295, 0.568)	45.6%	0.360 (0.233, 0.555)	1.137 (0.660, 1.958)	0.644
LAM vs LIT	0.971 (0.429, 2.199)	1.373 (0.499, 3.781)	0.0%	0.509 (0.128, 2.031)	2.697 (0.485, 14.984)	0.257
LAM vs PLA	0.696 (0.313, 1.547)	0.595 (0.252, 1.401)	na	1.953 (0.217, 17.586)	0.304 (0.029, 3.221)	0.323
LIT vs TOP	0.593 (0.393, 0.896)	0.737 (0.409, 1.328)	0.0%	0.483 (0.272, 0.858)	1.526 (0.670, 3.477)	0.315
LIT vs VALP	1.151 (0.802, 1.652)	1.086 (0.615, 1.919)	0.0%	1.197 (0.750, 1.910)	0.908 (0.435, 1.895)	0.796

LIT vs PLA	0.716 (0.534, 0.961)	0.631 (0.461, 0.864)	0.0%	1.690 (0.746, 3.831)	0.373 (0.155, 0.897)	0.028
OLA vs RIS	1.172 (0.759, 1.809)	0.994 (0.343, 2.882)	na	1.211 (0.752, 1.949)	0.821 (0.256, 2.635)	0.740
OLA vs VALP	0.805 (0.593, 1.094)	0.888 (0.529, 1.491)	0.0%	0.764 (0.522, 1.117)	1.163 (0.612, 2.211)	0.645
OLA vs PLA	0.501 (0.398, 0.631)	0.538 (0.405, 0.714)	0.0%	0.437 (0.294, 0.650)	1.230 (0.755, 2.002)	0.405
PAL vs QUE	0.669 (0.378, 1.182)	0.396 (0.150, 1.043)	na	0.882 (0.436, 1.784)	0.449 (0.135, 1.488)	0.190
PAL vs PLA	0.349 (0.216, 0.564)	0.348 (0.214, 0.565)	72.3%	0.401 (0.015, 10.623)	0.868 (0.032, 23.865)	0.933
QUE vs PLA	0.522 (0.366, 0.743)	0.530 (0.366, 0.766)	0.0%	0.439 (0.125, 1.539)	1.207 (0.326, 4.466)	0.778
RIS vs VALN	0.248 (0.098, 0.627)	0.370 (0.085, 1.602)	na	0.191 (0.058, 0.630)	1.940 (0.293, 12.864)	0.492
RIS vs PLA	0.428 (0.290, 0.630)	0.410 (0.270, 0.621)	0.0%	0.566 (0.197, 1.630)	0.723 (0.232, 2.254)	0.576
TAM vs PLA		0.667 (0.145, 3.061)	na			
TOP vs PLA	1.207 (0.853, 1.710)	1.226 (0.851, 1.768)	57.8%	1.038 (0.333, 3.239)	1.181 (0.358, 3.904)	0.784
VALN vs PLA	1.722 (0.710, 4.176)	1.972 (0.750, 5.183)	na	0.846 (0.092, 7.745)	2.331 (0.208, 26.120)	0.492
VALP vs PLA	0.623 (0.483, 0.803)	0.638 (0.477, 0.853)	0.0%	0.576 (0.342, 0.970)	1.108 (0.610, 2.014)	0.737
VER vs PLA		1.103 (0.558, 2.181)	na			
ZIP vs PLA	0.591 (0.441, 0.792)	0.553 (0.409, 0.748)	0.0%	1.629 (0.500, 5.307)	0.339 (0.100, 1.148)	0.082

Funnel plot (only double-blind, placebo-controlled trials)

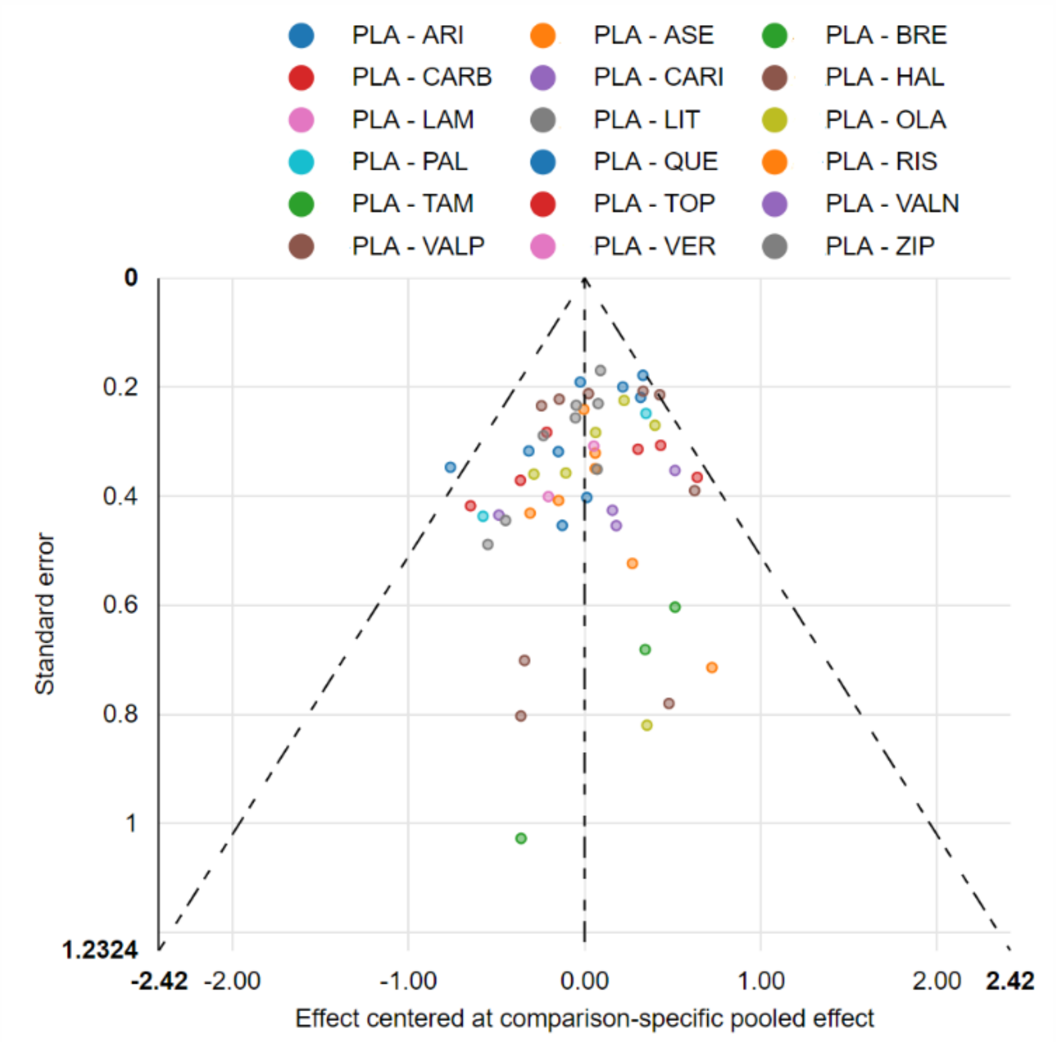

CINeMA confidence rating

Comparison	Number of studies	Within-study bias	Reporting bias	Indirectness	Imprecision	Heterogeneity	Incoherence	Confidence rating
ARI vs HAL	2	Some concerns	Suspected	No concerns	No concerns	No concerns	No concerns	Low
ARI vs LIT	1	Some concerns	Suspected	No concerns	Major concerns	No concerns	Major concerns	Very low
ARI vs PLA	6	Some concerns	Suspected	No concerns	No concerns	No concerns	No concerns	Low
ASE vs OLA	2	Some concerns	Suspected	No concerns	Major concerns	No concerns	No concerns	Very low
ASE vs PLA	3	Some concerns	Suspected	No concerns	No concerns	No concerns	No concerns	Low
BRE vs PLA	2	Some concerns	Suspected	No concerns	Major concerns	No concerns	Some concerns	Very low
CARB vs PLA	2	Some concerns	Suspected	No concerns	No concerns	No concerns	Some concerns	Very low
CARI vs PLA	3	Some concerns	Suspected	No concerns	No concerns	No concerns	Some concerns	Very low
HAL vs OLA	2	Some concerns	Suspected	No concerns	Major concerns	No concerns	No concerns	Very low
HAL vs QUE	1	Some concerns	Suspected	No concerns	Major concerns	No concerns	No concerns	Very low
HAL vs RIS	1	Some concerns	Suspected	No concerns	Major concerns	No concerns	No concerns	Very low
HAL vs ZIP	1	Some concerns	Suspected	No concerns	No concerns	Major concerns	Major concerns	Very low
HAL vs PLA	5	Some concerns	Suspected	No concerns	No concerns	No concerns	No concerns	Low
LAM vs LIT	2	Some concerns	Suspected	No concerns	Major concerns	No concerns	No concerns	Very low
LAM vs PLA	1	Some concerns	Suspected	No concerns	Major concerns	No concerns	No concerns	Very low
LIT vs TOP	2	No concerns	Suspected	No concerns	No concerns	No concerns	No concerns	Moderate
LIT vs VALP	3	Some concerns	Suspected	No concerns	Major concerns	No concerns	No concerns	Very low
LIT vs PLA	5	Some concerns	Suspected	No concerns	No concerns	Major concerns	Major concerns	Very low
OLA vs RIS	1	Some concerns	Suspected	No concerns	Major concerns	No concerns	No concerns	Very low
OLA vs VALP	4	Some concerns	Suspected	No concerns	Major concerns	No concerns	No concerns	Very low
OLA vs PLA	6	Some concerns	Suspected	No concerns	No concerns	No concerns	No concerns	Low
PAL vs QUE	1	No concerns	Suspected	No concerns	Major concerns	No concerns	No concerns	Low
PAL vs PLA	2	No concerns	Suspected	No concerns	No concerns	No concerns	No concerns	Moderate

QUE vs PLA	3	Some concerns	Suspected	No concerns	No concerns	No concerns	No concerns	Low
RIS vs VALN	1	No concerns	Suspected	No concerns	No concerns	No concerns	No concerns	Moderate
RIS vs PLA	4	Some concerns	Suspected	No concerns	No concerns	No concerns	No concerns	Low
TAM vs PLA	1	No concerns	Suspected	No concerns	Major concerns	No concerns	Some concerns	Very low
TOP vs PLA	4	No concerns	Suspected	No concerns	Major concerns	No concerns	No concerns	Low
VALN vs PLA	1	No concerns	Suspected	No concerns	Major concerns	No concerns	No concerns	Low
VALP vs PLA	4	Some concerns	Suspected	No concerns	No concerns	No concerns	No concerns	Low
VER vs PLA	1	No concerns	Suspected	No concerns	Major concerns	No concerns	Some concerns	Very low
ZIP vs PLA	3	Some concerns	Suspected	No concerns	No concerns	No concerns	Major concerns	Very low
ARI vs ASE	0	Some concerns	Suspected	No concerns	Major concerns	No concerns	Some concerns	Very low
ARI vs BRE	0	Some concerns	Suspected	No concerns	Major concerns	No concerns	Some concerns	Very low
ARI vs CARB	0	Some concerns	Suspected	No concerns	Major concerns	No concerns	Some concerns	Very low
ARI vs CARI	0	Some concerns	Suspected	No concerns	Major concerns	No concerns	Some concerns	Very low
ARI vs LAM	0	Some concerns	Suspected	No concerns	Major concerns	No concerns	Some concerns	Very low
ARI vs OLA	0	Some concerns	Suspected	No concerns	Major concerns	No concerns	Some concerns	Very low
ARI vs PAL	0	Some concerns	Suspected	No concerns	No concerns	Major concerns	Some concerns	Very low
ARI vs QUE	0	Some concerns	Suspected	No concerns	Major concerns	No concerns	Some concerns	Very low
ARI vs RIS	0	Some concerns	Suspected	No concerns	Major concerns	No concerns	Some concerns	Very low
ARI vs TAM	0	Some concerns	Suspected	No concerns	Major concerns	No concerns	Some concerns	Very low
ARI vs TOP	0	Some concerns	Suspected	No concerns	No concerns	No concerns	Some concerns	Very low
ARI vs VALN	0	Some concerns	Suspected	No concerns	No concerns	No concerns	Some concerns	Very low
ARI vs VALP	0	Some concerns	Suspected	No concerns	Major concerns	No concerns	Some concerns	Very low
ARI vs VER	0	Some concerns	Suspected	No concerns	Major concerns	No concerns	Some concerns	Very low
ARI vs ZIP	0	Some concerns	Suspected	No concerns	Major concerns	No concerns	Some concerns	Very low
ASE vs BRE	0	Some concerns	Suspected	No concerns	Major concerns	No concerns	Some concerns	Very low

ASE vs CARB	0	Some concerns	Suspected	No concerns	Major concerns	No concerns	Some concerns	Very low
ASE vs CARI	0	Some concerns	Suspected	No concerns	Major concerns	No concerns	Some concerns	Very low
ASE vs HAL	0	Some concerns	Suspected	No concerns	Major concerns	No concerns	Some concerns	Very low
ASE vs LAM	0	Some concerns	Suspected	No concerns	Major concerns	No concerns	Some concerns	Very low
ASE vs LIT	0	Some concerns	Suspected	No concerns	Major concerns	No concerns	Some concerns	Very low
ASE vs PAL	0	No concerns	Suspected	No concerns	Major concerns	No concerns	Some concerns	Very low
ASE vs QUE	0	Some concerns	Suspected	No concerns	Major concerns	No concerns	Some concerns	Very low
ASE vs RIS	0	Some concerns	Suspected	No concerns	Major concerns	No concerns	Some concerns	Very low
ASE vs TAM	0	No concerns	Suspected	No concerns	Major concerns	No concerns	Some concerns	Very low
ASE vs TOP	0	No concerns	Suspected	No concerns	No concerns	No concerns	Some concerns	Very low
ASE vs VALN	0	No concerns	Suspected	No concerns	No concerns	No concerns	Some concerns	Very low
ASE vs VALP	0	Some concerns	Suspected	No concerns	Major concerns	No concerns	Some concerns	Very low
ASE vs VER	0	No concerns	Suspected	No concerns	Major concerns	No concerns	Some concerns	Very low
ASE vs ZIP	0	Some concerns	Suspected	No concerns	Major concerns	No concerns	Some concerns	Very low
BRE vs CARB	0	Some concerns	Suspected	No concerns	Major concerns	No concerns	Some concerns	Very low
BRE vs CARI	0	Some concerns	Suspected	No concerns	Major concerns	No concerns	Some concerns	Very low
BRE vs HAL	0	Some concerns	Suspected	No concerns	Major concerns	No concerns	Some concerns	Very low
BRE vs LAM	0	Some concerns	Suspected	No concerns	Major concerns	No concerns	Some concerns	Very low
BRE vs LIT	0	Some concerns	Suspected	No concerns	Major concerns	No concerns	Some concerns	Very low
BRE vs OLA	0	Some concerns	Suspected	No concerns	Major concerns	No concerns	Some concerns	Very low
BRE vs PAL	0	Some concerns	Suspected	No concerns	No concerns	Major concerns	Some concerns	Very low
BRE vs QUE	0	Some concerns	Suspected	No concerns	Major concerns	No concerns	Some concerns	Very low
BRE vs RIS	0	Some concerns	Suspected	No concerns	Major concerns	No concerns	Some concerns	Very low
BRE vs TAM	0	Some concerns	Suspected	No concerns	Major concerns	No concerns	Some concerns	Very low
BRE vs TOP	0	Some concerns	Suspected	No concerns	Major concerns	No concerns	Some concerns	Very low

BRE vs VALN	0	Some concerns	Suspected	No concerns	Major concerns	No concerns	Some concerns	Very low
BRE vs VALP	0	Some concerns	Suspected	No concerns	Major concerns	No concerns	Some concerns	Very low
BRE vs VER	0	Some concerns	Suspected	No concerns	Major concerns	No concerns	Some concerns	Very low
BRE vs ZIP	0	Some concerns	Suspected	No concerns	Major concerns	No concerns	Some concerns	Very low
CARB vs CARI	0	Some concerns	Suspected	No concerns	Major concerns	No concerns	Some concerns	Very low
CARB vs HAL	0	Some concerns	Suspected	No concerns	Major concerns	No concerns	Some concerns	Very low
CARB vs LAM	0	Some concerns	Suspected	No concerns	Major concerns	No concerns	Some concerns	Very low
CARB vs LIT	0	Some concerns	Suspected	No concerns	Major concerns	No concerns	Some concerns	Very low
CARB vs OLA	0	Some concerns	Suspected	No concerns	Major concerns	No concerns	Some concerns	Very low
CARB vs PAL	0	Some concerns	Suspected	No concerns	Major concerns	No concerns	Some concerns	Very low
CARB vs QUE	0	Some concerns	Suspected	No concerns	Major concerns	No concerns	Some concerns	Very low
CARB vs RIS	0	Some concerns	Suspected	No concerns	Major concerns	No concerns	Some concerns	Very low
CARB vs TAM	0	Some concerns	Suspected	No concerns	Major concerns	No concerns	Some concerns	Very low
CARB vs TOP	0	Some concerns	Suspected	No concerns	No concerns	No concerns	Some concerns	Very low
CARB vs VALN	0	Some concerns	Suspected	No concerns	No concerns	No concerns	Some concerns	Very low
CARB vs VALP	0	Some concerns	Suspected	No concerns	Major concerns	No concerns	Some concerns	Very low
CARB vs VER	0	Some concerns	Suspected	No concerns	No concerns	Major concerns	Some concerns	Very low
CARB vs ZIP	0	Some concerns	Suspected	No concerns	Major concerns	No concerns	Some concerns	Very low
CARI vs HAL	0	Some concerns	Suspected	No concerns	Major concerns	No concerns	Some concerns	Very low
CARI vs LAM	0	Some concerns	Suspected	No concerns	Major concerns	No concerns	Some concerns	Very low
CARI vs LIT	0	Some concerns	Suspected	No concerns	Major concerns	No concerns	Some concerns	Very low
CARI vs OLA	0	Some concerns	Suspected	No concerns	Major concerns	No concerns	Some concerns	Very low
CARI vs PAL	0	Some concerns	Suspected	No concerns	Major concerns	No concerns	Some concerns	Very low
CARI vs QUE	0	Some concerns	Suspected	No concerns	Major concerns	No concerns	Some concerns	Very low
CARI vs RIS	0	Some concerns	Suspected	No concerns	Major concerns	No concerns	Some concerns	Very low

CARI vs TAM	0	Some concerns	Suspected	No concerns	Major concerns	No concerns	Some concerns	Very low
CARI vs TOP	0	Some concerns	Suspected	No concerns	No concerns	No concerns	Some concerns	Very low
CARI vs VALN	0	Some concerns	Suspected	No concerns	No concerns	No concerns	Some concerns	Very low
CARI vs VALP	0	Some concerns	Suspected	No concerns	Major concerns	No concerns	Some concerns	Very low
CARI vs VER	0	Some concerns	Suspected	No concerns	No concerns	No concerns	Some concerns	Very low
CARI vs ZIP	0	Some concerns	Suspected	No concerns	Major concerns	No concerns	Some concerns	Very low
HAL vs LAM	0	Some concerns	Suspected	No concerns	Major concerns	No concerns	Some concerns	Very low
HAL vs LIT	0	Some concerns	Suspected	No concerns	No concerns	No concerns	Some concerns	Very low
HAL vs PAL	0	Some concerns	Suspected	No concerns	Major concerns	No concerns	Some concerns	Very low
HAL vs TAM	0	Some concerns	Suspected	No concerns	Major concerns	No concerns	Some concerns	Very low
HAL vs TOP	0	Some concerns	Suspected	No concerns	No concerns	No concerns	Some concerns	Very low
HAL vs VALN	0	Some concerns	Suspected	No concerns	No concerns	No concerns	Some concerns	Very low
HAL vs VALP	0	Some concerns	Suspected	No concerns	No concerns	Major concerns	Some concerns	Very low
HAL vs VER	0	Some concerns	Suspected	No concerns	No concerns	No concerns	Some concerns	Very low
LAM vs OLA	0	Some concerns	Suspected	No concerns	Major concerns	No concerns	Some concerns	Very low
LAM vs PAL	0	Some concerns	Suspected	No concerns	Major concerns	No concerns	Some concerns	Very low
LAM vs QUE	0	Some concerns	Suspected	No concerns	Major concerns	No concerns	Some concerns	Very low
LAM vs RIS	0	Some concerns	Suspected	No concerns	Major concerns	No concerns	Some concerns	Very low
LAM vs TAM	0	Some concerns	Suspected	No concerns	Major concerns	No concerns	Some concerns	Very low
LAM vs TOP	0	Some concerns	Suspected	No concerns	Major concerns	No concerns	Some concerns	Very low
LAM vs VALN	0	Some concerns	Suspected	No concerns	Major concerns	No concerns	Some concerns	Very low
LAM vs VALP	0	Some concerns	Suspected	No concerns	Major concerns	No concerns	Some concerns	Very low
LAM vs VER	0	Some concerns	Suspected	No concerns	Major concerns	No concerns	Some concerns	Very low
LAM vs ZIP	0	Some concerns	Suspected	No concerns	Major concerns	No concerns	Some concerns	Very low
LIT vs OLA	0	Some concerns	Suspected	No concerns	Major concerns	No concerns	Some concerns	Very low

LIT vs PAL	0	No concerns	Suspected	No concerns	No concerns	No concerns	Some concerns	Very low
LIT vs QUE	0	Some concerns	Suspected	No concerns	Major concerns	No concerns	Some concerns	Very low
LIT vs RIS	0	Some concerns	Suspected	No concerns	No concerns	Major concerns	Some concerns	Very low
LIT vs TAM	0	No concerns	Suspected	No concerns	Major concerns	No concerns	Some concerns	Very low
LIT vs VALN	0	No concerns	Suspected	No concerns	Major concerns	No concerns	Some concerns	Very low
LIT vs VER	0	No concerns	Suspected	No concerns	Major concerns	No concerns	Some concerns	Very low
LIT vs ZIP	0	Some concerns	Suspected	No concerns	Major concerns	No concerns	Some concerns	Very low
OLA vs PAL	0	No concerns	Suspected	No concerns	Major concerns	No concerns	Some concerns	Very low
OLA vs QUE	0	Some concerns	Suspected	No concerns	Major concerns	No concerns	Some concerns	Very low
OLA vs TAM	0	No concerns	Suspected	No concerns	Major concerns	No concerns	Some concerns	Very low
OLA vs TOP	0	No concerns	Suspected	No concerns	No concerns	No concerns	Some concerns	Very low
OLA vs VALN	0	No concerns	Suspected	No concerns	No concerns	No concerns	Some concerns	Very low
OLA vs VER	0	No concerns	Suspected	No concerns	No concerns	Major concerns	Some concerns	Very low
OLA vs ZIP	0	Some concerns	Suspected	No concerns	Major concerns	No concerns	Some concerns	Very low
PAL vs RIS	0	No concerns	Suspected	No concerns	Major concerns	No concerns	Some concerns	Very low
PAL vs TAM	0	No concerns	Suspected	No concerns	Major concerns	No concerns	Some concerns	Very low
PAL vs TOP	0	No concerns	Suspected	No concerns	No concerns	No concerns	Some concerns	Very low
PAL vs VALN	0	No concerns	Suspected	No concerns	No concerns	No concerns	Some concerns	Very low
PAL vs VALP	0	No concerns	Suspected	No concerns	No concerns	Major concerns	Some concerns	Very low
PAL vs VER	0	No concerns	Suspected	No concerns	No concerns	No concerns	Some concerns	Very low
PAL vs ZIP	0	No concerns	Suspected	No concerns	Major concerns	No concerns	Some concerns	Very low
QUE vs RIS	0	Some concerns	Suspected	No concerns	Major concerns	No concerns	Some concerns	Very low
QUE vs TAM	0	No concerns	Suspected	No concerns	Major concerns	No concerns	Some concerns	Very low
QUE vs TOP	0	No concerns	Suspected	No concerns	No concerns	No concerns	Some concerns	Very low
QUE vs VALN	0	No concerns	Suspected	No concerns	No concerns	No concerns	Some concerns	Very low

QUE vs VALP	0	Some concerns	Suspected	No concerns	Major concerns	No concerns	Some concerns	Very low
QUE vs VER	0	No concerns	Suspected	No concerns	Major concerns	No concerns	Some concerns	Very low
QUE vs ZIP	0	Some concerns	Suspected	No concerns	Major concerns	No concerns	Some concerns	Very low
RIS vs TAM	0	No concerns	Suspected	No concerns	Major concerns	No concerns	Some concerns	Very low
RIS vs TOP	0	Some concerns	Suspected	No concerns	No concerns	No concerns	Some concerns	Very low
RIS vs VALP	0	Some concerns	Suspected	No concerns	Major concerns	No concerns	Some concerns	Very low
RIS vs VER	0	No concerns	Suspected	No concerns	No concerns	No concerns	Some concerns	Very low
RIS vs ZIP	0	Some concerns	Suspected	No concerns	Major concerns	No concerns	Some concerns	Very low
TAM vs TOP	0	No concerns	Suspected	No concerns	Major concerns	No concerns	Some concerns	Very low
TAM vs VALN	0	No concerns	Suspected	No concerns	Major concerns	No concerns	Some concerns	Very low
TAM vs VALP	0	No concerns	Suspected	No concerns	Major concerns	No concerns	Some concerns	Very low
TAM vs VER	0	No concerns	Suspected	No concerns	Major concerns	No concerns	Some concerns	Very low
TAM vs ZIP	0	No concerns	Suspected	No concerns	Major concerns	No concerns	Some concerns	Very low
TOP vs VALN	0	No concerns	Suspected	No concerns	Major concerns	No concerns	Some concerns	Very low
TOP vs VALP	0	No concerns	Suspected	No concerns	No concerns	No concerns	Some concerns	Very low
TOP vs VER	0	No concerns	Suspected	No concerns	Major concerns	No concerns	Some concerns	Very low
TOP vs ZIP	0	No concerns	Suspected	No concerns	No concerns	No concerns	Some concerns	Very low
VALN vs VALP	0	No concerns	Suspected	No concerns	No concerns	No concerns	Some concerns	Very low
VALN vs VER	0	No concerns	Suspected	No concerns	Major concerns	No concerns	Some concerns	Very low
VALN vs ZIP	0	No concerns	Suspected	No concerns	No concerns	No concerns	Some concerns	Very low
VALP vs VER	0	No concerns	Suspected	No concerns	Major concerns	No concerns	Some concerns	Very low
VALP vs ZIP	0	Some concerns	Suspected	No concerns	Major concerns	No concerns	Some concerns	Very low
VER vs ZIP	0	No concerns	Suspected	No concerns	Major concerns	No concerns	Some concerns	Very low

Supplementary Appendix 5. Clinical remission (N = 31, n = 9320)

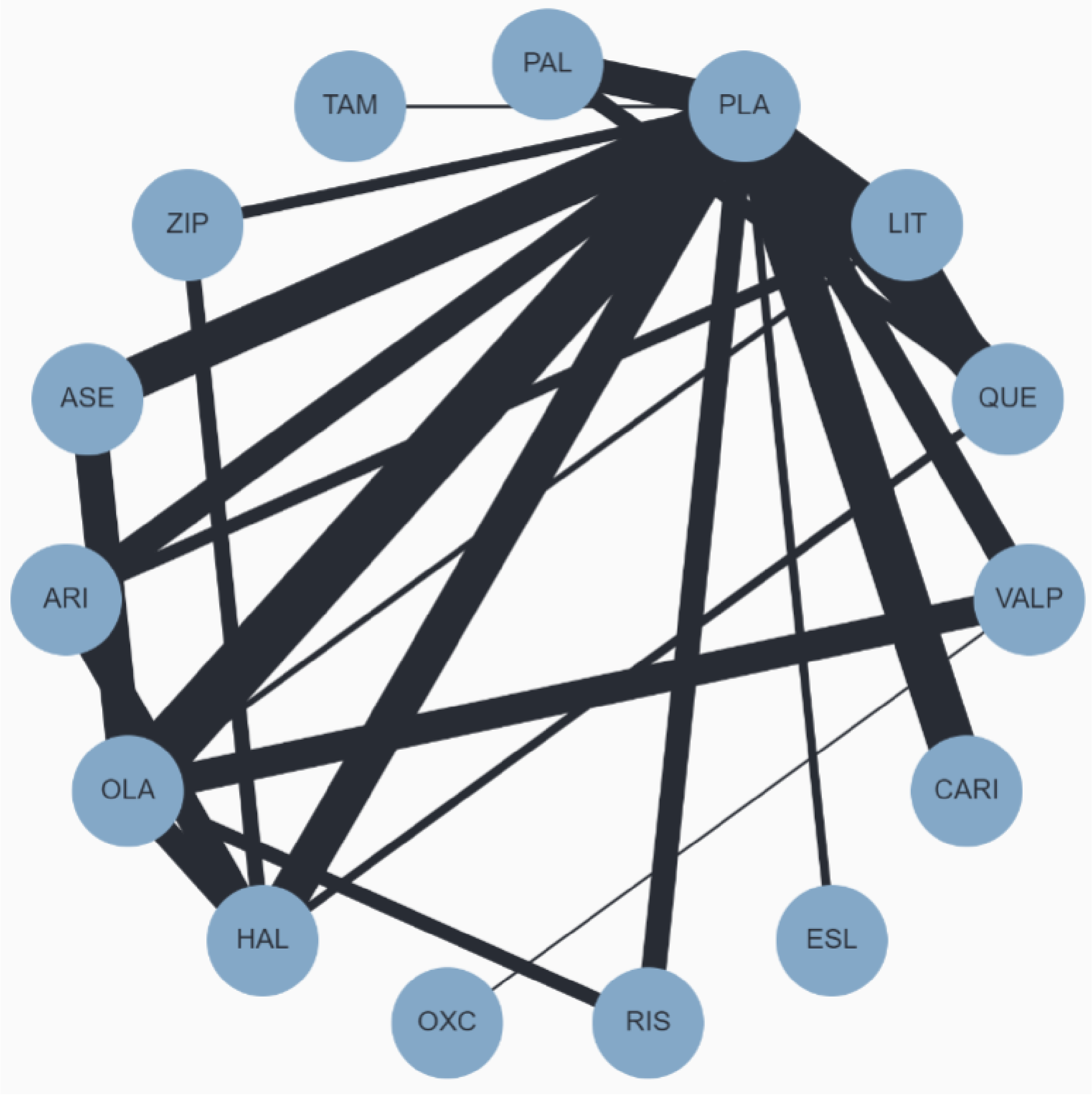

League table (risk ratio with 95% confidence interval)

ARI	1.065 (0.764, 1.484)	0.903 (0.634, 1.287)	1.316 (0.827, 2.093)	0.974 (0.761, 1.246)	1.134 (0.848, 1.515)	0.929 (0.709, 1.218)	1.285 (0.772, 2.139)	1.027 (0.714, 1.478)	0.960 (0.710, 1.297)	0.834 (0.572, 1.216)	0.169 (0.022, 1.299)	1.142 (0.816, 1.600)	1.695 (0.056, 51.183)	**1.428 (1.115, 1.829)**
	ASE	0.848 (0.600, 1.199)	1.236 (0.781, 1.953)	0.914 (0.679, 1.231)	1.065 (0.781, 1.451)	0.873 (0.689, 1.106)	1.207 (0.733, 1.986)	0.965 (0.672, 1.384)	0.901 (0.663, 1.225)	0.783 (0.545, 1.126)	0.159 (0.021, 1.218)	1.073 (0.779, 1.476)	1.592 (0.053, 48.139)	**1.341 (1.059, 1.698)**
		CARI	1.457 (0.913, 2.324)	1.078 (0.777, 1.496)	1.255 (0.895, 1.759)	1.029 (0.763, 1.387)	1.423 (0.847, 2.391)	1.137 (0.782, 1.654)	1.062 (0.766, 1.474)	0.923 (0.626, 1.361)	0.187 (0.024, 1.439)	1.265 (0.890, 1.797)	1.877 (0.062, 56.860)	**1.581 (1.228, 2.035)**
			ESL	0.740 (0.474, 1.154)	0.862 (0.548, 1.354)	0.706 (0.462, 1.079)	0.977 (0.536, 1.779)	0.781 (0.483, 1.262)	0.729 (0.468, 1.138)	0.634 (0.388, 1.036)	0.129 (0.016, 1.010)	0.868 (0.547, 1.378)	1.288 (0.042, 39.559)	1.085 (0.733, 1.607)
				HAL	1.165 (0.885, 1.532)	0.954 (0.768, 1.186)	1.320 (0.811, 2.150)	1.055 (0.753, 1.479)	0.986 (0.754, 1.289)	0.857 (0.605, 1.213)	0.174 (0.023, 1.328)	1.173 (0.867, 1.588)	1.741 (0.058, 52.269)	**1.467 (1.191, 1.807)**
					LIT	0.820 (0.647, 1.038)	1.134 (0.691, 1.860)	0.906 (0.648, 1.267)	0.847 (0.668, 1.074)	0.736 (0.514, 1.053)	0.149 (0.019, 1.143)	1.008 (0.735, 1.381)	1.495 (0.050, 45.155)	**1.259 (1.007, 1.576)**
						OLA	1.383 (0.880, 2.174)	1.106 (0.808, 1.513)	1.033 (0.809, 1.319)	0.898 (0.664, 1.213)	0.182 (0.024, 1.386)	1.229 (0.965, 1.566)	1.824 (0.061, 54.883)	**1.537 (1.309, 1.803)**
							OXC	0.799 (0.471, 1.357)	0.747 (0.456, 1.224)	0.649 (0.382, 1.103)	0.132 (0.017, 1.047)	0.889 (0.606, 1.303)	1.319 (0.043, 40.772)	1.111 (0.706, 1.748)

								PAL	0.934 (0.697, 1.252)	0.812 (0.543, 1.214)	0.165 (0.021, 1.269)	1.112 (0.771, 1.604)	1.650 (0.054, 50.074)	**1.390 (1.054, 1.833)**
									QUE	0.869 (0.609, 1.240)	0.176 (0.023, 1.347)	1.190 (0.870, 1.627)	1.766 (0.059, 53.298)	**1.488 (1.208, 1.832)**
										RIS	0.203 (0.026, 1.567)	1.370 (0.948, 1.978)	2.033 (0.067, 61.757)	**1.712 (1.275, 2.299)**
											TAM	6.753 (0.880, 51.832)	10.022 (0.191, 524.634)	**8.441 (1.116, 63.841)**
												VALP	1.484 (0.049, 44.898)	1.250 (0.980, 1.595)
													ZIP	0.842 (0.028, 25.282)
														PLA

Study	The definition of clinical remission
Berwaerts 2012	YMRS ≤ 12
Bowden 2005	YMRS ≤ 12
Bowden 2006	MRS ≤ 12
Calabrese 2015	YMRS ≤ 12

Cutler 2011	YMRS ≤ 12
Durgam 2015	YMRS ≤ 12
Grunze 2015 (203)	YMRS < 12
Grunze 2015 (204)	YMRS < 12
Hirschfeld 2004	YMRS ≤ 12
Kakkar 2009	YMRS ≤ 12
Katagiri 2012	YMRS ≤ 12
Keck 2009	YMRS ≤ 12
Khanna 2005	YMRS ≤ 8
Landbloom 2016	YMRS ≤ 12
Li 2008	YMRS ≤ 12
McIntyre 2005	YMRS ≤ 12
McIntyre 2009	YMRS ≤ 12
McIntyre 2010	YMRS ≤ 12
Niufan 2008	YMRS ≤ 12
Perlis 2006	YMRS ≤ 12 + HAMD ≤ 8
Sachs 2015	YMRS ≤ 12
Tohen 2000	YMRS ≤ 12
Tohen 2002	YMRS ≤ 12
Tohen 2003	YMRS ≤ 12 + HAMD ≤ 8
Tohen 2008	YMRS ≤ 12
Vieta 2005	YMRS < 12
Vieta 2010 ZIP	MRS ≤ 8
Vieta 2010 PAL	YMRS ≤ 12
Yildiz 2008	YMRS ≤ 12

Young 2009	YMRS $\leq$ 12
Zarate 2007	YMRS $\leq$ 7

HAMD: Hamilton Rating Scale for Depression, MRS: Mania Rating Scale (Schedule for Affective Disorders and Schizophrenia), YMRS: Young Mania Rating Scale

Evaluation of heterogeneity and inconsistency

Between study variance (τ^2)	Heterogeneity assessment	Random-effects design-by-treatment interaction model		
		Q	df	p
0.021	Low	22.110	20	0.335

Incoherence

	NMA, RR (95% CI)	Direct, RR (95% CI)	I^2	Indirect, RR (95% CI)	Inconsistency measures	
					Difference of RR	P value
ARI vs HAL	0.974 (0.761, 1.246)	1.039 (0.773, 1.395)	0.0%	0.838 (0.534, 1.314)	1.240 (0.724, 2.123)	0.433
ARI vs LIT	1.134 (0.848, 1.515)	1.120 (0.731, 1.716)	na	1.146 (0.772, 1.701)	0.978 (0.547, 1.749)	0.939
ARI vs PLA	1.428 (1.115, 1.829)	1.293 (0.955, 1.751)	0.0%	1.742 (1.134, 2.677)	0.742 (0.439, 1.255)	0.266
ASE vs OLA	0.873 (0.689, 1.106)	0.885 (0.666, 1.175)	61.4%	0.845 (0.550, 1.301)	1.046 (0.625, 1.752)	0.864
ASE vs PLA	1.341 (1.059, 1.698)	1.342 (1.024, 1.757)	35.3%	1.338 (0.824, 2.173)	1.002 (0.575, 1.746)	0.994
CARI vs PLA		1.581 (1.228, 2.035)	0.0%			
ESL vs PLA		1.085 (0.733, 1.607)	80.5%			
HAL vs OLA	0.954 (0.768, 1.186)	1.036 (0.771, 1.392)	76.2%	0.866 (0.628, 1.194)	1.197 (0.774, 1.852)	0.420
HAL vs QUE	0.986 (0.754, 1.289)	1.325 (0.791, 2.221)	na	0.884 (0.646, 1.210)	1.499 (0.819, 2.742)	0.189
HAL vs ZIP	1.741 (0.058, 52.269)	1.035 (0.020, 52.541)	na	8.314 (0.009, 7530.374)	0.125 (0.000, 322.660)	0.603
HAL vs PLA	1.467 (1.191, 1.807)	1.394 (1.058, 1.836)	0.0%	1.570 (1.141, 2.161)	0.888 (0.582, 1.353)	0.580
LIT vs OLA	0.820 (0.647, 1.038)	0.852 (0.591, 1.230)	na	0.797 (0.586, 1.085)	1.069 (0.662, 1.727)	0.784
LIT vs QUE	0.847 (0.668, 1.074)	0.768 (0.566, 1.042)	23.6%	0.985 (0.674, 1.439)	0.780 (0.479, 1.269)	0.316
LIT vs PLA	1.259 (1.007, 1.576)	1.476 (1.041, 2.091)	44.5%	1.127 (0.841, 1.510)	1.310 (0.831, 2.065)	0.245
OLA vs RIS	0.898 (0.664, 1.213)	1.354 (0.870, 2.105)	na	0.627 (0.415, 0.947)	2.158 (1.180, 3.950)	0.013
OLA vs VALP	1.229 (0.965, 1.566)	1.215 (0.903, 1.633)	48.2%	1.260 (0.828, 1.917)	0.964 (0.577, 1.611)	0.889
OLA vs PLA	1.537 (1.309, 1.803)	1.401 (1.134, 1.732)	35.1%	1.737 (1.361, 2.218)	0.807 (0.584, 1.115)	0.193

OXC vs VALP		0.889 (0.606, 1.303)	na			
PAL vs QUE	0.934 (0.697, 1.252)	1.099 (0.755, 1.600)	na	0.726 (0.455, 1.158)	1.514 (0.832, 2.755)	0.175
PAL vs PLA	1.390 (1.054, 1.833)	1.333 (0.979, 1.815)	84.5%	1.650 (0.882, 3.085)	0.808 (0.402, 1.624)	0.549
QUE vs PLA	1.488 (1.208, 1.832)	1.579 (1.233, 2.021)	17.3%	1.289 (0.877, 1.893	1.225 (0.776, 1.935	0.384
RIS vs PLA	1.712 (1.275, 2.299)	2.312 (1.585, 3.372)	48.1%	1.071 (0.668, 1.717)	2.158 (1.180, 3.950)	0.013
TAM vs PLA		8.441 (1.116, 63.841)	0.0%			
VALP vs PLA	1.250 (0.980, 1.595)	1.222 (0.905, 1.650)	38.1%	1.306 (0.860, 1.982)	0.936 (0.560, 1.565)	0.801
ZIP vs PLA	0.842 (0.028, 25.282)	0.501 (0.010, 25.381)	na	4.039 (0.004, 3708.935)	0.124 (0.000, 325.219)	0.603

Forest plot (vs placebo, the numbers are risk ratio with 95% confidence interval)

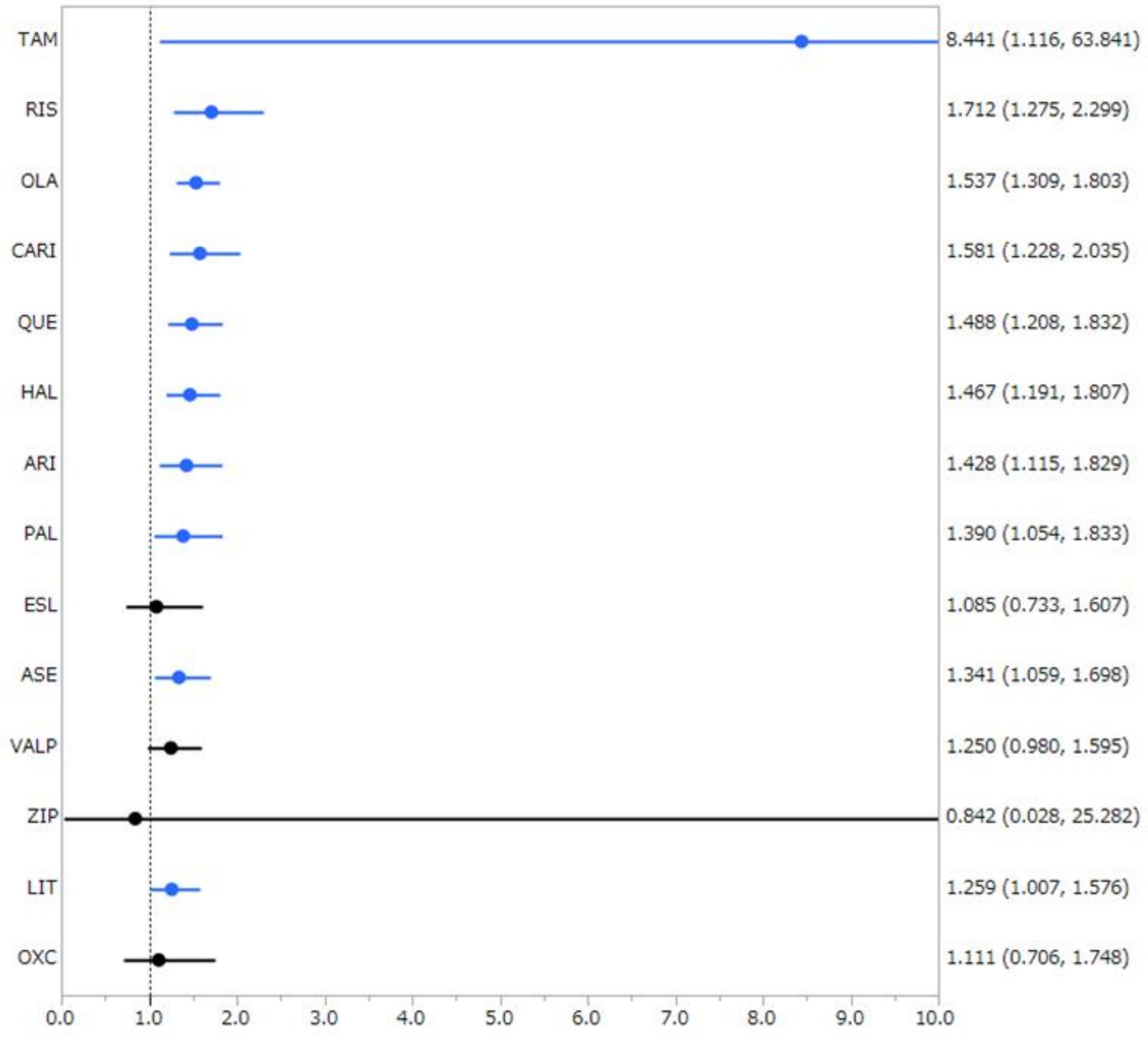

P-score

TAM	0.989
RIS	0.762
OLA	0.721
CARI	0.693
QUE	0.595
HAL	0.571
ARI	0.513
PAL	0.506
ESL	0.463
ASE	0.452
VALP	0.374
ZIP	0.303
LIT	0.270
OXC	0.165

Funnel plot (only double-blind, placebo-controlled trials)

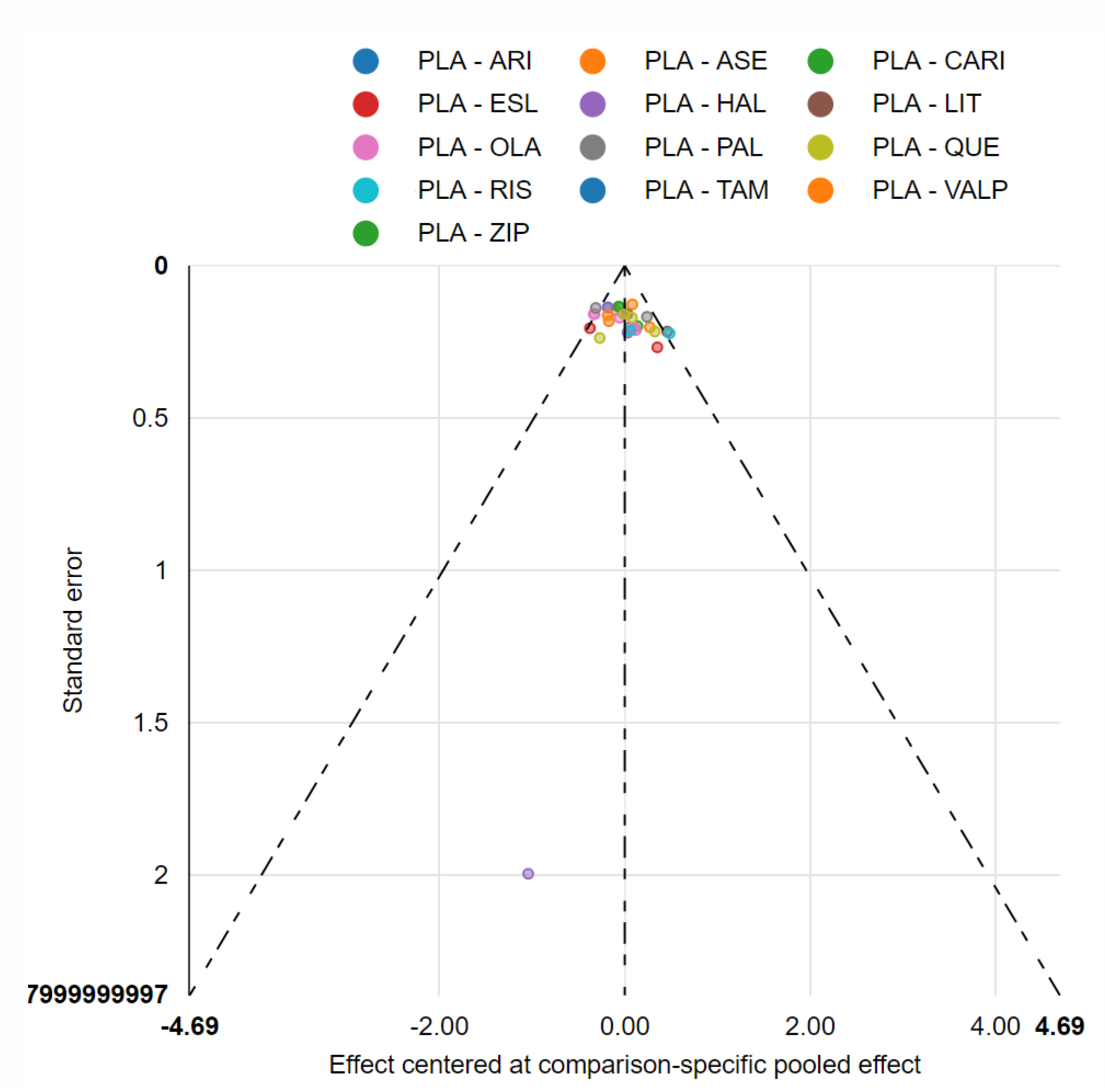

CINeMA confidence rating

Comparison	Number of studies	Within-study bias	Reporting bias	Indirectness	Imprecision	Heterogeneity	Incoherence	Confidence rating
ARI vs HAL	2	Some concerns	Suspected	No concerns	Major concerns	No concerns	No concerns	Very low
ARI vs LIT	1	Some concerns	Suspected	No concerns	Major concerns	No concerns	No concerns	Very low
ARI vs PLA	2	Some concerns	Suspected	No concerns	No concerns	Major concerns	No concerns	Very low
ASE vs OLA	2	Some concerns	Suspected	No concerns	Major concerns	No concerns	No concerns	Very low
ASE vs PLA	3	Some concerns	Suspected	No concerns	No concerns	Major concerns	No concerns	Very low
CARI vs PLA	3	Some concerns	Suspected	No concerns	No concerns	No concerns	No concerns	Low
ESL vs PLA	2	Some concerns	Suspected	No concerns	Major concerns	No concerns	No concerns	Very low
HAL vs OLA	2	Some concerns	Suspected	No concerns	Major concerns	No concerns	No concerns	Very low
HAL vs QUE	1	Some concerns	Suspected	No concerns	Major concerns	No concerns	No concerns	Very low
HAL vs ZIP	1	Some concerns	Suspected	No concerns	Major concerns	No concerns	No concerns	Very low
HAL vs PLA	4	Some concerns	Suspected	No concerns	No concerns	Major concerns	No concerns	Very low
LIT vs OLA	1	No concerns	Suspected	No concerns	Major concerns	No concerns	No concerns	Low
LIT vs QUE	2	Some concerns	Suspected	No concerns	Major concerns	No concerns	No concerns	Very low
LIT vs PLA	2	Some concerns	Suspected	No concerns	No concerns	Major concerns	No concerns	Very low
OLA vs RIS	1	Some concerns	Suspected	No concerns	Major concerns	No concerns	Major concerns	Very low
OLA vs VALP	2	No concerns	Suspected	No concerns	Major concerns	No concerns	No concerns	Low
OLA vs PLA	5	Some concerns	Suspected	No concerns	No concerns	No concerns	No concerns	Low
OXC vs VALP	1	Some concerns	Suspected	No concerns	Major concerns	No concerns	No concerns	Very low
PAL vs QUE	1	No concerns	Suspected	No concerns	Major concerns	No concerns	No concerns	Low
PAL vs PLA	2	No concerns	Suspected	No concerns	No concerns	Major concerns	No concerns	Low
QUE vs PLA	4	No concerns	Suspected	No concerns	No concerns	Major concerns	No concerns	Low
RIS vs PLA	2	Some concerns	Suspected	No concerns	No concerns	No concerns	Major concerns	Very low
TAM vs PLA	2	No concerns	Suspected	No concerns	No concerns	Major concerns	No concerns	Low

VALP vs PLA	2	No concerns	Suspected	No concerns	Major concerns	No concerns	No concerns	Low
ZIP vs PLA	1	Some concerns	Suspected	No concerns	Major concerns	No concerns	No concerns	Very low
ARI vs ASE	0	Some concerns	Suspected	No concerns	Major concerns	No concerns	No concerns	Very low
ARI vs CARI	0	Some concerns	Suspected	No concerns	Major concerns	No concerns	No concerns	Very low
ARI vs ESL	0	Some concerns	Suspected	No concerns	Major concerns	No concerns	No concerns	Very low
ARI vs OLA	0	Some concerns	Suspected	No concerns	Major concerns	No concerns	No concerns	Very low
ARI vs OXC	0	Some concerns	Suspected	No concerns	Major concerns	No concerns	No concerns	Very low
ARI vs PAL	0	Some concerns	Suspected	No concerns	Major concerns	No concerns	No concerns	Very low
ARI vs QUE	0	Some concerns	Suspected	No concerns	Major concerns	No concerns	No concerns	Very low
ARI vs RIS	0	Some concerns	Suspected	No concerns	Major concerns	No concerns	No concerns	Very low
ARI vs TAM	0	Some concerns	Suspected	No concerns	Major concerns	No concerns	No concerns	Very low
ARI vs VALP	0	Some concerns	Suspected	No concerns	Major concerns	No concerns	No concerns	Very low
ARI vs ZIP	0	Some concerns	Suspected	No concerns	Major concerns	No concerns	No concerns	Very low
ASE vs CARI	0	Some concerns	Suspected	No concerns	Major concerns	No concerns	No concerns	Very low
ASE vs ESL	0	Some concerns	Suspected	No concerns	Major concerns	No concerns	No concerns	Very low
ASE vs HAL	0	Some concerns	Suspected	No concerns	Major concerns	No concerns	No concerns	Very low
ASE vs LIT	0	Some concerns	Suspected	No concerns	Major concerns	No concerns	No concerns	Very low
ASE vs OXC	0	Some concerns	Suspected	No concerns	Major concerns	No concerns	No concerns	Very low
ASE vs PAL	0	No concerns	Suspected	No concerns	Major concerns	No concerns	No concerns	Very low
ASE vs QUE	0	No concerns	Suspected	No concerns	Major concerns	No concerns	No concerns	Very low
ASE vs RIS	0	Some concerns	Suspected	No concerns	Major concerns	No concerns	No concerns	Very low
ASE vs TAM	0	No concerns	Suspected	No concerns	Major concerns	No concerns	No concerns	Very low
ASE vs VALP	0	No concerns	Suspected	No concerns	Major concerns	No concerns	No concerns	Very low
ASE vs ZIP	0	Some concerns	Suspected	No concerns	Major concerns	No concerns	No concerns	Very low
CARI vs ESL	0	Some concerns	Suspected	No concerns	Major concerns	No concerns	No concerns	Very low

CARI vs HAL	0	Some concerns	Suspected	No concerns	Major concerns	No concerns	No concerns	Very low
CARI vs LIT	0	Some concerns	Suspected	No concerns	Major concerns	No concerns	No concerns	Very low
CARI vs OLA	0	Some concerns	Suspected	No concerns	Major concerns	No concerns	No concerns	Very low
CARI vs OXC	0	Some concerns	Suspected	No concerns	Major concerns	No concerns	No concerns	Very low
CARI vs PAL	0	No concerns	Suspected	No concerns	Major concerns	No concerns	No concerns	Very low
CARI vs QUE	0	Some concerns	Suspected	No concerns	Major concerns	No concerns	No concerns	Very low
CARI vs RIS	0	Some concerns	Suspected	No concerns	Major concerns	No concerns	No concerns	Very low
CARI vs TAM	0	Some concerns	Suspected	No concerns	Major concerns	No concerns	No concerns	Very low
CARI vs VALP	0	Some concerns	Suspected	No concerns	Major concerns	No concerns	No concerns	Very low
CARI vs ZIP	0	Some concerns	Suspected	No concerns	Major concerns	No concerns	No concerns	Very low
ESL vs HAL	0	Some concerns	Suspected	No concerns	Major concerns	No concerns	No concerns	Very low
ESL vs LIT	0	Some concerns	Suspected	No concerns	Major concerns	No concerns	No concerns	Very low
ESL vs OLA	0	Some concerns	Suspected	No concerns	Major concerns	No concerns	No concerns	Very low
ESL vs OXC	0	Some concerns	Suspected	No concerns	Major concerns	No concerns	No concerns	Very low
ESL vs PAL	0	No concerns	Suspected	No concerns	Major concerns	No concerns	No concerns	Very low
ESL vs QUE	0	Some concerns	Suspected	No concerns	Major concerns	No concerns	No concerns	Very low
ESL vs RIS	0	Some concerns	Suspected	No concerns	Major concerns	No concerns	No concerns	Very low
ESL vs TAM	0	Some concerns	Suspected	No concerns	Major concerns	No concerns	No concerns	Very low
ESL vs VALP	0	Some concerns	Suspected	No concerns	Major concerns	No concerns	No concerns	Very low
ESL vs ZIP	0	Some concerns	Suspected	No concerns	Major concerns	No concerns	No concerns	Very low
HAL vs LIT	0	Some concerns	Suspected	No concerns	Major concerns	No concerns	No concerns	Very low
HAL vs OXC	0	Some concerns	Suspected	No concerns	Major concerns	No concerns	No concerns	Very low
HAL vs PAL	0	No concerns	Suspected	No concerns	Major concerns	No concerns	No concerns	Very low
HAL vs RIS	0	Some concerns	Suspected	No concerns	Major concerns	No concerns	No concerns	Very low
HAL vs TAM	0	No concerns	Suspected	No concerns	Major concerns	No concerns	No concerns	Very low

HAL vs VALP	0	No concerns	Suspected	No concerns	Major concerns	No concerns	No concerns	Very low
LIT vs OXC	0	No concerns	Suspected	No concerns	Major concerns	No concerns	No concerns	Very low
LIT vs PAL	0	No concerns	Suspected	No concerns	Major concerns	No concerns	No concerns	Very low
LIT vs RIS	0	Some concerns	Suspected	No concerns	Major concerns	No concerns	No concerns	Very low
LIT vs TAM	0	No concerns	Suspected	No concerns	Major concerns	No concerns	No concerns	Very low
LIT vs VALP	0	No concerns	Suspected	No concerns	Major concerns	No concerns	No concerns	Very low
LIT vs ZIP	0	Some concerns	Suspected	No concerns	Major concerns	No concerns	No concerns	Very low
OLA vs OXC	0	Some concerns	Suspected	No concerns	Major concerns	No concerns	No concerns	Very low
OLA vs PAL	0	No concerns	Suspected	No concerns	Major concerns	No concerns	No concerns	Very low
OLA vs QUE	0	No concerns	Suspected	No concerns	Major concerns	No concerns	No concerns	Very low
OLA vs TAM	0	No concerns	Suspected	No concerns	Major concerns	No concerns	No concerns	Very low
OLA vs ZIP	0	Some concerns	Suspected	No concerns	Major concerns	No concerns	No concerns	Very low
OXC vs PAL	0	No concerns	Suspected	No concerns	Major concerns	No concerns	No concerns	Very low
OXC vs QUE	0	No concerns	Suspected	No concerns	Major concerns	No concerns	No concerns	Very low
OXC vs RIS	0	Some concerns	Suspected	No concerns	Major concerns	No concerns	No concerns	Very low
OXC vs TAM	0	No concerns	Suspected	No concerns	Major concerns	No concerns	No concerns	Very low
OXC vs ZIP	0	Some concerns	Suspected	No concerns	Major concerns	No concerns	No concerns	Very low
OXC vs PLA	0	Some concerns	Suspected	No concerns	Major concerns	No concerns	No concerns	Very low
PAL vs RIS	0	No concerns	Suspected	No concerns	Major concerns	No concerns	No concerns	Very low
PAL vs TAM	0	No concerns	Suspected	No concerns	Major concerns	No concerns	No concerns	Very low
PAL vs VALP	0	No concerns	Suspected	No concerns	Major concerns	No concerns	No concerns	Very low
PAL vs ZIP	0	Some concerns	Suspected	No concerns	Major concerns	No concerns	No concerns	Very low
QUE vs RIS	0	Some concerns	Suspected	No concerns	Major concerns	No concerns	No concerns	Very low
QUE vs TAM	0	No concerns	Suspected	No concerns	Major concerns	No concerns	No concerns	Very low
QUE vs VALP	0	No concerns	Suspected	No concerns	Major concerns	No concerns	No concerns	Very low

QUE vs ZIP	0	Some concerns	Suspected	No concerns	Major concerns	No concerns	No concerns	Very low
RIS vs TAM	0	Some concerns	Suspected	No concerns	Major concerns	No concerns	No concerns	Very low
RIS vs VALP	0	Some concerns	Suspected	No concerns	Major concerns	No concerns	No concerns	Very low
RIS vs ZIP	0	Some concerns	Suspected	No concerns	Major concerns	No concerns	No concerns	Very low
TAM vs VALP	0	No concerns	Suspected	No concerns	Major concerns	No concerns	No concerns	Very low
TAM vs ZIP	0	Some concerns	Suspected	No concerns	Major concerns	No concerns	No concerns	Very low
VALP vs ZIP	0	Some concerns	Suspected	No concerns	Major concerns	No concerns	No concerns	Very low

Supplementary Appendix 6. Psychotic symptoms (N = 30, n = 7029)

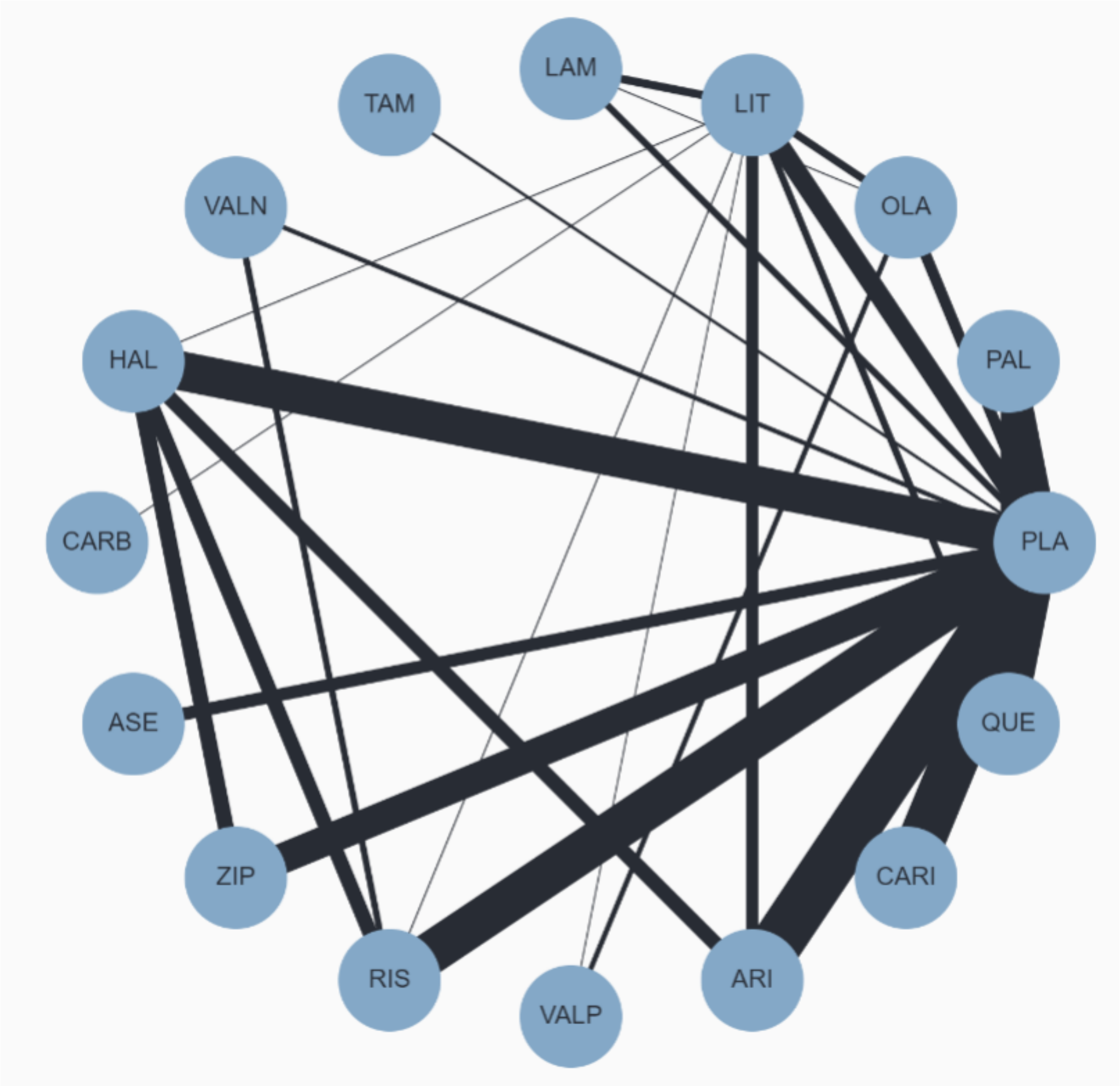

League table (standardized mean difference with 95% confidence interval)

ARI	0.094 (-0.434, 0.622)	-0.100 (-0.998, 0.798)	0.158 (-0.193, 0.508)	0.210 (-0.088, 0.507)	-0.315 (-0.768, 0.138)	-0.160 (-0.464, 0.142)	0.123 (-0.239, 0.485)	-0.036 (-0.421, 0.348)	0.088 (-0.240, 0.416)	0.161 (-0.154, 0.476)	**1.374 (0.644, 2.104)**	-0.211 (-0.747, 0.325)	-0.392 (-0.959, 0.174)	0.117 (-0.230, 0.465)	**-0.266 (-0.490, -0.041)**
	ASE	-0.194 (-1.196, 0.807)	0.063 (-0.485, 0.612)	0.115 (-0.418, 0.649)	-0.409 (-1.037, 0.218)	-0.255 (-0.791, 0.282)	0.029 (-0.534, 0.592)	-0.130 (-0.702, 0.442)	-0.006 (-0.544, 0.532)	0.067 (-0.464, 0.597)	**1.280 (0.437, 2.123)**	-0.305 (-0.989, 0.379)	-0.487 (-1.201, 0.227)	0.023 (-0.527, 0.574)	-0.360 (-0.838, 0.118)
		CARB	0.258 (-0.662, 1.178)	0.310 (-0.597, 1.217)	-0.215 (-1.149, 0.719)	-0.061 (-0.906, 0.785)	0.223 (-0.676, 1.122)	0.064 (-0.866, 0.994)	0.188 (-0.712, 1.088)	0.261 (-0.645, 1.167)	**1.474 (0.353, 2.595)**	-0.111 (-1.115, 0.894)	-0.293 (-1.284, 0.699)	0.217 (-0.703, 1.138)	-0.166 (-1.045, 0.714)
			CARI	0.052 (-0.306, 0.411)	-0.473 (-0.960, 0.014)	-0.318 (-0.681, 0.045)	-0.034 (-0.436, 0.367)	-0.194 (-0.607, 0.220)	-0.070 (-0.435, 0.296)	0.003 (-0.351, 0.358)	**1.217 (0.472, 1.962)**	-0.368 (-0.927, 0.190)	-0.550 (-1.145, 0.044)	-0.040 (-0.424, 0.343)	**-0.423 (-0.692, -0.154)**
				HAL	**-0.525 (-0.990, -0.059)**	**-0.370 (-0.699, -0.042)**	-0.086 (-0.463, 0.290)	-0.246 (-0.639, 0.147)	-0.122 (-0.462, 0.219)	-0.049 (-0.341, 0.244)	**1.165 (0.431, 1.899)**	-0.420 (-0.951, 0.110)	**-0.602 (-1.179, -0.026)**	-0.092 (-0.414, 0.230)	**-0.475 (-0.712, -0.238)**
					LAM	0.155 (-0.242, 0.551)	0.438 (-0.011, 0.887)	0.279 (-0.230, 0.788)	0.403 (-0.057, 0.863)	**0.476 (0.013, 0.939)**	**1.690 (0.885, 2.494)**	0.104 (-0.530, 0.738)	-0.077 (-0.702, 0.547)	0.432 (-0.056, 0.921)	0.050 (-0.357, 0.456)
						LIT	0.284 (-0.023, 0.590)	0.124 (-0.263, 0.512)	0.249 (-0.059, 0.556)	0.322 (-0.003, 0.646)	**1.535 (0.799, 2.271)**	-0.050 (-0.593, 0.492)	-0.232 (-0.750, 0.286)	0.278 (-0.086, 0.642)	-0.105 (-0.349, 0.139)
							OLA	-0.159 (-0.588, 0.270)	-0.035 (-0.407, 0.337)	0.038 (-0.335, 0.410)	**1.251 (0.495, 2.007)**	-0.334 (-0.905, 0.237)	**-0.516 (-0.992, -0.040)**	-0.006 (-0.409, 0.398)	**-0.389 (-0.687, -0.090)**

								PAL	0.124 (-0.225, 0.473)	0.197 (-0.192, 0.586)	**1.411 (0.648, 2.173)**	-0.175 (-0.756, 0.407)	-0.356 (-0.969, 0.256)	0.154 (-0.263, 0.570)	-0.229 (-0.544, 0.085)
									QUE	0.073 (-0.263, 0.409)	**1.286 (0.549, 2.024)**	-0.299 (-0.846, 0.249)	-0.481 (-1.052, 0.091)	0.029 (-0.339, 0.398)	**-0.354 (-0.601, -0.106)**
										RIS	**1.213 (0.482, 1.945)**	-0.372 (-0.850, 0.106)	-0.554 (-1.128, 0.021)	-0.044 (-0.393, 0.306)	**-0.426 (-0.657, -0.196)**
											TAM	**-1.585 (-2.435, -0.736)**	**-1.767 (-2.641, -0.893)**	**-1.257 (-2.003, -0.510)**	**-1.640 (-2.335, -0.945)**
												VALN	-0.182 (-0.901, 0.538)	0.328 (-0.229, 0.886)	-0.055 (-0.544, 0.435)
													VALP	0.510 (-0.086, 1.105)	0.127 (-0.403, 0.657)
														ZIP	**-0.383 (-0.656, -0.110)**
															PLA

The outcome was synthesized the following psychosis-rating scale scores.

Positive and Negative Syndrome Scale[1] and Brief Psychiatric Rating Scale[2]

1. Kay SR, Opler LA, Lindenmayer JP. The Positive and Negative Syndrome Scale (PANSS): rationale and standardisation. The British journal of psychiatry

Supplement 1989; (7): 59-67.
2. Overall JE, Gorham DR. The Brief Psychiatric Rating Scale. Psychol Rep 1962; 10: 799–812.

Study	Psychosis rating scale
Berk 1999	BPRS
Berwaerts 2012	PANSS
Bowden 2000	BPRS
Bowden 2005	PANSS
Calabrese 2015	PANSS
Durgam 2015	BPRS
El Mallakh 2010	PANSS
Freeman 1992	BPRS
Hirschfeld 2004	PANSS
Keck 2003 ZIP	PANSS
Keck 2009	PANSS
Khanna 2005	PANSS
Landbloom 2016	PANSS
Lerer 1987	BPRS
Li 2008	PANSS
McIntyre 2005	PANSS
Niufan 2008	BPRS
Potkin 2005	PANSS
Sachs 2006	PANSS
Sachs 2015	PANSS
Segal 1998	BPRS

Smulevich 2005	BPRS
Tohen 1999	PANSS
Tohen 2000	PANSS
Vieta 2010 ZIP	PANSS
Vieta 2010 PAL	PANSS
Weiser 2017	PANSS
Yildiz 2008	PANSS
Young 2009	PANSS
Zajecka 2002	BPRS

BPRS: Brief Psychiatric Rating Scale, PANSS: Positive and Negative Syndrome Scale

Evaluation of heterogeneity and inconsistency

Between study variance (τ^2)	Heterogeneity assessment	Random-effects design-by-treatment interaction model		
		Q	df	p
0.043	Low to moderate	46.164	17	0.000

Incoherence

	NMA, SMD (95% CI)	Direct, SMD (95% CI)	I^2	Indirect, SMD (95% CI)	Inconsistency measures	
					Difference of SMD	P value
ARI vs HAL	0.210 (-0.088, 0.507)	0.047 (-0.414, 0.509)	na	0.325 (-0.064, 0.715)	-0.278 (-0.882, 0.326)	0.367
ARI vs LIT	-0.160 (-0.464, 0.142)	-0.152 (-0.625, 0.320)	na	-0.166 (-0.561, 0.229)	0.014 (-0.602, 0.630)	0.964
ARI vs PLA	-0.266 (-0.490, -0.041)	-0.240 (-0.477, -0.004)	22.1%	-0.493 (-1.205, 0.219)	0.253 (-0.498, 1.003)	0.509
ASE vs PLA		-0.360 (-0.838, 0.118)	na			
CARB vs LIT		-0.061 (-0.906, 0.785)	na			
CARI vs PLA		-0.423 (-0.692, -0.154)	67.7%			
HAL vs LIT	-0.370 (-0.699, -0.042)	-2.043 (-3.036, -1.050)	na	-0.165 (-0.513, 0.184)	-1.879 (-2.931, -0.827)	0.001
HAL vs RIS	-0.049 (-0.341, 0.244)	-0.150 (-0.559, 0.259)	76.3%	0.058 (-0.361, 0.476)	-0.207 (-0.793, 0.378)	0.487
HAL vs ZIP	-0.092 (-0.414, 0.230)	-0.226 (-0.685, 0.233)	na	0.038 (-0.415, 0.491)	-0.264 (-0.909, 0.380)	0.421
HAL vs PLA	-0.475 (-0.712, -0.238)	-0.327 (-0.600, -0.055)	40.8%	-0.934 (-1.414, -0.453)	0.606 (0.054, 1.159)	0.032
LAM vs LIT	0.155 (-0.242, 0.551)	0.275 (-0.164, 0.714)	0.0%	-0.384 (-1.311, 0.544)	0.659 (-0.367, 1.685)	0.208
LAM vs OLA	0.438 (-0.011, 0.887)	0.268 (-0.559, 1.094)	na	0.510 (-0.025, 1.045)	-0.242 (-1.227, 0.742)	0.630
LAM vs PLA	0.050 (-0.357, 0.456)	-0.051 (-0.568, 0.467)	na	0.211 (-0.445, 0.867)	-0.261 (-1.097, 0.574)	0.540
LIT vs OLA	0.284 (-0.023, 0.590)	0.238 (-0.205, 0.682)	0.0%	0.325 (-0.099, 0.749)	-0.086 (-0.700, 0.527)	0.782
LIT vs QUE	0.249 (-0.059, 0.556)	0.286 (-0.230, 0.803)	na	0.228 (-0.155, 0.611)	0.059 (-0.584, 0.702)	0.858
LIT vs RIS	0.322 (-0.003, 0.646)	1.265 (0.373, 2.157)	na	0.177 (-0.171, 0.526)	1.087 (0.130, 2.045)	0.026
LIT vs VALP	-0.232 (-0.750, 0.286)	-0.798 (-1.686, 0.090)	na	0.059 (-0.578, 0.697)	-0.857 (-1.950, 0.236)	0.124

LIT vs PLA	-0.105 (-0.349, 0.139)	-0.219 (-0.566, 0.129)	0.0%	0.005 (-0.337, 0.347)	-0.224 (-0.712, 0.264)	0.368
OLA vs VALP	-0.516 (-0.992, -0.040)	-0.298 (-0.849, 0.253)	na	-1.155 (-2.099, -0.211)	0.857 (-0.236, 1.950)	0.124
OLA vs PLA	-0.389 (-0.687, -0.090)	-0.534 (-0.919, -0.148)	0.0%	-0.173 (-0.643, 0.298)	-0.361 (-0.970, 0.247)	0.245
PAL vs QUE	0.124 (-0.225, 0.473)	-0.098 (-0.556, 0.359)	na	0.434 (-0.106, 0.974)	-0.532 (-1.240, 0.175)	0.141
PAL vs PLA	-0.229 (-0.544, 0.085)	-0.167 (-0.501, 0.168)	76.1%	-0.713 (-1.639, 0.214)	0.546 (-0.439, 1.531)	0.277
QUE vs PLA	-0.354 (-0.601, -0.106)	-0.389 (-0.671, -0.107)	0.0%	-0.234 (-0.752, 0.284)	-0.155 (-0.745, 0.434)	0.606
RIS vs VALN	-0.372 (-0.850, 0.106)	-0.078 (-0.602, 0.447)	na	-1.834 (-3.002, -0.665)	1.756 (0.475, 3.037)	0.007
RIS vs PLA	-0.426 (-0.657, -0.196)	-0.384 (-0.632, -0.137)	88.3%	-0.699 (-1.327, -0.070)	0.315 (-0.361, 0.990)	0.361
TAM vs PLA		-1.640 (-2.335, -0.945)	na			
VALN vs PLA	-0.055 (-0.544, 0.435)	0.388 (-0.198, 0.974)	na	-1.072 (-1.961, -0.183)	1.460 (0.395, 2.525)	0.007
ZIP vs PLA	-0.383 (-0.656, -0.110)	-0.406 (-0.693, -0.119)	0.0%	-0.155 (-1.052, 0.742)	-0.251 (-1.193, 0.690)	0.601

Forest plot (vs placebo, the numbers are standardized mean difference with 95% confidence interval)

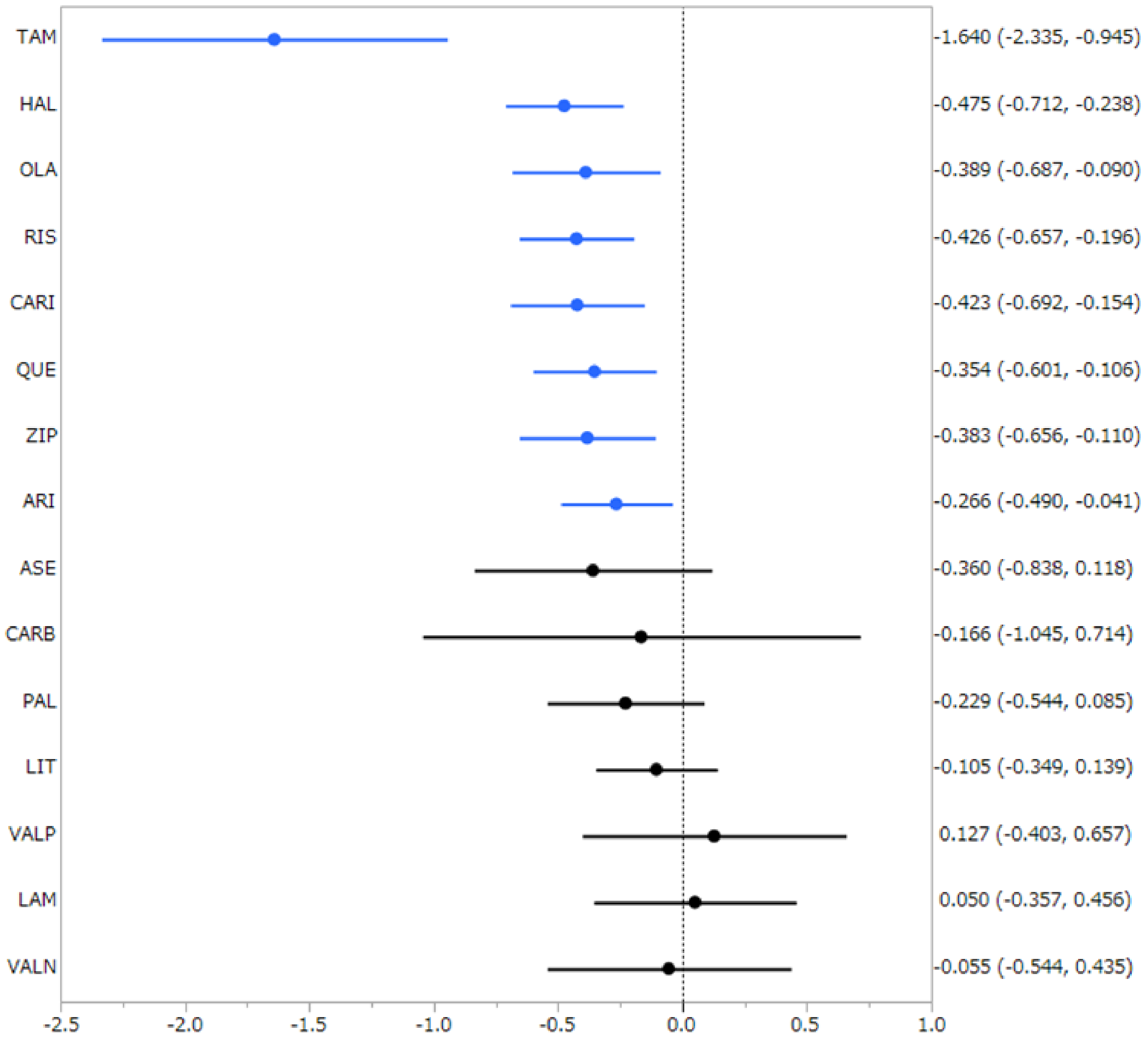

P-score

TAM	1.000
HAL	0.700
OLA	0.696
RIS	0.664
CARI	0.642
QUE	0.641
ZIP	0.560
ARI	0.504
ASE	0.500
CARB	0.497
PAL	0.429
LIT	0.384
VALP	0.227
LAM	0.225
VALN	0.215

Funnel plot (only double-blind, placebo-controlled trials)

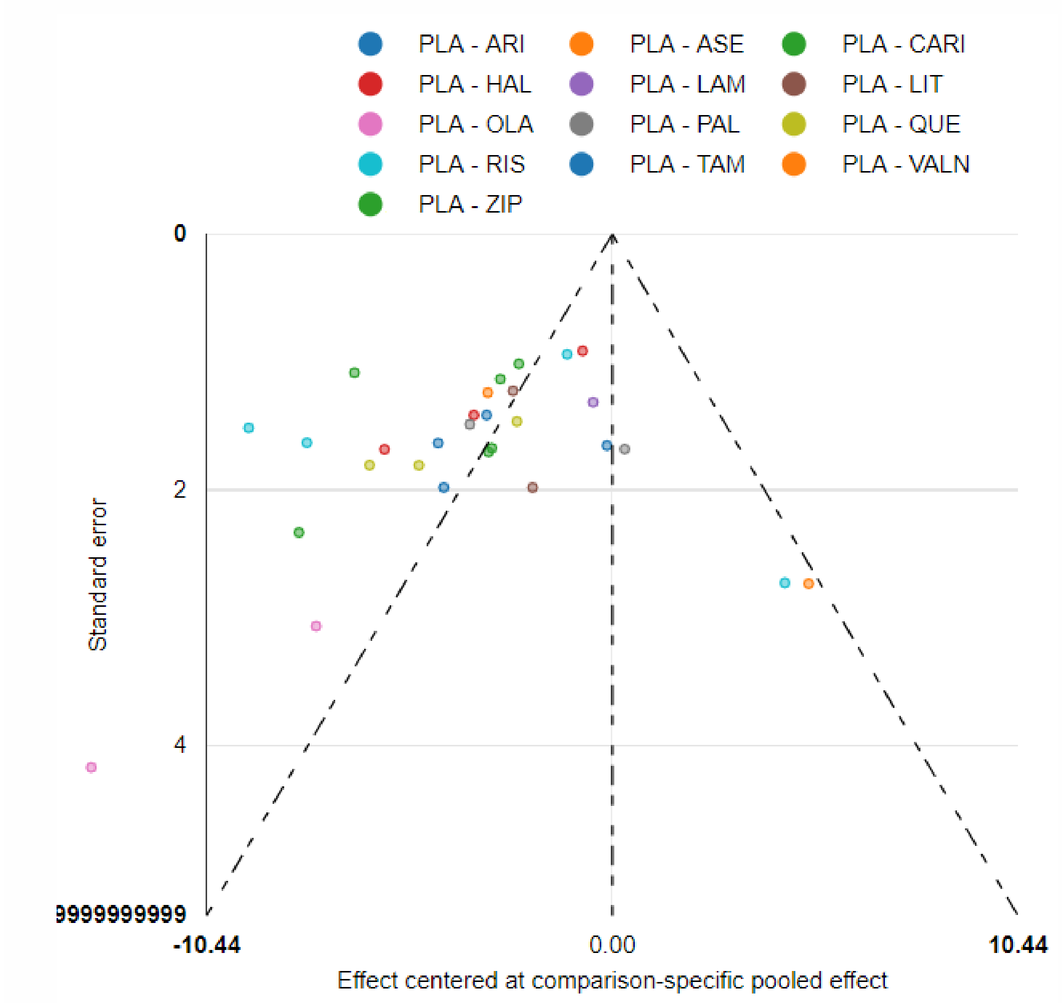

Sensitivity analysis excluding BPRS data (i.e., only PANSS data)

Evaluation of heterogeneity and inconsistency

Between study variance (τ^2)	Heterogeneity assessment	Random-effects design-by-treatment interaction model		
		Q	df	p
0.002	Low	15.459	8	0.051

ARI	0.102 (-0.396, 0.599)	0.229 (-0.141, 0.600)	0.174 (-0.142, 0.491)	-0.161 (-0.506, 0.184)	0.275 (-0.151, 0.701)	-0.029 (-0.391, 0.332)	0.092 (-0.218, 0.402)	0.203 (-0.147, 0.552)	**1.382 (0.674, 2.090)**	-0.176 (-0.700, 0.347)	0.113 (-0.215, 0.441)	**-0.258 (-0.473, -0.044)**
	ASE	0.128 (-0.413, 0.668)	0.073 (-0.457, 0.603)	-0.263 (-0.815, 0.290)	0.174 (-0.406, 0.754)	-0.131 (-0.668, 0.406)	-0.009 (-0.517, 0.499)	0.101 (-0.425, 0.628)	**1.280 (0.470, 2.091)**	-0.278 (-0.933, 0.377)	0.012 (-0.507, 0.530)	-0.360 (-0.808, 0.089)
		CARI	-0.055 (-0.468, 0.358)	-0.391 (-0.832, 0.051)	0.046 (-0.430, 0.521)	-0.259 (-0.680, 0.163)	-0.137 (-0.522, 0.247)	-0.027 (-0.435, 0.382)	**1.152 (0.413, 1.892)**	-0.406 (-0.970, 0.159)	-0.116 (-0.515, 0.282)	**-0.488 (-0.789, -0.186)**
			HAL	-0.336 (-0.754, 0.082)	0.100 (-0.363, 0.564)	-0.204 (-0.611, 0.203)	-0.082 (-0.449, 0.284)	0.028 (-0.366, 0.422)	**1.207 (0.476, 1.939)**	-0.351 (-0.905, 0.204)	-0.062 (-0.391, 0.268)	**-0.433 (-0.715, -0.151)**
				LIT	0.436 (-0.053, 0.925)	0.132 (-0.288, 0.552)	0.254 (-0.086, 0.594)	0.364 (-0.060, 0.788)	**1.543 (0.795, 2.291)**	-0.015 (-0.591, 0.561)	0.274 (-0.138, 0.686)	-0.097 (-0.419, 0.225)
					OLA	-0.304 (-0.775, 0.167)	-0.183 (-0.621, 0.255)	-0.072 (-0.532, 0.387)	**1.107 (0.338, 1.875)**	-0.451 (-1.054, 0.151)	-0.162 (-0.613, 0.289)	**-0.533 (-0.901, -0.166)**
						PAL	0.122 (-0.207, 0.450)	0.232 (-0.171, 0.635)	**1.411 (0.675, 2.147)**	-0.147 (-0.708, 0.414)	0.142 (-0.251, 0.536)	-0.229 (-0.524, 0.066)
							QUE	0.110 (-0.254, 0.475)	**1.289 (0.574, 2.005)**	-0.268 (-0.802, 0.265)	0.021 (-0.332, 0.373)	**-0.351 (-0.589, -0.112)**
								RIS	**1.179 (0.450, 1.908)**	-0.379 (-0.838, 0.081)	-0.090 (-0.469, 0.290)	**-0.461 (-0.736, -0.185)**

									TAM	**-1.558 (-2.385, -0.731)**	**-1.269 (-1.992, -0.545)**	**-1.640 (-2.315, -0.965)**
										VALN	0.289 (-0.255, 0.833)	-0.082 (-0.559, 0.395)
											ZIP	**-0.371 (-0.632, -0.110)**
												PLA

CINeMA confidence rating

Comparison	Number of studies	Within-study bias	Reporting bias	Indirectness	Imprecision	Heterogeneity	Incoherence	Confidence rating
ARI vs HAL	1	Some concerns	Suspected	No concerns	Major concerns	No concerns	No concerns	Very low
ARI vs LIT	1	Some concerns	Suspected	No concerns	Major concerns	No concerns	No concerns	Very low
ARI vs PLA	4	Some concerns	Suspected	No concerns	No concerns	Major concerns	No concerns	Very low
ASE vs PLA	1	No concerns	Suspected	No concerns	Major concerns	No concerns	Major concerns	Very low
CARB vs LIT	1	No concerns	Suspected	No concerns	Major concerns	No concerns	Major concerns	Very low
CARI vs PLA	3	Some concerns	Suspected	No concerns	No concerns	Major concerns	Major concerns	Very low
HAL vs LIT	1	Some concerns	Suspected	No concerns	No concerns	Major concerns	Major concerns	Very low
HAL vs RIS	2	Some concerns	Suspected	No concerns	Major concerns	No concerns	No concerns	Very low
HAL vs ZIP	1	Some concerns	Suspected	No concerns	Major concerns	No concerns	No concerns	Very low
HAL vs PLA	3	Some concerns	Suspected	No concerns	No concerns	Major concerns	No concerns	Very low
LAM vs LIT	2	Some concerns	Suspected	No concerns	Major concerns	No concerns	No concerns	Very low
LAM vs OLA	1	Some concerns	Suspected	No concerns	Major concerns	No concerns	No concerns	Very low
LAM vs PLA	1	Some concerns	Suspected	No concerns	Major concerns	No concerns	No concerns	Very low
LIT vs OLA	2	Some concerns	Suspected	No concerns	Major concerns	No concerns	No concerns	Very low
LIT vs QUE	1	Some concerns	Suspected	No concerns	Major concerns	No concerns	No concerns	Very low
LIT vs RIS	1	Some concerns	Suspected	No concerns	Major concerns	No concerns	Major concerns	Very low
LIT vs VALP	1	Some concerns	Suspected	No concerns	Major concerns	No concerns	No concerns	Very low
LIT vs PLA	2	Some concerns	Suspected	No concerns	Major concerns	No concerns	No concerns	Very low
OLA vs VALP	1	Some concerns	Suspected	No concerns	No concerns	Major concerns	No concerns	Very low
OLA vs PLA	2	Some concerns	Suspected	No concerns	No concerns	Major concerns	No concerns	Very low
PAL vs QUE	1	No concerns	Suspected	No concerns	Major concerns	No concerns	No concerns	Low
PAL vs PLA	2	No concerns	Suspected	No concerns	Major concerns	No concerns	No concerns	Low
QUE vs PLA	3	No concerns	Suspected	No concerns	No concerns	Major concerns	No concerns	Low

RIS vs VALN	1	No concerns	Suspected	No concerns	Major concerns	No concerns	Major concerns	Very low
RIS vs PLA	4	Some concerns	Suspected	No concerns	No concerns	Major concerns	No concerns	Very low
TAM vs PLA	1	No concerns	Suspected	No concerns	No concerns	No concerns	Major concerns	Low
VALN vs PLA	1	No concerns	Suspected	No concerns	Major concerns	No concerns	Major concerns	Very low
ZIP vs PLA	3	Some concerns	Suspected	No concerns	No concerns	Major concerns	No concerns	Very low
ARI vs ASE	0	Some concerns	Suspected	No concerns	Major concerns	No concerns	Major concerns	Very low
ARI vs CARB	0	Some concerns	Suspected	No concerns	Major concerns	No concerns	Major concerns	Very low
ARI vs CARI	0	Some concerns	Suspected	No concerns	Major concerns	No concerns	Major concerns	Very low
ARI vs LAM	0	Some concerns	Suspected	No concerns	Major concerns	No concerns	Major concerns	Very low
ARI vs OLA	0	Some concerns	Suspected	No concerns	Major concerns	No concerns	Major concerns	Very low
ARI vs PAL	0	Some concerns	Suspected	No concerns	Major concerns	No concerns	Major concerns	Very low
ARI vs QUE	0	Some concerns	Suspected	No concerns	Major concerns	No concerns	Major concerns	Very low
ARI vs RIS	0	Some concerns	Suspected	No concerns	Major concerns	No concerns	Major concerns	Very low
ARI vs TAM	0	Some concerns	Suspected	No concerns	No concerns	No concerns	Major concerns	Very low
ARI vs VALN	0	Some concerns	Suspected	No concerns	Major concerns	No concerns	Major concerns	Very low
ARI vs VALP	0	Some concerns	Suspected	No concerns	Major concerns	No concerns	Major concerns	Very low
ARI vs ZIP	0	Some concerns	Suspected	No concerns	Major concerns	No concerns	Major concerns	Very low
ASE vs CARB	0	No concerns	Suspected	No concerns	Major concerns	No concerns	Major concerns	Very low
ASE vs CARI	0	Some concerns	Suspected	No concerns	Major concerns	No concerns	Major concerns	Very low
ASE vs HAL	0	Some concerns	Suspected	No concerns	Major concerns	No concerns	Major concerns	Very low
ASE vs LAM	0	Some concerns	Suspected	No concerns	Major concerns	No concerns	Major concerns	Very low
ASE vs LIT	0	Some concerns	Suspected	No concerns	Major concerns	No concerns	Major concerns	Very low
ASE vs OLA	0	Some concerns	Suspected	No concerns	Major concerns	No concerns	Major concerns	Very low
ASE vs PAL	0	No concerns	Suspected	No concerns	Major concerns	No concerns	Major concerns	Very low
ASE vs QUE	0	No concerns	Suspected	No concerns	Major concerns	No concerns	Major concerns	Very low

ASE vs RIS	0	No concerns	Suspected	No concerns	Major concerns	No concerns	Major concerns	Very low
ASE vs TAM	0	No concerns	Suspected	No concerns	No concerns	No concerns	Major concerns	Very low
ASE vs VALN	0	No concerns	Suspected	No concerns	Major concerns	No concerns	Major concerns	Very low
ASE vs VALP	0	Some concerns	Suspected	No concerns	Major concerns	No concerns	Major concerns	Very low
ASE vs ZIP	0	No concerns	Suspected	No concerns	Major concerns	No concerns	Major concerns	Very low
CARB vs CARI	0	Some concerns	Suspected	No concerns	Major concerns	No concerns	Major concerns	Very low
CARB vs HAL	0	Some concerns	Suspected	No concerns	Major concerns	No concerns	Major concerns	Very low
CARB vs LAM	0	Some concerns	Suspected	No concerns	Major concerns	No concerns	Major concerns	Very low
CARB vs OLA	0	No concerns	Suspected	No concerns	Major concerns	No concerns	Major concerns	Very low
CARB vs PAL	0	No concerns	Suspected	No concerns	Major concerns	No concerns	Major concerns	Very low
CARB vs QUE	0	No concerns	Suspected	No concerns	Major concerns	No concerns	Major concerns	Very low
CARB vs RIS	0	Some concerns	Suspected	No concerns	Major concerns	No concerns	Major concerns	Very low
CARB vs TAM	0	No concerns	Suspected	No concerns	No concerns	No concerns	Major concerns	Very low
CARB vs VALN	0	No concerns	Suspected	No concerns	Major concerns	No concerns	Major concerns	Very low
CARB vs VALP	0	Some concerns	Suspected	No concerns	Major concerns	No concerns	Major concerns	Very low
CARB vs ZIP	0	Some concerns	Suspected	No concerns	Major concerns	No concerns	Major concerns	Very low
CARB vs PLA	0	Some concerns	Suspected	No concerns	Major concerns	No concerns	Major concerns	Very low
CARI vs HAL	0	Some concerns	Suspected	No concerns	Major concerns	No concerns	Major concerns	Very low
CARI vs LAM	0	Some concerns	Suspected	No concerns	Major concerns	No concerns	Major concerns	Very low
CARI vs LIT	0	Some concerns	Suspected	No concerns	Major concerns	No concerns	Major concerns	Very low
CARI vs OLA	0	Some concerns	Suspected	No concerns	Major concerns	No concerns	Major concerns	Very low
CARI vs PAL	0	Some concerns	Suspected	No concerns	Major concerns	No concerns	Major concerns	Very low
CARI vs QUE	0	Some concerns	Suspected	No concerns	Major concerns	No concerns	Major concerns	Very low
CARI vs RIS	0	Some concerns	Suspected	No concerns	Major concerns	No concerns	Major concerns	Very low
CARI vs TAM	0	Some concerns	Suspected	No concerns	No concerns	No concerns	Major concerns	Very low

CARI vs VALN	0	Some concerns	Suspected	No concerns	Major concerns	No concerns	Major concerns	Very low
CARI vs VALP	0	Some concerns	Suspected	No concerns	Major concerns	No concerns	Major concerns	Very low
CARI vs ZIP	0	Some concerns	Suspected	No concerns	Major concerns	No concerns	Major concerns	Very low
HAL vs LAM	0	Some concerns	Suspected	No concerns	No concerns	Major concerns	Major concerns	Very low
HAL vs OLA	0	Some concerns	Suspected	No concerns	Major concerns	No concerns	Major concerns	Very low
HAL vs PAL	0	Some concerns	Suspected	No concerns	Major concerns	No concerns	Major concerns	Very low
HAL vs QUE	0	Some concerns	Suspected	No concerns	Major concerns	No concerns	Major concerns	Very low
HAL vs TAM	0	Some concerns	Suspected	No concerns	No concerns	No concerns	Major concerns	Very low
HAL vs VALN	0	Some concerns	Suspected	No concerns	Major concerns	No concerns	Major concerns	Very low
HAL vs VALP	0	Some concerns	Suspected	No concerns	No concerns	Major concerns	Major concerns	Very low
LAM vs PAL	0	Some concerns	Suspected	No concerns	Major concerns	No concerns	Major concerns	Very low
LAM vs QUE	0	Some concerns	Suspected	No concerns	Major concerns	No concerns	Major concerns	Very low
LAM vs RIS	0	Some concerns	Suspected	No concerns	No concerns	Major concerns	Major concerns	Very low
LAM vs TAM	0	Some concerns	Suspected	No concerns	No concerns	No concerns	Major concerns	Very low
LAM vs VALN	0	Some concerns	Suspected	No concerns	Major concerns	No concerns	Major concerns	Very low
LAM vs VALP	0	Some concerns	Suspected	No concerns	Major concerns	No concerns	Major concerns	Very low
LAM vs ZIP	0	Some concerns	Suspected	No concerns	Major concerns	No concerns	Major concerns	Very low
LIT vs PAL	0	Some concerns	Suspected	No concerns	Major concerns	No concerns	Major concerns	Very low
LIT vs TAM	0	Some concerns	Suspected	No concerns	No concerns	No concerns	Major concerns	Very low
LIT vs VALN	0	Some concerns	Suspected	No concerns	Major concerns	No concerns	Major concerns	Very low
LIT vs ZIP	0	Some concerns	Suspected	No concerns	Major concerns	No concerns	Major concerns	Very low
OLA vs PAL	0	No concerns	Suspected	No concerns	Major concerns	No concerns	Major concerns	Very low
OLA vs QUE	0	Some concerns	Suspected	No concerns	Major concerns	No concerns	Major concerns	Very low
OLA vs RIS	0	Some concerns	Suspected	No concerns	Major concerns	No concerns	Major concerns	Very low
OLA vs TAM	0	Some concerns	Suspected	No concerns	No concerns	No concerns	Major concerns	Very low

OLA vs VALN	0	Some concerns	Suspected	No concerns	Major concerns	No concerns	Major concerns	Very low
OLA vs ZIP	0	Some concerns	Suspected	No concerns	Major concerns	No concerns	Major concerns	Very low
PAL vs RIS	0	No concerns	Suspected	No concerns	Major concerns	No concerns	Major concerns	Very low
PAL vs TAM	0	No concerns	Suspected	No concerns	No concerns	No concerns	Major concerns	Very low
PAL vs VALN	0	No concerns	Suspected	No concerns	Major concerns	No concerns	Major concerns	Very low
PAL vs VALP	0	Some concerns	Suspected	No concerns	Major concerns	No concerns	Major concerns	Very low
PAL vs ZIP	0	No concerns	Suspected	No concerns	Major concerns	No concerns	Major concerns	Very low
QUE vs RIS	0	Some concerns	Suspected	No concerns	Major concerns	No concerns	Major concerns	Very low
QUE vs TAM	0	No concerns	Suspected	No concerns	No concerns	No concerns	Major concerns	Very low
QUE vs VALN	0	No concerns	Suspected	No concerns	Major concerns	No concerns	Major concerns	Very low
QUE vs VALP	0	Some concerns	Suspected	No concerns	Major concerns	No concerns	Major concerns	Very low
QUE vs ZIP	0	Some concerns	Suspected	No concerns	Major concerns	No concerns	Major concerns	Very low
RIS vs TAM	0	No concerns	Suspected	No concerns	No concerns	No concerns	Major concerns	Very low
RIS vs VALP	0	Some concerns	Suspected	No concerns	Major concerns	No concerns	Major concerns	Very low
RIS vs ZIP	0	Some concerns	Suspected	No concerns	Major concerns	No concerns	Major concerns	Very low
TAM vs VALN	0	No concerns	Suspected	No concerns	No concerns	No concerns	Major concerns	Very low
TAM vs VALP	0	Some concerns	Suspected	No concerns	No concerns	No concerns	Major concerns	Very low
TAM vs ZIP	0	No concerns	Suspected	No concerns	No concerns	No concerns	Major concerns	Very low
VALN vs VALP	0	Some concerns	Suspected	No concerns	Major concerns	No concerns	Major concerns	Very low
VALN vs ZIP	0	No concerns	Suspected	No concerns	Major concerns	No concerns	Major concerns	Very low
VALP vs ZIP	0	Some concerns	Suspected	No concerns	Major concerns	No concerns	Major concerns	Very low
VALP vs PLA	0	Some concerns	Suspected	No concerns	Major concerns	No concerns	Major concerns	Very low

Supplementary Appendix 7. Discontinuation due to adverse events (N = 52, n = 14629)

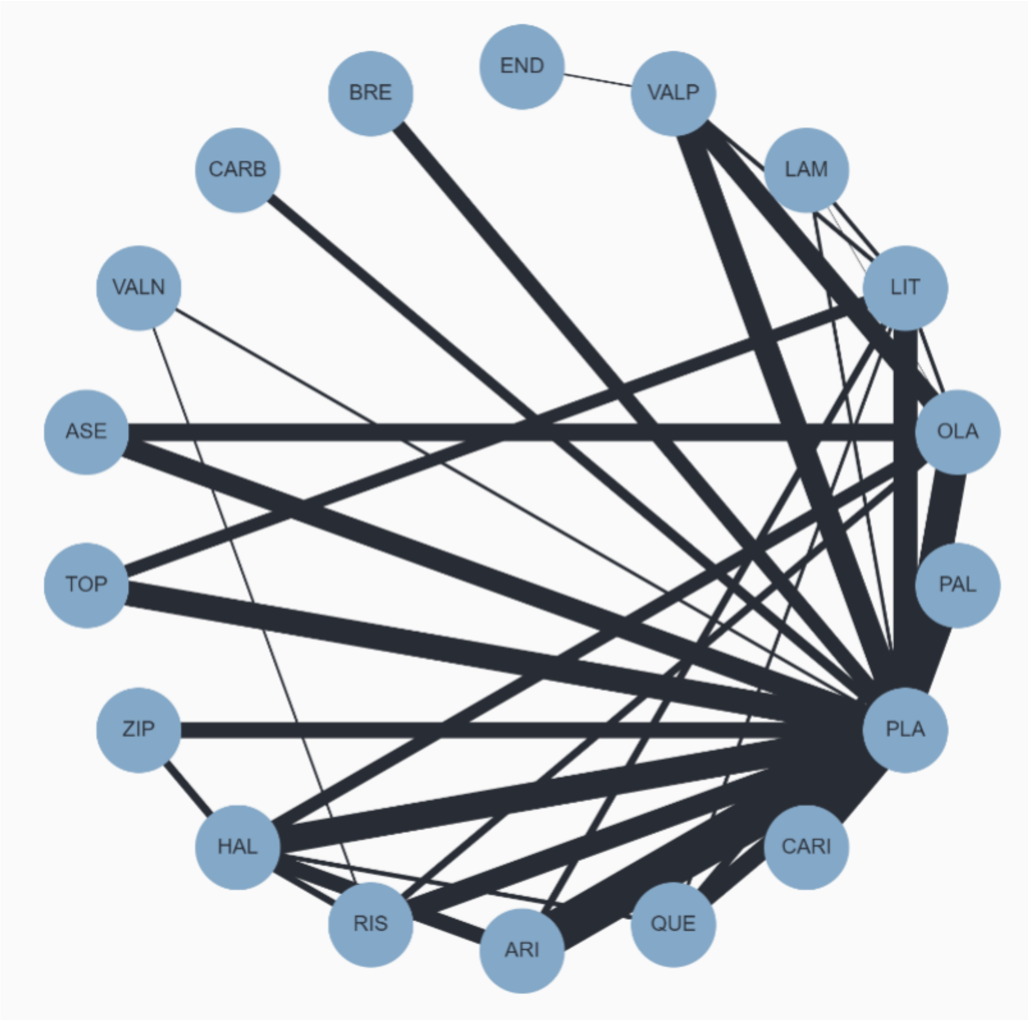

League table (risk ratio with 95% confidence interval)

ARI	0.640 (0.341, 1.199)	1.377 (0.491, 3.864)	0.613 (0.254, 1.480)	0.731 (0.377, 1.416)	0.670 (0.056, 8.062)	0.649 (0.412, 1.023)	0.612 (0.239, 1.571)	0.677 (0.389, 1.177)	1.115 (0.661, 1.881)	0.972 (0.416, 2.267)	**2.248 (1.040, 4.860)**	0.843 (0.430, 1.653)	1.194 (0.520, 2.745)	0.746 (0.134, 4.151)	0.993 (0.522, 1.892)	0.954 (0.448, 2.030)	1.212 (0.850, 1.728)
	ASE	2.153 (0.713, 6.495)	0.959 (0.366, 2.516)	1.143 (0.530, 2.466)	1.048 (0.085, 12.865)	1.016 (0.539, 1.913)	0.957 (0.342, 2.679)	1.058 (0.520, 2.155)	1.743 (0.984, 3.090)	1.520 (0.596, 3.872)	**3.515 (1.470, 8.406)**	1.318 (0.615, 2.825)	1.868 (0.736, 4.739)	1.166 (0.201, 6.769)	1.553 (0.757, 3.186)	1.492 (0.629, 3.540)	**1.896 (1.117, 3.218)**
		BRE	0.445 (0.126, 1.572)	0.531 (0.174, 1.625)	0.487 (0.034, 6.894)	0.472 (0.166, 1.345)	0.445 (0.119, 1.661)	0.492 (0.166, 1.459)	0.810 (0.281, 2.330)	0.706 (0.204, 2.438)	1.633 (0.494, 5.396)	0.612 (0.197, 1.901)	0.868 (0.251, 2.994)	0.542 (0.078, 3.772)	0.722 (0.236, 2.211)	0.693 (0.210, 2.288)	0.881 (0.334, 2.321)
			CARB	1.192 (0.447, 3.178)	1.093 (0.082, 14.646)	1.059 (0.431, 2.602)	0.998 (0.300, 3.324)	1.103 (0.429, 2.841)	1.818 (0.732, 4.514)	1.584 (0.519, 4.840)	**3.665 (1.260, 10.659)**	1.374 (0.507, 3.725)	1.947 (0.638, 5.945)	1.216 (0.188, 7.848)	1.620 (0.607, 4.325)	1.556 (0.536, 4.518)	1.977 (0.882, 4.427)
				CARI	0.917 (0.073, 11.501)	0.888 (0.448, 1.762)	0.837 (0.292, 2.400)	0.926 (0.439, 1.950)	1.525 (0.758, 3.068)	1.329 (0.513, 3.447)	**3.075 (1.257, 7.524)**	1.153 (0.513, 2.591)	1.634 (0.631, 4.233)	1.020 (0.174, 5.998)	1.359 (0.616, 2.996)	1.305 (0.534, 3.188)	1.658 (0.949, 2.897)
					END	0.969 (0.081, 11.641)	0.913 (0.067, 12.446)	1.010 (0.083, 12.277)	1.663 (0.142, 19.459)	1.450 (0.109, 19.213)	3.353 (0.259, 43.402)	1.258 (0.101, 15.655)	1.782 (0.135, 23.446)	1.113 (0.056, 21.935)	1.482 (0.134, 16.377)	1.424 (0.110, 18.359)	1.809 (0.153, 21.317)
						HAL	0.943 (0.359, 2.474)	1.042 (0.567, 1.913)	**1.717 (1.058, 2.784)**	1.496 (0.634, 3.532)	**3.461 (1.622, 7.383)**	1.298 (0.669, 2.519)	1.839 (0.779, 4.343)	1.148 (0.205, 6.422)	1.530 (0.807, 2.899)	1.469 (0.739, 2.919)	**1.867 (1.255, 2.776)**
							LAM	1.106 (0.456, 2.678)	1.821 (0.696, 4.765)	1.587 (0.488, 5.160)	**3.671 (1.191, 11.320)**	1.377 (0.477, 3.975)	1.951 (0.631, 6.036)	1.218 (0.182, 8.163)	1.623 (0.582, 4.524)	1.559 (0.505, 4.812)	1.980 (0.811, 4.836)

								LIT	1.647<br>(0.896,<br>3.028)	1.436<br>(0.576,<br>3.577)	**3.321<br>(1.438,<br>7.670)**	1.246<br>(0.586,<br>2.649)	1.765<br>(0.804,<br>3.875)	1.102<br>(0.191,<br>6.345)	1.468<br>(0.740,<br>2.911)	1.410<br>(0.606,<br>3.280)	**1.791<br>(1.093,<br>2.936)**
									OLA	0.872<br>(0.363,<br>2.094)	2.016<br>(0.905,<br>4.490)	0.756<br>(0.401,<br>1.427)	1.071<br>(0.451,<br>2.546)	0.669<br>(0.120,<br>3.742)	0.891<br>(0.526,<br>1.509)	0.856<br>(0.392,<br>1.867)	1.087<br>(0.714,<br>1.656)
										PAL	2.313<br>(0.914,<br>5.857)	0.867<br>(0.329,<br>2.284)	1.229<br>(0.413,<br>3.657)	0.768<br>(0.121,<br>4.882)	1.022<br>(0.395,<br>2.649)	0.982<br>(0.348,<br>2.768)	1.248<br>(0.576,<br>2.701)
											QUE	0.375<br>(0.152,<br>0.927)	0.531<br>(0.189,<br>1.495)	0.332<br>(0.054,<br>2.047)	0.442<br>(0.182,<br>1.072)	0.425<br>(0.161,<br>1.117)	0.539<br>(0.268,<br>1.086)
												RIS	1.417<br>(0.540,<br>3.718)	0.885<br>(0.160,<br>4.905)	1.179<br>(0.548,<br>2.535)	1.132<br>(0.464,<br>2.764)	1.438<br>(0.800,<br>2.585)
													TOP	0.624<br>(0.098,<br>3.968)	0.832<br>(0.327,<br>2.113)	0.799<br>(0.283,<br>2.252)	1.015<br>(0.469,<br>2.195)
														VALN	1.332<br>(0.228,<br>7.783)	1.279<br>(0.208,<br>7.873)	1.625<br>(0.303,<br>8.734)
															VALP	0.961<br>(0.400,<br>2.305)	1.220<br>(0.697,<br>2.137)

																ZIP	1.270 (0.632, 2.552)
																	PLA

Evaluation of heterogeneity and inconsistency

Between study variance (τ^2)	Heterogeneity assessment	Random-effects design-by-treatment interaction model		
		Q	df	p
0.106	Moderate to high	53.990	31	0.006

Incoherence

	NMA, RR (95% CI)	Direct, RR (95% CI)	I^2	Indirect, RR (95% CI)	Inconsistency measures	
					Difference of RR	P value
ARI vs HAL	0.649 (0.412, 1.023)	0.594 (0.312, 1.132)	91.3%	0.709 (0.374, 1.345)	0.838 (0.338, 2.079)	0.703
ARI vs LIT	0.677 (0.389, 1.177)	1.187 (0.508, 2.771)	na	0.446 (0.215, 0.926)	2.663 (0.869, 8.155)	0.086
ARI vs PLA	1.212 (0.850, 1.728)	1.223 (0.826, 1.812)	0.0%	1.164 (0.511, 2.655)	1.051 (0.422, 2.618)	0.915
ASE vs OLA	1.743 (0.984, 3.090)	2.583 (1.235, 5.404)	0.0%	0.963 (0.389, 2.384)	2.683 (0.834, 8.635)	0.098
ASE vs PLA	1.896 (1.117, 3.218)	1.550 (0.862, 2.785)	0.0%	4.605 (1.346, 15.760)	0.336 (0.086, 1.315)	0.117
BRE vs PLA		0.881 (0.334, 2.321)	35.9%			
CARB vs PLA		1.977 (0.882, 4.427)	0.0%			
CARI vs PLA		1.658 (0.949, 2.897)	0.0%			
END vs VALP		1.482 (0.134, 16.377)	0.0%			
HAL vs OLA	1.717 (1.058, 2.784)	1.811 (0.908, 3.613)	37.0%	1.631 (0.828, 3.209)	1.111 (0.422, 2.922)	0.832
HAL vs QUE	3.461 (1.622, 7.383)	2.061 (0.609, 6.969)	na	4.799 (1.824, 12.629)	0.429 (0.091, 2.035)	0.287
HAL vs RIS	1.298 (0.669, 2.519)	0.713 (0.176, 2.889)	na	1.544 (0.727, 3.278)	0.462 (0.094, 2.262)	0.341
HAL vs ZIP	1.469 (0.739, 2.919)	2.204 (0.956, 5.080)	na	0.629 (0.188, 2.104)	3.503 (0.807, 15.197)	0.094
HAL vs PLA	1.867 (1.255, 2.776)	1.437 (0.847, 2.438)	77.7%	2.618 (1.435, 4.776)	0.549 (0.246, 1.222)	0.142
LAM vs LIT	1.106 (0.456, 2.678)	1.381 (0.511, 3.732)	0.0%	0.473 (0.068, 3.299)	2.918 (0.329, 25.850)	0.336
LAM vs OLA	1.821 (0.696, 4.765)	1.000 (0.064, 15.690)	na	1.979 (0.709, 5.526)	0.505 (0.027, 9.539)	0.649
LAM vs PLA	1.980 (0.811, 4.836)	1.635 (0.546, 4.900)	na	2.880 (0.620, 13.370)	0.568 (0.086, 3.748)	0.557

LIT vs OLA	1.647 (0.896, 3.028)	1.565 (0.192, 12.775)	0.0%	1.655 (0.876, 3.127)	0.945 (0.105, 8.481)	0.960
LIT vs QUE	3.321 (1.438, 7.670)	7.000 (0.343, 142.722)	na	3.121 (1.306, 7.458)	2.243 (0.097, 51.733)	0.614
LIT vs TOP	1.765 (0.804, 3.875)	2.704 (0.987, 7.403)	11.8%	0.907 (0.258, 3.193)	2.981 (0.595, 14.939)	0.184
LIT vs VALP	1.468 (0.740, 2.911)	1.813 (0.494, 6.653)	0.0%	1.354 (0.605, 3.029)	1.340 (0.290, 6.182)	0.708
LIT vs PLA	1.791 (1.093, 2.936)	1.892 (1.043, 3.430)	10.9%	1.588 (0.655, 3.849)	1.191 (0.410, 3.463)	0.748
OLA vs RIS	0.756 (0.401, 1.427)	0.639 (0.228, 1.792)	na	0.838 (0.374, 1.877)	0.762 (0.206, 2.823)	0.685
OLA vs VALP	0.891 (0.526, 1.509)	1.193 (0.628, 2.264)	33.3%	0.486 (0.193, 1.225)	2.454 (0.797, 7.560)	0.118
OLA vs PLA	1.087 (0.714, 1.656)	1.047 (0.558, 1.966)	25.4%	1.121 (0.636, 1.974)	0.934 (0.401, 2.179)	0.875
PAL vs QUE	2.313 (0.914, 5.857)	2.227 (0.592, 8.380)	na	2.400 (0.652, 8.829)	0.928 (0.145, 5.951)	0.937
PAL vs PLA	1.248 (0.576, 2.701)	1.227 (0.544, 2.771)	0.0%	1.446 (0.125, 16.733)	0.849 (0.064, 11.200)	0.901
QUE vs PLA	0.539 (0.268, 1.086)	0.503 (0.232, 1.091)	0.0%	0.735 (0.145, 3.740)	0.684 (0.113, 4.145)	0.679
RIS vs VALN	0.885 (0.160, 4.905)	0.740 (0.073, 7.483)	na	1.099 (0.086, 14.031)	0.673 (0.022, 21.008)	0.821
RIS vs PLA	1.438 (0.800, 2.585)	1.153 (0.570, 2.335)	0.0%	2.358 (0.820, 6.779)	0.489 (0.137, 1.742)	0.270
TOP vs PLA	1.015 (0.469, 2.195)	1.495 (0.614, 3.642)	19.8%	0.316 (0.067, 1.482)	4.734 (0.795, 28.177)	0.088
VALN vs PLA	1.625 (0.303, 8.734)	1.479 (0.228, 9.605)	na	2.417 (0.052, 111.631)	0.612 (0.009, 43.550)	0.821
VALP vs PLA	1.220 (0.697, 2.137)	2.354 (1.024, 5.413)	0.0%	0.709 (0.332, 1.511)	3.322 (1.078, 10.242)	0.037
ZIP vs PLA	1.270 (0.632, 2.552)	2.082 (0.884, 4.902)	0.0%	0.481 (0.144, 1.598)	4.332 (0.990, 18.952)	0.051

For some outcomes of a network meta-analysis, the odds ratio may be considered a more appropriate effect size than the RR (Huhn 2020). For example, the odds ratio has mathematical properties that make it more appropriate for some outcomes of a network meta-analysis (e.g., the odds ratio is symmetrical). The odds ratio may also be a better effect size when the definition of an outcome differs between RCTs. In our study, there were differences in the definition of the outcome among RCTs. For example, recent studies have reported that the most common reason for discontinuation due to adverse events is the worsening of mania. Therefore, although we performed a network meta-analysis for this outcome using the odds ratio, the results also had global heterogeneity (τ^2 = 0.142) and global inconsistency (p = 0.003). These results are reported for presentation purposes only because these analyses were not included in a prespecified analysis plan; thus, further study will be needed to explore the reasons for this global heterogeneity and the global inconsistency.

Huhn M, et al. Lancet 2019; 394(10202): 939-951.

Forest plot (vs placebo, the numbers are risk ratio with 95% confidence interval)

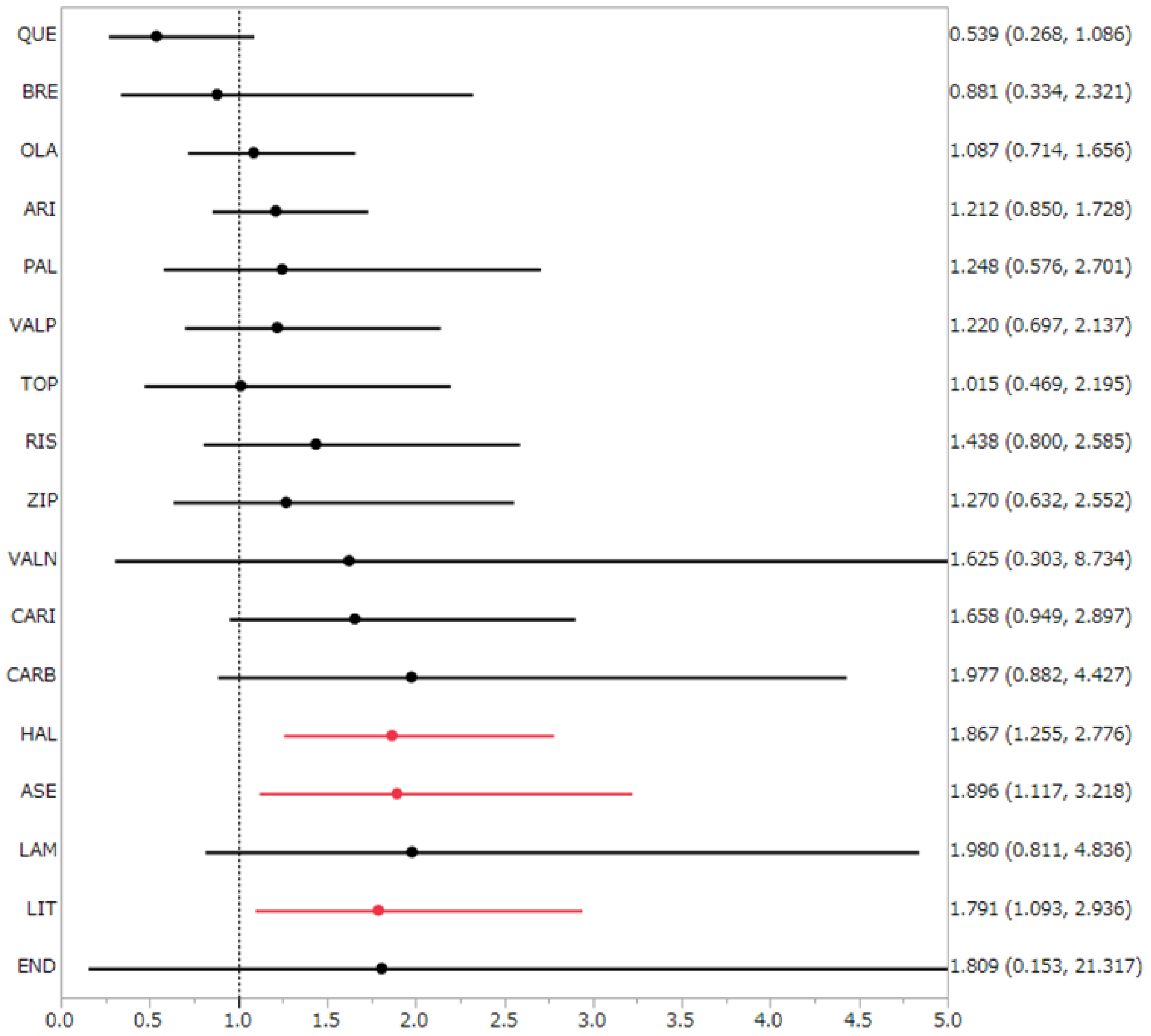

P-score

QUE	0.962
BRE	0.766
OLA	0.690
ARI	0.620
PAL	0.607
VALP	0.587
TOP	0.575
RIS	0.502
ZIP	0.500
VALN	0.464
CARI	0.417
CARB	0.340
HAL	0.334
ASE	0.315
LAM	0.290
LIT	0.232
END	0.009

Funnel plot (only double-blind, placebo-controlled trials)

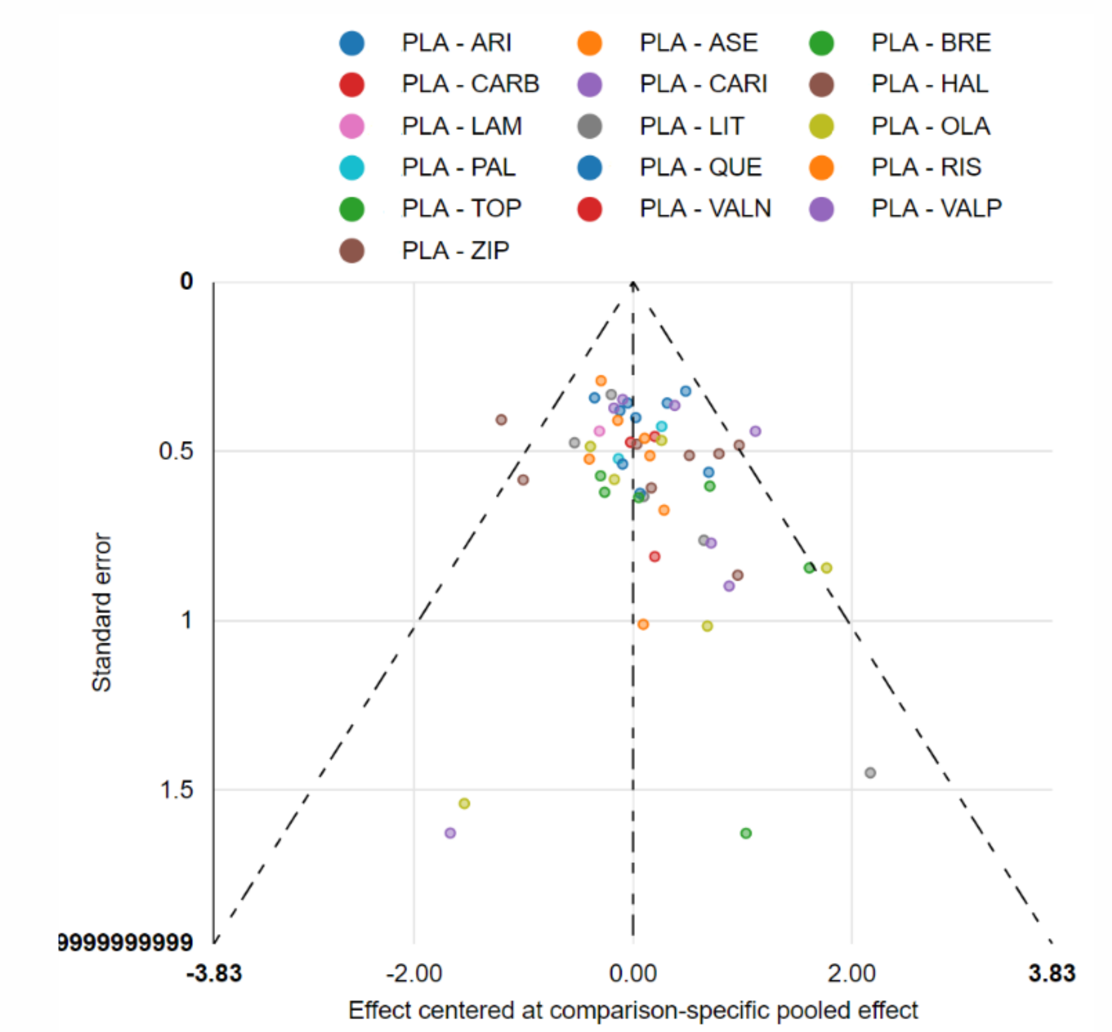

CINeMA confidence rating

Comparison	Number of studies	Within-study bias	Reporting bias	Indirectness	Imprecision	Heterogeneity	Incoherence	Confidence rating
ARI vs HAL	2	Some concerns	Suspected	No concerns	Major concerns	No concerns	No concerns	Very low
ARI vs LIT	1	Some concerns	Suspected	No concerns	Major concerns	No concerns	Major concerns	Very low
ARI vs PLA	6	Some concerns	Suspected	No concerns	Major concerns	No concerns	No concerns	Very low
ASE vs OLA	2	Some concerns	Suspected	No concerns	Major concerns	No concerns	Major concerns	Very low
ASE vs PLA	3	Some concerns	Suspected	No concerns	No concerns	Major concerns	No concerns	Very low
BRE vs PLA	2	Some concerns	Suspected	No concerns	Major concerns	No concerns	Major concerns	Very low
CARB vs PLA	2	Some concerns	Suspected	No concerns	Major concerns	No concerns	Major concerns	Very low
CARI vs PLA	3	Some concerns	Suspected	No concerns	Major concerns	No concerns	Major concerns	Very low
END vs VALP	2	No concerns	Suspected	No concerns	Major concerns	No concerns	Major concerns	Very low
HAL vs OLA	2	Some concerns	Suspected	No concerns	No concerns	Major concerns	No concerns	Very low
HAL vs QUE	1	Some concerns	Suspected	No concerns	No concerns	No concerns	No concerns	Low
HAL vs RIS	1	Some concerns	Suspected	No concerns	Major concerns	No concerns	No concerns	Very low
HAL vs ZIP	1	Some concerns	Suspected	No concerns	Major concerns	No concerns	No concerns	Very low
HAL vs PLA	5	Some concerns	Suspected	No concerns	No concerns	Major concerns	No concerns	Very low
LAM vs LIT	2	Some concerns	Suspected	No concerns	Major concerns	No concerns	No concerns	Very low
LAM vs OLA	1	Some concerns	Suspected	No concerns	Major concerns	No concerns	No concerns	Very low
LAM vs PLA	1	Some concerns	Suspected	No concerns	Major concerns	No concerns	No concerns	Very low
LIT vs OLA	2	Some concerns	Suspected	No concerns	Major concerns	No concerns	No concerns	Very low
LIT vs QUE	1	Some concerns	Suspected	No concerns	No concerns	No concerns	No concerns	Low
LIT vs TOP	2	No concerns	Suspected	No concerns	Major concerns	No concerns	No concerns	Low
LIT vs VALP	3	Some concerns	Suspected	No concerns	Major concerns	No concerns	No concerns	Very low
LIT vs PLA	5	Some concerns	Suspected	No concerns	No concerns	Major concerns	No concerns	Very low
OLA vs RIS	1	Some concerns	Suspected	No concerns	Major concerns	No concerns	No concerns	Very low

OLA vs VALP	4	No concerns	Suspected	No concerns	Major concerns	No concerns	No concerns	Low
OLA vs PLA	6	Some concerns	Suspected	No concerns	Major concerns	No concerns	No concerns	Very low
PAL vs QUE	1	No concerns	Suspected	No concerns	Major concerns	No concerns	No concerns	Low
PAL vs PLA	2	No concerns	Suspected	No concerns	Major concerns	No concerns	No concerns	Low
QUE vs PLA	3	No concerns	Suspected	No concerns	Major concerns	No concerns	No concerns	Low
RIS vs VALN	1	No concerns	Suspected	No concerns	Major concerns	No concerns	No concerns	Low
RIS vs PLA	4	Some concerns	Suspected	No concerns	Major concerns	No concerns	No concerns	Very low
TOP vs PLA	4	No concerns	Suspected	No concerns	Major concerns	No concerns	No concerns	Low
VALN vs PLA	1	No concerns	Suspected	No concerns	Major concerns	No concerns	No concerns	Low
VALP vs PLA	4	No concerns	Suspected	No concerns	Major concerns	No concerns	Major concerns	Very low
ZIP vs PLA	3	Some concerns	Suspected	No concerns	Major concerns	No concerns	No concerns	Very low
ARI vs ASE	0	Some concerns	Suspected	No concerns	Major concerns	No concerns	Major concerns	Very low
ARI vs BRE	0	Some concerns	Suspected	No concerns	Major concerns	No concerns	Major concerns	Very low
ARI vs CARB	0	Some concerns	Suspected	No concerns	Major concerns	No concerns	Major concerns	Very low
ARI vs CARI	0	Some concerns	Suspected	No concerns	Major concerns	No concerns	Major concerns	Very low
ARI vs END	0	No concerns	Suspected	No concerns	Major concerns	No concerns	Major concerns	Very low
ARI vs LAM	0	Some concerns	Suspected	No concerns	Major concerns	No concerns	Major concerns	Very low
ARI vs OLA	0	Some concerns	Suspected	No concerns	Major concerns	No concerns	Major concerns	Very low
ARI vs PAL	0	Some concerns	Suspected	No concerns	Major concerns	No concerns	Major concerns	Very low
ARI vs QUE	0	Some concerns	Suspected	No concerns	No concerns	Major concerns	Major concerns	Very low
ARI vs RIS	0	Some concerns	Suspected	No concerns	Major concerns	No concerns	Major concerns	Very low
ARI vs TOP	0	Some concerns	Suspected	No concerns	Major concerns	No concerns	Major concerns	Very low
ARI vs VALN	0	Some concerns	Suspected	No concerns	Major concerns	No concerns	Major concerns	Very low
ARI vs VALP	0	Some concerns	Suspected	No concerns	Major concerns	No concerns	Major concerns	Very low
ARI vs ZIP	0	Some concerns	Suspected	No concerns	Major concerns	No concerns	Major concerns	Very low

ASE vs BRE	0	Some concerns	Suspected	No concerns	Major concerns	No concerns	Major concerns	Very low
ASE vs CARB	0	Some concerns	Suspected	No concerns	Major concerns	No concerns	Major concerns	Very low
ASE vs CARI	0	Some concerns	Suspected	No concerns	Major concerns	No concerns	Major concerns	Very low
ASE vs END	0	No concerns	Suspected	No concerns	Major concerns	No concerns	Major concerns	Very low
ASE vs HAL	0	Some concerns	Suspected	No concerns	Major concerns	No concerns	Major concerns	Very low
ASE vs LAM	0	Some concerns	Suspected	No concerns	Major concerns	No concerns	Major concerns	Very low
ASE vs LIT	0	Some concerns	Suspected	No concerns	Major concerns	No concerns	Major concerns	Very low
ASE vs PAL	0	No concerns	Suspected	No concerns	Major concerns	No concerns	Major concerns	Very low
ASE vs QUE	0	Some concerns	Suspected	No concerns	No concerns	No concerns	Major concerns	Very low
ASE vs RIS	0	Some concerns	Suspected	No concerns	Major concerns	No concerns	Major concerns	Very low
ASE vs TOP	0	No concerns	Suspected	No concerns	Major concerns	No concerns	Major concerns	Very low
ASE vs VALN	0	No concerns	Suspected	No concerns	Major concerns	No concerns	Major concerns	Very low
ASE vs VALP	0	Some concerns	Suspected	No concerns	Major concerns	No concerns	Major concerns	Very low
ASE vs ZIP	0	Some concerns	Suspected	No concerns	Major concerns	No concerns	Major concerns	Very low
BRE vs CARB	0	Some concerns	Suspected	No concerns	Major concerns	No concerns	Major concerns	Very low
BRE vs CARI	0	Some concerns	Suspected	No concerns	Major concerns	No concerns	Major concerns	Very low
BRE vs END	0	Some concerns	Suspected	No concerns	Major concerns	No concerns	Major concerns	Very low
BRE vs HAL	0	Some concerns	Suspected	No concerns	Major concerns	No concerns	Major concerns	Very low
BRE vs LAM	0	Some concerns	Suspected	No concerns	Major concerns	No concerns	Major concerns	Very low
BRE vs LIT	0	Some concerns	Suspected	No concerns	Major concerns	No concerns	Major concerns	Very low
BRE vs OLA	0	Some concerns	Suspected	No concerns	Major concerns	No concerns	Major concerns	Very low
BRE vs PAL	0	Some concerns	Suspected	No concerns	Major concerns	No concerns	Major concerns	Very low
BRE vs QUE	0	Some concerns	Suspected	No concerns	Major concerns	No concerns	Major concerns	Very low
BRE vs RIS	0	Some concerns	Suspected	No concerns	Major concerns	No concerns	Major concerns	Very low
BRE vs TOP	0	Some concerns	Suspected	No concerns	Major concerns	No concerns	Major concerns	Very low

BRE vs VALN	0	Some concerns	Suspected	No concerns	Major concerns	No concerns	Major concerns	Very low
BRE vs VALP	0	Some concerns	Suspected	No concerns	Major concerns	No concerns	Major concerns	Very low
BRE vs ZIP	0	Some concerns	Suspected	No concerns	Major concerns	No concerns	Major concerns	Very low
CARB vs CARI	0	Some concerns	Suspected	No concerns	Major concerns	No concerns	Major concerns	Very low
CARB vs END	0	Some concerns	Suspected	No concerns	Major concerns	No concerns	Major concerns	Very low
CARB vs HAL	0	Some concerns	Suspected	No concerns	Major concerns	No concerns	Major concerns	Very low
CARB vs LAM	0	Some concerns	Suspected	No concerns	Major concerns	No concerns	Major concerns	Very low
CARB vs LIT	0	Some concerns	Suspected	No concerns	Major concerns	No concerns	Major concerns	Very low
CARB vs OLA	0	Some concerns	Suspected	No concerns	Major concerns	No concerns	Major concerns	Very low
CARB vs PAL	0	Some concerns	Suspected	No concerns	Major concerns	No concerns	Major concerns	Very low
CARB vs QUE	0	Some concerns	Suspected	No concerns	No concerns	No concerns	Major concerns	Very low
CARB vs RIS	0	Some concerns	Suspected	No concerns	Major concerns	No concerns	Major concerns	Very low
CARB vs TOP	0	Some concerns	Suspected	No concerns	Major concerns	No concerns	Major concerns	Very low
CARB vs VALN	0	Some concerns	Suspected	No concerns	Major concerns	No concerns	Major concerns	Very low
CARB vs VALP	0	Some concerns	Suspected	No concerns	Major concerns	No concerns	Major concerns	Very low
CARB vs ZIP	0	Some concerns	Suspected	No concerns	Major concerns	No concerns	Major concerns	Very low
CARI vs END	0	Some concerns	Suspected	No concerns	Major concerns	No concerns	Major concerns	Very low
CARI vs HAL	0	Some concerns	Suspected	No concerns	Major concerns	No concerns	Major concerns	Very low
CARI vs LAM	0	Some concerns	Suspected	No concerns	Major concerns	No concerns	Major concerns	Very low
CARI vs LIT	0	Some concerns	Suspected	No concerns	Major concerns	No concerns	Major concerns	Very low
CARI vs OLA	0	Some concerns	Suspected	No concerns	Major concerns	No concerns	Major concerns	Very low
CARI vs PAL	0	Some concerns	Suspected	No concerns	Major concerns	No concerns	Major concerns	Very low
CARI vs QUE	0	Some concerns	Suspected	No concerns	No concerns	Major concerns	Major concerns	Very low
CARI vs RIS	0	Some concerns	Suspected	No concerns	Major concerns	No concerns	Major concerns	Very low
CARI vs TOP	0	Some concerns	Suspected	No concerns	Major concerns	No concerns	Major concerns	Very low

CARI vs VALN	0	Some concerns	Suspected	No concerns	Major concerns	No concerns	Major concerns	Very low
CARI vs VALP	0	Some concerns	Suspected	No concerns	Major concerns	No concerns	Major concerns	Very low
CARI vs ZIP	0	Some concerns	Suspected	No concerns	Major concerns	No concerns	Major concerns	Very low
END vs HAL	0	No concerns	Suspected	No concerns	Major concerns	No concerns	Major concerns	Very low
END vs LAM	0	Some concerns	Suspected	No concerns	Major concerns	No concerns	Major concerns	Very low
END vs LIT	0	No concerns	Suspected	No concerns	Major concerns	No concerns	Major concerns	Very low
END vs OLA	0	No concerns	Suspected	No concerns	Major concerns	No concerns	Major concerns	Very low
END vs PAL	0	No concerns	Suspected	No concerns	Major concerns	No concerns	Major concerns	Very low
END vs QUE	0	No concerns	Suspected	No concerns	Major concerns	No concerns	Major concerns	Very low
END vs RIS	0	No concerns	Suspected	No concerns	Major concerns	No concerns	Major concerns	Very low
END vs TOP	0	No concerns	Suspected	No concerns	Major concerns	No concerns	Major concerns	Very low
END vs VALN	0	No concerns	Suspected	No concerns	Major concerns	No concerns	Major concerns	Very low
END vs ZIP	0	No concerns	Suspected	No concerns	Major concerns	No concerns	Major concerns	Very low
END vs PLA	0	No concerns	Suspected	No concerns	Major concerns	No concerns	Major concerns	Very low
HAL vs LAM	0	Some concerns	Suspected	No concerns	Major concerns	No concerns	Major concerns	Very low
HAL vs LIT	0	Some concerns	Suspected	No concerns	Major concerns	No concerns	Major concerns	Very low
HAL vs PAL	0	Some concerns	Suspected	No concerns	Major concerns	No concerns	Major concerns	Very low
HAL vs TOP	0	Some concerns	Suspected	No concerns	Major concerns	No concerns	Major concerns	Very low
HAL vs VALN	0	Some concerns	Suspected	No concerns	Major concerns	No concerns	Major concerns	Very low
HAL vs VALP	0	Some concerns	Suspected	No concerns	Major concerns	No concerns	Major concerns	Very low
LAM vs PAL	0	Some concerns	Suspected	No concerns	Major concerns	No concerns	Major concerns	Very low
LAM vs QUE	0	Some concerns	Suspected	No concerns	No concerns	Major concerns	Major concerns	Very low
LAM vs RIS	0	Some concerns	Suspected	No concerns	Major concerns	No concerns	Major concerns	Very low
LAM vs TOP	0	Some concerns	Suspected	No concerns	Major concerns	No concerns	Major concerns	Very low
LAM vs VALN	0	Some concerns	Suspected	No concerns	Major concerns	No concerns	Major concerns	Very low

LAM vs VALP	0	Some concerns	Suspected	No concerns	Major concerns	No concerns	Major concerns	Very low
LAM vs ZIP	0	Some concerns	Suspected	No concerns	Major concerns	No concerns	Major concerns	Very low
LIT vs PAL	0	No concerns	Suspected	No concerns	Major concerns	No concerns	Major concerns	Very low
LIT vs RIS	0	Some concerns	Suspected	No concerns	Major concerns	No concerns	Major concerns	Very low
LIT vs VALN	0	No concerns	Suspected	No concerns	Major concerns	No concerns	Major concerns	Very low
LIT vs ZIP	0	Some concerns	Suspected	No concerns	Major concerns	No concerns	Major concerns	Very low
OLA vs PAL	0	No concerns	Suspected	No concerns	Major concerns	No concerns	Major concerns	Very low
OLA vs QUE	0	Some concerns	Suspected	No concerns	Major concerns	No concerns	Major concerns	Very low
OLA vs TOP	0	No concerns	Suspected	No concerns	Major concerns	No concerns	Major concerns	Very low
OLA vs VALN	0	No concerns	Suspected	No concerns	Major concerns	No concerns	Major concerns	Very low
OLA vs ZIP	0	Some concerns	Suspected	No concerns	Major concerns	No concerns	Major concerns	Very low
PAL vs RIS	0	Some concerns	Suspected	No concerns	Major concerns	No concerns	Major concerns	Very low
PAL vs TOP	0	No concerns	Suspected	No concerns	Major concerns	No concerns	Major concerns	Very low
PAL vs VALN	0	No concerns	Suspected	No concerns	Major concerns	No concerns	Major concerns	Very low
PAL vs VALP	0	No concerns	Suspected	No concerns	Major concerns	No concerns	Major concerns	Very low
PAL vs ZIP	0	Some concerns	Suspected	No concerns	Major concerns	No concerns	Major concerns	Very low
QUE vs RIS	0	Some concerns	Suspected	No concerns	No concerns	Major concerns	Major concerns	Very low
QUE vs TOP	0	No concerns	Suspected	No concerns	Major concerns	No concerns	Major concerns	Very low
QUE vs VALN	0	No concerns	Suspected	No concerns	Major concerns	No concerns	Major concerns	Very low
QUE vs VALP	0	No concerns	Suspected	No concerns	Major concerns	No concerns	Major concerns	Very low
QUE vs ZIP	0	Some concerns	Suspected	No concerns	Major concerns	No concerns	Major concerns	Very low
RIS vs TOP	0	Some concerns	Suspected	No concerns	Major concerns	No concerns	Major concerns	Very low
RIS vs VALP	0	Some concerns	Suspected	No concerns	Major concerns	No concerns	Major concerns	Very low
RIS vs ZIP	0	Some concerns	Suspected	No concerns	Major concerns	No concerns	Major concerns	Very low
TOP vs VALN	0	No concerns	Suspected	No concerns	Major concerns	No concerns	Major concerns	Very low

TOP vs VALP	0	No concerns	Suspected	No concerns	Major concerns	No concerns	Major concerns	Very low
TOP vs ZIP	0	Some concerns	Suspected	No concerns	Major concerns	No concerns	Major concerns	Very low
VALN vs VALP	0	No concerns	Suspected	No concerns	Major concerns	No concerns	Major concerns	Very low
VALN vs ZIP	0	Some concerns	Suspected	No concerns	Major concerns	No concerns	Major concerns	Very low
VALP vs ZIP	0	Some concerns	Suspected	No concerns	Major concerns	No concerns	Major concerns	Very low

Supplementary Appendix 8. Discontinuation due to withdrawal consent (N = 42, n = 11968)

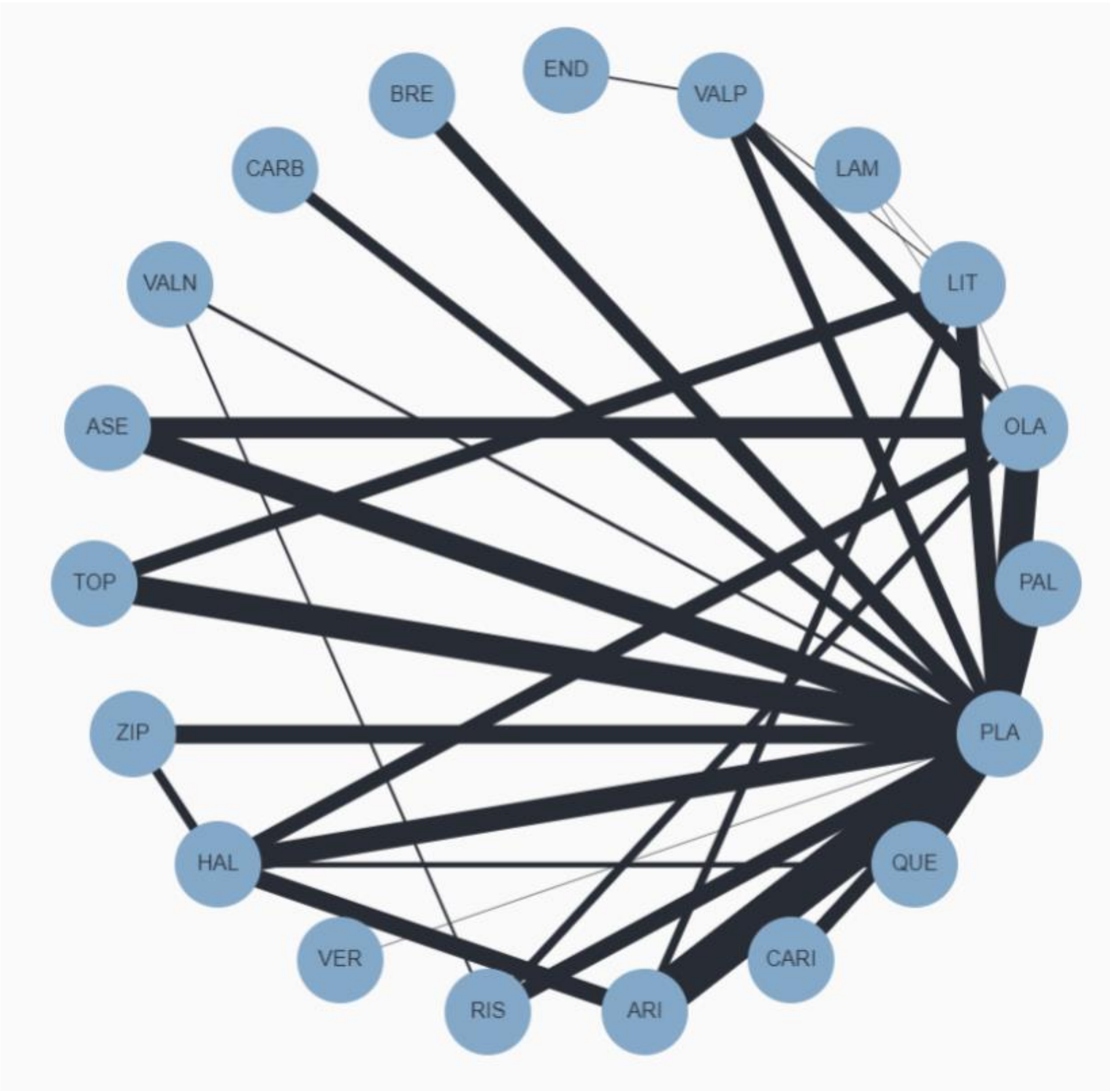

League table (risk ratio with 95% confidence interval)

ARI	1.002 (0.637, 1.578)	0.906 (0.548, 1.499)	1.292 (0.750, 2.226)	0.966 (0.576, 1.618)	0.687 (0.064, 7.417)	1.006 (0.730, 1.388)	1.530 (0.232, 10.108)	1.075 (0.719, 1.606)	**1.866 (1.266, 2.750)**	0.913 (0.548, 1.522)	1.202 (0.738, 1.958)	0.969 (0.573, 1.638)	0.961 (0.587, 1.574)	0.725 (0.198, 2.651)	1.298 (0.773, 2.179)	0.151 (0.009, 2.616)	1.069 (0.714, 1.601)	1.200 (0.950, 1.516)
	ASE	0.904 (0.499, 1.640)	1.289 (0.686, 2.421)	0.963 (0.525, 1.766)	0.685 (0.063, 7.477)	1.004 (0.628, 1.605)	1.527 (0.224, 10.413)	1.072 (0.617, 1.863)	**1.861 (1.258, 2.754)**	0.911 (0.498, 1.664)	1.199 (0.669, 2.152)	0.967 (0.536, 1.745)	0.959 (0.529, 1.737)	0.724 (0.192, 2.726)	1.294 (0.736, 2.276)	0.150 (0.008, 2.656)	1.067 (0.635, 1.792)	1.197 (0.807, 1.777)
		BRE	1.425 (0.734, 2.766)	1.065 (0.561, 2.021)	0.758 (0.068, 8.443)	1.110 (0.653, 1.887)	1.688 (0.244, 11.705)	1.186 (0.655, 2.147)	**2.058 (1.187, 3.569)**	1.007 (0.532, 1.906)	1.326 (0.712, 2.471)	1.069 (0.558, 2.047)	1.061 (0.565, 1.991)	0.800 (0.207, 3.090)	1.431 (0.750, 2.734)	0.166 (0.009, 2.959)	1.180 (0.671, 2.073)	1.324 (0.848, 2.067)
			CARB	0.747 (0.381, 1.466)	0.532 (0.047, 5.977)	0.779 (0.441, 1.377)	1.185 (0.169, 8.305)	0.832 (0.444, 1.561)	1.444 (0.802, 2.601)	0.707 (0.361, 1.382)	0.931 (0.483, 1.794)	0.750 (0.379, 1.484)	0.744 (0.384, 1.444)	0.562 (0.143, 2.203)	1.005 (0.509, 1.982)	0.117 (0.006, 2.092)	0.828 (0.454, 1.509)	0.929 (0.568, 1.519)
				CARI	0.711 (0.064, 7.949)	1.042 (0.606, 1.794)	1.585 (0.228, 11.026)	1.113 (0.608, 2.039)	**1.932 (1.101, 3.391)**	0.946 (0.494, 1.808)	1.245 (0.661, 2.346)	1.004 (0.519, 1.942)	0.996 (0.525, 1.889)	0.751 (0.194, 2.916)	1.344 (0.696, 2.593)	0.156 (0.009, 2.785)	1.107 (0.623, 1.969)	1.243 (0.785, 1.970)
					END	1.465 (0.135, 15.863)	2.228 (0.108, 45.741)	1.565 (0.142, 17.212)	2.716 (0.255, 28.968)	1.329 (0.119, 14.834)	1.750 (0.158, 19.445)	1.411 (0.127, 15.691)	1.399 (0.126, 15.579)	1.056 (0.072, 15.493)	1.889 (0.185, 19.259)	0.219 (0.005, 8.888)	1.556 (0.142, 17.032)	1.747 (0.163, 18.673)
						HAL	1.521 (0.227, 10.179)	1.068 (0.667, 1.709)	**1.854 (1.258, 2.732)**	0.907 (0.533, 1.544)	1.195 (0.729, 1.958)	0.963 (0.559, 1.659)	0.955 (0.564, 1.618)	0.721 (0.196, 2.657)	1.289 (0.758, 2.194)	0.150 (0.009, 2.612)	1.062 (0.722, 1.563)	1.193 (0.894, 1.591)
							LAM	0.702 (0.110, 4.489)	1.219 (0.182, 8.163)	0.597 (0.086, 4.146)	0.786 (0.114, 5.429)	0.633 (0.091, 4.406)	0.628 (0.092, 4.295)	0.474 (0.049, 4.604)	0.848 (0.123, 5.865)	0.098 (0.003, 2.986)	0.699 (0.103, 4.738)	0.784 (0.119, 5.162)

								LIT	**1.736 (1.054, 2.858)**	0.850 (0.465, 1.550)	1.119 (0.624, 2.005)	0.902 (0.489, 1.663)	0.894 (0.522, 1.532)	0.675 (0.178, 2.562)	1.207 (0.663, 2.197)	0.140 (0.008, 2.477)	0.995 (0.592, 1.670)	1.117 (0.754, 1.654)
									OLA	**0.489 (0.280, 0.855)**	0.644 (0.377, 1.101)	**0.519 (0.310, 0.871)**	**0.515 (0.298, 0.892)**	0.389 (0.106, 1.424)	0.696 (0.439, 1.102)	0.081 (0.005, 1.415)	**0.573 (0.362, 0.908)**	**0.643 (0.466, 0.889)**
										PAL	1.317 (0.771, 2.249)	1.062 (0.550, 2.047)	1.053 (0.557, 1.992)	0.794 (0.205, 3.079)	1.421 (0.739, 2.733)	0.165 (0.009, 2.944)	1.171 (0.662, 2.072)	1.315 (0.833, 2.075)
											QUE	0.806 (0.425, 1.530)	0.800 (0.429, 1.489)	0.603 (0.157, 2.321)	1.079 (0.571, 2.041)	0.125 (0.007, 2.228)	0.889 (0.514, 1.537)	0.998 (0.646, 1.542)
												RIS	0.992 (0.518, 1.898)	0.748 (0.219, 2.557)	1.339 (0.705, 2.542)	0.155 (0.009, 2.780)	1.103 (0.616, 1.976)	1.238 (0.772, 1.987)
													TOP	0.755 (0.195, 2.913)	1.350 (0.709, 2.570)	0.157 (0.009, 2.790)	1.112 (0.634, 1.952)	1.248 (0.800, 1.948)
														VALN	1.789 (0.464, 6.901)	0.208 (0.009, 4.693)	1.474 (0.393, 5.522)	1.655 (0.462, 5.926)
															VALP	0.116 (0.006, 2.075)	0.824 (0.463, 1.468)	0.925 (0.579, 1.479)

																VER	7.100 (0.404, 124.773)	7.972 (0.463, 137.181)
																	ZIP	1.123 (0.794, 1.586)
																		PLA

Evaluation of heterogeneity and inconsistency

Between study variance (τ^2)	Heterogeneity assessment	Random-effects design-by-treatment interaction model		
		Q	df	p
0.007	Low	31.822	23	0.104

Incoherence

	NMA, RR (95% CI)	Direct, RR (95% CI)	I^2	Indirect, RR (95% CI)	Inconsistency measures	
					Difference of RR	P value
ARI vs HAL	1.006 (0.730, 1.388)	0.778 (0.487, 1.244)	0.0%	1.263 (0.813, 1.964)	0.616 (0.323, 1.173)	0.140
ARI vs LIT	1.075 (0.719, 1.606)	1.180 (0.726, 1.917)	na	0.879 (0.431, 1.795)	1.342 (0.566, 3.182)	0.504
ARI vs PLA	1.200 (0.950, 1.516)	1.267 (0.986, 1.627)	0.0%	0.845 (0.445, 1.604)	1.500 (0.753, 2.986)	0.249
ASE vs OLA	1.861 (1.258, 2.754)	1.792 (1.159, 2.770)	0.0%	2.186 (0.892, 5.358)	0.819 (0.302, 2.220)	0.695
ASE vs PLA	1.197 (0.807, 1.777)	1.200 (0.772, 1.865)	57.2%	1.186 (0.488, 2.881)	1.012 (0.376, 2.726)	0.981
BRE vs PLA		1.324 (0.848, 2.067)	0.0%			
CARB vs PLA		0.929 (0.568, 1.519)	0.0%			
CARI vs PLA		1.243 (0.785, 1.970)	0.0%			
END vs VALP		1.889 (0.185, 19.259)	0.0%			
HAL vs OLA	1.854 (1.258, 2.732)	1.518 (0.786, 2.933)	78.9%	2.061 (1.276, 3.330)	0.737 (0.326, 1.664)	0.462
HAL vs QUE	1.195 (0.729, 1.958)	0.916 (0.363, 2.312)	na	1.328 (0.741, 2.380)	0.690 (0.231, 2.062)	0.506
HAL vs ZIP	1.062 (0.722, 1.563)	1.041 (0.632, 1.713)	na	1.095 (0.595, 2.015)	0.950 (0.432, 2.089)	0.899
HAL vs PLA	1.193 (0.894, 1.591)	1.146 (0.779, 1.687)	54.8%	1.253 (0.814, 1.931)	0.915 (0.512, 1.633)	0.763
LAM vs LIT	0.702 (0.110, 4.489)	0.600 (0.089, 4.032)	na	12.472 (0.004, 42890.258)	0.048 (0.000, 206.149)	0.477
LAM vs OLA	1.219 (0.182, 8.163)	3.000 (0.132, 68.388)	na	0.719 (0.065, 7.885)	4.175 (0.081, 214.391)	0.477
LIT vs OLA	1.736 (1.054, 2.858)	5.000 (0.260, 96.322)	na	1.683 (1.015, 2.791)	2.971 (0.148, 59.746)	0.477
LIT vs TOP	0.894 (0.522, 1.532)	0.844 (0.364, 1.957)	0.0%	0.931 (0.463, 1.876)	0.906 (0.303, 2.706)	0.860

LIT vs VALP	1.207 (0.663, 2.197)	4.211 (0.404, 43.860)	na	1.106 (0.596, 2.055)	3.806 (0.337, 42.961)	0.280
LIT vs PLA	1.117 (0.754, 1.654)	1.111 (0.702, 1.760)	0.0%	1.132 (0.531, 2.410)	0.982 (0.405, 2.379)	0.968
OLA vs RIS	0.519 (0.310, 0.871)	0.387 (0.163, 0.915)	na	0.614 (0.321, 1.171)	0.630 (0.214, 1.851)	0.401
OLA vs VALP	0.696 (0.439, 1.102)	0.675 (0.391, 1.163)	48.5%	0.750 (0.318, 1.767)	0.899 (0.326, 2.483)	0.838
OLA vs PLA	0.643 (0.466, 0.889)	0.569 (0.381, 0.850)	0.0%	0.806 (0.468, 1.388)	0.706 (0.359, 1.389)	0.313
PAL vs QUE	1.317 (0.771, 2.249)	1.414 (0.721, 2.774)	na	1.167 (0.484, 2.811)	1.212 (0.400, 3.670)	0.734
PAL vs PLA	1.315 (0.833, 2.075)	1.183 (0.731, 1.913)	72.7%	3.471 (0.809, 14.884)	0.341 (0.074, 1.578)	0.169
QUE vs PLA	0.998 (0.646, 1.542)	0.907 (0.566, 1.456)	49.8%	1.691 (0.557, 5.134)	0.537 (0.161, 1.794)	0.312
RIS vs VALN	0.748 (0.219, 2.557)	1.109 (0.293, 4.203)	0.0%	0.079 (0.003, 1.909)	13.984 (0.445, 439.703)	0.134
RIS vs PLA	1.238 (0.772, 1.987)	1.098 (0.634, 1.903)	53.2%	1.743 (0.690, 4.404)	0.630 (0.214, 1.851)	0.401
TOP vs PLA	1.248 (0.800, 1.948)	1.222 (0.763, 1.958)	0.0%	1.489 (0.384, 5.777)	0.821 (0.195, 3.450)	0.788
VALN vs PLA	1.655 (0.462, 5.926)	5.916 (0.726, 48.192)	na	0.783 (0.157, 3.908)	7.550 (0.537, 106.059)	0.134
VALP vs PLA	0.925 (0.579, 1.479)	1.083 (0.594, 1.977)	0.0%	0.724 (0.342, 1.532)	1.497 (0.572, 3.917)	0.411
VER vs PLA		7.972 (0.463, 137.181)	na			
ZIP vs PLA	1.123 (0.794, 1.586)	1.031 (0.713, 1.490)	45.6%	2.109 (0.774, 5.747)	0.489 (0.168, 1.423)	0.189

Forest plot (vs placebo, the numbers are risk ratio with 95% confidence interval)

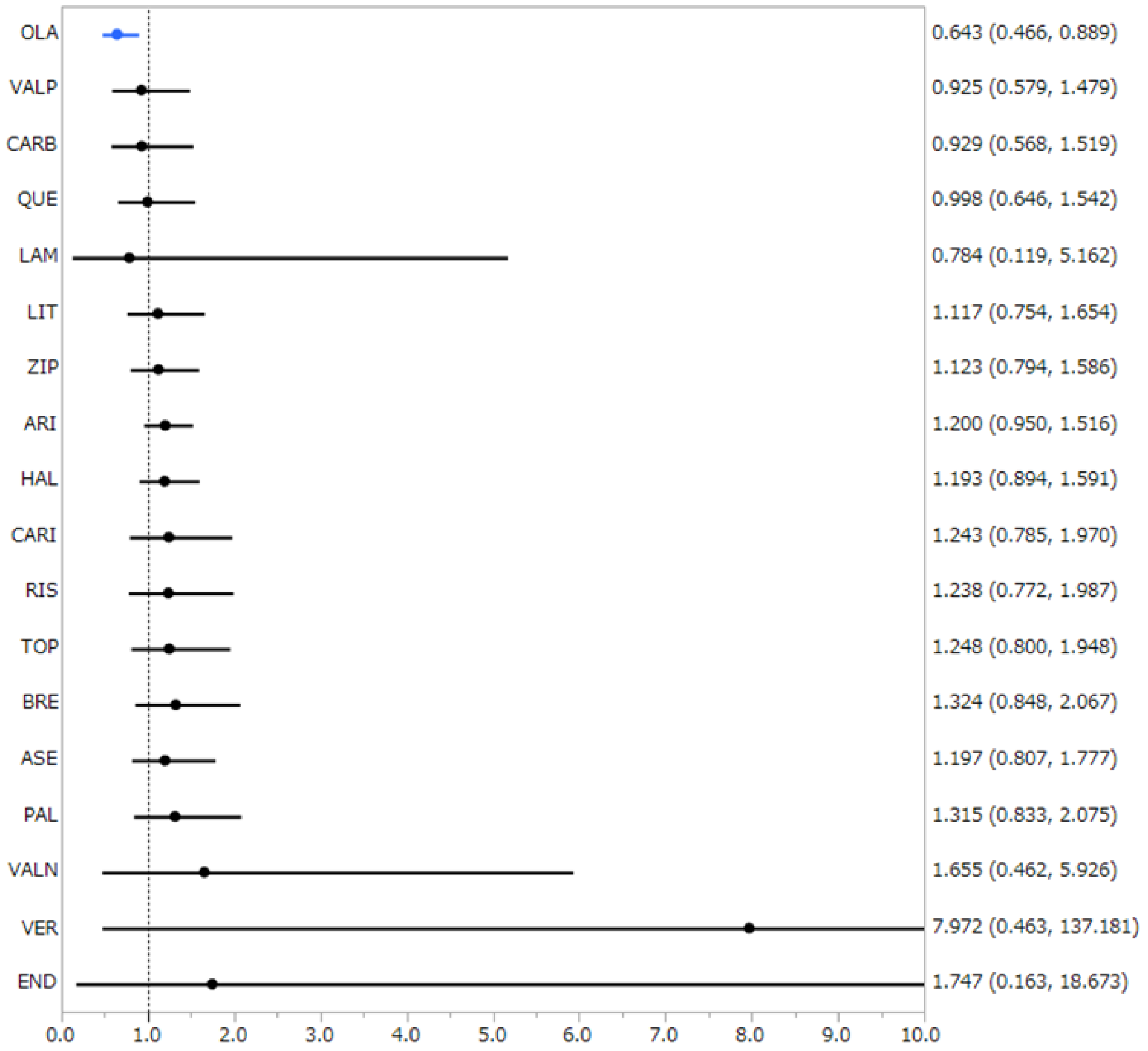

P-score

OLA	0.956
VALP	0.744
CARB	0.742
QUE	0.663
LAM	0.643
LIT	0.544
ZIP	0.541
ARI	0.505
HAL	0.494
CARI	0.489
RIS	0.479
TOP	0.462
BRE	0.430
ASE	0.384
PAL	0.381
VALN	0.247
VER	0.030
END	0.029

Funnel plot (only double-blind, placebo-controlled trials)

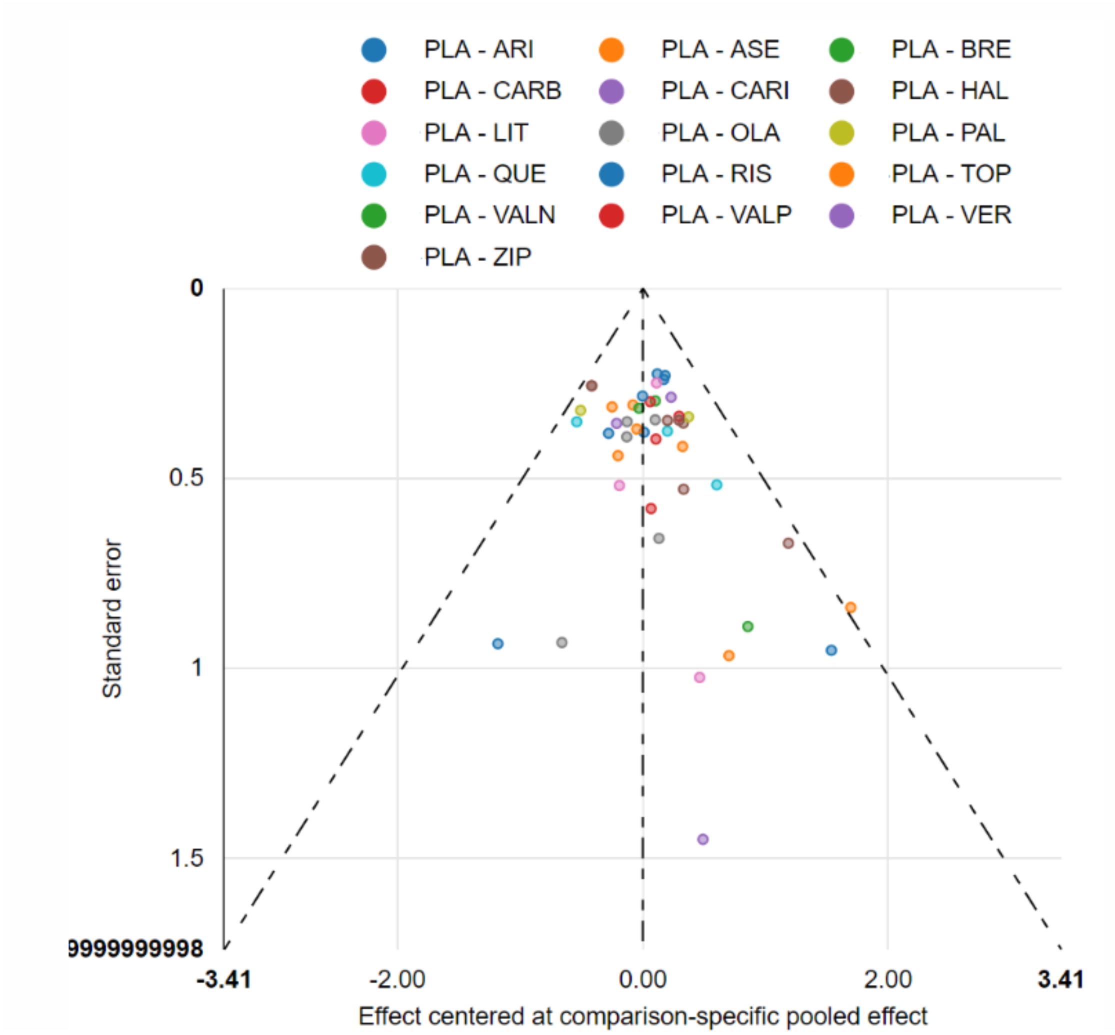

CINeMA confidence rating

Comparison	Number of studies	Within-study bias	Reporting bias	Indirectness	Imprecision	Heterogeneity	Incoherence	Confidence rating
ARI vs HAL	2	Some concerns	Suspected	No concerns	Major concerns	No concerns	No concerns	Very low
ARI vs LIT	1	Some concerns	Suspected	No concerns	Major concerns	No concerns	No concerns	Very low
ARI vs PLA	5	Some concerns	Suspected	No concerns	Major concerns	No concerns	No concerns	Very low
ASE vs OLA	2	Some concerns	Suspected	No concerns	No concerns	No concerns	No concerns	Low
ASE vs PLA	3	Some concerns	Suspected	No concerns	Major concerns	No concerns	No concerns	Very low
BRE vs PLA	2	Some concerns	Suspected	No concerns	Major concerns	No concerns	No concerns	Very low
CARB vs PLA	2	Some concerns	Suspected	No concerns	Major concerns	No concerns	No concerns	Very low
CARI vs PLA	2	Some concerns	Suspected	No concerns	Major concerns	No concerns	No concerns	Very low
END vs VALP	2	No concerns	Suspected	No concerns	Major concerns	No concerns	No concerns	Low
HAL vs OLA	2	Some concerns	Suspected	No concerns	No concerns	No concerns	No concerns	Low
HAL vs QUE	1	Some concerns	Suspected	No concerns	Major concerns	No concerns	No concerns	Very low
HAL vs ZIP	1	Some concerns	Suspected	No concerns	Major concerns	No concerns	No concerns	Very low
HAL vs PLA	4	Some concerns	Suspected	No concerns	Major concerns	No concerns	No concerns	Very low
LAM vs LIT	1	Some concerns	Suspected	No concerns	Major concerns	No concerns	No concerns	Very low
LAM vs OLA	1	Some concerns	Suspected	No concerns	Major concerns	No concerns	No concerns	Very low
LIT vs OLA	1	Some concerns	Suspected	No concerns	No concerns	No concerns	No concerns	Low
LIT vs TOP	2	No concerns	Suspected	No concerns	Major concerns	No concerns	No concerns	Low
LIT vs VALP	1	Some concerns	Suspected	No concerns	Major concerns	No concerns	No concerns	Very low
LIT vs PLA	3	Some concerns	Suspected	No concerns	Major concerns	No concerns	No concerns	Very low
OLA vs RIS	1	Some concerns	Suspected	No concerns	No concerns	No concerns	No concerns	Low
OLA vs VALP	3	No concerns	Suspected	No concerns	Major concerns	No concerns	No concerns	Low
OLA vs PLA	5	Some concerns	Suspected	No concerns	No concerns	No concerns	No concerns	Low
PAL vs QUE	1	No concerns	Suspected	No concerns	Major concerns	No concerns	No concerns	Low

PAL vs PLA	2	No concerns	Suspected	No concerns	Major concerns	No concerns	No concerns	Low
QUE vs PLA	3	No concerns	Suspected	No concerns	Major concerns	No concerns	No concerns	Low
RIS vs VALN	1	No concerns	Suspected	No concerns	Major concerns	No concerns	No concerns	Low
RIS vs PLA	3	Some concerns	Suspected	No concerns	Major concerns	No concerns	No concerns	Very low
TOP vs PLA	4	No concerns	Suspected	No concerns	Major concerns	No concerns	No concerns	Low
VALN vs PLA	1	No concerns	Suspected	No concerns	Major concerns	No concerns	No concerns	Low
VALP vs PLA	2	No concerns	Suspected	No concerns	Major concerns	No concerns	No concerns	Low
VER vs PLA	1	No concerns	Suspected	No concerns	Major concerns	No concerns	No concerns	Low
ZIP vs PLA	3	Some concerns	Suspected	No concerns	Major concerns	No concerns	No concerns	Very low
ARI vs ASE	0	Some concerns	Suspected	No concerns	Major concerns	No concerns	No concerns	Very low
ARI vs BRE	0	Some concerns	Suspected	No concerns	Major concerns	No concerns	No concerns	Very low
ARI vs CARB	0	Some concerns	Suspected	No concerns	Major concerns	No concerns	No concerns	Very low
ARI vs CARI	0	Some concerns	Suspected	No concerns	Major concerns	No concerns	No concerns	Very low
ARI vs END	0	No concerns	Suspected	No concerns	Major concerns	No concerns	No concerns	Very low
ARI vs LAM	0	Some concerns	Suspected	No concerns	Major concerns	No concerns	No concerns	Very low
ARI vs OLA	0	Some concerns	Suspected	No concerns	No concerns	No concerns	No concerns	Very low
ARI vs PAL	0	Some concerns	Suspected	No concerns	Major concerns	No concerns	No concerns	Very low
ARI vs QUE	0	Some concerns	Suspected	No concerns	Major concerns	No concerns	No concerns	Very low
ARI vs RIS	0	Some concerns	Suspected	No concerns	Major concerns	No concerns	No concerns	Very low
ARI vs TOP	0	Some concerns	Suspected	No concerns	Major concerns	No concerns	No concerns	Very low
ARI vs VALN	0	Some concerns	Suspected	No concerns	Major concerns	No concerns	No concerns	Very low
ARI vs VALP	0	Some concerns	Suspected	No concerns	Major concerns	No concerns	No concerns	Very low
ARI vs VER	0	Some concerns	Suspected	No concerns	Major concerns	No concerns	No concerns	Very low
ARI vs ZIP	0	Some concerns	Suspected	No concerns	Major concerns	No concerns	No concerns	Very low
ASE vs BRE	0	Some concerns	Suspected	No concerns	Major concerns	No concerns	No concerns	Very low

ASE vs CARB	0	Some concerns	Suspected	No concerns	Major concerns	No concerns	No concerns	Very low
ASE vs CARI	0	Some concerns	Suspected	No concerns	Major concerns	No concerns	No concerns	Very low
ASE vs END	0	No concerns	Suspected	No concerns	Major concerns	No concerns	No concerns	Very low
ASE vs HAL	0	Some concerns	Suspected	No concerns	Major concerns	No concerns	No concerns	Very low
ASE vs LAM	0	Some concerns	Suspected	No concerns	Major concerns	No concerns	No concerns	Very low
ASE vs LIT	0	Some concerns	Suspected	No concerns	Major concerns	No concerns	No concerns	Very low
ASE vs PAL	0	No concerns	Suspected	No concerns	Major concerns	No concerns	No concerns	Very low
ASE vs QUE	0	Some concerns	Suspected	No concerns	Major concerns	No concerns	No concerns	Very low
ASE vs RIS	0	Some concerns	Suspected	No concerns	Major concerns	No concerns	No concerns	Very low
ASE vs TOP	0	Some concerns	Suspected	No concerns	Major concerns	No concerns	No concerns	Very low
ASE vs VALN	0	Some concerns	Suspected	No concerns	Major concerns	No concerns	No concerns	Very low
ASE vs VALP	0	Some concerns	Suspected	No concerns	Major concerns	No concerns	No concerns	Very low
ASE vs VER	0	Some concerns	Suspected	No concerns	Major concerns	No concerns	No concerns	Very low
ASE vs ZIP	0	Some concerns	Suspected	No concerns	Major concerns	No concerns	No concerns	Very low
BRE vs CARB	0	Some concerns	Suspected	No concerns	Major concerns	No concerns	No concerns	Very low
BRE vs CARI	0	Some concerns	Suspected	No concerns	Major concerns	No concerns	No concerns	Very low
BRE vs END	0	Some concerns	Suspected	No concerns	Major concerns	No concerns	No concerns	Very low
BRE vs HAL	0	Some concerns	Suspected	No concerns	Major concerns	No concerns	No concerns	Very low
BRE vs LAM	0	Some concerns	Suspected	No concerns	Major concerns	No concerns	No concerns	Very low
BRE vs LIT	0	Some concerns	Suspected	No concerns	Major concerns	No concerns	No concerns	Very low
BRE vs OLA	0	Some concerns	Suspected	No concerns	No concerns	No concerns	No concerns	Very low
BRE vs PAL	0	No concerns	Suspected	No concerns	Major concerns	No concerns	No concerns	Very low
BRE vs QUE	0	Some concerns	Suspected	No concerns	Major concerns	No concerns	No concerns	Very low
BRE vs RIS	0	Some concerns	Suspected	No concerns	Major concerns	No concerns	No concerns	Very low
BRE vs TOP	0	Some concerns	Suspected	No concerns	Major concerns	No concerns	No concerns	Very low

BRE vs VALN	0	Some concerns	Suspected	No concerns	Major concerns	No concerns	No concerns	Very low
BRE vs VALP	0	Some concerns	Suspected	No concerns	Major concerns	No concerns	No concerns	Very low
BRE vs VER	0	Some concerns	Suspected	No concerns	Major concerns	No concerns	No concerns	Very low
BRE vs ZIP	0	Some concerns	Suspected	No concerns	Major concerns	No concerns	No concerns	Very low
CARB vs CARI	0	Some concerns	Suspected	No concerns	Major concerns	No concerns	No concerns	Very low
CARB vs END	0	Some concerns	Suspected	No concerns	Major concerns	No concerns	No concerns	Very low
CARB vs HAL	0	Some concerns	Suspected	No concerns	Major concerns	No concerns	No concerns	Very low
CARB vs LAM	0	Some concerns	Suspected	No concerns	Major concerns	No concerns	No concerns	Very low
CARB vs LIT	0	Some concerns	Suspected	No concerns	Major concerns	No concerns	No concerns	Very low
CARB vs OLA	0	Some concerns	Suspected	No concerns	Major concerns	No concerns	No concerns	Very low
CARB vs PAL	0	No concerns	Suspected	No concerns	Major concerns	No concerns	No concerns	Very low
CARB vs QUE	0	Some concerns	Suspected	No concerns	Major concerns	No concerns	No concerns	Very low
CARB vs RIS	0	Some concerns	Suspected	No concerns	Major concerns	No concerns	No concerns	Very low
CARB vs TOP	0	Some concerns	Suspected	No concerns	Major concerns	No concerns	No concerns	Very low
CARB vs VALN	0	Some concerns	Suspected	No concerns	Major concerns	No concerns	No concerns	Very low
CARB vs VALP	0	Some concerns	Suspected	No concerns	Major concerns	No concerns	No concerns	Very low
CARB vs VER	0	Some concerns	Suspected	No concerns	Major concerns	No concerns	No concerns	Very low
CARB vs ZIP	0	Some concerns	Suspected	No concerns	Major concerns	No concerns	No concerns	Very low
CARI vs END	0	Some concerns	Suspected	No concerns	Major concerns	No concerns	No concerns	Very low
CARI vs HAL	0	Some concerns	Suspected	No concerns	Major concerns	No concerns	No concerns	Very low
CARI vs LAM	0	Some concerns	Suspected	No concerns	Major concerns	No concerns	No concerns	Very low
CARI vs LIT	0	Some concerns	Suspected	No concerns	Major concerns	No concerns	No concerns	Very low
CARI vs OLA	0	Some concerns	Suspected	No concerns	No concerns	No concerns	No concerns	Very low
CARI vs PAL	0	No concerns	Suspected	No concerns	Major concerns	No concerns	No concerns	Very low
CARI vs QUE	0	Some concerns	Suspected	No concerns	Major concerns	No concerns	No concerns	Very low

CARI vs RIS	0	Some concerns	Suspected	No concerns	Major concerns	No concerns	No concerns	Very low
CARI vs TOP	0	Some concerns	Suspected	No concerns	Major concerns	No concerns	No concerns	Very low
CARI vs VALN	0	Some concerns	Suspected	No concerns	Major concerns	No concerns	No concerns	Very low
CARI vs VALP	0	Some concerns	Suspected	No concerns	Major concerns	No concerns	No concerns	Very low
CARI vs VER	0	Some concerns	Suspected	No concerns	Major concerns	No concerns	No concerns	Very low
CARI vs ZIP	0	Some concerns	Suspected	No concerns	Major concerns	No concerns	No concerns	Very low
END vs HAL	0	No concerns	Suspected	No concerns	Major concerns	No concerns	No concerns	Very low
END vs LAM	0	Some concerns	Suspected	No concerns	Major concerns	No concerns	No concerns	Very low
END vs LIT	0	No concerns	Suspected	No concerns	Major concerns	No concerns	No concerns	Very low
END vs OLA	0	No concerns	Suspected	No concerns	Major concerns	No concerns	No concerns	Very low
END vs PAL	0	No concerns	Suspected	No concerns	Major concerns	No concerns	No concerns	Very low
END vs QUE	0	No concerns	Suspected	No concerns	Major concerns	No concerns	No concerns	Very low
END vs RIS	0	No concerns	Suspected	No concerns	Major concerns	No concerns	No concerns	Very low
END vs TOP	0	No concerns	Suspected	No concerns	Major concerns	No concerns	No concerns	Very low
END vs VALN	0	No concerns	Suspected	No concerns	Major concerns	No concerns	No concerns	Very low
END vs VER	0	No concerns	Suspected	No concerns	Major concerns	No concerns	No concerns	Very low
END vs ZIP	0	No concerns	Suspected	No concerns	Major concerns	No concerns	No concerns	Very low
END vs PLA	0	No concerns	Suspected	No concerns	Major concerns	No concerns	No concerns	Very low
HAL vs LAM	0	Some concerns	Suspected	No concerns	Major concerns	No concerns	No concerns	Very low
HAL vs LIT	0	Some concerns	Suspected	No concerns	Major concerns	No concerns	No concerns	Very low
HAL vs PAL	0	Some concerns	Suspected	No concerns	Major concerns	No concerns	No concerns	Very low
HAL vs RIS	0	Some concerns	Suspected	No concerns	Major concerns	No concerns	No concerns	Very low
HAL vs TOP	0	Some concerns	Suspected	No concerns	Major concerns	No concerns	No concerns	Very low
HAL vs VALN	0	Some concerns	Suspected	No concerns	Major concerns	No concerns	No concerns	Very low
HAL vs VALP	0	Some concerns	Suspected	No concerns	Major concerns	No concerns	No concerns	Very low

HAL vs VER	0	Some concerns	Suspected	No concerns	Major concerns	No concerns	No concerns	Very low
LAM vs PAL	0	Some concerns	Suspected	No concerns	Major concerns	No concerns	No concerns	Very low
LAM vs QUE	0	Some concerns	Suspected	No concerns	Major concerns	No concerns	No concerns	Very low
LAM vs RIS	0	Some concerns	Suspected	No concerns	Major concerns	No concerns	No concerns	Very low
LAM vs TOP	0	Some concerns	Suspected	No concerns	Major concerns	No concerns	No concerns	Very low
LAM vs VALN	0	Some concerns	Suspected	No concerns	Major concerns	No concerns	No concerns	Very low
LAM vs VALP	0	Some concerns	Suspected	No concerns	Major concerns	No concerns	No concerns	Very low
LAM vs VER	0	Some concerns	Suspected	No concerns	Major concerns	No concerns	No concerns	Very low
LAM vs ZIP	0	Some concerns	Suspected	No concerns	Major concerns	No concerns	No concerns	Very low
LAM vs PLA	0	Some concerns	Suspected	No concerns	Major concerns	No concerns	No concerns	Very low
LIT vs PAL	0	No concerns	Suspected	No concerns	Major concerns	No concerns	No concerns	Very low
LIT vs QUE	0	Some concerns	Suspected	No concerns	Major concerns	No concerns	No concerns	Very low
LIT vs RIS	0	Some concerns	Suspected	No concerns	Major concerns	No concerns	No concerns	Very low
LIT vs VALN	0	Some concerns	Suspected	No concerns	Major concerns	No concerns	No concerns	Very low
LIT vs VER	0	No concerns	Suspected	No concerns	Major concerns	No concerns	No concerns	Very low
LIT vs ZIP	0	Some concerns	Suspected	No concerns	Major concerns	No concerns	No concerns	Very low
OLA vs PAL	0	No concerns	Suspected	No concerns	No concerns	No concerns	No concerns	Low
OLA vs QUE	0	No concerns	Suspected	No concerns	Major concerns	No concerns	No concerns	Very low
OLA vs TOP	0	No concerns	Suspected	No concerns	No concerns	No concerns	No concerns	Low
OLA vs VALN	0	Some concerns	Suspected	No concerns	Major concerns	No concerns	No concerns	Very low
OLA vs VER	0	No concerns	Suspected	No concerns	Major concerns	No concerns	No concerns	Very low
OLA vs ZIP	0	Some concerns	Suspected	No concerns	No concerns	No concerns	No concerns	Very low
PAL vs RIS	0	No concerns	Suspected	No concerns	Major concerns	No concerns	No concerns	Very low
PAL vs TOP	0	No concerns	Suspected	No concerns	Major concerns	No concerns	No concerns	Very low
PAL vs VALN	0	No concerns	Suspected	No concerns	Major concerns	No concerns	No concerns	Very low

PAL vs VALP	0	No concerns	Suspected	No concerns	Major concerns	No concerns	No concerns	Very low
PAL vs VER	0	No concerns	Suspected	No concerns	Major concerns	No concerns	No concerns	Very low
PAL vs ZIP	0	No concerns	Suspected	No concerns	Major concerns	No concerns	No concerns	Very low
QUE vs RIS	0	Some concerns	Suspected	No concerns	Major concerns	No concerns	No concerns	Very low
QUE vs TOP	0	No concerns	Suspected	No concerns	Major concerns	No concerns	No concerns	Very low
QUE vs VALN	0	No concerns	Suspected	No concerns	Major concerns	No concerns	No concerns	Very low
QUE vs VALP	0	No concerns	Suspected	No concerns	Major concerns	No concerns	No concerns	Very low
QUE vs VER	0	No concerns	Suspected	No concerns	Major concerns	No concerns	No concerns	Very low
QUE vs ZIP	0	Some concerns	Suspected	No concerns	Major concerns	No concerns	No concerns	Very low
RIS vs TOP	0	Some concerns	Suspected	No concerns	Major concerns	No concerns	No concerns	Very low
RIS vs VALP	0	Some concerns	Suspected	No concerns	Major concerns	No concerns	No concerns	Very low
RIS vs VER	0	No concerns	Suspected	No concerns	Major concerns	No concerns	No concerns	Very low
RIS vs ZIP	0	Some concerns	Suspected	No concerns	Major concerns	No concerns	No concerns	Very low
TOP vs VALN	0	No concerns	Suspected	No concerns	Major concerns	No concerns	No concerns	Very low
TOP vs VALP	0	No concerns	Suspected	No concerns	Major concerns	No concerns	No concerns	Very low
TOP vs VER	0	No concerns	Suspected	No concerns	Major concerns	No concerns	No concerns	Very low
TOP vs ZIP	0	No concerns	Suspected	No concerns	Major concerns	No concerns	No concerns	Very low
VALN vs VALP	0	No concerns	Suspected	No concerns	Major concerns	No concerns	No concerns	Very low
VALN vs VER	0	No concerns	Suspected	No concerns	Major concerns	No concerns	No concerns	Very low
VALN vs ZIP	0	Some concerns	Suspected	No concerns	Major concerns	No concerns	No concerns	Very low
VALP vs VER	0	No concerns	Suspected	No concerns	Major concerns	No concerns	No concerns	Very low
VALP vs ZIP	0	Some concerns	Suspected	No concerns	Major concerns	No concerns	No concerns	Very low
VER vs ZIP	0	No concerns	Suspected	No concerns	Major concerns	No concerns	No concerns	Very low

Supplementary Appendix 9. Depression (N = 19, n = 5740)

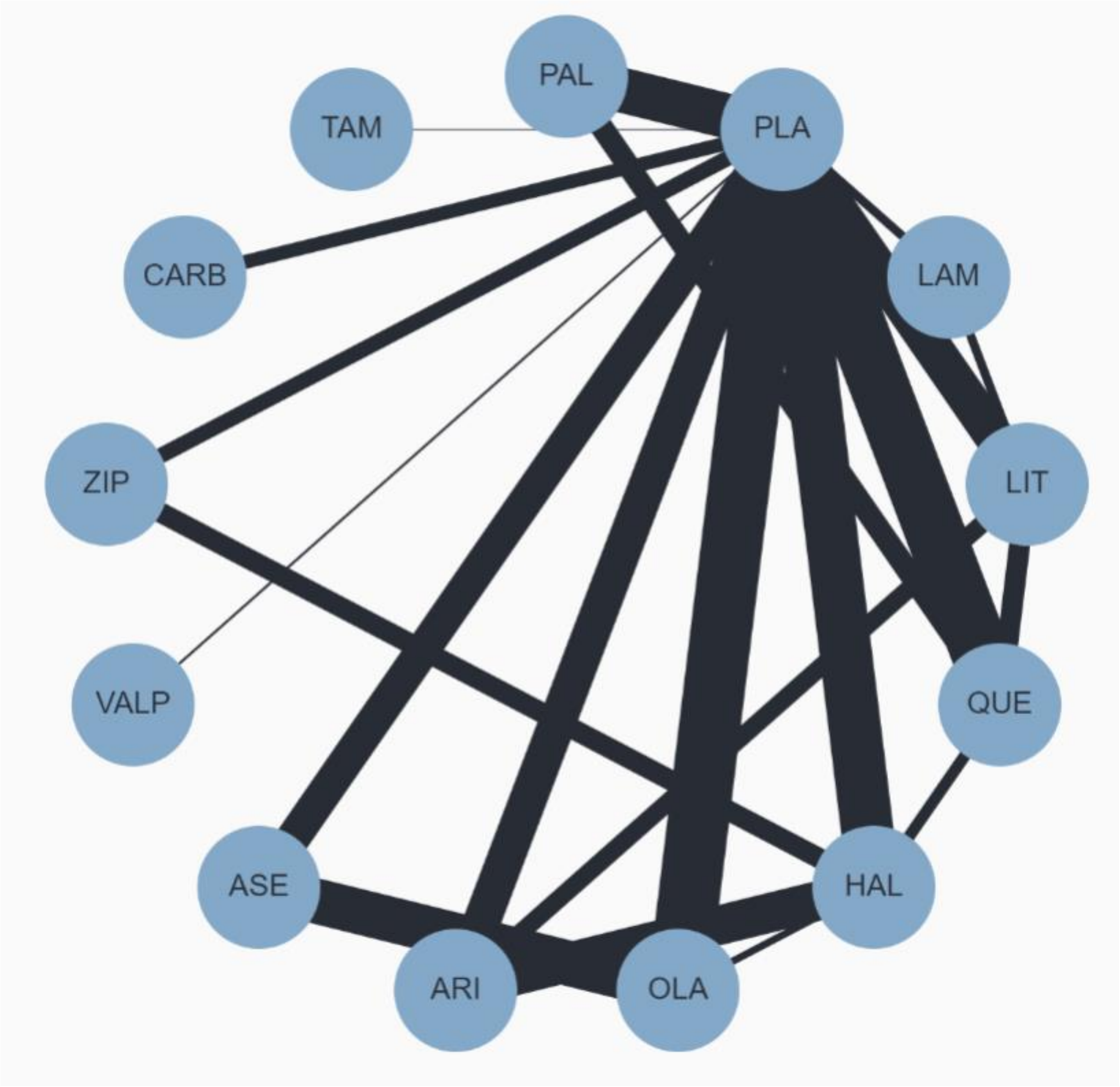

League table (risk ratio with 95% confidence interval)

ARI	2.291 (0.447, 11.754)	0.563 (0.040, 7.850)	0.796 (0.388, 1.634)	1.108 (0.169, 7.241)	1.269 (0.533, 3.021)	1.867 (0.718, 4.857)	1.270 (0.443, 3.637)	1.968 (0.811, 4.776)	0.720 (0.111, 4.656)	0.314 (0.011, 8.839)	3.704 (0.595, 23.042)	1.081 (0.542, 2.154)
	ASE	0.246 (0.013, 4.683)	0.347 (0.068, 1.767)	0.483 (0.048, 4.848)	0.554 (0.104, 2.948)	0.815 (0.182, 3.649)	0.554 (0.102, 3.012)	0.859 (0.171, 4.306)	0.314 (0.032, 3.093)	0.137 (0.004, 4.963)	1.617 (0.158, 16.563)	0.472 (0.106, 2.093)
		CARB	1.413 (0.101, 19.674)	1.966 (0.089, 43.285)	2.252 (0.158, 32.024)	3.314 (0.237, 46.247)	2.254 (0.157, 32.431)	3.493 (0.255, 47.832)	1.279 (0.059, 27.752)	0.558 (0.009, 34.956)	6.574 (0.292, 148.028)	1.918 (0.151, 24.378)
			HAL	1.392 (0.212, 9.150)	1.595 (0.615, 4.132)	2.346 (0.929, 5.924)	1.596 (0.559, 4.554)	**2.473 (1.036, 5.901)**	0.905 (0.140, 5.847)	0.395 (0.014, 11.104)	4.654 (0.848, 25.541)	1.358 (0.682, 2.703)
				LAM	1.146 (0.176, 7.443)	1.686 (0.254, 11.174)	1.147 (0.166, 7.924)	1.777 (0.277, 11.407)	0.650 (0.055, 7.692)	0.284 (0.007, 11.574)	3.344 (0.271, 41.236)	0.976 (0.168, 5.667)
					LIT	1.471 (0.528, 4.101)	1.001 (0.335, 2.992)	1.551 (0.628, 3.833)	0.568 (0.085, 3.775)	0.248 (0.009, 7.077)	2.919 (0.428, 19.924)	0.852 (0.397, 1.827)
						OLA	0.680 (0.235, 1.967)	1.054 (0.418, 2.661)	0.386 (0.060, 2.500)	0.168 (0.006, 4.741)	1.984 (0.295, 13.346)	0.579 (0.289, 1.161)
							PAL	1.550 (0.607, 3.957)	0.567 (0.084, 3.836)	0.247 (0.009, 7.139)	2.917 (0.408, 20.822)	0.851 (0.381, 1.901)
								QUE	0.366 (0.058, 2.308)	0.160 (0.006, 4.430)	1.882 (0.287, 12.326)	0.549 (0.295, 1.020)
									TAM	0.436 (0.011, 17.584)	5.141 (0.423, 62.527)	1.500 (0.265, 8.495)
										VALP	11.787 (0.284, 490.046)	3.439 (0.131, 90.017)
											ZIP	0.292 (0.048, 1.763)

												PLA

Evaluation of heterogeneity and inconsistency

Between study variance (τ^2)	Heterogeneity assessment	Random-effects design-by-treatment interaction model		
		Q	df	p
0.199	High	23.930	14	0.047

Incoherence

	NMA, RR (95% CI)	Direct, RR (95% CI)	I^2	Indirect, RR (95% CI)	Inconsistency measures	
					Difference of RR	P value
ARI vs HAL	0.796 (0.388, 1.634)	1.249 (0.528, 2.952)	75.0%	0.279 (0.075, 1.037)	4.469 (0.931, 21.447)	0.061
ARI vs LIT	1.269 (0.533, 3.021)	0.700 (0.243, 2.021)	na	4.235 (0.937, 19.144)	0.165 (0.026, 1.045)	0.056
ARI vs PLA	1.081 (0.542, 2.154)	1.015 (0.446, 2.312)	0.0%	1.252 (0.354, 4.426)	0.811 (0.180, 3.658)	0.785
ASE vs OLA	0.815 (0.182, 3.649)	0.857 (0.160, 4.587)	9.7%	0.669 (0.024, 18.831)	1.281 (0.031, 53.737)	0.896
ASE vs PLA	0.472 (0.106, 2.093)	0.372 (0.071, 1.954)	56.0%	1.274 (0.043, 38.103)	0.292 (0.007, 12.817)	0.524
CARB vs PLA		1.918 (0.151, 24.378)	na			
HAL vs OLA	2.346 (0.929, 5.924)	7.875 (1.139, 54.445)	na	1.636 (0.569, 4.698)	4.815 (0.532, 43.576)	0.162
HAL vs QUE	2.473 (1.036, 5.901)	2.748 (0.574, 13.144)	na	2.360 (0.829, 6.716)	1.164 (0.177, 7.651)	0.874
HAL vs ZIP	4.654 (0.848, 25.541)	5.725 (1.015, 32.285)	na	0.007 (0.000, 114.069)	783.679 (0.043, 14270428.257)	0.183
HAL vs PLA	1.358 (0.682, 2.703)	1.333 (0.573, 3.103)	70.2%	1.408 (0.430, 4.614)	0.946 (0.221, 4.063)	0.941
LAM vs LIT	1.146 (0.176, 7.443)	5.268 (0.227, 122.217)	na	0.496 (0.048, 5.093)	10.619 (0.212, 531.179)	0.237
LAM vs PLA	0.976 (0.168, 5.667)	0.743 (0.121, 4.569)	na	61.590 (0.052, 73629.423)	0.012 (0.000, 18.136)	0.237
LIT vs QUE	1.551 (0.628, 3.833)	0.760 (0.178, 3.248)	0.0%	2.439 (0.767, 7.758)	0.312 (0.049, 1.996)	0.218
LIT vs PLA	0.852 (0.397, 1.827)	0.739 (0.322, 1.700)	54.0%	1.793 (0.265, 12.135)	0.412 (0.051, 3.319)	0.405
OLA vs PLA	0.579 (0.289, 1.161)	0.703 (0.343, 1.441)	0.0%	0.025 (0.001, 0.450)	27.586 (1.430, 532.083)	0.028
PAL vs QUE	1.550 (0.607, 3.957)	1.583 (0.388, 6.455)	na	1.523 (0.433, 5.361)	1.039 (0.158, 6.855)	0.968
PAL vs PLA	0.851 (0.381, 1.901)	0.794 (0.345, 1.829)	56.0%	2.057 (0.105, 40.472)	0.386 (0.017, 8.519)	0.547

QUE vs PLA	0.549 (0.295, 1.020)	0.483 (0.252, 0.926)	0.0%	1.910 (0.250, 14.569)	0.253 (0.030, 2.136)	0.207
TAM vs PLA		1.500 (0.265, 8.495)	na			
VALP vs PLA		3.439 (0.131, 90.017)	na			
ZIP vs PLA	0.292 (0.048, 1.763)	0.989 (0.078, 12.565)	na	0.086 (0.007, 1.094)	11.516 (0.316, 420.313)	0.183

For some outcomes of a network meta-analysis, odds ratio may be considered a more appropriate effect size than risk ratio (Huhn 2020). For example, odds ratio has mathematical properties that make it more appropriate for some outcome of network meta-analysis (e.g. the odds ratio is symmetrical). Odds ratio may also be a better effect size when the definition of an outcome differs between RCTs. In our study, there were the differences in the definition of the outcome among RCTs. Therefore, although we performed a network meta-analysis for this outcome using odds ratio, the results also had global heterogeneity (τ^2 = 0.226) and global inconsistency (p = 0.043). These results are reported for presentation purposes only because these analyses were not included in a prespecified analysis plan; thus, further study will be needed to explore the reasons for the global heterogeneity and the global inconsistency.

Huhn M, et al. Lancet 2019; 394(10202): 939-951.

Forest plot (vs placebo, the numbers are risk ratio with 95% confidence interval)

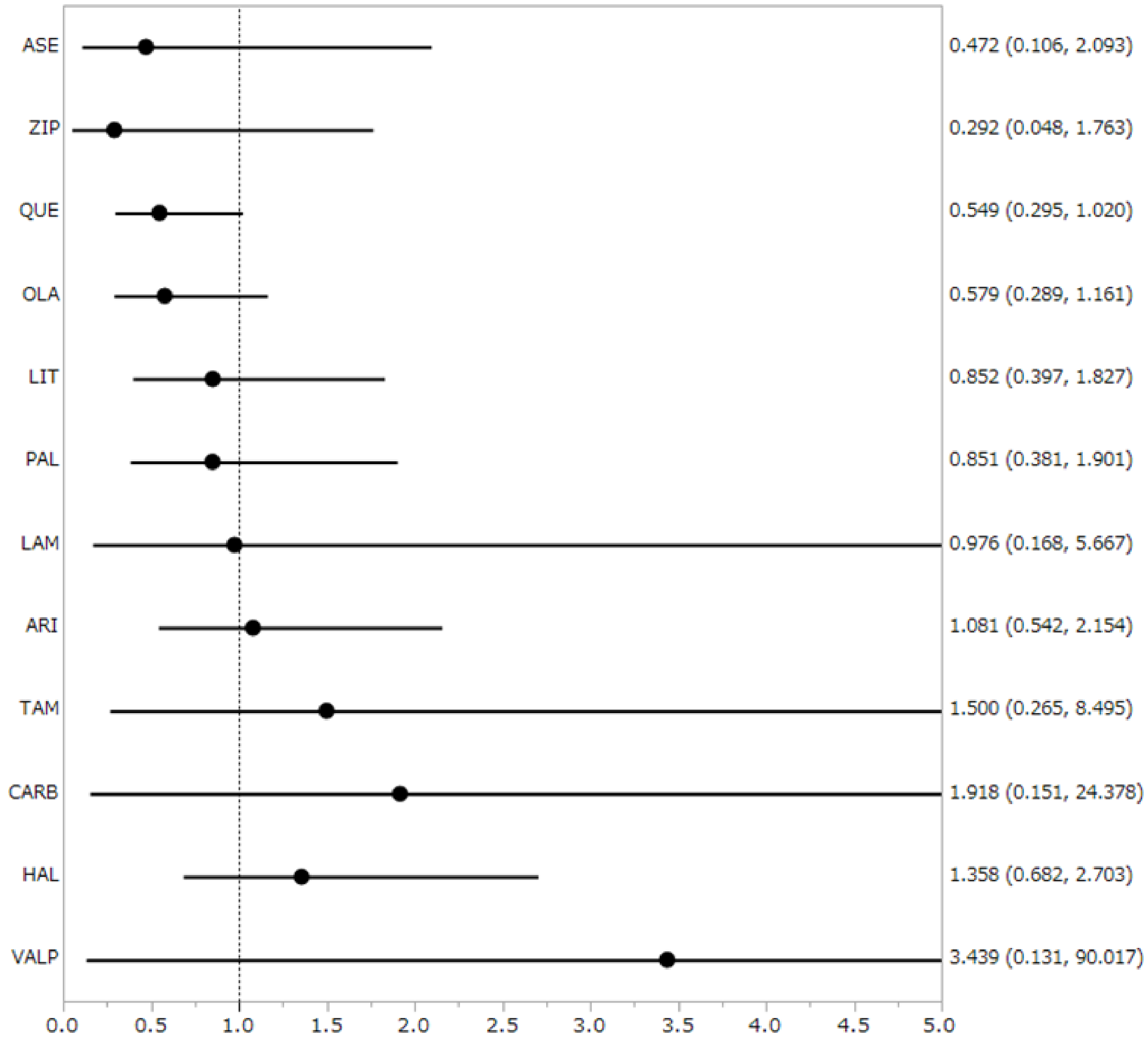

P-score

ASE	0.847
ZIP	0.805
QUE	0.754
OLA	0.686
LIT	0.601
PAL	0.532
LAM	0.429
ARI	0.414
TAM	0.343
CARB	0.320
HAL	0.301
VALP	0.023

Funnel plot (only double-blind, placebo-controlled trials)

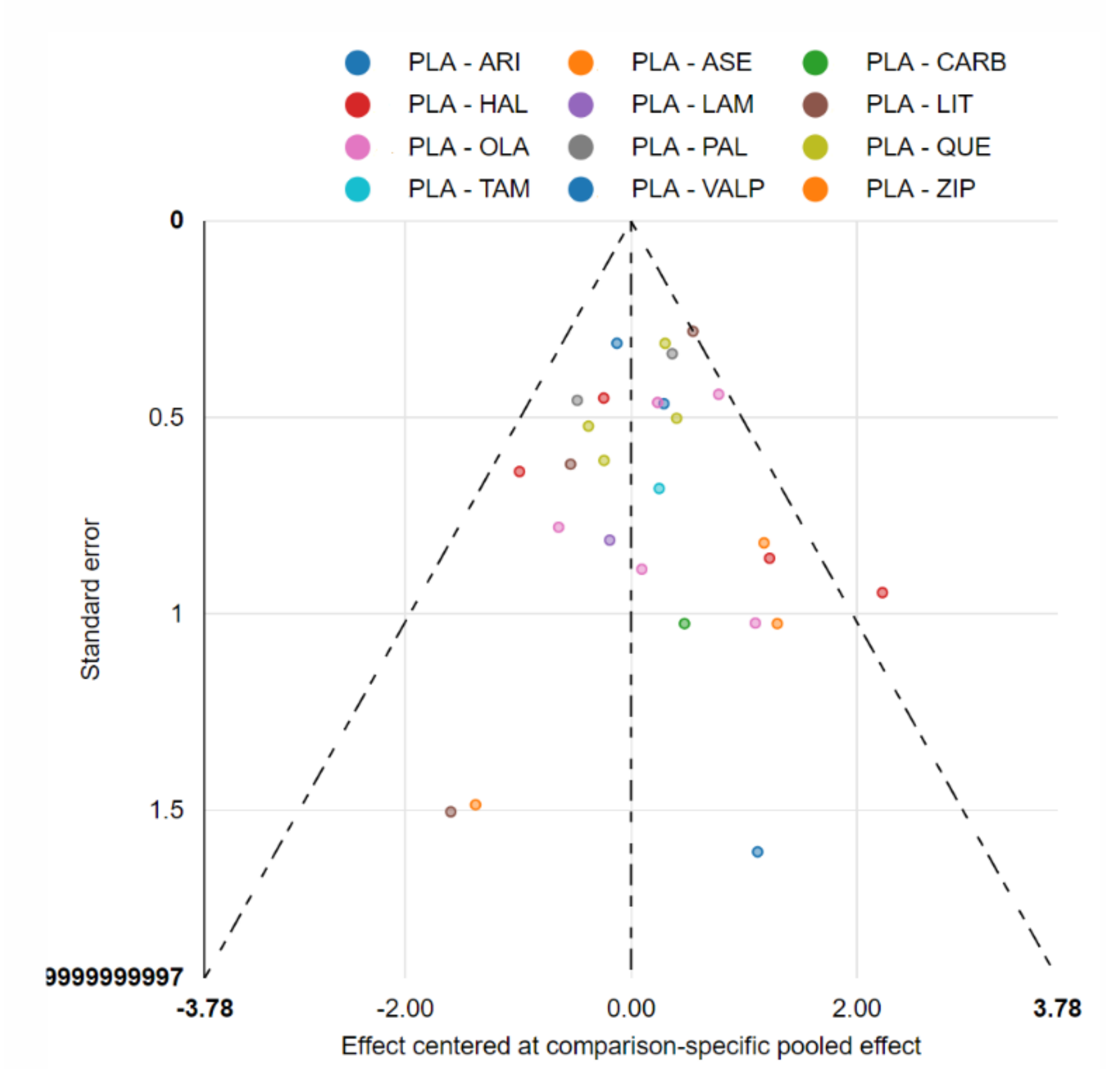

CINeMA confidence rating

Comparison	Number of studies	Within-study bias	Reporting bias	Indirectness	Imprecision	Heterogeneity	Incoherence	Confidence rating
ARI vs HAL	2	Some concerns	Suspected	No concerns	Major concerns	No concerns	No concerns	Very low
ARI vs LIT	1	Some concerns	Suspected	No concerns	Major concerns	No concerns	No concerns	Very low
ARI vs PLA	2	Some concerns	Suspected	No concerns	Major concerns	No concerns	No concerns	Very low
ASE vs OLA	2	Some concerns	Suspected	No concerns	Major concerns	No concerns	No concerns	Very low
ASE vs PLA	2	Some concerns	Suspected	No concerns	Major concerns	No concerns	No concerns	Very low
CARB vs PLA	1	Some concerns	Suspected	No concerns	Major concerns	No concerns	Major concerns	Very low
HAL vs OLA	1	Some concerns	Suspected	No concerns	Major concerns	No concerns	No concerns	Very low
HAL vs QUE	1	Some concerns	Suspected	No concerns	No concerns	Major concerns	No concerns	Very low
HAL vs ZIP	1	Some concerns	Suspected	No concerns	Major concerns	No concerns	No concerns	Very low
HAL vs PLA	4	Some concerns	Suspected	No concerns	Major concerns	No concerns	No concerns	Very low
LAM vs LIT	1	Some concerns	Suspected	No concerns	Major concerns	No concerns	No concerns	Very low
LAM vs PLA	1	Some concerns	Suspected	No concerns	Major concerns	No concerns	No concerns	Very low
LIT vs QUE	2	No concerns	Suspected	No concerns	Major concerns	No concerns	No concerns	Low
LIT vs PLA	3	Some concerns	Suspected	No concerns	Major concerns	No concerns	No concerns	Very low
OLA vs PLA	5	Some concerns	Suspected	No concerns	Major concerns	No concerns	Major concerns	Very low
PAL vs QUE	1	No concerns	Suspected	No concerns	Major concerns	No concerns	No concerns	Low
PAL vs PLA	2	No concerns	Suspected	No concerns	Major concerns	No concerns	No concerns	Low
QUE vs PLA	4	No concerns	Suspected	No concerns	Major concerns	No concerns	No concerns	Low
TAM vs PLA	1	No concerns	Suspected	No concerns	Major concerns	No concerns	Major concerns	Very low
VALP vs PLA	1	No concerns	Suspected	No concerns	Major concerns	No concerns	Major concerns	Very low
ZIP vs PLA	1	Some concerns	Suspected	No concerns	Major concerns	No concerns	No concerns	Very low
ARI vs ASE	0	Some concerns	Suspected	No concerns	Major concerns	No concerns	Major concerns	Very low
ARI vs CARB	0	Some concerns	Suspected	No concerns	Major concerns	No concerns	Major concerns	Very low

ARI vs LAM	0	Some concerns	Suspected	No concerns	Major concerns	No concerns	Major concerns	Very low
ARI vs OLA	0	Some concerns	Suspected	No concerns	Major concerns	No concerns	Major concerns	Very low
ARI vs PAL	0	Some concerns	Suspected	No concerns	Major concerns	No concerns	Major concerns	Very low
ARI vs QUE	0	Some concerns	Suspected	No concerns	Major concerns	No concerns	Major concerns	Very low
ARI vs TAM	0	Some concerns	Suspected	No concerns	Major concerns	No concerns	Major concerns	Very low
ARI vs VALP	0	Some concerns	Suspected	No concerns	Major concerns	No concerns	Major concerns	Very low
ARI vs ZIP	0	Some concerns	Suspected	No concerns	Major concerns	No concerns	Major concerns	Very low
ASE vs CARB	0	Some concerns	Suspected	No concerns	Major concerns	No concerns	Major concerns	Very low
ASE vs HAL	0	Some concerns	Suspected	No concerns	Major concerns	No concerns	Major concerns	Very low
ASE vs LAM	0	Some concerns	Suspected	No concerns	Major concerns	No concerns	Major concerns	Very low
ASE vs LIT	0	Some concerns	Suspected	No concerns	Major concerns	No concerns	Major concerns	Very low
ASE vs PAL	0	Some concerns	Suspected	No concerns	Major concerns	No concerns	Major concerns	Very low
ASE vs QUE	0	Some concerns	Suspected	No concerns	Major concerns	No concerns	Major concerns	Very low
ASE vs TAM	0	Some concerns	Suspected	No concerns	Major concerns	No concerns	Major concerns	Very low
ASE vs VALP	0	Some concerns	Suspected	No concerns	Major concerns	No concerns	Major concerns	Very low
ASE vs ZIP	0	Some concerns	Suspected	No concerns	Major concerns	No concerns	Major concerns	Very low
CARB vs HAL	0	Some concerns	Suspected	No concerns	Major concerns	No concerns	Major concerns	Very low
CARB vs LAM	0	Some concerns	Suspected	No concerns	Major concerns	No concerns	Major concerns	Very low
CARB vs LIT	0	Some concerns	Suspected	No concerns	Major concerns	No concerns	Major concerns	Very low
CARB vs OLA	0	Some concerns	Suspected	No concerns	Major concerns	No concerns	Major concerns	Very low
CARB vs PAL	0	No concerns	Suspected	No concerns	Major concerns	No concerns	Major concerns	Very low
CARB vs QUE	0	Some concerns	Suspected	No concerns	Major concerns	No concerns	Major concerns	Very low
CARB vs TAM	0	Some concerns	Suspected	No concerns	Major concerns	No concerns	Major concerns	Very low
CARB vs VALP	0	Some concerns	Suspected	No concerns	Major concerns	No concerns	Major concerns	Very low
CARB vs ZIP	0	Some concerns	Suspected	No concerns	Major concerns	No concerns	Major concerns	Very low

HAL vs LAM	0	Some concerns	Suspected	No concerns	Major concerns	No concerns	Major concerns	Very low
HAL vs LIT	0	Some concerns	Suspected	No concerns	Major concerns	No concerns	Major concerns	Very low
HAL vs PAL	0	Some concerns	Suspected	No concerns	Major concerns	No concerns	Major concerns	Very low
HAL vs TAM	0	Some concerns	Suspected	No concerns	Major concerns	No concerns	Major concerns	Very low
HAL vs VALP	0	Some concerns	Suspected	No concerns	Major concerns	No concerns	Major concerns	Very low
LAM vs OLA	0	Some concerns	Suspected	No concerns	Major concerns	No concerns	Major concerns	Very low
LAM vs PAL	0	Some concerns	Suspected	No concerns	Major concerns	No concerns	Major concerns	Very low
LAM vs QUE	0	Some concerns	Suspected	No concerns	Major concerns	No concerns	Major concerns	Very low
LAM vs TAM	0	Some concerns	Suspected	No concerns	Major concerns	No concerns	Major concerns	Very low
LAM vs VALP	0	Some concerns	Suspected	No concerns	Major concerns	No concerns	Major concerns	Very low
LAM vs ZIP	0	Some concerns	Suspected	No concerns	Major concerns	No concerns	Major concerns	Very low
LIT vs OLA	0	Some concerns	Suspected	No concerns	Major concerns	No concerns	Major concerns	Very low
LIT vs PAL	0	No concerns	Suspected	No concerns	Major concerns	No concerns	Major concerns	Very low
LIT vs TAM	0	No concerns	Suspected	No concerns	Major concerns	No concerns	Major concerns	Very low
LIT vs VALP	0	No concerns	Suspected	No concerns	Major concerns	No concerns	Major concerns	Very low
LIT vs ZIP	0	Some concerns	Suspected	No concerns	Major concerns	No concerns	Major concerns	Very low
OLA vs PAL	0	Some concerns	Suspected	No concerns	Major concerns	No concerns	Major concerns	Very low
OLA vs QUE	0	Some concerns	Suspected	No concerns	Major concerns	No concerns	Major concerns	Very low
OLA vs TAM	0	Some concerns	Suspected	No concerns	Major concerns	No concerns	Major concerns	Very low
OLA vs VALP	0	Some concerns	Suspected	No concerns	Major concerns	No concerns	Major concerns	Very low
OLA vs ZIP	0	Some concerns	Suspected	No concerns	Major concerns	No concerns	Major concerns	Very low
PAL vs TAM	0	No concerns	Suspected	No concerns	Major concerns	No concerns	Major concerns	Very low
PAL vs VALP	0	No concerns	Suspected	No concerns	Major concerns	No concerns	Major concerns	Very low
PAL vs ZIP	0	Some concerns	Suspected	No concerns	Major concerns	No concerns	Major concerns	Very low
QUE vs TAM	0	No concerns	Suspected	No concerns	Major concerns	No concerns	Major concerns	Very low

QUE vs VALP	0	No concerns	Suspected	No concerns	Major concerns	No concerns	Major concerns	Very low
QUE vs ZIP	0	Some concerns	Suspected	No concerns	Major concerns	No concerns	Major concerns	Very low
TAM vs VALP	0	No concerns	Suspected	No concerns	Major concerns	No concerns	Major concerns	Very low
TAM vs ZIP	0	Some concerns	Suspected	No concerns	Major concerns	No concerns	Major concerns	Very low
VALP vs ZIP	0	Some concerns	Suspected	No concerns	Major concerns	No concerns	Major concerns	Very low

Supplementary Appendix 10. Use of anxiolytics (N = 28, n = 8082)

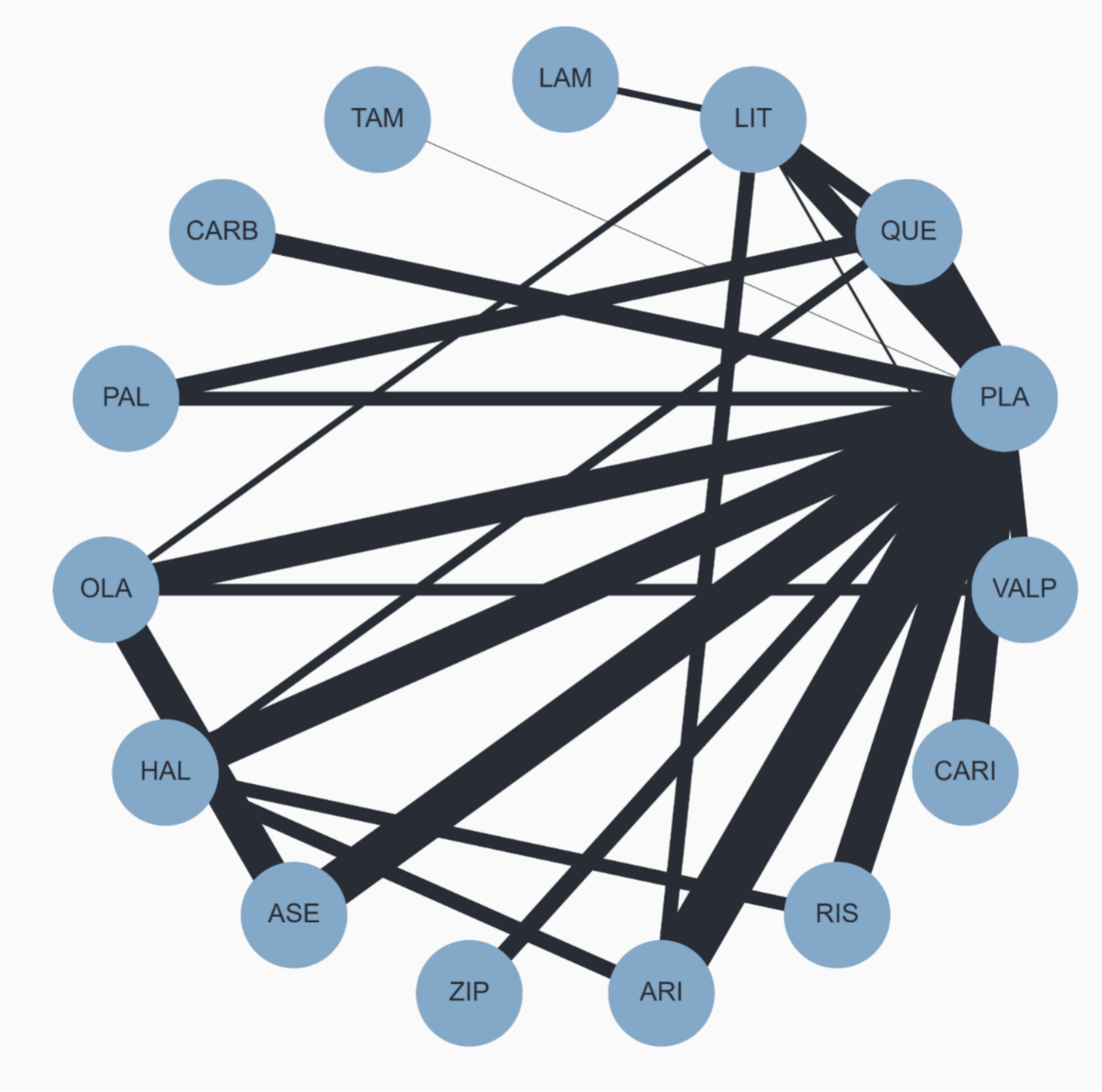

League table (risk ratio with 95% confidence interval)

ARI	1.107 (0.969, 1.265)	1.041 (0.901, 1.203)	1.041 (0.914, 1.185)	1.032 (0.886, 1.203)	0.954 (0.814, 1.117)	1.067 (0.975, 1.169)	**1.173 (1.047, 1.314)**	1.181 (0.962, 1.450)	**1.347 (1.151, 1.578)**	1.042 (0.913, 1.189)	1.550 (0.692, 3.474)	1.132 (0.996, 1.286)	0.990 (0.855, 1.146)	1.033 (0.964, 1.108)
	ASE	0.940 (0.792, 1.116)	0.940 (0.801, 1.103)	0.932 (0.772, 1.125)	0.861 (0.714, 1.039)	0.964 (0.841, 1.104)	1.059 (0.936, 1.198)	1.066 (0.851, 1.336)	**1.217 (1.012, 1.462)**	0.941 (0.799, 1.107)	1.400 (0.621, 3.154)	1.022 (0.877, 1.191)	0.894 (0.752, 1.063)	0.933 (0.831, 1.048)
		CARB	1.000 (0.846, 1.182)	0.992 (0.816, 1.206)	0.916 (0.750, 1.118)	1.025 (0.881, 1.193)	1.127 (0.961, 1.321)	1.134 (0.900, 1.429)	**1.294 (1.069, 1.567)**	1.001 (0.844, 1.187)	1.489 (0.660, 3.360)	1.087 (0.919, 1.286)	0.951 (0.794, 1.139)	0.993 (0.875, 1.126)
			CARI	0.992 (0.824, 1.193)	0.916 (0.758, 1.107)	1.025 (0.893, 1.178)	1.127 (0.973, 1.304)	1.134 (0.908, 1.417)	**1.294 (1.081, 1.550)**	1.001 (0.854, 1.173)	1.489 (0.662, 3.353)	1.087 (0.930, 1.271)	0.951 (0.803, 1.127)	0.993 (0.890, 1.108)
				HAL	0.924 (0.748, 1.141)	1.034 (0.875, 1.222)	1.136 (0.953, 1.355)	1.144 (0.896, 1.460)	**1.305 (1.063, 1.603)**	1.009 (0.848, 1.201)	1.502 (0.663, 3.401)	1.096 (0.911, 1.319)	0.959 (0.788, 1.168)	1.001 (0.863, 1.162)
					LAM	1.119 (0.984, 1.274)	**1.230 (1.041, 1.454)**	1.238 (0.968, 1.584)	**1.413 (1.149, 1.738)**	1.093 (0.902, 1.323)	1.626 (0.717, 3.687)	1.187 (0.990, 1.423)	1.038 (0.849, 1.270)	1.084 (0.929, 1.265)
						LIT	1.099 (0.988, 1.222)	1.106 (0.897, 1.364)	**1.262 (1.074, 1.483)**	0.976 (0.847, 1.124)	1.452 (0.647, 3.260)	1.060 (0.933, 1.205)	0.928 (0.795, 1.082)	0.968 (0.890, 1.053)
							OLA	1.007 (0.811, 1.249)	1.149 (0.968, 1.363)	0.888 (0.765, 1.031)	1.322 (0.588, 2.970)	0.965 (0.851, 1.093)	**0.844 (0.719, 0.992)**	**0.881 (0.800, 0.971)**
								PAL	1.141 (0.939, 1.387)	0.882 (0.705, 1.105)	1.313 (0.574, 3.002)	0.958 (0.767, 1.198)	0.839 (0.665, 1.058)	0.875 (0.721, 1.062)
									QUE	**0.773 (0.644, 0.928)**	1.151 (0.508, 2.604)	0.840 (0.701, 1.006)	**0.735 (0.606, 0.891)**	**0.767 (0.665, 0.885)**
										RIS	1.488 (0.661, 3.352)	1.086 (0.926, 1.274)	0.950 (0.800, 1.129)	0.992 (0.885, 1.112)
											TAM	0.730 (0.324, 1.644)	0.639 (0.283, 1.442)	0.667 (0.298, 1.490)

												VALP	0.875 (0.738, 1.037)	0.913 (0.817, 1.021)
													ZIP	1.044 (0.918, 1.187)
														PLA

Evaluation of heterogeneity and inconsistency

Between study variance (τ^2)	Heterogeneity assessment	Random-effects design-by-treatment interaction model		
		Q	df	p
0.002	Low	20.451	15	0.155

Incoherence

	NMA, RR (95% CI)	Direct, RR (95% CI)	I^2	Indirect, RR (95% CI)	Inconsistency measures	
					Difference of RR	P value
ARI vs HAL	1.032 (0.886, 1.203)	1.224 (1.005, 1.490)	na	0.800 (0.628, 1.018)	1.530 (1.121, 2.090)	0.007
ARI vs LIT	1.067 (0.975, 1.169)	1.032 (0.927, 1.149)	na	1.160 (0.979, 1.374)	0.890 (0.728, 1.087)	0.254
ARI vs PLA	1.033 (0.964, 1.108)	1.033 (0.962, 1.110)	0.0%	1.037 (0.787, 1.366)	0.996 (0.750, 1.324)	0.979
ASE vs OLA	1.059 (0.936, 1.198)	1.078 (0.933, 1.247)	0.0%	1.011 (0.802, 1.275)	1.067 (0.811, 1.403)	0.645
ASE vs PLA	0.933 (0.831, 1.048)	0.917 (0.809, 1.039)	0.0%	1.041 (0.762, 1.421)	0.881 (0.630, 1.232)	0.459
CARB vs PLA		0.993 (0.875, 1.126)	0.0%			
CARI vs PLA		0.993 (0.890, 1.108)	66.1%			
HAL vs QUE	1.305 (1.063, 1.603)	4.808 (1.422, 16.260)	na	1.256 (1.020, 1.547)	3.827 (1.112, 13.173)	0.033
HAL vs RIS	1.009 (0.848, 1.201)	1.167 (0.866, 1.571)	na	0.935 (0.754, 1.160)	1.247 (0.864, 1.800)	0.238
HAL vs PLA	1.001 (0.863, 1.162)	1.044 (0.878, 1.240)	65.1%	0.886 (0.659, 1.190)	1.178 (0.837, 1.658)	0.346
LAM vs LIT		1.119 (0.984, 1.274)	na			
LIT vs OLA	1.099 (0.988, 1.222)	1.074 (0.918, 1.257)	na	1.120 (0.970, 1.293)	0.959 (0.775, 1.187)	0.701
LIT vs QUE	1.262 (1.074, 1.483)	1.001 (0.723, 1.384)	0.0%	1.363 (1.131, 1.641)	0.734 (0.505, 1.067)	0.105
LIT vs VALP	1.060 (0.933, 1.205)	1.089 (0.798, 1.486)	na	1.054 (0.917, 1.213)	1.033 (0.734, 1.452)	0.853
LIT vs PLA	0.968 (0.890, 1.053)	0.973 (0.876, 1.081)	81.8%	0.959 (0.834, 1.104)	1.014 (0.851, 1.209)	0.873
OLA vs VALP	0.965 (0.851, 1.093)	0.996 (0.819, 1.211)	na	0.944 (0.802, 1.110)	1.055 (0.818, 1.360)	0.678
OLA vs PLA	0.881 (0.800, 0.971)	0.850 (0.743, 0.973)	0.0%	0.915 (0.796, 1.051)	0.930 (0.766, 1.129)	0.462

PAL vs QUE	1.141 (0.939, 1.387)	1.168 (0.941, 1.448)	na	1.025 (0.643, 1.634)	1.140 (0.682, 1.905)	0.618
PAL vs PLA	0.875 (0.721, 1.062)	0.857 (0.693, 1.058)	na	0.980 (0.604, 1.590)	0.874 (0.515, 1.483)	0.618
QUE vs PLA	0.767 (0.665, 0.885)	0.745 (0.643, 0.863)	1.3%	1.344 (0.707, 2.555)	0.554 (0.287, 1.071)	0.079
RIS vs PLA	0.992 (0.885, 1.112)	1.018 (0.906, 1.144)	0.0%	0.569 (0.331, 0.980)	1.788 (1.026, 3.116)	0.040
TAM vs PLA		0.667 (0.298, 1.490)	na			
VALP vs PLA	0.913 (0.817, 1.021)	0.933 (0.812, 1.072)	na	0.879 (0.729, 1.059)	1.062 (0.842, 1.340)	0.613
ZIP vs PLA		1.044 (0.918, 1.187)	16.3%			

Forest plot (vs placebo, the numbers are risk ratio with 95% confidence interval)

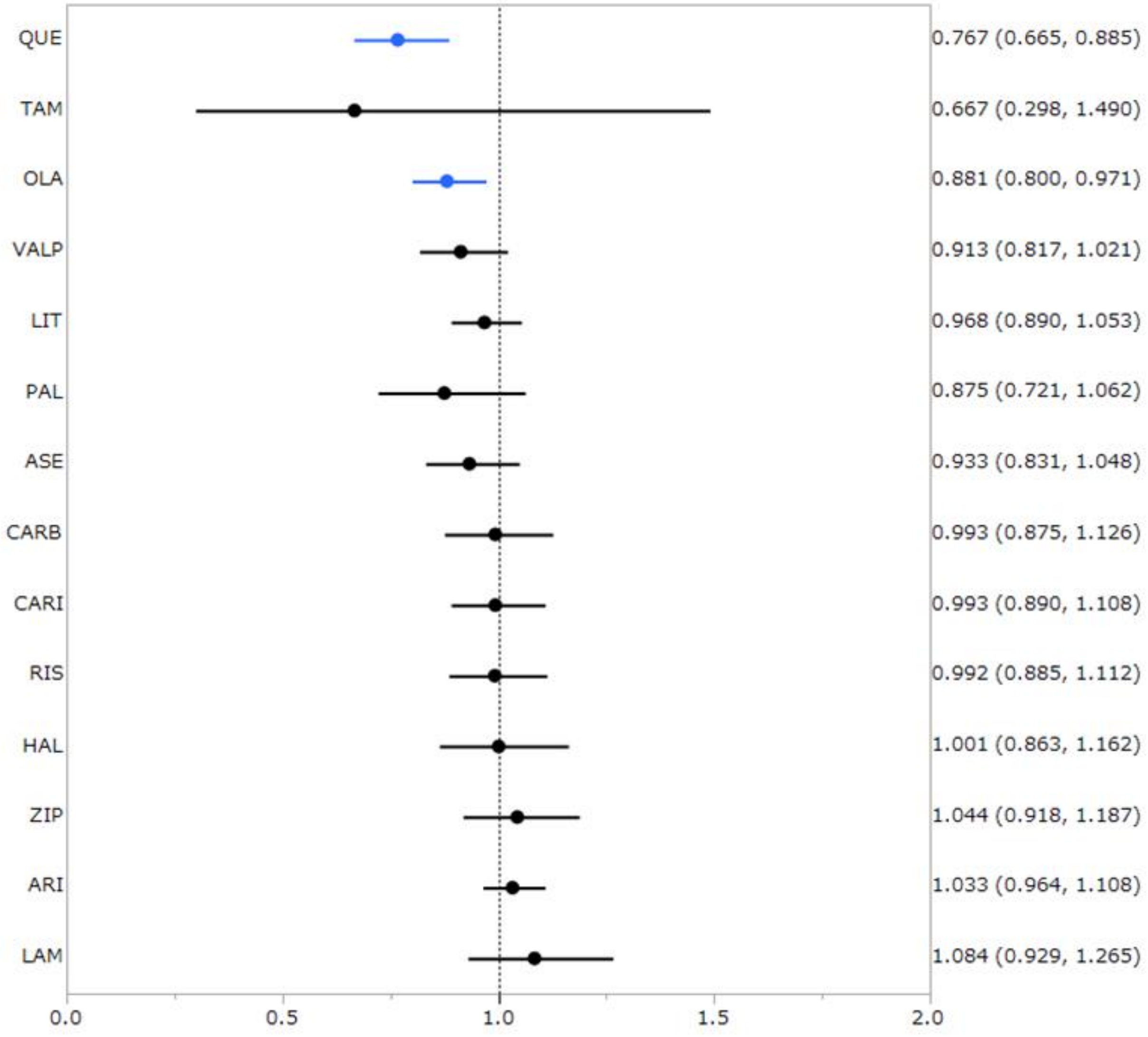

P-score

QUE	0.887
TAM	0.834
OLA	0.796
VALP	0.747
LIT	0.688
PAL	0.667
ASE	0.611
CARB	0.410
CARI	0.369
RIS	0.359
HAL	0.269
ZIP	0.234
ARI	0.195
LAM	0.042

Funnel plot (only double-blind, placebo-controlled trials)

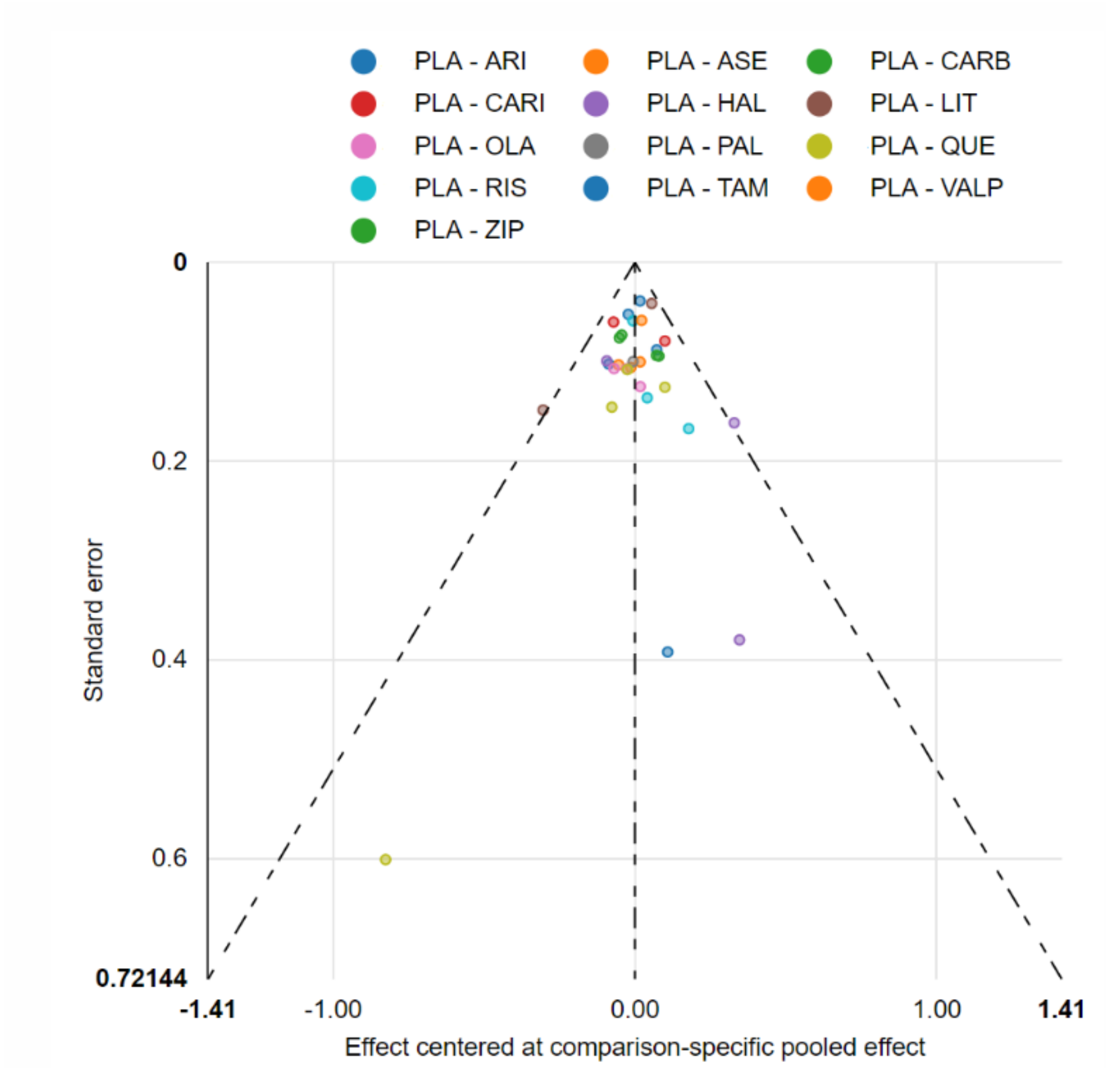

CINeMA confidence rating

Comparison	Number of studies	Within-study bias	Reporting bias	Indirectness	Imprecision	Heterogeneity	Incoherence	Confidence rating
ARI vs HAL	1	Some concerns	Suspected	No concerns	Major concerns	No concerns	Major concerns	Very low
ARI vs LIT	1	Some concerns	Suspected	No concerns	Major concerns	No concerns	No concerns	Very low
ARI vs PLA	4	Some concerns	Suspected	No concerns	Major concerns	No concerns	No concerns	Very low
ASE vs OLA	2	Some concerns	Suspected	No concerns	Major concerns	No concerns	No concerns	Very low
ASE vs PLA	3	Some concerns	Suspected	No concerns	Major concerns	No concerns	No concerns	Very low
CARB vs PLA	2	Some concerns	Suspected	No concerns	Major concerns	No concerns	No concerns	Very low
CARI vs PLA	2	Some concerns	Suspected	No concerns	Major concerns	No concerns	No concerns	Very low
HAL vs QUE	1	Some concerns	Suspected	No concerns	No concerns	No concerns	No concerns	Low
HAL vs RIS	1	Some concerns	Suspected	No concerns	Major concerns	No concerns	No concerns	Very low
HAL vs PLA	3	Some concerns	Suspected	No concerns	Major concerns	No concerns	No concerns	Very low
LAM vs LIT	1	Some concerns	Suspected	No concerns	Major concerns	No concerns	No concerns	Very low
LIT vs OLA	1	Some concerns	Suspected	No concerns	Major concerns	No concerns	No concerns	Very low
LIT vs QUE	2	No concerns	Suspected	No concerns	No concerns	No concerns	No concerns	Moderate
LIT vs VALP	1	No concerns	Suspected	No concerns	Major concerns	No concerns	No concerns	Low
LIT vs PLA	2	Some concerns	Suspected	No concerns	Major concerns	No concerns	No concerns	Very low
OLA vs VALP	1	No concerns	Suspected	No concerns	Major concerns	No concerns	No concerns	Low
OLA vs PLA	3	Some concerns	Suspected	No concerns	No concerns	Major concerns	No concerns	Very low
PAL vs QUE	1	No concerns	Suspected	No concerns	Major concerns	No concerns	No concerns	Low
PAL vs PLA	1	No concerns	Suspected	No concerns	Major concerns	No concerns	No concerns	Low
QUE vs PLA	4	No concerns	Suspected	No concerns	No concerns	No concerns	Major concerns	Low
RIS vs PLA	3	Some concerns	Suspected	No concerns	Major concerns	No concerns	Major concerns	Very low
TAM vs PLA	1	No concerns	Suspected	No concerns	Major concerns	No concerns	No concerns	Low
VALP vs PLA	1	No concerns	Suspected	No concerns	Major concerns	No concerns	No concerns	Low

ZIP vs PLA	2	Some concerns	Suspected	No concerns	Major concerns	No concerns	No concerns	Very low
ARI vs ASE	0	Some concerns	Suspected	No concerns	Major concerns	No concerns	No concerns	Very low
ARI vs CARB	0	Some concerns	Suspected	No concerns	Major concerns	No concerns	No concerns	Very low
ARI vs CARI	0	Some concerns	Suspected	No concerns	Major concerns	No concerns	No concerns	Very low
ARI vs LAM	0	Some concerns	Suspected	No concerns	Major concerns	No concerns	No concerns	Very low
ARI vs OLA	0	Some concerns	Suspected	No concerns	No concerns	No concerns	No concerns	Very low
ARI vs PAL	0	No concerns	Suspected	No concerns	Major concerns	No concerns	No concerns	Very low
ARI vs QUE	0	Some concerns	Suspected	No concerns	No concerns	No concerns	No concerns	Very low
ARI vs RIS	0	Some concerns	Suspected	No concerns	Major concerns	No concerns	No concerns	Very low
ARI vs TAM	0	Some concerns	Suspected	No concerns	Major concerns	No concerns	No concerns	Very low
ARI vs VALP	0	Some concerns	Suspected	No concerns	Major concerns	No concerns	No concerns	Very low
ARI vs ZIP	0	Some concerns	Suspected	No concerns	Major concerns	No concerns	No concerns	Very low
ASE vs CARB	0	Some concerns	Suspected	No concerns	Major concerns	No concerns	No concerns	Very low
ASE vs CARI	0	Some concerns	Suspected	No concerns	Major concerns	No concerns	No concerns	Very low
ASE vs HAL	0	Some concerns	Suspected	No concerns	Major concerns	No concerns	No concerns	Very low
ASE vs LAM	0	Some concerns	Suspected	No concerns	Major concerns	No concerns	No concerns	Very low
ASE vs LIT	0	Some concerns	Suspected	No concerns	Major concerns	No concerns	No concerns	Very low
ASE vs PAL	0	No concerns	Suspected	No concerns	Major concerns	No concerns	No concerns	Very low
ASE vs QUE	0	No concerns	Suspected	No concerns	No concerns	Major concerns	No concerns	Very low
ASE vs RIS	0	Some concerns	Suspected	No concerns	Major concerns	No concerns	No concerns	Very low
ASE vs TAM	0	No concerns	Suspected	No concerns	Major concerns	No concerns	No concerns	Very low
ASE vs VALP	0	No concerns	Suspected	No concerns	Major concerns	No concerns	No concerns	Very low
ASE vs ZIP	0	Some concerns	Suspected	No concerns	Major concerns	No concerns	No concerns	Very low
CARB vs CARI	0	Some concerns	Suspected	No concerns	Major concerns	No concerns	No concerns	Very low
CARB vs HAL	0	Some concerns	Suspected	No concerns	Major concerns	No concerns	No concerns	Very low

CARB vs LAM	0	Some concerns	Suspected	No concerns	Major concerns	No concerns	No concerns	Very low
CARB vs LIT	0	Some concerns	Suspected	No concerns	Major concerns	No concerns	No concerns	Very low
CARB vs OLA	0	Some concerns	Suspected	No concerns	Major concerns	No concerns	No concerns	Very low
CARB vs PAL	0	No concerns	Suspected	No concerns	Major concerns	No concerns	No concerns	Very low
CARB vs QUE	0	Some concerns	Suspected	No concerns	No concerns	No concerns	No concerns	Very low
CARB vs RIS	0	Some concerns	Suspected	No concerns	Major concerns	No concerns	No concerns	Very low
CARB vs TAM	0	Some concerns	Suspected	No concerns	Major concerns	No concerns	No concerns	Very low
CARB vs VALP	0	Some concerns	Suspected	No concerns	Major concerns	No concerns	No concerns	Very low
CARB vs ZIP	0	Some concerns	Suspected	No concerns	Major concerns	No concerns	No concerns	Very low
CARI vs HAL	0	Some concerns	Suspected	No concerns	Major concerns	No concerns	No concerns	Very low
CARI vs LAM	0	Some concerns	Suspected	No concerns	Major concerns	No concerns	No concerns	Very low
CARI vs LIT	0	Some concerns	Suspected	No concerns	Major concerns	No concerns	No concerns	Very low
CARI vs OLA	0	Some concerns	Suspected	No concerns	Major concerns	No concerns	No concerns	Very low
CARI vs PAL	0	No concerns	Suspected	No concerns	Major concerns	No concerns	No concerns	Very low
CARI vs QUE	0	Some concerns	Suspected	No concerns	No concerns	No concerns	No concerns	Very low
CARI vs RIS	0	Some concerns	Suspected	No concerns	Major concerns	No concerns	No concerns	Very low
CARI vs TAM	0	Some concerns	Suspected	No concerns	Major concerns	No concerns	No concerns	Very low
CARI vs VALP	0	Some concerns	Suspected	No concerns	Major concerns	No concerns	No concerns	Very low
CARI vs ZIP	0	Some concerns	Suspected	No concerns	Major concerns	No concerns	No concerns	Very low
HAL vs LAM	0	Some concerns	Suspected	No concerns	Major concerns	No concerns	No concerns	Very low
HAL vs LIT	0	Some concerns	Suspected	No concerns	Major concerns	No concerns	No concerns	Very low
HAL vs OLA	0	Some concerns	Suspected	No concerns	Major concerns	No concerns	No concerns	Very low
HAL vs PAL	0	Some concerns	Suspected	No concerns	Major concerns	No concerns	No concerns	Very low
HAL vs TAM	0	Some concerns	Suspected	No concerns	Major concerns	No concerns	No concerns	Very low
HAL vs VALP	0	Some concerns	Suspected	No concerns	Major concerns	No concerns	No concerns	Very low

HAL vs ZIP	0	Some concerns	Suspected	No concerns	Major concerns	No concerns	No concerns	Very low
LAM vs OLA	0	Some concerns	Suspected	No concerns	No concerns	No concerns	No concerns	Very low
LAM vs PAL	0	Some concerns	Suspected	No concerns	Major concerns	No concerns	No concerns	Very low
LAM vs QUE	0	Some concerns	Suspected	No concerns	No concerns	No concerns	No concerns	Very low
LAM vs RIS	0	Some concerns	Suspected	No concerns	Major concerns	No concerns	No concerns	Very low
LAM vs TAM	0	Some concerns	Suspected	No concerns	Major concerns	No concerns	No concerns	Very low
LAM vs VALP	0	Some concerns	Suspected	No concerns	Major concerns	No concerns	No concerns	Very low
LAM vs ZIP	0	Some concerns	Suspected	No concerns	Major concerns	No concerns	No concerns	Very low
LAM vs PLA	0	Some concerns	Suspected	No concerns	Major concerns	No concerns	No concerns	Very low
LIT vs PAL	0	No concerns	Suspected	No concerns	Major concerns	No concerns	No concerns	Very low
LIT vs RIS	0	Some concerns	Suspected	No concerns	Major concerns	No concerns	No concerns	Very low
LIT vs TAM	0	No concerns	Suspected	No concerns	Major concerns	No concerns	No concerns	Very low
LIT vs ZIP	0	Some concerns	Suspected	No concerns	Major concerns	No concerns	No concerns	Very low
OLA vs PAL	0	No concerns	Suspected	No concerns	Major concerns	No concerns	No concerns	Very low
OLA vs QUE	0	No concerns	Suspected	No concerns	Major concerns	No concerns	No concerns	Very low
OLA vs RIS	0	Some concerns	Suspected	No concerns	Major concerns	No concerns	No concerns	Very low
OLA vs TAM	0	No concerns	Suspected	No concerns	Major concerns	No concerns	No concerns	Very low
OLA vs ZIP	0	Some concerns	Suspected	No concerns	No concerns	Major concerns	No concerns	Very low
PAL vs RIS	0	No concerns	Suspected	No concerns	Major concerns	No concerns	No concerns	Very low
PAL vs TAM	0	No concerns	Suspected	No concerns	Major concerns	No concerns	No concerns	Very low
PAL vs VALP	0	No concerns	Suspected	No concerns	Major concerns	No concerns	No concerns	Very low
PAL vs ZIP	0	No concerns	Suspected	No concerns	Major concerns	No concerns	No concerns	Very low
QUE vs RIS	0	Some concerns	Suspected	No concerns	No concerns	No concerns	No concerns	Very low
QUE vs TAM	0	No concerns	Suspected	No concerns	Major concerns	No concerns	No concerns	Very low
QUE vs VALP	0	No concerns	Suspected	No concerns	Major concerns	No concerns	No concerns	Very low

QUE vs ZIP	0	No concerns	Suspected	No concerns	No concerns	No concerns	No concerns	Low
RIS vs TAM	0	Some concerns	Suspected	No concerns	Major concerns	No concerns	No concerns	Very low
RIS vs VALP	0	Some concerns	Suspected	No concerns	Major concerns	No concerns	No concerns	Very low
RIS vs ZIP	0	Some concerns	Suspected	No concerns	Major concerns	No concerns	No concerns	Very low
TAM vs VALP	0	No concerns	Suspected	No concerns	Major concerns	No concerns	No concerns	Very low
TAM vs ZIP	0	No concerns	Suspected	No concerns	Major concerns	No concerns	No concerns	Very low
VALP vs ZIP	0	No concerns	Suspected	No concerns	Major concerns	No concerns	No concerns	Very low

Supplementary Appendix 11. Use of anticholinergic agent (N = 20, n = 6256)

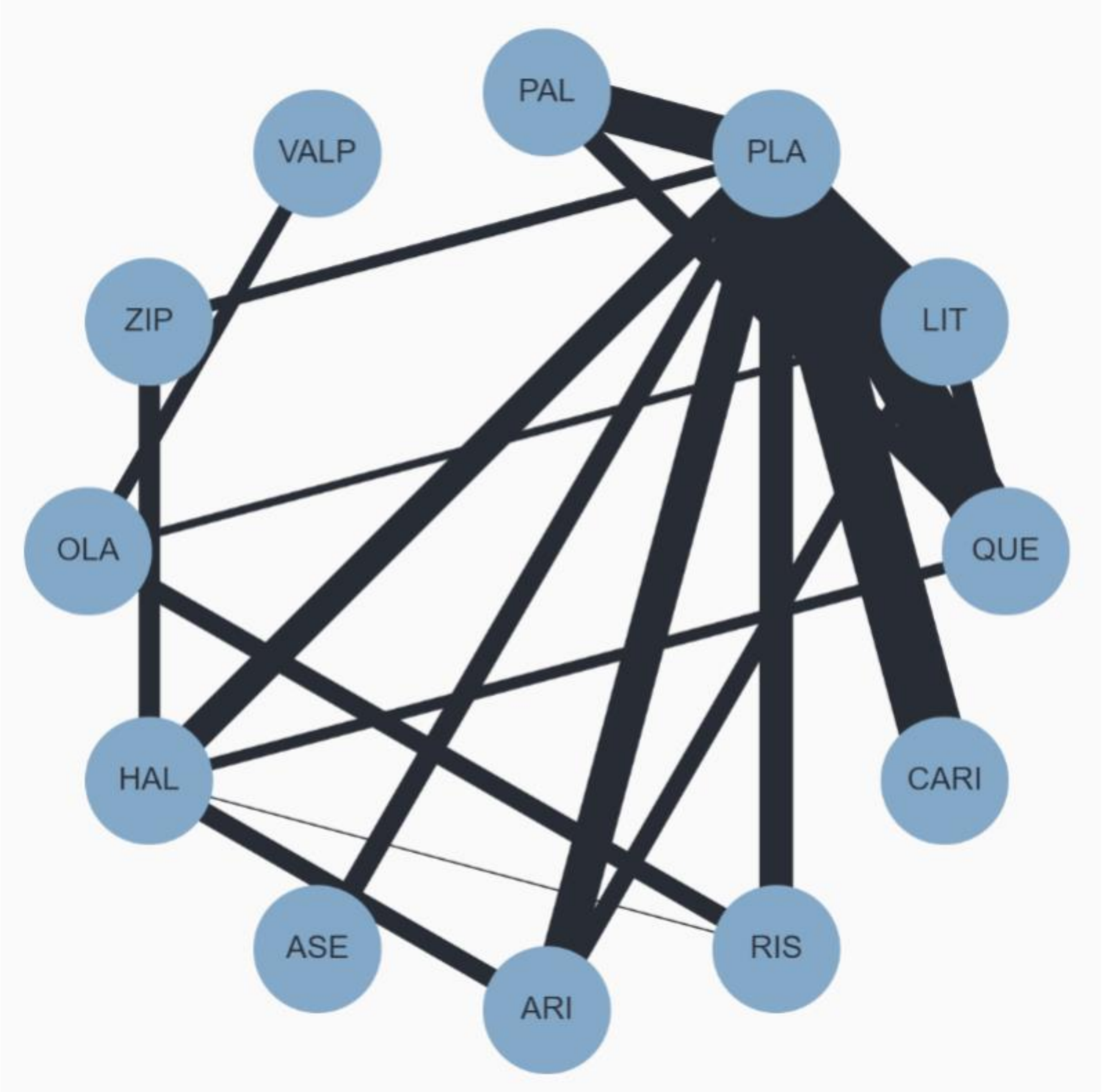

League table (risk ratio with 95% confidence interval)

ARI	0.863 (0.215, 3.453)	0.988 (0.519, 1.881)	**0.406 (0.246, 0.670)**	**2.134 (1.176, 3.873)**	1.362 (0.558, 3.323)	1.076 (0.529, 2.190)	**2.448 (1.337, 4.480)**	0.839 (0.422, 1.669)	1.830 (0.535, 6.256)	0.647 (0.334, 1.254)	**2.556 (1.553, 4.205)**
	ASE	1.145 (0.295, 4.450)	0.470 (0.121, 1.832)	2.474 (0.611, 10.018)	1.578 (0.350, 7.124)	1.248 (0.307, 5.073)	2.837 (0.719, 11.195)	0.973 (0.243, 3.905)	2.121 (0.377, 11.942)	0.750 (0.182, 3.090)	2.963 (0.812, 10.812)
		CARI	**0.411 (0.229, 0.736)**	**2.161 (1.107, 4.218)**	1.379 (0.576, 3.301)	1.090 (0.554, 2.145)	**2.478 (1.343, 4.572)**	0.850 (0.444, 1.627)	1.853 (0.549, 6.247)	0.655 (0.324, 1.325)	**2.588 (1.719, 3.895)**
			HAL	**5.260 (2.881, 9.605)**	**3.356 (1.438, 7.832)**	**2.653 (1.383, 5.089)**	**6.033 (3.563, 10.214)**	**2.069 (1.115, 3.841)**	**4.510 (1.362, 14.932)**	1.594 (0.999, 2.543)	**6.299 (4.159, 9.541)**
				LIT	0.638 (0.266, 1.532)	0.504 (0.249, 1.021)	1.147 (0.666, 1.974)	**0.393 (0.196, 0.791)**	0.857 (0.254, 2.898)	**0.303 (0.146, 0.630)**	1.197 (0.705, 2.033)
					OLA	0.791 (0.311, 2.009)	1.798 (0.752, 4.296)	0.617 (0.329, 1.156)	1.344 (0.577, 3.131)	0.475 (0.186, 1.216)	1.877 (0.868, 4.060)
						PAL	**2.274 (1.269, 4.073)**	0.780 (0.375, 1.622)	1.700 (0.483, 5.985)	0.601 (0.279, 1.293)	**2.374 (1.384, 4.072)**
							QUE	**0.343 (0.177, 0.665)**	0.748 (0.222, 2.517)	**0.264 (0.135, 0.518)**	1.044 (0.662, 1.647)
								RIS	2.180 (0.760, 6.251)	0.770 (0.368, 1.615)	**3.044 (1.838, 5.041)**
									VALP	0.353 (0.100, 1.252)	1.397 (0.445, 4.387)
										ZIP	**3.952 (2.226, 7.015)**
											PLA

Evaluation of heterogeneity and inconsistency

Between study variance (τ^2)	Heterogeneity assessment	Random-effects design-by-treatment interaction model		
		Q	df	p
0.056	Low to moderate	1.439	11	1.000

Incoherence

	NMA, RR (95% CI)	Direct, RR (95% CI)	I^2	Indirect, RR (95% CI)	Inconsistency measures	
					Difference of RR	P value
ARI vs HAL	0.406 (0.246, 0.670)	0.338 (0.183, 0.625)	na	0.585 (0.245, 1.395)	0.579 (0.200, 1.677)	0.314
ARI vs LIT	2.134 (1.176, 3.873)	2.581 (1.178, 5.654)	na	1.646 (0.658, 4.118)	1.568 (0.469, 5.240)	0.465
ARI vs PLA	2.556 (1.553, 4.205)	2.713 (1.506, 4.887)	0.0%	2.199 (0.864, 5.595)	1.234 (0.409, 3.721)	0.709
ASE vs PLA		2.963 (0.812, 10.812)	na			
CARI vs PLA		2.588 (1.719, 3.895)	77.4%			
HAL vs QUE	6.033 (3.563, 10.214)	5.358 (2.473, 11.606)	na	6.686 (3.257, 13.725)	0.801 (0.279, 2.303)	0.681
HAL vs RIS	2.069 (1.115, 3.841)	3.000 (0.666, 13.511)	na	1.919 (0.973, 3.782)	1.564 (0.300, 8.148)	0.596
HAL vs ZIP	1.594 (0.999, 2.543)	1.611 (0.969, 2.679)	na	1.504 (0.461, 4.901)	1.072 (0.296, 3.878)	0.916
HAL vs PLA	6.299 (4.159, 9.541)	5.398 (3.170, 9.191)	0.0%	8.009 (4.123, 15.555)	0.674 (0.288, 1.578)	0.363
LIT vs OLA	0.638 (0.266, 1.532)	0.648 (0.105, 3.993)	na	0.635 (0.234, 1.726)	1.020 (0.128, 8.130)	0.985
LIT vs QUE	1.147 (0.666, 1.974)	1.175 (0.598, 2.308)	0.0%	1.097 (0.440, 2.736)	1.071 (0.344, 3.337)	0.905
LIT vs PLA	1.197 (0.705, 2.033)	1.351 (0.690, 2.646)	0.0%	0.984 (0.417, 2.321)	1.374 (0.462, 4.087)	0.568
OLA vs RIS	0.617 (0.329, 1.156)	0.618 (0.318, 1.199)	na	0.606 (0.085, 4.328)	1.020 (0.128, 8.130)	0.985
OLA vs VALP		1.344 (0.577, 3.131)	na			
PAL vs QUE	2.274 (1.269, 4.073)	2.512 (1.167, 5.409)	na	1.983 (0.808, 4.867)	1.267 (0.389, 4.125)	0.695
PAL vs PLA	2.374 (1.384, 4.072)	2.348 (1.271, 4.338)	27.3%	2.464 (0.794, 7.651)	0.953 (0.263, 3.457)	0.942
QUE vs PLA	1.044 (0.662, 1.647)	1.087 (0.636, 1.859)	0.0%	0.939 (0.394, 2.236)	1.158 (0.418, 3.213)	0.778

RIS vs PLA	3.044 (1.838, 5.041)	3.205 (1.846, 5.564)	82.9%	2.344 (0.675, 8.139)	1.367 (0.350, 5.336)	0.653
ZIP vs PLA	3.952 (2.226, 7.015)	4.155 (1.389, 12.426)	na	3.878 (1.977, 7.606)	1.072 (0.296, 3.878)	0.916

Forest plot (vs placebo, the numbers are risk ratio with 95% confidence interval)

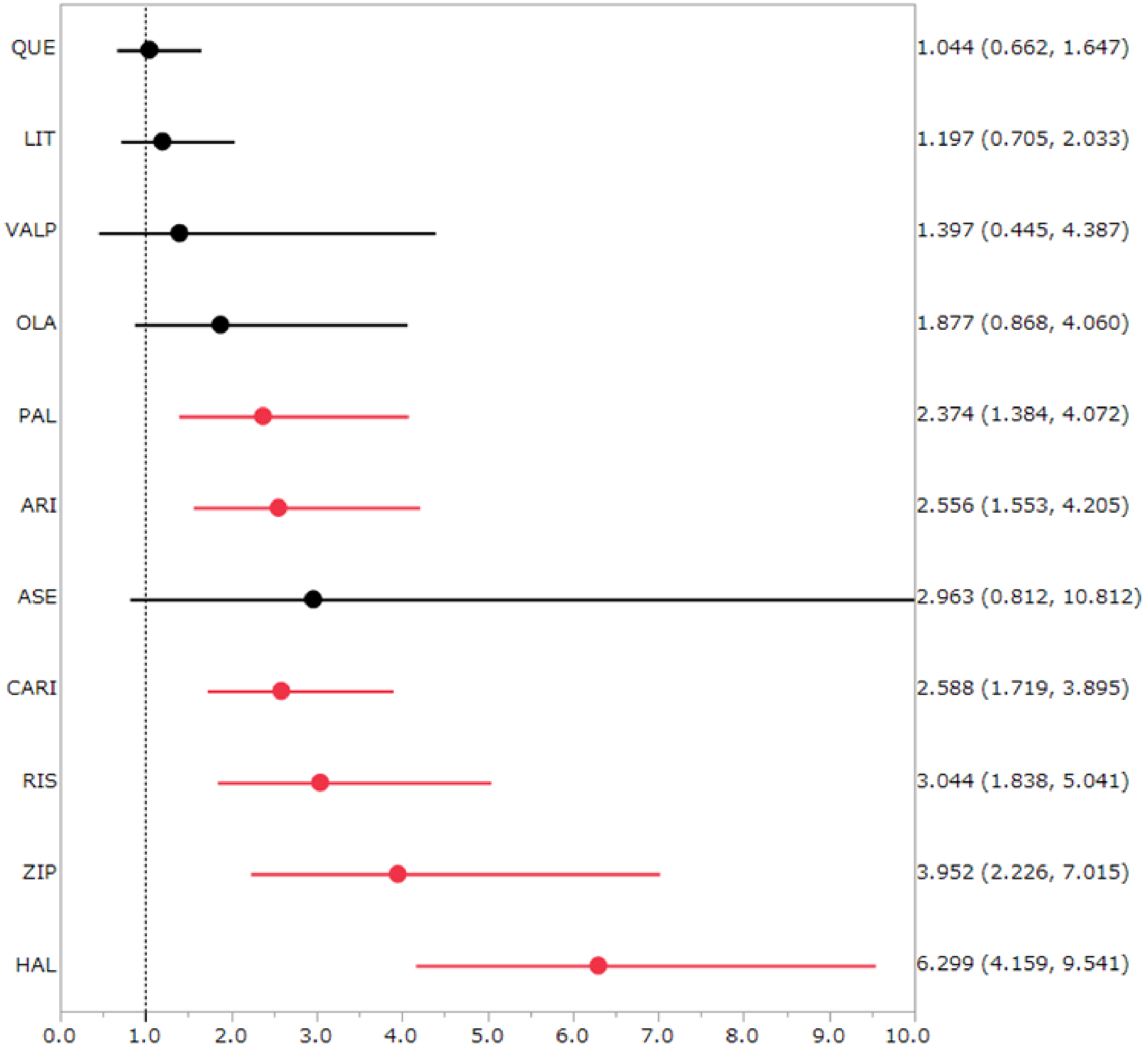

P-score

QUE	0.878
LIT	0.800
VALP	0.701
OLA	0.566
PAL	0.477
ARI	0.416
ASE	0.382
CARI	0.356
RIS	0.271
ZIP	0.238
HAL	0.009

Funnel plot (only double-blind, placebo-controlled trials)

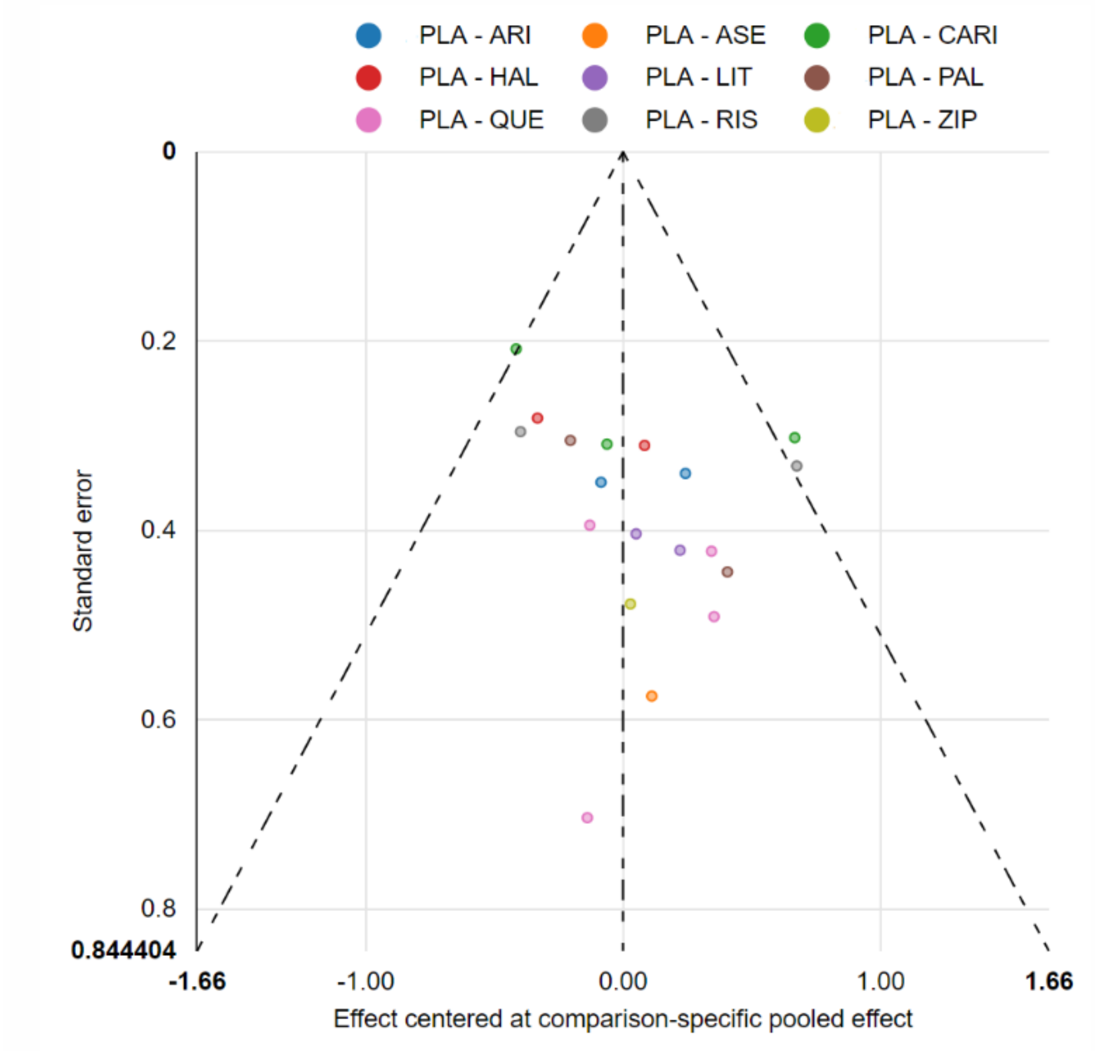

CINeMA confidence rating

Comparison	Number of studies	Within-study bias	Reporting bias	Indirectness	Imprecision	Heterogeneity	Incoherence	Confidence rating
ARI vs HAL	1	Some concerns	Suspected	No concerns	No concerns	No concerns	No concerns	Low
ARI vs LIT	1	Some concerns	Suspected	No concerns	No concerns	Major concerns	No concerns	Very low
ARI vs PLA	2	Some concerns	Suspected	No concerns	No concerns	No concerns	No concerns	Low
ASE vs PLA	1	No concerns	Suspected	No concerns	Major concerns	No concerns	No concerns	Low
CARI vs PLA	3	Some concerns	Suspected	No concerns	No concerns	No concerns	No concerns	Low
HAL vs QUE	1	Some concerns	Suspected	No concerns	No concerns	No concerns	No concerns	Low
HAL vs RIS	1	Some concerns	Suspected	No concerns	No concerns	Major concerns	No concerns	Very low
HAL vs ZIP	1	Some concerns	Suspected	No concerns	Major concerns	No concerns	No concerns	Very low
HAL vs PLA	2	Some concerns	Suspected	No concerns	No concerns	No concerns	No concerns	Low
LIT vs OLA	1	Some concerns	Suspected	No concerns	Major concerns	No concerns	No concerns	Very low
LIT vs QUE	2	No concerns	Suspected	No concerns	Major concerns	No concerns	No concerns	Low
LIT vs PLA	2	Some concerns	Suspected	No concerns	Major concerns	No concerns	No concerns	Very low
OLA vs RIS	1	Some concerns	Suspected	No concerns	Major concerns	No concerns	No concerns	Very low
OLA vs VALP	1	No concerns	Suspected	No concerns	Major concerns	No concerns	No concerns	Low
PAL vs QUE	1	No concerns	Suspected	No concerns	No concerns	Major concerns	No concerns	Low
PAL vs PLA	2	No concerns	Suspected	No concerns	No concerns	No concerns	No concerns	Low
QUE vs PLA	4	No concerns	Suspected	No concerns	Major concerns	No concerns	No concerns	Low
RIS vs PLA	2	Some concerns	Suspected	No concerns	No concerns	No concerns	No concerns	Low
ZIP vs PLA	1	Some concerns	Suspected	No concerns	No concerns	No concerns	No concerns	Low
ARI vs ASE	0	Some concerns	Suspected	No concerns	Major concerns	No concerns	No concerns	Very low
ARI vs CARI	0	Some concerns	Suspected	No concerns	Major concerns	No concerns	No concerns	Very low
ARI vs OLA	0	Some concerns	Suspected	No concerns	Major concerns	No concerns	No concerns	Very low
ARI vs PAL	0	Some concerns	Suspected	No concerns	Major concerns	No concerns	No concerns	Very low

ARI vs QUE	0	Some concerns	Suspected	No concerns	No concerns	No concerns	No concerns	Very low
ARI vs RIS	0	Some concerns	Suspected	No concerns	Major concerns	No concerns	No concerns	Very low
ARI vs VALP	0	Some concerns	Suspected	No concerns	Major concerns	No concerns	No concerns	Very low
ARI vs ZIP	0	Some concerns	Suspected	No concerns	Major concerns	No concerns	No concerns	Very low
ASE vs CARI	0	Some concerns	Suspected	No concerns	Major concerns	No concerns	No concerns	Very low
ASE vs HAL	0	Some concerns	Suspected	No concerns	Major concerns	No concerns	No concerns	Very low
ASE vs LIT	0	No concerns	Suspected	No concerns	Major concerns	No concerns	No concerns	Very low
ASE vs OLA	0	Some concerns	Suspected	No concerns	Major concerns	No concerns	No concerns	Very low
ASE vs PAL	0	No concerns	Suspected	No concerns	Major concerns	No concerns	No concerns	Very low
ASE vs QUE	0	No concerns	Suspected	No concerns	Major concerns	No concerns	No concerns	Very low
ASE vs RIS	0	Some concerns	Suspected	No concerns	Major concerns	No concerns	No concerns	Very low
ASE vs VALP	0	No concerns	Suspected	No concerns	Major concerns	No concerns	No concerns	Very low
ASE vs ZIP	0	No concerns	Suspected	No concerns	Major concerns	No concerns	No concerns	Very low
CARI vs HAL	0	Some concerns	Suspected	No concerns	No concerns	No concerns	No concerns	Very low
CARI vs LIT	0	Some concerns	Suspected	No concerns	No concerns	Major concerns	No concerns	Very low
CARI vs OLA	0	Some concerns	Suspected	No concerns	Major concerns	No concerns	No concerns	Very low
CARI vs PAL	0	No concerns	Suspected	No concerns	Major concerns	No concerns	No concerns	Very low
CARI vs QUE	0	Some concerns	Suspected	No concerns	No concerns	No concerns	No concerns	Very low
CARI vs RIS	0	Some concerns	Suspected	No concerns	Major concerns	No concerns	No concerns	Very low
CARI vs VALP	0	Some concerns	Suspected	No concerns	Major concerns	No concerns	No concerns	Very low
CARI vs ZIP	0	Some concerns	Suspected	No concerns	Major concerns	No concerns	No concerns	Very low
HAL vs LIT	0	Some concerns	Suspected	No concerns	No concerns	No concerns	No concerns	Very low
HAL vs OLA	0	Some concerns	Suspected	No concerns	No concerns	No concerns	No concerns	Very low
HAL vs PAL	0	Some concerns	Suspected	No concerns	No concerns	No concerns	No concerns	Very low
HAL vs VALP	0	Some concerns	Suspected	No concerns	No concerns	No concerns	No concerns	Very low

LIT vs PAL	0	No concerns	Suspected	No concerns	Major concerns	No concerns	No concerns	Very low
LIT vs RIS	0	Some concerns	Suspected	No concerns	No concerns	No concerns	No concerns	Very low
LIT vs VALP	0	No concerns	Suspected	No concerns	Major concerns	No concerns	No concerns	Very low
LIT vs ZIP	0	Some concerns	Suspected	No concerns	No concerns	No concerns	No concerns	Very low
OLA vs PAL	0	Some concerns	Suspected	No concerns	Major concerns	No concerns	No concerns	Very low
OLA vs QUE	0	Some concerns	Suspected	No concerns	Major concerns	No concerns	No concerns	Very low
OLA vs ZIP	0	Some concerns	Suspected	No concerns	Major concerns	No concerns	No concerns	Very low
OLA vs PLA	0	Some concerns	Suspected	No concerns	Major concerns	No concerns	No concerns	Very low
PAL vs RIS	0	No concerns	Suspected	No concerns	Major concerns	No concerns	No concerns	Very low
PAL vs VALP	0	No concerns	Suspected	No concerns	Major concerns	No concerns	No concerns	Very low
PAL vs ZIP	0	Some concerns	Suspected	No concerns	Major concerns	No concerns	No concerns	Very low
QUE vs RIS	0	Some concerns	Suspected	No concerns	No concerns	No concerns	No concerns	Very low
QUE vs VALP	0	No concerns	Suspected	No concerns	Major concerns	No concerns	No concerns	Very low
QUE vs ZIP	0	Some concerns	Suspected	No concerns	No concerns	No concerns	No concerns	Very low
RIS vs VALP	0	No concerns	Suspected	No concerns	Major concerns	No concerns	No concerns	Very low
RIS vs ZIP	0	Some concerns	Suspected	No concerns	Major concerns	No concerns	No concerns	Very low
VALP vs ZIP	0	Some concerns	Suspected	No concerns	Major concerns	No concerns	No concerns	Very low
VALP vs PLA	0	Some concerns	Suspected	No concerns	Major concerns	No concerns	No concerns	Very low

Supplementary Appendix 12. Akathisia (N = 25, n = 8711)

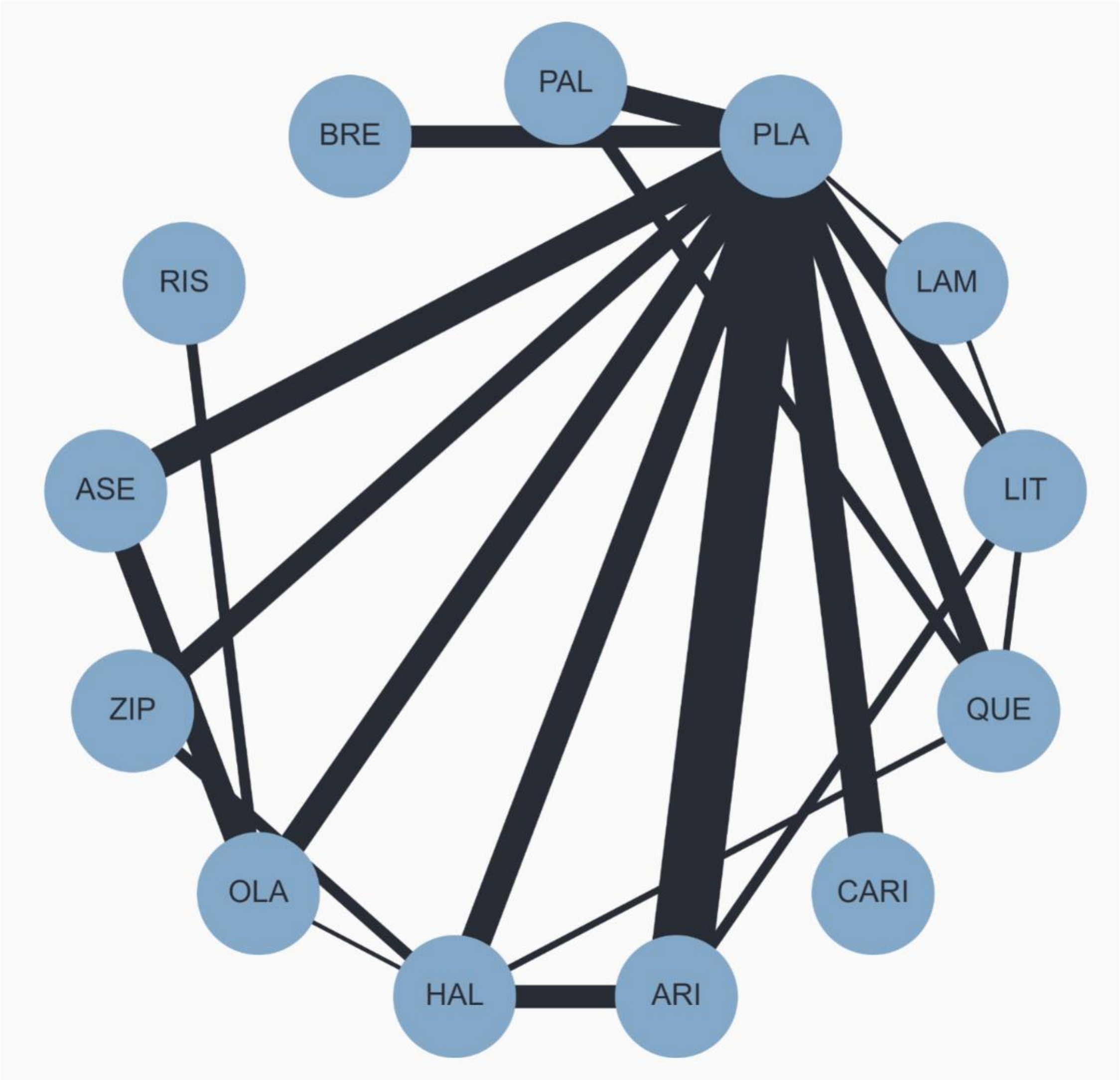

League table (risk ratio with 95% confidence interval)

ARI	1.547 (0.642, 3.727)	0.841 (0.324, 2.180)	0.706 (0.400, 1.247)	**0.548 (0.398, 0.756)**	1.594 (0.339, 7.486)	**2.857 (1.456, 5.607)**	1.408 (0.613, 3.230)	1.183 (0.519, 2.695)	**3.519 (1.751, 7.071)**	1.070 (0.364, 3.148)	0.724 (0.448, 1.172)	**3.058 (2.196, 4.259)**
	ASE	0.544 (0.161, 1.838)	0.456 (0.177, 1.178)	**0.354 (0.148, 0.849)**	1.031 (0.183, 5.803)	1.847 (0.634, 5.379)	0.910 (0.482, 1.720)	0.765 (0.247, 2.370)	2.276 (0.793, 6.531)	0.692 (0.271, 1.767)	0.468 (0.185, 1.188)	1.978 (0.864, 4.525)
		BRE	0.840 (0.307, 2.297)	0.652 (0.250, 1.699)	1.896 (0.326, 11.038)	**3.399 (1.102, 10.481)**	1.674 (0.511, 5.487)	1.407 (0.430, 4.601)	**4.186 (1.370, 12.792)**	1.273 (0.323, 5.020)	0.862 (0.316, 2.352)	**3.639 (1.489, 8.894)**
			CARI	0.777 (0.437, 1.382)	2.259 (0.462, 11.039)	**4.048 (1.771, 9.254)**	1.994 (0.805, 4.943)	1.675 (0.678, 4.143)	**4.986 (2.209, 11.256)**	1.516 (0.485, 4.737)	1.026 (0.535, 1.968)	**4.334 (2.728, 6.884)**
				HAL	2.908 (0.616, 13.727)	**5.211 (2.545, 10.672)**	**2.567 (1.131, 5.829)**	2.157 (0.960, 4.849)	**6.419 (3.316, 12.424)**	1.952 (0.669, 5.695)	1.321 (0.885, 1.972)	**5.579 (3.959, 7.862)**
					LAM	1.792 (0.355, 9.042)	0.883 (0.160, 4.861)	0.742 (0.135, 4.076)	2.207 (0.422, 11.556)	0.671 (0.107, 4.224)	0.454 (0.093, 2.211)	1.918 (0.421, 8.750)
						LIT	0.493 (0.176, 1.381)	0.414 (0.149, 1.147)	1.232 (0.490, 3.094)	0.374 (0.108, 1.293)	**0.254 (0.115, 0.560)**	1.070 (0.540, 2.124)
							OLA	0.840 (0.281, 2.512)	2.500 (0.907, 6.891)	0.760 (0.382, 1.514)	0.515 (0.213, 1.245)	2.173 (0.995, 4.743)
								PAL	**2.976 (1.377, 6.431)**	0.905 (0.248, 3.299)	0.613 (0.255, 1.471)	**2.586 (1.188, 5.631)**
									QUE	0.304 (0.089, 1.036)	**0.206 (0.097, 0.437)**	0.869 (0.445, 1.698)
										RIS	0.677 (0.221, 2.076)	**2.859 (1.009, 8.099)**
											ZIP	**4.222 (2.672, 6.672)**

												PLA

Evaluation of heterogeneity and inconsistency

Between study variance (τ^2)	Heterogeneity assessment	Random-effects design-by-treatment interaction model		
		Q	df	p
0.000	Low	19.221	14	0.157

Incoherence

	NMA, RR (95% CI)	Direct, RR (95% CI)	I^2	Indirect, RR (95% CI)	Inconsistency measures	
					Difference of RR	P value
ARI vs HAL	0.548 (0.398, 0.756)	0.470 (0.323, 0.683)	0.0%	0.851 (0.453, 1.598)	0.552 (0.265, 1.149)	0.112
ARI vs LIT	2.857 (1.456, 5.607)	2.194 (0.975, 4.935)	na	5.173 (1.534, 17.442)	0.424 (0.098, 1.828)	0.250
ARI vs PLA	3.058 (2.196, 4.259)	3.572 (2.429, 5.252)	0.0%	1.981 (1.038, 3.777)	1.803 (0.850, 3.826)	0.124
ASE vs OLA	0.910 (0.482, 1.720)	0.765 (0.396, 1.480)	44.1%	9.675 (0.847, 110.465)	0.079 (0.006, 0.986)	0.049
ASE vs PLA	1.978 (0.864, 4.525)	2.396 (0.981, 5.849)	37.9%	0.607 (0.066, 5.559)	3.947 (0.363, 42.983)	0.260
BRE vs PLA		3.639 (1.489, 8.894)	0.0%			
CARI vs PLA		4.334 (2.728, 6.884)	0.0%			
HAL vs OLA	2.567 (1.131, 5.829)	7.205 (1.525, 34.032)	na	1.723 (0.656, 4.524)	4.183 (0.672, 26.031)	0.125
HAL vs QUE	6.419 (3.316, 12.424)	5.667 (2.484, 12.927)	na	8.020 (2.663, 24.146)	0.707 (0.178, 2.799)	0.621
HAL vs ZIP	1.321 (0.885, 1.972)	1.041 (0.663, 1.635)	na	3.185 (1.339, 7.581)	0.327 (0.123, 0.868)	0.025
HAL vs PLA	5.579 (3.959, 7.862)	5.347 (3.236, 8.834)	0.0%	5.790 (3.620, 9.261)	0.923 (0.464, 1.836)	0.820
LAM vs LIT	1.792 (0.355, 9.042)	7.376 (0.388, 140.387)	na	0.972 (0.140, 6.745)	7.586 (0.223, 257.804)	0.260
LAM vs PLA	1.918 (0.421, 8.750)	1.456 (0.297, 7.153)	na	30.317 (0.197, 4667.599)	0.048 (0.000, 9.455)	0.260
LIT vs QUE	1.232 (0.490, 3.094)	3.276 (0.346, 30.969)	na	1.011 (0.368, 2.775)	3.240 (0.276, 38.042)	0.349
LIT vs PLA	1.070 (0.540, 2.124)	0.908 (0.400, 2.064)	30.8%	1.562 (0.450, 5.428)	0.581 (0.131, 2.583)	0.476
OLA vs PLA	2.173 (0.995, 4.743)	2.252 (0.898, 5.647)	0.0%	1.980 (0.451, 8.691)	1.137 (0.199, 6.488)	0.885
PAL vs QUE	2.976 (1.377, 6.431)	2.639 (1.055, 6.602)	na	3.973 (0.958, 16.476)	0.664 (0.122, 3.609)	0.636

PAL vs PLA	2.586 (1.188, 5.631)	2.928 (1.156, 7.415)	0.0%	1.933 (0.466, 8.022)	1.514 (0.277, 8.285)	0.632
QUE vs PLA	0.869 (0.445, 1.698)	0.783 (0.355, 1.730)	27.7%	1.128 (0.322, 3.956)	0.695 (0.157, 3.063)	0.630
ZIP vs PLA	4.222 (2.672, 6.672)	2.668 (1.380, 5.159)	0.0%	6.466 (3.425, 12.207)	0.413 (0.165, 1.031)	0.058

Forest plot (vs placebo, the numbers are risk ratio with 95% confidence interval)

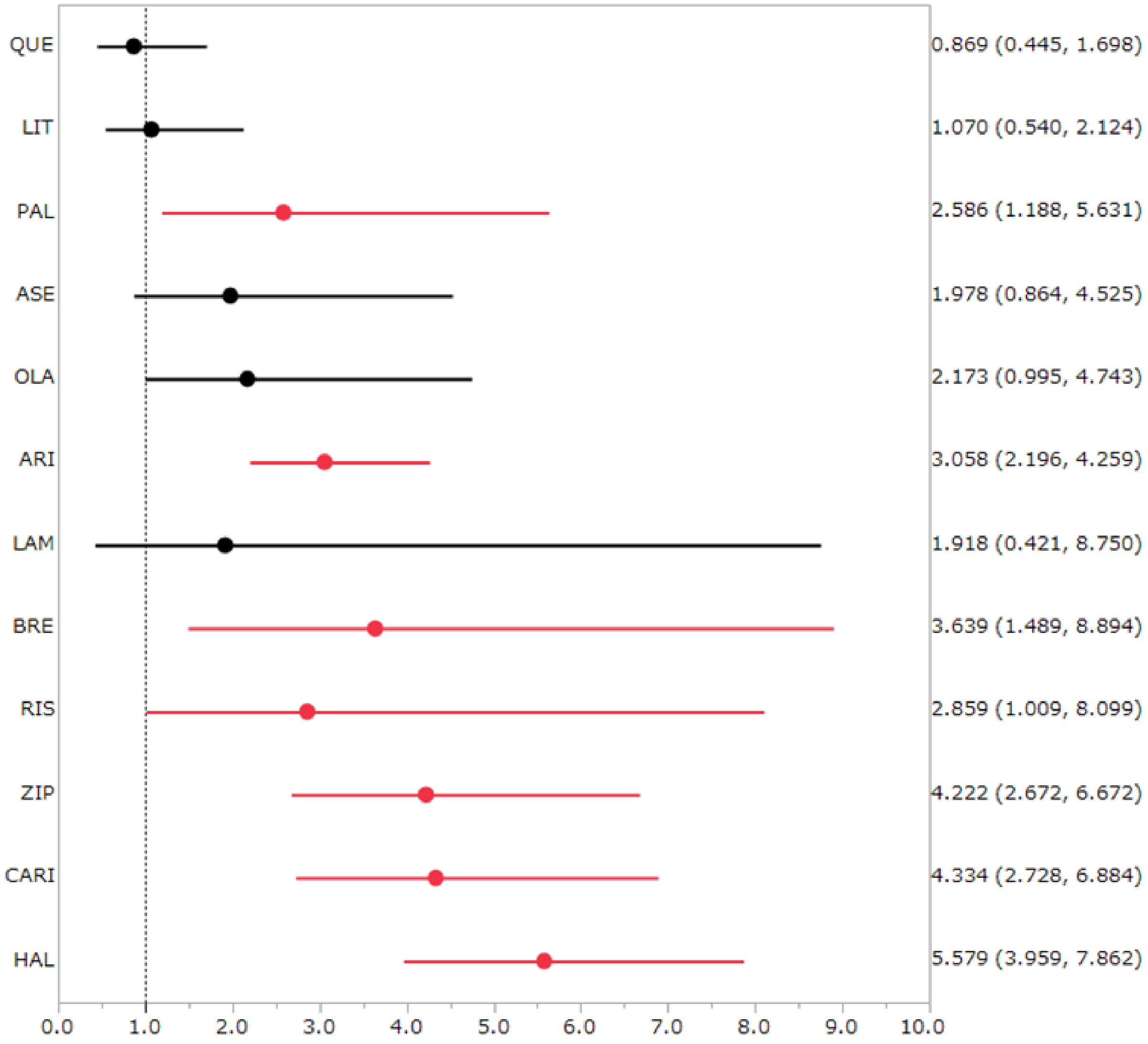

P-score

QUE	0.953
LIT	0.866
PAL	0.567
ASE	0.520
OLA	0.481
ARI	0.445
LAM	0.432
BRE	0.389
RIS	0.328
ZIP	0.302
CARI	0.244
HAL	0.084

Funnel plot (only double-blind, placebo-controlled trials)

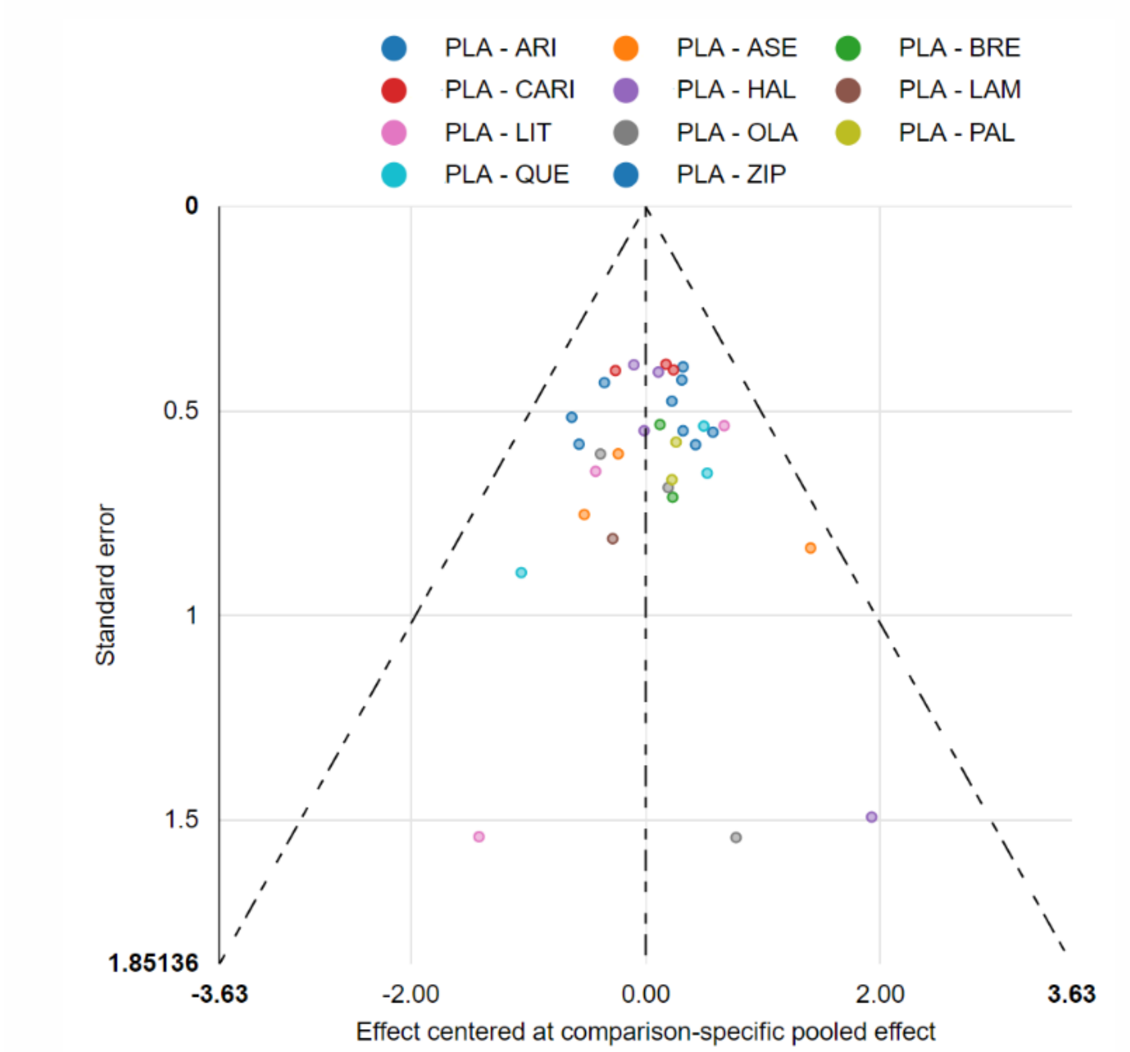

CINeMA confidence rating

Comparison	Number of studies	Within-study bias	Reporting bias	Indirectness	Imprecision	Heterogeneity	Incoherence	Confidence rating
ARI vs HAL	2	Some concerns	Suspected	No concerns	No concerns	No concerns	No concerns	Low
ARI vs LIT	1	Some concerns	Suspected	No concerns	No concerns	No concerns	No concerns	Low
ARI vs PLA	6	Some concerns	Suspected	No concerns	No concerns	No concerns	No concerns	Low
ASE vs OLA	2	Some concerns	Suspected	No concerns	Major concerns	No concerns	No concerns	Very low
ASE vs PLA	3	Some concerns	Suspected	No concerns	Major concerns	No concerns	No concerns	Very low
BRE vs PLA	2	Some concerns	Suspected	No concerns	No concerns	No concerns	No concerns	Low
CARI vs PLA	3	Some concerns	Suspected	No concerns	No concerns	No concerns	No concerns	Low
HAL vs OLA	1	Some concerns	Suspected	No concerns	No concerns	No concerns	No concerns	Low
HAL vs QUE	1	Some concerns	Suspected	No concerns	No concerns	No concerns	No concerns	Low
HAL vs ZIP	1	Some concerns	Suspected	No concerns	Major concerns	No concerns	Major concerns	Very low
HAL vs PLA	4	Some concerns	Suspected	No concerns	No concerns	No concerns	No concerns	Low
LAM vs LIT	1	Some concerns	Suspected	No concerns	Major concerns	No concerns	No concerns	Very low
LAM vs PLA	1	Some concerns	Suspected	No concerns	Major concerns	No concerns	No concerns	Very low
LIT vs QUE	1	Some concerns	Suspected	No concerns	Major concerns	No concerns	No concerns	Very low
LIT vs PLA	3	Some concerns	Suspected	No concerns	Major concerns	No concerns	No concerns	Very low
OLA vs RIS	1	Some concerns	Suspected	No concerns	Major concerns	No concerns	No concerns	Very low
OLA vs PLA	3	Some concerns	Suspected	No concerns	Major concerns	No concerns	No concerns	Very low
PAL vs QUE	1	No concerns	Suspected	No concerns	No concerns	No concerns	No concerns	Moderate
PAL vs PLA	2	No concerns	Suspected	No concerns	No concerns	No concerns	No concerns	Moderate
QUE vs PLA	3	Some concerns	Suspected	No concerns	Major concerns	No concerns	No concerns	Very low
ZIP vs PLA	3	Some concerns	Suspected	No concerns	No concerns	No concerns	No concerns	Low
ARI vs ASE	0	Some concerns	Suspected	No concerns	Major concerns	No concerns	No concerns	Very low
ARI vs BRE	0	Some concerns	Suspected	No concerns	Major concerns	No concerns	No concerns	Very low

ARI vs CARI	0	Some concerns	Suspected	No concerns	Major concerns	No concerns	No concerns	Very low
ARI vs LAM	0	Some concerns	Suspected	No concerns	Major concerns	No concerns	No concerns	Very low
ARI vs OLA	0	Some concerns	Suspected	No concerns	Major concerns	No concerns	No concerns	Very low
ARI vs PAL	0	Some concerns	Suspected	No concerns	Major concerns	No concerns	No concerns	Very low
ARI vs QUE	0	Some concerns	Suspected	No concerns	No concerns	No concerns	No concerns	Very low
ARI vs RIS	0	Some concerns	Suspected	No concerns	Major concerns	No concerns	No concerns	Very low
ARI vs ZIP	0	Some concerns	Suspected	No concerns	Major concerns	No concerns	No concerns	Very low
ASE vs BRE	0	Some concerns	Suspected	No concerns	Major concerns	No concerns	No concerns	Very low
ASE vs CARI	0	Some concerns	Suspected	No concerns	Major concerns	No concerns	No concerns	Very low
ASE vs HAL	0	Some concerns	Suspected	No concerns	No concerns	No concerns	No concerns	Very low
ASE vs LAM	0	Some concerns	Suspected	No concerns	Major concerns	No concerns	No concerns	Very low
ASE vs LIT	0	Some concerns	Suspected	No concerns	Major concerns	No concerns	No concerns	Very low
ASE vs PAL	0	No concerns	Suspected	No concerns	Major concerns	No concerns	No concerns	Very low
ASE vs QUE	0	Some concerns	Suspected	No concerns	Major concerns	No concerns	No concerns	Very low
ASE vs RIS	0	Some concerns	Suspected	No concerns	Major concerns	No concerns	No concerns	Very low
ASE vs ZIP	0	Some concerns	Suspected	No concerns	Major concerns	No concerns	No concerns	Very low
BRE vs CARI	0	Some concerns	Suspected	No concerns	Major concerns	No concerns	No concerns	Very low
BRE vs HAL	0	Some concerns	Suspected	No concerns	Major concerns	No concerns	No concerns	Very low
BRE vs LAM	0	Some concerns	Suspected	No concerns	Major concerns	No concerns	No concerns	Very low
BRE vs LIT	0	Some concerns	Suspected	No concerns	No concerns	No concerns	No concerns	Very low
BRE vs OLA	0	Some concerns	Suspected	No concerns	Major concerns	No concerns	No concerns	Very low
BRE vs PAL	0	Some concerns	Suspected	No concerns	Major concerns	No concerns	No concerns	Very low
BRE vs QUE	0	Some concerns	Suspected	No concerns	No concerns	No concerns	No concerns	Very low
BRE vs RIS	0	Some concerns	Suspected	No concerns	Major concerns	No concerns	No concerns	Very low
BRE vs ZIP	0	Some concerns	Suspected	No concerns	Major concerns	No concerns	No concerns	Very low

CARI vs HAL	0	Some concerns	Suspected	No concerns	Major concerns	No concerns	No concerns	Very low
CARI vs LAM	0	Some concerns	Suspected	No concerns	Major concerns	No concerns	No concerns	Very low
CARI vs LIT	0	Some concerns	Suspected	No concerns	No concerns	No concerns	No concerns	Very low
CARI vs OLA	0	Some concerns	Suspected	No concerns	Major concerns	No concerns	No concerns	Very low
CARI vs PAL	0	Some concerns	Suspected	No concerns	Major concerns	No concerns	No concerns	Very low
CARI vs QUE	0	Some concerns	Suspected	No concerns	No concerns	No concerns	No concerns	Very low
CARI vs RIS	0	Some concerns	Suspected	No concerns	Major concerns	No concerns	No concerns	Very low
CARI vs ZIP	0	Some concerns	Suspected	No concerns	Major concerns	No concerns	No concerns	Very low
HAL vs LAM	0	Some concerns	Suspected	No concerns	Major concerns	No concerns	No concerns	Very low
HAL vs LIT	0	Some concerns	Suspected	No concerns	No concerns	No concerns	No concerns	Very low
HAL vs PAL	0	Some concerns	Suspected	No concerns	Major concerns	No concerns	No concerns	Very low
HAL vs RIS	0	Some concerns	Suspected	No concerns	Major concerns	No concerns	No concerns	Very low
LAM vs OLA	0	Some concerns	Suspected	No concerns	Major concerns	No concerns	No concerns	Very low
LAM vs PAL	0	Some concerns	Suspected	No concerns	Major concerns	No concerns	No concerns	Very low
LAM vs QUE	0	Some concerns	Suspected	No concerns	Major concerns	No concerns	No concerns	Very low
LAM vs RIS	0	Some concerns	Suspected	No concerns	Major concerns	No concerns	No concerns	Very low
LAM vs ZIP	0	Some concerns	Suspected	No concerns	Major concerns	No concerns	No concerns	Very low
LIT vs OLA	0	Some concerns	Suspected	No concerns	Major concerns	No concerns	No concerns	Very low
LIT vs PAL	0	No concerns	Suspected	No concerns	Major concerns	No concerns	No concerns	Very low
LIT vs RIS	0	Some concerns	Suspected	No concerns	Major concerns	No concerns	No concerns	Very low
LIT vs ZIP	0	Some concerns	Suspected	No concerns	No concerns	No concerns	No concerns	Very low
OLA vs PAL	0	Some concerns	Suspected	No concerns	Major concerns	No concerns	No concerns	Very low
OLA vs QUE	0	Some concerns	Suspected	No concerns	Major concerns	No concerns	No concerns	Very low
OLA vs ZIP	0	Some concerns	Suspected	No concerns	Major concerns	No concerns	No concerns	Very low
PAL vs RIS	0	Some concerns	Suspected	No concerns	Major concerns	No concerns	No concerns	Very low

PAL vs ZIP	0	Some concerns	Suspected	No concerns	Major concerns	No concerns	No concerns	Very low
QUE vs RIS	0	Some concerns	Suspected	No concerns	Major concerns	No concerns	No concerns	Very low
QUE vs ZIP	0	Some concerns	Suspected	No concerns	No concerns	No concerns	No concerns	Very low
RIS vs ZIP	0	Some concerns	Suspected	No concerns	Major concerns	No concerns	No concerns	Very low
RIS vs PLA	0	Some concerns	Suspected	No concerns	No concerns	Major concerns	No concerns	Very low

Supplementary Appendix 13. Extrapyramidal symptoms (N = 31, n = 9265)

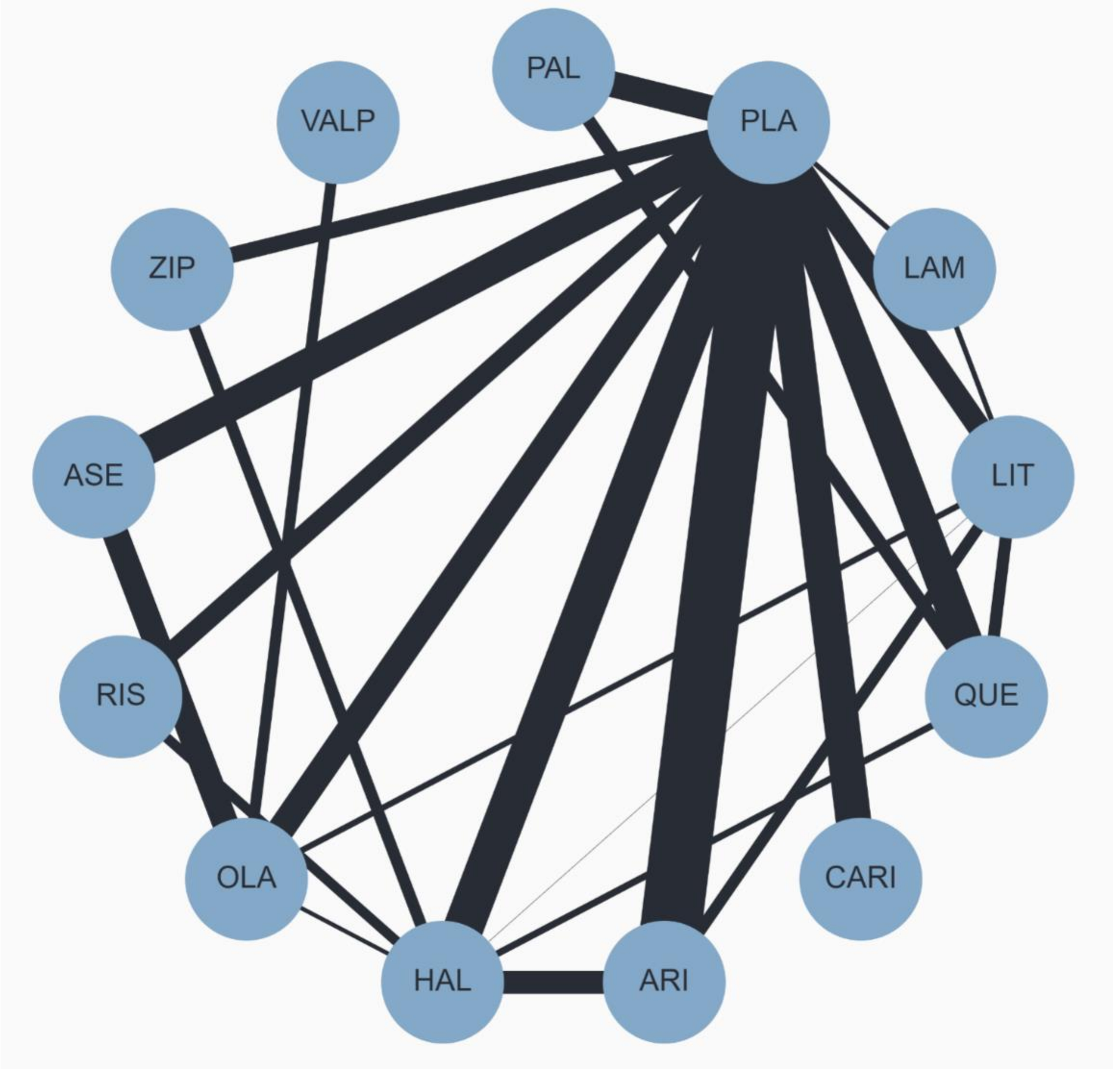

League table (risk ratio with 95% confidence interval)

ARI	0.976 (0.519, 1.836)	0.887 (0.494, 1.594)	**0.442 (0.325, 0.600)**	0.742 (0.226, 2.438)	1.162 (0.763, 1.768)	1.298 (0.691, 2.440)	1.867 (0.842, 4.144)	**2.250 (1.377, 3.677)**	0.804 (0.485, 1.331)	**4.380 (1.251, 15.337)**	0.711 (0.404, 1.251)	**2.359 (1.766, 3.150)**
	ASE	0.908 (0.421, 1.961)	**0.453 (0.241, 0.849)**	0.760 (0.208, 2.770)	1.190 (0.613, 2.309)	1.330 (0.802, 2.204)	1.913 (0.745, 4.914)	**2.305 (1.127, 4.711)**	0.823 (0.397, 1.705)	**4.486 (1.358, 14.820)**	0.728 (0.333, 1.590)	**2.416 (1.358, 4.299)**
		CARI	**0.498 (0.277, 0.896)**	0.836 (0.233, 3.009)	1.310 (0.683, 2.512)	1.464 (0.674, 3.180)	2.106 (0.849, 5.221)	**2.537 (1.287, 5.000)**	0.906 (0.457, 1.797)	**4.939 (1.303, 18.713)**	0.801 (0.381, 1.686)	**2.660 (1.598, 4.428)**
			HAL	1.678 (0.507, 5.549)	**2.629 (1.682, 4.107)**	**2.937 (1.569, 5.497)**	**4.225 (1.913, 9.331)**	**5.091 (3.197, 8.104)**	**1.818 (1.156, 2.860)**	**9.909 (2.835, 34.629)**	1.608 (0.978, 2.643)	**5.337 (3.997, 7.126)**
				LAM	1.566 (0.495, 4.953)	1.750 (0.483, 6.347)	2.518 (0.629, 10.075)	3.033 (0.887, 10.373)	1.083 (0.309, 3.795)	**5.904 (1.097, 31.776)**	0.958 (0.265, 3.459)	3.180 (0.983, 10.290)
					LIT	1.117 (0.587, 2.125)	1.607 (0.697, 3.706)	**1.937 (1.160, 3.232)**	0.692 (0.383, 1.247)	**3.770 (1.070, 13.283)**	0.612 (0.320, 1.169)	**2.030 (1.354, 3.044)**
						OLA	1.438 (0.558, 3.707)	1.733 (0.850, 3.533)	0.619 (0.298, 1.285)	**3.374 (1.142, 9.963)**	0.547 (0.251, 1.196)	**1.817 (1.012, 3.261)**
							PAL	1.205 (0.540, 2.688)	0.430 (0.180, 1.031)	2.345 (0.557, 9.882)	**0.381 (0.152, 0.953)**	1.263 (0.596, 2.678)
								QUE	**0.357 (0.194, 0.658)**	1.947 (0.533, 7.116)	**0.316 (0.163, 0.614)**	1.048 (0.670, 1.641)
									RIS	**5.451 (1.476, 20.130)**	0.885 (0.458, 1.710)	**2.936 (1.858, 4.639)**
										VALP	**0.162 (0.043, 0.617)**	0.539 (0.157, 1.844)
											ZIP	**3.319 (1.931, 5.706)**

												PLA

Evaluation of heterogeneity and inconsistency

Between study variance (τ^2)	Heterogeneity assessment	Random-effects design-by-treatment interaction model		
		Q	df	p
0.035	Low to moderate	20.528	21	0.488

Incoherence

	NMA, RR (95% CI)	Direct, RR (95% CI)	I^2	Indirect, RR (95% CI)	Inconsistency measures	
					Difference of RR	P value
ARI vs HAL	0.442 (0.325, 0.600)	0.431 (0.289, 0.643)	27.5%	0.458 (0.285, 0.738)	0.940 (0.504, 1.750)	0.844
ARI vs LIT	1.162 (0.763, 1.768)	1.422 (0.817, 2.473)	0.0%	0.884 (0.464, 1.684	1.608 (0.688, 3.762)	0.273
ARI vs PLA	2.359 (1.766, 3.150)	2.283 (1.603, 3.252)	29.7%	2.519 (1.524, 4.162)	0.906 (0.490, 1.675)	0.754
ASE vs OLA	1.330 (0.802, 2.204)	1.182 (0.684, 2.043)	4.6%	2.632 (0.703, 9.844)	0.449 (0.108, 1.874)	0.272
ASE vs PLA	2.416 (1.358, 4.299)	2.899 (1.527, 5.501)	0.0%	1.119 (0.300, 4.177)	2.592 (0.599, 11.217)	0.203
CARI vs PLA		2.660 (1.598, 4.428)	58.4%			
HAL vs LIT	2.629 (1.682, 4.107)	5.000 (0.740, 33.764)	na	2.533 (1.601, 4.008)	1.974 (0.277, 14.075)	0.497
HAL vs OLA	2.937 (1.569, 5.497)	3.676 (0.745, 18.127)	na	2.819 (1.426, 5.573)	1.304 (0.230, 7.392)	0.764
HAL vs QUE	5.091 (3.197, 8.104)	4.676 (2.446, 8.939)	na	5.571 (2.857, 10.862)	0.839 (0.331, 2.128)	0.712
HAL vs RIS	1.818 (1.156, 2.860)	2.386 (1.382, 4.118)	na	0.997 (0.443, 2.245)	2.392 (0.900, 6.360)	0.081
HAL vs ZIP	1.608 (0.978, 2.643)	1.592 (0.939, 2.699)	na	1.738 (0.397, 7.605)	0.916 (0.191, 4.390)	0.912
HAL vs PLA	5.337 (3.997, 7.126)	4.426 (3.006, 6.518)	0.0%	6.761 (4.376, 10.448)	0.655 (0.366, 1.172)	0.154
LAM vs LIT	1.566 (0.495, 4.953)	1.581 (0.440, 5.679)	na	1.505 (0.107, 21.166)	1.051 (0.056, 19.808)	0.974
LAM vs PLA	3.180 (0.983, 10.290)	3.122 (0.623, 15.630)	na	3.248 (0.584, 18.058)	0.961 (0.091, 10.113)	0.974
LIT vs OLA	1.117 (0.587, 2.125)	2.244 (0.736, 6.837)	0.0%	0.789 (0.359, 1.734)	2.844 (0.727, 11.131)	0.133
LIT vs QUE	1.937 (1.160, 3.232)	2.368 (1.090, 5.146)	30.6%	1.658 (0.838, 3.278)	1.428 (0.508, 4.014)	0.499
LIT vs PLA	2.030 (1.354, 3.044)	1.757 (1.024, 3.016)	56.0%	2.444 (1.325, 4.506)	0.719 (0.318, 1.626)	0.428

OLA vs VAL		3.374 (1.142, 9.963)	0.0%			
OLA vs PLA	1.817 (1.012, 3.261)	2.697 (1.138, 6.388)	0.0%	1.298 (0.586, 2.877)	2.077 (0.642, 6.714)	0.222
PAL vs QUE	1.205 (0.540, 2.688)	1.979 (0.573, 6.837)	na	0.842 (0.294, 2.413)	2.351 (0.462, 11.956)	0.303
PAL vs PLA	1.263 (0.596, 2.678)	1.086 (0.466, 2.532)	11.1%	2.215 (0.433, 11.319)	0.490 (0.078, 3.081)	0.447
QUE vs PLA	1.048 (0.670, 1.641)	1.108 (0.650, 1.889)	0.0%	0.917 (0.402, 2.094)	1.208 (0.452, 3.227)	0.706
RIS vs PLA	2.936 (1.858, 4.639)	3.267 (1.918, 5.566)	79.3%	2.173 (0.889, 5.310)	1.504 (0.531, 4.255)	0.442
ZIP vs PLA	3.319 (1.931, 5.706)	2.827 (1.284, 6.221)	0.0%	3.831 (1.817, 8.074)	0.738 (0.249, 2.185)	0.583

Forest plot (vs placebo, the numbers are risk ratio with 95% confidence interval)

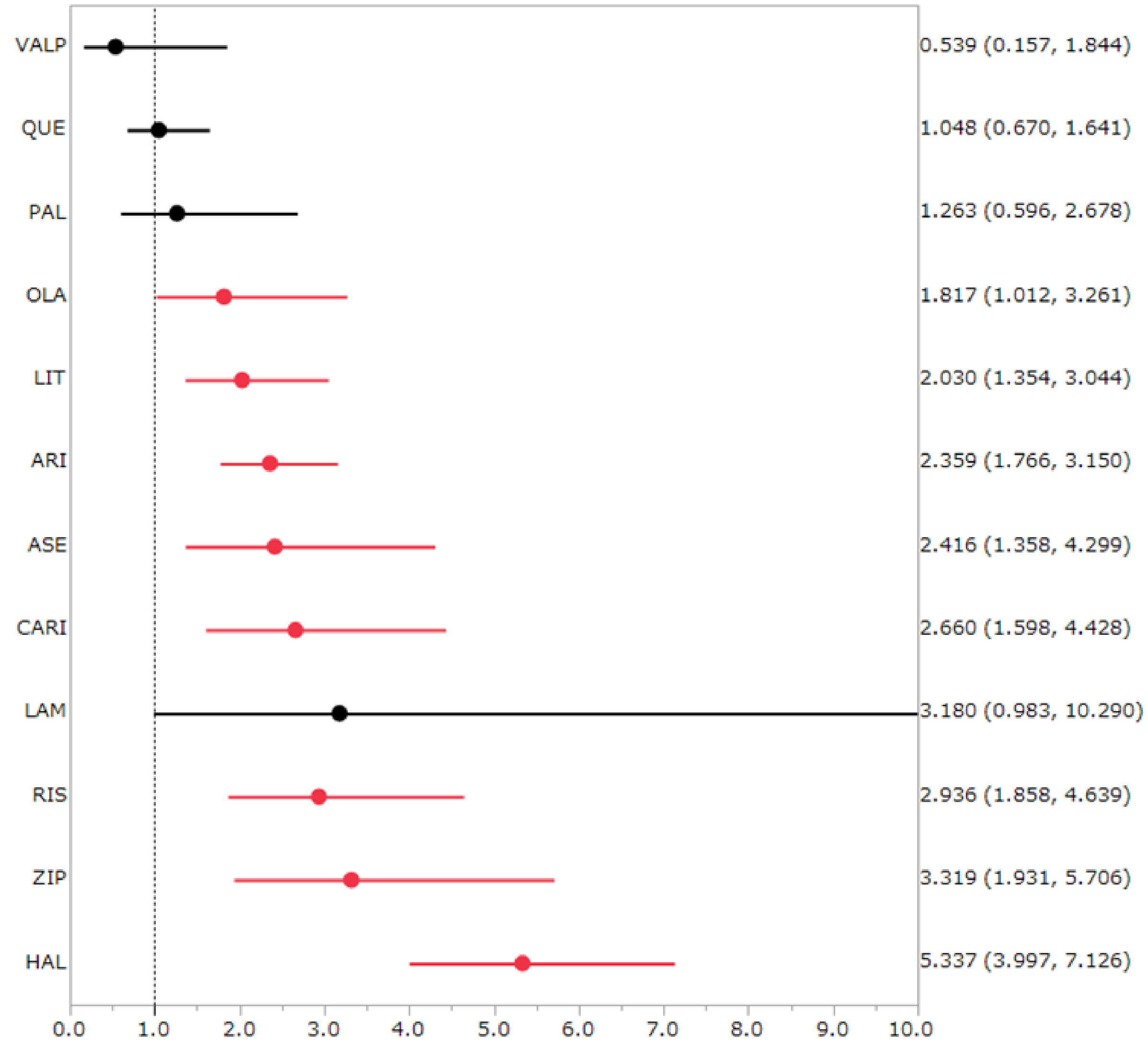

P-score

VALP	0.958
QUE	0.846
PAL	0.742
OLA	0.573
LIT	0.535
ARI	0.422
ASE	0.420
CARI	0.345
LAM	0.336
RIS	0.252
ZIP	0.191
HAL	0.012

Funnel plot (only double-blind, placebo-controlled trials)

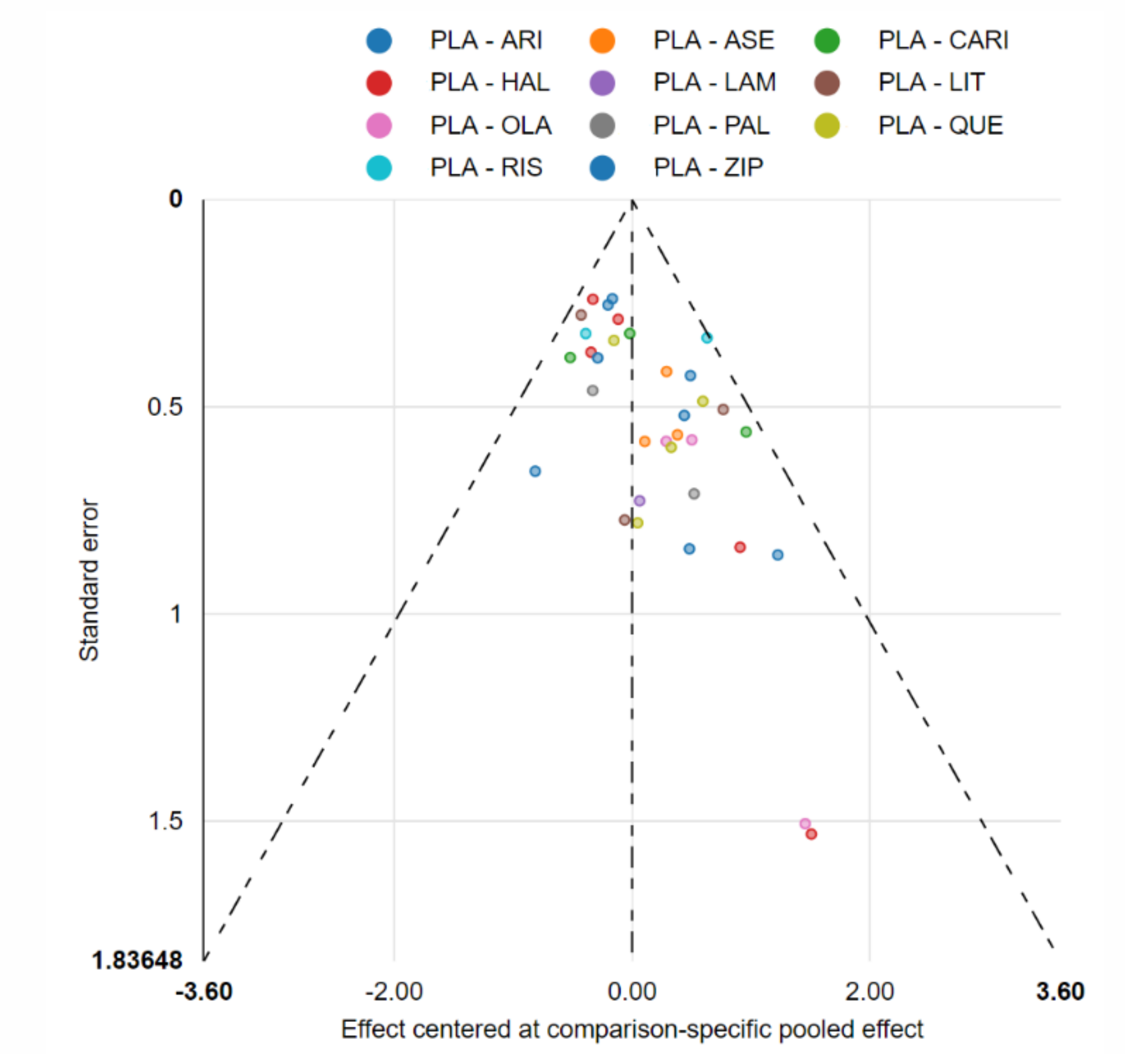

CINeMA confidence rating

Comparison	Number of studies	Within-study bias	Reporting bias	Indirectness	Imprecision	Heterogeneity	Incoherence	Confidence rating
ARI vs HAL	2	Some concerns	Suspected	No concerns	No concerns	No concerns	No concerns	Low
ARI vs LIT	2	Some concerns	Suspected	No concerns	Major concerns	No concerns	No concerns	Very low
ARI vs PLA	6	Some concerns	Suspected	No concerns	No concerns	No concerns	No concerns	Low
ASE vs OLA	2	Some concerns	Suspected	No concerns	Major concerns	No concerns	No concerns	Very low
ASE vs PLA	3	Some concerns	Suspected	No concerns	No concerns	No concerns	No concerns	Low
CARI vs PLA	3	Some concerns	Suspected	No concerns	No concerns	No concerns	No concerns	Low
HAL vs LIT	1	Some concerns	Suspected	No concerns	No concerns	No concerns	No concerns	Low
HAL vs OLA	1	Some concerns	Suspected	No concerns	No concerns	No concerns	No concerns	Low
HAL vs QUE	1	Some concerns	Suspected	No concerns	No concerns	No concerns	No concerns	Low
HAL vs RIS	1	Some concerns	Suspected	No concerns	No concerns	Major concerns	Major concerns	Very low
HAL vs ZIP	1	Some concerns	Suspected	No concerns	Major concerns	No concerns	No concerns	Very low
HAL vs PLA	5	Some concerns	Suspected	No concerns	No concerns	No concerns	No concerns	Low
LAM vs LIT	1	Some concerns	Suspected	No concerns	Major concerns	No concerns	No concerns	Very low
LAM vs PLA	1	Some concerns	Suspected	No concerns	Major concerns	No concerns	No concerns	Very low
LIT vs OLA	2	Some concerns	Suspected	No concerns	Major concerns	No concerns	No concerns	Very low
LIT vs QUE	2	Some concerns	Suspected	No concerns	No concerns	No concerns	No concerns	Low
LIT vs PLA	3	Some concerns	Suspected	No concerns	No concerns	No concerns	No concerns	Low
OLA vs VALP	2	No concerns	Suspected	No concerns	No concerns	No concerns	No concerns	Moderate
OLA vs PLA	3	Some concerns	Suspected	No concerns	No concerns	Major concerns	No concerns	Very low
PAL vs QUE	1	No concerns	Suspected	No concerns	Major concerns	No concerns	No concerns	Low
PAL vs PLA	2	No concerns	Suspected	No concerns	Major concerns	No concerns	No concerns	Low
QUE vs PLA	4	Some concerns	Suspected	No concerns	Major concerns	No concerns	No concerns	Very low
RIS vs PLA	2	Some concerns	Suspected	No concerns	No concerns	No concerns	No concerns	Low

ZIP vs PLA	2	Some concerns	Suspected	No concerns	No concerns	No concerns	No concerns	Low
ARI vs ASE	0	Some concerns	Suspected	No concerns	Major concerns	No concerns	No concerns	Very low
ARI vs CARI	0	Some concerns	Suspected	No concerns	Major concerns	No concerns	No concerns	Very low
ARI vs LAM	0	Some concerns	Suspected	No concerns	Major concerns	No concerns	No concerns	Very low
ARI vs OLA	0	Some concerns	Suspected	No concerns	Major concerns	No concerns	No concerns	Very low
ARI vs PAL	0	Some concerns	Suspected	No concerns	Major concerns	No concerns	No concerns	Very low
ARI vs QUE	0	Some concerns	Suspected	No concerns	No concerns	No concerns	No concerns	Very low
ARI vs RIS	0	Some concerns	Suspected	No concerns	Major concerns	No concerns	No concerns	Very low
ARI vs VALP	0	Some concerns	Suspected	No concerns	No concerns	No concerns	No concerns	Very low
ARI vs ZIP	0	Some concerns	Suspected	No concerns	Major concerns	No concerns	No concerns	Very low
ASE vs CARI	0	Some concerns	Suspected	No concerns	Major concerns	No concerns	No concerns	Very low
ASE vs HAL	0	Some concerns	Suspected	No concerns	No concerns	No concerns	No concerns	Very low
ASE vs LAM	0	Some concerns	Suspected	No concerns	Major concerns	No concerns	No concerns	Very low
ASE vs LIT	0	Some concerns	Suspected	No concerns	Major concerns	No concerns	No concerns	Very low
ASE vs PAL	0	No concerns	Suspected	No concerns	Major concerns	No concerns	No concerns	Very low
ASE vs QUE	0	Some concerns	Suspected	No concerns	No concerns	Major concerns	No concerns	Very low
ASE vs RIS	0	Some concerns	Suspected	No concerns	Major concerns	No concerns	No concerns	Very low
ASE vs VALP	0	Some concerns	Suspected	No concerns	No concerns	No concerns	No concerns	Very low
ASE vs ZIP	0	Some concerns	Suspected	No concerns	Major concerns	No concerns	No concerns	Very low
CARI vs HAL	0	Some concerns	Suspected	No concerns	No concerns	Major concerns	No concerns	Very low
CARI vs LAM	0	Some concerns	Suspected	No concerns	Major concerns	No concerns	No concerns	Very low
CARI vs LIT	0	Some concerns	Suspected	No concerns	Major concerns	No concerns	No concerns	Very low
CARI vs OLA	0	Some concerns	Suspected	No concerns	Major concerns	No concerns	No concerns	Very low
CARI vs PAL	0	Some concerns	Suspected	No concerns	Major concerns	No concerns	No concerns	Very low
CARI vs QUE	0	Some concerns	Suspected	No concerns	No concerns	No concerns	No concerns	Very low

CARI vs RIS	0	Some concerns	Suspected	No concerns	Major concerns	No concerns	No concerns	Very low
CARI vs VALP	0	Some concerns	Suspected	No concerns	No concerns	No concerns	No concerns	Very low
CARI vs ZIP	0	Some concerns	Suspected	No concerns	Major concerns	No concerns	No concerns	Very low
HAL vs LAM	0	Some concerns	Suspected	No concerns	Major concerns	No concerns	No concerns	Very low
HAL vs PAL	0	Some concerns	Suspected	No concerns	No concerns	No concerns	No concerns	Very low
HAL vs VALP	0	Some concerns	Suspected	No concerns	No concerns	No concerns	No concerns	Very low
LAM vs OLA	0	Some concerns	Suspected	No concerns	Major concerns	No concerns	No concerns	Very low
LAM vs PAL	0	Some concerns	Suspected	No concerns	Major concerns	No concerns	No concerns	Very low
LAM vs QUE	0	Some concerns	Suspected	No concerns	Major concerns	No concerns	No concerns	Very low
LAM vs RIS	0	Some concerns	Suspected	No concerns	Major concerns	No concerns	No concerns	Very low
LAM vs VALP	0	Some concerns	Suspected	No concerns	No concerns	Major concerns	No concerns	Very low
LAM vs ZIP	0	Some concerns	Suspected	No concerns	Major concerns	No concerns	No concerns	Very low
LIT vs PAL	0	No concerns	Suspected	No concerns	Major concerns	No concerns	No concerns	Very low
LIT vs RIS	0	Some concerns	Suspected	No concerns	Major concerns	No concerns	No concerns	Very low
LIT vs VALP	0	Some concerns	Suspected	No concerns	No concerns	Major concerns	No concerns	Very low
LIT vs ZIP	0	Some concerns	Suspected	No concerns	Major concerns	No concerns	No concerns	Very low
OLA vs PAL	0	No concerns	Suspected	No concerns	Major concerns	No concerns	No concerns	Very low
OLA vs QUE	0	Some concerns	Suspected	No concerns	Major concerns	No concerns	No concerns	Very low
OLA vs RIS	0	Some concerns	Suspected	No concerns	Major concerns	No concerns	No concerns	Very low
OLA vs ZIP	0	Some concerns	Suspected	No concerns	Major concerns	No concerns	No concerns	Very low
PAL vs RIS	0	Some concerns	Suspected	No concerns	Major concerns	No concerns	No concerns	Very low
PAL vs VALP	0	No concerns	Suspected	No concerns	Major concerns	No concerns	No concerns	Very low
PAL vs ZIP	0	Some concerns	Suspected	No concerns	No concerns	Major concerns	No concerns	Very low
QUE vs RIS	0	Some concerns	Suspected	No concerns	No concerns	No concerns	No concerns	Very low
QUE vs VALP	0	No concerns	Suspected	No concerns	Major concerns	No concerns	No concerns	Very low

QUE vs ZIP	0	Some concerns	Suspected	No concerns	No concerns	No concerns	No concerns	Very low
RIS vs VALP	0	Some concerns	Suspected	No concerns	No concerns	No concerns	No concerns	Very low
RIS vs ZIP	0	Some concerns	Suspected	No concerns	Major concerns	No concerns	No concerns	Very low
VALP vs ZIP	0	Some concerns	Suspected	No concerns	No concerns	No concerns	No concerns	Very low
VALP vs PLA	0	Some concerns	Suspected	No concerns	Major concerns	No concerns	No concerns	Very low

Supplementary Appendix 14. Somnolence (N = 37, n = 10395)

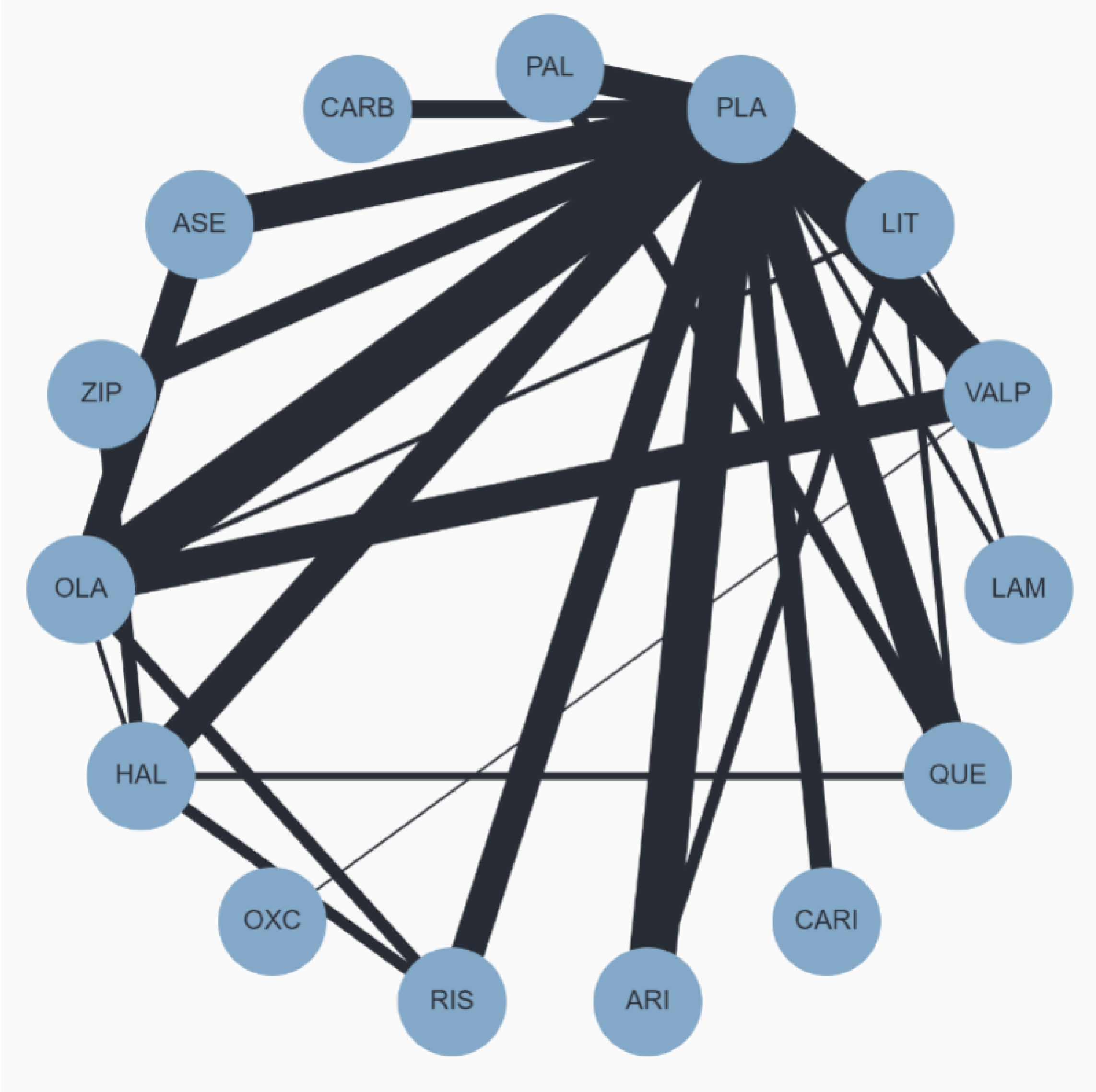

League table (risk ratio with 95% confidence interval)

ARI	**0.516 (0.299, 0.892)**	0.874 (0.511, 1.493)	0.414 (0.115, 1.483)	0.662 (0.359, 1.218)	1.625 (0.289, 9.126)	1.326 (0.813, 2.163)	**0.620 (0.404, 0.953)**	1.874 (0.459, 7.655)	0.974 (0.521, 1.821)	**0.536 (0.321, 0.896)**	0.679 (0.418, 1.104)	1.124 (0.729, 1.734)	0.861 (0.456, 1.627)	**2.134 (1.490, 3.057)**
	ASE	1.692 (0.952, 3.007)	0.801 (0.220, 2.922)	1.282 (0.682, 2.408)	3.147 (0.547, 18.103)	**2.568 (1.439, 4.583)**	1.202 (0.821, 1.760)	3.630 (0.889, 14.823)	1.886 (0.970, 3.667)	1.039 (0.592, 1.823)	1.316 (0.823, 2.104)	**2.178 (1.413, 3.357)**	1.668 (0.855, 3.255)	**4.133 (2.727, 6.264)**
		CARB	0.473 (0.131, 1.716)	0.758 (0.400, 1.434)	1.860 (0.324, 10.688)	1.518 (0.850, 2.709)	0.710 (0.446, 1.132)	2.145 (0.519, 8.868)	1.115 (0.578, 2.151)	0.614 (0.352, 1.070)	0.777 (0.464, 1.304)	1.287 (0.804, 2.060)	0.986 (0.511, 1.904)	**2.442 (1.642, 3.634)**
			CARI	1.600 (0.426, 6.006)	3.928 (0.482, 32.005)	3.206 (0.877, 11.712)	1.500 (0.430, 5.230)	4.530 (0.725, 28.307)	2.354 (0.621, 8.922)	1.296 (0.358, 4.688)	1.642 (0.462, 5.841)	2.718 (0.778, 9.495)	2.082 (0.549, 7.895)	**5.158 (1.515, 17.561)**
				HAL	2.455 (0.419, 14.385)	**2.004 (1.068, 3.759)**	0.937 (0.555, 1.584)	2.832 (0.668, 12.001)	1.471 (0.747, 2.898)	0.810 (0.465, 1.412)	1.026 (0.589, 1.789)	1.699 (0.989, 2.919)	1.302 (0.645, 2.627)	**3.224 (1.957, 5.312)**
					LAM	0.816 (0.153, 4.361)	0.382 (0.069, 2.123)	1.153 (0.131, 10.162)	0.599 (0.102, 3.513)	0.330 (0.059, 1.857)	0.418 (0.074, 2.360)	0.692 (0.124, 3.847)	0.530 (0.089, 3.148)	1.313 (0.239, 7.208)
						LIT	**0.468 (0.294, 0.745)**	1.413 (0.343, 5.828)	0.734 (0.393, 1.373)	**0.404 (0.247, 0.661)**	**0.512 (0.304, 0.862)**	0.848 (0.534, 1.347)	0.650 (0.332, 1.272)	**1.609 (1.055, 2.453)**
							OLA	3.021 (0.776, 11.762)	1.570 (0.886, 2.779)	0.864 (0.552, 1.352)	1.095 (0.819, 1.463)	**1.812 (1.432, 2.293)**	1.388 (0.780, 2.472)	**3.439 (2.695, 4.389)**
								OXC	0.520 (0.121, 2.232)	0.286 (0.070, 1.176)	0.362 (0.091, 1.447)	0.600 (0.157, 2.289)	0.460 (0.107, 1.978)	1.139 (0.292, 4.447)
									PAL	**0.551 (0.346, 0.876)**	0.698 (0.378, 1.287)	1.155 (0.649, 2.053)	0.885 (0.423, 1.852)	**2.191 (1.298, 3.700)**
										QUE	1.267 (0.769, 2.086)	**2.097 (1.333, 3.300)**	1.607 (0.840, 3.074)	**3.980 (2.696, 5.874)**
											RIS	**1.655 (1.163, 2.357)**	1.268 (0.684, 2.353)	**3.142 (2.256, 4.375)**

												VALP	0.766 (0.428, 1.370)	**1.898 (1.475, 2.442)**
													ZIP	**2.477 (1.466, 4.186)**
														PLA

Evaluation of heterogeneity and inconsistency

Between study variance (τ^2)	Heterogeneity assessment	Random-effects design-by-treatment interaction model		
		Q	df	p
0.000	Low	21.238	22	0.506

Incoherence

	NMA, RR (95% CI)	Direct, RR (95% CI)	I^2	Indirect, RR (95% CI)	Inconsistency measures	
					Difference of RR	P value
ARI vs LIT	1.326 (0.813, 2.163)	1.689 (0.825, 3.459)	na	1.073 (0.549, 2.097)	1.575 (0.590, 4.200)	0.364
ARI vs PLA	2.134 (1.490, 3.057)	2.039 (1.399, 2.971)	53.8%	3.401 (1.021, 11.336)	0.599 (0.170, 2.116)	0.426
ASE vs OLA	1.202 (0.821, 1.760)	1.107 (0.722, 1.697)	0.0%	1.661 (0.711, 3.883)	0.666 (0.258, 1.723)	0.402
ASE vs PLA	4.133 (2.727, 6.264)	4.911 (2.595, 9.293)	0.0%	3.638 (2.102, 6.296)	1.350 (0.582, 3.131)	0.484
CARB vs PLA		2.442 (1.642, 3.634)	0.0%			
CARI vs PLA		5.158 (1.515, 17.561)	0.0%			
HAL vs OLA	0.937 (0.555, 1.584)	0.750 (0.247, 2.278)	na	0.999 (0.551, 1.812)	0.750 (0.213, 2.647)	0.655
HAL vs QUE	0.810 (0.465, 1.412)	0.713 (0.319, 1.593)	na	0.910 (0.422, 1.963)	0.784 (0.258, 2.382)	0.667
HAL vs RIS	1.026 (0.589, 1.789)	0.764 (0.248, 2.353)	na	1.129 (0.596, 2.139)	0.677 (0.186, 2.467)	0.554
HAL vs ZIP	1.302 (0.645, 2.627)	27.064 (3.714, 197.256)	na	0.844 (0.398, 1.788)	32.066 (3.836, 268.057)	0.001
HAL vs PLA	3.224 (1.957, 5.312)	2.429 (1.316, 4.484)	0.0%	5.633 (2.383, 13.314)	0.431 (0.150, 1.240)	0.119
LAM vs LIT	0.816 (0.153, 4.361)	0.703 (0.121, 4.087)	na	3.454 (0.015, 819.341)	0.203 (0.001, 63.644)	0.587
LAM vs PLA	1.313 (0.239, 7.208)	2.081 (0.193, 22.466)	na	0.810 (0.071, 9.272)	2.570 (0.085, 77.533)	0.587
LIT vs OLA	0.468 (0.294, 0.745)	0.389 (0.078, 1.937)	na	0.476 (0.293, 0.773)	0.817 (0.153, 4.374)	0.813
LIT vs QUE	0.404 (0.247, 0.661)	0.468 (0.225, 0.972)	na	0.358 (0.184, 0.697)	1.306 (0.486, 3.510)	0.597
LIT vs VALP	0.848 (0.534, 1.347)	1.032 (0.452, 2.357)	na	0.775 (0.443, 1.356)	1.332 (0.491, 3.611)	0.573
LIT vs PLA	1.609 (1.055, 2.453)	1.636 (0.954, 2.803)	0.0%	1.568 (0.797, 3.088)	1.043 (0.439, 2.478)	0.924

OLA vs RIS	1.095 (0.819, 1.463)	1.149 (0.821, 1.606)	na	0.949 (0.532, 1.693)	1.210 (0.620, 2.361)	0.577
OLA vs VALP	1.812 (1.432, 2.293)	1.932 (1.462, 2.552)	0.0%	1.547 (0.997, 2.400)	1.249 (0.742, 2.101)	0.402
OLA vs PLA	3.439 (2.695, 4.389)	2.694 (1.869, 3.884)	0.0%	4.181 (3.014, 5.798)	0.644 (0.395, 1.053)	0.079
OXC vs VALP		0.600 (0.157, 2.289)	na			
PAL vs QUE	0.551 (0.346, 0.876)	0.537 (0.319, 0.905)	na	0.605 (0.219, 1.672)	0.889 (0.283, 2.787)	0.840
PAL vs PLA	2.191 (1.298, 3.700)	2.384 (1.193, 4.764)	0.0%	1.958 (0.879, 4.362)	1.218 (0.422, 3.511)	0.715
QUE vs PLA	3.980 (2.696, 5.874)	3.980 (2.450, 6.464)	0.0%	3.979 (2.072, 7.642)	1.000 (0.444, 2.255)	1.000
RIS vs PLA	3.142 (2.256, 4.375)	3.366 (1.941, 5.838)	0.0%	3.021 (1.997, 4.573)	1.114 (0.559, 2.219)	0.759
VALP vs PLA	1.898 (1.475, 2.442)	1.993 (1.448, 2.745)	0.3%	1.750 (1.161, 2.638)	1.139 (0.677, 1.916)	0.625
ZIP vs PLA	2.477 (1.466, 4.186)	2.640 (1.557, 4.475)	68.5%	0.013 (0.000, 1.560)	207.493 (1.643, 26196.995)	0.031

Forest plot (vs placebo, the numbers are risk ratio with 95% confidence interval)

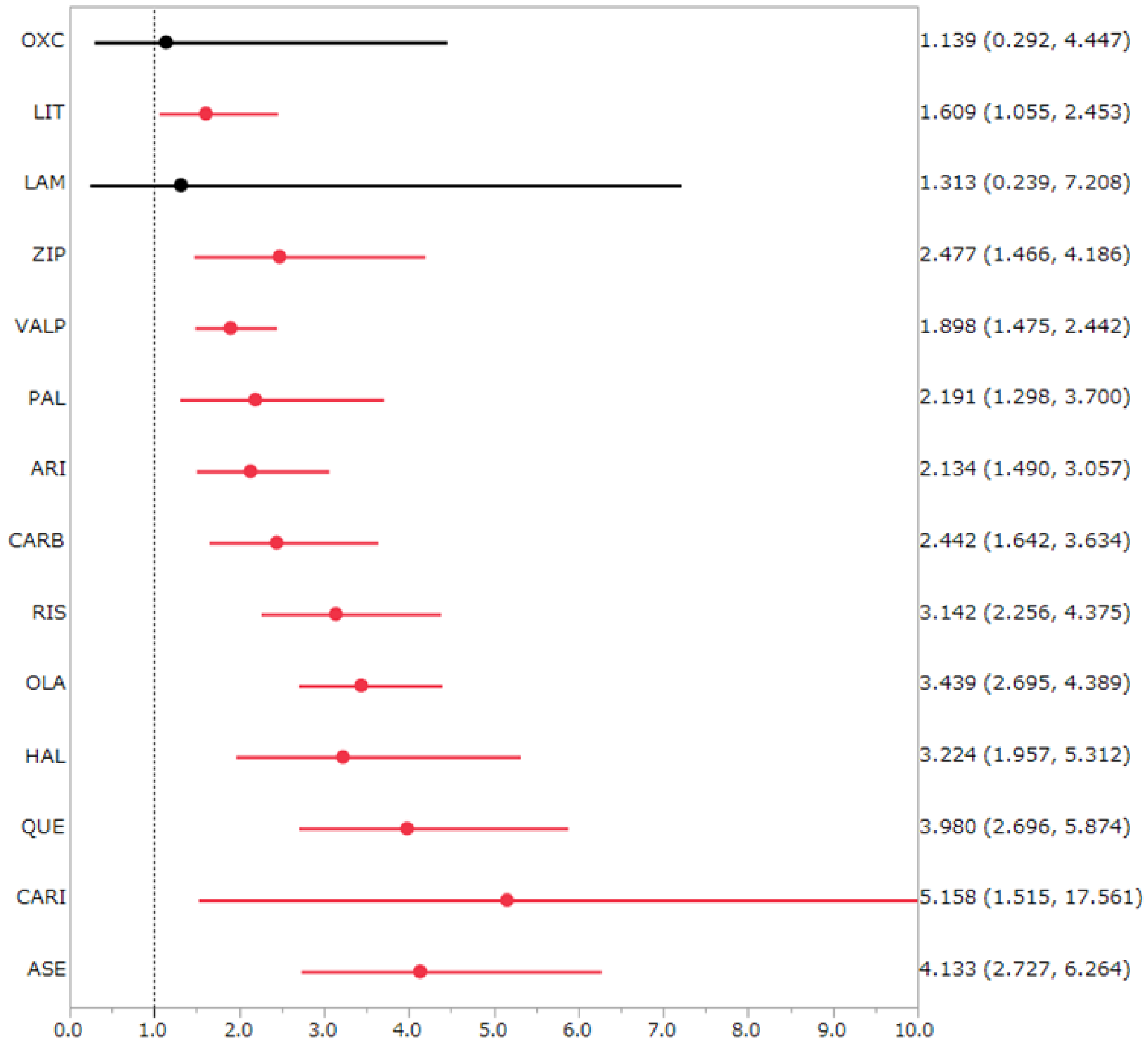

P-score

OXC	0.816
LIT	0.746
LAM	0.744
ZIP	0.691
VALP	0.688
PAL	0.577
ARI	0.575
CARB	0.453
RIS	0.310
OLA	0.232
HAL	0.210
QUE	0.187
CARI	0.186
ASE	0.147

Funnel plot (only double-blind, placebo-controlled trials)

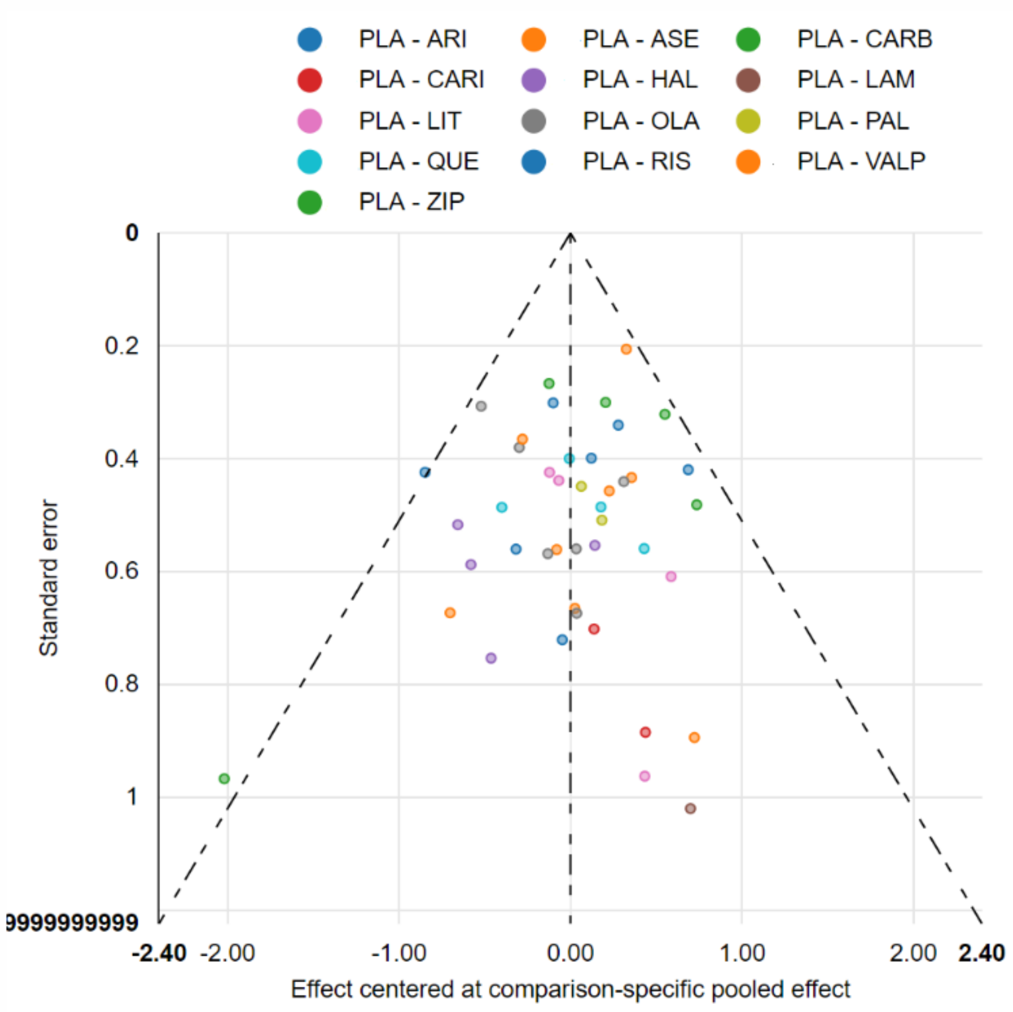

CINeMA confidence rating

Comparison	Number of studies	Within-study bias	Reporting bias	Indirectness	Imprecision	Heterogeneity	Incoherence	Confidence rating
ARI vs LIT	1	Some concerns	Suspected	No concerns	Major concerns	No concerns	No concerns	Very low
ARI vs PLA	4	Some concerns	Suspected	No concerns	No concerns	No concerns	No concerns	Low
ASE vs OLA	2	Some concerns	Suspected	No concerns	Major concerns	No concerns	No concerns	Very low
ASE vs PLA	3	Some concerns	Suspected	No concerns	No concerns	No concerns	No concerns	Low
CARB vs PLA	2	Some concerns	Suspected	No concerns	No concerns	No concerns	No concerns	Low
CARI vs PLA	2	Some concerns	Suspected	No concerns	No concerns	No concerns	No concerns	Low
HAL vs OLA	1	Some concerns	Suspected	No concerns	Major concerns	No concerns	No concerns	Very low
HAL vs QUE	1	Some concerns	Suspected	No concerns	Major concerns	No concerns	No concerns	Very low
HAL vs RIS	1	Some concerns	Suspected	No concerns	Major concerns	No concerns	No concerns	Very low
HAL vs ZIP	1	Some concerns	Suspected	No concerns	Major concerns	No concerns	Major concerns	Very low
HAL vs PLA	4	Some concerns	Suspected	No concerns	No concerns	No concerns	No concerns	Low
LAM vs LIT	1	Some concerns	Suspected	No concerns	Major concerns	No concerns	No concerns	Very low
LAM vs PLA	1	Some concerns	Suspected	No concerns	Major concerns	No concerns	No concerns	Very low
LIT vs OLA	1	Some concerns	Suspected	No concerns	No concerns	No concerns	No concerns	Low
LIT vs QUE	1	No concerns	Suspected	No concerns	No concerns	No concerns	No concerns	Moderate
LIT vs VALP	1	Some concerns	Suspected	No concerns	Major concerns	No concerns	No concerns	Very low
LIT vs PLA	4	Some concerns	Suspected	No concerns	No concerns	No concerns	No concerns	Low
OLA vs RIS	1	Some concerns	Suspected	No concerns	Major concerns	No concerns	No concerns	Very low
OLA vs VALP	4	No concerns	Suspected	No concerns	No concerns	No concerns	No concerns	Moderate
OLA vs PLA	6	Some concerns	Suspected	No concerns	No concerns	No concerns	No concerns	Low
OXC vs VALP	1	Some concerns	Suspected	No concerns	Major concerns	No concerns	No concerns	Very low
PAL vs QUE	1	No concerns	Suspected	No concerns	No concerns	No concerns	No concerns	Moderate
PAL vs PLA	2	No concerns	Suspected	No concerns	No concerns	No concerns	No concerns	Moderate

QUE vs PLA	4	No concerns	Suspected	No concerns	No concerns	No concerns	No concerns	Moderate
RIS vs PLA	3	Some concerns	Suspected	No concerns	No concerns	No concerns	No concerns	Low
VALP vs PLA	5	No concerns	Suspected	No concerns	No concerns	No concerns	No concerns	Moderate
ZIP vs PLA	3	Some concerns	Suspected	No concerns	No concerns	No concerns	Major concerns	Very low
ARI vs ASE	0	Some concerns	Suspected	No concerns	No concerns	No concerns	No concerns	Very low
ARI vs CARB	0	Some concerns	Suspected	No concerns	Major concerns	No concerns	No concerns	Very low
ARI vs CARI	0	Some concerns	Suspected	No concerns	Major concerns	No concerns	No concerns	Very low
ARI vs HAL	0	Some concerns	Suspected	No concerns	Major concerns	No concerns	No concerns	Very low
ARI vs LAM	0	Some concerns	Suspected	No concerns	Major concerns	No concerns	No concerns	Very low
ARI vs OLA	0	Some concerns	Suspected	No concerns	No concerns	No concerns	No concerns	Very low
ARI vs OXC	0	Some concerns	Suspected	No concerns	Major concerns	No concerns	No concerns	Very low
ARI vs PAL	0	No concerns	Suspected	No concerns	Major concerns	No concerns	No concerns	Very low
ARI vs QUE	0	Some concerns	Suspected	No concerns	No concerns	No concerns	No concerns	Very low
ARI vs RIS	0	Some concerns	Suspected	No concerns	Major concerns	No concerns	No concerns	Very low
ARI vs VALP	0	Some concerns	Suspected	No concerns	Major concerns	No concerns	No concerns	Very low
ARI vs ZIP	0	Some concerns	Suspected	No concerns	Major concerns	No concerns	No concerns	Very low
ASE vs CARB	0	Some concerns	Suspected	No concerns	Major concerns	No concerns	No concerns	Very low
ASE vs CARI	0	Some concerns	Suspected	No concerns	Major concerns	No concerns	No concerns	Very low
ASE vs HAL	0	Some concerns	Suspected	No concerns	Major concerns	No concerns	No concerns	Very low
ASE vs LAM	0	Some concerns	Suspected	No concerns	Major concerns	No concerns	No concerns	Very low
ASE vs LIT	0	Some concerns	Suspected	No concerns	No concerns	No concerns	No concerns	Very low
ASE vs OXC	0	Some concerns	Suspected	No concerns	Major concerns	No concerns	No concerns	Very low
ASE vs PAL	0	No concerns	Suspected	No concerns	Major concerns	No concerns	No concerns	Very low
ASE vs QUE	0	Some concerns	Suspected	No concerns	Major concerns	No concerns	No concerns	Very low
ASE vs RIS	0	Some concerns	Suspected	No concerns	Major concerns	No concerns	No concerns	Very low

ASE vs VALP	0	Some concerns	Suspected	No concerns	No concerns	No concerns	No concerns	Very low
ASE vs ZIP	0	Some concerns	Suspected	No concerns	Major concerns	No concerns	No concerns	Very low
CARB vs CARI	0	Some concerns	Suspected	No concerns	Major concerns	No concerns	No concerns	Very low
CARB vs HAL	0	Some concerns	Suspected	No concerns	Major concerns	No concerns	No concerns	Very low
CARB vs LAM	0	Some concerns	Suspected	No concerns	Major concerns	No concerns	No concerns	Very low
CARB vs LIT	0	Some concerns	Suspected	No concerns	Major concerns	No concerns	No concerns	Very low
CARB vs OLA	0	Some concerns	Suspected	No concerns	Major concerns	No concerns	No concerns	Very low
CARB vs OXC	0	Some concerns	Suspected	No concerns	Major concerns	No concerns	No concerns	Very low
CARB vs PAL	0	No concerns	Suspected	No concerns	Major concerns	No concerns	No concerns	Very low
CARB vs QUE	0	Some concerns	Suspected	No concerns	Major concerns	No concerns	No concerns	Very low
CARB vs RIS	0	Some concerns	Suspected	No concerns	Major concerns	No concerns	No concerns	Very low
CARB vs VALP	0	Some concerns	Suspected	No concerns	Major concerns	No concerns	No concerns	Very low
CARB vs ZIP	0	Some concerns	Suspected	No concerns	Major concerns	No concerns	No concerns	Very low
CARI vs HAL	0	Some concerns	Suspected	No concerns	Major concerns	No concerns	No concerns	Very low
CARI vs LAM	0	Some concerns	Suspected	No concerns	Major concerns	No concerns	No concerns	Very low
CARI vs LIT	0	Some concerns	Suspected	No concerns	Major concerns	No concerns	No concerns	Very low
CARI vs OLA	0	Some concerns	Suspected	No concerns	Major concerns	No concerns	No concerns	Very low
CARI vs OXC	0	Some concerns	Suspected	No concerns	Major concerns	No concerns	No concerns	Very low
CARI vs PAL	0	No concerns	Suspected	No concerns	Major concerns	No concerns	No concerns	Very low
CARI vs QUE	0	Some concerns	Suspected	No concerns	Major concerns	No concerns	No concerns	Very low
CARI vs RIS	0	Some concerns	Suspected	No concerns	Major concerns	No concerns	No concerns	Very low
CARI vs VALP	0	Some concerns	Suspected	No concerns	Major concerns	No concerns	No concerns	Very low
CARI vs ZIP	0	Some concerns	Suspected	No concerns	Major concerns	No concerns	No concerns	Very low
HAL vs LAM	0	Some concerns	Suspected	No concerns	Major concerns	No concerns	No concerns	Very low
HAL vs LIT	0	Some concerns	Suspected	No concerns	No concerns	No concerns	No concerns	Very low

HAL vs OXC	0	Some concerns	Suspected	No concerns	Major concerns	No concerns	No concerns	Very low
HAL vs PAL	0	Some concerns	Suspected	No concerns	Major concerns	No concerns	No concerns	Very low
HAL vs VALP	0	Some concerns	Suspected	No concerns	Major concerns	No concerns	No concerns	Very low
LAM vs OLA	0	Some concerns	Suspected	No concerns	Major concerns	No concerns	No concerns	Very low
LAM vs OXC	0	Some concerns	Suspected	No concerns	Major concerns	No concerns	No concerns	Very low
LAM vs PAL	0	Some concerns	Suspected	No concerns	Major concerns	No concerns	No concerns	Very low
LAM vs QUE	0	Some concerns	Suspected	No concerns	Major concerns	No concerns	No concerns	Very low
LAM vs RIS	0	Some concerns	Suspected	No concerns	Major concerns	No concerns	No concerns	Very low
LAM vs VALP	0	Some concerns	Suspected	No concerns	Major concerns	No concerns	No concerns	Very low
LAM vs ZIP	0	Some concerns	Suspected	No concerns	Major concerns	No concerns	No concerns	Very low
LIT vs OXC	0	Some concerns	Suspected	No concerns	Major concerns	No concerns	No concerns	Very low
LIT vs PAL	0	No concerns	Suspected	No concerns	Major concerns	No concerns	No concerns	Very low
LIT vs RIS	0	Some concerns	Suspected	No concerns	No concerns	No concerns	No concerns	Very low
LIT vs ZIP	0	Some concerns	Suspected	No concerns	Major concerns	No concerns	No concerns	Very low
OLA vs OXC	0	Some concerns	Suspected	No concerns	Major concerns	No concerns	No concerns	Very low
OLA vs PAL	0	No concerns	Suspected	No concerns	Major concerns	No concerns	No concerns	Very low
OLA vs QUE	0	Some concerns	Suspected	No concerns	Major concerns	No concerns	No concerns	Very low
OLA vs ZIP	0	Some concerns	Suspected	No concerns	Major concerns	No concerns	No concerns	Very low
OXC vs PAL	0	No concerns	Suspected	No concerns	Major concerns	No concerns	No concerns	Very low
OXC vs QUE	0	Some concerns	Suspected	No concerns	Major concerns	No concerns	No concerns	Very low
OXC vs RIS	0	Some concerns	Suspected	No concerns	Major concerns	No concerns	No concerns	Very low
OXC vs ZIP	0	Some concerns	Suspected	No concerns	Major concerns	No concerns	No concerns	Very low
OXC vs PLA	0	Some concerns	Suspected	No concerns	Major concerns	No concerns	No concerns	Very low
PAL vs RIS	0	Some concerns	Suspected	No concerns	Major concerns	No concerns	No concerns	Very low
PAL vs VALP	0	No concerns	Suspected	No concerns	Major concerns	No concerns	No concerns	Very low

PAL vs ZIP	0	No concerns	Suspected	No concerns	Major concerns	No concerns	No concerns	Very low
QUE vs RIS	0	Some concerns	Suspected	No concerns	Major concerns	No concerns	No concerns	Very low
QUE vs VALP	0	No concerns	Suspected	No concerns	No concerns	No concerns	No concerns	Low
QUE vs ZIP	0	Some concerns	Suspected	No concerns	Major concerns	No concerns	No concerns	Very low
RIS vs VALP	0	Some concerns	Suspected	No concerns	No concerns	No concerns	No concerns	Very low
RIS vs ZIP	0	Some concerns	Suspected	No concerns	Major concerns	No concerns	No concerns	Very low
VALP vs ZIP	0	Some concerns	Suspected	No concerns	Major concerns	No concerns	No concerns	Very low

Supplementary Appendix 15. Dizziness (N = 33, n = 8775)

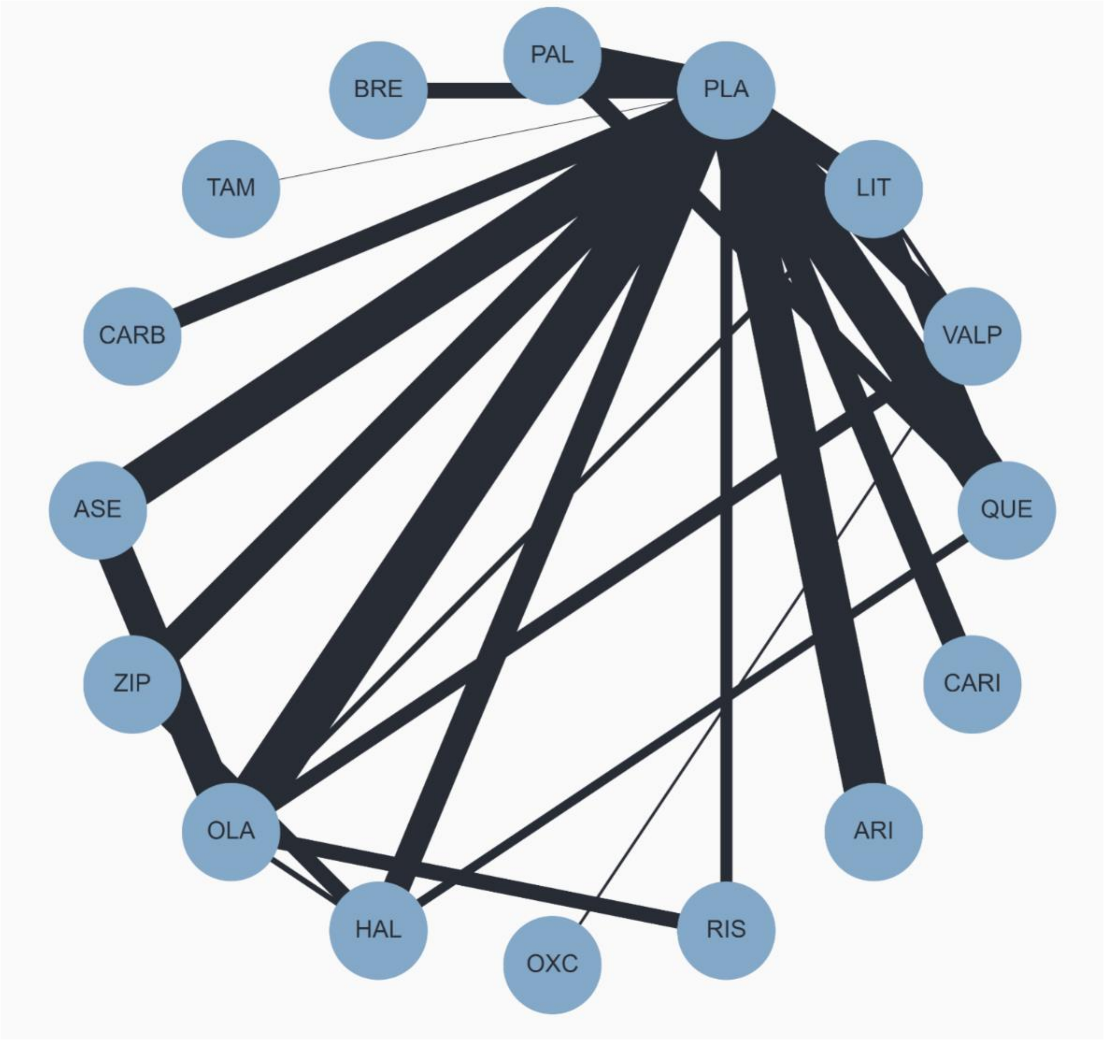

League table (risk ratio with 95% confidence interval)

ARI	**0.372 (0.195, 0.708)**	0.200 (0.022, 1.776)	**0.295 (0.165, 0.529)**	0.625 (0.287, 1.359)	0.400 (0.141, 1.133)	0.826 (0.411, 1.662)	**0.445 (0.250, 0.790)**	1.201 (0.296, 4.875)	0.581 (0.250, 1.350)	**0.345 (0.182, 0.656)**	0.654 (0.332, 1.288)	0.524 (0.056, 4.917)	**0.515 (0.283, 0.935)**	**0.412 (0.188, 0.903)**	1.048 (0.688, 1.597)
	ASE	0.538 (0.060, 4.847)	0.794 (0.421, 1.498)	1.681 (0.743, 3.802)	1.077 (0.374, 3.102)	**2.224 (1.086, 4.551)**	1.197 (0.783, 1.830)	3.232 (0.802, 13.015)	1.564 (0.654, 3.740)	0.929 (0.472, 1.828)	1.760 (0.941, 3.290)	1.410 (0.148, 13.415)	1.385 (0.777, 2.469)	1.108 (0.489, 2.514)	**2.820 (1.730, 4.599)**
		BRE	1.477 (0.167, 13.095)	3.126 (0.332, 29.409)	2.003 (0.192, 20.918)	4.136 (0.451, 37.909)	2.227 (0.252, 19.688)	6.012 (0.481, 75.196)	2.909 (0.302, 28.014)	1.729 (0.192, 15.576)	3.274 (0.360, 29.809)	2.623 (0.122, 56.583)	2.576 (0.290, 22.915)	2.062 (0.219, 19.445)	5.247 (0.615, 44.777)
			CARB	2.116 (0.981, 4.565)	1.356 (0.482, 3.816)	**2.800 (1.405, 5.577)**	1.507 (0.859, 2.646)	**4.069 (1.007, 16.440)**	1.969 (0.854, 4.537)	1.170 (0.622, 2.201)	**2.216 (1.137, 4.321)**	1.776 (0.190, 16.612)	1.744 (0.971, 3.132)	1.396 (0.642, 3.034)	**3.552 (2.369, 5.323)**
				CARI	0.641 (0.202, 2.034)	1.323 (0.560, 3.124)	0.712 (0.333, 1.525)	1.923 (0.434, 8.511)	0.930 (0.349, 2.479)	0.553 (0.245, 1.248)	1.047 (0.451, 2.431)	0.839 (0.085, 8.319)	0.824 (0.378, 1.795)	0.659 (0.260, 1.673)	1.678 (0.873, 3.227)
					HAL	2.065 (0.711, 5.995)	1.112 (0.405, 3.054)	3.001 (0.585, 15.385)	1.452 (0.458, 4.602)	0.863 (0.319, 2.338)	1.635 (0.555, 4.810)	1.310 (0.119, 14.384)	1.286 (0.458, 3.609)	1.029 (0.406, 2.608)	**2.620 (1.011, 6.788)**
						LIT	0.538 (0.284, 1.022)	1.453 (0.350, 6.034)	0.703 (0.311, 1.588)	**0.418 (0.242, 0.723)**	0.792 (0.374, 1.673)	0.634 (0.066, 6.131)	0.623 (0.326, 1.191)	0.498 (0.212, 1.170)	1.269 (0.726, 2.216)
							OLA	2.699 (0.701, 10.392)	1.306 (0.577, 2.959)	0.776 (0.425, 1.420)	1.470 (0.894, 2.418)	1.178 (0.126, 10.992)	1.157 (0.731, 1.831)	0.926 (0.432, 1.985)	**2.356 (1.594, 3.482)**

								OXC	0.484 (0.106, 2.203)	0.288 (0.070, 1.180)	0.545 (0.133, 2.235)	0.436 (0.033, 5.720)	0.429 (0.121, 1.522)	0.343 (0.077, 1.521)	0.873 (0.229, 3.321)
									PAL	0.594 (0.310, 1.138)	1.126 (0.459, 2.760)	0.902 (0.089, 9.150)	0.886 (0.386, 2.034)	0.709 (0.268, 1.872)	1.804 (0.869, 3.744)
										QUE	1.894 (0.932, 3.847)	1.517 (0.160, 14.420)	1.490 (0.801, 2.772)	1.192 (0.538, 2.645)	**3.035 (1.869, 4.927)**
											RIS	0.801 (0.083, 7.695)	0.787 (0.422, 1.466)	0.630 (0.270, 1.467)	1.603 (0.942, 2.725)
												TAM	0.982 (0.105, 9.218)	0.786 (0.079, 7.812)	2.000 (0.222, 18.031)
													VALP	0.800 (0.366, 1.750)	**2.037 (1.334, 3.110)**
														ZIP	**2.545 (1.312, 4.938)**
															PLA

Evaluation of heterogeneity and inconsistency

Between study variance (τ^2)	Heterogeneity assessment	Random-effects design-by-treatment interaction model		
		Q	df	p
0.009	Low	19.170	17	0.319

Incoherence

	NMA, RR (95% CI)	Direct, RR (95% CI)	I^2	Indirect, RR (95% CI)	Inconsistency measures	
					Difference of RR	P value
ARI vs PLA		1.048 (0.688, 1.597)	34.4%			
ASE vs OLA	1.197 (0.783, 1.830)	1.495 (0.935, 2.391)	0.0%	0.446 (0.166, 1.200)	3.353 (1.120, 10.037)	0.030
ASE vs PLA	2.820 (1.730, 4.599)	2.016 (1.053, 3.860)	60.0%	4.375 (2.082, 9.194)	0.461 (0.172, 1.236)	0.124
BRE vs PLA		5.247 (0.615, 44.777)	na			
CARB vs PLA		3.552 (2.369, 5.323)	0.0%			
CARI vs PLA		1.678 (0.873, 3.227)	0.0%			
HAL vs OLA	1.112 (0.405, 3.054)	0.396 (0.023, 6.797)	na	1.291 (0.438, 3.805)	0.307 (0.015, 6.422)	0.446
HAL vs QUE	0.863 (0.319, 2.338)	0.343 (0.070, 1.679)	na	1.573 (0.437, 5.658)	0.218 (0.028, 1.677)	0.144
HAL vs ZIP	1.029 (0.406, 2.608)	1.735 (0.542, 5.557)	na	0.411 (0.088, 1.925)	4.220 (0.610, 29.175)	0.144
HAL vs PLA	2.620 (1.011, 6.788)	3.723 (0.708, 19.562)	0.0%	2.204 (0.689, 7.051)	1.689 (0.223, 12.810)	0.612
LIT vs OLA	0.538 (0.284, 1.022)	0.486 (0.091, 2.593)	na	0.548 (0.274, 1.096)	0.887 (0.145, 5.433)	0.897
LIT vs QUE	0.418 (0.242, 0.723)	0.508 (0.260, 0.994)	0.0%	0.282 (0.109, 0.730)	1.801 (0.562, 5.766)	0.322
LIT vs VALP	0.623 (0.326, 1.191)	0.523 (0.154, 1.779)	na	0.667 (0.311, 1.430)	0.784 (0.185, 3.320)	0.741
LIT vs PLA	1.269 (0.726, 2.216)	1.297 (0.600, 2.802)	28.0%	1.238 (0.552, 2.779)	1.048 (0.343, 3.201)	0.935
OLA vs RIS	1.470 (0.894, 2.418)	1.270 (0.693, 2.328)	na	1.992 (0.832, 4.765)	0.638 (0.220, 1.845)	0.406
OLA vs VALP	1.157 (0.731, 1.831)	1.501 (0.798, 2.825)	56.2%	0.866 (0.444, 1.687)	1.734 (0.692, 4.347)	0.240
OLA vs PLA	2.356 (1.594, 3.482)	2.856 (1.607, 5.073)	0.0%	1.997 (1.173, 3.402)	1.430 (0.653, 3.130)	0.371

OXC vs VALP		0.429 (0.121, 1.522)	na			
PAL vs QUE	0.594 (0.310, 1.138)	0.516 (0.258, 1.033)	na	1.626 (0.255, 10.377)	0.318 (0.044, 2.299)	0.256
PAL vs PLA	1.804 (0.869, 3.744)	1.926 (0.770, 4.820)	62.9%	1.609 (0.481, 5.380)	1.197 (0.263, 5.450)	0.816
QUE vs PLA	3.035 (1.869, 4.927)	2.921 (1.647, 5.178)	0.0%	3.343 (1.346, 8.302)	0.874 (0.298, 2.560)	0.806
RIS vs PLA	1.603 (0.942, 2.725)	1.272 (0.594, 2.723)	na	1.995 (0.951, 4.185)	0.638 (0.220, 1.845)	0.406
TAM vs PLA		2.000 (0.222, 18.031)	na			
VALP vs PLA	2.037 (1.334, 3.110)	2.408 (1.425, 4.068)	0.0%	1.490 (0.727, 3.051)	1.616 (0.665, 3.929)	0.289
ZIP vs PLA	2.545 (1.312, 4.938)	3.115 (1.543, 6.286)	0.0%	0.491 (0.066, 3.646)	6.338 (0.758, 52.985)	0.088

Forest plot (vs placebo, the numbers are risk ratio with 95% confidence interval)

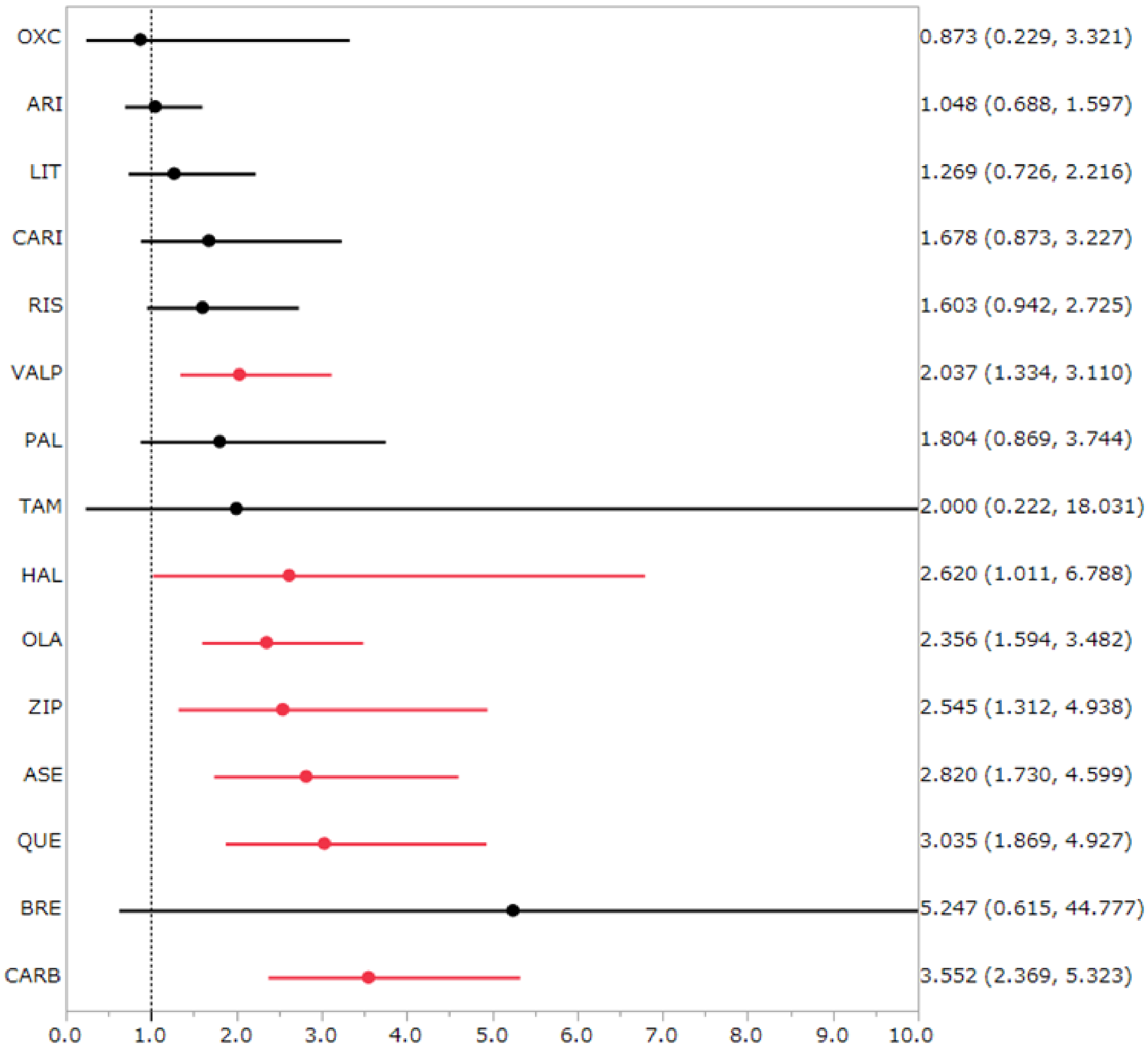

P-score

OXC	0.864
ARI	0.848
LIT	0.747
CARI	0.630
RIS	0.622
VALP	0.550
PAL	0.471
TAM	0.455
HAL	0.395
OLA	0.347
ZIP	0.332
ASE	0.293
QUE	0.243
BRE	0.192
CARB	0.123

Funnel plot (only double-blind, placebo-controlled trials)

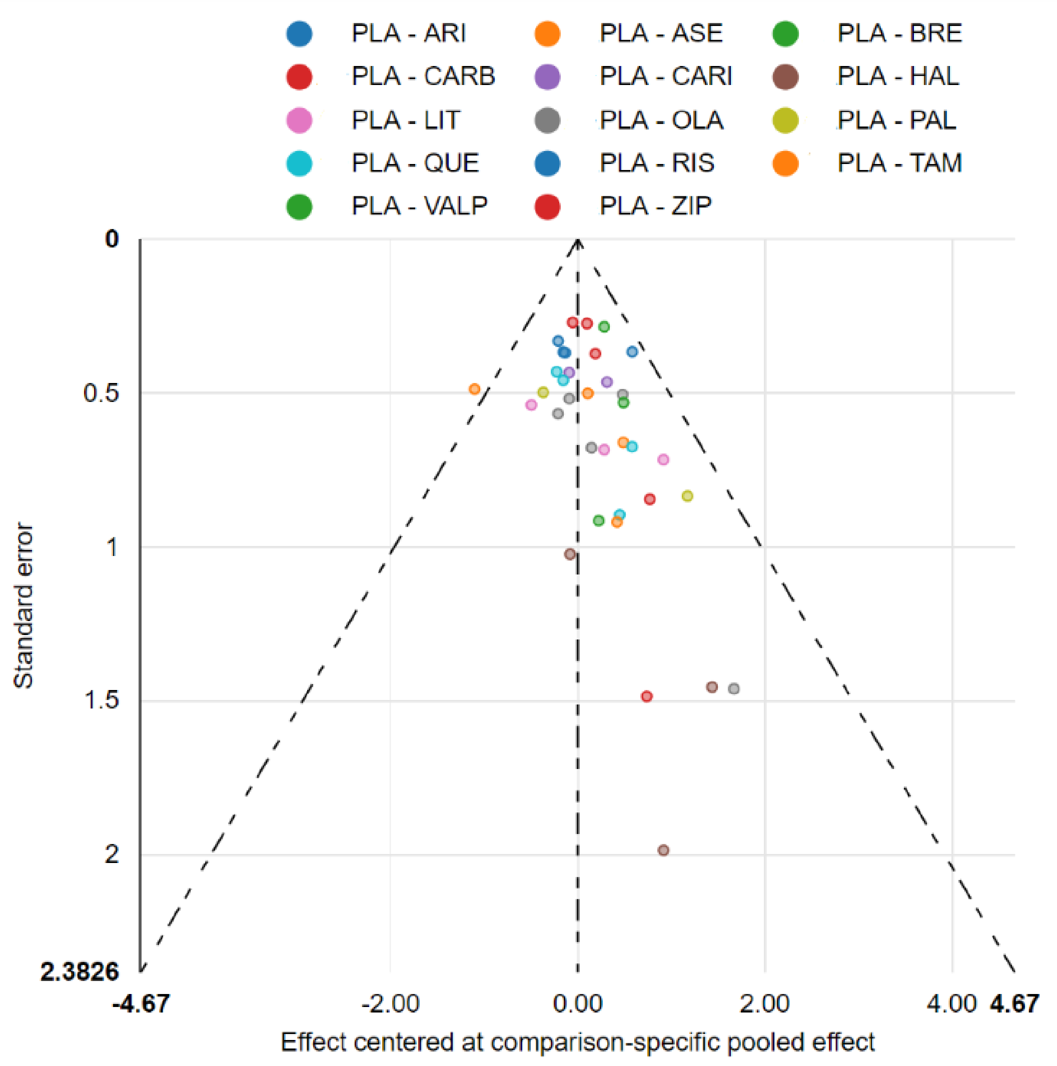

CINeMA confidence rating

Comparison	Number of studies	Within-study bias	Reporting bias	Indirectness	Imprecision	Heterogeneity	Incoherence	Confidence rating
ARI vs PLA	3	Some concerns	Suspected	No concerns	Major concerns	No concerns	No concerns	Very low
ASE vs OLA	2	Some concerns	Suspected	No concerns	Major concerns	No concerns	No concerns	Very low
ASE vs PLA	3	Some concerns	Suspected	No concerns	No concerns	No concerns	No concerns	Low
BRE vs PLA	1	Some concerns	Suspected	No concerns	Major concerns	No concerns	No concerns	Very low
CARB vs PLA	2	Some concerns	Suspected	No concerns	No concerns	No concerns	No concerns	Low
CARI vs PLA	2	Some concerns	Suspected	No concerns	Major concerns	No concerns	No concerns	Very low
HAL vs OLA	1	Some concerns	Suspected	No concerns	Major concerns	No concerns	No concerns	Very low
HAL vs QUE	1	Some concerns	Suspected	No concerns	Major concerns	No concerns	No concerns	Very low
HAL vs ZIP	1	Some concerns	Suspected	No concerns	Major concerns	No concerns	No concerns	Very low
HAL vs PLA	3	Some concerns	Suspected	No concerns	No concerns	Major concerns	No concerns	Very low
LIT vs OLA	1	Some concerns	Suspected	No concerns	Major concerns	No concerns	No concerns	Very low
LIT vs QUE	2	No concerns	Suspected	No concerns	No concerns	No concerns	No concerns	Moderate
LIT vs VALP	1	Some concerns	Suspected	No concerns	Major concerns	No concerns	No concerns	Very low
LIT vs PLA	3	Some concerns	Suspected	No concerns	Major concerns	No concerns	No concerns	Very low
OLA vs RIS	1	Some concerns	Suspected	No concerns	Major concerns	No concerns	No concerns	Very low
OLA vs VALP	2	No concerns	Suspected	No concerns	Major concerns	No concerns	No concerns	Low
OLA vs PLA	5	Some concerns	Suspected	No concerns	No concerns	No concerns	No concerns	Low
OXC vs VALP	1	Some concerns	Suspected	No concerns	Major concerns	No concerns	No concerns	Very low
PAL vs QUE	1	No concerns	Suspected	No concerns	Major concerns	No concerns	No concerns	Low
PAL vs PLA	2	No concerns	Suspected	No concerns	Major concerns	No concerns	No concerns	Low
QUE vs PLA	4	No concerns	Suspected	No concerns	No concerns	No concerns	No concerns	Moderate
RIS vs PLA	1	Some concerns	Suspected	No concerns	Major concerns	No concerns	No concerns	Very low
TAM vs PLA	1	No concerns	Suspected	No concerns	Major concerns	No concerns	No concerns	Low

VALP vs PLA	2	No concerns	Suspected	No concerns	No concerns	No concerns	No concerns	Moderate
ZIP vs PLA	3	Some concerns	Suspected	No concerns	No concerns	No concerns	Major concerns	Very low
ARI vs ASE	0	Some concerns	Suspected	No concerns	No concerns	No concerns	No concerns	Very low
ARI vs BRE	0	Some concerns	Suspected	No concerns	Major concerns	No concerns	No concerns	Very low
ARI vs CARB	0	Some concerns	Suspected	No concerns	No concerns	No concerns	No concerns	Very low
ARI vs CARI	0	Some concerns	Suspected	No concerns	Major concerns	No concerns	No concerns	Very low
ARI vs HAL	0	Some concerns	Suspected	No concerns	Major concerns	No concerns	No concerns	Very low
ARI vs LIT	0	Some concerns	Suspected	No concerns	Major concerns	No concerns	No concerns	Very low
ARI vs OLA	0	Some concerns	Suspected	No concerns	No concerns	No concerns	No concerns	Very low
ARI vs OXC	0	Some concerns	Suspected	No concerns	Major concerns	No concerns	No concerns	Very low
ARI vs PAL	0	No concerns	Suspected	No concerns	Major concerns	No concerns	No concerns	Very low
ARI vs QUE	0	Some concerns	Suspected	No concerns	No concerns	No concerns	No concerns	Very low
ARI vs RIS	0	Some concerns	Suspected	No concerns	Major concerns	No concerns	No concerns	Very low
ARI vs TAM	0	Some concerns	Suspected	No concerns	Major concerns	No concerns	No concerns	Very low
ARI vs VALP	0	Some concerns	Suspected	No concerns	No concerns	No concerns	No concerns	Very low
ARI vs ZIP	0	Some concerns	Suspected	No concerns	No concerns	No concerns	No concerns	Very low
ASE vs BRE	0	Some concerns	Suspected	No concerns	Major concerns	No concerns	No concerns	Very low
ASE vs CARB	0	Some concerns	Suspected	No concerns	Major concerns	No concerns	No concerns	Very low
ASE vs CARI	0	Some concerns	Suspected	No concerns	Major concerns	No concerns	No concerns	Very low
ASE vs HAL	0	Some concerns	Suspected	No concerns	Major concerns	No concerns	No concerns	Very low
ASE vs LIT	0	Some concerns	Suspected	No concerns	No concerns	No concerns	No concerns	Very low
ASE vs OXC	0	Some concerns	Suspected	No concerns	Major concerns	No concerns	No concerns	Very low
ASE vs PAL	0	No concerns	Suspected	No concerns	Major concerns	No concerns	No concerns	Very low
ASE vs QUE	0	No concerns	Suspected	No concerns	Major concerns	No concerns	No concerns	Very low
ASE vs RIS	0	Some concerns	Suspected	No concerns	Major concerns	No concerns	No concerns	Very low

ASE vs TAM	0	No concerns	Suspected	No concerns	Major concerns	No concerns	No concerns	Very low
ASE vs VALP	0	No concerns	Suspected	No concerns	Major concerns	No concerns	No concerns	Very low
ASE vs ZIP	0	Some concerns	Suspected	No concerns	Major concerns	No concerns	No concerns	Very low
BRE vs CARB	0	Some concerns	Suspected	No concerns	Major concerns	No concerns	No concerns	Very low
BRE vs CARI	0	Some concerns	Suspected	No concerns	Major concerns	No concerns	No concerns	Very low
BRE vs HAL	0	Some concerns	Suspected	No concerns	Major concerns	No concerns	No concerns	Very low
BRE vs LIT	0	Some concerns	Suspected	No concerns	Major concerns	No concerns	No concerns	Very low
BRE vs OLA	0	Some concerns	Suspected	No concerns	Major concerns	No concerns	No concerns	Very low
BRE vs OXC	0	Some concerns	Suspected	No concerns	Major concerns	No concerns	No concerns	Very low
BRE vs PAL	0	No concerns	Suspected	No concerns	Major concerns	No concerns	No concerns	Very low
BRE vs QUE	0	Some concerns	Suspected	No concerns	Major concerns	No concerns	No concerns	Very low
BRE vs RIS	0	Some concerns	Suspected	No concerns	Major concerns	No concerns	No concerns	Very low
BRE vs TAM	0	Some concerns	Suspected	No concerns	Major concerns	No concerns	No concerns	Very low
BRE vs VALP	0	Some concerns	Suspected	No concerns	Major concerns	No concerns	No concerns	Very low
BRE vs ZIP	0	Some concerns	Suspected	No concerns	Major concerns	No concerns	No concerns	Very low
CARB vs CARI	0	Some concerns	Suspected	No concerns	Major concerns	No concerns	No concerns	Very low
CARB vs HAL	0	Some concerns	Suspected	No concerns	Major concerns	No concerns	No concerns	Very low
CARB vs LIT	0	Some concerns	Suspected	No concerns	No concerns	No concerns	No concerns	Very low
CARB vs OLA	0	Some concerns	Suspected	No concerns	Major concerns	No concerns	No concerns	Very low
CARB vs OXC	0	Some concerns	Suspected	No concerns	No concerns	Major concerns	No concerns	Very low
CARB vs PAL	0	No concerns	Suspected	No concerns	Major concerns	No concerns	No concerns	Very low
CARB vs QUE	0	Some concerns	Suspected	No concerns	Major concerns	No concerns	No concerns	Very low
CARB vs RIS	0	Some concerns	Suspected	No concerns	No concerns	No concerns	No concerns	Very low
CARB vs TAM	0	Some concerns	Suspected	No concerns	Major concerns	No concerns	No concerns	Very low
CARB vs VALP	0	Some concerns	Suspected	No concerns	Major concerns	No concerns	No concerns	Very low

CARB vs ZIP	0	Some concerns	Suspected	No concerns	Major concerns	No concerns	No concerns	Very low
CARI vs HAL	0	Some concerns	Suspected	No concerns	Major concerns	No concerns	No concerns	Very low
CARI vs LIT	0	Some concerns	Suspected	No concerns	Major concerns	No concerns	No concerns	Very low
CARI vs OLA	0	Some concerns	Suspected	No concerns	Major concerns	No concerns	No concerns	Very low
CARI vs OXC	0	Some concerns	Suspected	No concerns	Major concerns	No concerns	No concerns	Very low
CARI vs PAL	0	No concerns	Suspected	No concerns	Major concerns	No concerns	No concerns	Very low
CARI vs QUE	0	Some concerns	Suspected	No concerns	Major concerns	No concerns	No concerns	Very low
CARI vs RIS	0	Some concerns	Suspected	No concerns	Major concerns	No concerns	No concerns	Very low
CARI vs TAM	0	Some concerns	Suspected	No concerns	Major concerns	No concerns	No concerns	Very low
CARI vs VALP	0	Some concerns	Suspected	No concerns	Major concerns	No concerns	No concerns	Very low
CARI vs ZIP	0	Some concerns	Suspected	No concerns	Major concerns	No concerns	No concerns	Very low
HAL vs LIT	0	Some concerns	Suspected	No concerns	Major concerns	No concerns	No concerns	Very low
HAL vs OXC	0	Some concerns	Suspected	No concerns	Major concerns	No concerns	No concerns	Very low
HAL vs PAL	0	Some concerns	Suspected	No concerns	Major concerns	No concerns	No concerns	Very low
HAL vs RIS	0	Some concerns	Suspected	No concerns	Major concerns	No concerns	No concerns	Very low
HAL vs TAM	0	Some concerns	Suspected	No concerns	Major concerns	No concerns	No concerns	Very low
HAL vs VALP	0	Some concerns	Suspected	No concerns	Major concerns	No concerns	No concerns	Very low
LIT vs OXC	0	Some concerns	Suspected	No concerns	Major concerns	No concerns	No concerns	Very low
LIT vs PAL	0	No concerns	Suspected	No concerns	Major concerns	No concerns	No concerns	Very low
LIT vs RIS	0	Some concerns	Suspected	No concerns	Major concerns	No concerns	No concerns	Very low
LIT vs TAM	0	No concerns	Suspected	No concerns	Major concerns	No concerns	No concerns	Very low
LIT vs ZIP	0	Some concerns	Suspected	No concerns	Major concerns	No concerns	No concerns	Very low
OLA vs OXC	0	Some concerns	Suspected	No concerns	Major concerns	No concerns	No concerns	Very low
OLA vs PAL	0	No concerns	Suspected	No concerns	Major concerns	No concerns	No concerns	Very low
OLA vs QUE	0	No concerns	Suspected	No concerns	Major concerns	No concerns	No concerns	Very low

OLA vs TAM	0	No concerns	Suspected	No concerns	Major concerns	No concerns	No concerns	Very low
OLA vs ZIP	0	Some concerns	Suspected	No concerns	Major concerns	No concerns	No concerns	Very low
OXC vs PAL	0	No concerns	Suspected	No concerns	Major concerns	No concerns	No concerns	Very low
OXC vs QUE	0	Some concerns	Suspected	No concerns	Major concerns	No concerns	No concerns	Very low
OXC vs RIS	0	Some concerns	Suspected	No concerns	Major concerns	No concerns	No concerns	Very low
OXC vs TAM	0	No concerns	Suspected	No concerns	Major concerns	No concerns	No concerns	Very low
OXC vs ZIP	0	Some concerns	Suspected	No concerns	Major concerns	No concerns	No concerns	Very low
OXC vs PLA	0	Some concerns	Suspected	No concerns	Major concerns	No concerns	No concerns	Very low
PAL vs RIS	0	No concerns	Suspected	No concerns	Major concerns	No concerns	No concerns	Very low
PAL vs TAM	0	No concerns	Suspected	No concerns	Major concerns	No concerns	No concerns	Very low
PAL vs VALP	0	No concerns	Suspected	No concerns	Major concerns	No concerns	No concerns	Very low
PAL vs ZIP	0	No concerns	Suspected	No concerns	Major concerns	No concerns	No concerns	Very low
QUE vs RIS	0	Some concerns	Suspected	No concerns	Major concerns	No concerns	No concerns	Very low
QUE vs TAM	0	No concerns	Suspected	No concerns	Major concerns	No concerns	No concerns	Very low
QUE vs VALP	0	No concerns	Suspected	No concerns	Major concerns	No concerns	No concerns	Very low
QUE vs ZIP	0	Some concerns	Suspected	No concerns	Major concerns	No concerns	No concerns	Very low
RIS vs TAM	0	Some concerns	Suspected	No concerns	Major concerns	No concerns	No concerns	Very low
RIS vs VALP	0	Some concerns	Suspected	No concerns	Major concerns	No concerns	No concerns	Very low
RIS vs ZIP	0	Some concerns	Suspected	No concerns	Major concerns	No concerns	No concerns	Very low
TAM vs VALP	0	No concerns	Suspected	No concerns	Major concerns	No concerns	No concerns	Very low
TAM vs ZIP	0	No concerns	Suspected	No concerns	Major concerns	No concerns	No concerns	Very low
VALP vs ZIP	0	Some concerns	Suspected	No concerns	Major concerns	No concerns	No concerns	Very low

Supplementary Appendix 16. Headache (N = 37, n = 10330)

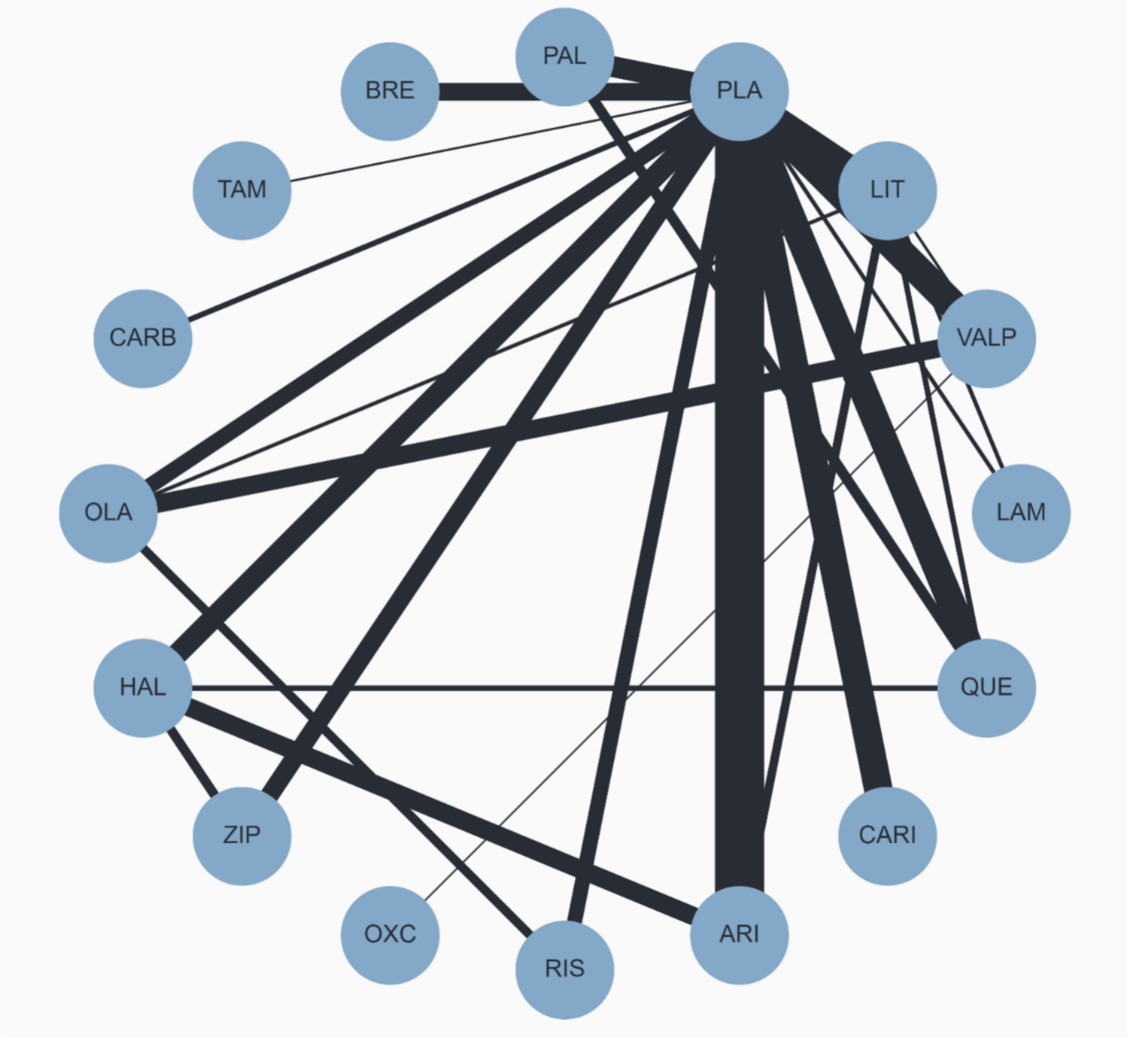

League table (risk ratio with 95% confidence interval)

ARI	1.835 (0.993, 3.390)	1.125 (0.666, 1.900)	0.992 (0.686, 1.434)	1.146 (0.775, 1.695)	0.479 (0.157, 1.461)	0.954 (0.707, 1.286)	1.272 (0.894, 1.810)	2.328 (0.620, 8.742)	0.944 (0.627, 1.423)	1.103 (0.759, 1.604)	1.001 (0.646, 1.552)	1.472 (0.559, 3.879)	1.164 (0.868, 1.560)	0.785 (0.508, 1.213)	1.056 (0.890, 1.252)
	BRE	0.613 (0.284, 1.325)	0.541 (0.276, 1.061)	0.625 (0.307, 1.270)	**0.261 (0.075, 0.914)**	**0.520 (0.271, 0.997)**	0.693 (0.356, 1.350)	1.269 (0.301, 5.350)	0.515 (0.256, 1.035)	0.601 (0.304, 1.187)	0.546 (0.267, 1.116)	0.803 (0.262, 2.463)	0.635 (0.336, 1.200)	**0.428 (0.209, 0.875)**	0.575 (0.319, 1.038)
		CARB	0.882 (0.487, 1.596)	1.019 (0.541, 1.918)	0.426 (0.127, 1.430)	0.848 (0.481, 1.494)	1.130 (0.630, 2.028)	2.069 (0.509, 8.415)	0.839 (0.451, 1.562)	0.980 (0.538, 1.787)	0.890 (0.469, 1.687)	1.309 (0.447, 3.833)	1.035 (0.596, 1.795)	0.698 (0.368, 1.324)	0.938 (0.572, 1.540)
			CARI	1.155 (0.693, 1.927)	0.483 (0.152, 1.529)	0.961 (0.627, 1.474)	1.282 (0.817, 2.011)	2.346 (0.607, 9.074)	0.952 (0.579, 1.565)	1.111 (0.694, 1.781)	1.009 (0.600, 1.697)	1.484 (0.542, 4.066)	1.173 (0.782, 1.761)	0.791 (0.470, 1.332)	1.064 (0.767, 1.475)
				HAL	0.418 (0.129, 1.349)	0.832 (0.520, 1.331)	1.109 (0.673, 1.830)	2.031 (0.516, 7.992)	0.824 (0.482, 1.407)	0.962 (0.586, 1.580)	0.873 (0.497, 1.536)	1.284 (0.458, 3.604)	1.015 (0.641, 1.610)	0.685 (0.406, 1.157)	0.921 (0.621, 1.365)
					LAM	1.991 (0.659, 6.015)	2.655 (0.844, 8.355)	4.860 (0.875, 26.986)	1.971 (0.614, 6.326)	2.302 (0.727, 7.287)	2.090 (0.644, 6.778)	3.074 (0.714, 13.237)	2.430 (0.786, 7.513)	1.639 (0.505, 5.321)	2.204 (0.729, 6.659)
						LIT	1.334 (0.890, 1.997)	2.441 (0.642, 9.286)	0.990 (0.627, 1.564)	1.156 (0.765, 1.748)	1.050 (0.646, 1.705)	1.544 (0.572, 4.166)	1.221 (0.863, 1.727)	0.823 (0.505, 1.342)	1.107 (0.840, 1.458)
							OLA	1.831 (0.485, 6.901)	0.742 (0.457, 1.207)	0.867 (0.548, 1.371)	0.787 (0.524, 1.184)	1.158 (0.425, 3.155)	0.915 (0.671, 1.249)	0.617 (0.371, 1.028)	0.830 (0.609, 1.132)

								OXC	0.406 (0.104, 1.588)	0.474 (0.122, 1.837)	0.430 (0.110, 1.681)	0.632 (0.125, 3.203)	0.500 (0.138, 1.817)	0.337 (0.085, 1.332)	0.453 (0.122, 1.685)
									PAL	1.168 (0.771, 1.769)	1.060 (0.611, 1.840)	1.559 (0.560, 4.344)	1.233 (0.790, 1.924)	0.832 (0.479, 1.443)	1.118 (0.768, 1.627)
										QUE	0.908 (0.536, 1.539)	1.335 (0.485, 3.674)	1.055 (0.698, 1.597)	0.712 (0.421, 1.204)	0.957 (0.682, 1.344)
											RIS	1.471 (0.522, 4.143)	1.163 (0.749, 1.805)	0.784 (0.442, 1.390)	1.054 (0.704, 1.580)
												TAM	0.791 (0.296, 2.114)	0.533 (0.189, 1.503)	0.717 (0.276, 1.861)
													VALP	0.675 (0.421, 1.081)	0.907 (0.713, 1.154)
														ZIP	1.344 (0.896, 2.017)
															PLA

Evaluation of heterogeneity and inconsistency

Between study variance (τ^2)	Heterogeneity assessment	Random-effects design-by-treatment interaction model		
		Q	df	p
0.000	Low	17.976	19	0.524

Incoherence

	NMA, RR (95% CI)	Direct, RR (95% CI)	I^2	Indirect, RR (95% CI)	Inconsistency measures	
					Difference of RR	P value
ARI vs HAL	1.146 (0.775, 1.695)	1.037 (0.635, 1.696)	0.0%	1.362 (0.714, 2.597)	0.762 (0.338, 1.715)	0.511
ARI vs LIT	0.954 (0.707, 1.286)	1.161 (0.762, 1.770)	na	0.781 (0.512, 1.194)	1.486 (0.818, 2.702)	0.194
ARI vs PLA	1.056 (0.890, 1.252)	1.045 (0.876, 1.246)	0.0%	1.242 (0.622, 2.481)	0.841 (0.412, 1.717)	0.634
BRE vs PLA		0.575 (0.319, 1.038)	0.0%			
CARB vs PLA		0.938 (0.572, 1.540)	na			
CARI vs PLA		1.064 (0.767, 1.475)	0.0%			
HAL vs QUE	0.962 (0.586, 1.580)	1.649 (0.558, 4.867)	na	0.834 (0.477, 1.457)	1.977 (0.585, 6.683)	0.273
HAL vs ZIP	0.685 (0.406, 1.157)	0.367 (0.148, 0.910)	na	0.936 (0.493, 1.779)	0.392 (0.129, 1.192)	0.099
HAL vs PLA	0.921 (0.621, 1.365)	0.713 (0.401, 1.268)	56.4%	1.153 (0.672, 1.978)	0.618 (0.281, 1.362)	0.233
LAM vs LIT	1.991 (0.659, 6.015)	2.108 (0.547, 8.123)	na	1.770 (0.257, 12.208)	1.191 (0.113, 12.552)	0.884
LAM vs PLA	2.204 (0.729, 6.659)	2.081 (0.540, 8.017)	na	2.478 (0.359, 17.099)	0.840 (0.080, 8.857)	0.884
LIT vs OLA	1.334 (0.890, 1.997)	0.486 (0.045, 5.237)	na	1.374 (0.912, 2.071)	0.354 (0.032, 3.947)	0.398
LIT vs QUE	1.156 (0.765, 1.748)	1.638 (0.699, 3.838)	na	1.039 (0.648, 1.666)	1.576 (0.595, 4.174)	0.360
LIT vs VALP	1.221 (0.863, 1.727)	1.789 (0.975, 3.281)	na	1.013 (0.664, 1.547)	1.765 (0.842, 3.698)	0.132
LIT vs PLA	1.107 (0.840, 1.458)	1.090 (0.801, 1.484)	29.0%	1.176 (0.634, 2.181)	0.927 (0.465, 1.848)	0.829
OLA vs RIS	0.787 (0.524, 1.184)	0.835 (0.487, 1.430)	na	0.727 (0.390, 1.358)	1.148 (0.503, 2.618)	0.743
OLA vs VALP	0.915 (0.671, 1.249)	0.806 (0.544, 1.195)	60.7%	1.129 (0.680, 1.874)	0.714 (0.376, 1.356)	0.303

OLA vs PLA	0.830 (0.609, 1.132)	0.800 (0.508, 1.258)	0.0%	0.857 (0.561, 1.312)	0.933 (0.501, 1.736)	0.827
OXC vs VALP		0.500 (0.138, 1.817)	na			
PAL vs QUE	1.168 (0.771, 1.769)	0.950 (0.563, 1.604)	na	1.658 (0.838, 3.280)	0.573 (0.243, 1.354)	0.204
PAL vs PLA	1.118 (0.768, 1.627)	1.210 (0.807, 1.814)	0.0%	0.695 (0.258, 1.876)	1.740 (0.596, 5.085)	0.311
QUE vs PLA	0.957 (0.682, 1.344)	1.040 (0.710, 1.525)	0.0%	0.701 (0.335, 1.467)	1.484 (0.646, 3.409)	0.352
RIS vs PLA	1.054 (0.704, 1.580)	1.115 (0.660, 1.882)	41.8%	0.971 (0.514, 1.836)	1.148 (0.503, 2.618)	0.743
TAM vs PLA		0.717 (0.276, 1.861)	0.0%			
VALP vs PLA	0.907 (0.713, 1.154)	0.894 (0.686, 1.165)	0.0%	0.972 (0.544, 1.735)	0.920 (0.486, 1.740)	0.797
ZIP vs PLA	1.344 (0.896, 2.017)	1.201 (0.787, 1.833)	0.0%	4.879 (1.169, 20.369)	0.246 (0.055, 1.092)	0.065

Forest plot (vs placebo, the numbers are risk ratio with 95% confidence interval)

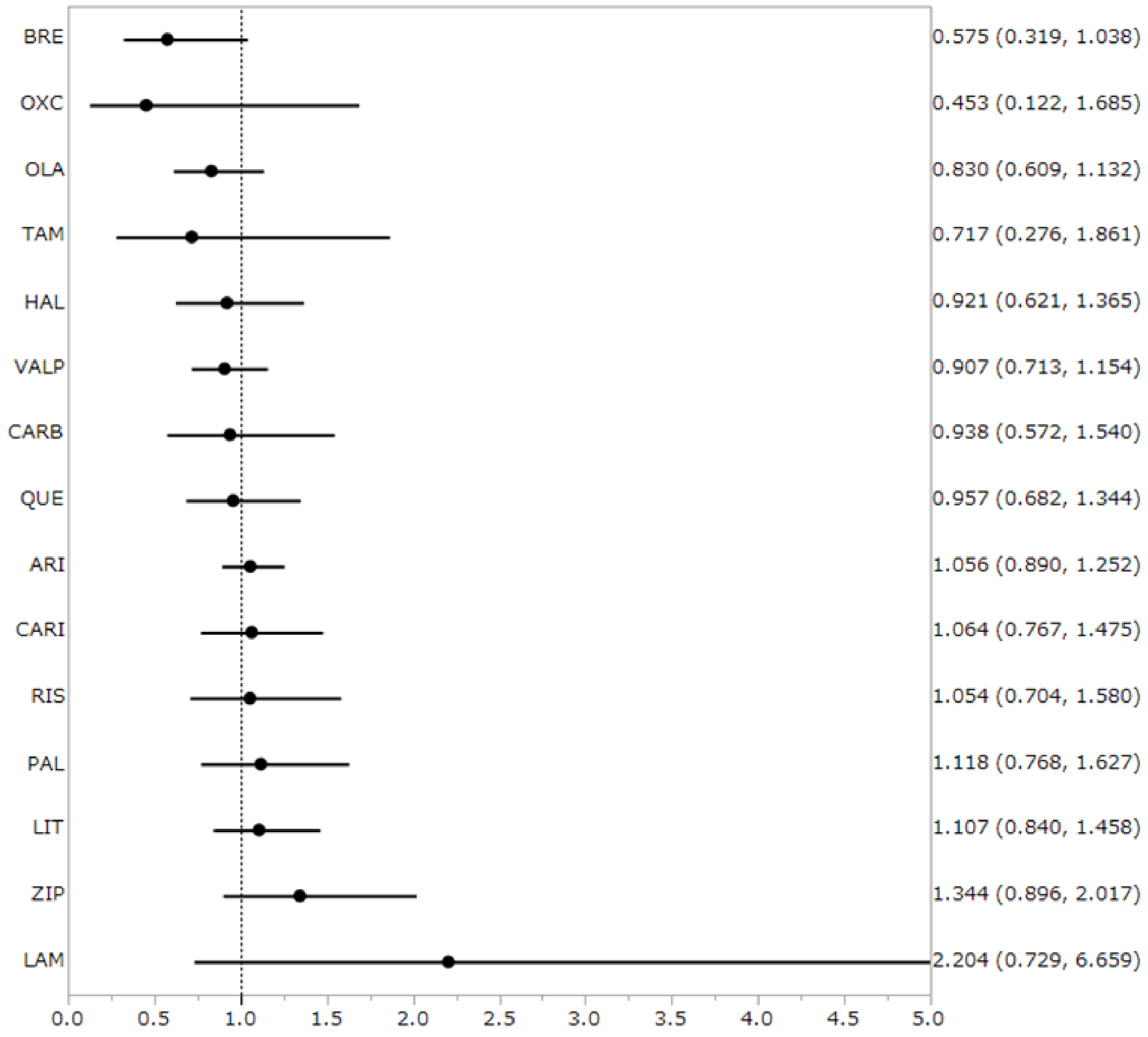

P-score

BRE	0.874
OXC	0.864
OLA	0.730
TAM	0.664
HAL	0.611
VALP	0.606
CARB	0.549
QUE	0.514
ARI	0.406
CARI	0.395
RIS	0.394
PAL	0.329
LIT	0.328
ZIP	0.155
LAM	0.089

Funnel plot (only double-blind, placebo-controlled trials)

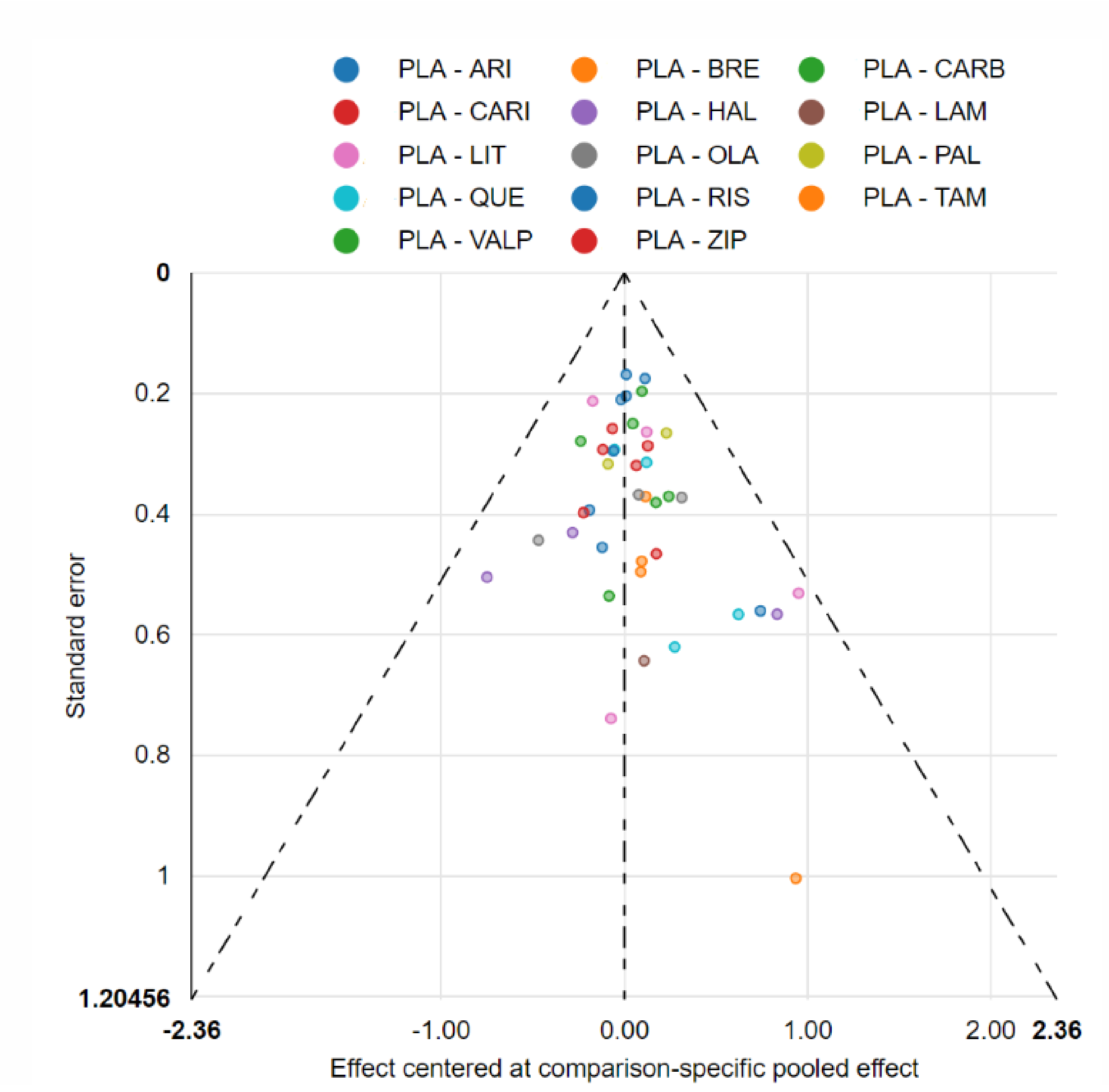

CINeMA confidence rating

Comparison	Number of studies	Within-study bias	Reporting bias	Indirectness	Imprecision	Heterogeneity	Incoherence	Confidence rating
ARI vs HAL	2	Some concerns	Suspected	No concerns	Major concerns	No concerns	No concerns	Very low
ARI vs LIT	1	Some concerns	Suspected	No concerns	Major concerns	No concerns	No concerns	Very low
ARI vs PLA	6	Some concerns	Suspected	No concerns	Major concerns	No concerns	No concerns	Very low
BRE vs PLA	2	Some concerns	Suspected	No concerns	Major concerns	No concerns	No concerns	Very low
CARB vs PLA	1	Some concerns	Suspected	No concerns	Major concerns	No concerns	No concerns	Very low
CARI vs PLA	3	Some concerns	Suspected	No concerns	Major concerns	No concerns	No concerns	Very low
HAL vs QUE	1	Some concerns	Suspected	No concerns	Major concerns	No concerns	No concerns	Very low
HAL vs ZIP	1	Some concerns	Suspected	No concerns	Major concerns	No concerns	Major concerns	Very low
HAL vs PLA	3	Some concerns	Suspected	No concerns	Major concerns	No concerns	No concerns	Very low
LAM vs LIT	1	Some concerns	Suspected	No concerns	Major concerns	No concerns	No concerns	Very low
LAM vs PLA	1	Some concerns	Suspected	No concerns	Major concerns	No concerns	No concerns	Very low
LIT vs OLA	1	Some concerns	Suspected	No concerns	Major concerns	No concerns	No concerns	Very low
LIT vs QUE	1	No concerns	Suspected	No concerns	Major concerns	No concerns	No concerns	Low
LIT vs VALP	1	Some concerns	Suspected	No concerns	Major concerns	No concerns	No concerns	Very low
LIT vs PLA	4	Some concerns	Suspected	No concerns	Major concerns	No concerns	No concerns	Very low
OLA vs RIS	1	Some concerns	Suspected	No concerns	Major concerns	No concerns	No concerns	Very low
OLA vs VALP	2	No concerns	Suspected	No concerns	Major concerns	No concerns	No concerns	Low
OLA vs PLA	3	Some concerns	Suspected	No concerns	Major concerns	No concerns	No concerns	Very low
OXC vs VALP	1	Some concerns	Suspected	No concerns	Major concerns	No concerns	No concerns	Very low
PAL vs QUE	1	No concerns	Suspected	No concerns	Major concerns	No concerns	No concerns	Low
PAL vs PLA	2	No concerns	Suspected	No concerns	Major concerns	No concerns	No concerns	Low
QUE vs PLA	4	No concerns	Suspected	No concerns	Major concerns	No concerns	No concerns	Low
RIS vs PLA	2	Some concerns	Suspected	No concerns	Major concerns	No concerns	No concerns	Very low

TAM vs PLA	2	No concerns	Suspected	No concerns	Major concerns	No concerns	No concerns	Low
VALP vs PLA	5	No concerns	Suspected	No concerns	Major concerns	No concerns	No concerns	Low
ZIP vs PLA	3	Some concerns	Suspected	No concerns	Major concerns	No concerns	Major concerns	Very low
ARI vs BRE	0	Some concerns	Suspected	No concerns	Major concerns	No concerns	No concerns	Very low
ARI vs CARB	0	Some concerns	Suspected	No concerns	Major concerns	No concerns	No concerns	Very low
ARI vs CARI	0	Some concerns	Suspected	No concerns	Major concerns	No concerns	No concerns	Very low
ARI vs LAM	0	Some concerns	Suspected	No concerns	Major concerns	No concerns	No concerns	Very low
ARI vs OLA	0	Some concerns	Suspected	No concerns	Major concerns	No concerns	No concerns	Very low
ARI vs OXC	0	Some concerns	Suspected	No concerns	Major concerns	No concerns	No concerns	Very low
ARI vs PAL	0	No concerns	Suspected	No concerns	Major concerns	No concerns	No concerns	Very low
ARI vs QUE	0	Some concerns	Suspected	No concerns	Major concerns	No concerns	No concerns	Very low
ARI vs RIS	0	Some concerns	Suspected	No concerns	Major concerns	No concerns	No concerns	Very low
ARI vs TAM	0	Some concerns	Suspected	No concerns	Major concerns	No concerns	No concerns	Very low
ARI vs VALP	0	Some concerns	Suspected	No concerns	Major concerns	No concerns	No concerns	Very low
ARI vs ZIP	0	Some concerns	Suspected	No concerns	Major concerns	No concerns	No concerns	Very low
BRE vs CARB	0	Some concerns	Suspected	No concerns	Major concerns	No concerns	No concerns	Very low
BRE vs CARI	0	Some concerns	Suspected	No concerns	Major concerns	No concerns	No concerns	Very low
BRE vs HAL	0	Some concerns	Suspected	No concerns	Major concerns	No concerns	No concerns	Very low
BRE vs LAM	0	Some concerns	Suspected	No concerns	No concerns	No concerns	No concerns	Very low
BRE vs LIT	0	Some concerns	Suspected	No concerns	No concerns	Major concerns	No concerns	Very low
BRE vs OLA	0	Some concerns	Suspected	No concerns	Major concerns	No concerns	No concerns	Very low
BRE vs OXC	0	Some concerns	Suspected	No concerns	Major concerns	No concerns	No concerns	Very low
BRE vs PAL	0	No concerns	Suspected	No concerns	Major concerns	No concerns	No concerns	Very low
BRE vs QUE	0	Some concerns	Suspected	No concerns	Major concerns	No concerns	No concerns	Very low
BRE vs RIS	0	Some concerns	Suspected	No concerns	Major concerns	No concerns	No concerns	Very low

BRE vs TAM	0	Some concerns	Suspected	No concerns	Major concerns	No concerns	No concerns	Very low
BRE vs VALP	0	Some concerns	Suspected	No concerns	Major concerns	No concerns	No concerns	Very low
BRE vs ZIP	0	Some concerns	Suspected	No concerns	No concerns	No concerns	No concerns	Very low
CARB vs CARI	0	Some concerns	Suspected	No concerns	Major concerns	No concerns	No concerns	Very low
CARB vs HAL	0	Some concerns	Suspected	No concerns	Major concerns	No concerns	No concerns	Very low
CARB vs LAM	0	Some concerns	Suspected	No concerns	Major concerns	No concerns	No concerns	Very low
CARB vs LIT	0	Some concerns	Suspected	No concerns	Major concerns	No concerns	No concerns	Very low
CARB vs OLA	0	Some concerns	Suspected	No concerns	Major concerns	No concerns	No concerns	Very low
CARB vs OXC	0	Some concerns	Suspected	No concerns	Major concerns	No concerns	No concerns	Very low
CARB vs PAL	0	No concerns	Suspected	No concerns	Major concerns	No concerns	No concerns	Very low
CARB vs QUE	0	Some concerns	Suspected	No concerns	Major concerns	No concerns	No concerns	Very low
CARB vs RIS	0	Some concerns	Suspected	No concerns	Major concerns	No concerns	No concerns	Very low
CARB vs TAM	0	Some concerns	Suspected	No concerns	Major concerns	No concerns	No concerns	Very low
CARB vs VALP	0	Some concerns	Suspected	No concerns	Major concerns	No concerns	No concerns	Very low
CARB vs ZIP	0	Some concerns	Suspected	No concerns	Major concerns	No concerns	No concerns	Very low
CARI vs HAL	0	Some concerns	Suspected	No concerns	Major concerns	No concerns	No concerns	Very low
CARI vs LAM	0	Some concerns	Suspected	No concerns	Major concerns	No concerns	No concerns	Very low
CARI vs LIT	0	Some concerns	Suspected	No concerns	Major concerns	No concerns	No concerns	Very low
CARI vs OLA	0	Some concerns	Suspected	No concerns	Major concerns	No concerns	No concerns	Very low
CARI vs OXC	0	Some concerns	Suspected	No concerns	Major concerns	No concerns	No concerns	Very low
CARI vs PAL	0	No concerns	Suspected	No concerns	Major concerns	No concerns	No concerns	Very low
CARI vs QUE	0	Some concerns	Suspected	No concerns	Major concerns	No concerns	No concerns	Very low
CARI vs RIS	0	Some concerns	Suspected	No concerns	Major concerns	No concerns	No concerns	Very low
CARI vs TAM	0	Some concerns	Suspected	No concerns	Major concerns	No concerns	No concerns	Very low
CARI vs VALP	0	Some concerns	Suspected	No concerns	Major concerns	No concerns	No concerns	Very low

CARI vs ZIP	0	Some concerns	Suspected	No concerns	Major concerns	No concerns	No concerns	Very low
HAL vs LAM	0	Some concerns	Suspected	No concerns	Major concerns	No concerns	No concerns	Very low
HAL vs LIT	0	Some concerns	Suspected	No concerns	Major concerns	No concerns	No concerns	Very low
HAL vs OLA	0	Some concerns	Suspected	No concerns	Major concerns	No concerns	No concerns	Very low
HAL vs OXC	0	Some concerns	Suspected	No concerns	Major concerns	No concerns	No concerns	Very low
HAL vs PAL	0	Some concerns	Suspected	No concerns	Major concerns	No concerns	No concerns	Very low
HAL vs RIS	0	Some concerns	Suspected	No concerns	Major concerns	No concerns	No concerns	Very low
HAL vs TAM	0	Some concerns	Suspected	No concerns	Major concerns	No concerns	No concerns	Very low
HAL vs VALP	0	Some concerns	Suspected	No concerns	Major concerns	No concerns	No concerns	Very low
LAM vs OLA	0	Some concerns	Suspected	No concerns	Major concerns	No concerns	No concerns	Very low
LAM vs OXC	0	Some concerns	Suspected	No concerns	Major concerns	No concerns	No concerns	Very low
LAM vs PAL	0	Some concerns	Suspected	No concerns	Major concerns	No concerns	No concerns	Very low
LAM vs QUE	0	Some concerns	Suspected	No concerns	Major concerns	No concerns	No concerns	Very low
LAM vs RIS	0	Some concerns	Suspected	No concerns	Major concerns	No concerns	No concerns	Very low
LAM vs TAM	0	Some concerns	Suspected	No concerns	Major concerns	No concerns	No concerns	Very low
LAM vs VALP	0	Some concerns	Suspected	No concerns	Major concerns	No concerns	No concerns	Very low
LAM vs ZIP	0	Some concerns	Suspected	No concerns	Major concerns	No concerns	No concerns	Very low
LIT vs OXC	0	Some concerns	Suspected	No concerns	Major concerns	No concerns	No concerns	Very low
LIT vs PAL	0	No concerns	Suspected	No concerns	Major concerns	No concerns	No concerns	Very low
LIT vs RIS	0	Some concerns	Suspected	No concerns	Major concerns	No concerns	No concerns	Very low
LIT vs TAM	0	No concerns	Suspected	No concerns	Major concerns	No concerns	No concerns	Very low
LIT vs ZIP	0	Some concerns	Suspected	No concerns	Major concerns	No concerns	No concerns	Very low
OLA vs OXC	0	Some concerns	Suspected	No concerns	Major concerns	No concerns	No concerns	Very low
OLA vs PAL	0	No concerns	Suspected	No concerns	Major concerns	No concerns	No concerns	Very low
OLA vs QUE	0	No concerns	Suspected	No concerns	Major concerns	No concerns	No concerns	Very low

OLA vs TAM	0	No concerns	Suspected	No concerns	Major concerns	No concerns	No concerns	Very low
OLA vs ZIP	0	Some concerns	Suspected	No concerns	Major concerns	No concerns	No concerns	Very low
OXC vs PAL	0	No concerns	Suspected	No concerns	Major concerns	No concerns	No concerns	Very low
OXC vs QUE	0	No concerns	Suspected	No concerns	Major concerns	No concerns	No concerns	Very low
OXC vs RIS	0	Some concerns	Suspected	No concerns	Major concerns	No concerns	No concerns	Very low
OXC vs TAM	0	No concerns	Suspected	No concerns	Major concerns	No concerns	No concerns	Very low
OXC vs ZIP	0	Some concerns	Suspected	No concerns	Major concerns	No concerns	No concerns	Very low
OXC vs PLA	0	Some concerns	Suspected	No concerns	Major concerns	No concerns	No concerns	Very low
PAL vs RIS	0	No concerns	Suspected	No concerns	Major concerns	No concerns	No concerns	Very low
PAL vs TAM	0	No concerns	Suspected	No concerns	Major concerns	No concerns	No concerns	Very low
PAL vs VALP	0	No concerns	Suspected	No concerns	Major concerns	No concerns	No concerns	Very low
PAL vs ZIP	0	No concerns	Suspected	No concerns	Major concerns	No concerns	No concerns	Very low
QUE vs RIS	0	Some concerns	Suspected	No concerns	Major concerns	No concerns	No concerns	Very low
QUE vs TAM	0	No concerns	Suspected	No concerns	Major concerns	No concerns	No concerns	Very low
QUE vs VALP	0	No concerns	Suspected	No concerns	Major concerns	No concerns	No concerns	Very low
QUE vs ZIP	0	No concerns	Suspected	No concerns	Major concerns	No concerns	No concerns	Very low
RIS vs TAM	0	No concerns	Suspected	No concerns	Major concerns	No concerns	No concerns	Very low
RIS vs VALP	0	Some concerns	Suspected	No concerns	Major concerns	No concerns	No concerns	Very low
RIS vs ZIP	0	Some concerns	Suspected	No concerns	Major concerns	No concerns	No concerns	Very low
TAM vs VALP	0	No concerns	Suspected	No concerns	Major concerns	No concerns	No concerns	Very low
TAM vs ZIP	0	No concerns	Suspected	No concerns	Major concerns	No concerns	No concerns	Very low
VALP vs ZIP	0	Some concerns	Suspected	No concerns	Major concerns	No concerns	No concerns	Very low

Supplementary Appendix 17. Dry mouth (N = 16, n = 3967)

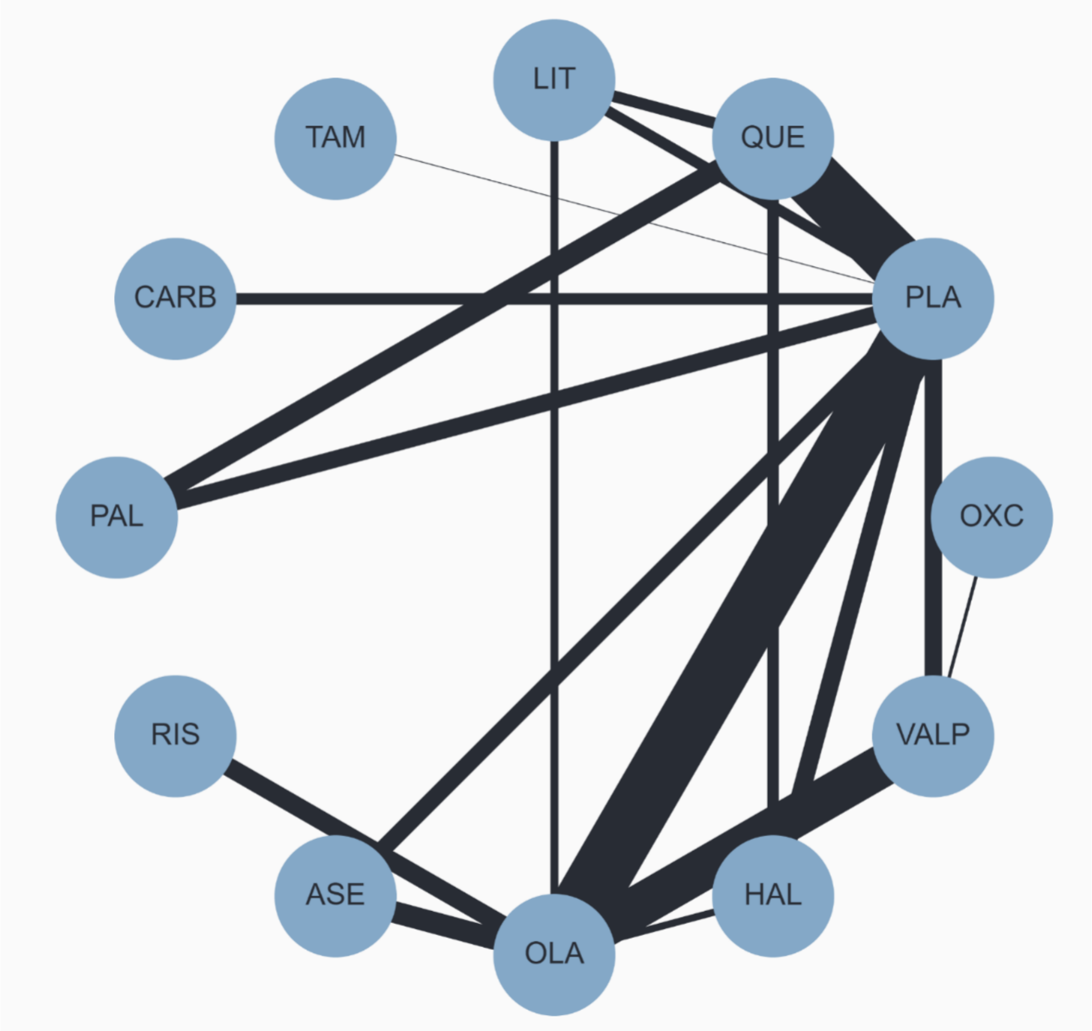

League table (risk ratio with 95% confidence interval)

ASE	0.285 (0.055, 1.486)	0.741 (0.182, 3.025)	1.176 (0.295, 4.683)	**0.310 (0.131, 0.734)**	2.247 (0.167, 30.299)	1.174 (0.309, 4.455)	**0.321 (0.106, 0.973)**	0.629 (0.218, 1.811)	0.582 (0.050, 6.740)	1.124 (0.396, 3.190)	1.164 (0.423, 3.205)
	CARB	2.598 (0.488, 13.820)	4.120 (0.808, 21.014)	1.085 (0.263, 4.481)	7.878 (0.466, 133.087)	4.114 (0.855, 19.810)	1.124 (0.280, 4.507)	2.204 (0.471, 10.326)	2.040 (0.154, 26.994)	3.939 (0.861, 18.029)	**4.079 (1.109, 15.010)**
		HAL	1.586 (0.406, 6.192)	0.418 (0.136, 1.282)	3.032 (0.205, 44.777)	1.584 (0.430, 5.838)	0.433 (0.151, 1.238)	0.849 (0.237, 3.043)	0.785 (0.067, 9.224)	1.516 (0.433, 5.310)	1.570 (0.551, 4.474)
			LIT	**0.263 (0.088, 0.786)**	1.912 (0.131, 27.877)	0.999 (0.301, 3.314)	**0.273 (0.111, 0.668)**	0.535 (0.153, 1.871)	0.495 (0.043, 5.653)	0.956 (0.281, 3.257)	0.990 (0.372, 2.633)
				OLA	7.258 (0.624, 84.487)	**3.791 (1.351, 10.641)**	1.035 (0.504, 2.126)	**2.031 (1.102, 3.744)**	1.879 (0.189, 18.731)	**3.629 (2.015, 6.536)**	**3.758 (2.147, 6.577)**
					OXC	0.522 (0.037, 7.428)	0.143 (0.011, 1.826)	0.280 (0.022, 3.511)	0.259 (0.009, 7.430)	0.500 (0.046, 5.418)	0.518 (0.042, 6.364)
						PAL	**0.273 (0.121, 0.614)**	0.536 (0.161, 1.778)	0.496 (0.045, 5.449)	0.957 (0.297, 3.086)	0.991 (0.412, 2.388)
							QUE	1.962 (0.763, 5.043)	1.815 (0.185, 17.773)	**3.505 (1.415, 8.682)**	**3.630 (2.243, 5.876)**
								RIS	0.925 (0.086, 9.989)	1.787 (0.765, 4.175)	1.850 (0.808, 4.239)
									TAM	1.931 (0.182, 20.543)	2.000 (0.215, 18.603)
										VALP	1.036 (0.472, 2.271)
											PLA

Evaluation of heterogeneity and inconsistency

Between study variance (τ^2)	Heterogeneity assessment	Random-effects design-by-treatment interaction model		
		Q	df	p
0.045	Low to moderate	11.190	9	0.263

Incoherence

	NMA, RR (95% CI)	Direct, RR (95% CI)	I^2	Indirect, RR (95% CI)	Inconsistency measures	
					Difference of RR	P value
ASE vs OLA	0.310 (0.131, 0.734)	0.289 (0.121, 0.688)	na	66.287 (0.033, 131491.830)	0.004 (0.000, 9.077)	0.163
ASE vs PLA	1.164 (0.423, 3.205)	4.330 (0.527, 35.588)	na	0.784 (0.247, 2.489)	5.523 (0.500, 61.038)	0.163
CARB vs PLA		4.079 (1.109, 15.010)	na			
HAL vs OLA	0.418 (0.136, 1.282)	0.350 (0.047, 2.613)	na	0.453 (0.117, 1.748)	0.773 (0.069, 8.715)	0.835
HAL vs QUE	0.433 (0.151, 1.238)	0.589 (0.166, 2.090)	na	0.218 (0.033, 1.440)	2.694 (0.278, 26.120)	0.392
HAL vs PLA	1.570 (0.551, 4.474)	1.276 (0.376, 4.322)	0.0%	2.803 (0.365, 21.516)	0.455 (0.042, 4.896)	0.516
LIT vs OLA	0.263 (0.088, 0.786)	0.194 (0.009, 4.091)	na	0.275 (0.085, 0.888)	0.706 (0.027, 18.453)	0.834
LIT vs QUE	0.273 (0.111, 0.668)	0.252 (0.098, 0.645)	na	0.583 (0.032, 10.725)	0.432 (0.020, 9.223)	0.591
LIT vs PLA	0.990 (0.372, 2.633)	2.970 (0.582, 15.142)	na	0.533 (0.157, 1.811)	5.573 (0.727, 42.751)	0.098
OLA vs RIS		2.031 (1.102, 3.744)	na			
OLA vs VALP	3.629 (2.015, 6.536)	3.504 (1.935, 6.343)	17.7%	25.907 (0.306, 2189.876)	0.135 (0.002, 11.892)	0.381
OLA vs PLA	3.758 (2.147, 6.577)	3.608 (2.012, 6.470)	0.0%	5.951 (0.839, 42.199)	0.606 (0.079, 4.681)	0.631
OXC vs VALP		0.500 (0.046, 5.418)	na			
PAL vs QUE	0.273 (0.121, 0.614)	0.307 (0.134, 0.705)	na	0.030 (0.001, 1.107)	10.146 (0.252, 407.932)	0.219
PAL vs PLA	0.991 (0.412, 2.388)	0.696 (0.245, 1.978)	na	2.341 (0.460, 11.915)	0.297 (0.043, 2.056)	0.219
QUE vs PLA	3.630 (2.243, 5.876)	3.669 (2.248, 5.988)	53.3%	2.655 (0.189, 37.263)	1.382 (0.094, 20.296)	0.813
TAM vs PLA		2.000 (0.215, 18.603)	na			

VALP vs PLA	1.036 (0.472, 2.271)	0.871 (0.200, 3.792)	na	1.110 (0.439, 2.808)	0.785 (0.138, 4.469)	0.785

Forest plot (vs placebo, the numbers are risk ratio with 95% confidence interval)

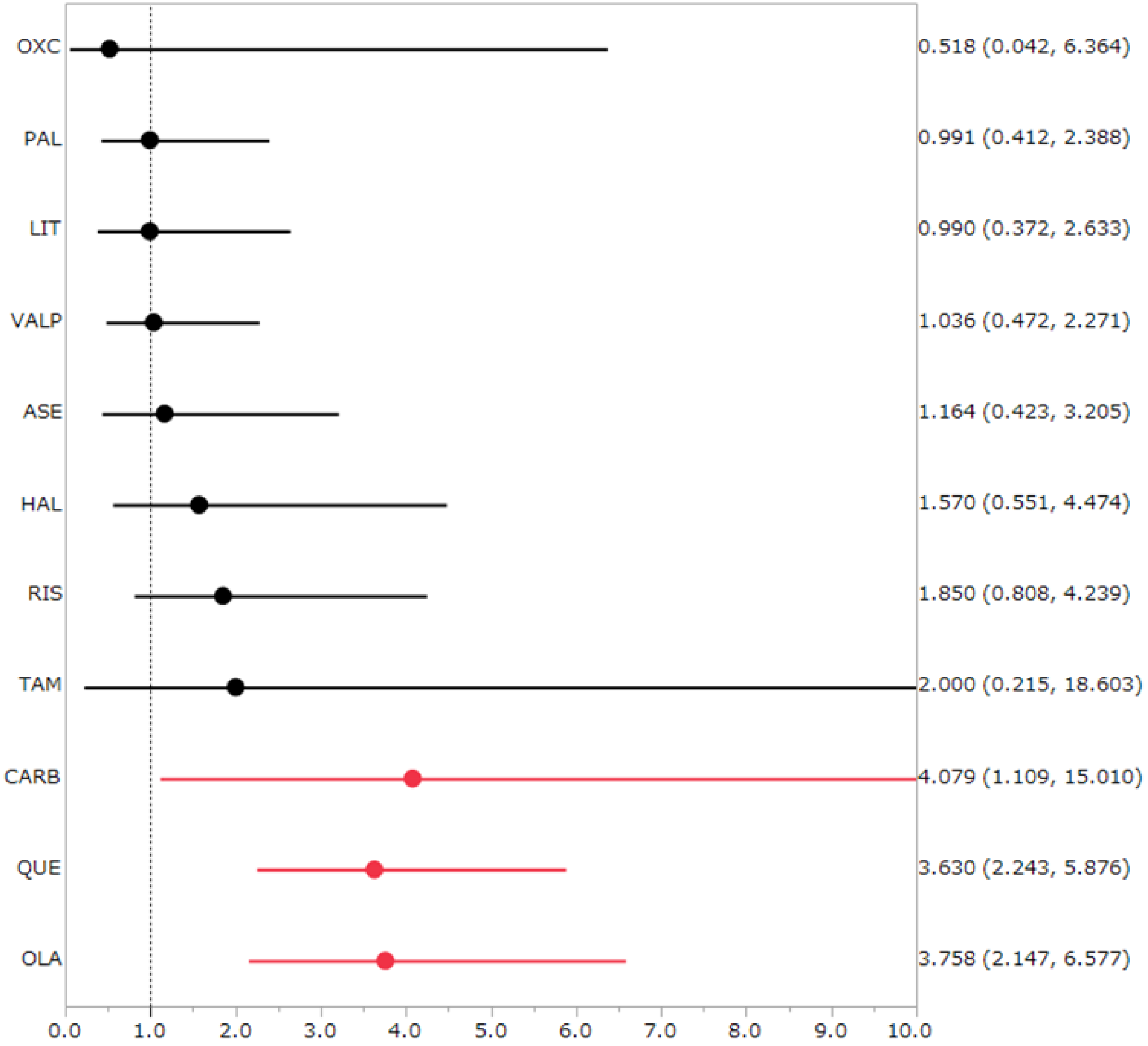

P-score

OXC	0.790
PAL	0.701
LIT	0.695
VALP	0.652
ASE	0.527
HAL	0.518
RIS	0.434
TAM	0.395
CARB	0.220
QUE	0.188
OLA	0.136

Funnel plot (only double-blind, placebo-controlled trials)

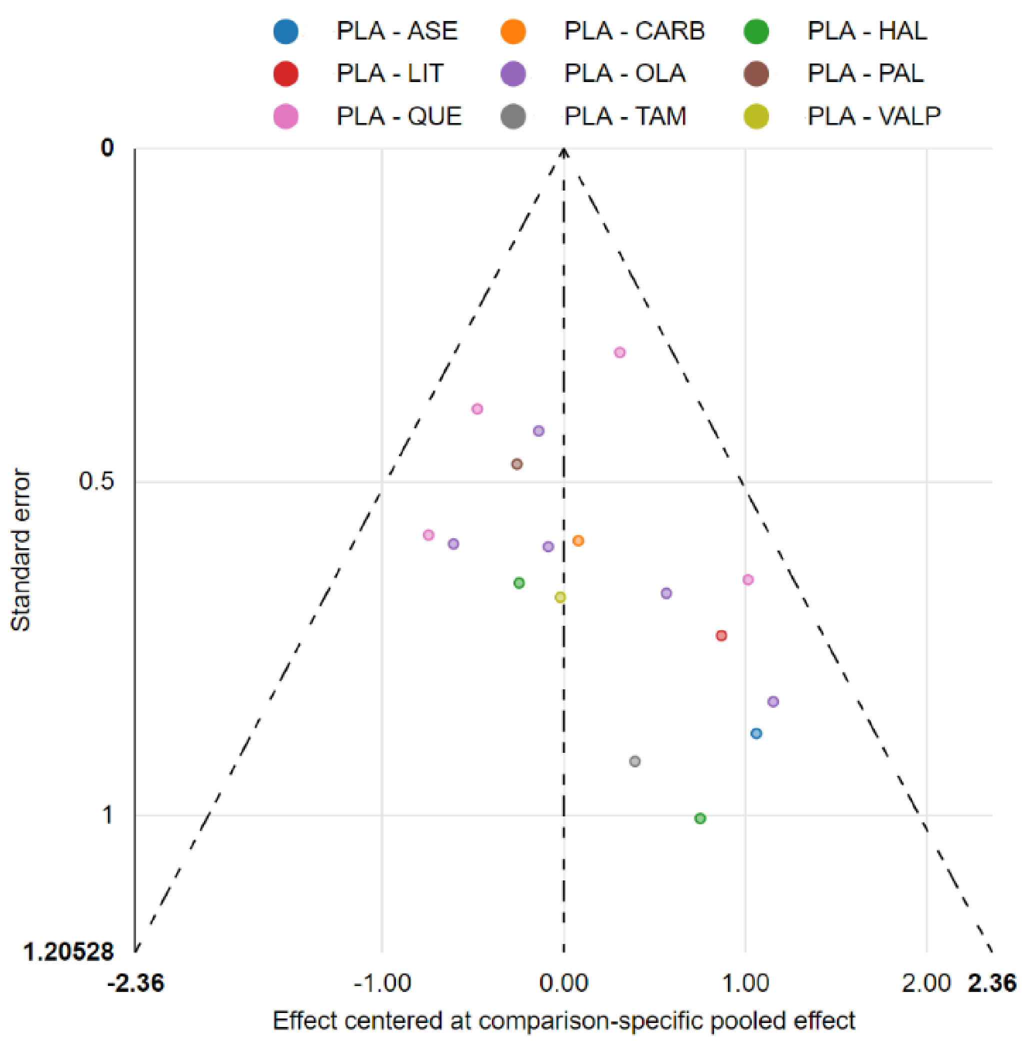

CINeMA confidence rating

Comparison	Number of studies	Within-study bias	Reporting bias	Indirectness	Imprecision	Heterogeneity	Incoherence	Confidence rating
ASE vs OLA	1	Some concerns	Suspected	No concerns	No concerns	No concerns	No concerns	Low
ASE vs PLA	1	Some concerns	Suspected	No concerns	Major concerns	No concerns	No concerns	Very low
CARB vs PLA	1	Some concerns	Suspected	No concerns	No concerns	Major concerns	No concerns	Very low
HAL vs OLA	1	Some concerns	Suspected	No concerns	Major concerns	No concerns	No concerns	Very low
HAL vs QUE	1	Some concerns	Suspected	No concerns	Major concerns	No concerns	No concerns	Very low
HAL vs PLA	2	Some concerns	Suspected	No concerns	Major concerns	No concerns	No concerns	Very low
LIT vs OLA	1	No concerns	Suspected	No concerns	No concerns	No concerns	No concerns	Moderate
LIT vs QUE	1	No concerns	Suspected	No concerns	No concerns	No concerns	No concerns	Moderate
LIT vs PLA	1	No concerns	Suspected	No concerns	Major concerns	No concerns	No concerns	Low
OLA vs RIS	1	Some concerns	Suspected	No concerns	No concerns	Major concerns	No concerns	Very low
OLA vs VALP	3	No concerns	Suspected	No concerns	No concerns	No concerns	No concerns	Moderate
OLA vs PLA	5	Some concerns	Suspected	No concerns	No concerns	No concerns	No concerns	Low
OXC vs VALP	1	Some concerns	Suspected	No concerns	Major concerns	No concerns	No concerns	Very low
PAL vs QUE	1	No concerns	Suspected	No concerns	No concerns	No concerns	No concerns	Moderate
PAL vs PLA	1	No concerns	Suspected	No concerns	Major concerns	No concerns	No concerns	Low
QUE vs PLA	4	No concerns	Suspected	No concerns	No concerns	No concerns	No concerns	Moderate
TAM vs PLA	1	No concerns	Suspected	No concerns	Major concerns	No concerns	No concerns	Low
VALP vs PLA	1	No concerns	Suspected	No concerns	Major concerns	No concerns	No concerns	Low
ASE vs CARB	0	Some concerns	Suspected	No concerns	Major concerns	No concerns	No concerns	Very low
ASE vs HAL	0	Some concerns	Suspected	No concerns	Major concerns	No concerns	No concerns	Very low
ASE vs LIT	0	No concerns	Suspected	No concerns	Major concerns	No concerns	No concerns	Very low
ASE vs OXC	0	Some concerns	Suspected	No concerns	Major concerns	No concerns	No concerns	Very low
ASE vs PAL	0	Some concerns	Suspected	No concerns	Major concerns	No concerns	No concerns	Very low

ASE vs QUE	0	Some concerns	Suspected	No concerns	No concerns	Major concerns	No concerns	Very low
ASE vs RIS	0	Some concerns	Suspected	No concerns	Major concerns	No concerns	No concerns	Very low
ASE vs TAM	0	Some concerns	Suspected	No concerns	Major concerns	No concerns	No concerns	Very low
ASE vs VALP	0	Some concerns	Suspected	No concerns	Major concerns	No concerns	No concerns	Very low
CARB vs HAL	0	Some concerns	Suspected	No concerns	Major concerns	No concerns	No concerns	Very low
CARB vs LIT	0	No concerns	Suspected	No concerns	Major concerns	No concerns	No concerns	Very low
CARB vs OLA	0	Some concerns	Suspected	No concerns	Major concerns	No concerns	No concerns	Very low
CARB vs OXC	0	Some concerns	Suspected	No concerns	Major concerns	No concerns	No concerns	Very low
CARB vs PAL	0	No concerns	Suspected	No concerns	Major concerns	No concerns	No concerns	Very low
CARB vs QUE	0	Some concerns	Suspected	No concerns	Major concerns	No concerns	No concerns	Very low
CARB vs RIS	0	Some concerns	Suspected	No concerns	Major concerns	No concerns	No concerns	Very low
CARB vs TAM	0	Some concerns	Suspected	No concerns	Major concerns	No concerns	No concerns	Very low
CARB vs VALP	0	Some concerns	Suspected	No concerns	Major concerns	No concerns	No concerns	Very low
HAL vs LIT	0	No concerns	Suspected	No concerns	Major concerns	No concerns	No concerns	Very low
HAL vs OXC	0	Some concerns	Suspected	No concerns	Major concerns	No concerns	No concerns	Very low
HAL vs PAL	0	No concerns	Suspected	No concerns	Major concerns	No concerns	No concerns	Very low
HAL vs RIS	0	Some concerns	Suspected	No concerns	Major concerns	No concerns	No concerns	Very low
HAL vs TAM	0	No concerns	Suspected	No concerns	Major concerns	No concerns	No concerns	Very low
HAL vs VALP	0	Some concerns	Suspected	No concerns	Major concerns	No concerns	No concerns	Very low
LIT vs OXC	0	No concerns	Suspected	No concerns	Major concerns	No concerns	No concerns	Very low
LIT vs PAL	0	No concerns	Suspected	No concerns	Major concerns	No concerns	No concerns	Very low
LIT vs RIS	0	Some concerns	Suspected	No concerns	Major concerns	No concerns	No concerns	Very low
LIT vs TAM	0	No concerns	Suspected	No concerns	Major concerns	No concerns	No concerns	Very low
LIT vs VALP	0	No concerns	Suspected	No concerns	Major concerns	No concerns	No concerns	Very low
OLA vs OXC	0	Some concerns	Suspected	No concerns	Major concerns	No concerns	No concerns	Very low

OLA vs PAL	0	No concerns	Suspected	No concerns	No concerns	No concerns	No concerns	Low
OLA vs QUE	0	No concerns	Suspected	No concerns	Major concerns	No concerns	No concerns	Very low
OLA vs TAM	0	No concerns	Suspected	No concerns	Major concerns	No concerns	No concerns	Very low
OXC vs PAL	0	No concerns	Suspected	No concerns	Major concerns	No concerns	No concerns	Very low
OXC vs QUE	0	No concerns	Suspected	No concerns	Major concerns	No concerns	No concerns	Very low
OXC vs RIS	0	Some concerns	Suspected	No concerns	Major concerns	No concerns	No concerns	Very low
OXC vs TAM	0	No concerns	Suspected	No concerns	Major concerns	No concerns	No concerns	Very low
OXC vs PLA	0	Some concerns	Suspected	No concerns	Major concerns	No concerns	No concerns	Very low
PAL vs RIS	0	Some concerns	Suspected	No concerns	Major concerns	No concerns	No concerns	Very low
PAL vs TAM	0	No concerns	Suspected	No concerns	Major concerns	No concerns	No concerns	Very low
PAL vs VALP	0	No concerns	Suspected	No concerns	Major concerns	No concerns	No concerns	Very low
QUE vs RIS	0	Some concerns	Suspected	No concerns	Major concerns	No concerns	No concerns	Very low
QUE vs TAM	0	No concerns	Suspected	No concerns	Major concerns	No concerns	No concerns	Very low
QUE vs VALP	0	No concerns	Suspected	No concerns	No concerns	No concerns	No concerns	Low
RIS vs TAM	0	Some concerns	Suspected	No concerns	Major concerns	No concerns	No concerns	Very low
RIS vs VALP	0	Some concerns	Suspected	No concerns	Major concerns	No concerns	No concerns	Very low
RIS vs PLA	0	Some concerns	Suspected	No concerns	Major concerns	No concerns	No concerns	Very low
TAM vs VALP	0	No concerns	Suspected	No concerns	Major concerns	No concerns	No concerns	Very low

Supplementary Appendix 18. Diarrhea (N = 20, n = 4981)

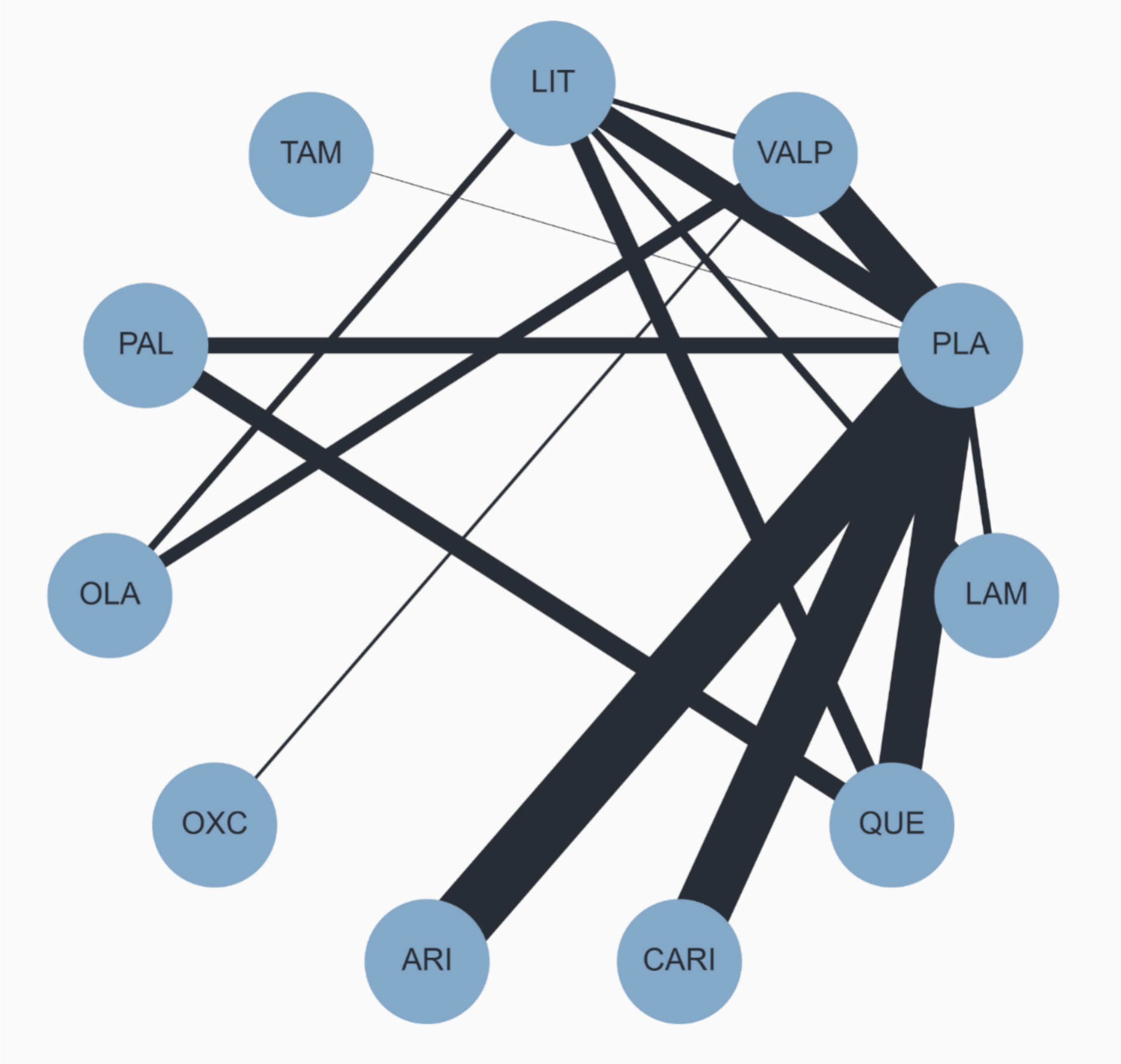

League table (risk ratio with 95% confidence interval)

ARI	0.938 (0.393, 2.239)	2.760 (0.266, 28.606)	0.999 (0.419, 2.383)	1.061 (0.333, 3.381)	3.287 (0.138, 78.038)	3.069 (0.538, 17.496)	0.998 (0.404, 2.463)	4.690 (0.232, 94.737)	0.658 (0.302, 1.432)	0.938 (0.563, 1.563)
	CARI	2.942 (0.270, 32.050)	1.065 (0.393, 2.882)	1.131 (0.322, 3.973)	3.504 (0.142, 86.323)	3.271 (0.537, 19.931)	1.063 (0.381, 2.966)	5.000 (0.238, 104.994)	0.701 (0.280, 1.754)	1.000 (0.494, 2.023)
		LAM	0.362 (0.039, 3.333)	0.384 (0.033, 4.436)	1.191 (0.025, 56.003)	1.112 (0.068, 18.212)	0.361 (0.035, 3.703)	1.699 (0.040, 71.450)	0.238 (0.023, 2.434)	0.340 (0.035, 3.328)
			LIT	1.062 (0.358, 3.151)	3.291 (0.138, 78.649)	3.072 (0.534, 17.681)	0.999 (0.459, 2.173)	4.695 (0.224, 98.573)	0.658 (0.294, 1.473)	0.939 (0.465, 1.898)
				OLA	3.098 (0.125, 76.570)	2.891 (0.415, 20.160)	0.940 (0.281, 3.147)	4.419 (0.191, 102.023)	0.620 (0.245, 1.569)	0.884 (0.312, 2.501)
					OXC	0.933 (0.027, 32.031)	0.303 (0.012, 7.444)	1.427 (0.019, 105.795)	0.200 (0.009, 4.308)	0.285 (0.013, 6.499)
						PAL	0.325 (0.061, 1.745)	1.529 (0.051, 45.686)	0.214 (0.037, 1.238)	0.306 (0.058, 1.615)
							QUE	4.702 (0.222, 99.703)	0.659 (0.267, 1.625)	0.940 (0.446, 1.982)
								TAM	0.140 (0.007, 2.871)	0.200 (0.010, 3.867)
									VALP	1.427 (0.793, 2.567)
										PLA

Evaluation of heterogeneity and inconsistency

Between study variance (τ^2)	Heterogeneity assessment	Random-effects design-by-treatment interaction model		
		Q	df	p
0.119	Moderate to high	6.651	7	0.466

Incoherence

	NMA, RR (95% CI)	Direct, RR (95% CI)	I^2	Indirect, RR (95% CI)	Inconsistency measures	
					Difference of RR	P value
ARI vs PLA		0.938 (0.563, 1.563)	0.0%			
CARI vs PLA		1.000 (0.494, 2.023)	70.9%			
LAM vs LIT	0.362 (0.039, 3.333)	0.264 (0.027, 2.553)	na	349.324 (0.009, 13612512.983)	0.001 (0.000, 37.412)	0.193
LAM vs PLA	0.340 (0.035, 3.328)	1.040 (0.061, 17.724)	na	0.043 (0.001, 2.029)	23.963 (0.202, 2844.663)	0.193
LIT vs OLA	1.062 (0.358, 3.151)	0.486 (0.081, 2.930)	na	1.669 (0.426, 6.541)	0.291 (0.030, 2.781)	0.284
LIT vs QUE	0.999 (0.459, 2.173)	0.812 (0.319, 2.067)	0.0%	1.590 (0.392, 6.446)	0.511 (0.095, 2.749)	0.434
LIT vs VALP	0.658 (0.294, 1.473)	1.198 (0.346, 4.149)	na	0.426 (0.148, 1.228)	2.810 (0.550, 14.368)	0.215
LIT vs PLA	0.939 (0.465, 1.898)	1.149 (0.494, 2.673)	0.0%	0.594 (0.167, 2.120)	1.934 (0.420, 8.900)	0.397
OLA vs VALP	0.620 (0.245, 1.569)	0.474 (0.166, 1.355)	na	1.630 (0.221, 12.016)	0.291 (0.030, 2.781)	0.284
OXC vs VALP		0.200 (0.009, 4.308)	na			
PAL vs QUE	0.325 (0.061, 1.745)	0.495 (0.080, 3.042)	na	0.027 (0.000, 2.241)	18.582 (0.154, 2235.902)	0.232
PAL vs PLA	0.306 (0.058, 1.615)	0.216 (0.037, 1.256)	na	6.062 (0.034, 1068.061)	0.036 (0.000, 8.415)	0.232
QUE vs PLA	0.940 (0.446, 1.982)	0.801 (0.341, 1.881)	0.0%	1.576 (0.340, 7.302)	0.508 (0.088, 2.940)	0.450
TAM vs PLA		0.200 (0.010, 3.867)	na			
VALP vs PLA	1.427 (0.793, 2.567)	1.330 (0.722, 2.450)	42.3%	3.371 (0.397, 28.648)	0.395 (0.043, 3.654)	0.413

For some outcomes of a network meta-analysis, odds ratio may be considered a more appropriate effect size than risk ratio (Huhn 2020). For example, odds ratio has mathematical properties that make it more appropriate for some outcome of network meta-analysis (e.g. the odds ratio is symmetrical). Therefore, although we

performed a network meta-analysis for this outcome using odds ratio, the results also had global heterogeneity (τ^2 = 0.143). The result is reported for presentation purposes only because these analyses were not included in a prespecified analysis plan; thus, further study will be needed to explore the reasons for the global heterogeneity.

Huhn M, et al. Lancet 2019; 394(10202): 939-951.

Forest plot (vs placebo, the numbers are risk ratio with 95% confidence interval)

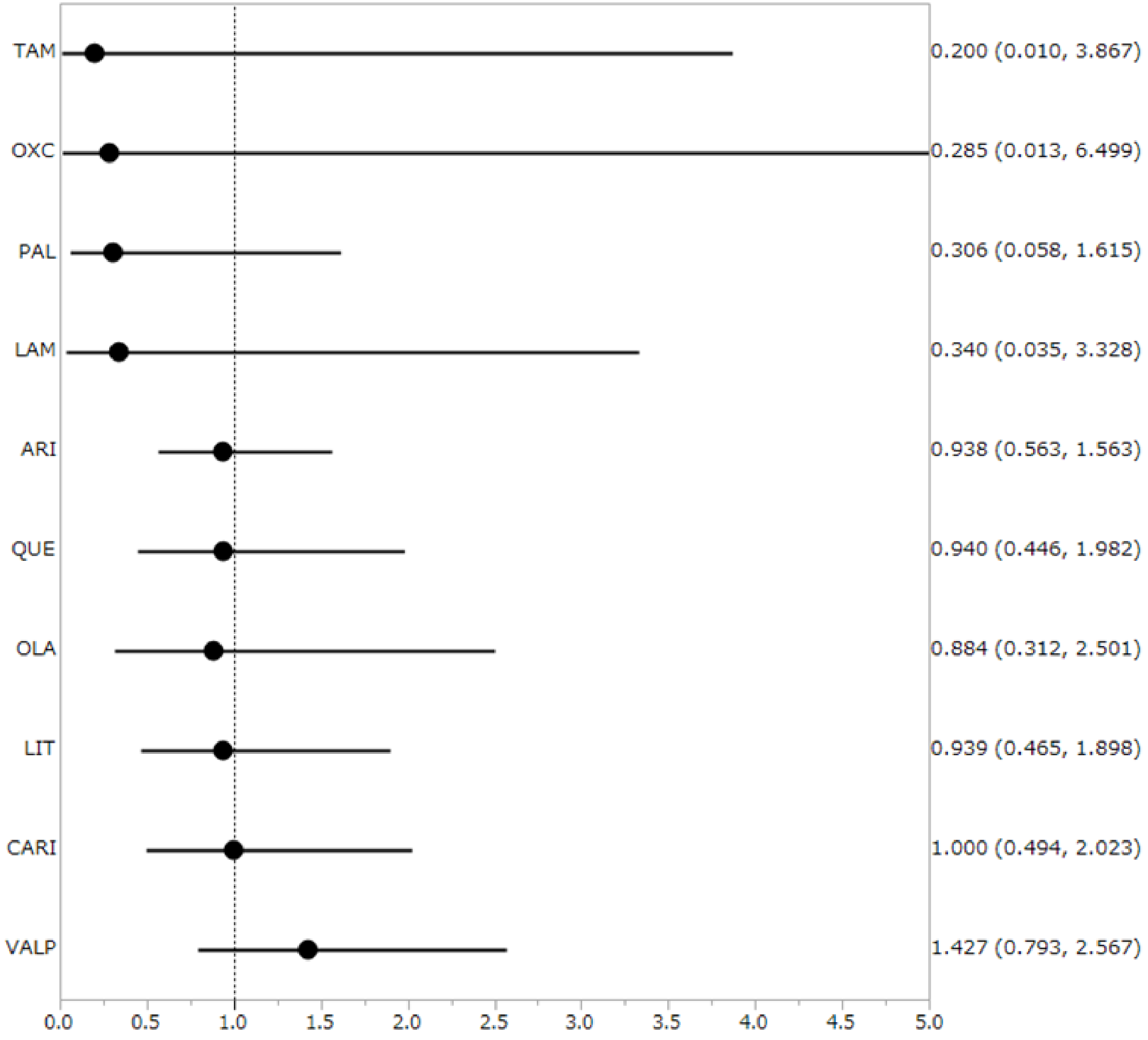

P-score

TAM	0.958
OXC	0.910
PAL	0.693
LAM	0.629
ARI	0.410
QUE	0.381
OLA	0.370
LIT	0.361
CARI	0.296
VALP	0.114

Funnel plot (only double-blind, placebo-controlled trials)

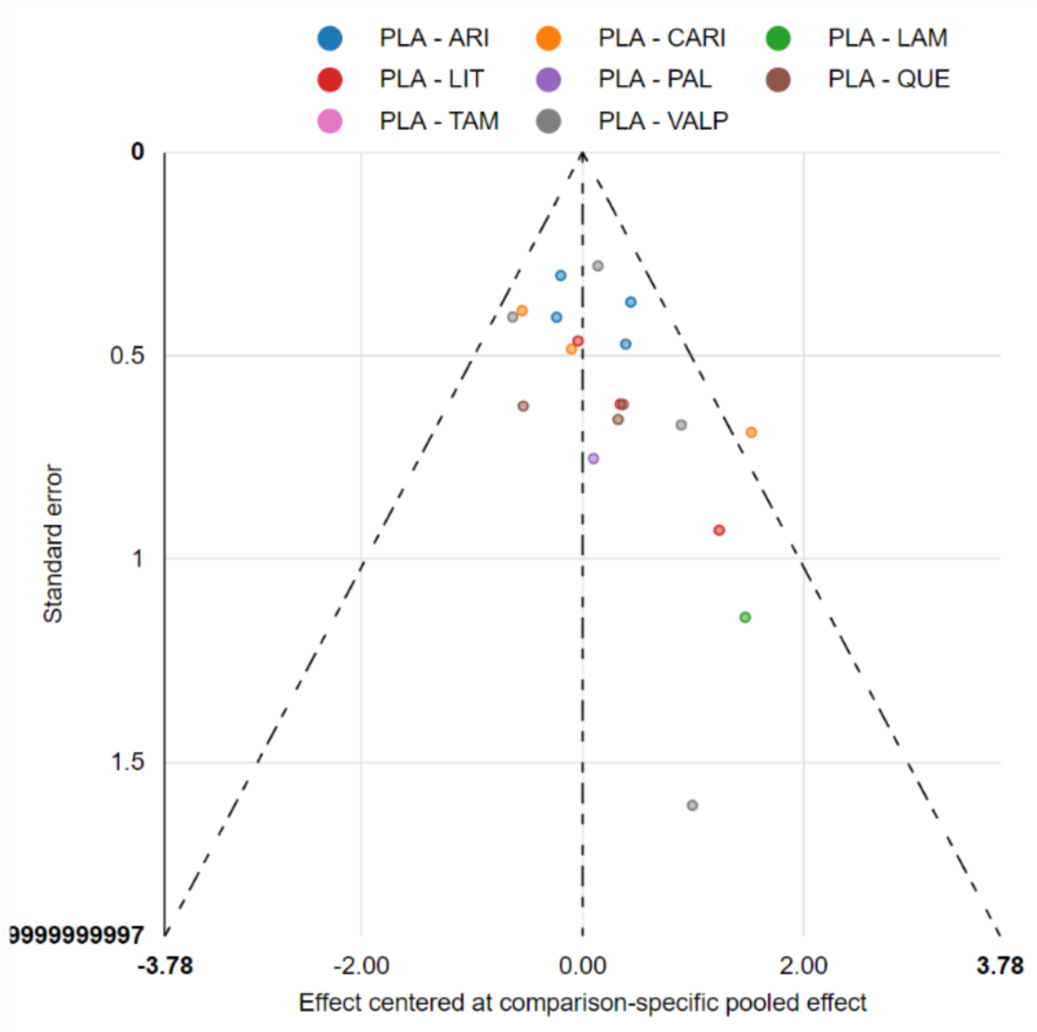

CINeMA confidence rating

Comparison	Number of studies	Within-study bias	Reporting bias	Indirectness	Imprecision	Heterogeneity	Incoherence	Confidence rating
ARI vs PLA	4	Some concerns	Suspected	No concerns	Major concerns	No concerns	No concerns	Very low
CARI vs PLA	3	Some concerns	Suspected	No concerns	Major concerns	No concerns	No concerns	Very low
LAM vs LIT	1	Some concerns	Suspected	No concerns	Major concerns	No concerns	No concerns	Very low
LAM vs PLA	1	Some concerns	Suspected	No concerns	Major concerns	No concerns	No concerns	Very low
LIT vs OLA	1	No concerns	Suspected	No concerns	Major concerns	No concerns	No concerns	Low
LIT vs QUE	2	Some concerns	Suspected	No concerns	Major concerns	No concerns	No concerns	Very low
LIT vs VALP	1	Some concerns	Suspected	No concerns	Major concerns	No concerns	No concerns	Very low
LIT vs PLA	3	Some concerns	Suspected	No concerns	Major concerns	No concerns	No concerns	Very low
OLA vs VALP	1	No concerns	Suspected	No concerns	Major concerns	No concerns	No concerns	Low
OXC vs VALP	1	Some concerns	Suspected	No concerns	Major concerns	No concerns	No concerns	Very low
PAL vs QUE	1	No concerns	Suspected	No concerns	Major concerns	No concerns	No concerns	Low
PAL vs PLA	1	No concerns	Suspected	No concerns	Major concerns	No concerns	No concerns	Low
QUE vs PLA	3	No concerns	Suspected	No concerns	Major concerns	No concerns	No concerns	Low
TAM vs PLA	1	No concerns	Suspected	No concerns	Major concerns	No concerns	No concerns	Low
VALP vs PLA	4	No concerns	Suspected	No concerns	Major concerns	No concerns	No concerns	Low
ARI vs CARI	0	Some concerns	Suspected	No concerns	Major concerns	No concerns	No concerns	Very low
ARI vs LAM	0	Some concerns	Suspected	No concerns	Major concerns	No concerns	No concerns	Very low
ARI vs LIT	0	Some concerns	Suspected	No concerns	Major concerns	No concerns	No concerns	Very low
ARI vs OLA	0	Some concerns	Suspected	No concerns	Major concerns	No concerns	No concerns	Very low
ARI vs OXC	0	Some concerns	Suspected	No concerns	Major concerns	No concerns	No concerns	Very low
ARI vs PAL	0	No concerns	Suspected	No concerns	Major concerns	No concerns	No concerns	Very low
ARI vs QUE	0	Some concerns	Suspected	No concerns	Major concerns	No concerns	No concerns	Very low
ARI vs TAM	0	Some concerns	Suspected	No concerns	Major concerns	No concerns	No concerns	Very low

ARI vs VALP	0	Some concerns	Suspected	No concerns	Major concerns	No concerns	No concerns	Very low
CARI vs LAM	0	Some concerns	Suspected	No concerns	Major concerns	No concerns	No concerns	Very low
CARI vs LIT	0	Some concerns	Suspected	No concerns	Major concerns	No concerns	No concerns	Very low
CARI vs OLA	0	Some concerns	Suspected	No concerns	Major concerns	No concerns	No concerns	Very low
CARI vs OXC	0	Some concerns	Suspected	No concerns	Major concerns	No concerns	No concerns	Very low
CARI vs PAL	0	No concerns	Suspected	No concerns	Major concerns	No concerns	No concerns	Very low
CARI vs QUE	0	Some concerns	Suspected	No concerns	Major concerns	No concerns	No concerns	Very low
CARI vs TAM	0	Some concerns	Suspected	No concerns	Major concerns	No concerns	No concerns	Very low
CARI vs VALP	0	Some concerns	Suspected	No concerns	Major concerns	No concerns	No concerns	Very low
LAM vs OLA	0	Some concerns	Suspected	No concerns	Major concerns	No concerns	No concerns	Very low
LAM vs OXC	0	Some concerns	Suspected	No concerns	Major concerns	No concerns	No concerns	Very low
LAM vs PAL	0	Some concerns	Suspected	No concerns	Major concerns	No concerns	No concerns	Very low
LAM vs QUE	0	Some concerns	Suspected	No concerns	Major concerns	No concerns	No concerns	Very low
LAM vs TAM	0	Some concerns	Suspected	No concerns	Major concerns	No concerns	No concerns	Very low
LAM vs VALP	0	Some concerns	Suspected	No concerns	Major concerns	No concerns	No concerns	Very low
LIT vs OXC	0	Some concerns	Suspected	No concerns	Major concerns	No concerns	No concerns	Very low
LIT vs PAL	0	No concerns	Suspected	No concerns	Major concerns	No concerns	No concerns	Very low
LIT vs TAM	0	No concerns	Suspected	No concerns	Major concerns	No concerns	No concerns	Very low
OLA vs OXC	0	Some concerns	Suspected	No concerns	Major concerns	No concerns	No concerns	Very low
OLA vs PAL	0	No concerns	Suspected	No concerns	Major concerns	No concerns	No concerns	Very low
OLA vs QUE	0	No concerns	Suspected	No concerns	Major concerns	No concerns	No concerns	Very low
OLA vs TAM	0	No concerns	Suspected	No concerns	Major concerns	No concerns	No concerns	Very low
OLA vs PLA	0	No concerns	Suspected	No concerns	Major concerns	No concerns	No concerns	Very low
OXC vs PAL	0	No concerns	Suspected	No concerns	Major concerns	No concerns	No concerns	Very low
OXC vs QUE	0	Some concerns	Suspected	No concerns	Major concerns	No concerns	No concerns	Very low

OXC vs TAM	0	No concerns	Suspected	No concerns	Major concerns	No concerns	No concerns	Very low
OXC vs PLA	0	Some concerns	Suspected	No concerns	Major concerns	No concerns	No concerns	Very low
PAL vs TAM	0	No concerns	Suspected	No concerns	Major concerns	No concerns	No concerns	Very low
PAL vs VALP	0	No concerns	Suspected	No concerns	Major concerns	No concerns	No concerns	Very low
QUE vs TAM	0	No concerns	Suspected	No concerns	Major concerns	No concerns	No concerns	Very low
QUE vs VALP	0	No concerns	Suspected	No concerns	Major concerns	No concerns	No concerns	Very low
TAM vs VALP	0	No concerns	Suspected	No concerns	Major concerns	No concerns	No concerns	Very low

Supplementary Appendix 19. Constipation (N = 27, n = 6670)

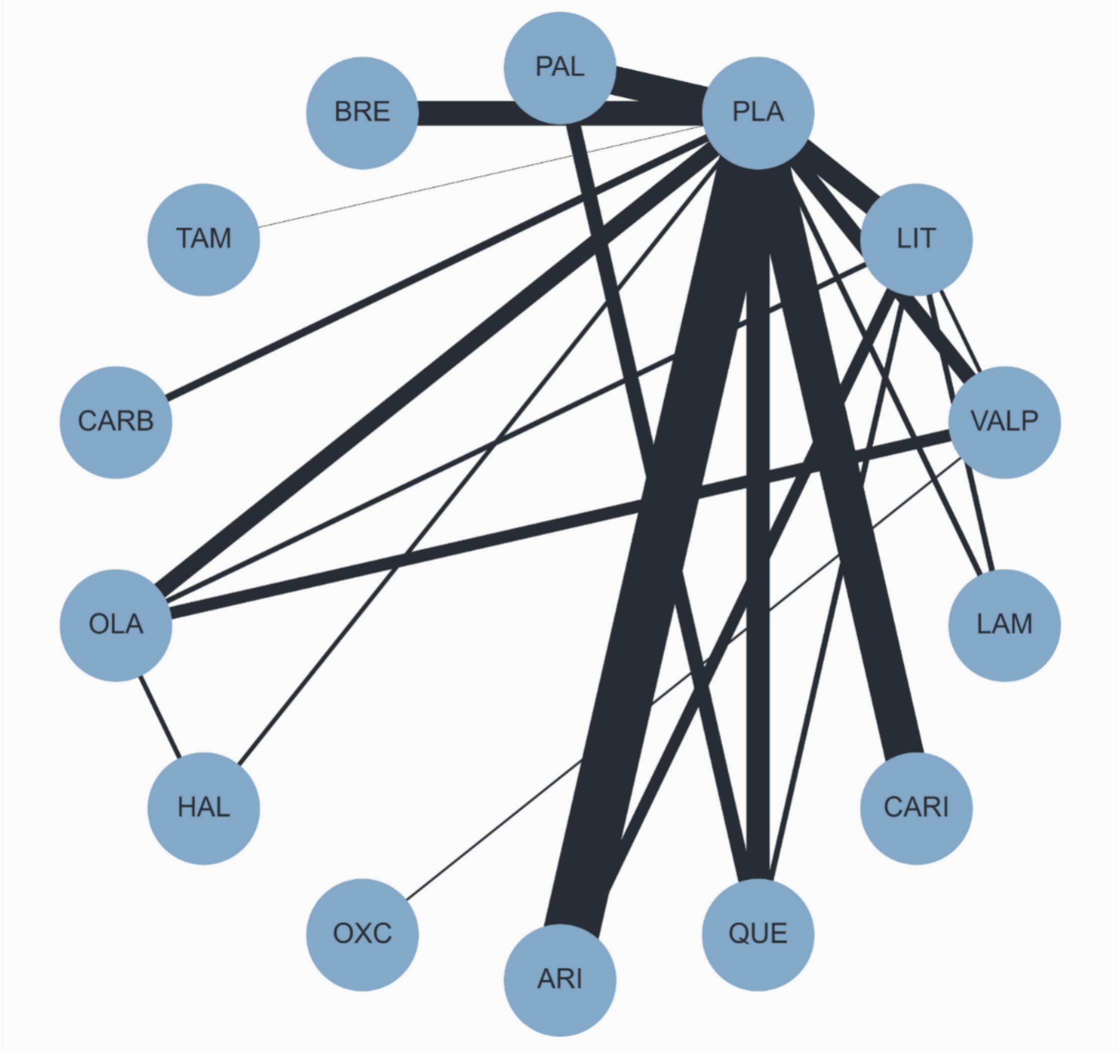

League table (risk ratio with 95% confidence interval)

ARI	2.224 (0.748, 6.613)	0.993 (0.330, 2.988)	0.959 (0.478, 1.923)	0.506 (0.094, 2.721)	0.861 (0.168, 4.408)	1.330 (0.742, 2.381)	0.896 (0.435, 1.849)	14.276 (0.677, 301.087)	1.006 (0.445, 2.277)	0.605 (0.296, 1.237)	1.735 (0.120, 25.126)	2.039 (0.997, 4.173)	**1.735 (1.152, 2.613)**
	BRE	0.446 (0.106, 1.880)	0.431 (0.136, 1.371)	0.227 (0.033, 1.560)	0.387 (0.059, 2.539)	0.598 (0.192, 1.859)	0.403 (0.123, 1.319)	6.421 (0.264, 155.929)	0.452 (0.131, 1.564)	**0.272 (0.083, 0.892)**	0.780 (0.046, 13.193)	0.917 (0.282, 2.986)	0.780 (0.284, 2.143)
		CARB	0.966 (0.301, 3.107)	0.509 (0.074, 3.520)	0.867 (0.131, 5.728)	1.340 (0.426, 4.214)	0.903 (0.273, 2.988)	14.384 (0.590, 350.829)	1.014 (0.290, 3.543)	0.610 (0.184, 2.022)	1.748 (0.103, 29.702)	2.055 (0.624, 6.766)	1.748 (0.628, 4.865)
			CARI	0.527 (0.093, 2.985)	0.898 (0.167, 4.832)	1.386 (0.646, 2.975)	0.935 (0.405, 2.159)	14.887 (0.686, 323.145)	1.049 (0.421, 2.616)	0.631 (0.273, 1.462)	1.809 (0.122, 26.937)	2.127 (0.927, 4.879)	**1.809 (1.031, 3.177)**
				HAL	1.703 (0.175, 16.592)	2.630 (0.491, 14.102)	1.773 (0.375, 8.382)	28.242 (0.952, 838.069)	1.990 (0.335, 11.841)	1.198 (0.211, 6.795)	3.433 (0.153, 76.884)	4.035 (0.777, 20.941)	3.433 (0.666, 17.695)
					LAM	1.545 (0.299, 7.968)	1.041 (0.191, 5.673)	16.587 (0.547, 503.206)	1.169 (0.206, 6.639)	0.703 (0.130, 3.818)	2.016 (0.093, 43.908)	2.370 (0.436, 12.866)	2.016 (0.413, 9.850)
						LIT	0.674 (0.334, 1.360)	10.737 (0.512, 225.293)	0.757 (0.332, 1.722)	**0.455 (0.242, 0.856)**	1.305 (0.088, 19.248)	1.534 (0.766, 3.071)	1.305 (0.779, 2.186)
							OLA	15.928 (0.776, 327.013)	1.122 (0.441, 2.854)	0.676 (0.293, 1.557)	1.936 (0.128, 29.183)	**2.276 (1.260, 4.110)**	**1.936 (1.041, 3.599)**
								OXC	0.070 (0.003, 1.572)	**0.042 (0.002, 0.920)**	0.122 (0.002, 6.746)	0.143 (0.007, 2.767)	0.122 (0.006, 2.505)
									PAL	0.602 (0.281, 1.287)	1.725 (0.112, 26.648)	2.027 (0.802, 5.122)	1.725 (0.839, 3.543)
										QUE	2.866 (0.190, 43.237)	**3.369 (1.472, 7.711)**	**2.866 (1.537, 5.345)**
											TAM	1.175 (0.078, 17.681)	1.000 (0.071, 14.031)

												VALP	0.851 (0.462, 1.567)
													PLA

Evaluation of heterogeneity and inconsistency

Between study variance (τ^2)	Heterogeneity assessment	Random-effects design-by-treatment interaction model		
		Q	df	p
0.066	Low to moderate	15.033	10	0.131

Incoherence

	NMA, RR (95% CI)	Direct, RR (95% CI)	I^2	Indirect, RR (95% CI)	Inconsistency measures	
					Difference of RR	P value
ARI vs LIT	1.330 (0.742, 2.381)	0.972 (0.428, 2.204)		1.834 (0.800, 4.205)	0.530 (0.165, 1.700)	0.285
ARI vs PLA	1.735 (1.152, 2.613)	1.843 (1.209, 2.809)	0.0%	0.640 (0.115, 3.552)	2.878 (0.493, 16.804)	0.240
BRE vs PLA		0.780 (0.284, 2.143)	40.1%			
CARB vs PLA		1.748 (0.628, 4.865)				
CARI vs PLA		1.809 (1.031, 3.177)	0.0%			
HAL vs OLA	1.773 (0.375, 8.382)	1.500 (0.309, 7.278)		245.231 (0.046, 1296657.036)	0.006 (0.000, 37.360)	0.252
HAL vs PLA	3.433 (0.666, 17.695)	9.600 (0.867, 106.346)		1.404 (0.149, 13.218)	6.837 (0.255, 183.186)	0.252
LAM vs LIT	1.545 (0.299, 7.968)	7.376 (0.371, 146.511)		0.787 (0.111, 5.604)	9.369 (0.262, 334.655)	0.220
LAM vs PLA	2.016 (0.413, 9.850)	1.456 (0.274, 7.731)		41.767 (0.255, 6832.186)	0.035 (0.000, 7.445)	0.220
LIT vs OLA	0.674 (0.334, 1.360)	0.324 (0.083, 1.263)		0.879 (0.388, 1.995)	0.368 (0.075, 1.803)	0.218
LIT vs QUE	0.455 (0.242, 0.856)	0.375 (0.164, 0.856)		0.599 (0.224, 1.598)	0.626 (0.174, 2.258)	0.475
LIT vs VALP	1.534 (0.766, 3.071)	1.643 (0.530, 5.094)		1.472 (0.611, 3.544)	1.116 (0.266, 4.677)	0.880
LIT vs PLA	1.305 (0.779, 2.186)	1.748 (0.861, 3.548)	15.4%	0.937 (0.441, 1.990)	1.867 (0.664, 5.249)	0.237
OLA vs VALP	2.276 (1.260, 4.110)	1.598 (0.777, 3.284)	59.9%	4.724 (1.677, 13.306)	0.338 (0.096, 1.194)	0.092
OLA vs PLA	1.936 (1.041, 3.599)	2.425 (0.997, 5.898)	15.8%	1.563 (0.658, 3.715)	1.552 (0.449, 5.365)	0.488
OXC vs VALP		0.143 (0.007, 2.767)				
PAL vs QUE	0.602 (0.281, 1.287)	0.900 (0.340, 2.381)		0.321 (0.095, 1.084)	2.803 (0.590, 13.314)	0.195

PAL vs PLA	1.725 (0.839, 3.543)	1.410 (0.618, 3.217)	5.0%	3.288 (0.752, 14.385)	0.429 (0.079, 2.325)	0.326
QUE vs PLA	2.866 (1.537, 5.345)	3.121 (1.262, 7.720)	0.0%	2.654 (1.124, 6.267)	1.176 (0.337, 4.097)	0.799
TAM vs PLA		1.000 (0.071, 14.031)				
VALP vs PLA	0.851 (0.462, 1.567)	0.620 (0.281, 1.367)	55.7%	1.359 (0.519, 3.557)	0.456 (0.131, 1.585)	0.217

Forest plot (vs placebo, the numbers are risk ratio with 95% confidence interval)

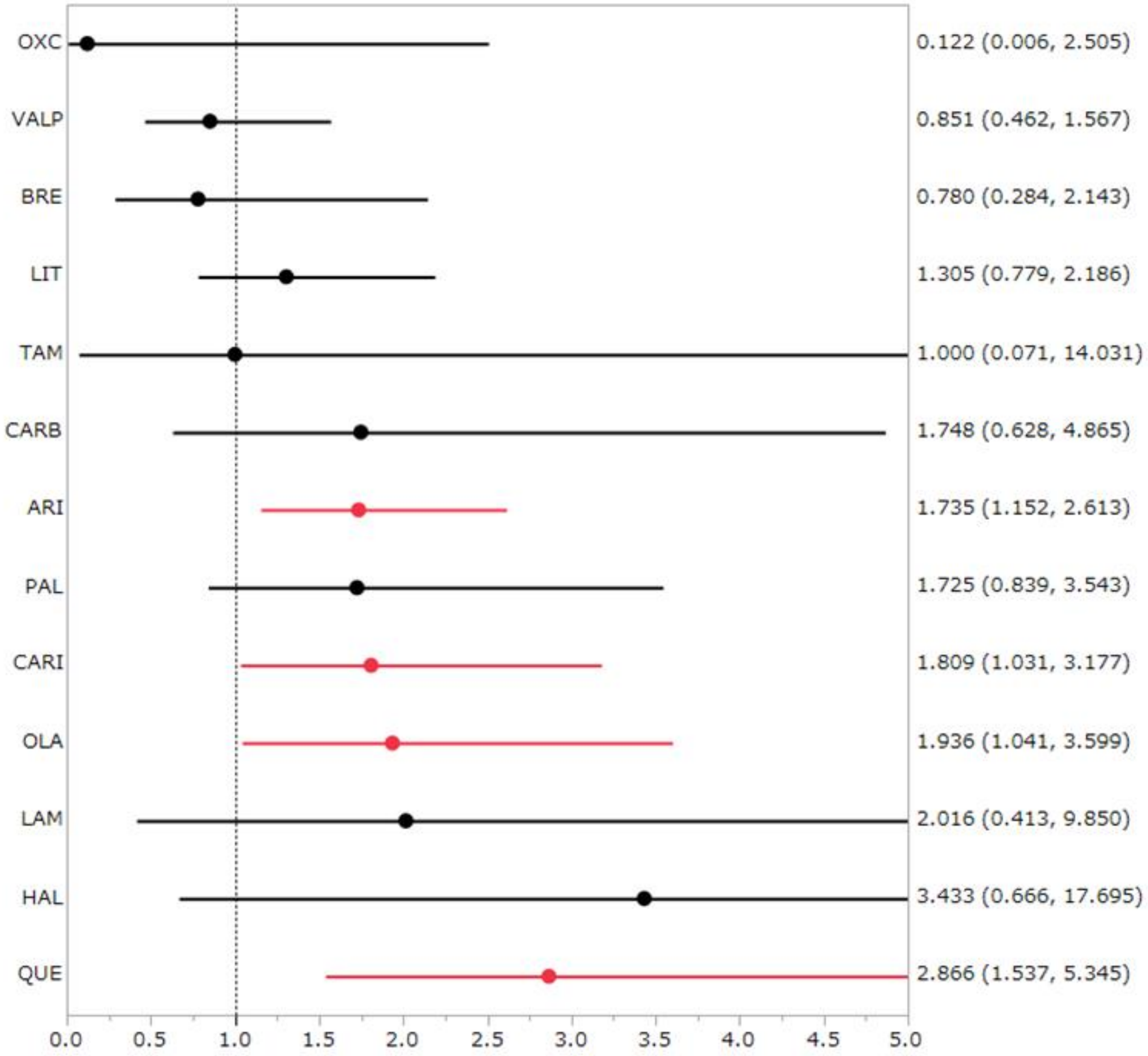

P-score

OXC	0.997
VALP	0.792
BRE	0.776
LIT	0.614
TAM	0.564
CARB	0.414
ARI	0.412
PAL	0.408
CARI	0.377
OLA	0.314
LAM	0.226
HAL	0.217
QUE	0.173

Funnel plot (only double-blind, placebo-controlled trials)

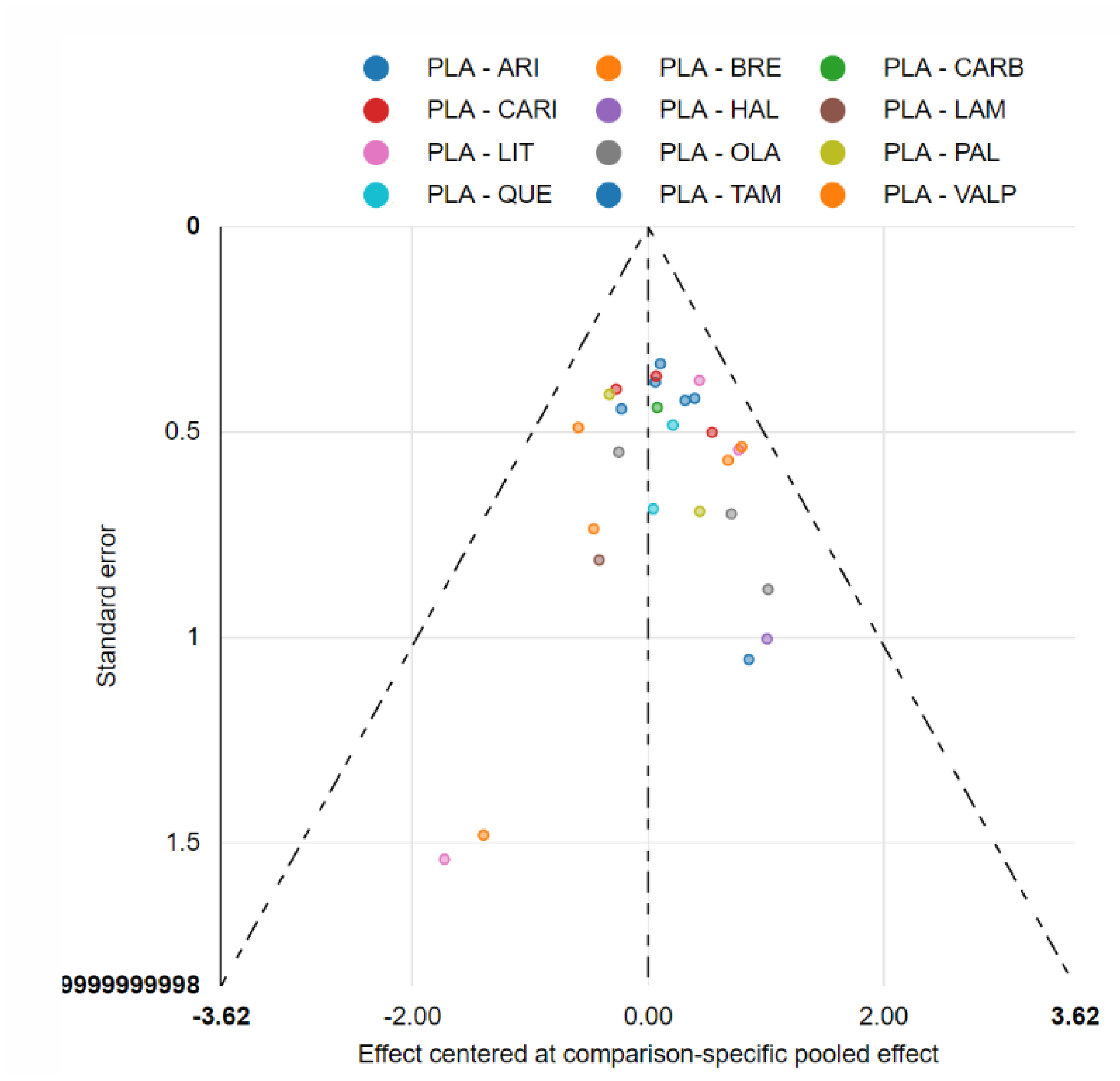

CINeMA confidence rating

Comparison	Number of studies	Within-study bias	Reporting bias	Indirectness	Imprecision	Heterogeneity	Incoherence	Confidence rating
ARI vs LIT	1	Some concerns	Suspected	No concerns	Major concerns	No concerns	No concerns	Very low
ARI vs PLA	5	Some concerns	Suspected	No concerns	No concerns	Major concerns	No concerns	Very low
BRE vs PLA	2	Some concerns	Suspected	No concerns	Major concerns	No concerns	No concerns	Very low
CARB vs PLA	1	Some concerns	Suspected	No concerns	Major concerns	No concerns	No concerns	Very low
CARI vs PLA	3	Some concerns	Suspected	No concerns	No concerns	Major concerns	No concerns	Very low
HAL vs OLA	1	Some concerns	Suspected	No concerns	Major concerns	No concerns	No concerns	Very low
HAL vs PLA	1	Some concerns	Suspected	No concerns	Major concerns	No concerns	No concerns	Very low
LAM vs LIT	1	Some concerns	Suspected	No concerns	Major concerns	No concerns	No concerns	Very low
LAM vs PLA	1	Some concerns	Suspected	No concerns	Major concerns	No concerns	No concerns	Very low
LIT vs OLA	1	Some concerns	Suspected	No concerns	Major concerns	No concerns	No concerns	Very low
LIT vs QUE	1	Some concerns	Suspected	No concerns	No concerns	Major concerns	No concerns	Very low
LIT vs VALP	1	Some concerns	Suspected	No concerns	Major concerns	No concerns	No concerns	Very low
LIT vs PLA	3	Some concerns	Suspected	No concerns	Major concerns	No concerns	No concerns	Very low
OLA vs VALP	2	No concerns	Suspected	No concerns	No concerns	Major concerns	Major concerns	Very low
OLA vs PLA	3	Some concerns	Suspected	No concerns	No concerns	Major concerns	No concerns	Very low
OXC vs VALP	1	Some concerns	Suspected	No concerns	Major concerns	No concerns	No concerns	Very low
PAL vs QUE	1	No concerns	Suspected	No concerns	Major concerns	No concerns	No concerns	Low
PAL vs PLA	2	No concerns	Suspected	No concerns	Major concerns	No concerns	No concerns	Low
QUE vs PLA	2	No concerns	Suspected	No concerns	No concerns	No concerns	No concerns	Moderate
TAM vs PLA	1	No concerns	Suspected	No concerns	Major concerns	No concerns	No concerns	Low
VALP vs PLA	3	Some concerns	Suspected	No concerns	Major concerns	No concerns	No concerns	Very low
ARI vs BRE	0	Some concerns	Suspected	No concerns	Major concerns	No concerns	No concerns	Very low
ARI vs CARB	0	Some concerns	Suspected	No concerns	Major concerns	No concerns	No concerns	Very low

ARI vs CARI	0	Some concerns	Suspected	No concerns	Major concerns	No concerns	No concerns	Very low
ARI vs HAL	0	Some concerns	Suspected	No concerns	Major concerns	No concerns	No concerns	Very low
ARI vs LAM	0	Some concerns	Suspected	No concerns	Major concerns	No concerns	No concerns	Very low
ARI vs OLA	0	Some concerns	Suspected	No concerns	Major concerns	No concerns	No concerns	Very low
ARI vs OXC	0	Some concerns	Suspected	No concerns	Major concerns	No concerns	No concerns	Very low
ARI vs PAL	0	Some concerns	Suspected	No concerns	Major concerns	No concerns	No concerns	Very low
ARI vs QUE	0	Some concerns	Suspected	No concerns	Major concerns	No concerns	No concerns	Very low
ARI vs TAM	0	Some concerns	Suspected	No concerns	Major concerns	No concerns	No concerns	Very low
ARI vs VALP	0	Some concerns	Suspected	No concerns	Major concerns	No concerns	No concerns	Very low
BRE vs CARB	0	Some concerns	Suspected	No concerns	Major concerns	No concerns	No concerns	Very low
BRE vs CARI	0	Some concerns	Suspected	No concerns	Major concerns	No concerns	No concerns	Very low
BRE vs HAL	0	Some concerns	Suspected	No concerns	Major concerns	No concerns	No concerns	Very low
BRE vs LAM	0	Some concerns	Suspected	No concerns	Major concerns	No concerns	No concerns	Very low
BRE vs LIT	0	Some concerns	Suspected	No concerns	Major concerns	No concerns	No concerns	Very low
BRE vs OLA	0	Some concerns	Suspected	No concerns	Major concerns	No concerns	No concerns	Very low
BRE vs OXC	0	Some concerns	Suspected	No concerns	Major concerns	No concerns	No concerns	Very low
BRE vs PAL	0	Some concerns	Suspected	No concerns	Major concerns	No concerns	No concerns	Very low
BRE vs QUE	0	Some concerns	Suspected	No concerns	No concerns	Major concerns	No concerns	Very low
BRE vs TAM	0	Some concerns	Suspected	No concerns	Major concerns	No concerns	No concerns	Very low
BRE vs VALP	0	Some concerns	Suspected	No concerns	Major concerns	No concerns	No concerns	Very low
CARB vs CARI	0	Some concerns	Suspected	No concerns	Major concerns	No concerns	No concerns	Very low
CARB vs HAL	0	Some concerns	Suspected	No concerns	Major concerns	No concerns	No concerns	Very low
CARB vs LAM	0	Some concerns	Suspected	No concerns	Major concerns	No concerns	No concerns	Very low
CARB vs LIT	0	Some concerns	Suspected	No concerns	Major concerns	No concerns	No concerns	Very low
CARB vs OLA	0	Some concerns	Suspected	No concerns	Major concerns	No concerns	No concerns	Very low

CARB vs OXC	0	Some concerns	Suspected	No concerns	Major concerns	No concerns	No concerns	Very low
CARB vs PAL	0	Some concerns	Suspected	No concerns	Major concerns	No concerns	No concerns	Very low
CARB vs QUE	0	Some concerns	Suspected	No concerns	Major concerns	No concerns	No concerns	Very low
CARB vs TAM	0	Some concerns	Suspected	No concerns	Major concerns	No concerns	No concerns	Very low
CARB vs VALP	0	Some concerns	Suspected	No concerns	Major concerns	No concerns	No concerns	Very low
CARI vs HAL	0	Some concerns	Suspected	No concerns	Major concerns	No concerns	No concerns	Very low
CARI vs LAM	0	Some concerns	Suspected	No concerns	Major concerns	No concerns	No concerns	Very low
CARI vs LIT	0	Some concerns	Suspected	No concerns	Major concerns	No concerns	No concerns	Very low
CARI vs OLA	0	Some concerns	Suspected	No concerns	Major concerns	No concerns	No concerns	Very low
CARI vs OXC	0	Some concerns	Suspected	No concerns	Major concerns	No concerns	No concerns	Very low
CARI vs PAL	0	Some concerns	Suspected	No concerns	Major concerns	No concerns	No concerns	Very low
CARI vs QUE	0	Some concerns	Suspected	No concerns	Major concerns	No concerns	No concerns	Very low
CARI vs TAM	0	Some concerns	Suspected	No concerns	Major concerns	No concerns	No concerns	Very low
CARI vs VALP	0	Some concerns	Suspected	No concerns	Major concerns	No concerns	No concerns	Very low
HAL vs LAM	0	Some concerns	Suspected	No concerns	Major concerns	No concerns	No concerns	Very low
HAL vs LIT	0	Some concerns	Suspected	No concerns	Major concerns	No concerns	No concerns	Very low
HAL vs OXC	0	Some concerns	Suspected	No concerns	Major concerns	No concerns	No concerns	Very low
HAL vs PAL	0	Some concerns	Suspected	No concerns	Major concerns	No concerns	No concerns	Very low
HAL vs QUE	0	Some concerns	Suspected	No concerns	Major concerns	No concerns	No concerns	Very low
HAL vs TAM	0	Some concerns	Suspected	No concerns	Major concerns	No concerns	No concerns	Very low
HAL vs VALP	0	Some concerns	Suspected	No concerns	Major concerns	No concerns	No concerns	Very low
LAM vs OLA	0	Some concerns	Suspected	No concerns	Major concerns	No concerns	No concerns	Very low
LAM vs OXC	0	Some concerns	Suspected	No concerns	Major concerns	No concerns	No concerns	Very low
LAM vs PAL	0	Some concerns	Suspected	No concerns	Major concerns	No concerns	No concerns	Very low
LAM vs QUE	0	Some concerns	Suspected	No concerns	Major concerns	No concerns	No concerns	Very low

LAM vs TAM	0	Some concerns	Suspected	No concerns	Major concerns	No concerns	No concerns	Very low
LAM vs VALP	0	Some concerns	Suspected	No concerns	Major concerns	No concerns	No concerns	Very low
LIT vs OXC	0	Some concerns	Suspected	No concerns	Major concerns	No concerns	No concerns	Very low
LIT vs PAL	0	Some concerns	Suspected	No concerns	Major concerns	No concerns	No concerns	Very low
LIT vs TAM	0	Some concerns	Suspected	No concerns	Major concerns	No concerns	No concerns	Very low
OLA vs OXC	0	Some concerns	Suspected	No concerns	Major concerns	No concerns	No concerns	Very low
OLA vs PAL	0	No concerns	Suspected	No concerns	Major concerns	No concerns	No concerns	Very low
OLA vs QUE	0	Some concerns	Suspected	No concerns	Major concerns	No concerns	No concerns	Very low
OLA vs TAM	0	No concerns	Suspected	No concerns	Major concerns	No concerns	No concerns	Very low
OXC vs PAL	0	Some concerns	Suspected	No concerns	Major concerns	No concerns	No concerns	Very low
OXC vs QUE	0	Some concerns	Suspected	No concerns	No concerns	Major concerns	No concerns	Very low
OXC vs TAM	0	Some concerns	Suspected	No concerns	Major concerns	No concerns	No concerns	Very low
OXC vs PLA	0	Some concerns	Suspected	No concerns	Major concerns	No concerns	No concerns	Very low
PAL vs TAM	0	No concerns	Suspected	No concerns	Major concerns	No concerns	No concerns	Very low
PAL vs VALP	0	No concerns	Suspected	No concerns	Major concerns	No concerns	No concerns	Very low
QUE vs TAM	0	No concerns	Suspected	No concerns	Major concerns	No concerns	No concerns	Very low
QUE vs VALP	0	Some concerns	Suspected	No concerns	No concerns	No concerns	No concerns	Very low
TAM vs VALP	0	No concerns	Suspected	No concerns	Major concerns	No concerns	No concerns	Very low

Supplementary Appendix 20. Nausea (N = 29, n = 7915)

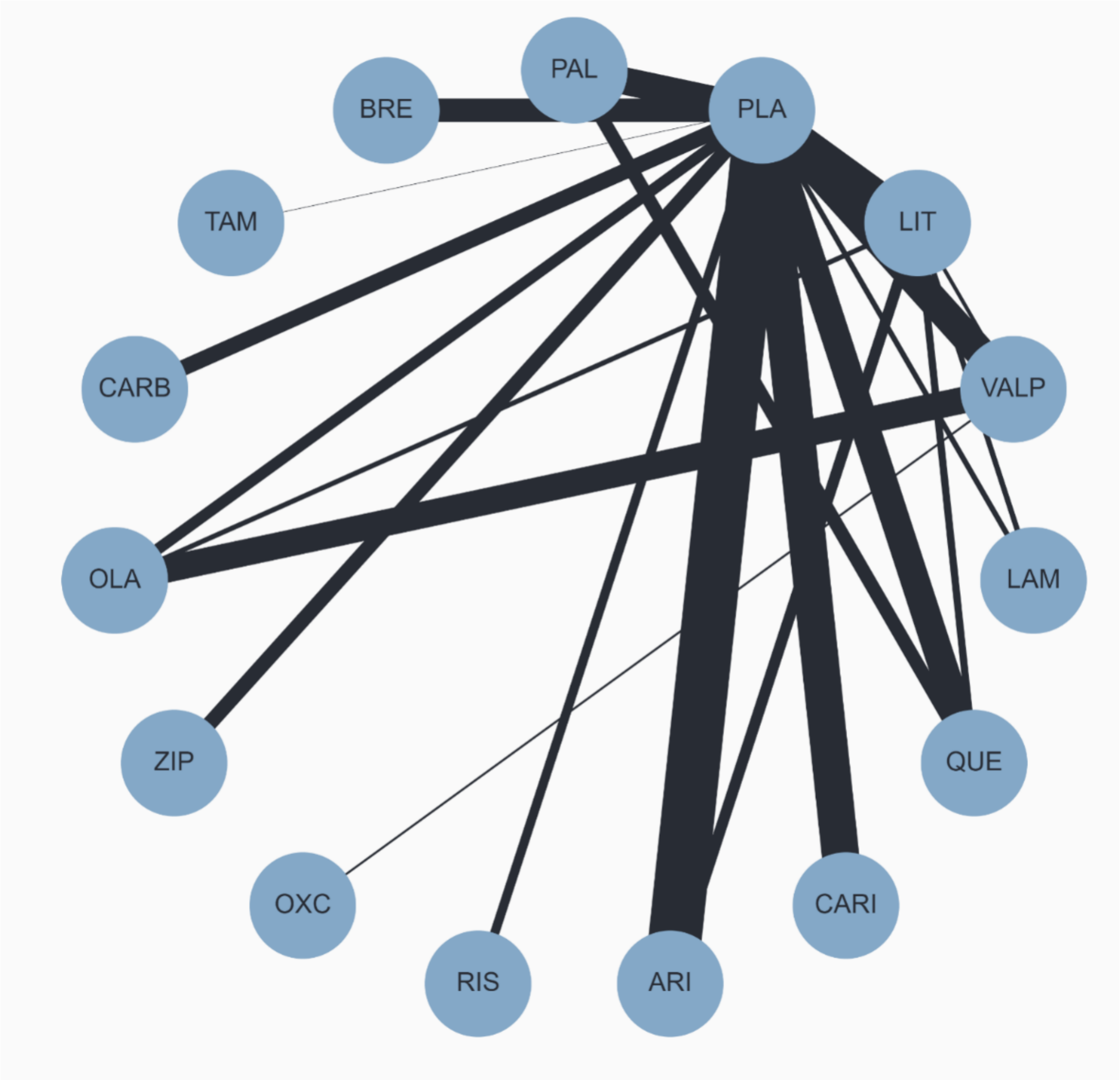

League table (risk ratio with 95% confidence interval)

ARI	2.873 (0.989, 8.344)	**0.519 (0.289, 0.929)**	0.954 (0.555, 1.641)	2.206 (0.239, 20.334)	0.882 (0.586, 1.328)	**2.621 (1.360, 5.051)**	1.299 (0.402, 4.199)	**2.905 (1.243, 6.790)**	**4.971 (1.971, 12.533)**	0.334 (0.091, 1.220)	4.675 (0.579, 37.743)	0.928 (0.594, 1.450)	1.103 (0.457, 2.661)	**1.558 (1.164, 2.085)**
	BRE	**0.181 (0.058, 0.566)**	0.332 (0.108, 1.021)	0.768 (0.067, 8.795)	**0.307 (0.103, 0.916)**	0.912 (0.277, 3.006)	0.452 (0.097, 2.100)	1.011 (0.276, 3.709)	1.730 (0.447, 6.695)	**0.116 (0.023, 0.591)**	1.627 (0.162, 16.372)	**0.323 (0.109, 0.958)**	0.384 (0.103, 1.438)	0.542 (0.195, 1.513)
		CARB	1.840 (0.930, 3.637)	4.254 (0.440, 41.137)	1.702 (0.906, 3.197)	**5.054 (2.291, 11.145)**	2.506 (0.718, 8.738)	**5.602 (2.179, 14.405)**	**9.586 (3.467, 26.499)**	0.644 (0.165, 2.509)	**9.015 (1.072, 75.793)**	1.790 (0.963, 3.325)	2.127 (0.804, 5.627)	**3.005 (1.813, 4.982)**
			CARI	2.313 (0.242, 22.136)	0.925 (0.512, 1.673)	**2.747 (1.283, 5.881)**	1.362 (0.398, 4.662)	**3.046 (1.214, 7.639)**	**5.211 (1.929, 14.078)**	0.350 (0.091, 1.341)	4.901 (0.589, 40.756)	0.973 (0.544, 1.739)	1.157 (0.448, 2.986)	**1.634 (1.034, 2.581)**
				LAM	0.400 (0.045, 3.596)	1.188 (0.122, 11.557)	0.589 (0.049, 7.029)	1.317 (0.125, 13.823)	2.253 (0.209, 24.237)	0.151 (0.012, 1.933)	2.119 (0.103, 43.785)	0.421 (0.045, 3.911)	0.500 (0.047, 5.312)	0.706 (0.077, 6.452)
					LIT	**2.970 (1.583, 5.572)**	1.472 (0.455, 4.766)	**3.292 (1.365, 7.941)**	**5.633 (2.202, 14.413)**	0.379 (0.101, 1.413)	5.298 (0.647, 43.358)	1.052 (0.670, 1.651)	1.250 (0.502, 3.113)	**1.766 (1.212, 2.574)**
						OLA	0.496 (0.148, 1.665)	1.109 (0.407, 3.021)	1.897 (0.654, 5.499)	**0.127 (0.031, 0.518)**	1.784 (0.207, 15.404)	**0.354 (0.206, 0.608)**	0.421 (0.150, 1.179)	0.595 (0.324, 1.093)
							OXC	2.236 (0.555, 9.006)	3.826 (0.906, 16.153)	0.257 (0.047, 1.411)	3.598 (0.339, 38.214)	0.714 (0.241, 2.113)	0.849 (0.207, 3.487)	1.199 (0.383, 3.759)
								PAL	1.711 (0.549, 5.336)	**0.115 (0.026, 0.512)**	1.609 (0.175, 14.767)	**0.319 (0.133, 0.766)**	0.380 (0.120, 1.201)	0.536 (0.242, 1.191)
									QUE	**0.067 (0.014, 0.314)**	0.940 (0.099, 8.910)	**0.187 (0.072, 0.482)**	**0.222 (0.066, 0.746)**	**0.313 (0.130, 0.758)**
										RIS	**13.992 (1.241, 157.811)**	2.778 (0.748, 10.315)	3.302 (0.729, 14.963)	**4.664 (1.320, 16.479)**
											TAM	0.199 (0.024, 1.619)	0.236 (0.025, 2.192)	0.333 (0.042, 2.637)

												VALP	1.189 (0.481, 2.938)	**1.679 (1.174, 2.402)**
													ZIP	1.412 (0.615, 3.242)
														PLA

Evaluation of heterogeneity and inconsistency

Between study variance (τ^2)	Heterogeneity assessment	Random-effects design-by-treatment interaction model		
		Q	df	p
0.030	Low to moderate	16.649	11	0.119

Incoherence

	NMA, RR (95% CI)	Direct, RR (95% CI)	I^2	Indirect, RR (95% CI)	Inconsistency measures	
					Difference of RR	P value
ARI vs LIT	0.882 (0.586, 1.328)	0.977 (0.575, 1.658)	na	0.760 (0.399, 1.446)	1.286 (0.559, 2.959)	0.554
ARI vs PLA	1.558 (1.164, 2.085)	1.533 (1.136, 2.069)	0.5%	2.057 (0.595, 7.114)	0.745 (0.208, 2.671)	0.652
BRE vs PLA		0.542 (0.195, 1.513)	0.0%			
CARB vs PLA		3.005 (1.813, 4.982)	0.0%			
CARI vs PLA		1.634(1.034, 2.581)	0.0%			
LAM vs LIT	0.400 (0.045, 3.596)	0.351 (0.036, 3.389)	na	2.923 (0.000, 20899.907)	0.120 (0.000, 1142.415)	0.650
LAM vs PLA	0.706 (0.077, 6.452)	1.040 (0.065, 16.678)	na	0.359 (0.009, 14.031)	2.896 (0.029, 287.034)	0.650
LIT vs OLA	2.970 (1.583, 5.572)	1.749 (0.585, 5.234)	na	3.853 (1.786, 8.310)	0.454 (0.119, 1.732)	0.248
LIT vs QUE	5.633 (2.202, 14.413)	6.551 (0.781, 54.938)	na	5.430 (1.906, 15.475)	1.206 (0.113, 12.910)	0.877
LIT vs VALP	1.052 (0.670, 1.651)	1.318 (0.631, 2.753)	na	0.919 (0.520, 1.625)	1.434 (0.565, 3.640)	0.448
LIT vs PLA	1.766 (1.212, 2.574)	1.944 (1.243, 3.043)	0.0%	1.398 (0.696, 2.807)	1.391 (0.608, 3.186)	0.435
OLA vs VALP	0.354 (0.206, 0.608)	0.298 (0.163, 0.545)	11.0%	0.701 (0.211, 2.327)	0.425 (0.111, 1.630)	0.212
OLA vs PLA	0.595 (0.324, 1.093)	0.326 (0.053, 1.982)	na	0.642 (0.337, 1.225)	0.507 (0.074, 3.452)	0.488
OXC vs VALP		0.714 (0.241, 2.113)	na			
PAL vs QUE	1.711 (0.549, 5.336)	0.990 (0.195, 5.020)	na	2.899 (0.589, 14.265)	0.341 (0.035, 3.322)	0.355
PAL vs PLA	0.536 (0.242, 1.191)	0.510 (0.229, 1.137)	88.1%	311.282 (0.036, 2721789.156)	0.002 (0.000, 14.854)	0.168
QUE vs PLA	0.313 (0.130, 0.758)	0.291 (0.118, 0.721)	45.5%	1.187 (0.025, 55.908)	0.245 (0.005, 12.833)	0.486

RIS vs PLA		4.664 (1.320, 16.479)	na			
TAM vs PLA		0.333 (0.042, 2.637)	na			
VALP vs PLA	1.679 (1.174, 2.402)	1.656 (1.132, 2.423)	0.0%	1.868 (0.650, 5.364)	0.887 (0.289, 2.722)	0.833
ZIP vs PLA		1.412 (0.615, 3.242)	26.8%			

Forest plot (vs placebo, the numbers are risk ratio with 95% confidence interval)

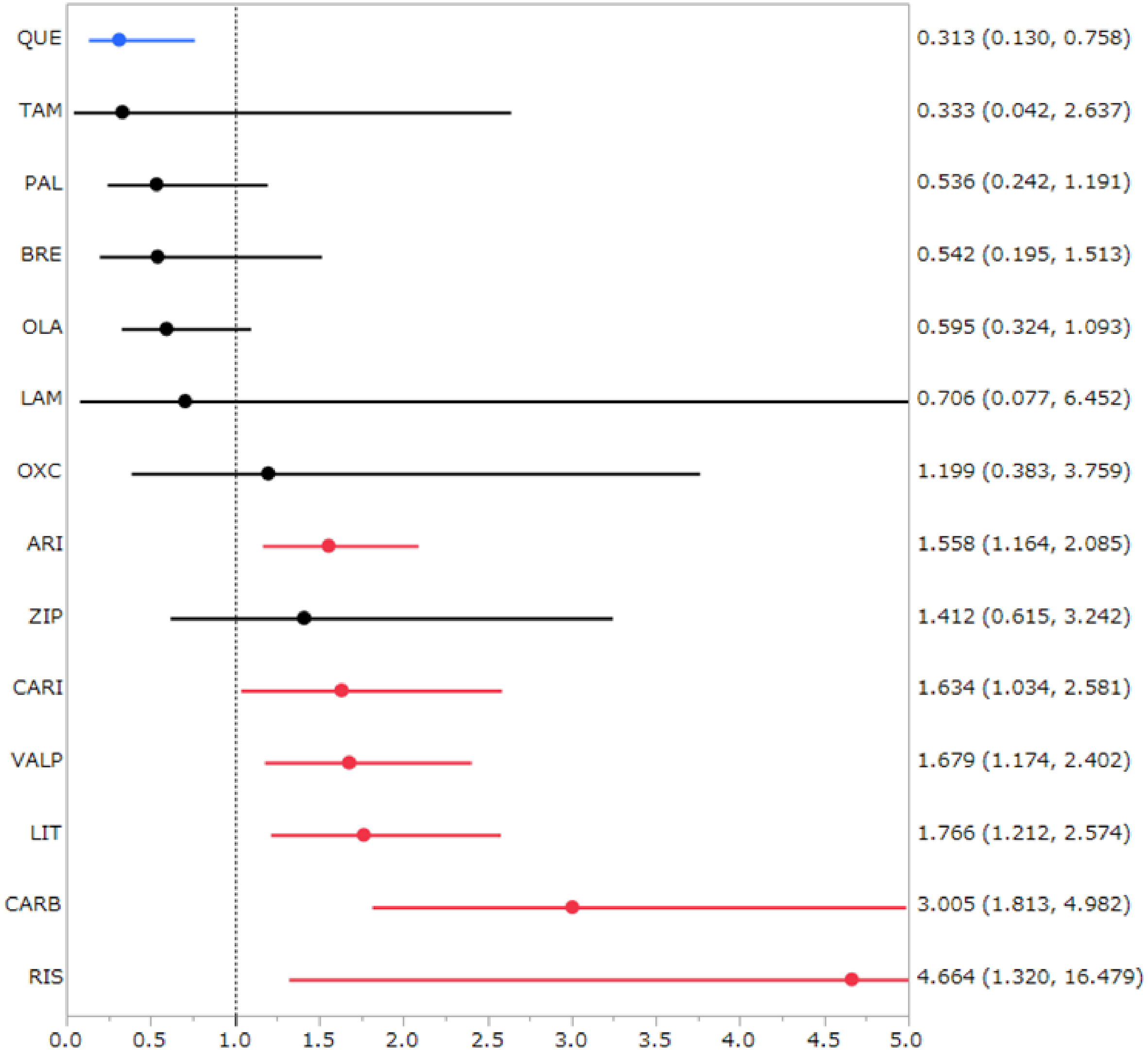

P-score

QUE	0.880
TAM	0.873
PAL	0.772
BRE	0.764
OLA	0.760
LAM	0.665
OXC	0.463
ARI	0.354
ZIP	0.351
CARI	0.345
VALP	0.280
LIT	0.278
CARB	0.079
RIS	0.059

Funnel plot (only double-blind, placebo-controlled trials)

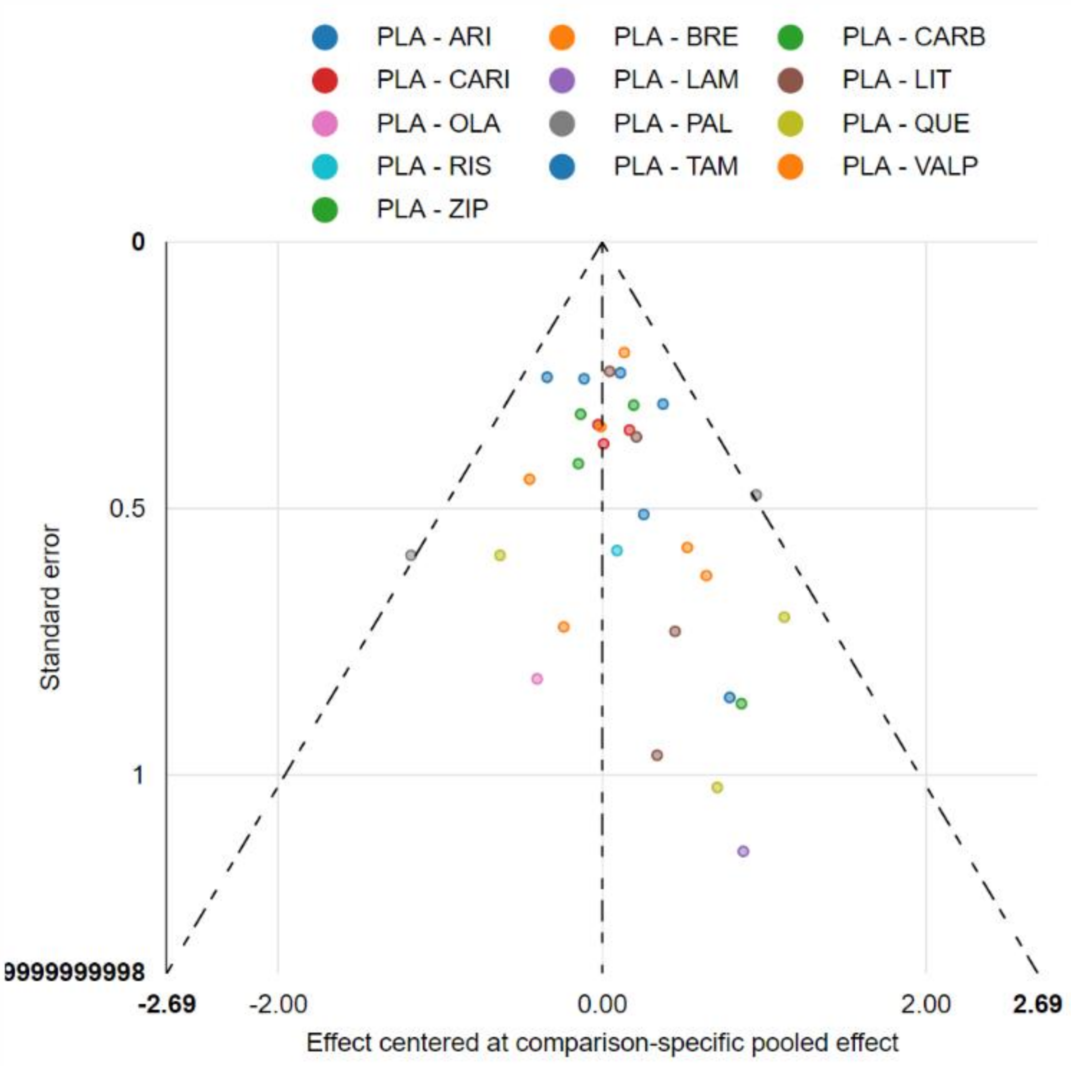

CINeMA confidence rating

Comparison	Number of studies	Within-study bias	Reporting bias	Indirectness	Imprecision	Heterogeneity	Incoherence	Confidence rating
ARI vs LIT	1	Some concerns	Suspected	No concerns	Major concerns	No concerns	No concerns	Very low
ARI vs PLA	5	Some concerns	Suspected	No concerns	No concerns	Major concerns	No concerns	Very low
BRE vs PLA	2	Some concerns	Suspected	No concerns	Major concerns	No concerns	No concerns	Very low
CARB vs PLA	2	Some concerns	Suspected	No concerns	No concerns	No concerns	No concerns	Low
CARI vs PLA	3	Some concerns	Suspected	No concerns	No concerns	Major concerns	No concerns	Very low
LAM vs LIT	1	Some concerns	Suspected	No concerns	Major concerns	No concerns	No concerns	Very low
LAM vs PLA	1	Some concerns	Suspected	No concerns	Major concerns	No concerns	No concerns	Very low
LIT vs OLA	1	No concerns	Suspected	No concerns	No concerns	No concerns	No concerns	Moderate
LIT vs QUE	1	No concerns	Suspected	No concerns	No concerns	No concerns	No concerns	Moderate
LIT vs VALP	1	Some concerns	Suspected	No concerns	Major concerns	No concerns	No concerns	Very low
LIT vs PLA	4	Some concerns	Suspected	No concerns	No concerns	No concerns	No concerns	Low
OLA vs VALP	3	No concerns	Suspected	No concerns	No concerns	No concerns	No concerns	Moderate
OLA vs PLA	1	No concerns	Suspected	No concerns	Major concerns	No concerns	No concerns	Low
OXC vs VALP	1	Some concerns	Suspected	No concerns	Major concerns	No concerns	No concerns	Very low
PAL vs QUE	1	No concerns	Suspected	No concerns	Major concerns	No concerns	No concerns	Low
PAL vs PLA	2	No concerns	Suspected	No concerns	Major concerns	No concerns	No concerns	Low
QUE vs PLA	3	No concerns	Suspected	No concerns	No concerns	No concerns	No concerns	Moderate
RIS vs PLA	1	Some concerns	Suspected	No concerns	No concerns	No concerns	No concerns	Low
TAM vs PLA	1	No concerns	Suspected	No concerns	Major concerns	No concerns	No concerns	Low
VALP vs PLA	4	No concerns	Suspected	No concerns	No concerns	Major concerns	No concerns	Low
ZIP vs PLA	2	Some concerns	Suspected	No concerns	Major concerns	No concerns	No concerns	Very low
ARI vs BRE	0	Some concerns	Suspected	No concerns	Major concerns	No concerns	No concerns	Very low
ARI vs CARB	0	Some concerns	Suspected	No concerns	No concerns	Major concerns	No concerns	Very low

ARI vs CARI	0	Some concerns	Suspected	No concerns	Major concerns	No concerns	No concerns	Very low
ARI vs LAM	0	Some concerns	Suspected	No concerns	Major concerns	No concerns	No concerns	Very low
ARI vs OLA	0	No concerns	Suspected	No concerns	No concerns	No concerns	No concerns	Low
ARI vs OXC	0	Some concerns	Suspected	No concerns	Major concerns	No concerns	No concerns	Very low
ARI vs PAL	0	No concerns	Suspected	No concerns	No concerns	No concerns	No concerns	Low
ARI vs QUE	0	No concerns	Suspected	No concerns	No concerns	No concerns	No concerns	Low
ARI vs RIS	0	Some concerns	Suspected	No concerns	Major concerns	No concerns	No concerns	Very low
ARI vs TAM	0	Some concerns	Suspected	No concerns	Major concerns	No concerns	No concerns	Very low
ARI vs VALP	0	Some concerns	Suspected	No concerns	Major concerns	No concerns	No concerns	Very low
ARI vs ZIP	0	Some concerns	Suspected	No concerns	Major concerns	No concerns	No concerns	Very low
BRE vs CARB	0	Some concerns	Suspected	No concerns	No concerns	No concerns	No concerns	Very low
BRE vs CARI	0	Some concerns	Suspected	No concerns	Major concerns	No concerns	No concerns	Very low
BRE vs LAM	0	Some concerns	Suspected	No concerns	Major concerns	No concerns	No concerns	Very low
BRE vs LIT	0	Some concerns	Suspected	No concerns	No concerns	Major concerns	No concerns	Very low
BRE vs OLA	0	No concerns	Suspected	No concerns	Major concerns	No concerns	No concerns	Very low
BRE vs OXC	0	Some concerns	Suspected	No concerns	Major concerns	No concerns	No concerns	Very low
BRE vs PAL	0	No concerns	Suspected	No concerns	Major concerns	No concerns	No concerns	Very low
BRE vs QUE	0	No concerns	Suspected	No concerns	Major concerns	No concerns	No concerns	Very low
BRE vs RIS	0	Some concerns	Suspected	No concerns	No concerns	No concerns	No concerns	Very low
BRE vs TAM	0	Some concerns	Suspected	No concerns	Major concerns	No concerns	No concerns	Very low
BRE vs VALP	0	Some concerns	Suspected	No concerns	No concerns	Major concerns	No concerns	Very low
BRE vs ZIP	0	Some concerns	Suspected	No concerns	Major concerns	No concerns	No concerns	Very low
CARB vs CARI	0	Some concerns	Suspected	No concerns	Major concerns	No concerns	No concerns	Very low
CARB vs LAM	0	Some concerns	Suspected	No concerns	Major concerns	No concerns	No concerns	Very low
CARB vs LIT	0	Some concerns	Suspected	No concerns	Major concerns	No concerns	No concerns	Very low

CARB vs OLA	0	No concerns	Suspected	No concerns	No concerns	No concerns	No concerns	Low
CARB vs OXC	0	Some concerns	Suspected	No concerns	Major concerns	No concerns	No concerns	Very low
CARB vs PAL	0	No concerns	Suspected	No concerns	No concerns	No concerns	No concerns	Low
CARB vs QUE	0	No concerns	Suspected	No concerns	No concerns	No concerns	No concerns	Low
CARB vs RIS	0	Some concerns	Suspected	No concerns	Major concerns	No concerns	No concerns	Very low
CARB vs TAM	0	Some concerns	Suspected	No concerns	No concerns	Major concerns	No concerns	Very low
CARB vs VALP	0	Some concerns	Suspected	No concerns	Major concerns	No concerns	No concerns	Very low
CARB vs ZIP	0	Some concerns	Suspected	No concerns	Major concerns	No concerns	No concerns	Very low
CARI vs LAM	0	Some concerns	Suspected	No concerns	Major concerns	No concerns	No concerns	Very low
CARI vs LIT	0	Some concerns	Suspected	No concerns	Major concerns	No concerns	No concerns	Very low
CARI vs OLA	0	No concerns	Suspected	No concerns	No concerns	No concerns	No concerns	Low
CARI vs OXC	0	Some concerns	Suspected	No concerns	Major concerns	No concerns	No concerns	Very low
CARI vs PAL	0	No concerns	Suspected	No concerns	No concerns	No concerns	No concerns	Low
CARI vs QUE	0	No concerns	Suspected	No concerns	No concerns	No concerns	No concerns	Low
CARI vs RIS	0	Some concerns	Suspected	No concerns	Major concerns	No concerns	No concerns	Very low
CARI vs TAM	0	Some concerns	Suspected	No concerns	Major concerns	No concerns	No concerns	Very low
CARI vs VALP	0	Some concerns	Suspected	No concerns	Major concerns	No concerns	No concerns	Very low
CARI vs ZIP	0	Some concerns	Suspected	No concerns	Major concerns	No concerns	No concerns	Very low
LAM vs OLA	0	Some concerns	Suspected	No concerns	Major concerns	No concerns	No concerns	Very low
LAM vs OXC	0	Some concerns	Suspected	No concerns	Major concerns	No concerns	No concerns	Very low
LAM vs PAL	0	Some concerns	Suspected	No concerns	Major concerns	No concerns	No concerns	Very low
LAM vs QUE	0	Some concerns	Suspected	No concerns	Major concerns	No concerns	No concerns	Very low
LAM vs RIS	0	Some concerns	Suspected	No concerns	Major concerns	No concerns	No concerns	Very low
LAM vs TAM	0	Some concerns	Suspected	No concerns	Major concerns	No concerns	No concerns	Very low
LAM vs VALP	0	Some concerns	Suspected	No concerns	Major concerns	No concerns	No concerns	Very low

LAM vs ZIP	0	Some concerns	Suspected	No concerns	Major concerns	No concerns	No concerns	Very low
LIT vs OXC	0	Some concerns	Suspected	No concerns	Major concerns	No concerns	No concerns	Very low
LIT vs PAL	0	No concerns	Suspected	No concerns	No concerns	No concerns	No concerns	Low
LIT vs RIS	0	Some concerns	Suspected	No concerns	Major concerns	No concerns	No concerns	Very low
LIT vs TAM	0	No concerns	Suspected	No concerns	Major concerns	No concerns	No concerns	Low
LIT vs ZIP	0	Some concerns	Suspected	No concerns	Major concerns	No concerns	No concerns	Very low
OLA vs OXC	0	Some concerns	Suspected	No concerns	Major concerns	No concerns	No concerns	Very low
OLA vs PAL	0	No concerns	Suspected	No concerns	Major concerns	No concerns	No concerns	Very low
OLA vs QUE	0	No concerns	Suspected	No concerns	Major concerns	No concerns	No concerns	Very low
OLA vs RIS	0	No concerns	Suspected	No concerns	No concerns	No concerns	No concerns	Low
OLA vs TAM	0	No concerns	Suspected	No concerns	Major concerns	No concerns	No concerns	Very low
OLA vs ZIP	0	No concerns	Suspected	No concerns	Major concerns	No concerns	No concerns	Very low
OXC vs PAL	0	No concerns	Suspected	No concerns	Major concerns	No concerns	No concerns	Very low
OXC vs QUE	0	No concerns	Suspected	No concerns	Major concerns	No concerns	No concerns	Very low
OXC vs RIS	0	Some concerns	Suspected	No concerns	Major concerns	No concerns	No concerns	Very low
OXC vs TAM	0	No concerns	Suspected	No concerns	Major concerns	No concerns	No concerns	Very low
OXC vs ZIP	0	Some concerns	Suspected	No concerns	Major concerns	No concerns	No concerns	Very low
OXC vs PLA	0	Some concerns	Suspected	No concerns	Major concerns	No concerns	No concerns	Very low
PAL vs RIS	0	No concerns	Suspected	No concerns	No concerns	No concerns	No concerns	Low
PAL vs TAM	0	No concerns	Suspected	No concerns	Major concerns	No concerns	No concerns	Very low
PAL vs VALP	0	No concerns	Suspected	No concerns	No concerns	No concerns	No concerns	Low
PAL vs ZIP	0	No concerns	Suspected	No concerns	Major concerns	No concerns	No concerns	Very low
QUE vs RIS	0	No concerns	Suspected	No concerns	No concerns	No concerns	No concerns	Low
QUE vs TAM	0	No concerns	Suspected	No concerns	Major concerns	No concerns	No concerns	Very low
QUE vs VALP	0	No concerns	Suspected	No concerns	No concerns	No concerns	No concerns	Low

QUE vs ZIP	0	No concerns	Suspected	No concerns	No concerns	No concerns	No concerns	Low
RIS vs TAM	0	Some concerns	Suspected	No concerns	No concerns	No concerns	No concerns	Very low
RIS vs VALP	0	Some concerns	Suspected	No concerns	Major concerns	No concerns	No concerns	Very low
RIS vs ZIP	0	Some concerns	Suspected	No concerns	Major concerns	No concerns	No concerns	Very low
TAM vs VALP	0	No concerns	Suspected	No concerns	Major concerns	No concerns	No concerns	Very low
TAM vs ZIP	0	No concerns	Suspected	No concerns	Major concerns	No concerns	No concerns	Very low
VALP vs ZIP	0	Some concerns	Suspected	No concerns	Major concerns	No concerns	No concerns	Very low

Supplementary Appendix 21. Weight gain (N = 31, n = 8704)

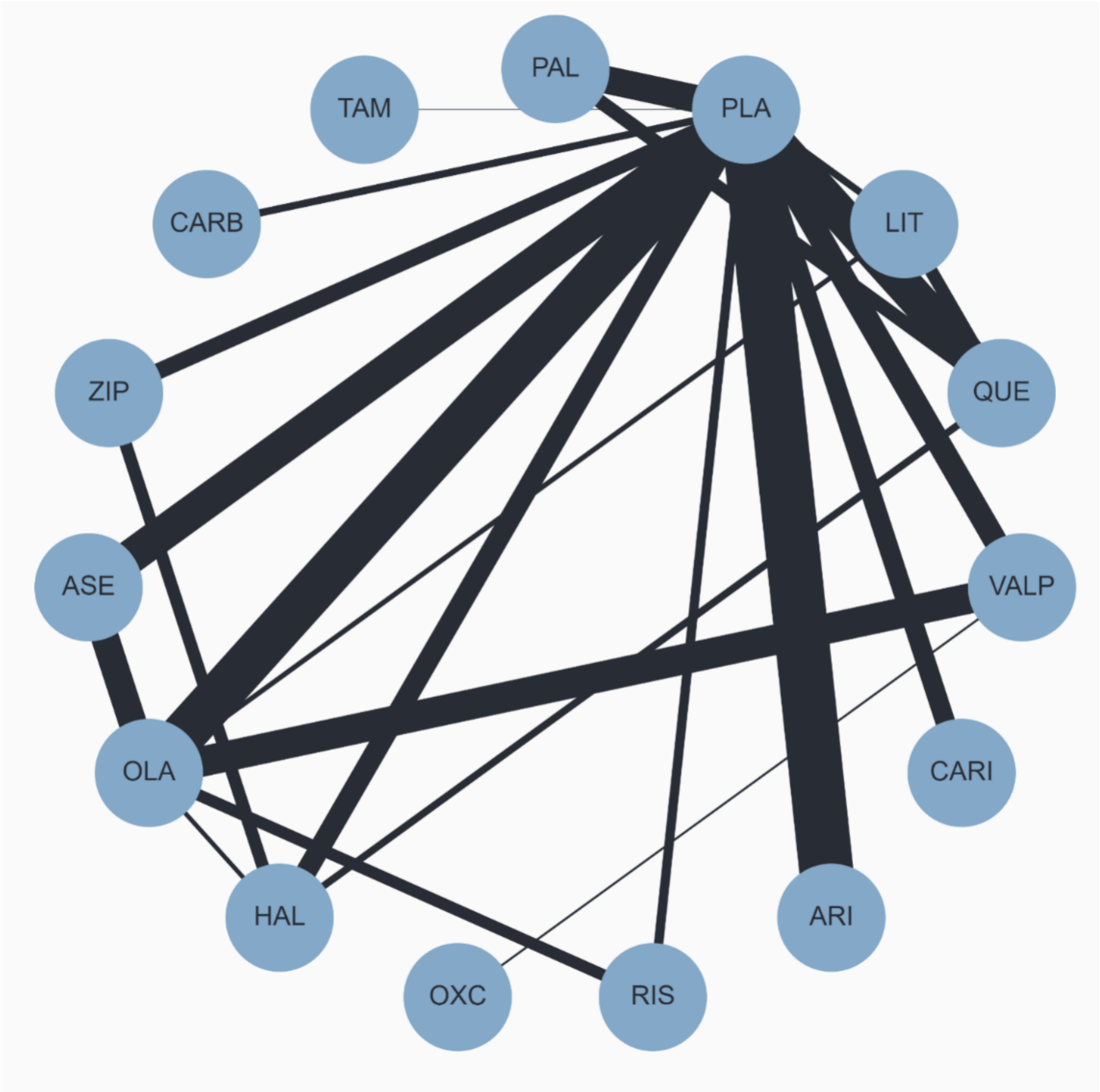

League table (risk ratio with 95% confidence interval)

ARI	**0.187 (0.078, 0.451)**	0.664 (0.013, 34.155)	0.447 (0.102, 1.961)	**0.374 (0.150, 0.931)**	**0.361 (0.132, 0.986)**	**0.083 (0.038, 0.181)**	1.186 (0.060, 23.474)	**0.227 (0.075, 0.684)**	**0.165 (0.072, 0.376)**	**0.322 (0.106, 0.977)**	2.031 (0.092, 44.844)	**0.132 (0.060, 0.291)**	**0.231 (0.087, 0.612)**	0.677 (0.416, 1.102)
	ASE	3.541 (0.066, 189.199)	2.384 (0.493, 11.533)	1.996 (0.731, 5.450)	1.928 (0.661, 5.627)	**0.441 (0.286, 0.681)**	6.324 (0.340, 117.648)	1.208 (0.361, 4.039)	0.878 (0.343, 2.248)	1.717 (0.677, 4.349)	10.834 (0.468, 250.937)	0.703 (0.421, 1.172)	1.233 (0.419, 3.632)	**3.611 (1.737, 7.507)**
		CARB	0.673 (0.011, 42.790)	0.564 (0.010, 30.332)	0.544 (0.010, 29.952)	0.125 (0.002, 6.530)	1.786 (0.013, 238.770)	0.341 (0.006, 19.275)	0.248 (0.005, 13.095)	0.485 (0.009, 27.423)	3.059 (0.021, 437.510)	0.198 (0.004, 10.410)	0.348 (0.006, 19.022)	1.020 (0.020, 50.902)
			CARI	0.837 (0.170, 4.126)	0.809 (0.155, 4.209)	**0.185 (0.040, 0.852)**	2.653 (0.102, 69.103)	0.507 (0.091, 2.810)	0.368 (0.078, 1.731)	0.720 (0.129, 4.007)	4.545 (0.158, 130.856)	0.295 (0.064, 1.362)	0.517 (0.101, 2.645)	1.515 (0.375, 6.121)
				HAL	0.966 (0.353, 2.644)	**0.221 (0.088, 0.555)**	3.168 (0.154, 65.301)	0.605 (0.191, 1.915)	**0.440 (0.196, 0.985)**	0.860 (0.255, 2.896)	5.428 (0.232, 126.875)	**0.352 (0.138, 0.895)**	0.618 (0.304, 1.256)	1.809 (0.837, 3.908)
					LIT	**0.229 (0.085, 0.617)**	3.280 (0.156, 69.186)	0.627 (0.205, 1.915)	**0.455 (0.236, 0.879)**	0.890 (0.250, 3.172)	5.620 (0.234, 135.098)	**0.364 (0.133, 0.996)**	0.640 (0.210, 1.954)	1.873 (0.778, 4.507)
						OLA	14.323 (0.795, 258.088)	2.737 (0.876, 8.550)	1.989 (0.850, 4.654)	**3.888 (1.706, 8.860)**	**24.540 (1.086, 554.408)**	**1.592 (1.209, 2.095)**	**2.794 (1.024, 7.619)**	**8.180 (4.419, 15.142)**
							OXC	0.191 (0.009, 4.235)	0.139 (0.007, 2.802)	0.271 (0.013, 5.483)	1.713 (0.025, 119.474)	0.111 (0.006, 1.976)	0.195 (0.009, 4.125)	0.571 (0.030, 10.864)

								PAL	0.727 (0.289, 1.830)	1.421 (0.355, 5.678)	8.967 (0.361, 222.850)	0.582 (0.185, 1.832)	1.021 (0.297, 3.511)	**2.989 (1.109, 8.058)**
									QUE	1.955 (0.612, 6.243)	12.338 (0.540, 281.688)	0.800 (0.338, 1.897)	1.405 (0.546, 3.618)	**4.113 (2.111, 8.012)**
										RIS	6.312 (0.253, 157.181)	**0.409 (0.172, 0.973)**	0.719 (0.200, 2.576)	2.104 (0.775, 5.708)
											TAM	0.065 (0.003, 1.468)	0.114 (0.005, 2.712)	0.333 (0.016, 7.082)
												VALP	1.755 (0.637, 4.836)	**5.140 (2.746, 9.620)**
													ZIP	**2.928 (1.259, 6.807)**
														PLA

Evaluation of heterogeneity and inconsistency

Between study variance (τ^2)	Heterogeneity assessment	Random-effects design-by-treatment interaction model		
		Q	df	p
0.000	Low	12.598	16	0.702

Incoherence

	NMA, RR (95% CI)	Direct, RR (95% CI)	I^2	Indirect, RR (95% CI)	Inconsistency measures	
					Difference of RR	P value
ARI vs PLA		0.677(0.416,1.102)	0.0%			
ASE vs OLA	0.441 (0.286, 0.681)	0.423 (0.272, 0.656)	0.0%	2.403 (0.153, 37.728)	0.176 (0.011, 2.862)	0.222
ASE vs PLA	3.611 (1.737, 7.507)	8.973 (2.168, 37.136)	0.0%	2.599 (1.106, 6.104)	3.453 (0.658, 18.111)	0.143
CARB vs PLA		1.020 (0.020, 50.902)	na			
CARI vs PLA		1.515 (0.375, 6.121)	24.8%			
HAL vs OLA	0.221 (0.088, 0.555)	0.789 (0.103, 6.056)	na	0.160 (0.057, 0.448)	4.947 (0.504, 48.550)	0.170
HAL vs QUE	0.440 (0.196, 0.985)	0.396 (0.147, 1.070)	na	0.538 (0.135, 2.140)	0.736 (0.134, 4.035)	0.724
HAL vs ZIP	0.618 (0.304, 1.256)	0.551 (0.253, 1.202)	na	1.068 (0.194, 5.883)	0.516 (0.079, 3.367)	0.489
HAL vs PLA	1.809 (0.837, 3.908)	1.863 (0.741, 4.681)	0.0%	1.690 (0.415, 6.881)	1.102 (0.206, 5.910)	0.909
LIT vs OLA	0.229 (0.085, 0.617)	0.088 (0.012, 0.666)	na	0.310 (0.099, 0.968)	0.285 (0.028, 2.898)	0.289
LIT vs QUE	0.455 (0.236, 0.879)	0.490 (0.246, 0.975)	0.0%	0.215 (0.024, 1.953)	2.283 (0.226, 23.074)	0.484
LIT vs PLA	1.873 (0.778, 4.507)	5.939 (0.728, 48.415)	na	1.466 (0.558, 3.855)	4.050 (0.402, 40.817)	0.235
OLA vs RIS	3.888 (1.706, 8.860)	4.473 (1.897, 10.547)	na	0.739 (0.039, 14.158)	6.051 (0.280, 130.974)	0.251
OLA vs VALP	1.592 (1.209, 2.095)	1.528 (1.153, 2.025)	0.0%	3.702 (1.030, 13.312)	0.413 (0.111, 1.530)	0.185
OLA vs PLA	8.180 (4.419, 15.142)	10.197 (3.931, 26.454)	0.0%	6.985 (3.117, 15.650)	1.460 (0.419, 5.090)	0.553
OXC vs VALP		0.111 (0.006, 1.976)	na			
PAL vs QUE	0.727 (0.289, 1.830)	0.742 (0.262, 2.099)	na	0.671 (0.090, 5.016)	1.106 (0.115, 10.639	0.931

PAL vs PLA	2.989 (1.109, 8.058)	2.405 (0.691, 8.365)	0.0%	4.348 (0.846, 22.356)	0.553 (0.071, 4.330)	0.573
QUE vs PLA	4.113 (2.111, 8.012)	4.163 (1.910, 9.075)	12.0%	3.978 (1.095, 14.447)	1.047 (0.232, 4.723)	0.953
RIS vs PLA	2.104 (0.775, 5.708)	10.264 (0.573, 183.736)	na	1.696 (0.585, 4.914)	6.051 (0.280, 130.974)	0.251
TAM vs PLA		0.333 (0.016, 7.082)	na			
VALP vs PLA	5.140 (2.746, 9.620)	3.114 (1.282, 7.562)	0.0%	8.466 (3.492, 20.526)	0.368 (0.105, 1.288)	0.118
ZIP vs PLA	2.928 (1.259, 6.807)	2.554 (0.883, 7.389)	0.0%	3.697 (0.922, 14.819)	0.691 (0.120, 3.968)	0.678

Forest plot (vs placebo, the numbers are risk ratio with 95% confidence interval)

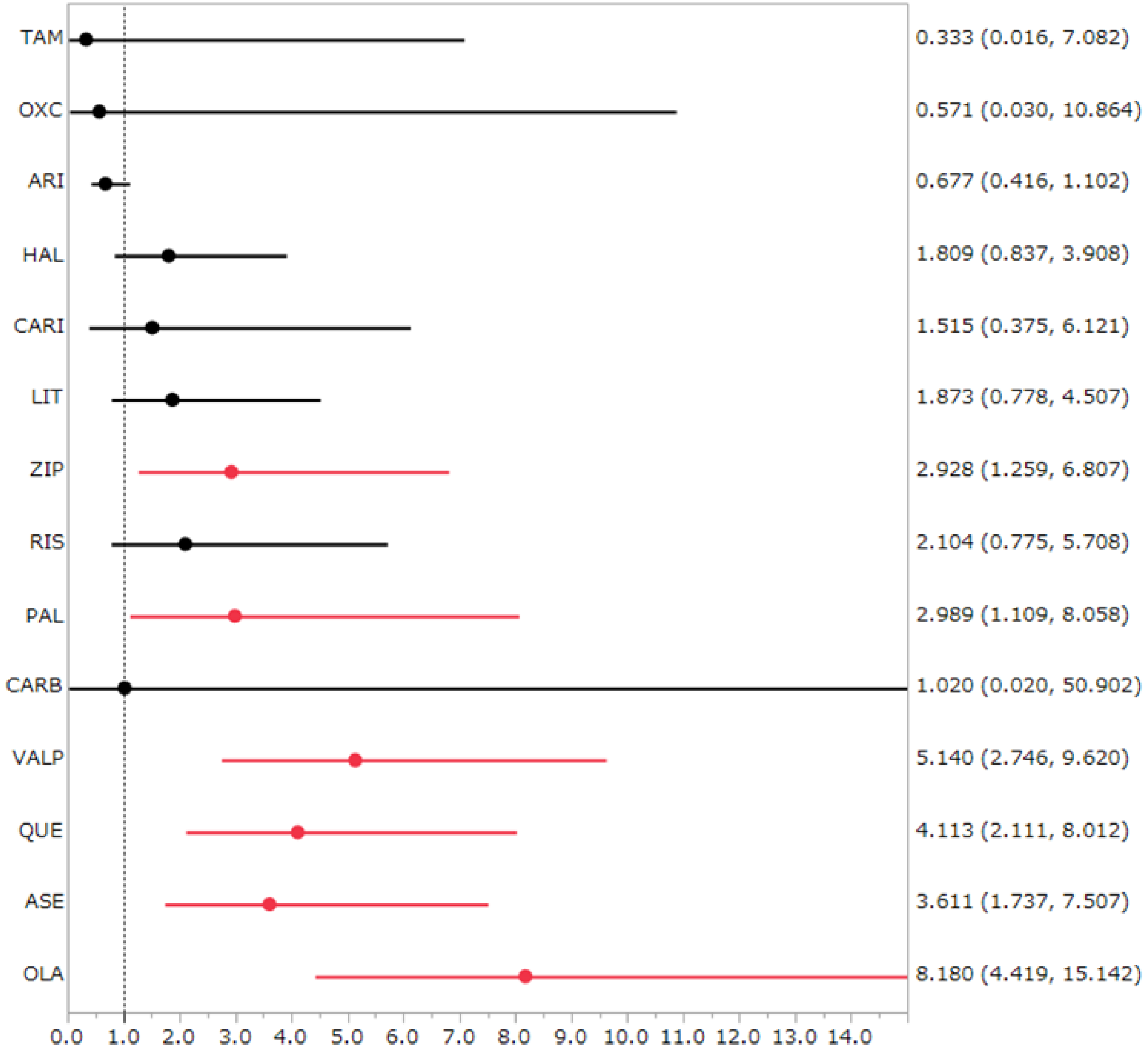

P-score

TAM	0.971
OXC	0.940
ARI	0.819
HAL	0.597
CARI	0.556
LIT	0.543
ZIP	0.438
RIS	0.402
PAL	0.395
CARB	0.293
VALP	0.255
QUE	0.255
ASE	0.229
OLA	0.052

Funnel plot (only double-blind, placebo-controlled trials)

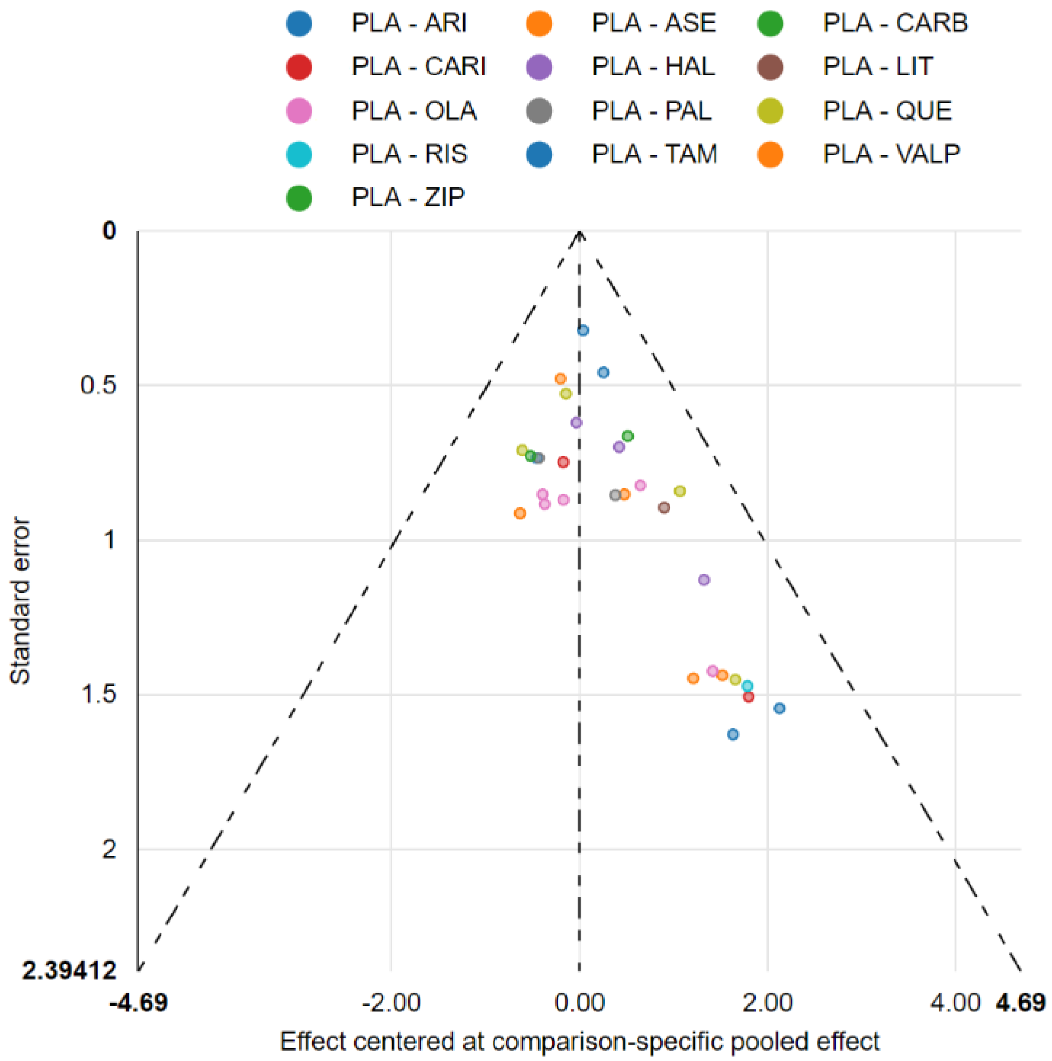

CINeMA confidence rating

Comparison	Number of studies	Within-study bias	Reporting bias	Indirectness	Imprecision	Heterogeneity	Incoherence	Confidence rating
ARI vs PLA	5	Some concerns	Suspected	No concerns	Major concerns	No concerns	No concerns	Very low
ASE vs OLA	2	Some concerns	Suspected	No concerns	No concerns	No concerns	No concerns	Low
ASE vs PLA	3	Some concerns	Suspected	No concerns	No concerns	No concerns	No concerns	Low
CARB vs PLA	1	Some concerns	Suspected	No concerns	Major concerns	No concerns	No concerns	Very low
CARI vs PLA	2	Some concerns	Suspected	No concerns	Major concerns	No concerns	No concerns	Very low
HAL vs OLA	1	Some concerns	Suspected	No concerns	No concerns	No concerns	No concerns	Low
HAL vs QUE	1	Some concerns	Suspected	No concerns	No concerns	Major concerns	No concerns	Very low
HAL vs ZIP	1	Some concerns	Suspected	No concerns	Major concerns	No concerns	No concerns	Very low
HAL vs PLA	3	Some concerns	Suspected	No concerns	Major concerns	No concerns	No concerns	Very low
LIT vs OLA	1	No concerns	Suspected	No concerns	No concerns	No concerns	No concerns	Moderate
LIT vs QUE	2	No concerns	Suspected	No concerns	No concerns	No concerns	No concerns	Moderate
LIT vs PLA	1	No concerns	Suspected	No concerns	Major concerns	No concerns	No concerns	Low
OLA vs RIS	1	Some concerns	Suspected	No concerns	No concerns	No concerns	No concerns	Low
OLA vs VALP	4	No concerns	Suspected	No concerns	No concerns	No concerns	No concerns	Moderate
OLA vs PLA	5	Some concerns	Suspected	No concerns	No concerns	No concerns	No concerns	Low
OXC vs VALP	1	Some concerns	Suspected	No concerns	Major concerns	No concerns	No concerns	Very low
PAL vs QUE	1	No concerns	Suspected	No concerns	Major concerns	No concerns	No concerns	Low
PAL vs PLA	2	No concerns	Suspected	No concerns	No concerns	No concerns	No concerns	Moderate
QUE vs PLA	4	Some concerns	Suspected	No concerns	No concerns	No concerns	No concerns	Low
RIS vs PLA	1	Some concerns	Suspected	No concerns	Major concerns	No concerns	No concerns	Very low
TAM vs PLA	1	No concerns	Suspected	No concerns	Major concerns	No concerns	No concerns	Low
VALP vs PLA	2	No concerns	Suspected	No concerns	No concerns	No concerns	No concerns	Moderate
ZIP vs PLA	2	Some concerns	Suspected	No concerns	No concerns	No concerns	No concerns	Low

ARI vs ASE	0	Some concerns	Suspected	No concerns	No concerns	No concerns	No concerns	Very low
ARI vs CARB	0	Some concerns	Suspected	No concerns	Major concerns	No concerns	No concerns	Very low
ARI vs CARI	0	Some concerns	Suspected	No concerns	Major concerns	No concerns	No concerns	Very low
ARI vs HAL	0	Some concerns	Suspected	No concerns	No concerns	No concerns	No concerns	Very low
ARI vs LIT	0	Some concerns	Suspected	No concerns	No concerns	Major concerns	No concerns	Very low
ARI vs OLA	0	Some concerns	Suspected	No concerns	No concerns	No concerns	No concerns	Very low
ARI vs OXC	0	Some concerns	Suspected	No concerns	Major concerns	No concerns	No concerns	Very low
ARI vs PAL	0	Some concerns	Suspected	No concerns	No concerns	No concerns	No concerns	Very low
ARI vs QUE	0	Some concerns	Suspected	No concerns	No concerns	No concerns	No concerns	Very low
ARI vs RIS	0	Some concerns	Suspected	No concerns	No concerns	Major concerns	No concerns	Very low
ARI vs TAM	0	Some concerns	Suspected	No concerns	Major concerns	No concerns	No concerns	Very low
ARI vs VALP	0	Some concerns	Suspected	No concerns	No concerns	No concerns	No concerns	Very low
ARI vs ZIP	0	Some concerns	Suspected	No concerns	No concerns	No concerns	No concerns	Very low
ASE vs CARB	0	Some concerns	Suspected	No concerns	Major concerns	No concerns	No concerns	Very low
ASE vs CARI	0	Some concerns	Suspected	No concerns	Major concerns	No concerns	No concerns	Very low
ASE vs HAL	0	Some concerns	Suspected	No concerns	Major concerns	No concerns	No concerns	Very low
ASE vs LIT	0	Some concerns	Suspected	No concerns	Major concerns	No concerns	No concerns	Very low
ASE vs OXC	0	Some concerns	Suspected	No concerns	Major concerns	No concerns	No concerns	Very low
ASE vs PAL	0	No concerns	Suspected	No concerns	Major concerns	No concerns	No concerns	Very low
ASE vs QUE	0	Some concerns	Suspected	No concerns	Major concerns	No concerns	No concerns	Very low
ASE vs RIS	0	Some concerns	Suspected	No concerns	Major concerns	No concerns	No concerns	Very low
ASE vs TAM	0	No concerns	Suspected	No concerns	Major concerns	No concerns	No concerns	Very low
ASE vs VALP	0	Some concerns	Suspected	No concerns	Major concerns	No concerns	No concerns	Very low
ASE vs ZIP	0	Some concerns	Suspected	No concerns	Major concerns	No concerns	No concerns	Very low
CARB vs CARI	0	Some concerns	Suspected	No concerns	Major concerns	No concerns	No concerns	Very low

CARB vs HAL	0	Some concerns	Suspected	No concerns	Major concerns	No concerns	No concerns	Very low
CARB vs LIT	0	Some concerns	Suspected	No concerns	Major concerns	No concerns	No concerns	Very low
CARB vs OLA	0	Some concerns	Suspected	No concerns	Major concerns	No concerns	No concerns	Very low
CARB vs OXC	0	Some concerns	Suspected	No concerns	Major concerns	No concerns	No concerns	Very low
CARB vs PAL	0	Some concerns	Suspected	No concerns	Major concerns	No concerns	No concerns	Very low
CARB vs QUE	0	Some concerns	Suspected	No concerns	Major concerns	No concerns	No concerns	Very low
CARB vs RIS	0	Some concerns	Suspected	No concerns	Major concerns	No concerns	No concerns	Very low
CARB vs TAM	0	Some concerns	Suspected	No concerns	Major concerns	No concerns	No concerns	Very low
CARB vs VALP	0	Some concerns	Suspected	No concerns	Major concerns	No concerns	No concerns	Very low
CARB vs ZIP	0	Some concerns	Suspected	No concerns	Major concerns	No concerns	No concerns	Very low
CARI vs HAL	0	Some concerns	Suspected	No concerns	Major concerns	No concerns	No concerns	Very low
CARI vs LIT	0	Some concerns	Suspected	No concerns	Major concerns	No concerns	No concerns	Very low
CARI vs OLA	0	Some concerns	Suspected	No concerns	No concerns	No concerns	No concerns	Very low
CARI vs OXC	0	Some concerns	Suspected	No concerns	Major concerns	No concerns	No concerns	Very low
CARI vs PAL	0	Some concerns	Suspected	No concerns	Major concerns	No concerns	No concerns	Very low
CARI vs QUE	0	Some concerns	Suspected	No concerns	Major concerns	No concerns	No concerns	Very low
CARI vs RIS	0	Some concerns	Suspected	No concerns	Major concerns	No concerns	No concerns	Very low
CARI vs TAM	0	Some concerns	Suspected	No concerns	Major concerns	No concerns	No concerns	Very low
CARI vs VALP	0	Some concerns	Suspected	No concerns	Major concerns	No concerns	No concerns	Very low
CARI vs ZIP	0	Some concerns	Suspected	No concerns	Major concerns	No concerns	No concerns	Very low
HAL vs LIT	0	Some concerns	Suspected	No concerns	Major concerns	No concerns	No concerns	Very low
HAL vs OXC	0	Some concerns	Suspected	No concerns	Major concerns	No concerns	No concerns	Very low
HAL vs PAL	0	No concerns	Suspected	No concerns	Major concerns	No concerns	No concerns	Very low
HAL vs RIS	0	Some concerns	Suspected	No concerns	Major concerns	No concerns	No concerns	Very low
HAL vs TAM	0	No concerns	Suspected	No concerns	Major concerns	No concerns	No concerns	Very low

HAL vs VALP	0	Some concerns	Suspected	No concerns	No concerns	No concerns	No concerns	Very low
LIT vs OXC	0	No concerns	Suspected	No concerns	Major concerns	No concerns	No concerns	Very low
LIT vs PAL	0	No concerns	Suspected	No concerns	Major concerns	No concerns	No concerns	Very low
LIT vs RIS	0	Some concerns	Suspected	No concerns	Major concerns	No concerns	No concerns	Very low
LIT vs TAM	0	No concerns	Suspected	No concerns	Major concerns	No concerns	No concerns	Very low
LIT vs VALP	0	No concerns	Suspected	No concerns	No concerns	Major concerns	No concerns	Very low
LIT vs ZIP	0	Some concerns	Suspected	No concerns	Major concerns	No concerns	No concerns	Very low
OLA vs OXC	0	Some concerns	Suspected	No concerns	Major concerns	No concerns	No concerns	Very low
OLA vs PAL	0	No concerns	Suspected	No concerns	Major concerns	No concerns	No concerns	Very low
OLA vs QUE	0	Some concerns	Suspected	No concerns	Major concerns	No concerns	No concerns	Very low
OLA vs TAM	0	No concerns	Suspected	No concerns	No concerns	Major concerns	No concerns	Very low
OLA vs ZIP	0	Some concerns	Suspected	No concerns	No concerns	Major concerns	No concerns	Very low
OXC vs PAL	0	No concerns	Suspected	No concerns	Major concerns	No concerns	No concerns	Very low
OXC vs QUE	0	Some concerns	Suspected	No concerns	Major concerns	No concerns	No concerns	Very low
OXC vs RIS	0	Some concerns	Suspected	No concerns	Major concerns	No concerns	No concerns	Very low
OXC vs TAM	0	No concerns	Suspected	No concerns	Major concerns	No concerns	No concerns	Very low
OXC vs ZIP	0	Some concerns	Suspected	No concerns	Major concerns	No concerns	No concerns	Very low
OXC vs PLA	0	Some concerns	Suspected	No concerns	Major concerns	No concerns	No concerns	Very low
PAL vs RIS	0	Some concerns	Suspected	No concerns	Major concerns	No concerns	No concerns	Very low
PAL vs TAM	0	No concerns	Suspected	No concerns	Major concerns	No concerns	No concerns	Very low
PAL vs VALP	0	No concerns	Suspected	No concerns	Major concerns	No concerns	No concerns	Very low
PAL vs ZIP	0	No concerns	Suspected	No concerns	Major concerns	No concerns	No concerns	Very low
QUE vs RIS	0	Some concerns	Suspected	No concerns	Major concerns	No concerns	No concerns	Very low
QUE vs TAM	0	No concerns	Suspected	No concerns	Major concerns	No concerns	No concerns	Very low
QUE vs VALP	0	No concerns	Suspected	No concerns	Major concerns	No concerns	No concerns	Very low

QUE vs ZIP	0	Some concerns	Suspected	No concerns	Major concerns	No concerns	No concerns	Very low
RIS vs TAM	0	No concerns	Suspected	No concerns	Major concerns	No concerns	No concerns	Very low
RIS vs VALP	0	Some concerns	Suspected	No concerns	No concerns	Major concerns	No concerns	Very low
RIS vs ZIP	0	Some concerns	Suspected	No concerns	Major concerns	No concerns	No concerns	Very low
TAM vs VALP	0	No concerns	Suspected	No concerns	Major concerns	No concerns	No concerns	Very low
TAM vs ZIP	0	No concerns	Suspected	No concerns	Major concerns	No concerns	No concerns	Very low
VALP vs ZIP	0	No concerns	Suspected	No concerns	Major concerns	No concerns	No concerns	Very low
